# Evaluation of Comparative Efficacy and Safety of Surgical Approaches for Total Hip Arthroplasty: A Systematic Review and Network Meta-analysis

**DOI:** 10.1001/jamanetworkopen.2022.53942

**Published:** 2023-01-31

**Authors:** Lei Yan, Long Ge, Shengjie Dong, Kiran Saluja, Dijun Li, K. Srikanth Reddy, Qi Wang, Liang Yao, Jiao Jiao Li, Bruno Roza da Costa, Dan Xing, Bin Wang

**Affiliations:** 1 Department of Orthopaedic Surgery, The First Affiliated Hospital, Zhejiang University School of Medicine, Hangzhou, China; 2 Second Clinical Medical College, Shanxi Medical University, Taiyuan, China; 3 Department of Orthopedics, The Second Hospital of Shanxi Medical University, Taiyuan, China; 4 Evidence Based Social Science Research Centre, School of Public Health, Lanzhou University, Lanzhou, China; 5 Department of Social Medicine and Health Management, School of Public Health, Lanzhou University, Lanzhou, China; 6 Department of Health Research Methods, Evidence and Impact, McMaster University, Hamilton, Ontario, Canada; 7 Orthopedic Department, Yantaishan Hospital, Yantai, China; 8 Bruyere Research Institute, University of Ottawa, Ottawa, Ontario, Canada; 9 School of Epidemiology and Public Health, Faculty of Medicine, University of Ottawa, Ottawa, Ontario, Canada; 10 World Health Organization, Geneva, Switzerland; 11 Health Policy PhD Program and McMaster Health Forum, McMaster University, Hamilton, Ontario, Canada; 12 Department of Health Research Methods, Evidence, and Impact, Faculty of Health Sciences, McMaster University, Hamilton, Ontario, Canada; 13 School of Biomedical Engineering, Faculty of Engineering and IT, University of Technology Sydney, Sydney, New South Wales, Australia; 14 Institute of Health Policy, Management, and Evaluation, Department of Medicine, University of Toronto, Toronto, Ontario, Canada; 15 Applied Health Research Centre (AHRC), Li Ka Shing Knowledge Institute of St Michael's Hospital, Toronto, Ontario, Canada; 16 Arthritis Clinic & Research Center, Peking University People’s Hospital, Peking University, Beijing, China

## Abstract

**Importance:**

Each approach for primary total hip arthroplasty (THA) has a long learning curve, so a surgeon’s choice to change their preferred approach needs to be guided by clear justifications. However, current evidence does not suggest that any of the THA approaches are more beneficial than others, and the choice of approach is mainly based on the knowledge and experience of the surgeon and individual patient characteristics.

**Objective:**

To assess the efficacy and safety associated with different surgical approaches for THA.

**Data Sources:**

A comprehensive search of PubMed, EMBASE, and Cochrane databases from inception to March 26, 2022; reference lists of eligible trials; and related reviews.

**Study Selection:**

Randomized clinical trials (RCTs) comparing different surgical approaches, including the 2-incision approach, direct anterior approach (DAA), direct lateral approach (DLA), minimally invasive direct lateral approach (MIS-DLA), minimally invasive anterolateral approach (MIS-ALA), posterior approach (PA), minimally invasive posterior approach (MIS-PA), and supercapsular percutaneously assisted total hip arthroplasty (SuperPath), for primary THA.

**Data Extraction and Synthesis:**

Following the Preferred Reporting Items for Systematic Reviews and Meta-Analyses, 2 reviewers independently extracted data on study participants, interventions, and outcomes as well as assessed the risk of bias using the Cochrane risk of bias tool and the certainty of evidence using the Grading of Recommendations, Assessment, Development, and Evaluation framework. A frequentist framework was used to inform a series of random-effects network meta-analyses.

**Main Outcomes and Measures:**

The outcomes were hip score (range, 0-100, with higher scores indicating better overall hip condition), pain score (range, 0-100, with higher scores indicating more pain), hospitalization time, operation time, quality of life score, blood loss, cup abduction angle, and cup anteversion angle.

**Results:**

Of 2130 retrieved studies, 63 RCTs including 4859 participants (median [IQR] age, 64.0 [60.3-66.5] years; median [IQR] percentage male, 46.74% [38.64%-54.74%]) were eligible for analysis. Eight surgical approaches were evaluated. For hip score, DAA (mean difference [MD], 4.04; 95% CI, 1.92 to 6.16; moderate certainty), MIS-ALA (MD, 3.00; 95% CI, 0.43 to 5.59; moderate certainty), MIS-DLA (MD, 3.37; 95% CI, 1.05 to 5.68; moderate certainty), MIS-PA (MD, 4.46; 95% CI, 1.60 to 7.31; moderate certainty), PA (MD, 4.37; 95% CI, 1.87 to 6.88; high certainty), and SuperPath (MD, 5.00; 95% CI, 0.58 to 9.42; high certainty) were associated with greater improvement in hip score compared with DLA. DLA was associated with lower decrease in pain score than SuperPath (MD, 1.16; 95% CI, 0.13 to 2.20; high certainty) and MIS-DLA (MD, 0.90; 95% CI, 0.04 to 1.76; moderate certainty). PA was associated with shorter operation times compared with 2-incision (MD, −23.85 minutes; 95% CI, −36.60 to −11.10 minutes; high certainty), DAA (MD, −13.94 minutes; 95% CI, −18.79 to −9.08 minutes; moderate certainty), DLA (MD, −10.50 minutes; 95% CI, −16.07 to −4.94 minutes; high certainty), MIS-ALA (MD, −6.76 minutes; 95% CI, −12.86 to −0.65 minutes; moderate certainty), and SuperPath (MD, −13.91 minutes; 95% CI, −21.87 to −5.95 minutes; moderate certainty). The incidence of 6 types of complications did not differ significantly between the approaches.

**Conclusions and Relevance:**

In this study, moderate to high certainty evidence indicated that compared with PA, all surgical approaches except DLA were associated with similar improvements of hip score but longer operation time. DLA was associated with smaller improvement of hip score. The safety of the different approaches did not show significant differences. These findings will help health professionals and patients with better clinical decision-making and also provide references for policy makers.

## Key Points

**Question:**

Which total hip arthroplasty approach is associated with the best efficacy and acceptability?

**Findings:**

In this systematic review and meta-analysis of 63 randomized clinical trials of surgical approaches for total hip arthroplasty with 4859 participants, all surgical approaches except the direct lateral approach were associated with greater improvements of hip score when compared with the posterior approach. The safety of different approaches did not show significant differences.

**Meaning:**

These findings may support improved clinical decision-making among health care professionals and patients and also provide information for policy makers.

## Teaser

This systematic review and meta-analysis evaluates the efficacy and safety associated with different surgical approaches for total hip arthroplasty.

## Introduction

Total hip arthroplasty (THA), known as “the operation of the 21st century,”^1^ has shown great success in relieving joint pain and disability^2^ and has been a procedure of choice for the treatment of end-stage degenerative joint diseases and trauma.^3,4^ Kurtz et al^5^ noted a 50% increase in the prevalence of THA in the United States from 1990 to 2002 and projected that the prevalence of total hip replacement would increase from 208 600 in 2005 to 572 000 in 2030.^6^ Despite its high success rate, surgeons continue to seek new treatment variations and perioperative options for THA to further improve functional outcomes and shorten the length of hospital stay as well as to reduce intrasurgical tissue damage.

The choice of operative approach can affect the efficacy and safety of THA.^7^ A variety of surgical approaches can be used, including the 2-incision approach,^8^ direct anterior approach (DAA),^9^ direct lateral approach (DLA),^9^ minimally invasive direct lateral approach (MIS-DLA),^10^ minimally invasive anterolateral approach (MIS-ALA),^11^ posterior approach (PA),^12^ minimally invasive posterior approach (MIS-PA),^13^ and supercapsular percutaneously assisted total hip arthroplasty (SuperPath).^14^ Among them, DLA and PA are considered traditional approaches, while the other 6 are minimally invasive approaches. The optimal surgical approach for THA remains inconclusive. According to the National Institute for Health and Care Excellence 2020 guideline, current evidence does not suggest that any of the THA approaches are more beneficial than others, and the choice of approach is mainly based on the knowledge and experience of the surgeon and individual patient characteristics.^15^ Each approach has a long learning curve, so a surgeon’s choice to change their preferred approach needs to be guided by clear justifications.^16,17,18,19^

Most existing research compares only 2 approaches, and there is a lack of evidence for head-to-head comparisons among all existing approaches for THA. Limited reviews have used rigorous meta-analytical techniques to obtain quantitative estimates of the outcomes for different approaches.^20,21,22^ The vague definitions of approaches in some studies can lead to classification errors and hence inaccurate comparisons. In addition, existing meta-analyses have not assessed the certainty or quality of evidence.^23^ Addressing these gaps, we performed a network meta-analysis of randomized clinical trials (RCTs) to compare existing THA approaches of efficacy and safety through comprehensive evidence synthesis of both direct and indirect comparisons. Patients who underwent primary THA for any indication were included in this analysis, and the outcome time point was set as the follow-up end point.

## Methods

A multidisciplinary panel consisting of orthopedic surgeons, rehabilitation physicians, an epidemiologist, a systems evaluation expert, and a statistician provided input into the study protocol. We registered our protocol on the PROSPERO (CRD42020221715) and reported our study following the Preferred Reporting Items for Systematic Reviews and Meta-analyses (PRISMA) and PRISMA-2020 guidelines and the extension statement for network meta-analysis (PRISMA-NMA).^24,25^

### Literature Review

EMBASE, Medline, and the Cochrane Library (from inception to February 23, 2020) were searched to identify studies on surgical approaches in THA. We updated our search on March 26, 2022, to include recent eligible trials. In addition, ClinicalTrials.gov was searched to identify additional studies and unpublished data. The detailed search strategy is shown in eAppendix 1 in Supplement 1. All relevant meta-analyses and systematic reviews retrieved during our searches were assessed to identify potentially eligible studies. Articles were exported to Endnote X9 and duplicates were removed, after which teams of paired reviewers (D.L. and another researcher) independently screened the titles and abstracts of studies to identify those eligible for inclusion. The full text of potentially eligible studies was evaluated according to the inclusion and exclusion criteria. A third reviewer (L.Y.) was consulted to resolve any disagreements.

### Selection Criteria

The inclusion criteria were (1) studies that included patients undergoing primary THA surgery for any indication; (2) studies that compared at least 2 surgical approaches for THA, without restricting the control group setting; (3) studies presenting any relevant outcome measures (eTable 1 in Supplement 1), with no limitation on follow-up time points; and (4) articles written in English. Studies that had abstract only or unavailable full text were excluded.

### Data Extraction

Using standardized, pilot-tested forms, each eligible trial underwent duplicate data abstraction by a pair of reviewers (D.L. and another researcher) working independently. Reviewers addressed discrepancies through adjudication by a third reviewer (L.Y.). If a study reported outcomes at several time points, the longest follow-up was used for analysis.

We collected information regarding patient characteristics (including age, sex, body mass index, country, and follow-up time), surgery details (such as indications, expertise of surgeon, anesthetic regimes, incision length, implants used, and rehabilitation protocols), and all reported outcome measures (such as hip score change, pain score change, hospitalization time, operation time, blood loss, quality of life [QOL] score change, cup abduction angle, and cup anteversion angle). The definitions of these 8 outcomes and 24 other outcomes are shown in eTable 1 in Supplement 1. If multiple instruments were used to measure the same outcome domain (such as hip score and pain score), we collected data from the most commonly reported instrument across trials included in our review. In addition, we used pain at rest rather than on movement if both were reported. For adverse events reported in the included trials, we selected 6 as being the most important to patients: dislocation, fracture, infection, nerve injury, reoperation, and thromboembolism (eTable 1 in Supplement 1).

### Statistical Analysis

#### Data Analysis and Synthesis

We converted hip score and pain score to a common scale on a domain-by-domain basis for better clinical interpretability^26^: (1) hip score to Harris hip score (0-100), where higher scores represent better outcomes, and (2) pain score to the 100-mm visual analogue scale, where higher scores represent worse outcomes. Since the different scales of QOL used across included studies could not be converted to a single scale, the Hedges method was used to calculate the standardized mean difference (SMD) for the QOL. The mean difference (MD) was calculated for all other indicators except QOL.

We used change scores from baseline rather than end-of-study scores to account for interpatient variability. When authors reported data as measures before and after intervention, we used methods outlined in the *Cochrane Handbook* to calculate MD and SD for change.^27^ When SDs were missing, we estimated them from SEs, *P* values, confidence intervals, or graphs. If none of these methods was feasible, we derived SDs from other studies included in our network meta-analysis using a validated imputation technique (eAppendix 2A in Supplement 1).^28^

Network meta-analysis was performed using the frequentist model with a graph-theoretical method by R version 4.1.2 package netmeta (version 2.1-0) (R Project for Statistical Computing). We used the networkplot command of Stata version 16.0 (StataCorp) to draw the network plots.^29^ The estimator was based on weighted least-square regression with the Moore-Penrose pseudoinverse method.^30^ We conducted pairwise meta-analysis with DerSimonian-Laird random-effects model to estimate the variance in heterogeneity between studies and to obtain direct evidence.^31^ League tables of the relative treatment effect sizes were used to visualize comparisons of network estimations. Global and local statistical heterogeneity was assessed with generalized Cochran *Q*.^32^ All comparisons were 2-tailed using a threshold of *P* ≤ .05.

We compared distributions of characteristics across study groups, organized by approaches, to assess the transitivity assumption of indirect comparisons. Local inconsistency of direct and indirect results was assessed with the node-splitting method for all comparison loops, and indirect results were derived from direct and network results by the back-calculation method.^33,34^ The detailed methods of multiple sensitivity analyses are presented in eAppendix 2B in Supplement 1, and the detailed methods of publication bias assessments are presented in eAppendix 2C in Supplement 1. We performed a network metaregression assuming a common coefficient across comparisons to explore the associations of covariates of interest with each outcome.^35^ The change from protocol is displayed in eAppendix 2D in Supplement 1.

#### Certainty of Evidence

We rated the certainty of evidence for each network estimate using the Grading of Recommendations, Assessment, Development, and Evaluation (GRADE) framework, which classifies evidence as high, moderate, low, or very low certainty. The starting point for certainty in direct estimates for RCTs is high, but it could be rated down based on limitations due to risk of bias, imprecision, inconsistency (heterogeneity), indirectness, and publication bias.^23^ The Cochrane Collaboration Risk of Bias 1 (ROB-1) tool^36^ was used independently by 2 reviewers (D.L. and another researcher) to evaluate the risk of bias of included studies (eTable 2 in Supplement 1). Additional details of the GRADE assessment are presented in eAppendix 2E in Supplement 1.

## Results

**Figure 1.**
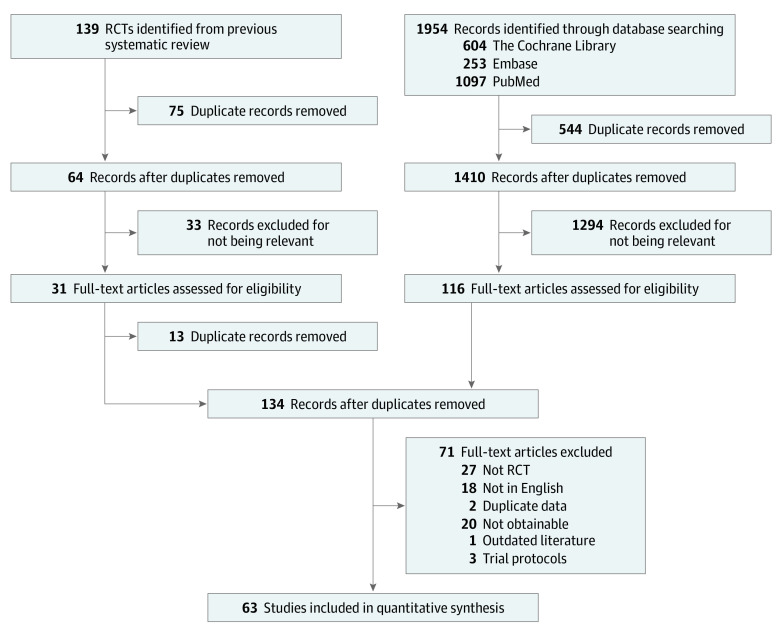
Study Flow Diagram of Study Selection Process RCT indicates randomized clinical trials.

We identified 2130 potential studies from database searches, of which 63 studies^8,9,11,12,13,37,38,39,40,41,42,43,44,45,46,47,48,49,50,51,52,53,54,55,56,57,58,59,60,61,62,63,64,65,66,67,68,69,70,71,72,73,74,75,76,77,78,79,80,81,82,83,84,85,86,87,88,89,90,91,92^ were eligible for inclusion (Figure 1). These studies were published between 2005 and 2021 (eAppendix 3 in Supplement 1).

### Characteristics of Included Studies

The included studies were RCTs that involved a total of 4859 patients, with a median (IQR) age of 64.0 (60.3-66.5) years, median (IQR) body mass index (calculated as weight in kilograms divided by height in meters squared) of 27.00 (25.58-28.27), median (IQR) percentage male of 46.74% (38.64%-54.74%) and median (IQR) follow-up time of 1.0 (0.5-2.0) years (eTable 3A in Supplement 1). The detailed characteristics of included studies are shown in eTables 3B, C, D, and E in Supplement 1. Regarding the learning curve, 31 studies^9,11,37,38,39,40,43,46,47,49,51,52,56,61,64,65,66,67,68,69,71,72,75,81,82,83,86,89,91,92,93^ (49.2%) showed relevant information, with surgeons in 7 studies^39,51,52,61,65,75,92^ (11.1%) in the learning phase and surgeons in the remaining 28 studies^9,11,37,38,40,43,46,47,49,56,64,66,67,68,69,71,72,81,82,83,86,89,91,93^ (38.1%) experienced (eTable 3C in Supplement 1).

**Figure 2.**
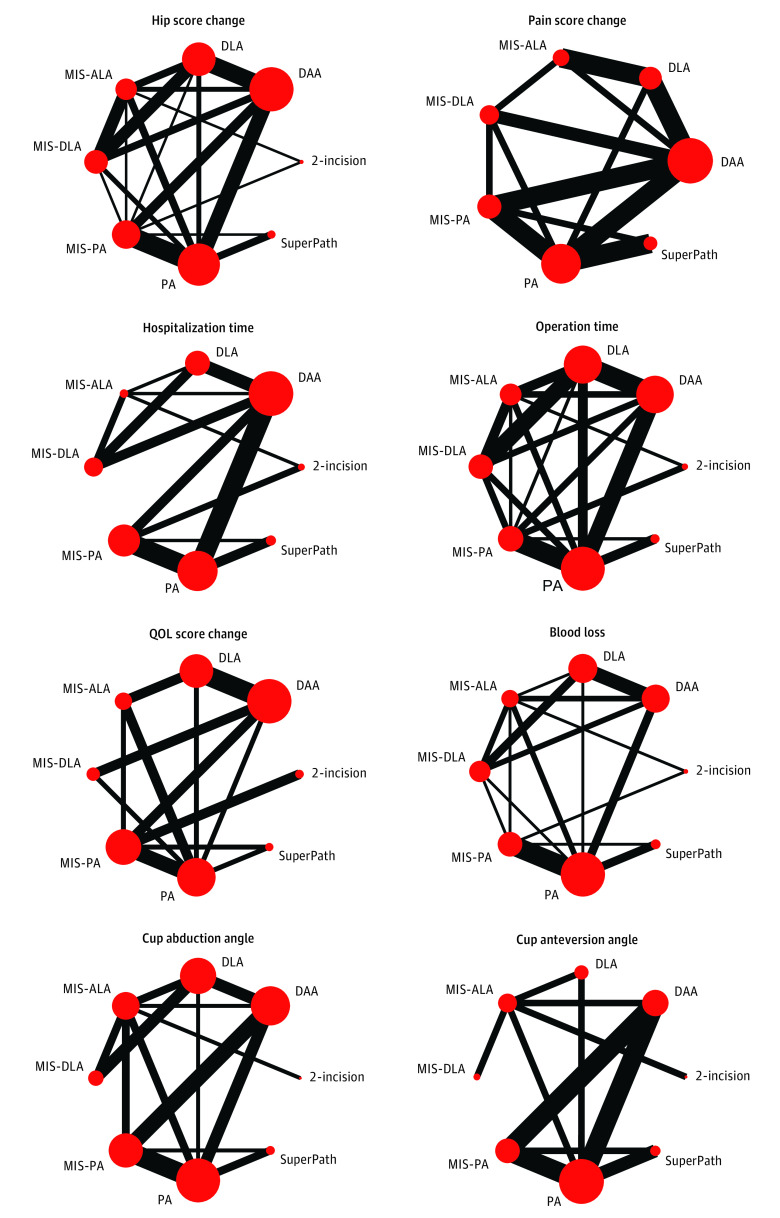
Network Plots Comparing Approaches in Primary Total Hip Arthroplasty for 8 Outcome Measures The line width is proportional to the number of studies comparing each pair of treatments, and the size of each node is proportional to the number of participants (sample size). DAA indicates direct anterior approach; DLA, direct lateral approach; MIS-ALA, minimally invasive anterolateral approach; MIS-DLA, minimally invasive direct lateral approach; MIS-PA, minimally invasive posterior approach; PA, posterior approach; QOL, quality of life; SuperPath, supercapsular percutaneously assisted total hip arthroplasty.

The categories and descriptions of these 8 surgical approaches for THA are displayed in eAppendix 4 in Supplement 1, and the schematic showing the entrance location of the 8 approaches is shown in eFigure 1 in Supplement 1. To eliminate inconsistencies caused by different naming methods among included studies, we redefined the naming of 8 approaches with specific text descriptions (eTable 4 in Supplement 1). The network plot for the 8 outcome measures is shown in Figure 2. The network plots for all other outcomes are shown in eFigure 2 in Supplement 1. The evaluation results of ROB-1 are shown in eFigure 3 and eTable 5 in Supplement 1.

### Outcomes

**Figure 3.**
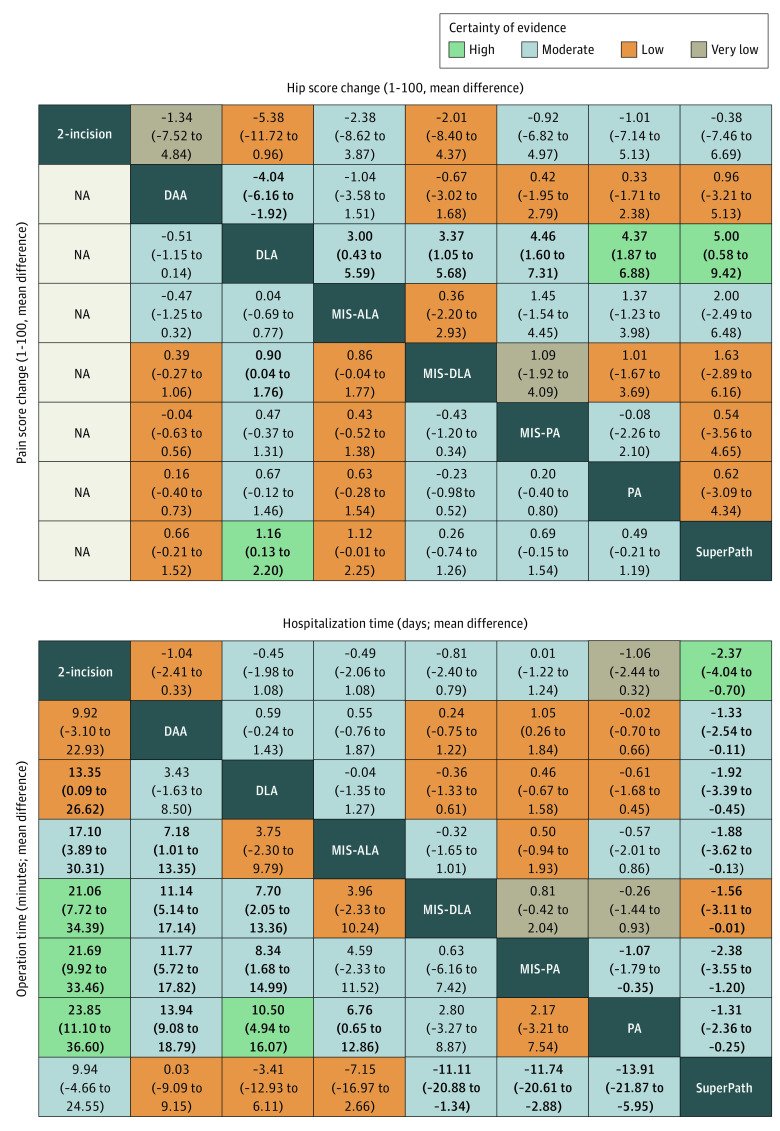
League Tables of Hip Score Change, Pain Score Change, Hospitalization Time, and Operation Time The relative effect sizes are measured as a mean difference along with 95% CIs. Bold indicates statistical significance. The color of each cell indicates the certainty of evidence according to Grading of Recommendations, Assessment, Development, and Evaluation. The treatments are listed in alphabetical order. Comparisons between treatments should be read from left to right and the estimate is in the cell in common between the column-defining treatment and the row-defining treatment. For pain score change, hospitalization time, and operation time, a mean difference lower than 0 favors the column-defining treatment. For hip score change, a mean difference lower than 0 favors the row-defining treatment. In the left lower half, a mean difference lower than 0 favors the column-defining treatment and in the upper right half, a mean difference lower than 0 favors the row-defining treatment. DAA indicates direct anterior approach; DLA, direct lateral approach; MIS-ALA, minimally invasive anterolateral approach; MIS-DLA, minimally invasive direct lateral approach; MIS-PA, minimally invasive posterior approach; NA, not applicable; PA, posterior approach; SuperPath, supercapsular percutaneously assisted total hip arthroplasty.

**Figure 4.**
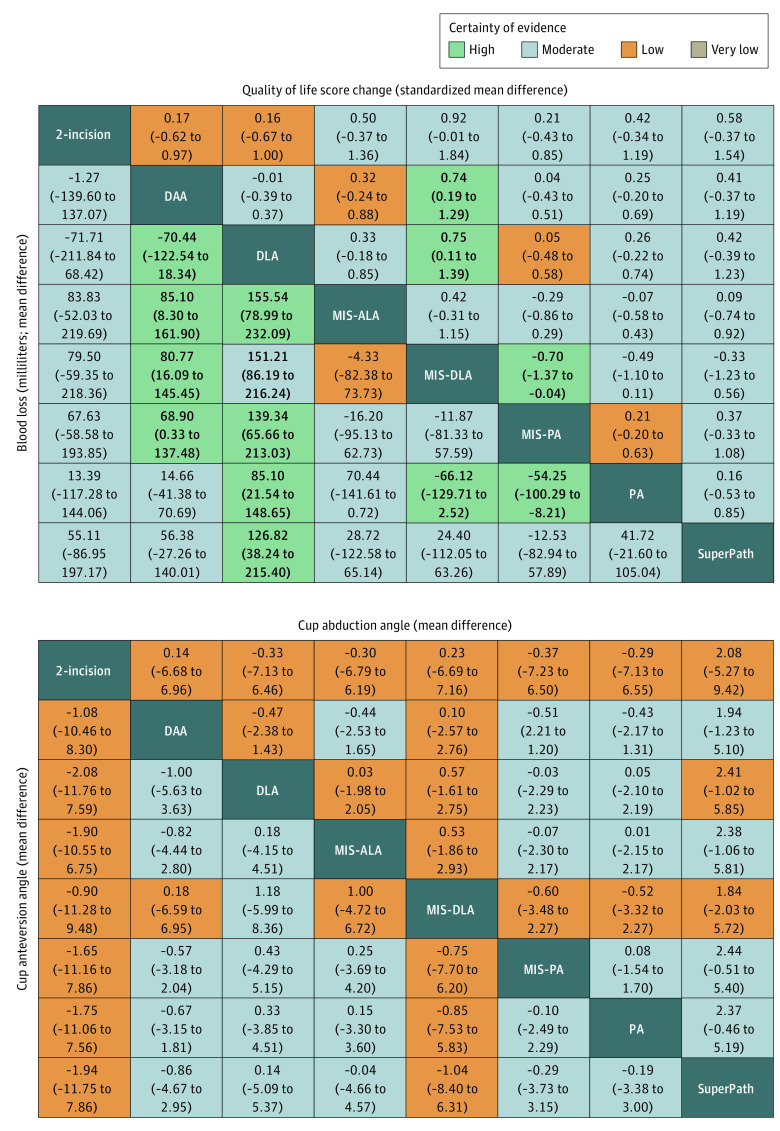
League Tables of Quality of Life Score Change, Blood Loss, Cup Abduction Angle, and Cup Anteversion Angle The league tables show the relative effect sizes of each approach, measured as a standardized mean difference for quality of life score change and mean difference for all other outcomes, along with 95% CIs. Bold indicates statistical significance. The color of each cell indicates the certainty of evidence according to Grading of Recommendations, Assessment, Development, and Evaluation. Treatments are listed in alphabetical order. Comparisons between treatments should be read from left to right, and the estimate is in the cell in common between the column-defining treatment and the row-defining treatment. For the quality of life score change, blood loss, and cup abduction angle, a mean difference lower than 0 favors the column-defining treatment. For cup anteversion angle, a mean difference lower than 0 favors the row-defining treatment. DAA indicates direct anterior approach; DLA, direct lateral approach; MIS-ALA, minimally invasive anterolateral approach; MIS-DLA, minimally invasive direct lateral approach; MIS-PA, minimally invasive posterior approach; PA, posterior approach; SuperPath, supercapsular percutaneously assisted total hip arthroplasty.

The GRADE assessment results showed that imprecision was the most frequent reason for downgrading certainty of evidence (eTable 6 and eFigure 4 in Supplement 1). League tables for outcome measures appear in Figure 3, Figure 4, and eTable 7 in Supplement 1. A corresponding metaregression analysis was also conducted (eTable 8 in Supplement 1). Heterogeneity (eTable 9 in Supplement 1), intransitivity (eFigure 5 in Supplement 1), and inconsistency (eFigure 6 in Supplement 1) of the network meta-analysis were evaluated. Most outcomes showed no significant publication bias (eFigure 7 in Supplement 1), and the sensitivity analyses all proved consistent with the primary results (eTable 10 in Supplement 1). The heat map illustrates the incidence rate of the 6 complication types for the 8 approaches (eFigure 8 in Supplement 1).

### Hip Score Change

**Figure 5.**
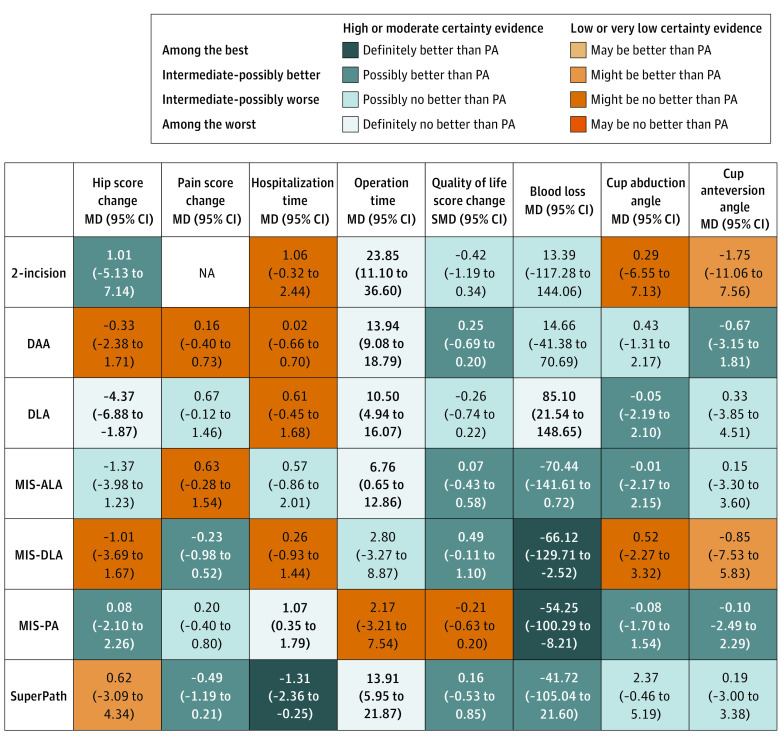
Summary of Relative Effect Sizes for Outcomes of Total Hip Arthroplasty Approaches on 8 Outcomes The certainty of evidence was rated by Grading of Recommendations, Assessment, Development, and Evaluation criteria, including imprecision. Imprecision was rated down only when the 95% CI crossed null effect. Approaches were categorized and certainty of evidence was rated in 1 of 2 ways: whether the intervention was clearly better or worse than the posterior approach (PA; the mean effect size exceeding or less than the null effect and the 95% CI not crossing the null effect threshold) or possibly better or worse than PA (the point estimate greater or less than the null effect and the 95% CI crossing the threshold). Bold text represents statistical significance. DAA indicates direct anterior approach; DLA, direct lateral approach; MIS-ALA, minimally invasive anterolateral approach; MIS-DLA, minimally invasive direct lateral approach; MIS-PA, minimally invasive posterior approach; MD, mean difference; NA, not available; SMD, standardized mean difference; SuperPath, supercapsular percutaneously assisted total hip arthroplasty.

A total of 50 studies^9,12,37,38,39,41,42,43,44,45,46,47,48,49,50,51,52,53,56,57,59,60,61,62,63,64,66,67,68,69,71,73,75,76,77,78,79,80,81,82,83,84,85,86,89,90,91,92^ (79%) with 3882 participants (80%) reported on hip score change from baseline to end point (Figure 3 and Figure 5). Compared with DLA, DAA (MD, 4.04; 95% CI, 1.92-6.16; moderate certainty), MIS-ALA (MD, 3.00; 95% CI, 0.43-5.59; moderate certainty), MIS-DLA (MD, 3.37; 95% CI, 1.05-5.68; moderate certainty), MIS-PA (MD, 4.46; 95% CI, 1.60-7.31; moderate certainty), PA (MD, 4.37; 95% CI, 1.87-6.88; high certainty), and SuperPath (MD, 5.00; 95% CI, 0.58-9.42; high certainty) showed significant improvement in hip score. However, no statistical differences were found between other approaches. Analysis of short- and long-term follow-up results of hip score showed that SuperPath (MD, 6.72; 95% CI, 1.16-12.28) and DAA (MD, 4.81; 95% CI, 0.42-9.19) were associated with better short-term results than PA, while there were no statistical differences in the long-term result (eTable 7 in Supplement 1).

### Pain Score Change

A total of 26 studies^9,11,12,13,43,49,53,55,57,59,60,61,63,66,67,75,76,77,83,84,85,86,90,91,92,93^ (41%) with 1936 participants (40%) reported on pain score change from baseline to endpoint (Figure 3 and Figure 5). DLA was associated with a lower decrease in pain score than SuperPath (MD, 1.16; 95% CI, 0.13-2.20; high certainty) and MIS-DLA (MD, 0.90; 95% CI, 0.04-1.76; moderate certainty).

### Hospitalization Time

A total of 33 studies^9,12,39,41,42,43,45,46,47,48,49,50,52,55,57,60,61,62,66,67,71,72,73,76,78,79,82,86,89,91,92,93^ (52%) with 2702 participants (56%) reported on hospitalization time (Figure 3 and Figure 5). MIS-PA was associated with longer hospitalization time than PA (MD, 1.07 days; 95% CI, 0.35-1.79 days; moderate certainty). SuperPath was associated with the shortest hospitalization time among all approaches. Metaregression analysis showed that increased incision length and more recent year of publication were associated with shorter hospitalization time (eTable 8 in Supplement 1).

### Operation Time

A total of 45 studies^9,12,13,38,39,41,42,43,44,47,48,49,50,52,53,54,55,56,57,58,59,60,61,63,64,66,67,68,70,72,73,76,77,78,79,80,81,82,83,88,89,90,91,93,94^ (71%) with 3437 participants (71%) reported on operation time (Figure 3 and Figure 5). PA was associated with showed shorter operation time compared with 2-incision (MD, −23.85 minutes; 95% CI, −36.60 to −11.10 minutes; high certainty), DAA (MD, −13.94 minutes; 95% CI, −18.79 to −9.08 minutes; moderate certainty), DLA (MD, −10.50 minutes; 95% CI, −16.07 to −4.94 minutes; high certainty), MIS-ALA (MD, −6.76 minutes; 95% CI, −12.86 to −0.65 minutes; moderate certainty), and SuperPath (MD, −13.91 minutes; 95% CI, −21.87 to −5.95 minutes; moderate certainty). Metaregression analysis showed that more recent year of publication was associated with shorter operation time (eTable 8 in Supplement 1).

### QOL Score Change

A total of 21 studies^9,11,12,37,38,47,48,51,57,61,71,72,75,76,77,84,85,86,90,92,93^ (33%) with 1904 participants (31%) reported on QOL score change from baseline to end point (Figure 4 and Figure 5). MIS-DLA was associated with a higher QOL score change than DAA (SMD, 0.74; 95% CI, 0.19-1.29; high certainty), DLA (SMD, 0.75; 95% CI, 0.11-1.39; high certainty), and MIS-PA (SMD, 0.70; 95% CI, 0.04-1.37; high certainty). Metaregression analysis showed that increased incision length was associated with lower QOL score (eTable 8 in Supplement 1).

### Blood Loss

A total of 33 studies^9,12,13,38,39,41,45,47,48,49,50,51,52,53,54,56,57,59,60,63,64,66,70,71,73,76,77,78,79,82,83,88,91^ (52%) with 2702 participants (56%) reported on blood loss (Figure 4 and Figure 5). DAA was associated with greater blood loss than MIS-ALA (MD, 85.10 mL; 95% CI, 8.30-161.90 mL; high certainty), MIS-DLA (MD, 80.77 mL; 95% CI, 16.09-145.45 mL; high certainty), and MIS-PA (MD, 68.90 mL; 95% CI, 0.33-137.48 mL; high certainty). DLA was associated with greater blood loss than DAA (MD, 70.44 mL; 95% CI, 18.34-122.54 mL; high certainty), MIS-ALA (MD, 155.54 mL; 95% CI, 78.99-232.09 mL; high certainty), MIS-DLA (MD, 151.21 mL; 95% CI, 86.19-212.24 mL; moderate certainty), MIS-PA (MD, 139.34 mL; 95% CI, 65.66-213.03 mL; high certainty), PA (MD, 85.10 mL; 95% CI, 21.54-148.65 mL; high certainty), and SuperPath (MD, 126.82 mL, 95% CI, 38.24-215.40 mL; high certainty). PA was associated with greater blood loss than MIS-DLA (MD, 66.12 mL; 95% CI, 2.52-129.71 mL; high certainty) and MIS-PA (MD, 54.25 mL; 95% CI, 8.21-100.29 mL; high certainty).

### Cup Abduction Angle and Cup Anteversion Angle

A total of 30 studies^12,38,42,45,48,49,51,52,53,54,55,56,57,58,59,61,62,64,66,67,70,75,79,82,83,85,86,89,91,93^ (48%) with 2364 participants (49%) reported on cup abduction angle, and 18 studies^12,38,49,52,53,54,55,56,57,59,66,67,85,86,91,93^ (26%) with 1392 participants (29%) reported on cup anteversion angle (Figure 4 and Figure 5). No significant differences were found among the 8 approaches for cup abduction angle or cup anteversion angle.

## Discussion

In this study, 63 RCTs including 4859 patients were analyzed to compare 8 commonly used approaches for primary THA. We found through moderate to high certainty evidence that PA was associated greater improvement in hip score than DLA. All sensitivity analyses proved consistent with the primary results. Regression analysis revealed a negative trend between publication year and hospitalization time. Metaregression analysis also showed that a longer incision length was associated with shorter hospitalization time and lower QOL score.

The high blood loss for DLA could be related to the amputation of the whole gluteus minimus and gluteus medius muscles, which could also be the reason for the poor hip score and pain score seen with this approach.^37^ The high incidence of nerve injury in DAA is mainly due to neurapraxia of the lateral thigh cutaneous nerve,^93,95,96^ which is probably still underestimated^97^ due to the wide anatomical variations in this nerve.^98^ Male gender and higher BMI are recognized factors for more challenging DAA cases.^99^ Although nerve injury is a common complication of DAA,^100,101^ one study^102^ showed it does not affect hip functionality. The longer operation time associated with DAA might be caused by its greater learning curve, or generally the increased complexity of this approach, as found in other studies.^94,103,104^ PA was associated with shorter operation time and more blood loss in our analysis. This may be related to the cutoff of obturator internus, piriformis, gemellus inferior, and gemellus superior muscles, providing the surgeon with a relatively large operating space.^38^

D’Arrigo et al^39^ found that whether the surgeon was on a learning curve for DAA, MIS-DLA, and MIS-ALA was associated with the operation time but not the efficacy and safety of THA. Moreover, Pagnano et al^105^ reported a 14% complication rate and 5% reoperation rate for the 2-incision approach that was not associated with the learning curve. Conversely, some researchers have pointed out increased complication rates of DAA during the learning curve phase.^17,106,107^ In this study, sensitivity analysis showed that whether the surgeon was in the learning phase or not did not have a significant association with the results. These conflicting findings suggest that there is controversy surrounding whether learning curves can affect surgical outcomes, and more high-quality studies and meta-analyses are needed to resolve this in the future.

We recommend that experts who wish to conduct RCT studies related to THA approaches focus more on minimally invasive approaches as well as compare 2 approaches that currently lack direct comparison, such as DAA vs SuperPath. The outcome measures should ideally include the 8 patient-important outcomes reported in our study. It is recommended that studies include patients with a single indication (such as osteoarthritis) and that all procedures are performed by the same experienced surgeon to improve the comparability of data. In addition, we recommend using the same incision appearance to achieve double-blinding. Finally, we advise larger sample sizes and longer follow-up periods to obtain more reliable long-term results.

### Limitations

We also note several limitations in our study. First, publication bias was detected in some of the outcomes, the consequences of which could be reduced by adequate retrieval. Second, the heterogeneity of different implants and surgeon expertise across the included studies was not accounted for in our analysis. Third, our analysis did not include the cost of each approach. Fourth, some of the approaches lacked evidence for direct comparisons, which may have affected our findings. The findings of our study should be interpreted with consideration given to these limitations.

## Conclusions

This systematic review and network meta-analysis provides important information for the choice of surgical approach for primary THA. Moderate to high certainty evidence indicated that compared with PA, all surgical approaches except DLA were associated with similar improvements in hip score but longer operation time. DLA was associated with lower improvement in hip score and higher blood loss. These findings will aid clinicians in balancing the risks and benefits of available approaches for primary THA and provide key evidence for producing recommendations for clinical practice.

## Notes

Supplement 1. **eTable 1.** Definition of Outcomes **eTable 2.** Classification of Individual Risk of Bias Items **eTable 3.** Characteristics of Included Studies **eTable 4.** Approach Name Redefinition of Articles Included **eTable 5.** Risk of Bias Results and Judgment Basis for Each Article **eTable 6.** Certainty of Evidence for Direct, Indirect, and Network Estimates **eTable 7.** League Table for Outcome Measures **eTable 8.** Results of Regression Analysis **eTable 9.** Heterogeneity Assessments **eTable 10.** Results of Sensitivity Analyses **eFigure 1.** Schematic Showing the Entrance Location of the 8 Surgical Approaches for THA **eFigure 2.** Network Plots for Other Outcome Measures **eFigure 3**. Risk of Bias Assessments **eFigure 4.** Contribution Matrices **eFigure 5.** Intransitivity Assessments **eFigure 6.** Inconsistency Assessments **eFigure 7.** Publication Bias: Funnel Plot **eFigure 8.** Incidence Rate (Sample Size) of 6 Complication Types **eAppendix 1.** Search Strategy **eAppendix 2.** Supplementary Methods **eAppendix 3.** Reference List of Eligible Studies **eAppendix 4.** Categories and Description of 8 Surgical Approaches in THA

Supplement 2.
Data Sharing Statement
