# Supplementary material for: Evaluation of Comparative Efficacy and Safety of Surgical Approaches for Total Hip Arthroplasty: A Systematic Review and Network Meta-analysis
Source: JAMA Netw Open. 2023 Jan 31;6(1):e2253942. doi: 10.1001/jamanetworkopen.2022.53942 (PMC9890287; doi:10.1001/jamanetworkopen.2022.53942)
Supplement: Supplement 1. — eTable 1. Definition of Outcomes eTable 2. Classification of Individual Risk of Bias Items eTable 3. Characteristics of Included Studies eTable 4. Approach Name Redefinition of Articles Included eTable 5. Risk of Bias Results and Judgment Basis for Each Article eTable 6. Certainty of Evidence for Direct, Indirect, and Network Estimates eTable 7. League Table for Outcome Measures eTable 8. Results of Regression Analysis eTable 9. Heterogeneity Assessments eTable 10. Results of Sensitivity Analyses eFigure 1. Schematic Showing the Entrance Location of the 8 Surgical Approaches for THA eFigure 2. Network Plots for Other Outcome Measures eFigure 3. Risk of Bias Assessments eFigure 4. Contribution Matrices eFigure 5. Intransitivity Assessments eFigure 6. Inconsistency Assessments eFigure 7. Publication Bias: Funnel Plot eFigure 8. Incidence Rate (Sample Size) of 6 Complication Types eAppendix 1. Search Strategy eAppendix 2. Supplementary Methods eAppendix 3. Reference List of Eligible Studies eAppendix 4. Categories and Description of 8 Surgical Approaches in THA [file jamanetwopen-e2253942-s001.pdf]

Supplemental Online Content

Yan L, Ge L, Dong S, et al. Evaluation of comparative efficacy and safety of surgical approaches for total hip arthroplasty: a systematic review and meta-analysis. *JAMA Netw Open.* 2023;6(1):e2253942. doi:10.1001/jamanetworkopen.2022.53942

eTable 1. Definition of Outcomes

eTable 2. Classification of Individual Risk of Bias Items

eTable 3. Characteristics of Included Studies

eTable 4. Approach Name Redefinition of Articles Included

eTable 5. Risk of Bias Results and Judgment Basis for Each Article

eTable 6. Certainty of Evidence for Direct, Indirect, and Network Estimates

eTable 7. League Table for Outcome Measures

eTable 8. Results of Regression Analysis

eTable 9. Heterogeneity Assessments

eTable 10. Results of Sensitivity Analyses

eFigure 1. Schematic Showing the Entrance Location of the 8 Surgical Approaches for THA

eFigure 2. Network Plots for Other Outcome Measures

eFigure 3. Risk of Bias Assessments

eFigure 4. Contribution Matrices

eFigure 5. Intransitivity Assessments

eFigure 6. Inconsistency Assessments

eFigure 7. Publication Bias: Funnel Plot

eFigure 8. Incidence Rate (Sample Size) of 6 Complication Types

eAppendix 1. Search Strategy

eAppendix 2. Supplementary Methods

eAppendix 3. Reference List of Eligible Studies

eAppendix 4. Categories and Description of 8 Surgical Approaches in THA

This supplemental material has been provided by the authors to give readers additional information about their work.

© 2023 Yan L et al. *JAMA Network Open.*

eTable 1. Definition of Outcomes

Outcomes	Definition/Description	Property
Hip score change	Follow-up-end hip score minus baseline hip score. We extracted scales that measured overall function of hip with the following hierarchy: HHS, WOMAC, OHS, UCLA activity score, LEFS, PMA, HOOS, LEM, JOA total, SANE, XSMFA function index. All scales were homogenized into HHS standard: larger numbers (0 to 100) represent better overall hip condition.	Continuous variable/ subjective outcome
Pain score change	Follow-up-end pain score minus baseline pain score. We extracted scales or subscales that measured pain after THA with the following hierarchy: VAS, WMOAS pain, HOOS pain, SF-36 pain, JOA pain. All scales were homogenized into VAS standard: smaller numbers (0 to 100) represent less severe hip pain conditions.	Continuous variable/ subjective outcome
Hospitalization time	Length of total hospitalization. The unit was standardized as "day".	Continuous variable/ objective outcome
Operation time	Duration of surgery. The unit was standardized as "min".	Continuous variable/ objective outcome
Blood loss	Total blood loss during surgery. The unit was standardized as "mL".	Continuous variable/ objective outcome
Quality of life score change	Follow-up-end pain score minus baseline pain score. We extracted scales or subscales that measured quality of life after THA with the following hierarchy: SF-36 physical health socre, SF-12 physical health score, EQ-5D, HOOS QOL, BI.	Continuous variable/ subjective outcome
Cup Abduction angle	Terms used were "cup abduction angle" or "cup inclination angle". The absolute value of the difference between the mean and 45 is used as the effect value.	Continuous variable/ objective outcome

© 2023 Yan L et al. *JAMA Network Open.*

Outcomes	Definition/Description	Property
Cup Anteversion angle	Term used was "cup anteversion angle". The absolute value of the difference between the mean and 15 is used as the effect value.	Continuous variable/ objective outcome
Short-term hip score	We extracted hip scores from 6±2 weeks of follow-up time.	Continuous variable/ objective outcome
Long-term hip score	We extracted the data closest to 1 year in the follow-up time of hip score of more than 1 year.	Continuous variable/ objective outcome
Dislocation	The number of dislocation was defined as the number of femoral head dislocation occurring after THA to the end point of follow-up.	Count variable/ objective outcome
Fracture	The number of fracture was defined as the number of fracture occurring after THA to the end point of follow-up.	Count variable/ objective outcome
Infection	The number of infection was defined as the number of infection occurring after THA to the end point of follow-up.	Count variable/ objective outcome
Nerve injury	The number of nerve injury was defined as the number of nerve injury occurring after THA to the end point of follow-up.	Count variable/ objective outcome
Reoperation	The number of reoperations was defined as the number of all reoperations after THA to the end of follow-up.	Count variable/ objective outcome
Thromboembolism	The number of thromboembolism was defined as the number of thromboembolism occurring after THA to the end point of follow-up.	Count variable/ objective outcome
AMSs change	Endpoint abductor muscle strengths values minus baseline bductor muscle strengths values.	Continuous variable/ objective outcome

© 2023 Yan L et al. *JAMA Network Open.*

Outcomes	Definition/Description	Property
Analgesic consumption	Quality of any analgesic (involving Morphine, Metamizol and Hydrocodone) comsumed by any form during hospitalization.<br>The total volume could be obtained by multiplying the daily consumption by the average number of hospital days. The unit was standardized as "mg".	Continuous variable/ objective outcome
Candence change	Endpoint candence values minus baseline candence values<br>The unit was standardized as "steps/min".	Continuous variable/ objective outcome
CK change	Endpoint CK values minus baseline CK values<br>The unit was standardized as "U/L".	Continuous variable/ objective outcome
CPR change	Endpoint CPR values minus baseline CPR values<br>The unit was standardized as "mg/L".	Continuous variable/ objective outcome
ESR change	Endpoint ESR values minus baseline ESR values<br>The unit was standardized as "mm/h".	Continuous variable/ objective outcome
Hb change	Endpoint Hb values minus baseline Hb values<br>The unit was standardized as "g/L".	Continuous variable/ objective outcome
HCT change	Endpoint HCT values minus baseline HCT values<br>The unit was standardized as "%".	Continuous variable/ objective outcome
IL-6 change	Endpoint IL-6 values minus baseline IL-6 values<br>The unit was standardized as "pg/mL".	Continuous variable/ objective outcome
Leg length discrepancy (LLD)	We extracted data of LLD after THA with the following hierarchy: LLD change, LLD endpoint.	Continuous variable/ objective outcome
Myoglobin change	Endpoint Myoglobin values minus baseline Myoglobin values	Continuous variable/

© 2023 Yan L et al. *JAMA Network Open.*

Outcomes	Definition/Description	Property
	The unit was standardized as "ug/L".	objective outcome
Stem alignment	Term used was "stem alignmen". The absolute value of the mean is used as the effect value.	Continuous variable/ objective outcome
Steps length change	Endpoint steps length values minus baseline steps length values<br>The unit was standardized as "meters".	Continuous variable/ objective outcome
Time up and go (TUG) test change	Endpoint Time up and go test values minus baseline Time up and go test values<br>The unit was standardized as "seconds".	Continuous variable/ objective outcome
Volume of blood transfusion	Term used was "blood" OR “transfusion” OR “red blood cell concentrate”.<br>The unit was standardized as "mL" and “U” was converted by multiplying by 200.	Continuous variable/ objective outcome
Walking speed change	Endpoint wakling speed values minus baseline walking speed values<br>The unit was standardized as "m/s".	Continuous variable/ objective outcome

PROM=Patient-reported outcome measures, HHS=Harris hip score, PMA=Postel Merle d'Aubigné, OHS=Oxford Hip Score, HOOS=Hip disablity and Osteoarthritis Outcome Score, WOMAC=Western Ontario and McMaster University Osteoarthritis Index, UCLA=University of California Los Angeles, LEFS=Lower Extremity Functional Scale, LEM=Lower Extremity Measure, JOA=Japanese Orthopaedic Association, XSMFA=The extra short musculoskeletal functional assessment questionnaire, SNAE=Single Assessment Numeric Evaluation, EQ-5D=European Quality of Life Health Questionnaire, SF-36=Short Form-36, SF-12=Short Form-12, QOL=Quality Of Life, BI=Barthel index, TUG=Timed Up and Go, CK=Creatine kinase, CPR=C-reactive protein, ESR=Erythrocyte sedimentation rate, Hb=Hemoglobin, HCT=Hematocrit, IL-6=Interleukin-6, AMSs=Abductor muscle strengths.

© 2023 Yan L et al. *JAMA Network Open.*

eTable 2. Classification of Individual Risk of Bias Items

Due to the specificity of surgery, it is not possible to be blinded for the operator, nor for the patient in the vast majority of cases due to the different incision locations. Therefore, we divided all outcome measures into two categories for ROB evaluation. The table below shows the criteria used to judge the subjective outcome measures, including hip score change, pain score change and QOL score change. For the rest of objective outcome measures, detection bias was not detected.

Risk of bias item	Low risk	Unclear risk	High risk
Randomisation	The investigators describe a random component in the sequence generation process such as:<br>·random blocks<br>·envelope<br>·throwing a dice<br>·computer random number generator<br>·random number table	·Insufficient information about the sequence generation process to permit judgement of 'Low risk' or 'High risk' (usually studies called 'randomised' without any further description)	·Sequence generated by odd or even date of birth;<br>·Sequence generated by some rule based on date (or day) of admission;<br>·Sequence generated by some rule based on hospital or clinic record number.
Allocation concealment	·Sequentially numbered, opaque, sealed envelopes.<br>·state blind<br>·Central allocation (including telephone, web-based and pharmacy-controlled randomization);	·Insufficient information to permit judgement of 'Low risk' or 'High risk'. This is usually the case if the study is just described as 'randomised' without any further description.	Participants or investigators enrolling participants could possibly foresee assignments and thus introduce selection bias, such as allocation based on:<br>·Using an open random allocation schedule (e.g. a list of random numbers)

© 2023 Yan L et al. *JAMA Network Open.*

Risk of bias item	Low risk	Unclear risk	High risk
Blinding of participants and personnel	· Blinding of participants	· Insufficient information to permit judgement of 'Low risk' or 'High risk';<br>· The study did not address this outcome;<br>· The preoperative blinding was achieved, but not postoperative.<br>· No blinding	· In this study, we do not think that blinding can have serious effects because there is no placebo.
Blinding of outcome assessment	· Independent observers who had not participated in the surgery	· Insufficient information to permit judgement of 'Low risk' or 'High risk';<br>· The study did not address this outcome.	· The assessor was not blinded to surgical approach.
Incomplete outcome data	· Lost of follow-up ＜10%<br>· Lost of follow-up >10%, similar number of missing in both groups, and dropouts with reasons reported.<br>· No missing outcome data;<br>· "reasonable" intention to treat analysis (e.g. at least one dose and one post baseline assessment)	· Lost of follow-up >10%, similar number of missing in both groups, but no reason given for missing visits.<br>· Insufficient reporting of attrition/exclusions to permit judgement of 'Low risk' or 'High risk'.	· Lost of follow-up >10%, and there is indeed a significant difference in the number of people between the two groups.
Selective reporting	· The study protocol is available and all of the study's pre-specified outcomes that are of interest in the review have been reported in the prespecified way; as we usually do not have the protocol, we will compare what is described in the method section with what is reported in the	· only abstract or poster<br>· Insufficient information to permit judgement of 'Low risk' or 'High risk'.	· Not all of the study's pre-specified primary outcomes have been reported.

© 2023 Yan L et al. *JAMA Network Open.*

Risk of bias item	Low risk	Unclear risk	High risk
	results.		
Other bias	· The study appears to be free of other sources of bias.	· Received funding from commercial companies	· Statistically significant baseline imbalance in an important Outcome<br>· Study has been claimed to be fraudulent

© 2023 Yan L et al. *JAMA Network Open.*

eTable 3. Characteristics of Included Studies

eTable 3A. Summary of characteristics of included studies

Study characteristics	No. (%)/ Median	IQR	Range
Eligible studies:			
Total No of trials	63		
No of participants	4859		
Median follow-up (year)	1	0.5 to 2	0.125 to 10
Median Publication year	2014	2010 to 2018	2005 to 2021
Region:			
Europe	29(14.29%)		
North America	14(22.22%)		
Asia	14(22.22%)		
Oceania	6(9.52%)		
Participants:			
Mean age (years)	64	60.3 to 66.5	44 to 84
Percentage male	46.74	38.64 to 54.74	12.9 to 100
Baseline mean BMI (kg/m2)	27	25.58 to 28.27	22.42 to 34.35

© 2023 Yan L et al. *JAMA Network Open.*

eTable 3B. Patient characteristics of included studies

Study	Interventions	Sample size	Sex (Male/ Female)	Mean age (years)	BMI (kg/m2)	Country	Follow-up
Abdel2017	2-incision	35	19/16	66(40-85)	30(21-45)	USA	8.5 y
	MIS-PA	36	20/16				
Barrett2019	DAA	41	NR	NR	NR	USA	5 y
	PA	42	NR	NR	NR		
Bon2019	DAA	50	21/29	67.26±10 (64.42-70.1)	26.46±3.58 (25.44-27.47)	France	3 mo
	MIS-PA	50	23/27	68.98±7.93 (66.73-71.23)	26.69±3.12 (25.8-27.58)		
Brismar2018	DAA	50	18/32	66(58-74)	27(24-29)	Sweden	5 y
	DLA	50	17/33	67(60-76)	27(24-30)		
Cao2020	DAA	65	27/38	61.4±12.8	24.7±1.9	China	6 mo
	PA	65	28/37	62.4±8.3	25.1±1.8		

© 2023 Yan L et al. *JAMA Network Open.*

Study	Interventions	Sample size	Sex (Male/ Female)	Mean age (years)	BMI (kg/m2)	Country	Follow-up
Catma2017	MIS-ALA	34	7/61	51.1±9.4(26-69)	NR	Japan	6 mo
	PA	34			NR		
Cheng2017	DAA	35	15/20	59(54-69)	27.7(25.8-30.0)	Australia	1 y
	PA	38	18/20	62.5(55-69)	28.3(24.8-31.1)		
Chimento2005	MIS-PA	28	16/12	67.2(47-83)	25.2(17.7-29.3)	USA	1-2year
	PA	32	13/19	65.6(47-85)	24.8(20-29)		
Christensen2015	DAA	28	13/15	64.3±9.1	31.1±5.1	USA	6 w
	PA	23	11/12	65.2±9.1	30.4±3.6		
D'Arrigo2009	DAA	20	12/8	64±8.0(47-72)	22.7±1.5(21.7-26.5)	Italy	6 w
	MIS-DLA	20	14/6	66.3±10.4(38-74)	27.6±3.0(20-30)		
	MIS-ALA	20	11/9	66±7.5(46-71)	23.1±1.5(22-27)		

© 2023 Yan L et al. *JAMA Network Open.*

Study	Interventions	Sample size	Sex (Male/ Female)	Mean age (years)	BMI (kg/m2)	Country	Follow-up
De2016	DAA	49	26/24	64.8±10.1	26.6±3.9	Spain	1 y
	DLA	50	26/23	63.5±12.5	26.9±3.1		
Della2010	MIS-PA	35	24/11	63.8±8.2	27.3±3.5	USA	1 y
	2-incision	37	25/12	61.2±8.0	27.6±3.3		
Dienstknecht2014	DAA	55	22/33	61.9±12.1(33-85)	27.6±6.0(15.7-42.0)	Germany	3 mo
	DLA	88	47/41	61.3±11.6(35-89)	30.1±5.6(17.6-48.8)		
Dorr2007	PA	30	16/14	63.9±13.6(34-87)	30.2±6.5(22.6-49.4)	USA	1 y
	MIS-PA	30	17/13	70.3±9.7(44-84)	27.6±4.5(18.9-37.8)		
Dutka2007	DLA	60	22/98	44(32-61)	27(22-29)	Poland	10.5mo
	MIS-DLA	60		46(40-67)	28(24-29)		8.5mo
Goosen2011	MIS-ALA	30	15/15	60±7.4	26.7±3.1	Netherlands	1 y

© 2023 Yan L et al. *JAMA Network Open.*

Study	Interventions	Sample size	Sex (Male/ Female)	Mean age (years)	BMI (kg/m2)	Country	Follow-up
	PA	30	13/17	62±6.3	26.8±2.7		
	MIS-PA	30	15/15	60±6.3	26.4±2.6		
Hu2012	2-incision	10	NR	51.6±14.5(29-79)	24.3±5(17.8-38)	China	2 y
	MIS-ALA	10	NR	52.1±14.9(29-82)	24.4±5(17-37.2)		
Inaba2011	MIS-ALA	50	12/38	63.5±10.7	22.9±4.0	Japan	1 y
	MIS-DLA	52	13/39	64.5±10.9	24.4±4.6		
Ji2012	PA	99	54:45	51±14.5	24.3±3.3	South Korea	37.5±10.0 mo
	DLA	97	58/39	52±15.1	24.3±3.0		38.3±9.2 mo
Khan2012	PA	52	19/33	72.8±1.1(59-90)	28.9±0.6	Australia	2 y
	MIS-PA	48	24/24	72.3±1.0(61-91)	28.5±0.7		
Kim2006	MIS-PA	35	53/17	55.6(43-68)	25.6(18.7~35.6)	Korea	26.4 mo

© 2023 Yan L et al. *JAMA Network Open.*

Study	Interventions	Sample size	Sex (Male/ Female)	Mean age (years)	BMI (kg/m2)	Country	Follow-up
	PA	35					
Korykin2021	MIS-PA	24	11/13	56.96±13.2(32-78)	29.04±4.91(20.2-39.9)	Russia	6 w
	SuperPath	20	10/10	56.75±12.86(20-70)	28.2±4.51(22.5-39.4)		
Laffosse2008	MIS-ALA	33	20/13	56.8(32-83)	25.9(18.9-34.9)	French	NR
	MIS-PA	43	28/15	55.7(23-77)	25.2(20.7-32.5)		
Landgraeber2013	DLA	40	14/26	71.03±5.38	26.76±3.83	Italy	3.5 y
	MIS-ALA	36	12/24	70.26±4.05	27.03±2.82		
Li2021	SuperPath	49	27/22	75.53±7.34	22.99±2.87	China	1 y
	PA	47	24/23	77.21±7.84	22.70±3.00		
Martin2011	DLA	41	14/27	63.1±10.2	29.4±5.5	Belgium	1 y
	MIS-ALA	42	12/30	66.7±10.1	30.6±6.1		

© 2023 Yan L et al. *JAMA Network Open.*

Study	Interventions	Sample size	Sex (Male/ Female)	Mean age (years)	BMI (kg/m2)	Country	Follow-up
Matziolis2011	MIS-DLA	20	6/14	62.4(30-87)	26.8(19-35)	Germany	1 y
	MIS-ALA	20	7/13	63.9(47-76)	27(20-39)		
Mayr2009	DAA	16	6/11	65(55–84)	27(20.8–36.1)	Austria	3mo
	DLA	17	8/9	69(59–78)	29(20.2–34.7)		
Mazoochain2009	DLA	26	11/14	NR	26.4±3.7	Germany	3mo
	MIS-DLA	26	9/17	NR	26.6±4.5		
Meneghini2008	2-incision	8	NR	54(38-74)	26(21-30)	USA	1 y
	MIS-PA	8	NR	54(38-74)	26(21-30)		
	MIS-DLA	7	NR	54(38-74)	26(21-30)		
Meng2021	SuperPath	20	8/12	64.55±9.06	23.36±2.55	China	1 y
	PA	20	9/11	65.25±10.33	22.82±2.61		

© 2023 Yan L et al. *JAMA Network Open.*

Study	Interventions	Sample size	Sex (Male/ Female)	Mean age (years)	BMI (kg/m2)	Country	Follow-up
Mjaaland2019	DAA	84	30/70	67±69	28±64	Norway	2 y
	DLA	80	38/62	66±69	28±64		
Moerenhout2020	DAA	28	11/17	70.4±9.1	27.6±4.4	Switzerland	5 y
	MIS-PA	27	18/9	68.9±8.8	26.5±4.3		
Muller2011	MIS-DLA	20	8/12	64(35-80)	26(24-34)	Germany	1 y
	MIS-ALA	24	12/12	66(52-79)	28(23-37)		
Muller2012	MIS-DLA	15	5/10	66.2±8	27.0±3.1	Germany	3 mo
	MIS-ALA	15	6/9	64.3±7	26.9±3.3		
Nistor2017	DAA	35	9/26	67(53.5-72.5)	27.45±3.76	Romania	3 mo
	DLA	35	16/19	64(54.5-67.5)	28.63±3.12		
Ogonda2005	MIS-PA	109	49/60	67.42±9.84	28.22±4.33	Britain	6 w

© 2023 Yan L et al. *JAMA Network Open.*

Study	Interventions	Sample size	Sex (Male/ Female)	Mean age (years)	BMI (kg/m2)	Country	Follow-up
	PA	110	58/52	65.85±10.33	28.94±4.33		
Pagnano2009	MIS-PA	36	20/16	66±12	30.2±5.6	USA	1 y
	2-incision	36	20/16	67±10.6	28.7±4.4		
Parvizi2016	DAA	44	18/26	NR	NR	USA	2 y
	DLA	40	14/26	NR	NR		
Pospischill2010	DLA	20	12/8	60.6	25.7	Austria	3mo
	MIS-ALA	20	8/12	61.9	25.7		
Reichert2018	DAA	77	45/32	63.2±8.2(44-77)	28.1±3.7(20.0-34.8)	Germany	1 y
	DLA	71	39/32	61.9±7.8(50-78)	28.3±3.4(20.9-42.2)		
Restrepo2010	DAA	63	NR	62.02(35.0-84.5)	25.18(18.8-29.9)	USA	2 y
	MIS-DLA	59	NR	59.91(40.1-76.1)	25.17(19.2-29.1)		

© 2023 Yan L et al. *JAMA Network Open.*

Study	Interventions	Sample size	Sex (Male/ Female)	Mean age (years)	BMI (kg/m2)	Country	Follow-up
Rosenlund2017	MIS-DLA	38	26/12	60±7	27±3	Denmark	1 y
	PA	39	26/13	62±6	28±4		
Roy2010	PA	31	4/27	84.0±8.1	NR	Canada	2 y
	MIS-PA	25	7/18	79.5±8.2	NR		
Rykov2021	DAA	23	8/15	62±9	27.8±7.3	Netherlands	1 y
	PA	23	11/12	63±15	28.6±8.4		
Schwarze2018	DLA	30	26/34	59(36-72)	26.7(20.6-37.2)	Germany	2 y
	MIS-ALA	30					
Sershon2017	MIS-PA	31	10/21	73.4±8.6	28.2±4	Chicago	8.2(5-10) y
	2-incision	32	9/23	70.9±7.3	28.7±2.9		
Shitama2009	DLA	8	4/24	53.4±13.3	23.0±3.7	Japan	6 mo

© 2023 Yan L et al. *JAMA Network Open.*

Study	Interventions	Sample size	Sex (Male/ Female)	Mean age (years)	BMI (kg/m2)	Country	Follow-up
	PA	20		61.3±10.7			
	MIS-DLA	15	5/29	61.7±11.5	23.2±3.6		
	MIS-PA	19		58.3±3.0			
Speranza2007	DLA	52	23/21	66.2(81-47)	29	Italy	6 mo
	MIS-DLA	45	20/26	65(81-38);	28		
Takada2018	DAA	30	26/4	62.6±10.4	24.4±4.4	Japan	1 y
	MIS-ALA	30					
Tan2018	MIS-PA	48	24/24	73±7.2	NR	Australia	10 y
	PA	52	19/33	73±7.8	NR		
Taunton2014	DAA	27	12/15	62.05	27.7	USA	1 y
	MIS-PA	27	13/14	66.4	29.2		

© 2023 Yan L et al. *JAMA Network Open.*

Study	Interventions	Sample size	Sex (Male/ Female)	Mean age (years)	BMI (kg/m2)	Country	Follow-up
Taunton2018	DAA	52	25/27	65±10(38-84)	29±22(19-39)	USA	1 y
	MIS-PA	49	24/25	64±11(37-85)	30±4(22-39)		
Thaler2018	DAA	16	NR	65(55~84)	27(20.8-36.1)	Australia	2y
	DLA	17	NR	69(59~78)	29(20.2-34.7)		
Ulivi2021	MIS-PA	22	7/15	74±8.9	23±2.8	Italy	6 mo
	PA	23	10/13	72±7.7	24±2.0		
Varela2013	DLA	25	12/13	63.8(9.65)	27.78(3.24)	Spain	5y
	MIS-DLA	25	12/13	64.8(10.45)	28.27(3.67)		
Vasilakis2012	DLA	18	13/5	65.36±11.3	26.5±3.65	Switzerland	4y
	MIS-ALA	19	15/4	66.76±10.07	27.3±3.43		
Wang2019	MIS-PA	26	15/11	55.85±17.62	23.70±4.55	China	1 y

© 2023 Yan L et al. *JAMA Network Open.*

Study	Interventions	Sample size	Sex (Male/ Female)	Mean age (years)	BMI (kg/m2)	Country	Follow-up
	MIS-DLA	28	17/11	54.96±12.95	23.52±3.28		
Witzleb2009	PA	30	NR	55(47-64)	28.9(20-38)	Germany	3 mo
	DLA	30	NR	58(46-64)	26.6(21-39)		
Xie2017	PA	46	19/27	64.47±12.09	24.06±2.72	China	1 y
	SuperPath	46	12/34	66.60±11.88	23.62±1.63		
Yang2009	PA	55	30/25	55.82±13.91	22.42±3.95	China	3 y
	MIS-ALA	55	26/29	59.47±13.24	23.12±3.23		
Zhao2017	DAA	64	24/36	64.88±12.13	34.35±3.1	China	6 mo
	PA	64	26/34	62.18±14.72	25.58±2.83		
Zomar2018	DAA	36	21/15	60.78±9.26	28.38±4.51	Britain	3 mo
	MIS-DLA	42	20/22	59.54±8.40	30.89±5.43		

© 2023 Yan L et al. *JAMA Network Open.*

NR=Not Reported. y=year. mo=month. w=week. d=day. DAA=direct anterior approach. DLA=direct lateral approach. MIS-DLA=minimally invasive direct lateral approach. MIS-ALA=minimally invasive anterolateral approach. PA=posterior approach. MIS-PA=minimally invasive posterior approach. SuperPath=supercapsular percutaneously assisted total hip arthroplasty.

© 2023 Yan L et al. *JAMA Network Open.*

eTable 3C. Surgery-related information for the included studies

Study	Interventions	Sample size	Indications	Expertise of the surgeon	Anaesthetic regimes	Incision length	Bilateral or Unilateral surgery	Number of surgeon
Abdel2017	2-incision	35	OA	Experienced	Spinal	NR	NR	1
	MIS-PA	36				NR		
Barrett2019	DAA	41	NR	NR	NR	NR	NR	1
	PA	42				NR		
Bon2019	DAA	50	OA	NR	NR	NR	Unilateral	1
	MIS-PA	50				NR		
Brismar2018	DAA	50	OA	Experienced	47 Spinal, 3 General	8-10	Unilateral	2
	DLA	50			45 Spinal, 5 General	10-20		
Cao2020	DAA	65	OA, AVN, DDH(Crowe I-II)	Experienced	NR	9.1±0.6	NR	NR
	PA	65				13.5±0.9		

© 2023 Yan L et al. *JAMA Network Open.*

Study	Interventions	Sample size	Indications	Expertise of the surgeon	Anaesthetic regimes	Incision length	Bilateral or Unilateral surgery	Number of surgeon
Catma2017	MIS-ALA	34	DDH(Crowe IV)	NR	General	NR	Bilateral and Unilateral	NR
	PA	34				NR		
Cheng2017	DAA	35	OA	Experienced	10 Spinal, 25 General	10.7(8.8-12)	Unilateral	2
	PA	38			17 Spinal, 21 General	13.5(12.7-15.5)		
Chimento2005	MIS-PA	28	NR	NR	NR	8cm	Unilateral	1
	PA	32				15cm		
Christensen2015	DAA	28	NR	Experienced	General	NR	NR	1
	PA	23				NR		
D'Arrigo2009	DAA	20	OA	Trained but not experienced	Spinal	NR	NR	1
	MIS-DLA	20				NR		
	MIS-ALA	20				NR		

© 2023 Yan L et al. *JAMA Network Open.*

Study	Interventions	Sample size	Indications	Expertise of the surgeon	Anaesthetic regimes	Incision length	Bilateral or Unilateral surgery	Number of surgeon
De2016	DAA	49	OA	NR	Spinal	11.5±0.7	NR	1
	DLA	50				10.4±0.9		
Della2010	MIS-PA	35	OA	Experienced	Spinal	7-10	Unilateral	1
	2-incision	37				NR		
Dienstknecht2014	DAA	55	OA	NR	Spinal	9.3±1.4	Unilateral	1
	DLA	88				13.4±2.7		
Dorr2007	PA	30	OA, DDH, AVN	Experienced	Spinal	19.78±1.2	Unilateral	2
	MIS-PA	30				9.8±1.0		
Dutka2007	DLA	60	OA,DDH, AVN	NR	NR	20-25	NR	NR
	MIS-DLA	60				6-8		
Goosen2011	MIS-ALA	30			NR	8.2±1.6	Unilateral	2

© 2023 Yan L et al. *JAMA Network Open.*

Study	Interventions	Sample size	Indications	Expertise of the surgeon	Anaesthetic regimes	Incision length	Bilateral or Unilateral surgery	Number of surgeon
	PA	30	OA, AVN, DDH, Trauma	Trained but not experienced		18		
	MIS-PA	30				8.6±2.3		
Hu2012	2-incision	10	Bilateral hip disease	Trained but not experienced	General	9.8±1.4(8-12)	Bilateral	1
	MIS-ALA	10				9.1±1.3(6.5-12)		
Inaba2011	MIS-ALA	50	DDH, OA, AVN, Pigmented villonodular synovitis	NR	NR	8.6±1.3	Unilateral	2
	MIS-DLA	52				7.7±1.2		
Ji2012	PA	99	AVN, OA, RA, AS, Fracture, Sequelae of pyogenic infection	NR	NR	16-22	Unilateral	1
	DLA	97				16-22		
Khan2012	PA	52	OA, RA, AVN	NR	Spinal	19.3±0.37	NR	2
	MIS-PA	48				12.6±0.72		

© 2023 Yan L et al. *JAMA Network Open.*

Study	Interventions	Sample size	Indications	Expertise of the surgeon	Anaesthetic regimes	Incision length	Bilateral or Unilateral surgery	Number of surgeon
Kim2006	MIS-PA	35	OA, AVN, AS	Experienced	Spinal	8	Bilateral	NR
	PA	35				15-20		
Korykin2021	MIS-PA	24	Unilateral hip disease	NR	NR	7-11	Unilateral	1
	SuperPath	20				7-11		
Laffosse2008	MIS-ALA	33	OA, AVN, RA	NR	NR	8	Unilateral	1
	MIS-PA	43				8		
Landgraeber2013	DLA	40	OA	NR	General	10.29±0.86	Unilateral	3
	MIS-ALA	36				11.72±1.69		
Li2021	SuperPath	49	AVN, Fracture	NR	Spinal	6.88±0.54	Unilateral	1
	PA	47				11.91±1.22		
Martin2011	DLA	41	OA, DDH, Coxa vara		General	14.8±3.3	Unilateral	1

© 2023 Yan L et al. *JAMA Network Open.*

Study	Interventions	Sample size	Indications	Expertise of the surgeon	Anaesthetic regimes	Incision length	Bilateral or Unilateral surgery	Number of surgeon
	MIS-ALA	42		Trained but not experienced		9.5±1.4		
Matziolis2011	MIS-DLA	20	Arthrosis	NR	NR	NR	Unilateral	3
	MIS-ALA	20				NR		
Mayr2009	DAA	16	NR	NR	NR	NR	Unilateral	1
	DLA	17				NR		
Mazoochain2009	DLA	26	OA, AVN	Experienced	NR	14.0(11-18)	Unilateral	2
	MIS-DLA	26				8.9(5-11)		
Meneghini2008	2-incision	8	OA	Trained but not experienced	NR	NR	Unilateral	1
	MIS-PA	8				NR		
	MIS-DLA	7				NR		
Meng2021	SuperPath	20	OA	Experienced	NR	7.83±1.12	Unilateral	1

© 2023 Yan L et al. *JAMA Network Open.*

Study	Interventions	Sample size	Indications	Expertise of the surgeon	Anaesthetic regimes	Incision length	Bilateral or Unilateral surgery	Number of surgeon
	PA	20				12.45±1.71		
Mjaaland2019	DAA	84	OA	Experienced	NR	8	Unilateral	5
	DLA	80				14		
Moerenhout2020	DAA	28	OA, AVN	Experienced	NR	NR	Unilateral	2
	MIS-PA	27				NR		
Muller2011	MIS-DLA	20	OA	Experienced	NR	10.4±2.0	Unilateral	2
	MIS-ALA	24				8.0±1.6		
Muller2012	MIS-DLA	15	OA	Experienced	NR	NR	NR	2
	MIS-ALA	15				NR		
Nistor2017	DAA	35	OA	NR	Spinal	12.18±1.91	Unilateral	1
	DLA	35				14.79±2.25		

© 2023 Yan L et al. *JAMA Network Open.*

Study	Interventions	Sample size	Indications	Expertise of the surgeon	Anaesthetic regimes	Incision length	Bilateral or Unilateral surgery	Number of surgeon
Ogonda2005	MIS-PA	109	OA, RA, AVN	Experienced	Spinal	9.50±0.95	Unilateral	1
	PA	110				15.81±0.93		
Pagnano2009	MIS-PA	36	OA	Experienced	Spinal	7-9.5	NR	1
	2-incision	36				6(3.8-5)		
Parvizi2016	DAA	44	OA	NR	Spinal	NR	NR	1
	DLA	40				NR		
Pospischill2010	DLA	20	OA	NR	NR	12	Unilateral	1
	MIS-ALA	20				8-10		
Reichert2018	DAA	77	OA	Trained but not experienced	NR	NR	Unilateral	8
	DLA	71				NR		
Restrepo2010	DAA	63	OA	NR	Spinal	9.45(8-11)	Unilateral	1

© 2023 Yan L et al. *JAMA Network Open.*

Study	Interventions	Sample size	Indications	Expertise of the surgeon	Anaesthetic regimes	Incision length	Bilateral or Unilateral surgery	Number of surgeon
	MIS-DLA	59				9.94(8-12)		
Rosenlund2017	MIS-DLA	38	OA	NR	NR	NR	Unilateral	2
	PA	39				NR		
Roy2010	PA	31	Fracture	NR	NR	16	NR	NR
	MIS-PA	25				8		
Rykov2021	DAA	23	OA	NR	NR	NR	Unilateral	3
	PA	23				NR		
Schwarze2018	DLA	30	OA	NR	NR	NR	NR	5
	MIS-ALA	30				NR		
Sershon2017	MIS-PA	31	OA	NR	Spinal	7-10	Unilateral	NR
	2-incision	32				NR		

© 2023 Yan L et al. *JAMA Network Open.*

Study	Interventions	Sample size	Indications	Expertise of the surgeon	Anaesthetic regimes	Incision length	Bilateral or Unilateral surgery	Number of surgeon
Shitama2009	DLA	8	AVN, DDH	Experienced	Spinal	13.1±1.1	Unilateral	1
	PA	20				14.7±2.8		
	MIS-DLA	15				9.0±0		
	MIS-PA	19				9.0±0		
Speranza2007	DLA	52	OA, AVN, Fracture	Experienced	Spinal	12.8±2.3	Unilateral	1
	MIS-DLA	45				7.1±1.1		
Takada2018	DAA	30	OA	Experienced	Spinal	10.5±1.3	Bilateral	1
	MIS-ALA	30				10.3±1.1		
Tan2018	MIS-PA	48	OA, RA, AVN	NR	NR	NR	NR	1
	PA	52				NR		
Taunton2014	DAA	27	OA	NR	NR	10	Unilateral	1

© 2023 Yan L et al. *JAMA Network Open.*

Study	Interventions	Sample size	Indications	Expertise of the surgeon	Anaesthetic regimes	Incision length	Bilateral or Unilateral surgery	Number of surgeon
	MIS-PA	27				10		
Taunton2018	DAA	52	OA	Experienced	Spinal	NR	Unilateral	4
	MIS-PA	49				NR		
Thaler2018	DAA	16	OA	NR	NR	NR	Unilateral	2
	DLA	17				NR		
Ulivi2021	MIS-PA	22	RA, OA	NR	NR	NR	NR	1
	PA	23				NR		
Varela2013	DLA	25	OA,AN	Experienced	NR	NR	NR	1
	MIS-DLA	25				NR		
Vasilakis2012	DLA	18	OA	Experienced	NR	14-16	Unilateral	1
	MIS-ALA	19				8-10		

© 2023 Yan L et al. *JAMA Network Open.*

Study	Interventions	Sample size	Indications	Expertise of the surgeon	Anaesthetic regimes	Incision length	Bilateral or Unilateral surgery	Number of surgeon
Wang2019	MIS-PA	26	OA	NR	NR	9.42±0.50	Unilateral	1
	MIS-DLA	28				7.46±0.51		
Witzleb2009	PA	30	OA	NR	NR	16(11-23)	Unilateral	2
	DLA	30				15(11-19)		
Xie2017	PA	46	OA	NR	NR	14.5±2.38	Unilateral	1
	SuperPath	46				7.4±1.06		
Yang2009	PA	55	OA, AVN, RA, Fracture	Experienced	Spinal	15.19±1.82	Unilateral	1
	MIS-ALA	55				7.49±0.86		
Zhao2017	DAA	64	OA, AVN, DDH (Crowe III-IV)	Experienced	NR	9.09±0.45	NR	1
	PA	64				13.14±0.31		
Zomar2018	DAA	36	OA		NR	NR	Unilateral	3

© 2023 Yan L et al. *JAMA Network Open.*

Study	Interventions	Sample size	Indications	Expertise of the surgeon	Anaesthetic regimes	Incision length	Bilateral or Unilateral surgery	Number of surgeon
	MIS-DLA	42		Trained but not experienced		NR		

NR=Not Reported. y=year. mo=month. w=week. d=day. OA=osteoarthritis. RA=rheumatoid arthritis. AVN=avascular necrosis. AS=ankylosing spondylitis. DAA=direct anterior approach. DLA=direct lateral approach. MIS-DLA=minimally invasive direct lateral approach. MIS-ALA=minimally invasive anterolateral approach. PA=posterior approach. MIS-PA=minimally invasive posterior approach. SuperPath=supercapsular percutaneously assisted total hip arthroplasty.

© 2023 Yan L et al. *JAMA Network Open.*

eTable 3D. Information about the implants used

Study	Interventions	Sample size	Fxiation of femoral component	Fxiation of acetabular component	Implants Used
Abdel2017	2-incision	35	NR	NR	Fully porousecoated femoral component (VerSys FullCoat; Zimmer; Warsaw, IN), acetabular component (Trilogy Modular Trabecular Metal; Zimmer) without additional acetabular screw fixation, and highly cross-linked polyethylene design (Longevity; Zimmer)
	MIS-PA	36			
Barrett2019	DAA	41	NR	NR	A Corail Total Hip System femoral stem, a Pinnacle Acetabular Cup System cup, an AltrX cross-linked polyethylene liner, and a cobalt-chromium-molybdenum femoral head, size 28, 32, or 36 mm (all DePuy Synthes, Warsaw, IN).
	PA	42			
Bon2019	DAA	50	cemented or cementless	cementless	NR
	MIS-PA	50			

© 2023 Yan L et al. *JAMA Network Open.*

Study	Interventions	Sample size	Fxiation of femoral component	Fxiation of acetabular component	Implants Used
Brismar2018	DAA	50	cementless	cementless	Accolade stem and Trident PSL cup, Stryker, Kalamazoo, MI, USA
	DLA	50			
Cao2020	DAA	65	cementless	cementless	Pinnacle + Corail, DePuy Synthes, USA
	PA	65			
Catma2017	MIS-ALA	34	cementless	cementless	Distal split and proximal HA-coated femoral stem (Secur-Fit; Stryker Orthopaedics, Mahwah, New Jersey, USA)
	PA	34			
Cheng2017	DAA	35	cementless	cementless	Implants utilized were the R3 acetabular system and Anthology femoral stem. Weight bearing surfaces used were either ceramic on ceramic Biolox Delta or Oxinium on polyethylene. (Smith & Nephew, Memphis, TN)
	PA	38			
Chimento2005	MIS-PA	28	cemented or cementless	cemented or cementless	A press-fit monoblock elliptical acetabular component
	PA	32			

© 2023 Yan L et al. *JAMA Network Open.*

Study	Interventions	Sample size	Fxiation of femoral component	Fxiation of acetabular component	Implants Used
Christensen2015	DAA	28	NR	NR	A short tapered wedge-shaped femoral component (Taperloc Microplasty, Biomet, Warsaw, IN)； a porous-coated hemispherical titanium acetabular component (Ringloc, Biomet)， Bearing couples consisted of secondgeneration highly cross-linked polyethylene (ArcomXL, Biomet) and either Biolox Delta ceramic or cobalt chrome femoral heads
	PA	23			
D'Arrigo2009	DAA	20	cementless	cementless	Hipstar femoral stem with trident acetabular component (Stryker Howmedica Osteonics)； Proxima femoral stem component with Pinnacle acetabular component (Depuy, Warsaw, IN)； ABG II femoral stem with Trident acetabular component (Stryker Howmedica Osteonics)
	MIS-DLA	20			
	MIS-ALA	20			
De2016	DAA	49	cemented or cementless	cementless	The Medacta hip system (Quadra stem, Versafit cup, Medacta international, Castel San Pietro,
	DLA	50			

© 2023 Yan L et al. *JAMA Network Open.*

Study	Interventions	Sample size	Fxiation of femoral component	Fxiation of acetabular component	Implants Used
					Switzerland) was used in both groups
Della2010	MIS-PA	35	cementless	cementless	Trilogy1 acetabular component and Longevity1 liner; Zimmer Inc, Warsaw, IN, USA； VerSys1 Epoch Full Coat; Zimmer
	2-incision	37			
Dienstknecht2014	DAA	55	cementless	cemented or cementless	Pressfit acetabular components and cement-free hydroxyapatite-coated stems with metal heads
	DLA	88			
Dorr2007	PA	30	NR	NR	A cementless Converge cup (Zimmer, Warsaw, Indiana)；The femoral component was a noncemented Anatomic Porous Replacement stem (Zimmer)
	MIS-PA	30			
Dutka2007	DLA	60	cemented or cementless	cemented or cementless	60 cementless and 60 cemented
	MIS-DLA	60			
Goosen2011	MIS-ALA	30	NR	NR	A Bi-Metric porous-coated uncemented stem and a metal-metal Magnum femoral head and
	PA	30			

© 2023 Yan L et al. *JAMA Network Open.*

Study	Interventions	Sample size	Fxiation of femoral component	Fxiation of acetabular component	Implants Used
	MIS-PA	30			acetabular shell (Biomet, Warsaw, IN)
Hu2012	2-incision	10	cementless	cementless	A Trilogy cup (Zimmer, Warsaw, Indiana, U.S.A.) and Fiber Metal Taper stem (Versys; Zimmer, Warsaw, Indiana)
	MIS-ALA	10			
Inaba2011	MIS-ALA	50	cementless	cementless	A cementless cup and stem were implanted；<br>a cobalt chrome-on-polyethylene bearing
	MIS-DLA	52			
Ji2012	PA	99	cementless	cementless	The acetabular components were of a hemispherical titanium cup (Plasmacup SC, Aesculap AG & Co, Tuttlingen, Germany) with an outer pure titanium plasma sprayed coating (Plasmapore, Aesculap AG & Co) and an alumina acetabular insert (BIOLOX forte, CeramTec AG, Plochingen, Germany). The femoral component was of a slightly tapered, rectangular, collarless titanium stem (BiCONTACT, Aesculap AG & Co). The proximal one third of the stem was coated with
	DLA	97			

© 2023 Yan L et al. *JAMA Network Open.*

Study	Interventions	Sample size	Fxiation of femoral component	Fxiation of acetabular component	Implants Used
					PLASMAPORE. A 28-mm alumina femoral head (BIOLOX forte, CeramTec AG) was used in all patients.
Khan2012	PA	52	cemented	cementless	The Spectron cemented femoral component and the Reflection uncemented acetabular component (both Smith & Nephew)
	MIS-PA	48			
Kim2006	MIS-PA	35	cementless	cementless	A cementless Duraloc Option acetabular component (DePuy, Leeds, UK)； A 28- mm (inner diameter) alumina ceramic liner (DePuy)
	PA	35			
Korykin2021	MIS-PA	24	cementless	cementless	The cementless acetabular component Dynasty® PC Shell and femoral component Profemur Z CLASSIC FEMORAL STEM with a cobalt chrome femoral head on Ultra high molecular weight Dynasty A-CLASS POLY LINER (MicroPort Orthopedics, Inc. Arlington, TN, USA)
	SuperPath	20			
Laffosse2008	MIS-ALA	33	cementless	cementless	

© 2023 Yan L et al. *JAMA Network Open.*

Study	Interventions	Sample size	Fxiation of femoral component	Fxiation of acetabular component	Implants Used
	MIS-PA	43			A cementless Schuster™ cup (Zimmer, Centerpulse)；a Durom™ (Zimmer, Centerpulse) cementless cup；a cementless hydroxyapatite coated Omnicase™ stem (Zimmer, Center_x005f_x0002_pulse)
Landgraeber2013	DLA	40	cemented	cemented	The Trident® cup (Stryker™, 325 Corporate Drive, Mahwah, New Jersey 07430, United States)； a Duraloc® cup (DePuy Orthopaedics Inc.™, 700 Orthopaedic Drive, Warsaw, IN 46582, United States)； A cemented Exeter® stem (Stryker™, 325 Corporate Drive, Mahwah, New Jersey 07430, United States)；the bearing surfaces were metal on highly cross-linked polyethylene
	MIS-ALA	36			
Li2021	SuperPath	49	cementless	cementless	The biomaterial prosthesis provided by Shanghai minimally invasive orthopedic medical technology Co., Ltd. was used.
	PA	47			

© 2023 Yan L et al. *JAMA Network Open.*

Study	Interventions	Sample size	Fxiation of femoral component	Fxiation of acetabular component	Implants Used
Martin2011	DLA	41	cemented	cemented or cementless	A cemented femoral stem (Versys; Zimmer Inc, Warsaw, IN) and either cemented or pressfit acetabular component (Allofit; Zimmer Inc)； Cemented femoral components were placed in all patients (Tha.lis; Orthogese, Brussels, Belgium), and cemented or press-fit acetabular components (Tha.hy. thi; Orthogese)
	MIS-ALA	42			
Matziolis2011	MIS-DLA	20	cementless	cementless	A cementless endoprosthesis with an Allofit cup and an Alloclassic stem (Zimmer, Warsaw, IN, USA)
	MIS-ALA	20			
Mayr2009	DAA	16	cementless	cementless	The Trident_x005f_x0003_ cup (Stryker Orthopaedics, Mahwah, NJ) and the Accolade TMZF stem (Stryker Orthopaedics, Mahwah, NJ)
	DLA	17			
Mazoochain2009	DLA	26	cementless	cementless	A standard cementless cup (SC-screwcup, Aesculap, Tuttlingen, Germany) and a cementless stem (CR-stem, Implantcast, Buxtehude, Germany)
	MIS-DLA	26			

© 2023 Yan L et al. *JAMA Network Open.*

Study	Interventions	Sample size	Fxiation of femoral component	Fxiation of acetabular component	Implants Used
Meneghini2008	2-incision	8	cementless	cementless	A press-fit acetabular component (Trilogy, Zimmer, Warsaw, Ind) and a cementless fully porous-coated femoral component (Beaded Fullcoat, Zimmer); acetabular liners were made of highly cross-linked polyethylene (Longevity, Zimmer, Warsaw, Ind)
	MIS-PA	8			
	MIS-DLA	7			
Meng2021	SuperPath	20	cementless	cementless	(SuperPath group: Microport Orthopaedics, Arlington, TN, USA; PLA group: DePuy Synthes, Warsaw, IN, USA)
	PA	20			
Mjaaland2019	DAA	84	cementless	cementless	A cemented cup (Marathon®; DePuy, Warsaw, IN, USA), uncemented stem (Corail®; DePuy), and a ceramic head with a diameter of 32 mm (Biolox®forte; Ceramtec, Plochingen, Germany)
	DLA	80			
Moerenhout2020	DAA	28	NR	NR	(Quadra-H stem and Versacup hip system, Medacta), with metal on polyethylene bearing
	MIS-PA	27			
Muller2011	MIS-DLA	20	cementless	cementless	A straight cementless titanium stem (Zweymu¨ller, Smith and

© 2023 Yan L et al. *JAMA Network Open.*

Study	Interventions	Sample size	Fxiation of femoral component	Fxiation of acetabular component	Implants Used
	MIS-ALA	24			Nephew_x005f_x0003_ , Rotkreuz, Switzerland) and an uncemented titanium press-fit cup (Allofit_x0003_ , Zimmer , Warsaw, Indiana, US)
Muller2012	MIS-DLA	15	cementless	cementless	Uncemented Press-Wt cups (AlloWt®, Zimmer®, Warsaw, Indiana, US) and uncemented straight stems type Zweymüller (Alloclassic®, Zimmer®, Warsaw, Indiana, US) were implanted
	MIS-ALA	15			
Nistor2017	DAA	35	cementless	cementless	A Metabloc™ uncemented femoral stem system, cobalt-chrome Versys® 32 mm diameter femoral head, polyethylene liner form Trilogy® acetabular system, and Trilogy® uncemented acetabular system shell, with acetabular self-tapping bone screws if needed (Zimmer Warsaw, IN 46580 U.S.A.)
	DLA	35			
Ogonda2005	MIS-PA	109	cemented	cementless	A cementless cup (Pinnacle; DePuy, Warsaw, Indiana) and a cemented stem (Xpress Rapid
	PA	110			

© 2023 Yan L et al. *JAMA Network Open.*

Study	Interventions	Sample size	Fxiation of femoral component	Fxiation of acetabular component	Implants Used
					Custom or C-Stem; DePuy, Leeds, United Kingdom)
Pagnano2009	MIS-PA	36	NR	NR	The same femoral component design (VerSys FullCoat; Zimmer, Warsaw, Indiana) and the same acetabular component design (Trilogy Modular Trabecular Metal; Zimmer)
	2-incision	36			
Parvizi2016	DAA	44	cementless	cementless	A proximally coated, collarless, tapered femoral stem (ML Taper, Zimmer, Warsaw, IN) and a porous tantalum acetabular component (Continuum, Zimmer, Warsaw, IN)； The type of bearing surface used was delta ceramic femoral head and highly cross-linked polyethylene (Longevity, Zimmer, Warsaw, IN)
	DLA	40			
Pospischill2010	DLA	20	cementless	cementless	The same standard type of implant was used, specifically, the cementless Alloclassic Variall system (Zimmer, Winterthur, Switzerland), a conical threaded cup in combination with a tapered straight stem. The articulating
	MIS-ALA	20			

© 2023 Yan L et al. *JAMA Network Open.*

Study	Interventions	Sample size	Fxiation of femoral component	Fxiation of acetabular component	Implants Used
					partners were ceramic-on-crosslinked polyethylene, metal-oncrosslinked polyethylene, or ceramic-on-ceramic
Reichert2018	DAA	77	cementless or cemented	cementless or cemented	Trilogy or Allofit cups (Trilogy® Acetabular Hip System; Allofit® Acetabular Cup System), the non-cemented M/ L-Taper stem or the cemented M. E. Müller straight stem (all Zimmer)
	DLA	71			
Restrepo2010	DAA	63	cementless	cementless	A proximally coated, collarless, tapered femoral stem (Accolade; Stryker Orthopaedics, Mahwah, NJ) and a plasma-sprayed acetabular component (Trident, Stryker Orthopaedics)
	MIS-DLA	59			
Rosenlund2017	MIS-DLA	38	cementless	cementless	Cementless components (Bi-metric stem and Exceed ABT Ringloc-x shell and metal head, size 32 mm or 36 mm)
	PA	39			
Roy2010	PA	31	cemented	cemented	Standard instrumentation (cemented Versys1 LD/FX femoral stem component with unipolar head, Zimmer, Warsaw, USA)
	MIS-PA	25			

© 2023 Yan L et al. *JAMA Network Open.*

Study	Interventions	Sample size	Fxiation of femoral component	Fxiation of acetabular component	Implants Used
Rykov2021	DAA	23	cementless	cemented	A cemented acetabular component (Stanmore, Biomet Corporation, the Netherlands) and an uncemented femoral component (Taperloc, Biomet Corporation, the Netherlands)
	PA	23			
Schwarze2018	DLA	30	cementless	cementless	A cementless short stem hip implant of appropriate size； The stem was made of titanium forged alloy (Ti4Al6V) with a coating of pure titanium, a 20- µm layer of calcium phosphate in the proximal part, and a polished tip； The acetabular component was the Plasmacup SC press-fit socket (Aesculap AG, Tuttlingen, Germany) with a polyethylene or ceramic insert
	MIS-ALA	30			
Sershon2017	MIS-PA	31	cementless	cementless	A cementless acetabular component with adjunctive screw fixation, a modular, highly cross-linked polyethylene liner, and a cementless, cylindrical, diaphyseal-engaging femoral component with a cobalt-chromium alloy femoral
	2-incision	32			

© 2023 Yan L et al. *JAMA Network Open.*

Study	Interventions	Sample size	Fxiation of femoral component	Fxiation of acetabular component	Implants Used
					head (Trilogy® acetabular component, Longevity® liner, and VerSys® Epoch Full Coat stem; Zimmer Inc, Warsaw, IN, USA)
Shitama2009	DLA	8	cementless	cementless	All patients for primary cementless total hip arthroplasty
	PA	20			
	MIS-DLA	15			
	MIS-PA	19			
Speranza2007	DLA	52	cementless	cementless	A cementless cup (Trident; Stryker Howmedica) and a cementless stem (Hypstar; Stryker Howmedica)
	MIS-DLA	45			
Takada2018	DAA	30	cementless	cementless	Cementless implan_x005f_x0002_tation with ceramic-on-highly cross-linked polyethylene bearings； Tapered femoral stems (TaperLoc Microplasty stem; Zimmer Biomet, Warsaw, IN) and hemispherical acetabular components (G7
	MIS-ALA	30			

© 2023 Yan L et al. *JAMA Network Open.*

Study	Interventions	Sample size	Fxiation of femoral component	Fxiation of acetabular component	Implants Used
					Acetabular System; Zimmer Biomet, Warsaw, IN)； Second-generation highly crosslinked polyethylene (E1; Zimmer Biomet) and delta ceramic head (32 or 36 mm)
Tan2018	MIS-PA	48	cemented	cementless	The Spectron cemented femoral component and a Reflection uncemented acetabular component (Smith & Nephew, Memphis, Tenn)
	PA	52			
Taunton2014	DAA	27	NR	NR	Femoral component design (Corail; DePuy, Warsaw, Indiana) and acetabular component design (Pinnacle; DePuy)
	MIS-PA	27			
Taunton2018	DAA	52	cementless	cementless	Hemispherical uncemented acetabular component (Pinnacle®; DePuy Orthopaedics Inc, Warsaw, IN, USA) and the same uncemented hydroxyapatitecoated femoral stem (Corail®; DePuy Orthopaedics Inc) with a Biolox® delta ceramic femoral head (CeramTec GmbH, Plochingen, Germany) and highly crosslinked
	MIS-PA	49			

© 2023 Yan L et al. *JAMA Network Open.*

Study	Interventions	Sample size	Fxiation of femoral component	Fxiation of acetabular component	Implants Used
					polyethylene acetabular bearing surfaces
Thaler2018	DAA	16	NR	NR	The Trident hemispherical cup (Stryker Orthopedics, Mahwah, NJ) and the Accolade TMZF stem (Stryker Orthopedics, Mahwah, NJ)
	DLA	17			
Ulivi2021	MIS-PA	22	metal	cementless	The Accolade II femoral stem (Stryker, Michigan, USA) and Trident cup with poly insert (Stryker, Michigan, USA)
	PA	23			
Varela2013	DLA	25	cementless	cementless	Press-Wt Bihapro acetabular component (Biomet® Bridgend, UK) and uncemented CeraWt stem (Ceraver® Gonesse, France)
	MIS-DLA	25			
Vasilakis2012	DLA	18	cementless	cementless	(cementless Zweymüller-Plus THA: a Bicon double-conus threaded cup with an SL-Plus tapered straight stem [Smith & Nephew Orthopaedics])；The articulating partners were ceramic-on-ceramic with a 28-mm ceramic ball head
	MIS-ALA	19			
Wang2019	MIS-PA	26	NR	NR	NR

© 2023 Yan L et al. *JAMA Network Open.*

Study	Interventions	Sample size	Fxiation of femoral component	Fxiation of acetabular component	Implants Used
	MIS-DLA	28			
Witzleb2009	PA	30	cementless	cementless	A cementless press-fit cup, cementless straight stem and a 28mm metal-onmetal (in cases of metal allergy ceramic-on-ceramic) articulation (Fitmore or Allofit cup, CLS stem, Metasul or Cerasul bearing, Zimmer Ltd., Warsaw, US)
	DLA	30			
Xie2017	PA	46	cementless	cementless	The same cementless THA implants (i.e., acetabular component, acetabular liner, femoral component, femoral head)
	SuperPath	46			
Yang2009	PA	55	cementless	cementless	An uncemented THA (Versys; Zimmer, Warsaw, Indiana)
	MIS-ALA	55			
Zhao2017	DAA	64	NR	NR	NR
	PA	64			
Zomar2018	DAA	36	NR	NR	NR

© 2023 Yan L et al. *JAMA Network Open.*

Study	Interventions	Sample size	Fxiation of femoral component	Fxiation of acetabular component	Implants Used
	MIS-DLA	42			

NR=Not Reported. DAA=direct anterior approach. DLA=direct lateral approach. MIS-DLA=minimally invasive direct lateral approach. MIS-ALA=minimally invasive anterolateral approach. PA=posterior approach. MIS-PA=minimally invasive posterior approach. SuperPath=supercapsular percutaneously assisted total hip arthroplasty.

© 2023 Yan L et al. *JAMA Network Open.*

eTable 3E. Rehab protocols of included studies.

Study	rehab protocols
Abdel2017	All patients were moved from bed to a chair on the day of surgery and began walking with weight-bearing as tolerated on the morning after surgery. Two sessions of supervised physical therapy were done on each day that the patient remained in the hospital. The patients were discharged from the hospital when they could move in and out of bed with minimal assistance, walk 100 ft (30.5 m) with a walker or crutches, walk up and down three stairs, and control their pain with oral medication. Traditional patient precautions to prevent total hip dislocations were not employed; the only warning was for the patients in the mini-posterior-incision group to avoid the combination offlexion of>100° combined with marked internal rotation of the hip. Otherwise, the patients were encouraged to proceed with activities as tolerated, allowing the hip symptoms to be their guide. Specifically, the patients were told that they did not need to feel as if the hip were fragile and that they were free to switch from the walker to a cane and then to get rid of the cane whenever they felt comfortable doing so.
Barrett2019	Not reported (NR)
Bon2019	Postoperatively, patients were raised during the first evening. Discharge was authorized, generally on postoperative day 2, only if walking and unassisted climbing up and down stairs was possible. Fifteen physiotherapy sessions were prescribed; rehabilitation in a residential center was authorized only for patients living alone.
Brismar2018	NR

© 2023 Yan L et al. *JAMA Network Open.*

Study	rehab protocols
Cao2020	All patients were encouraged to get out of bed on the day of surgery and start weight-bearing walking with the help of walking aids in the following days. Both groups had the same postoperative functional rehabilitation protocols. Patients of the PLA group were asked to avoid flexing their hip joints to more than 90° or adducting their hip joints beyond neutral. Patients in the DAA group had no range of motion restrictions. Patients with no serious complications or obvious anemia were discharged from hospital. In addition, patients were told that they could stop using the walking aids gradually after being discharged from the hospital and that activities which did not lead to discomfort were preferred. All patients were encouraged to get out of bed on the day of surgery and start weight-bearing walking with the help of walking aids in the following days. Patients of the PLA group were asked to avoid flexing their hip joints to more than 90° or adducting their hip joints beyond neutral. Patients in the DAA group had no range of motion restrictions. Patients with no serious complications or obvious anemia were discharged from hospital. In addition, patients were told that they could stop using the walking aids gradually after being discharged from the hospital and that activities which did not lead to discomfort were preferred.
Catma2017	NR
Cheng2017	All patients were mobilized the day after surgery. Routine hip precautions (avoidance of combined hip flexion >90° and internal rotation pas t the neutral plane) were instituted for the PA group. The DAA group did not have restrictions to hip movement. The target day of discharge for home or transfer to rehabilitation was the third post-operative day. This was assessed daily by physiotherapists and physicians supporting the orthopaedic team. Patients not meeting the discharge requirements were transferred to a rehabilitation facility
Chimento2005	NR
Christensen2015	All patients were weight-bearing as tolerated on the day of surgery regardless of approach. Patients undergoing PA THA were given standard postoperative precautions to prevent dislocation, whereas DAA THAs were not given any postoperative restrictions.
D'Arrigo2009	NR

© 2023 Yan L et al. *JAMA Network Open.*

Study	rehab protocols
De2016	All patients were allowed to stand on the second post-operative day, and were instructed to weight-bearing as tolerated with the use of a walker.
Della2010	All surgery on these 72 patients was performed as the first case of the day to facilitate discharge on the first postoperative day. If hemodynamically stable, patients were out of bed and ambulating with the assistance of a physical therapist on the day of surgery. Once discharged from the hospital, a physical therapist went to the patient's home three times per week for the first 3 weeks postoperatively, and patients were encouraged to transition to a cane and then no assist device when the patient and the therapist judged it was safe to do so. Patients then were encouraged to attend outpatient physical therapy for an additional 6 weeks for abductor strengthening and gait training. A nurse also visited the patient at home for the first 3 weeks postoperatively to monitor their wound and anticoagulation.
Dienstknecht2014	Mobilisation (partial weight bearing of a maximum of 30 kg for 6 weeks and then full weight bearing) was supervised by physiotherapists.
Dorr2007	NR
Dutka2007	NR
Goosen2011	NR
Hu2012	NR
Inaba2011	Physical therapy started on the first postoperative day, and all patients were allowed to commence walking exercises with full weight bearing.
Ji2012	Patients were instructed to walk with partial weight bearing with the aid of 2 crutches for 4 weeks after surgery.
Khan2012	No restrictions were imposed, and patients were mobilised weight-bearing as tolerated within 24 hours of surgery, and discharged when independently mobile.

© 2023 Yan L et al. *JAMA Network Open.*

Study	rehab protocols
Kim2006	The patients were allowed to stand on the day after surgery. The patients used crutches with weight bearing as tolerated for 4 weeks and then used a cane until they feel secure.
Korykin2021	All patients were weight-bearing as tolerated on the day of surgery regardless of approach. Early postoperative rehabilitation was the same for both groups and was performed by the same physiotherapy team at the same institution and started the first day after surgery. Upon discharge, patients were advised to resume activities as they could tolerate
Laffosse2008	NR
Landgraeber2013	Standardized physical therapy was commenced on the first postoperative day. Patients were mobilized with two crutches and full weight-bearing was allowed, depending on the individual level of pain.
Li2021	After surgery, the patient was asked to stay in supine position for 6 h, and the affected limb was raised with Brown's frame. The patient was asked to keep abduction neutral position to avoid hip dislocation caused by excessive flexion, adduction and internal rotation. After removing the drainage tube, patients were asked to perform muscle recovery training under the assistant of the nurses and physicians. The exercise time was no less than 2 h/ d during hospitalization
Martin2011	Weight-bearing status was protected with 2 crutches for a period of 3 weeks and then with one for an additional 3 weeks.
Matziolis2011	NR
Mayr2009	Patients were instructed to walk with two crutches during the first 6 weeks after surgery. For the following 6 weeks, patients were instructed to use one crutch on the contralateral side.
Mazoochain2009	If possible, patients were mobilized on the Wrst postoperative day with half-weight bearing until the healing of the wound was complete. During the Wrst few days they were mobilized with a trolley, afterwards with crutches. All of them were discharged between the 12th and 14th postoperative day for 3 weeks in a rehabilitation following inpatient treatment. The loading on the operated leg was increased by 20 kg per week
Meneghini2008	

© 2023 Yan L et al. *JAMA Network Open.*

Study	rehab protocols
	Once discharged, the patient continued a formal physical therapy program at home for up to 4 weeks. To minimize confounding variables, the therapist and patient were given specific instructions to perform gait training, muscle strengthening, and flexibility while maintaining hip range-of-motion precautions. Once deemed safe by the therapist, the patient was able to transition from the home to an outpatient physical therapy program with identical instructions and goals. The patient and therapist were encouraged to advance as quickly as possible. Patients were able to progress to a cane as tolerated and encouraged to use a cane until they could ambulate without a limp.
Meng2021	Immediate hip flexion, pneumatic compression with foot pumps, and deep breathing exercises were emphasized to minimize thromboembolic and pulmonary complications. After obtaining written approval from the physical therapist, patients began indoor walking independently with a tolerated weight bearing. Patients were educated in self-care and homebased rehabilitation before discharge. They were instructed to walk daily and to gradually increase their walking distance toward a goal of 2 km/day. All patients were discharged on postoperative day 3 and allowed to walk with a cane.
Mjaaland2019	NR
Moerenhout2020	NR
Muller2011	Physical therapy was initiated on the 1st postoperative day. The goals of therapy were to enable the patients to independently transfer, ambulate with full weight bearing using two crutches and negotiate stairs. All patients were transferred after successful completion of wound healing to a rehabilitation clinic for a 3 week standardized recovery program. This includes exercises for the entire lower extremity, the ankle, knee and all the muscles surrounding the hip. The goals for the patients were to regain full ROM/ flexibility, regain strength and endurance, and nearly all proprioception. Crutches had to be used for at least 6 weeks, depending on the preoperative muscular condition of each patient.
Muller2012	Postoperative mobilisation started on the Wrst day after surgery. Pain medication and physiotherapeutic treatment were equally applied to all patients. Forearm crutches had to be used during walking, with full weight bearing only after the 6th postoperative week

© 2023 Yan L et al. *JAMA Network Open.*

Study	rehab protocols
Nistor2017	NR
Ogonda2005	Unless they were not well medically, all patients were mobilized with full weight-bearing on the first postoperative day, as they had been instructed in the use of an appropriate walking aid preoperatively.
Pagnano2009	All patients were moved from bed to a chair on the day of surgery and began walking with weight-bearing as tolerated on the morning after surgery. Two sessions of supervised physical therapy were done on each day that the patient remained in the hospital. The patients were discharged from the hospital when they could move in and out of bed with minimal assistance, walk 100 ft (30.5 m) with a walker or crutches, walk up and down three stairs, and control their pain with oral medication. Traditional patient precautions to prevent total hip dislocations were not employed; the only warning was for the patients in the mini-posterior-incision group to avoid the combination of flexion of >100 degree combined with marked internal rotation of the hip. Otherwise, the patients were encouraged to proceed with activities as tolerated, allowing the hip symptoms to be their guide. Specifically, the patients were told that they did not need to feel as if the hip were fragile and that they were free to switch from the walker to a cane and then to get rid of the cane whenever they felt comfortable doing so. Return to driving was at the patient's discretion as long as the patient was not taking narcotic pain medication during the daytime. The safety of returning to driving at the patient's discretion has not been established
Parvizi2016	NR
Pospischill2010	Mobilization started on the first day after surgery with use of two forearm crutches with four-point walking. The use of two crutches was recommended for three weeks postoperatively. Patients were allowed to dispense with the crutches for full weight-bearing as soon as possible, depending on the individual level of mobilization and pain. Additional intensive physical therapy was started on the first day and was continued until the time of suture removal. All patients were discharged after a minimum hospital stay of ten days (range, ten to thirteen days)
Reichert2018	NR

© 2023 Yan L et al. *JAMA Network Open.*

Study	rehab protocols
Restrepo2010	The patients were seen by a physical therapist a few hours after arrival on the ward and helped to sit in a chair or ambulate with assistance if possible. Physical therapy occurred at least twice daily thereafter. Physical therapy protocol was identical for both groups; all patients in this study were allowed to progress with weight bearing as tolerated, also, the instructions to wean off support was left open to tolerance, and all muscular groups strengthening protocols were also identical among patients.
Rosenlund2017	The patients were mobilized with 2 canes and allowed full weight bearing immediately postoperatively, with no movement restrictions.
Roy2010	Rehabilitation protocol consisted of progressive weight-bearing as tolerated by the patient. In-hospital physical therapy was started on postoperative day one and continued until discharge.
Rykov2021	NR
Schwarze2018	NR
Sershon2017	NR
Shitama2009	The patients in both groups were allowed out of bed on the second postoperative day. Weight-bearing using a walker or crutches was begun as tolerated on the third postoperative day
Speranza2007	Physical therapy began on the first postoperative day. The major goals of therapy were to enable the patient to ambulate independently with a walker or with a cane. Patients were either discharged home or transferred to a rehabilitation facility according to their medical condition, progress in therapy, and home support system
Takada2018	The patients were mobilized on the first postoperative day with full weight bearing as tolerated. The patients were discharged when they were well enough to walk with aid.
Tan2018	NR

© 2023 Yan L et al. *JAMA Network Open.*

Study	rehab protocols
Taunton2014	All patients were encouraged to move from bed to a chair on the day of surgery and begin walking with weight-bearing as tolerated on the morning after surgery. Two sessions of supervised physical therapy were planned on each hospital day. The patients were discharged from the hospital when they could move in and out of bed with minimal assistance, walk 100 ft (30.5 m) with a walker or crutches, walk up and down three stairs, and control their pain with oral medication. Both groups had the same standardized muscle rehabilitation protocols. The posterior approach patients had range of motion arch restrictions for flexion limited to 90 degrees and no adduction beyond neutral. The anterior approach patients had no range of motion restrictions. Otherwise, the patients were encouraged to proceed with activities as tolerated, allowing the hip symptoms to be their guide.
Taunton2018	Structured physical therapy (PT) began the day after surgery and continued during the hospitalization. Patients were encouraged to sit up at the bedside the evening of their surgery. On postoperative Day 1, the patients began ambulation with the assistance of PT with a walker or crutches as well as active ROM. Weightbearing was progressed as tolerated. A home therapy program was given to the patient although formal PT did not continue on an outpatient basis. The patients were instructed to progress ambulation from a walker when they were able to walk stable without pain and then to continue with a crutch or cane until they were able to walk without a limp. The patients were encouraged to maximize independent ambulation and increase daily distance ambulated.
Thaler2018	Patients were instructed to walk with two crutches during the first 6 weeks after surgery. For the following 6 weeks, patients were instructed to use one crutch on the contralateral side. Physical therapy was not prescribed for any of the subjects.
Ulivi2021	NR
Varela2013	They were animated to walk since the day after surgery. No rapid recovery protocol was applied in any patient.
Vasilakis2012	Mobilization started on postoperative day 1 with the use of 2 forearm crutches with 4-point walking. The use of 2 crutches was recommended for 3 weeks postoperatively. Patients were allowed to discontinue the crutches for full weight bearing as soon as possible, depending on the individual level of mobilization and pain.
Wang2019	

© 2023 Yan L et al. *JAMA Network Open.*

Study	rehab protocols
	Passive and active leg-raising training from the first day, partial weight bearing walking from the third day, going up and down stairs from seventh to tenth day.
Witzleb2009	Walking training was started on the first postoperative day, with full weight-bearing allowed. All patients underwent a standardized physiotherapy program until hospital discharge at the seventh postoperative day. Following discharge, all patients trained walking under full weight-bearing with two crutches and received physiotherapy at an individual basis. During the first four weeks, hip flexion was limited to 90° and forced internal as well as external rotation was not allowed. Four weeks after surgery all patients were admitted to a cooperative rehabilitation department, where they underwent a standardized rehabilitation program for three weeks.
Xie2017	NR
Yang2009	NR
Zhao2017	The standard rehabilitation program consisting of weight bearing as tolerated with walking aid started the day after surgery. Patients were discharged when stable surgical wounds, hip flexion of 100°, hip abduction of 40°, and adequate mobility for daily activities were achieved.
Zomar2018	NR

© 2023 Yan L et al. *JAMA Network Open.*

eTable 4. Approach Name Redefinition of Articles Included

In order to eliminate the interference caused by different naming methods between different articles, we redefined the name of the approach with the specific text description of the article, as shown in the table below. If the apporach name we defined is different from the original text, it will be displayed in bold.

Study	Approach name in the article	Approach name after redefinition	Specific description of approach
Abdel2017	2-incision	2-incision	For the two-incision technique, the surgical approach involved a 6-cm anterior incision and dissection through the Smith-Petersen interval to expose the hip, to cut the femoral neck, and to prepare the socket. A second incision of 3.8 to 5 cm was then made in the buttock, and the abductors and external rotators were identified and were protected with use of a cannula, through which the reamers were placed. The femur was then reamed, and the femoral component was placed through that posterior incision. (PMID:18451391)
Abdel2017	Mini-Posterior	MIS-PA	For the mini-posterior-incision technique, the surgical approach involved a 7 to 9.5-cm incision along the posterior aspect of the femur, starting at the tip of the greater trochanter and proceeding distally. The fascia of the gluteus maximus was split, and blunt dissection revealed the underlying abductor and external rotator musculature. The external rotators and the hip capsule were incised and preserved as one layer, with an attempt being made to preserve the insertion of the quadratus femoris on the femur. The hip was dislocated posteriorly, and the femoral neck

© 2023 Yan L et al. *JAMA Network Open.*

Study	Approach name in the article	Approach name after redefinition	Specific description of approach
			was cut in accordance with the preoperative plan. Acetabular retractors were positioned, the acetabulum was reamed, and the real acetabular component was placed. The hip was then flexed, and retractors were placed around the femoral neck to allow reaming, broaching, and trial insertion of the femoral component. The femoral component was then impacted into place, the femoral head was assembled, and the hip was reduced. The hip capsule and the external rotators were meticulously repaired back to the greater trochanter through three drill-holes with use of nonabsorbable sutures that were placed in a locking-looped fashion. (PMID:18451391)
Barrett2019	DAA	DAA	The direct anterior approach utilizes a modern fracture table with the patient placed supine, both feet in boots for proper positioning. An anterior skin incision, 10–14 cm long, is used. An inter-muscular plane is utilized to access the anterior hip capsule. The hip capsule is opened anteriorly, a femoral neck osteotomy is performed based on pre-operative templating, and the femoral head removed. Acetabular retractors are placed and reaming of the acetabulum commenced. This is done under direct visualization with C-arm confirmation for positioning. The femoral side is then visualized with the aid of the fracture table. A hydraulic trochanteric hook elevates the proximal femur. Broaching of the femoral canal is started and proceeds up to the appropriate size. A trial reduction is performed, and the length and offset are checked manually and with C-arm confirmation. The trial components are removed and the prostheses are placed with press-fit fixation. Routine closure is performed. (PMID: 23523485)
Barrett2019	PA	PA	This approach uses a standard OR table with the patient placed in the lateral

© 2023 Yan L et al. *JAMA Network Open.*

Study	Approach name in the article	Approach name after redefinition	Specific description of approach
			decubitus position. A 10–14 cm skin incision is utilized over the posterior-lateral corner of the hip. The gluteus maximus muscle is split in line with its fibers and the short external rotators and posterior capsule are opened. The hip is dislocated posteriorly and a femoral neck osteotomy is performed. The acetabular and femoral components are inserted in the same manner as is done with the DAA with press fit fixation utilized. (PMID: 23523485)
Bon2019	anterior approach (AA)	DAA	AA was Hueter's anterior approach, performed with the patient supine on the traction table, without intraoperative radioscopic control. (PMID: 30853454)
Bon2019	PA	**MIS-PA**	PA was Moore's posterolateral approach, sparing the quadratus femoris muscle but not the piriformis, with transosseous reinsertion of the pelvitrochanteric muscles and capsule. (PMID: 30853454)
Brismar2018	direct anterior (DA)	DAA	The DA was carried out with the patient supine on a standard operating table allowing angulation at the level of the hip. The skin was incised at a point 2 fingerbreadths lateral to the anterior sciatic spine and extended 8–10 cm distally. The tensor fascia lata and gluteus medius muscles were retracted laterally and the sartorius and rectus muscles medially exposing the capsule. A special offset acetabular reamer and an offset broach handle were used. (PMID: 30350758)
Brismar2018	direct lateral (DL)	DLA	The DL was performed with the patient in a lateral decubitus position. Access to the joint was gained through a 10–20 cm long skin incision centered over the greater trochanter, splitting the fascia lata/gluteus maximus and detachment of the caudal 2/3 of the gluteus medius and the entire gluteus minimus tendon insertions. Finally, the capsule was excised anteriorly. The muscle tendons were reattached to the trochanter by osteosutures following implantation. (PMID:

© 2023 Yan L et al. *JAMA Network Open.*

Study	Approach name in the article	Approach name after redefinition	Specific description of approach
			30350758)
Cao2020	DAA	DAA	In this approach, the patient is positioned in a supine position on a regular operating table. A skin incision, around 8 cm long, is made along the inferolateral of the anterior superior iliac spine, towards the fibular head. The anterior hip capsule is exposed through the space between the tensor fascia lata and the rectus femoris. The ascending branch of the lateral femoral artery is found and ligation performed while it is exposed. After opening the hip capsule anteriorly, a measured femoral neck osteotomy is performed, based on results of preoperative template measurement, after which the femoral head is removed. After this, the acetabular reaming is performed and the acetabular component inserted. The operative limb is sufficiently externally rotated, adducted and stretched. The femoral canal is broached to the appropriate size, using the hook to raise the proximal femur for optimal exposure and operation. The femoral implant and head are placed following a trial reduction using the femoral implant trial to ensure leg length and offset suitability. (PMID: 32487264)
Cao2020	Posterolateral approach	PA	In this technique, the patient is positioned in the lateral decubitus position on a regular operating table. A 10–15 cm curvilinear incision is placed over the greater trochanter at the posterolateral aspect of the hip. A blunt dissection of gluteus maximus in line with its fibers is executed in order to reach the short external rotators and open the posterior capsule. A femoral neck osteotomy is then performed following the posterior dislocation of the hip joint. The acetabular and femur are prepared, and these components are then inserted into the appropriate location after trialing. The C-arm is used to confirm leg length and offset. Finally,

© 2023 Yan L et al. *JAMA Network Open.*

Study	Approach name in the article	Approach name after redefinition	Specific description of approach
			the articular capsule is repaired, but the external rotator is not reconstructed in the muscle group. Closure is performed as standard. (PMID: 32487264)
Catma2017	anterolateral	**MIS-ALA**	After an anterolateral incision, the space between tensor fascia and gluteus medius muscles was used to reach joint capsule and femoral head. Femoral head was removed and femur was reamed in each group. Femur was rasped with proper size. (PMID: 28659053)
Catma2017	PA	PA	The posterior approach was familiar with modification of the Gibson–Moore approach. After a posterior curve skin incision, external rotator muscles and tendons were revealed and hanged with a suture. Elongated joint capsule was exposed and femoral head was revealed with external rotation of the femur. (PMID: 28659053)
Cheng2017	DAA	DAA	An orthopaedic traction table (Maquet, Rastatt, Germany) was utilized for all DAA THAs. The anterior incision begins 3cm posterior and distal to the ASIS, extending distally approximately 10cm over the tensor fascia lata. Hueter's interval was then identified and developed to gain access to the hip joint. A capsulotomy and femoral neck osteotomy was performed. This was followed by the retrieval of the femoral head and repositioning of retractors to expose the acetabulum. Sequential reaming and acetabular component implantation was conducted and verified under fluoroscopy. Femoral preparation was undertaken with the leg extended, externally rotated and adducted. A superior capsulotomy was performed to aid in femoral exposure. Femoral broaching and trials were performed with fluoroscopic assistance. Definitive implantation of the remaining prosthesis was undertaken with routine capsular and wound closure. (PMID:

© 2023 Yan L et al. *JAMA Network Open.*

Study	Approach name in the article	Approach name after redefinition	Specific description of approach
			27687805)
Cheng2017	PA	PA	PA surgery was performed with the patient adopting a lateral position on a standard surgical table. The curvilinear incision 10-15 cm long centers over the posterior third of the greater trochanter. Dissection through the fascia in line with the fibres of gluteus maximus was conducted to reach the short external rotators. With the piriformis muscle identified, the short external rotators and hip capsule were tagged and reflected. Subsequent hip joint dislocation was followed by a femoral neck osteotomy at the templated level. Acetabular and femoral preparations were then performed in a routine manner. Definitive implants were trialled and inserted under direct vision. An enhanced intraosseous short rotator and capsular repair was performed for all cases. (PMID: 27687805)
Chimento2005	standard posterolateral approach	**PA**	The surgeries were performed either through a 15-cm incision using a standard posterolateral approach or an 8-cm incision using a modified posterolateral minimally invasive approach. Smaller specialized retractors are used. The incision is centered over the posterior aspect of the greater trochanter. The short external rotators and capsule are taken as a unit and tagged for later repair. The quadratus femoris is spared, as is the femoral insertion of the gluteus maximus. The quadratus and gluteal insertions are released in the 15-cm incision. (PMID: 15902851)
Chimento2005	modified posterolateral minimally invasive approach	**MIS-PA**	
Christensen2015	DAA	DAA	DAA THA was performed with the patient supine on a fracture table. An anterior incision was made from 3 cm lateral to the anterior superior iliac spine distally to the vastus ridge. No soft tissue undermining was performed. The fascia was divided in line with skin incision and the tensor fascia musculature was retracted

© 2023 Yan L et al. *JAMA Network Open.*

Study	Approach name in the article	Approach name after redefinition	Specific description of approach
			laterally. No wound towels or skin protecting devices were used. The rectus femoris was retracted medially, and the anterior circumflex vessels were identified, tied off, and divided, and the anterior capsule was excised. (PMID: 24890998) 同一个团队，之前发的文章
Christensen2015	PA	PA	PA THA was performed with the patient positioned in the lateral decubitus position. A posterolateral incision was utilized and the fascia was divided in line with the skin incision. The short external rotators and capsule were tagged in separate layers, and the femoris quadratis was preserved. (PMID: 24890998)
D'Arrigo2009	minimally invasive anterior	DAA	An anterior TSS approach utilising the interval between the tensor fasciae latae, gluteus medius and minimus muscle laterally and the sartorius and rectus femoris muscle medially was used. (PMID: 19384637)
D'Arrigo2009	lateral with mini incision	MIS-DLA	We used a modified Hardinge approach in which the anterior third of the gluteus medius and the underlying minimus is reflected anteriorly. The length of the skin incision to be made was measured and marked using a sterile ruler and marker pen after draping. The only difference from the modified Hardinge approach (control group) was the length of the skin incision (8 cm instead of 12–15 cm). (PMID: 19384637)
D'Arrigo2009	minimally invasive antero-lateral	MIS-ALA	An antero-lateral TSS approach utilising the intermuscular plane between gluteus medius and tensor fascia latae was used. (PMID: 19384637)
D'Arrigo2009	lateral direct Hardinge approach	DLA	For the control group, we used a lateral direct Hardinge approach with a cementless component. (PMID: 19384637)

© 2023 Yan L et al. *JAMA Network Open.*

Study	Approach name in the article	Approach name after redefinition	Specific description of approach
De2016	direct anterior	DAA	Arthrotomy was performed by retracting the muscles rectus femoris and iliopsoas medially and gluteus medius laterally. (PMID: 26753844)
De2016	lateral	DLA	In the lateral group, a direct lateral approach as described by Hardinge was used. Briefly, the gluteus medius and minimus were incised and detached ventrally from the greater trochanter. The incision was not extended more than 3 cm above greater trochanter to prevent injury to superior gluteal nerve. After implantation, the tendons were reattached with transperiosteal sutures. (PMID: 26753844)
Della2010	2-incision	2-incision	The two-incision technique was performed as described by Berger. An incision is made directly over the femoral neck from the base of the femoral head distally 1.5 inches to expose the fascia. The sartorius muscle is present in the proximomedial incision whereas the tensor fascia lata lies at the distal lateral portion of the incision. The sartorius is retracted medially and the tensor fascia lata is retracted laterally. A 1.25-inch incision is made in the posterior lateral buttocks, colinear with the pir- iformis fossa allowing access to the femoral canal. A Charnley awl is guided through this incision, down the femoral canal, posterior to the abductors, anterior to the piriformis fossa with the aid of fluoroscopy. (PMID: 14646722)
Della2010	mini-posterior	MIS-PA	The mini-posterior approach was performed with the patient in the lateral decubitus position. A straight incision of 7 to 10 cm in length was made over the posterior border of the greater trochanter and the gluteus maximus muscle was split in line with its fibers. The short external rotators were released including the piriformis; however, the quadratus femoris was preserved. The posterior capsule

© 2023 Yan L et al. *JAMA Network Open.*

Study	Approach name in the article	Approach name after redefinition	Specific description of approach
			and short external rotators were tagged and repaired at the end of the procedure. (PMID: 20668969)
Dienstknecht 2014	Micro-hip	**DAA**	The Micro-hip approach adopted the modified Smith-Petersen approach. Patients were positioned in a lateral decubitus position. The skin midway between the greater trochanter and the anterior superior iliac spine was incised. The subcutis and fascia were dissected, followed by the interval between the tensor fascia lata muscle and the rectus muscle. The joint capsule was split and left in place. The femoral neck was osteotomised and the femoral head removed. (PMID: 25163948). Next a straight Hohmann retractor is inserted between the tensor facia late muscle and the sartorius muscle, with the tip coming to rest on the femoral neck at the bottom of the greater trochanter. The Tesor muscle is then retracted lat- erally, together with the abductor Medius and Minimus muscle. A second retractor is placed on the femoral calcar to retract the Sartorius and Rectus muscles ventrally. This will expose the capsule over the femoral neck. (PMID: 17514174)
Dienstknecht 2014	Bauer approach	**DLA**	For the Bauer approach, patients were positioned supine. A slightly dorsally arcuated incision was made over the greater trochanter region.The subcutis and fascia lata were incised parallel to the skin incision, and the gluteal medius and minimus muscles were split along the line of their fibres. The joint capsule was split and left in place. (PMID: 25163948)
Dorr2007	Posterior Conventional	PA	The incision must be made over the posterior one-third of the trochanter and, the bigger the patient is, the more poste- rior the incision must be. The incision extends from the level of the vastus tubercle at the distal end of the greater
Dorr2007	Posterior	MIS-PA	

© 2023 Yan L et al. *JAMA Network Open.*

Study	Approach name in the article	Approach name after redefinition	Specific description of approach
	Minimally Invasive		trochan- ter proximally to 3 cm cephalad to the posterior tubercle of the greater trochanter. The first incision into hip tissue is done in the gluteus maximus muscle, which is incised for 6 to 8 cm along the posterior border of the greater trochanter. The second incision into hip tissue is through the small external rotators and the posterior capsule. This incision is made as a single flap from just proximal to the quadratus femoris muscle through the piriformis tendon, including 3 cm of the gluteus minimus muscle that lies under the piriformis ten- don. The third incision into hip tissue is the inferior medial capsule, which is incised from the anterior femur to the ace- tabulum through the transverse acetabular ligament.<br>(DOI: 10.1053/j.sart.2005.10.003)
Dutka2007	standard direct lateral approach	DLA	The greater trochanter thus presents in the centre of the incision. A small prominence lies at the uppermost end of the ridge of the vastus lateralis and starting at this point the tendon of the gluteus medius is incised using a diathermy needle but leaving a cuff of tendon still attached to the greater trochanter. (PMID: 7068713) A minimally invasive direct lateral approach (6-8cm), the standard direct lateral approach (20-25cm). (PMID: 17514173)
Dutka2007	minimally invasive direct lateral approach	MIS-DLA	
Goosen2011	modified anterolateral-MIS	MIS-ALA	"anterolateral" refers to approaching the hip anteriorly from the greater trochanter according to the guidelines of Frndak et al. (PMID: 20352383) The term "split" refers to the separation of cleavage within the abductor muscle mass. The surgeon palpates the neck of the femur beneath the abductor muscle mass, locating the femoral neck (PMID: 8403638)

© 2023 Yan L et al. *JAMA Network Open.*

Study	Approach name in the article	Approach name after redefinition	Specific description of approach
Goosen2011	posterolateral-MIS	MIS-PA	Approaching the hip posteriorly from the greater trochanter according to the criteria of Gibson. The MIS procedures are described as a small-incision technique in which the quantitative skin and muscle dissection of the gluteus muscles has been reduced with respect to the classic approach. Specially designed retractors and instruments were used. (PMID: 20352383)
Goosen2011	posterolateral-CLASS	**PA**	
Hu2012	2-incision	2-incision	For the two-incision THA, the patient was positioned in a lateral position as described by Lee et al. Dissection was carried out between the sartorius and tensor fasciae latae superficially and between the gluteus medius and rectus femoris underneath. A special acetabular reamer and cup inserter were used for the acetabular side from the anterior wound. Another posterior incision was made through the gluteus maximus superficially and between the piriformis and gluteus medius underneath for femoral preparation. The femoral canal was prepared with a rasp and reamer. (PMID: 22483428)
Hu2012	modified Watson-Jones	**MIS-ALA**	For the modified Watson-Jones THA, the patient was positioned in the lateral position on a special operating table in which one foot piece could be removed to facilitate hyperextension, external rotation, and adduction of the hip. The surgical procedure was followed the steps described by Bertin and Röttinger. Dissection was carried out between the tensor fasciae latae and the gluteus medius. A special acetabular reamer and cup inserter were used. On the femoral side, a special dog-legged broach handle and curved retractors were used. (PMID: 22483428)
Inaba2011	modified mini-incision direct lateral approach	MIS-DLA	In the modified mini-incision direct lateral approach group, a 7-cm skin incision was made on the lateral side of the hip. The anterior 30% to 40% of the gluteus medius and minimus were incised to a maximum of 3 cm, and the incision was

© 2023 Yan L et al. *JAMA Network Open.*

Study	Approach name in the article	Approach name after redefinition	Specific description of approach
			opened along the fiber course for dissection of the hip joint. Compared with the muscle-sparing approach, it was easier to expose the femur and acetabulum by separating the gluteus muscles in this approach. In our modified mini-incision direct lateral approach, the anterior part of the gluteus medius was not detached from the greater trochanter, and muscle splitting was only performed within the gluteus muscles; extension of the incision into the vastus lateralis was strictly avoided. The divided abductors were repaired after implantation. (PMID: 21602025)
Inaba2011	modified Watson-Jones approach	**MIS-ALA**	In the muscle-sparing group, a modified Watson-Jones approach was used; that is, an 8cm incision was made through the intermuscular interval between the gluteus medius and the tensor fascia lata. This approach provided good exposure of the hip joint while preserving muscle integrity. (PMID: 21602025)
Ji2012	Posterior Approach	PA	For the posterior approach, we used the technique described by Kocher and Langenbeck. The patient was transferred to the lateral decubitus position and the hip was flexed by 30°. A straight skin incision was made over the center of the greater trochanter, equidistant cephalad, and caudad to the center of the trochanter. The length of skin incision ranged from 16 to 22 cm. The fascia lata was incised between the muscle bellies of the tensor fascia lata and the gluteus maximus. The trochanteric bursa was incised, and fat tissue overlying short external rotators were removed to identify the posterior borders of the gluteus medius and the short external rotators. Short external rotators were detached with electrocautery as close as possible from their insertion. After reflecting these muscles, the posterior capsule was exposed along the base of the neck. Using

© 2023 Yan L et al. *JAMA Network Open.*

Study	Approach name in the article	Approach name after redefinition	Specific description of approach
			electrocautery, the capsule was incised from the acetabular labrum to the upper portion of the lesser trochanter along the base of the neck. A trapezoidal posteriorly broad-based capsular flap was created. The femoral head was dislocated posteriorly from the acetabulum with flexion and internal rotation of the femur. (PMID: 21802253)
Ji2012	modified lateral approach	**DLA**	For the modified lateral approach, we used the operative technique described by Mulliken et al. The patient was transferred to the lateral decubitus position and the hip was flexed by 30°. A straight lateral skin incision was made over the center of the greater trochanter midway between the anterior and posterior dimensions of the greater trochanter. The length of skin incision was similar to that of the posterior approach. The fascia lata was incised between the muscle bellies of the tensor fascia lata and the gluteus maximus. Muscle fibers of the gluteus medius were separated at its anterior middle one-third junction, up to 3 cm cephalad to its insertion. The combined tendon and periosteum of the gluteus medius and vastus lateralis were separated and detached with electrocautery. This division was carried anterior to the trochanter to leave behind a posterior tendinous cuff for later suturing. Distally, the incision was curved posteriorly at the vastus ridge and taken in line with the fibers of the vastus lateralis. A plane between the gluteus minimus and anterior capsule was found proximally. Blunt dissection was carried out in this plane to the acetabular rim, identifying and cutting the reflected head of the rectus femoris. With adequate exposure of the anterior capsule, an anterior capsulectomy was performed. The femoral head was dislocated anteriorly from the acetabulum with extension and external rotation of the femur. (PMID:

© 2023 Yan L et al. *JAMA Network Open.*

Study	Approach name in the article	Approach name after redefinition	Specific description of approach
			21802253)
Khan2012	standard posterior approach	PA	The piriformis-sparing approach has been described in detail. In summary, it involves a short (approximately 7 cm to 10 cm) oblique incision over the posterior aspect of the greater trochanter. The capsule is incised along the inferior border of piriformis from the edge of the acetabulum to the posterior border of the femur, continuing distally in an 'L' shape, detaching the capsule, gemelli and obturator internus as one. Quadratus femoris may be partially detached, as required, to visualise the inferior part of the neck. The hip is then dislocated and osteotomy of the neck performed in the normal manner. After insertion of the component a combined capsulotendinous repair is performed through two drill holes in the bone. Drill holes are placed from lateral to medial at the posterior aspect of the greater trochanter, resulting in an anatomical repair to their correct point of attachment. Apart from the longer incision (approximately 20 cm) and the division and repair of piriformis using the standard posterior approach, there is no other difference between the two approaches. (PMID: 22219246)
Khan2012	piriformis-sparing approach	**MIS-PA**	
Kim2006	modified posterolateral minimally invasive approach	**MIS-PA**	The surgeries were performed either through a 15- to 20-cm incision using a standard posterolateral approach or an 8-cm incision using a modified posterolateral minimally invasive approach. Smaller specialized retractors were used. The incision was centered over the posterior aspect of the greater trochanter. The short external rotators and posterior capsule were taken as a unit and tagged for repair in both approaches. The quadratus femoris was spared, as was the femoral insertion of the gluteus maximus in the minimally invasive technique. On the contrary, the quadratus femoris and gluteus maximus insertions were
Kim2006	standard posterolateral approach	PA	

© 2023 Yan L et al. *JAMA Network Open.*

Study	Approach name in the article	Approach name after redefinition	Specific description of approach
			released in the standard technique. Angled acetabular reamer and angled cup inserter were used in the minimally invasive technique, and regular straight acetabular reamer and straight cup inserter were used in the standard technique. The short external rotators and posterior capsule were repaired in both groups to the greater trochanter through a drill hole with nonabsorbable sutures. (PMID: 17162166)
Korykin2021	mini posterior approach (MPA)	MIS-PA	MPA THAs were operated on as reported by Inaba et al. The first inci- sion into hip tissue was done in the gluteus maximus muscle, which was incised for 6 to 8 cm along the posterior border of the greater trochanter. The second incision into hip tissue was through the small external rotators and the posterior capsule. This incision was made as a single flap from just proximal to the quadratus femoris muscle through the piriformis tendon, including 3 cm of the gluteus minimus muscle which lies under the piriformis tendon. The third incision into hip tissue was the inferior medial capsule, which was incised from the anterior femur to the acetabulum through the transverse acetabular ligament. (PMID: 16330992)
Korykin2021	SuperPATH	SuperPath	Patients in the SuperPATH group were treated according to the technique introduced by Chow et al. and described by Della Torre et al. SuperPATH utilizes powerful elements of both proce- dures. Preparing the hip in-situ allows the operative leg to rest on a Mayo stand during the entire procedure, obviating the need for a second assistant. Additionally, since the hip is not dislocated, the interval between the gluteus medius and piriformis is utilized, and the piriformis can be preserved in a majority of cases. Utilizing the percutaneous accessory portal for acetabular preparation keeps the wound visualization un-obscured by

© 2023 Yan L et al. *JAMA Network Open.*

Study	Approach name in the article	Approach name after redefinition	Specific description of approach
			tooling. This allows the procedure to be done safely through the smaller window created by an intact piriformis. The accessory portal also provides in-line access to the cup, simplifying the insertion of screws, and facilitating impaction of bone-ingrowth components. (PMID: 21789576)
Laffosse2008	Anterolateral minimally invasive modified Watson Jones approach	MIS-ALA	The patient was positioned in a true lateral position. The posterior and distal part of the operating table was removed. An incision of 8 cm is located 5 cm distal to the top of the greater trochanter from the anterior side and directed toward the anterior superior spine of the pelvis. The superficial fascia was opened. The intermuscular plane between the tensor fascia lata and the anterior side of the gluteus medius was developed until contact with the femoral neck. The lower limb was put in a position of slight flexion with a maximal external rotation to expose the anterior and inferior parts of the capsule with two Hohmann retractors. The capsule should be opened in "H" widely at its anterior superior acetabulum insertion, where the labrum was resected which helps the dislocation. The hip was dislocated or the neck was severed in place. The limb was placed in extension of 30°-adduction of 30°-external rotation of 90°; the vertical leg was protected by a sterile drape. The assistant keeps the limb in this position while pushing on the knee to exteriorize the femur with the aid of a retractor placed at the anteromedial side of the neck under the calcar. This position is called the "femoral position". The neck was cut at the level selected depending on the preoperative planning. (PMID: 17639434)
Laffosse2008	Posterior minimally	MIS-PA	The patient was positioned on the operating table in the lateral position. The 8 cm posterior incision ran from a point situated 5 cm distal to the top of the greater

© 2023 Yan L et al. *JAMA Network Open.*

Study	Approach name in the article	Approach name after redefinition	Specific description of approach
	invasive approach		trochanter and was directed toward the posterior–superior spine of the pelvis. The gluteus maximus was split along its fibers. The sciatic nerve was palpated. The posterior medial circumXex artery was then ligated in the quadratus femoris muscle. The limb was internally rotated, the superior gemellus, the obturator internus, the inferior gemellus were detached close to the femur. Dislocation of the hip was achieved by flexion, adduction and internal rotation. An axial load was applied to limit facilitate exposure of the femoral neck. As the lesser trochanter was not exposed, the trochanteric fossa was used as a bony landmark. The height of the neck cut was determined preoperatively by considering the thickness of the acetabular metal-back and polyethylene and the diameter of the head. The amount of neck to be removed was determined and represented by the distance between the top of the head and the cut. The distance was then reported peroperatively with a caliper. An assistant kept always the member vertical, flexed-adducted-rotated internally in order to control the anteversion of the rasp and the final stem. The external posterior part of the greater trochanter was scooped out with a gouge to prevent the tilting of femoral rasp. The femur was prepared normally. (PMID: 17639434)
Landgraeber 2013	lateral approach	DLA	In the conventional approach group the patients were placed in the supine position and a modified Bauer respectively Hardinge approach was performed according to Thomine. (PMID: 24191179) (PMID: 10507117)
Landgraeber 2013	minimally invasive	MIS-ALA	The patients in the MIS group were positioned on the operating table in the lateral position and the surgical procedure was performed as described by Bertin and Röttinger. (PMID: 24191179) (PMID: 15577495)

© 2023 Yan L et al. *JAMA Network Open.*

Study	Approach name in the article	Approach name after redefinition	Specific description of approach
	anterolateral approach		
Li2021	SuperPath	SuperPath	For SuperPath group, the patients received combined spinalepidural anesthesia and were set to the position of 45 degrees flexion of the affected hip joint. Under this position, the Internal rotation of the lower limbs was 10-15 degrees to ensure that the greater trochanter was upward. The incision was from the tip of greater trochanter to 6-8 cm along with the long axis of femur. After the gluteus maximus was split, the deep layer was further peeled and the gluteus medius was retracted until the interspace between piriformis and gluteus minimus was exposed. After separating the interspace and exposing the articular capsule, the articular capsule was incised longitudinally. The femoral neck osteotomewas used for slotting at the position of saddle of trochanteric fossa and the cancellous bone of the femoral neck was scraped off. After proximal reaming, the slope of the pulp cavity file was taken as the reference baseline and the femoral neck was sawed off. The femoral head was removed and the round ligament in acetabulum and residual soft tissue on labrum of pelvis were cleaned. Then, the femur was pulled forward using the trochanter retractor and the cutaneous channel was set in the posterior femoral space. (PMID: 33262048)
Li2021	conventional posterolateral approach	**PA**	For the conventional group, the patients received combined spinal-epidural anesthesia. A 10-14 cm arc incision was made around the tip of greater trochanter and the tissues were cut layer by layer. After the gluteus maximus was split, gluteus medius, circumflex muscles and quadratus femoris were exposed and the external circumflex muscles and part of quadratus femoris were cut off. Then, the

© 2023 Yan L et al. *JAMA Network Open.*

Study	Approach name in the article	Approach name after redefinition	Specific description of approach
			articular capsule was incised longitudinally and the femoral neck was exposed. Generally, the femoral neck was cut off 1-1.5 cm above the lesser trochanter after dislocation of hip joint. The femoral head was removed and acetabulum was exposed clearly. Then, hyperplastic osteophyte and labium were removed and the acetabulum was grinded until the ooze of subchondral bone. (PMID: 33262048)
Martin2011	standard lateral transgluteal Hardinge approach	DLA	The lateral Hardinge approach was modified according to Thomine et al. The anterior half of the gluteus medius and anterior third of the gluteus minimus tendons were elevated and subsequently repaired. (PMID: 21435823)
Martin2011	Anterolateral minimally invasive hip surgery (ALMIS)	MIS-ALA	The ALMIS approach was performed as described by Bertin and Röttinger. Patients were positioned in a lateral position, and the distal part of the table was removed. An 8- to 10-cm incision was made in line with anterior superior iliac spine and the anterior aspect of the greater trochanter. The intermuscular plane between tensor fascia lata and gluteus medius was exposed, and a Ushaped capsulotomy was made. Femoral neck was osteotomized and removed. The operative leg was kept in external rotation during acetabular reaming. (PMID: 21435823)
Matziolis2011	minimized transgluteal (TG)	**MIS-DLA**	20 using the minimally invasive transgluteal approach. (PMID: 20953874)
Matziolis2011	anterolateral (AL)	**MIS-ALA**	20 patients were treated using the minimally invasive anterolateral approach. (PMID: 20953874) (PMID: 18071930)

© 2023 Yan L et al. *JAMA Network Open.*

Study	Approach name in the article	Approach name after redefinition	Specific description of approach
Mayr2009	minimally invasive direct anterior approach	DAA	With the patient in the supine position, a 7 cm skin incision was made distally and laterally to the anterior superior iliac spine. The anterior aspect of the capsule of the hip was bluntly exposed by holding apart the rectus femoris muscle medially and the gluteus minimus muscle laterally (Krismer and Rachbauer, 2004). Following capsulotomy, the femoral neck was osteotomized. The reaming of the cup was performed with angulated reamers. Next, the femur was externally rotated and the capsule carefully detached from the greater trochanter. The entrance into the medullary canal was lifted to achieve unimpaired access for the offset broaches. A special two-pronged retractor was inserted between the tendons of the gluteus medius and minimus and the greater trochanter to provide additional leverage. The adducted femur was broached for a cementless stem. Since no muscles were split, the fascia between the Sartorius muscle and tensor muscle was sutured. The subcutaneous fat and skin were sutured. (PMID: 19699566)
Mayr2009	traditional anterolateral approach (AL)	**DLA**	The patient was placed in the supine position. After skin incision over the greater trochanter, the iliotibial band was split. The ventral third of vastus lateralis muscle and the gluteal muscle was detached from the bone in one coherent layer using diathermy (Bauer et al., 1979). The exposed capsule was then opened, and the femoral head was dislocated. Following osteotomy of the femoral neck, the cup was reamed for a cementless cup. Next, with a blunt Hohmann retractor, the femur was levered over the iliotibial band. The femur was externally rotated and adducted and the external rotators near the intertrochanteric fossa were tenotomized. While holding back the gluteal muscles, the femur was broached for

© 2023 Yan L et al. *JAMA Network Open.*

Study	Approach name in the article	Approach name after redefinition	Specific description of approach
			an uncemented stem. After implantation, the gluteus medius and vastus lateralis was adapted. Then, the fascia latae was closed. (PMID: 19699566)
Mazoochain2009	Standard lateral approach	DLA	The approach in the Standard group was the lateral approach by Bauer. (PMID: 19424709)
Mazoochain2009	modified lateral approach by Hardinge	**MIS-DLA**	The patient is placed in a supine position with the greater trochanter lying at the edge of the table. The skin incision is performed over the trochanter proximally in a slight dorsal direction and has a length of approximately 8 cm. The iliotibial band is incised parallel to the skin incision. The gluteus medius and minimus muscle are incised with a Bovey diathermy needle 2 cm proximally from the top of the greater trochanter in their tendineous portion in the direction of their fibers and detached from the capsula in a subperiostal manner in one sleeve. The vastus lateralis remains untouched. The splitting of the muscle of 2 cm is inside the so-called safe-zone which is approximately 4–5 cm over the top of the greater trochanter. Thus the superior gluteal nerve is not injured. The following the surgery is performed as usual with incision of the capsule and the luxation of the femoral head. (PMID: 19424709)
Meneghini2008	2-incision MIS approach	2-incision	The 2-incision MIS THA was performed as originally developed by Mears and popularized by Berger. (PMID: 18722305)
Meneghini2008	posterolateral MIS approach	**MIS-PA**	The mini-posterior approach was performed similar to that described by Dorr et al. (PMID: 18722305)
Meneghini2008	anterolateral approach	**MIS-DLA**	The mini-anterolateral approach was performed as described by Berger and is a modification of the Hardinge approach with elevation and subsequent repair of the anterior one third of the gluteus medius and minimus tendons. (PMID:

© 2023 Yan L et al. *JAMA Network Open.*

Study	Approach name in the article	Approach name after redefinition	Specific description of approach
			18722305)
Meng2021	SuperPath	SuperPath	This superior portal-assisted approach is proposed to access the hip capsule through the interval between the gluteus medius and piriformis and to preserve the periarticular soft tissues. (PMID: 33842613)
Meng2021	mini-incision posterolateral	**PA**	The conventional posterolateral approach (PLA) is the most widely utilized approach for THA, with excellent exposure for both primary and revision THA. Compared with the PLA group, the SuperPath group yielded a significantly shorter incision length (7.83 *vs.* 12.45 cm, P<0.001). (PMID: 33842613)
Mjaaland2019	Direct Anterior	DAA	The direct anterior approach was performed with the patient in the supine position on a standard operating table. A slightly oblique skin incision measuring approximately 8 cm was used, staring 3 cm distally and laterally to the superoanterior iliac spine. The subcutaneous tissue and the fascia centrally over the tensor fascia lata muscle were divided followed by blunt dissection to open the interval between the tensor facia lata and the sartorius muscle. The lateral circumflex arteries were identified and cauterized. The joint capsule was exposed and the anterior portion removed. (PMID: 30179928)
Mjaaland2019	Direct Lateral	DLA	The direct lateral approach was performed with the patient in a lateral decubitus position. A straight skin incision, measuring approximately 14 cm, centered over the greater trochanter was used. The subcutaneous tissue and the fascia lata were divided in line with the skin incision. The anterior third of the gluteus medius along with the gluteus minimus was released from the greater trochanter followed by exposure and removal of the anterior part of the joint capsule. The hip was dislocated, and an osteotomy was performed after releasing the capsule down to

© 2023 Yan L et al. *JAMA Network Open.*

Study	Approach name in the article	Approach name after redefinition	Specific description of approach
			the lesser trochanter to decide the level of the osteotomy compared with the preoperative template. (PMID: 30179928)
Moerenhout2020	DAA	DAA	The modified Hueter approach, based on the Smith-Peterson approach, was performed for the direct anterior minimally invasive surgery. A traction table was used for DAA as the surgeons were trained to use this method. No intraoperative fluoroscopy was used for implant confirmation. (PMID: 33009898)
Moerenhout2020	posterior approach	**MIS-PA**	The PA to the hip, which is considered to be a minimally invasive approach because of the smaller operative scar (under 10 cm), has been described by many authors potentially yield better results and could The modified Hueter approach, based on the Smith-Peterson approach, was performed for the direct anterior minimally invasive surgery. (PMID: 33009898)
Muller2011	modified direct lateral	**MIS-DLA**	The lateral approach was described initially by Bauer and Hardinge. Compared to the approach of Bauer and Hardinge, in the modified form the skin incision was minimized to approximately 10 cm. The gluteus medius was incised along the fiber course to a maximum length of 3 cm to protect the inferior branch of the superior gluteal nerve. To expose the joint capsule, the anterior third of the gluteus medius was detached together with the underlying gluteus minimus into ventrally from the trochanter major. Lengthening of the incision into the vastus lateralis was strictly avoided. The glutei tendons were refixed to the trochanter with two or three periosteal sutures. (PMID: 20490520)
Muller2011	minimally invasive anterolateral	MIS-ALA	The minimally invasive anterolateral approach is a modified Watson–Jones approach and was introduced by Bertin and Rottinger. This approach uses the intermuscular plane between the gluteus medius and the tensor fascia latae and

© 2023 Yan L et al. *JAMA Network Open.*

Study	Approach name in the article	Approach name after redefinition	Specific description of approach
			provides good exposure to the hip joint while preserving muscle integrity. (PMID: 20490520)
Muller2012	modified direct-lateral approach (mDL)	**MIS-DLA**	The intramuscular mDL-approach is a modiWed technique of the initial Bauer and Hardinge approach. The modification comprises shorter incisions than more traditional procedures: the approach is conducted using approximately a 10-cm skin incision, the gluteus medius muscle is incised by a maximum of 3 cm and the incision is extended only to the aponeurosis of the vastus lateralis muscle at the greater trochanter. The ventral aspect of the gluteus medius is then detached from the greater trochanter together with the underlying gluteus minimus. After implantation, the reinsertion is effected by two or three periostal sutures. (PMID: 22294091)
Muller2012	minimally invasive anterolateral approach (ALMI)	**MIS-ALA**	The ALMI approach is a modified version of the approach described by Watson-Jones. The preparation of the hip joint is performed intermuscularly between the gluteus medius and the tensor fasciae latae (TFL) without incising or detaching muscle or tendon fibres. While the muscle-sparing aspect of the approach is advantageous, the limited overview, the higher risk of trochanteric fractures and the difficult preparation of the proximal femur are all known detrimental factors. (PMID: 22294091)
Nistor2017	direct anterior approach	DAA	The DAA group underwent the THA through a modified Smith-Peterson direct anterior approach as described by Lovell, in a supine position, on a standard operating table that could be flexed so that hip hyperextension could be achieved. Both legs were completely draped separately to facilitate proximal femoral exposure (e.g. extension, adduction and external rotation with the operative leg

© 2023 Yan L et al. *JAMA Network Open.*

Study	Approach name in the article	Approach name after redefinition	Specific description of approach
			underneath the nonoperative leg). An 8 cm skin incision was made over the body of the tensor fascia lata muscle (TFL) and then lengthened as needed for a proper exposure. The fascia of the TFL was incised lengthwise and the TFL muscle dissected and retracted laterally. After coagulation of the anterior femoral circumflex vessels, the anterior capsulectomy was performed and joint exposure was accomplished. (PMID: 28439629)
Nistor2017	lateral approach (LA)	DLA	For the LA group, a direct lateral approach was used to perform the THA as described by Hardinge. With the patient on a standard operating table, in a supine position, skin incision was initiated 3 cm proximal to the tip of the greater trochanter and was continued 5 cm distally. The 8 cm incision that resulted was then lengthened if needed for a better exposure. Fascia lata was then split and the gluteus medius and vastus lateralis were devided. Antero-lateral capsulectomy was performed and the hip was dislocated. (PMID: 28439629)
Ogonda2005	Mini-lncision posterior approach	MIS-PA	In the standard-incision group the subcutaneous tissues and fascia lata were divided in line with the skin incision, but in the minimal-incision group only the proximal 1 cm of the fascia lata was incised. The distal fibers of the gluteus maximus were split by blunt dissection, and the short external rotators were detached close to their insertion into the greater trochanter. After reduction of the newly inserted prosthetic hip, the posterior capsule and short rotators were separately repaired with use of nonabsorbable sutures passed through drill-holes in the greater trochanter. Therefore, the only difference in surgical technique between the two groups was the length of the skin incision and the shorter incision of the fascia lata in the mini-incision group. (DOI: 10.2106/jbjs.d.02645)
Ogonda2005	Standard incision posterior approach	PA	

© 2023 Yan L et al. *JAMA Network Open.*

Study	Approach name in the article	Approach name after redefinition	Specific description of approach
Pagnano2009	2-incision	2-incision	For the two-incision technique, the surgical approach involved a 6-cm anterior incision and dissection through the Smith-Petersen interval to expose the hip, to cut the femoral neck, and to prepare the socket. A second incision of 3.8 to 5 cm was then made in the buttock, and the abductors and external rotators were identified and were protected with use of a cannula, through which the reamers were placed. The femur was then reamed, and the femoral component was placed through that posterior incision. (PMID: 18451391)
Pagnano2009	Mini-Posterior	MIS-PA	For the mini-posterior-incision technique, the surgical approach involved a 7 to 9.5-cm incision along the posterior aspect of the femur, starting at the tip of the greater trochanter and proceeding distally. The fascia of the gluteus maximus was split, and blunt dissection revealed the underlying abductor and external rotator musculature. The external rotators and the hip capsule were incised and preserved as one layer, with an attempt being made to preserve the insertion of the quadratus femoris on the femur. The hip was dislocated posteriorly, and the femoral neck was cut in accordance with the preoperative plan. (PMID: 18451391)
Parvizi2016	Direct Anterior Approach	DAA	The surgery was performed in supine position on a regular operating table that could be flexed at the hip for the DA patients. The initial incision length was 5 cm, and the incision was lengthened as dictated by the need for surgical exposure. The DA approach involved exposure of tensor fascia lata and division of its perimysium. The interval between sartorius and tensor fascia lata was not used in order to minimize the risk of injury to the lateral femoral cutaneous nerve. The lateral head or reflected portion of rectus was not incised but was retracted medially. Anterior capsulotomy was performed, preserving the capsule for later

© 2023 Yan L et al. *JAMA Network Open.*

Study	Approach name in the article	Approach name after redefinition	Specific description of approach
			closure, and the femoral neck was exposed. (PMID: 27241374)
Parvizi2016	direct lateral (DL)	DLA	The DL approach was performed by placement of the incision over the greater trochanter and division of the underlying fascia lata. The abductor mechanism was divided and the anterior one-half retracted anteriorly. Following capsulotomy, the hip was dislocated and the femoral neck was cut. Acetabular and femoral preparation was conducted in a conventional manner. (PMID: 27241374)
Pospischill2010	Traditional Transgluteal Approach	**DLA**	In the standard group, the patient was placed in the supine position with only the involved lower limb draped. A lateral skin incision, approximately 12 cm in length, was performed. With use of the transgluteal approach as described by Hardinge, the fascia lata was split longitudinally and retracted. The distal portion of the gluteus medius and the proximal portion ofthe vastus lateralis were split in the direction oftheir muscular fibers to expose the joint. The gluteus medius was split approximately 3 cm proximal to the upper tip of the greater trochanter. (PMID: 20124059)
Pospischill2010	minimally invasive modified Watson-Jones approach	**MIS-ALA**	In the minimally invasive group, the patient was positioned on the operating table in the supine position and both lower limbs were draped in a sterile fashion. An oblique skin incision measuring 8 to 10 cm was performed, extending distally from the anterior superior iliac spine and ending at the flare of the greater trochanter. After division of the subcutaneous tissue and fascia, the interval between the tensor fasciae latae and the gluteus medius was opened bluntly with the insertion of a finger. No muscle was split or detached with use of this technique. (PMID: 20124059)

© 2023 Yan L et al. *JAMA Network Open.*

Study	Approach name in the article	Approach name after redefinition	Specific description of approach
Reichert2018	direct anterior	DAA	The minimally invasive DAA as described by Rachbauer. (PMID: 30025519) (PMID: 16133154)
Reichert2018	transgluteal approach	**DLA**	The lateral transgluteal approach according to Bauer et al. (PMID: 30025519) (PMID: 526126)
Restrepo2010	modified Smith-Peterson approach	**DAA**	The direct anterior approach involved exposure of tensor fascia lata and division of its perimysium. The interval between sartorius and tensor fascia lata is reached by blunt dissection to minimize the risk of injury to lateral femoral cutaneous nerve. The lateral head or reflected portion of rectus was incised. Anterior capsulectomy was performed and the femoral neck was exposed. A wedge of bone from the femoral neck was removed to allow easy dislocation of the remaining head. The preparation of the acetabulum and the femoral neck was then carried out in routine manner. Exposure of the femoral canal involved selected soft tissue releases on the posterior aspect of the femoral neck. Modified instruments for reaming of the acetabulum and femoral canal were used for this procedure. (PMID: 20378307)
Restrepo2010	direct lateral approach	**MIS-DLA**	The direct lateral approach was performed using a modified Hardinge technique, similar as the technique described by Moskal and Mann, but with the patient in supine position, which included placement of the incision over the greater trochanter and division of the underlying fascia lata. The abductor mechanism was divided approximately in the anterior two thirds of the gluteus medius, the approach was extended into the anterior aspect of the vastus lateralis, and the anterior portion retracted anteriorly. A small portion of the tendon was left attached to the greater trochanter to facilitate reattachment at time of closure.

© 2023 Yan L et al. *JAMA Network Open.*

Study	Approach name in the article	Approach name after redefinition	Specific description of approach
			After capsulotomy, the hip was dislocated and the femoral neck was cut. (PMID: 20378307)
Rosenlund2017	lateral approach	**MIS-DLA**	LA was performed through a midline incision over the greater trochanter and involved detachment of the anterior one-third of the gluteus medius insertion and gluteus minimus insertion at the tip of the greater trochanter. The hip capsule was excised on the anterior side of the joint, from the basis of the collum femoris to the acetabular rim. The hip was dislocated by external rotation, adduction and fl exion. During closure of the wound, the detached parts of the gluteus medius and minimus were re-inserted using a heavy absorbable suture (coated VICRYL, size 2) to re-approximate the divided gluteus minimus and the anterior flap of gluteus medius. No capsular repair was performed. A detailed description of the approach can be found in the work by Mulliken et al. (PMID: 28464754)
Rosenlund2017	posterior approach	PA	PA was performed through an incision over the posterior part of the greater trochanter through the fascia, followed by blunt dissection of gluteus maximus. The external rotators were detached and the hip capsule incised (Hoppenfeld et al. 2009). The hip was dislocated by internal rotation and flexion. During closure of the wound, the capsule was repaired and the external rotators were re-inserted using a heavy absorbable suture (coated VICRYL, size 2). (PMID: 28464754)
Roy2010	standard posterior approach	PA	The standard technique aimed for an incision equal to or greater than 16 cm. The quadratus was released in the standard technique, and part of the gluteus maximus insertion was released as needed. (PMID: 19883910)

© 2023 Yan L et al. *JAMA Network Open.*

Study	Approach name in the article	Approach name after redefinition	Specific description of approach
Roy2010	posterior mini-incision approach	MIS-PA	The MIS approach aimed for an 8 cm incision centred over the posterior aspect ofthe greater trochanter. The short external rotators and capsule were taken as a unit and tagged for later repair. The quadratus femoris, piriformis tendon and anterior capsule were spared, as well as the femoral insertion of the gluteus maximus. (PMID: 19883910)
Rykov2021	Direct Anterior Approach	DAA	For the DAA, the patient is placed in a supine decubitus position. The skin incision is made over and in the direction of the lateral part of the femoral head and neck. After division of skin and subcutis, the interval between the tensor fasciae latae muscle and the sartorius muscle is identified and the overlying fascia is opened. In this part of the operation, care was taken to avoid damaging the lateral femoral cutaneous nerve. The intermuscular plane between the tensor fasciae latae and sartorius muscles is developed further down to the hip capsule. Subsequently, the hip capsule is opened, allowing access to the hip joint. (PMID: 19169792)
Rykov2021	Posterolateral approach	**PA**	For the PLA, the patient is placed in a lateral decubitus position. The skin incision is made over the greater trochanter to cranial, with a slight curve to posterior. After transection of the subcutis, the fascia latae and the gluteus maximus muscles are split. Next, the short external rotators, namely the piriformis, the inferior and superior gemellus, and the internal obturator muscles, are cut at the level of their insertion at the greater trochanter, so this approach is not muscle-sparing. After retraction of the short external rotators backward, the hip capsule can be incised, allowing access to the hip joint. Subsequently, the hip joint is dislocated and the osteotomy of the femoral neck is performed, followed by the removing of the

© 2023 Yan L et al. *JAMA Network Open.*

Study	Approach name in the article	Approach name after redefinition	Specific description of approach
			femoral head. (PMID: 34116911)
Schwarze2018	transgluteal lateral Harding	**DLA**	The conventional(CON) approach was a transgluteal lateral Harding. (PMID: 29287171)
Schwarze2018	anterolateral modified Watson-Jones	**MIS-ALA**	The minimally invasive (MIS) approach was an anterolateral modified Watson-Jones (Bertin and Röttinger 2004) performed without incision and detachment of the gluteus medius muscle. (PMID: 29287171) (PMID: 15577495)
Serson2017	mini-posterior approach	MIS-PA	The mini-posterior approach was performed with the patient in the lateral decubitus position through a 7 to 10 cm incision made over the posterior border of the greater trochanter. The gluteus maximus muscle was split in line with its fibers,and the short external rotators were released, including the piriformis but preserving the quadratus femoris. Posterior capsule and short external rotators were tagged and repaired at the end of the procedure. (PMID: 28434694)
Serson2017	2-incision	2-incision	The two-incision technique was performed as described by Berger. (PMID: 14646722)
Shitama2009	Standard-incision Translateral approach	**DLA**	The patients were randomly allocated to have surgery through either a minimally invasive incision of <10 cm or a standard incision of 15 cm.<br>In all patients, the same surgeon performed the surgery, employing either the translateral (PMID: 8403638)or posterolateral approach with a capsular repair. (PMID: 12728429)
Shitama2009	Standard-incision Posterolateral approach	**PA**	

© 2023 Yan L et al. *JAMA Network Open.*

Study	Approach name in the article	Approach name after redefinition	Specific description of approach
Shitama2009	Mini-incision Translateral approach	**MIS-DLA**	
Shitama2009	Mini-incision Posterolateral approach	**MIS-PA**	
Speranza2007	traditional lateral approach	DLA	In group A the skin incision was no more than 8 cm, in group B the skin incision was standard (12-14 cm). In all cases a total hip replacement with use of a cementless cup and a cementless stem and direct lateral approach was used. (PMID: 15586328)
Speranza2007	lateral minimal-incision	MIS-DLA	
Takada2018	direct anterior approach (DAA)	DAA	Initial incisions were made on the anterolateral aspect on both sides of the hips and slightly more anterior for the DAA sides in some cases. The incision was 8-10 cm long and extended at the surgeon's discretion in some cases. After the incision, in the side subjected to DAA (DAA side), the interval between the sartorius and TFL was reached by blunt dissection to minimize the risk of LFCN injury. The ascending branches of the lateral femoral circumflex artery were carefully coagulated to expose the anterior aspect of the capsule. (PMID: 29935972)
Takada2018	anterolateral approach (ALA)	**MIS-ALA**	In the side subjected to ALA (ALA side), the interval between the TFL and gluteus medius was also bluntly developed without muscle cutting or detachment, and the exposed plane was not developed too proximally to minimize the risk of SGN injury. The same procedure was performed after the exposure of the anterior aspect of the capsule in both approaches. (PMID: 29935972)

© 2023 Yan L et al. *JAMA Network Open.*

Study	Approach name in the article	Approach name after redefinition	Specific description of approach
Tan2018	standard posterior approach	PA	The piriformis-sparing approach has been described in detail. In summary, it involves a short (approximately 7 cm to 10 cm) oblique incision over the posterior aspect of the greater trochanter. The capsule is incised along the inferior border of piriformis from the edge of the acetabulum to the posterior border of the femur, continuing distally in an 'L' shape, detaching the capsule, gemelli and obturator internus as one. Quadratus femoris may be partially detached, as required, to visualise the inferior part of the neck. The hip is then dislocated and osteotomy of the neck performed in the normal manner. After insertion of the component a combined capsulotendinous repair is performed through two drill holes in the bone. Drill holes are placed from lateral to medial at the posterior aspect of the greater trochanter, resulting in an anatomical repair to their correct point of attachment. Apart from the longer incision (approximately 20 cm) and the division and repair of piriformis using the standard posterior approach, there is no other difference between the two approaches. (PMID: 22219246)
Tan2018	piriformis-sparing approach	**MIS-PA**	
Taunton2014	direct anterior	DAA	The patient is positioned in a supine position on an orthopedic table. An oblique incision is made over the anterior margin of the tensor muscle at a point approximately 2 centimeters lateral from the anterior superior iliac spine and extending 10 centimeters. The interval of the tensor fascia lata and sartorius is developed. A measured resection of the femoral neck is performed. (PMID: 25007723)
Taunton2014	mini-posterior approach	MIS-PA	The patient is positioned in the lateral decubitus position. A 10 cm incision is placed over the greater trochanter, slightly curved posteriorly. An incision is placed just superior to the piriformis tendon through the hip capsule. The hip

© 2023 Yan L et al. *JAMA Network Open.*

Study	Approach name in the article	Approach name after redefinition	Specific description of approach
			capsule is retracted posteriorly and is detached with the short external rotators from the posterior aspect of the greater trochanter extending down to and often including the quadratus muscle. Following dislocation and measured neck resection, the acetabulum and femur are prepared. Following implantation of the arthroplasty, the capsular closure includes suture re-approximation of the superior capsule to the posterior aspect of the greater trochanter. (PMID: 25007723)
Taunton2018	DAA	DAA	For the DAA technique, a specialized table with fluoroscopy was utilized. The specific technique is as described by Taunton et al. with capsulotomy and repair. (PMID: 25007723)
Taunton2018	miniposterior approach (MPA)	MIS-PA	For the MPA technique, the hip capsule and external rotators were incised as one layer and repaired formally at conclusion of THA as described by Pagnano et al. (PMID: 25007723)
Thaler2018	Direct anterior approach	DAA	All surgeries were performed in a supine position. The direct anterior approach was performed according to the publication by Krismer. (PMID: 30015203)
Thaler2018	traditional antero-lateral approach	**DLA**	The anterolateral approach was performed as described by Bauer. (PMID: 526126)
Ulivi2021	Direct superior approach	**MIS-PA**	In the DSA group, no iliotibial band section was performed; however, splitting of the gluteus maximus muscle, short external rotator preservation with selective division of the piriformis tendon and a posterior capsulotomy were performed. (PMID: 33410360)
Ulivi2021	posterolateral approach	**PA**	In the PL group a mini standard approach was used. The total incision of the fascia lata accounted for approximately 3-5 cm proximally and distally to the tip of the

© 2023 Yan L et al. *JAMA Network Open.*

Study	Approach name in the article	Approach name after redefinition	Specific description of approach
			greater trochanter. (PMID: 33410360)
Varela2013	conventional lateral approach	DLA	In the other group, the patients were operated through a classic lateral approach (control group). (PMID: 23412407)
Varela2013	minimally invasive lateral approach	MIS-DLA	Minimally invasive procedures were performed using the modified lateral technique described by Howell et al. (PMID: 15062696)
Vasilakis2012	conventional anterolateral approach	**DLA**	In group B, the patient was placed in the supine position with only the involved lower limb draped. A lateral skin incision approximately 14 to 16 cm in length was made, extending distally from the anterior superior iliac spine and ending at the flare of the greater trochanter. Using the modified Watson-Jones anterolateral approach, the fascia latae was split longitudinally and retracted. The distal half of the gluteus medius insertion at the greater trochanter was partially released to allow adduction for better orientation and hip dislocation. The hip capsule was subtotally resected. For preparation of the proximal part of the femur, the involved lower limb was positioned in external rotation over the contralateral lower limb. (PMID: 23218622)
Vasilakis2012	minimally invasive anterolateral approach	MIS-ALA	In group A, the patient was positioned on the operating table in the supine position with only the involved lower limb draped in a sterile fashion. An oblique skin incision measuring 8 to 10 cm was made, extending distally from the anterior superior iliac spine and ending at the flare of the greater trochanter. After division of the subcutaneous tissue and fascia, the interval between the tensor fasciae latae

© 2023 Yan L et al. *JAMA Network Open.*

Study	Approach name in the article	Approach name after redefinition	Specific description of approach
			and the gluteus medius was opened bluntly with the insertion of a finger. No muscle was split or detached. The hip capsule was divided in an H-shaped fashion and preserved. (PMID: 23218622)
Wang2019	MIS posterior approach	MIS-PA	For the MIS posterior approach, the minimally invasive form of that popularized by Gibson was utilized: the tendon insertion of the short external rotators including piriformis, internal obturator muscle, superior gemellus, and inferior gemellus were cut off; the posterior joint capsule was cut through with a flap-shaped incision. Only the tendon of piriformis in combination with the posterior joint capsule was non in-situ reattached through a suture hole on the posterior part of femoral great trochanter using the non-absorbable suture. (PMID: 31053885)
Wang2019	the modified direct lateral (mDL) approach	**MIS-DLA**	The mDL approach was amodification ofthe approach which was initially described by Hardinge with the detachment and subsequent in situ repair of the anterior forth to third of the tendons of GMED and gluteus minimus. Notably, the GMED was incised to a maximum length of 3 cm to protect the superior gluteal nerve (SGN) and the incision prolonging into the vastus lateralis was strictly avoided. (PMID: 31053885)
Witzleb2009	posterior approach	PA	The posterior approach entailed a curved incision centered on the greater trochanter in lateral decubitus position of the patient. The fascia lata was incised in line of the skin incision and the fibers of the gluteus maximus were split by blunt dissection. The short external rotators were then detached close to their femoral insertion leaving one centimeter of muscle tissue of the quadratus femoris at the dorsal aspect of the greater trochanter for re-attachment. The posterior hip capsule was incised and preserved. After implantation, the posterior capsule was

© 2023 Yan L et al. *JAMA Network Open.*

Study	Approach name in the article	Approach name after redefinition	Specific description of approach
			re-attached on the greater trochanter together with the short external rotators and the wound was closed in layers. (PMID: 19541586)
Witzleb2009	direct lateral approach	DLA	The direct lateral approach entailed a longitudinal skin incision centered over the greater trochanter in supine position. The tractus iliotibialis and the gluteal fascia were divided in the line of the skin incision. The anterior part of the gluteus medius and minimus insertion was incised down to the bone, prolonged distally through the vastus lateralis in a curved line to spare some tendinous tissue at the greater trochanter for reattachment. The anterior hip capsule was excised. After implantation, the tendinous tissue was re-attached at the greater trochanter and the wound was closed in layers. (PMID: 19541586)
Xie2017	Posterior approach technique (Moore approach)	PA	The patient was placed in a lateral position; the incision was started 10 cm distal to the posterior superior iliac spine and extended to the posterior margin of the greater trochanter. The length of the incision was 12–13 cm; exposure and division of the deep fascia was made in line with the skin incision. The fibers of the gluteus maximus were dissected bluntly and separated, and exposed the greater trochanter. Divisions of the distal fibers were exposed, and the external rotators were released. The muscles were retracted medially, and the capsule was exposed and split distally to the proximal along the line of the femoral neck in order to detach the distal part of the capsule from the femur the rim of the acetabulum. (PMID: 28946892)
Xie2017	supercapsular percutaneously	SuperPath	The patient was positioned in the lateral position with the hip in 45 degrees of flexion and 10-15 degrees of internal rotation. A 6-8 cm incision superior to the greater trochanter was made. The gluteal fascia was incised, and the gluteus

© 2023 Yan L et al. *JAMA Network Open.*

Study	Approach name in the article	Approach name after redefinition	Specific description of approach
	assisted approach		maximus was separated in line with fibers. The interval between the gluteus minimus and piriformis was exposed by using a Zelpi retractor. One blunt Hohmann retractor was placed anteriorly under the gluteus medius to protect the muscle, and the leg was elevated to reduce the tension on the external rotators making it easier to place another Hohmann retractor beneath the piriformis to protect the sciatic nerve. A Cobb elevator was used to push the posterior part of the gluteus minimus muscle anteriorly and expose the hip joint capsule. The hip joint capsule was then cut according to the incision from the base of the greater trochanter to 1 cm proximal to the acetabular rim. The capsule was elevated as a flap anterior and posterior to improve visualization, and the blunt Hohmann retractor was then moved to the intracapsular position. Starting in the anterior portion of the piriformis fossa, the femur was reamed and broached without dislocation. Occasionally, in osteoarthritis patients, huge osteophytes need to be removed by osteotome to expose the starting point. (PMID: 28946892)
Yang2009	postlateral approach	**PA**	Patients were randomly selected for surgery with standard posterolateral indision. (PMID: 19847593)
Yang2009	OCM approach	**MIS-ALA**	The skin incision was made on a line beginning at the anterior tubercle of the greater trochanter and extending along the femoral axis approximately 7 cm in length. One fourth of the incision was over the trochanter and the rest was proximal. Tensor fasciae latae muscle and gluteus medius were revealed in the gap, followed by the anterior capsule. When a Z-shaped capsulotomy was made, the retractors on the inferior and superior aspects of the neck were moved from an extracapsular to an intracapsular position to expose the femoral neck. After

© 2023 Yan L et al. *JAMA Network Open.*

Study	Approach name in the article	Approach name after redefinition	Specific description of approach
			using two separate osteotomies with the hip externally rotated, the femoral head and neck were removed. (PMID: 19847593)
Zhao2017	direct anterior	DAA	The DAA, which is a modification of the traditional Smith-Petersen approach, was performed using the interval between the tensor fascia latae and the sartorius muscle. (PMID: 28662957)
Zhao2017	posterolateral approach	**PA**	The PLA, a modification of the Gibson-Moore approach involving enhanced capsular closure, was performed with the patient in a lateral decubitus position on a standard operating table. After skin incision through the fascia over the greater trochanter, the gluteus maximus was split, the external rotators were detached, and an incision was made in the hip capsule. The hip was dislocated by internal rotation and flexion. The femoral neck was resected based on preoperative templating. (PMID: 28662957)
Zomar2018	direct anterior	DAA	78 participants were prospectively enrolled to undergo a THA through either a DA or direct lateral (DL) surgical approach (PMID: 17162165, 17514173).
	direct lateral	**MIS-DLA**	

© 2023 Yan L et al. *JAMA Network Open.*

eTable 5. Risk of Bias Results and Judgment Basis for Each Article

Abdel2017

**Bias**	**Authors' judgement**	**Support for judgement**
Random sequence generation (selection bias)	Unclear risk	Not reported
Allocation concealment (selection bias)	Low risk	All patients enrolled in the study received the treatment as randomized.
Blinding of participants and personnel (performance bias)	Low risk	The primary surgeon (MWP) was blinded before the surgical procedure, but not intraoperatively
Blinding of outcome assessment (detection bias)	Low risk	Radiographs were analyzed by 2 authors (MPA and BPC) not involved in the surgical interventions.
Incomplete outcome data (attrition bias)	Low risk	One male patient in the miniposterior cohort was lost to follow-up, leaving 71 patients available for the most recent follow-up.
Selective reporting (reporting bias)	Unclear risk	Not reported
Other bias	Low risk	One or more of the authors of this paper have disclosed potential or pertinent conflicts of interest, which may include receipt of payment, either direct or indirect, institutional support, or association with an entity in the biomedical field which may be perceived to have potential conflict of interest with this work. For full disclosure statements refer to http://dx.doi.org/10.1016/j.arth.2017.04.005.

© 2023 Yan L et al. *JAMA Network Open.*

Barrett 2019

Bias	Authors' judgement	Support for judgement
Random sequence generation (selection bias)	Low risk	Patients were randomized to 1 of 2 study groups, DAA or PA, using a randomized block scheduling method.
Allocation concealment (selection bias)	Low risk	Patients were randomized to 1 of 2 study groups, DAA or PA, using a randomized block scheduling method.
Blinding of participants and personnel (performance bias)	Unclear risk	Not reported
Blinding of outcome assessment (detection bias)	Low risk	All radiographs were examined by an independent reviewer (TBA) whowas not involved with patient care and thuswas blinded from all clinical information during the assessment of radiographic implants.
Incomplete outcome data (attrition bias)	Low risk	There were 2 deaths, one from each surgical approach group.Neither death was related to the implant or procedure. The patient in the DAA group died 6.6 years after surgery, while the PA patient passed away 4.1 years after surgical implant. Four patientswere lost to follow-up after the 1-year follow-up study, 2 from each surgical approach group.
Selective reporting (reporting bias)	Unclear risk	Not reported
Other bias	Low risk	One or more of the authors of this paper have disclosed potential or pertinent conflicts of interest, which may include receipt of payment, either direct or indirect, institutional support, or association with an entity in the biomedical field which may be perceived to have potential conflict of interest with this work. For full disclosure statements refer to https://doi.org/10.1016/j.arth.2019.01.060.

© 2023 Yan L et al. *JAMA Network Open.*

Bon2019

Bias	Authors' judgement	Support for judgement
Random sequence generation (selection bias)	Low risk	Patients were randomized to the AA and PA groups using the Randomizer for Clinical Trial software (Medsharing, Fontenaysous-Bois, France).
Allocation concealment (selection bias)	Unclear risk	Not reported
Blinding of participants and personnel (performance bias)	Unclear risk	Not reported
Blinding of outcome assessment (detection bias)	Unclear risk	Not reported
Incomplete outcome data (attrition bias)	Low risk	The flow chart shows the specific number and time of lost visits in each period.
Selective reporting (reporting bias)	Low risk	The trial was registered under RCB ID no 2017-A02875-48 after review board approval (registered with the Clinical Research and Innovation Delegation under no PI2017-843-0023).
Other bias	Low risk	The authors declare that they have no competing interest. Elsewhere, P Mertl receives fees from Stryker, DePuy, Zimmer, X-Nov and Adler and A Gabrion from X-Nov.

© 2023 Yan L et al. *JAMA Network Open.*

Brismar2018

Bias	Authors' judgement	Support for judgement
Random sequence generation (selection bias)	Low risk	100 consecutive patients were randomly allocated by a computer program to either surgery through a direct anterior (DA) single incision approach (Rachbauer 2005) or a direct lateral (DL) transgluteal approach (Duparc et al. 1997)
Allocation concealment (selection bias)	Low risk	Each patient was given a consecutively numbered sealed envelope containing an allocation paper put into the envelope by an independent research manager not further involved in the study.
Blinding of participants and personnel (performance bias)	Low risk	The surgeon and patient were blinded prior to the opening of the envelope.
Blinding of outcome assessment (detection bias)	Unclear risk	Not reported
Incomplete outcome data (attrition bias)	Low risk	There was no loss of follow-up.
Selective reporting (reporting bias)	Low risk	The study was registered by the Chinese Clinical Trial Registry (ChiCTR1900020770, 19 January 2019).
Other bias	Low risk	Stryker unconditionally sponsored the study. The authors declare no conflicts of interest.

© 2023 Yan L et al. *JAMA Network Open.*

Cao2020

Bias	Authors' judgement	Support for judgement
Random sequence generation (selection bias)	Unclear risk	Not reported
Allocation concealment (selection bias)	Low risk	Patients who met the inclusion criteria were assigned to the DAA or PLA group by choosing closed envelopes which contained random numbers.
Blinding of participants and personnel (performance bias)	Low risk	Due to obvious difference in surgical incisions, we did not use blind methods.
Blinding of outcome assessment (detection bias)	Low risk	Due to obvious difference in surgical incisions, we did not use blind methods.
Incomplete outcome data (attrition bias)	Low risk	The flow chart shows the specific number and time of lost visits in each period.
Selective reporting (reporting bias)	Low risk	The study was registered by the Chinese Clinical Trial Registry (ChiCTR1900020770, 19 January 2019).
Other bias	Unclear risk	Competing interests The authors declare that they have no competing interests.This research did not receive any specific grant from funding agencies in the public, commercial or not-for-profit sectors.

© 2023 Yan L et al. *JAMA Network Open.*

Catma 2017

Bias	Authors' judgement	Support for judgement
Random sequence generation (selection bias)	High risk	patients were randomized into two groups with regard to the admittance order to the orthopaedic outpatient clinic.
Allocation concealment (selection bias)	Unclear risk	Not reported
Blinding of participants and personnel (performance bias)	Unclear risk	Not reported
Blinding of outcome assessment (detection bias)	Unclear risk	Not reported
Incomplete outcome data (attrition bias)	Low risk	Seven patients lost to follow up and were excluded from the study.
Selective reporting (reporting bias)	Unclear risk	Not reported
Other bias	Low risk	The author(s) received no financial support for the research, authorship and/or publication of this article.

© 2023 Yan L et al. *JAMA Network Open.*

Cheng 2017

Bias	Authors' judgement	Support for judgement
Random sequence generation (selection bias)	Unclear risk	Not reported
Allocation concealment (selection bias)	Low risk	Participants were then stratified by age (<65 or ≥65 years), surgeon (1 or 2) and randomly allocated to either DAA or PA groups within strata using a concealed method.
Blinding of participants and personnel (performance bias)	Low risk	Surgeons and the primary investigator were blinded to the approach until the pre-operative planning meeting while participants were blinded pre-operatively.
Blinding of outcome assessment (detection bias)	Low risk	An independent researcher not involved in participant recruitment, treatment or assessment prepared the randomization sequence with allocation prepared in sequentially numbered opaque envelopes.
Incomplete outcome data (attrition bias)	Low risk	There were no lost cases
Selective reporting (reporting bias)	Low risk	The trial was prospectively registered in the Australian New Zealand Clinical Trials Registry ACTRN12614000131651
Other bias	High risk	The authors of the paper acknowledge the supervision and support of Professor Ian Davis and the Monash University Eastern Health Clinical School. We also acknowledge the generous donations from the Bulley Fellowship and Box Hill Golf Club for this research. Lastly, this body of research is dedicated to the work and memory of Mr Michael Armstrong.

© 2023 Yan L et al. *JAMA Network Open.*

Chimento 2005

Bias	Authors' judgement	Support for judgement
Random sequence generation (selection bias)	Unclear risk	Not reported
Allocation concealment (selection bias)	Unclear risk	Not reported
Blinding of participants and personnel (performance bias)	Unclear risk	Not reported
Blinding of outcome assessment (detection bias)	Low risk	The radiographs were evaluated by an orthopedic research fellow (VP) who was blinded as to the incision length.
Incomplete outcome data (attrition bias)	Low risk	In group A, 27 hybrid procedures (noncemented acetabulum and cemented femoral component) and 1 uncemented THA were performed. In group B, there were 28 hybrid procedures and 4 uncemented arthroplasties.
Selective reporting (reporting bias)	Unclear risk	Not reported
Other bias	Low risk	No benefits or funds were received in support of the study.

© 2023 Yan L et al. *JAMA Network Open.*

Christensen 2015

Bias	Authors' judgement	Support for judgement
Random sequence generation (selection bias)	Unclear risk	Not reported
Allocation concealment (selection bias)	Unclear risk	Not reported
Blinding of participants and personnel (performance bias)	Unclear risk	Not reported
Blinding of outcome assessment (detection bias)	Unclear risk	Not reported
Incomplete outcome data (attrition bias)	Low risk	The flow chart shows the specific number and time of lost visits in each period.
Selective reporting (reporting bias)	Low risk	(ClinicalTrials.gov Identifier NCT01807494),
Other bias	Low risk	One ormore ofthe authors ofthis paper have disclosedpotential or pertinent conflicts of interest, which may include receipt of payment, either direct or indirect, institutional support, or association with an entity in the biomedical field which may be perceived to have potential conflict of interest with this work. For full disclosure statements refer to http://dx.doi.org/10.1016/j.arth.2014.12.03

© 2023 Yan L et al. *JAMA Network Open.*

D'Arrigo2009

Bias	Authors' judgement	Support for judgement
Random sequence generation (selection bias)	Unclear risk	Not reported
Allocation concealment (selection bias)	Unclear risk	Not reported
Blinding of participants and personnel (performance bias)	Unclear risk	Not reported
Blinding of outcome assessment (detection bias)	Unclear risk	Demographic information, laboratory values and the postoperative course including post-operative complications were determined from a review of office charts.The radiographs were evaluated by an orthopaedic research fellow (A.S.) who was blinded as to the group of patients.
Incomplete outcome data (attrition bias)	Low risk	There was no loss of follow-up.
Selective reporting (reporting bias)	Low risk	All of our patients were admitted the day before surgery, which is current practice in our hospital, and the length of hospital stay was calculated from the day of surgery to the day of discharge.
Other bias	Low risk	Conflict of interest statement The authors declare that they have no conflict of interest related to the publication of this manuscript.

© 2023 Yan L et al. *JAMA Network Open.*

De Anta-Diza2016

Bias	Authors' judgement	Support for judgement
Random sequence generation (selection bias)	Low risk	Randomization to lateral or anterior group was based on a list of random numbers.
Allocation concealment (selection bias)	Unclear risk	Not reported
Blinding of participants and personnel (performance bias)	Unclear risk	Not reported
Blinding of outcome assessment (detection bias)	Low risk	All clinical data were collected by independent observers who had not participated in the surgery.
Incomplete outcome data (attrition bias)	Low risk	There was no loss to follow-up or discontinued study protocol.
Selective reporting (reporting bias)	Unclear risk	Not reported
Other bias	Low risk	Conflict of interest The authors declare have no conflict of interest.

© 2023 Yan L et al. *JAMA Network Open.*

Della2010

Bias	Authors' judgement	Support for judgement
Random sequence generation (selection bias)	Low risk	The envelopes were created from a computer-generated randomization list.
Allocation concealment (selection bias)	Low risk	Patients were randomized using opaque, sealed, numbered envelopes after the successful induction of neuraxial anesthesia.
Blinding of participants and personnel (performance bias)	Low risk	the patient was not blinded to the procedure performed
Blinding of outcome assessment (detection bias)	Low risk	all postoperative assessments were performed by a clinical nurse (ED) without knowledge of the surgical approach used. Patients were instructed not to share with the nurse the number of incisions used and they wore shorts during all clinical assessments.
Incomplete outcome data (attrition bias)	Low risk	All patients were followed and assessed at 1 year postoperatively with no patients lost to followup.
Selective reporting (reporting bias)	Low risk	this study also was registered with clinicaltrials.gov (NCT00594893)
Other bias	High risk	This study was supported by a grant from Zimmer Inc (Warsaw, IN). Dr. Della Valle has performed consulting services for Zimmer within the past year and presently is a consultant for Smith and Nephew Inc (Memphis, TN), Biomet Inc (Warsaw, IN), and Kinamed Inc (Camarillo, CA).

© 2023 Yan L et al. *JAMA Network Open.*

Dienstknecht2014

Bias	Authors' judgement	Support for judgement
Random sequence generation (selection bias)	Unclear risk	were randomised to undergo unilateral THA through a mini-incision approach (Micro-hip,13 transgluteal approach (Bauer,14 n=55) or a standard, lateral, n=88) by a dedicated team with extensive experience.
Allocation concealment (selection bias)	Unclear risk	Not reported
Blinding of participants and personnel (performance bias)	Unclear risk	Not reported
Blinding of outcome assessment (detection bias)	Unclear risk	Not reported
Incomplete outcome data (attrition bias)	Low risk	All patients were followed up for at least 3 months. No patient was lost to follow-up.
Selective reporting (reporting bias)	Unclear risk	Not reported
Other bias	Low risk	No conflicts of interest were declared by the authors.

© 2023 Yan L et al. *JAMA Network Open.*

Dorr2007

Bias	Authors' judgement	Support for judgement
Random sequence generation (selection bias)	Unclear risk	Not reported
Allocation concealment (selection bias)	Unclear risk	Not reported
Blinding of participants and personnel (performance bias)	Unclear risk	Not reported
Blinding of outcome assessment (detection bias)	Low risk	All data were collected prospectively each day during the hospitalization by individuals who were blinded to the operative technique used for that patient. The data were analyzed by a research team that was not directly involved with the patient care.
Incomplete outcome data (attrition bias)	Low risk	There was no loss of follow-up.
Selective reporting (reporting bias)	Low risk	There were no lost cases
Other bias	High risk	Disclosure: In support of their research for or preparation of this work, one or more of the authors received, in any one year, outside funding or grants in excess of $10,000 from Zimmer.

© 2023 Yan L et al. *JAMA Network Open.*

Dutka 2007

Bias	Authors' judgement	Support for judgement
Random sequence generation (selection bias)	High risk	Patients were randomized into both groups on the basis of the date that the operation was carried out (odd days – study group, even days – control group).
Allocation concealment (selection bias)	Unclear risk	Not reported
Blinding of participants and personnel (performance bias)	Unclear risk	Not reported
Blinding of outcome assessment (detection bias)	Unclear risk	The evaluation of outcomes was carried out by a physician having no knowledge of the surgical approach used in individual patients.
Incomplete outcome data (attrition bias)	low risk	There was no lost of follow-up.
Selective reporting (reporting bias)	Unclear risk	Not reported
Other bias	Unclear risk	Not reported

© 2023 Yan L et al. *JAMA Network Open.*

Gossen2011

Bias	Authors' judgement	Support for judgement
Random sequence generation (selection bias)	Unclear risk	Not reported
Allocation concealment (selection bias)	Low risk	After obtaining informed consent, patients were allocated (envelopes) to one of the two operations (MIS or CLASS) based on a stratified randomization scheme of two groups of surgeons, ie, an anterior approach group and a posterior approach group.
Blinding of participants and personnel (performance bias)	Low risk	Withdrawal of a patient for any reason or any operation (including revision) leading to a new incision of the wound area resulted in premature unblinding of the patient and exclusion from the study.
Blinding of outcome assessment (detection bias)	Low risk	All data were collected at baseline and prospectively (during hospital stay and 1-year followup) by an investigator (BMK) who had not been involved in the patients' care or surgery and was blinded to group allocation. The data were analyzed by two research members (JHG, BJK) who were not involved in the clinical procedures.
Incomplete outcome data (attrition bias)	Low risk	Missing value analysis shows 3.3% of the primary end point data, HHS at 6 weeks, and 9.2% at 1 year were missing.<br>There are corresponding records and explanations for the lost people
Selective reporting (reporting bias)	Unclear risk	Not reported
Other bias	Low risk	Each author certifies that he or she has no commercial associations (eg, consultancies, stock ownership, equity interest, patent/licensing arrangements, etc) that might pose a conflict of interest in connection with the submitted article.

© 2023 Yan L et al. *JAMA Network Open.*

Hu 2012

Bias	Authors' judgement	Support for judgement
Random sequence generation (selection bias)	Unclear risk	They were equally randomized into the two-incision first or modified Watson-Jones first group and followed regularly.The patients were scheduled for surgery on the side with more discomfort first and the choice of surgical approach was randomly assigned.
Allocation concealment (selection bias)	Unclear risk	Not reported
Blinding of participants and personnel (performance bias)	Unclear risk	Not reported
Blinding of outcome assessment (detection bias)	Unclear risk	Not reported
Incomplete outcome data (attrition bias)	Low risk	No loss of follow-up
Selective reporting (reporting bias)	Unclear risk	Not reported
Other bias	Unclear risk	Not reported

© 2023 Yan L et al. *JAMA Network Open.*

Inaba2011

Bias	Authors' judgement	Support for judgement
Random sequence generation (selection bias)	Unclear risk	Patients were randomly assigned to the muscle-sparing group (n = 57) or the mini-incision direct lateral approach group (n = 60).
Allocation concealment (selection bias)	Unclear risk	Not reported
Blinding of participants and personnel (performance bias)	Unclear risk	Not reported
Blinding of outcome assessment (detection bias)	Low risk	Two observers (Y.Y. and T.I.) blinded to patient information measured these angles.
Incomplete outcome data (attrition bias)	Low risk	During the follow-up period, 15 patients (7 of the muscle-sparing group, 8 of the mini-incision direct lateral approach group) were lost to follow-up.
Selective reporting (reporting bias)	Unclear risk	Not reported
Other bias	Low risk	No support from equipment manufacturers.

© 2023 Yan L et al. *JAMA Network Open.*

Ji2012

Bias	Authors' judgement	Support for judgement
Random sequence generation (selection bias)	Low risk	The remaining 205 hips in 205 patients were randomly chosen for a posterior approach or a modified lateral approach using a computer-generated random table.
Allocation concealment (selection bias)	Unclear risk	Not reported
Blinding of participants and personnel (performance bias)	Unclear risk	Not reported
Blinding of outcome assessment (detection bias)	Unclear risk	Not reported
Incomplete outcome data (attrition bias)	Low risk	Patients who had not returned for regularly scheduled visits were contacted by telephone. Two nurses and one private locator found and visited nonresponders.
Selective reporting (reporting bias)	Low risk	The study was registered in the Clinical Trials.gov Protocol Registration System (trial no. NCT00936949)
Other bias	Low risk	No support from equipment manufacturers

© 2023 Yan L et al. *JAMA Network Open.*

Khan 2012

**Bias**	**Authors' judgement**	**Support for judgement**
Random sequence generation (selection bias)	Low risk	Random allocation was computer generated
Allocation concealment (selection bias)	Low risk	Random allocation was computer generated using sealed identical opaque envelopes opened in theatre immediately pre-operatively
Blinding of participants and personnel (performance bias)	Low risk	patients were blinded until the two-week assessment
Blinding of outcome assessment (detection bias)	Low risk	Observers were blinded to the allocation throughout
Incomplete outcome data (attrition bias)	Low risk	Four patients in the piriformis-sparing group were lost to follow-up and seven in the standard group
Selective reporting (reporting bias)	Low risk	the trial was registered with the Australian New Zealand Clinical Trials Registry
Other bias	Unclear risk	The study was partly funded by a research grant from Smith & Nephew (Memphis, Tennessee).

© 2023 Yan L et al. *JAMA Network Open.*

Kim 2006

Bias	Authors' judgement	Support for judgement
Random sequence generation (selection bias)	low risk	Randomization of the use of either a minimally invasive technique or a standard technique was determined from a sequential pool on a table of randomized numbers.
Allocation concealment (selection bias)	Unclear risk	Not reported
Blinding of participants and personnel (performance bias)	Unclear risk	Not reported
Blinding of outcome assessment (detection bias)	low risk	A clinical hip fellow, who was blinded to thelength of the incision, analyzed a postoperative anteroposterior and lateral radiographs of both hips for each patient.
Incomplete outcome data (attrition bias)	Low risk	No lost of follow-up.
Selective reporting (reporting bias)	Unclear risk	Not reported
Other bias	low risk	No benefits or funds were received in support of the study.

© 2023 Yan L et al. *JAMA Network Open.*

Korytkin2021

Bias	Authors' judgement	Support for judgement
Random sequence generation (selection bias)	Low risk	Patients who met the inclusions criteria were randomly assigned to either the MPA or SuperPATH group according to a computed randomisation list,
Allocation concealment (selection bias)	Low risk	with numbered and sealed envelopes opened before the operation.
Blinding of participants and personnel (performance bias)	Unclear risk	Not reported
Blinding of outcome assessment (detection bias)	Low risk	2 unbiased biostatisticians, blinded to patient attribution and outcome, performed the statistical work.
Incomplete outcome data (attrition bias)	Low risk	s: 22 in the SuperPATH group and 27 in the MPA group.<br>Within the SuperPATH group, 2 patients were lost to follow-up. Thus 20 patients were available for analysis. In the MPA group, 3 patients were not available: 2 patients chose not to participate, 1 patient was still using a walking aid at 6 weeks follow-up
Selective reporting (reporting bias)	Unclear risk	Not reported
Other bias	High risk	The author(s) disclosed receipt of the following financial support for the research, authorship, and/or publication of this article: The study was supported by MicroPort Orthopedics Inc. (Grant Number 04.02 T003).

© 2023 Yan L et al. *JAMA Network Open.*

Laffosse2008

Bias	Authors' judgement	Support for judgement
Random sequence generation (selection bias)	Unclear risk	Not reported
Allocation concealment (selection bias)	Unclear risk	Not reported
Blinding of participants and personnel (performance bias)	Unclear risk	Not reported
Blinding of outcome assessment (detection bias)	Unclear risk	Not reported
Incomplete outcome data (attrition bias)	Low risk	There was no loss of follow-up.
Selective reporting (reporting bias)	Unclear risk	Not reported
Other bias	Unclear risk	Not reported

© 2023 Yan L et al. *JAMA Network Open.*

Landgraeber2013

Bias	Authors' judgement	Support for judgement
Random sequence generation (selection bias)	Low risk	Using computer-generated cards,
Allocation concealment (selection bias)	Unclear risk	Not reported
Blinding of participants and personnel (performance bias)	Unclear risk	Not reported
Blinding of outcome assessment (detection bias)	Low risk	The observers were blinded and were not involved in the surgery
Incomplete outcome data (attrition bias)	Low risk	Five patients died during the first 3.5 years after surgery. The causes of death were not related to the hip replacement procedure. Ten further patients were not available for all clinical follow-up examinations.
Selective reporting (reporting bias)	Unclear risk	Not reported
Other bias	Low risk	Conflict of interests: the authors declare no potential conflict of interests.

© 2023 Yan L et al. *JAMA Network Open.*

Li2021

**Bias**	**Authors' judgement**	**Support for judgement**
Random sequence generation (selection bias)	Unclear risk	Not reported
Allocation concealment (selection bias)	Unclear risk	Not reported
Blinding of participants and personnel (performance bias)	Unclear risk	Not reported
Blinding of outcome assessment (detection bias)	Unclear risk	Not reported
Incomplete outcome data (attrition bias)	Low risk	There was no loss of follow-up.
Selective reporting (reporting bias)	Unclear risk	Not reported
Other bias	Low risk	All authors declare no conflict of interest.

© 2023 Yan L et al. *JAMA Network Open.*

Martin2011

**Bias**	**Authors' judgement**	**Support for judgement**
Random sequence generation (selection bias)	Unclear risk	Patients were randomly allocated to either the anterolateral approach of Röttinger (n = 42) or the Hardinge lateral approach (n = 41).
Allocation concealment (selection bias)	Unclear risk	Not reported
Blinding of participants and personnel (performance bias)	Unclear risk	Not reported
Blinding of outcome assessment (detection bias)	Low risk	Observers were blinded.
Incomplete outcome data (attrition bias)	Low risk	No loss of follow-up
Selective reporting (reporting bias)	Unclear risk	Not reported
Other bias	Low risk	No support from equipment manufacturers

© 2023 Yan L et al. *JAMA Network Open.*

Matziolis2011

Bias	Authors' judgement	Support for judgement
Random sequence generation (selection bias)	Unclear risk	Forty patients who received a THA were enrolled in this prospective randomized study.
Allocation concealment (selection bias)	Unclear risk	Not reported
Blinding of participants and personnel (performance bias)	Unclear risk	Not reported
Blinding of outcome assessment (detection bias)	Unclear risk	Not reported
Incomplete outcome data (attrition bias)	Low risk	No loss of follow-up
Selective reporting (reporting bias)	Unclear risk	Not reported
Other bias	Low risk	Conflict of interest The authors declare that they have no conflict of interest.

© 2023 Yan L et al. *JAMA Network Open.*

Mayr 2009

Bias	Authors' judgement	Support for judgement
Random sequence generation (selection bias)	low risk	The type of surgical approach was selected according to a computed randomization list.
Allocation concealment (selection bias)	Unclear risk	Not reported
Blinding of participants and personnel (performance bias)	Unclear risk	Not reported
Blinding of outcome assessment (detection bias)	Unclear risk	Not reported
Incomplete outcome data (attrition bias)	low risk	Only a few patients lost one or two follow-ups (3 on 6 weeks and 2 at 12 weeks).
Selective reporting (reporting bias)	Unclear risk	Not reported
Other bias	low risk	The authors declare that they do not have any financial or personal relationships with other people or organizations that could have inappropriately influenced this study.

© 2023 Yan L et al. *JAMA Network Open.*

Mazoochian 2009

Bias	Authors' judgement	Support for judgement
Random sequence generation (selection bias)	high risk	As a result he will perform his or her more favoured procedure better and the surgeon cannot be blinded for the procedure he or she performs.
Allocation concealment (selection bias)	high risk	As a result he will perform his or her more favoured procedure better and the surgeon cannot be blinded for the procedure he or she performs.
Blinding of participants and personnel (performance bias)	high risk	A limitation to the observed studies, as well as our own, is the fact that patients cannot be blinded for the approach chosen for a period exceeding the time required for wound healing.
Blinding of outcome assessment (detection bias)	Unclear risk	Not reported
Incomplete outcome data (attrition bias)	low risk	No lost of follow-up.
Selective reporting (reporting bias)	Unclear risk	Not reported
Other bias	Unclear risk	Not reported

© 2023 Yan L et al. *JAMA Network Open.*

Meneghini2008

Bias	Authors' judgement	Support for judgement
Random sequence generation (selection bias)	Low risk	Computer randomization to 1 of 3 surgical approaches was performed.
Allocation concealment (selection bias)	Unclear risk	Not reported
Blinding of participants and personnel (performance bias)	Unclear risk	Not reported
Blinding of outcome assessment (detection bias)	Unclear risk	Not reported
Incomplete outcome data (attrition bias)	Low risk	Two patients were unable to complete the required preoperative gait analysis due to severe bilateral hip pain, stiffness, and subsequent gait disturbance.One patient in the anterolateral MIS group suffered an early postoperative infection and underwent irrigation and debridement with component retention at 3 weeks and, therefore, was removed from the study.
Selective reporting (reporting bias)	Unclear risk	Not reported
Other bias	High risk	Benefits or funds were received in partial or total support of the research material described in this article. These benefits orsupport were received from the following sources: Orthopaedic Research and Education Foundation, Rosemont, Illinois.

© 2023 Yan L et al. *JAMA Network Open.*

Meng2021

Bias	Authors' judgement	Support for judgement
Random sequence generation (selection bias)	Unclear risk	Not reported
Allocation concealment (selection bias)	Unclear risk	Not reported
Blinding of participants and personnel (performance bias)	Unclear risk	Not reported
Blinding of outcome assessment (detection bias)	Unclear risk	Not reported
Incomplete outcome data (attrition bias)	Low risk	There was no loss of follow-up.
Selective reporting (reporting bias)	Low risk	registered on the Research Registry (https://www.researchregistry.com; No. Researchregistry5326).
Other bias	Low risk	The authors thank the anesthesiologists, nurses, and research pharmacy staff from the West China Hospital, Sichuan University for their support and collaboration. Funding: Research funding and support was provided by the National Health and Family Planning Commission of the People's Republic of China (No. 201302007) and the Sichuan Science and Technology Support Project (No. 2018SZ0145 and No. 2018SZYZF000).

© 2023 Yan L et al. *JAMA Network Open.*

		Weikun Meng received financial support from the China Scholarship Council.

© 2023 Yan L et al. *JAMA Network Open.*

Mjaaland2019

**Bias**	**Authors' judgement**	**Support for judgement**
Random sequence generation (selection bias)	Low risk	The allocation sequence was generated by two of the authors (KEM, KK) by drawing from box notes with the word “anterior” or “lateral” hidden on them and placing them in opaque, sealed envelopes.The envelopes were then drawn from a box again and sequentially numbered.
Allocation concealment (selection bias)	Low risk	The sequence was concealed until assignment, which was done the day before surgery.
Blinding of participants and personnel (performance bias)	Unclear risk	Not reported
Blinding of outcome assessment (detection bias)	Low risk	A physiotherapist (ELM, ABS, HEA) blinded to the planned and used approach assessed all patients preoperatively and at subsequent followup
Incomplete outcome data (attrition bias)	Low risk	Of the 164 randomized patients, 154 patients (94%) completed the 24 month followup with five patients lost in each group.
Selective reporting (reporting bias)	Low risk	registered on ClinicalTrials.gov (ClinicalTrials.gov identifier: NCT01578746)
Other bias	Low risk	All ICMJE Conflict of Interest Forms for authors and Clinical Orthopaedics and Related Research® editors and board members are on file with the publication and can be viewed on request.

© 2023 Yan L et al. *JAMA Network Open.*

Moerenhout2020

Bias	Authors' judgement	Support for judgement
Random sequence generation (selection bias)	Low risk	This process used random blocks of 2 and 4, ensuring that group allocation was equal throughout the recruitment period.
Allocation concealment (selection bias)	Low risk	Group allocation was made immediately before surgery by an independent research coworker using sequentially numbered, sealed envelopes containing the designated surgical approach.
Blinding of participants and personnel (performance bias)	Low risk	Because of the nature of the intervention, it was impossible for the investigator and the patients to be blinded. However, the decision to discharge patients was made by physiotherapists who were blinded to treatment group assignment, and they made the decision on the basis of objective criteria.
Blinding of outcome assessment (detection bias)	Low risk	Data were collected by an independent research assistant at the outpatient clinic of both surgeons.
Incomplete outcome data (attrition bias)	Low risk	One patient in the PA group was lost to follow-up after 6 months; 4 patients (2 in the PA group, 2 in the DAA group) were lost to follow-up after 1 year.
Selective reporting (reporting bias)	Low risk	The trial was registered retrospectively with ClinicalTrials.gov (NCT03673514).
Other bias	Low risk	K. Moerenhout was supported by a grant for an arthroplasty fellowship awarded by the Édouard Samson fund from Hôpital Sacré-Cœur de Montréal; the Fonds de bourses Swiss Orthopaedics, Switzerland; and the Fonds de Perfectionnement du CHUV (Centre hospitalier universitaire Vaudois), Switzerland. There were no sources of outside funding for this study.

© 2023 Yan L et al. *JAMA Network Open.*

Muller2011

**Bias**	**Authors' judgement**	**Support for judgement**
Random sequence generation (selection bias)	Low risk	Randomization was done by throwing a dice, whereby an uneven number meant the standard approach and an even number the minimally invasive approach.
Allocation concealment (selection bias)	Low risk	A sealed envelope opened before surgery was used to determine the patient group.
Blinding of participants and personnel (performance bias)	Unclear risk	Not reported
Blinding of outcome assessment (detection bias)	Low risk	All analysis of the clinical data was carried out by the same single observer (M.M.) who was blinded to the patients.
Incomplete outcome data (attrition bias)	Low risk	No loss of follow-up
Selective reporting (reporting bias)	Low risk	registered in a clinical trial registry (GCTR, registry number: DRKS00000152).
Other bias	Unclear risk	Not reported

© 2023 Yan L et al. *JAMA Network Open.*

Muller2012

Bias	Authors' judgement	Support for judgement
Random sequence generation (selection bias)	Low risk	The randomisation was carried out by throwing dice where uneven numbers implied the mDL group and even numbers the ALMI group.
Allocation concealment (selection bias)	Unclear risk	Not reported
Blinding of participants and personnel (performance bias)	Unclear risk	Not reported
Blinding of outcome assessment (detection bias)	Unclear risk	All clinical assessments were conducted by an independent observer (M.M.) who was blinded to the patient cohorts and not involved in the operations.
Incomplete outcome data (attrition bias)	Low risk	All recruited patients underwent the complete follow-up.
Selective reporting (reporting bias)	Unclear risk	Not reported
Other bias	Unclear risk	Not reported

© 2023 Yan L et al. *JAMA Network Open.*

Nistor2017

Bias	Authors' judgement	Support for judgement
Random sequence generation (selection bias)	Low risk	Randomization to the anterior or lateral group was computer generated.
Allocation concealment (selection bias)	Unclear risk	Not reported
Blinding of participants and personnel (performance bias)	Unclear risk	Not reported
Blinding of outcome assessment (detection bias)	Unclear risk	Not reported
Incomplete outcome data (attrition bias)	Low risk	Four patients declined participating in the study and one patient from the lateral group voluntarily withdrew from the study after surgery. One patient from the lateral group developed a pulmonary embolism in the second postoperative week and was excluded from the study. We had no loss to follow-up.
Selective reporting (reporting bias)	Unclear risk	Not reported
Other bias	Low risk	There are no funding sources in support of this research.The authors have no conflict ofinterest to declare.

© 2023 Yan L et al. *JAMA Network Open.*

Ogonda 2005

Bias	Authors' judgement	Support for judgement
Random sequence generation (selection bias)	Low risk	On the evening before surgery, a sealed envelope was used to determine the patient randomization group.
Allocation concealment (selection bias)	Low risk	The operating surgeon, who was not involved in patient randomization, was then informed of the incision to be used at the time of templating of preoperative radiographs.
performance bias	Unclear risk	Not reported
Blinding of outcome assessment (detection bias)	Low risk	Following surgery, all patients had a standard-length wound dressing, ensuring that the patients and all staff, except those directly tending to wound care, were blinded to the technique used.When a change of dressing was required, the standard-length dressing was reapplied and was used for the duration of the hospital stay.
Incomplete outcome data (attrition bias)	Low risk	A total of 215 patients were evaluated at six weeks following surgery. One patient was seen at eight weeks, and another was seen at three months. The remaining two patients had died in the early postoperative period. Both were excluded from the analysis.
Selective reporting (reporting bias)	Unclear risk	Not reported
Other bias	Low risk	In support of their research or preparation of this manuscript, one or more of the authors received grants or outside funding from DePuy International. None of the authors received payments or other benefits or a commitment or agreement to provide such benefits from a commercial entity. No commercial entity paid or directed, or agreed to pay or direct, any benefits to any research fund, foundation, educational institution, or other charitable or nonprofit organization.

© 2023 Yan L et al. *JAMA Network Open.*

Pagnano2009

Bias	Authors' judgement	Support for judgement
Random sequence generation	Low risk	the randomization was carried out with use of a secure, web-based computerized process
Allocation concealment	Low risk	that was developed and implemented by our Department of Biostatistics
Blinding of participants and personnel (performance bias)	Low risk	Randomization was done after the surgeon had completed the preoperative examination and discussion with the patient.Both the patient and the surgeon were blinded with regard to the group assignment prior to surgery but not during or after the procedure.
Blinding of outcome assessment (detection bias)	Low risk	The study coordinator who collected the clinical data remained blinded to the group assignment throughout the follow-up period.
Incomplete outcome data (attrition bias)	Low risk	The flow chart shows the specific number and time of lost visits in each period.
Selective reporting	Unclear risk	Not reported
Other bias	Low risk	Disclosure: The authors did not receive any outside funding or grants in support of their research for or preparation of this work. One or more of the authors or a member of his or her immediate family received, in any one year, payments or other benefits in excess of $10,000 or a commitment or agreement to provide such benefits from a commercial entity (Zimmer, DePuy). Also, a commercial entity (OREF [Orthopaedic Research and Education Foundation]) paid or directed in any one year, or agreed to pay or direct, benefits in excess of $10,000 to a research fund, foundation, division, center, clinical practice, or other charitable or nonprofit organization with which one or more of the authors, or a member of his or her immediate family, is affiliated or associated.

© 2023 Yan L et al. *JAMA Network Open.*

Parvizi 2016

Bias	Authors' judgement	Support for judgement
Random sequence generation (selection bias)	Low risk	Randomization was performed using a random number generator in an electronic spread sheet.
Allocation concealment (selection bias)	Unclear risk	Not reported
Blinding of participants and personnel (performance bias)	Unclear risk	Not reported
Blinding of outcome assessment (detection bias)	Low risk	The independent blinded observer collected the data on all patients.
Incomplete outcome data (attrition bias)	Low risk	All patients were seen in the office and examined by an independent observer as well as the senior surgeon at or around 4 weeks postoperatively. Patients were then followed at routine intervals, which included a visit at 6 months, 1 year, and then at 2 years.
Selective reporting (reporting bias)	Unclear risk	Not reported
Other bias	Unclear risk	Not reported

© 2023 Yan L et al. *JAMA Network Open.*

Pospischill 2010

Bias	Authors' judgement	Support for judgement
Random sequence generation (selection bias)	low risk	The remaining forty patients were randomized into two groups following a computerized protocol.
Allocation concealment (selection bias)	high risk	Each patient received either the number 0 for the minimally invasive group or 1 for the standard group.
Blinding of participants and personnel (performance bias)	low risk	Neither the surgeon nor the staff treating the patients after the operation were blinded to the technique used.
Blinding of outcome assessment (detection bias)	Unclear risk	Not reported
Incomplete outcome data (attrition bias)	low risk	No lost of follow-up.
Selective reporting (reporting bias)	low risk	The trial was registered in the ClinicalTrials.gov Protocol Registration System with the ClinicalTrials.gov Identifier NCT00831363.
Other bias	low risk	The authors did not receive any outside funding or grants in support of their research for or preparation of this work. Neither they nor a member of their immediate families received payments or other benefits or a commitment or agreement to provide such benefits from a commercial entity.

© 2023 Yan L et al. *JAMA Network Open.*

Reichert2018

Bias	Authors' judgement	Support for judgement
Random sequence generation (selection bias)	Low risk	148 consecutive patients were enrolled and allocated to the treatment groups by the principal investigator utilizing a computer generated block randomization
Allocation concealment (selection bias)	Low risk	It was not possible to blind the patient for the allocated surgical technique, as the surgical incision site of the studied approaches was different.
Blinding of participants and personnel (performance bias)	Low risk	It was not possible to blind the patient for the allocated surgical technique, as the surgical incision site of the studied approaches was different.
Blinding of outcome assessment (detection bias)	Unclear risk	Not reported
Incomplete outcome data (attrition bias)	Low risk	The flow chart shows the specific number and time of lost visits in each period.
Selective reporting (reporting bias)	Low risk	Trial registration: DRKS00014808 (German Clinical Trial Register DRKS); date of registration: 31.05.2018.
Other bias	Low risk	The study was financially supported by the Deutsche Arthrose-Hilfe (Grant P178-A49-Eulert-EP2nöth3-hüfte-opII-156 k-2008-12 and P235-A284-RudertEP2-nöth1-hüfte-op-II-67 k-2001-12). The funding body was not involved in collection, analysis, and interpretation of data and in writing the manuscript.

© 2023 Yan L et al. *JAMA Network Open.*

Restrepo2010

Bias	Authors' judgement	Support for judgement
Random sequence generation (selection bias)	Low risk	computer-generated cards
Allocation concealment (selection bias)	Unclear risk	Not reported
Blinding of participants and personnel (performance bias)	Unclear risk	Not reported
Blinding of outcome assessment (detection bias)	Unclear risk	Not reported
Incomplete outcome data (attrition bias)	Low risk	The flow chart shows the specific number and time of lost visits in each period.
Selective reporting (reporting bias)	Unclear risk	Not reported
Other bias	High risk	Benefits or funds were received in partial or total support of the research material described in this article. These benefits or support were received from the following sources: J.P., Consultant for Stryker Orthopaedics (Mahwah, NJ), Intellectual Properties of SmarTech (Philadelphia, Pa).

© 2023 Yan L et al. *JAMA Network Open.*

Rosenlund2017

Bias	Authors' judgement	Support for judgement
Random sequence generation (selection bias)	Low risk	block randomization was performed using a computer-generated list,sequence was generated by a third person who was not involved in the trial.
Allocation concealment (selection bias)	Low risk	The sequence was written on paper and placed in sealed opaque consecutively numbered envelopes by a secretary not involved in the trial.The envelopes were opened in the order given, and the patient was allocated to a treatment group and scheduled for surgery.
Blinding of participants and personnel (performance bias)	Low risk	The patients were blinded to treatment and informed, prior to participation, that the type of intervention would not be revealed to them.
Blinding of outcome assessment (detection bias)	Low risk	The principal investigator was kept blind throughout the trial and the statistical analyses.
Incomplete outcome data (attrition bias)	Low risk	The flow chart shows the specific number and time of lost visits in each period.
Selective reporting (reporting bias)	Low risk	It was also registered at ClinicalTrials.gov (identifi er: NCT01616667)
Other bias	Low risk	The trial was supported by the Danish Rheumatoid Association, University of Southern Denmark, Region of Zealand, Region of Southern Denmark, the Bevica Foundation, the Bjarne Jensen Foundation, and Odense University Hospital. None of the trial sponsors played any role in the trial design, data collection, data analysis, or interpretation; nor did they have any infl uence on the writing of the manuscript or the decision to submit the manuscript for publication.

© 2023 Yan L et al. *JAMA Network Open.*

Roy2010

**Bias**	**Authors' judgement**	**Support for judgement**
Random sequence generation (selection bias)	Unclear risk	Not reported
Allocation concealment (selection bias)	Low risk	a total of 56 patients were randomised by sealed envelope between the MIS group and the standard incision group.
Blinding of participants and personnel (performance bias)	Unclear risk	Not reported
Blinding of outcome assessment (detection bias)	Unclear risk	Not reported
Incomplete outcome data (attrition bias)	Low risk	Patients were all followed for a minimum of 2 years after surgery.
Selective reporting (reporting bias)	Unclear risk	Not reported
Other bias	Low risk	The authors have declared no conflict of interest.

© 2023 Yan L et al. *JAMA Network Open.*

Rykov2021

Bias	Authors' judgement	Support for judgement
Random sequence generation (selection bias)	Low risk	A random allocation set of the type of THA approach was generated by means of a computer.
Allocation concealment (selection bias)	Low risk	These allocations were then sealed in consecutively numbered opaque envelopes.The THA approach was randomly assigned by opening the next sealed envelope by an independent investigator.
Blinding of participants and personnel (performance bias)	Unclear risk	Not reported
Blinding of outcome assessment (detection bias)	Low risk	and the quadratus femoris) were assessed by one radiologist (TM), who was blinded for the THA approach.The X-rays of included patients were anonymized, randomized, and then independently assessed by two authors (KR and BH), who were thus blinded to the THA approach.
Incomplete outcome data (attrition bias)	Low risk	For all 46 patients data regarding functional outcome and radiographic measurements were available, as there was no loss to follow-up at the final followup.
Selective reporting (reporting bias)	Low risk	was registered in the Dutch Trial Register (NTR3926).
Other bias	Low risk	No author associated with this paper has disclosed any potential or pertinent conflicts which may be perceived to have impending conflict with this work. For full disclosure statements refer to https://doi.org/10.1016/j.arth.2021.05.009.

© 2023 Yan L et al. *JAMA Network Open.*

Schwarze2018

Bias	Authors' judgement	Support for judgement
Random sequence generation (selection bias)	Low risk	Surgery was performed by one of five experienced surgeons with a randomized approach according to a randomization list.The list was based on a block randomization with a block size of six.After inclusion of a patient, group allocation was obtained from an uninvolved statistician.
Allocation concealment (selection bias)	Unclear risk	Not reported
Blinding of participants and personnel (performance bias)	Unclear risk	Not reported
Blinding of outcome assessment (detection bias)	Unclear risk	Not reported
Incomplete outcome data (attrition bias)	Low risk	At each follow-up, 6-10 patients were temporarily lost to follow-up
Selective reporting (reporting bias)	Unclear risk	Not reported
Other bias	High risk	The study was funded by Aesculap AG Tuttingen.

© 2023 Yan L et al. *JAMA Network Open.*

Sershon2017

**Bias**	**Authors' judgement**	**Support for judgement**
Random sequence generation (selection bias)	Unclear risk	Not reported
Allocation concealment (selection bias)	Unclear risk	Not reported
Blinding of participants and personnel (performance bias)	Unclear risk	Not reported
Blinding of outcome assessment (detection bias)	Low risk	Patient outcome measures were assessed on an annual basis by a nurse, resident or fellow who was not involved in the intial operative procedure.
Incomplete outcome data (attrition bias)	Unclear risk	There are corresponding records and explanations for the lost people.
Selective reporting (reporting bias)	Low risk	clinicaltrials.gov (NCT00594893)
Other bias	Unclear risk	Not reported

© 2023 Yan L et al. *JAMA Network Open.*

Shitama2009

**Bias**	**Authors' judgement**	**Support for judgement**
Random sequence generation (selection bias)	Unclear risk	Not reported
Allocation concealment (selection bias)	Unclear risk	Not reported
Blinding of participants and personnel (performance bias)	Unclear risk	Not reported
Blinding of outcome assessment (detection bias)	Unclear risk	Not reported
Incomplete outcome data (attrition bias)	Low risk	There was no loss of follow-up.
Selective reporting (reporting bias)	Unclear risk	Not reported
Other bias	Unclear risk	Not reported

© 2023 Yan L et al. *JAMA Network Open.*

Speranza 2007

Bias	Authors' judgement	Support for judgement
Random sequence generation (selection bias)	Unclear risk	Not reported
Allocation concealment (selection bias)	Unclear risk	Not reported
Blinding of participants and personnel (performance bias)	Unclear risk	Not reported
Blinding of outcome assessment (detection bias)	Unclear risk	Not reported
Incomplete outcome data (attrition bias)	Low risk	At follow-up one patient in group B was lost and two patients (one in group A and one in group B) refused to undergo radiological evaluation and were excluded from the study.
Selective reporting (reporting bias)	Unclear risk	Not reported
Other bias	Unclear risk	Not reported

© 2023 Yan L et al. *JAMA Network Open.*

Takada2018

Bias	Authors' judgement	Support for judgement
Random sequence generation (selection bias)	Low risk	randomization using envelope method
Allocation concealment (selection bias)	Low risk	The patients were blinded regarding the selected approach throughout the follow-up period.
Blinding of participants and personnel (performance bias)	Low risk	Not reported
Blinding of outcome assessment (detection bias)	High risk	The surgeon who evaluated the patients postoperatively was not strictly blinded to the approaches.
Incomplete outcome data (attrition bias)	Low risk	Not reported
Selective reporting (reporting bias)	Unclear risk	Not reported
Other bias	Low risk	All authors declare that they have no conflict of interest.

© 2023 Yan L et al. *JAMA Network Open.*

Tan2019

Bias	Authors' judgement	Support for judgement
Random sequence generation (selection bias)	Low risk	Random allocation was computer generated (www.randomization.com)
Allocation concealment (selection bias)	Low risk	using sealed identical opaque envelopes opened in theatre.
Blinding of participants and personnel (performance bias)	Unclear risk	Not reported
Blinding of outcome assessment (detection bias)	Low risk	Assessments were undertaken by independent and blinded physiotherapists pre-operatively and at 2 and 6 weeks, 3 months, 1, 2 and 10 years after surgery.
Incomplete outcome data (attrition bias)	Low risk	The flow chart shows the specific number and time of lost visits in each period.
Selective reporting (reporting bias)	Low risk	was registered with the Australian New Zealand Clinical Trials Registry
Other bias	Low risk	This research did not receive any specific grant from funding agencies in the public, commercial, or not-for-profit sectors.

© 2023 Yan L et al. *JAMA Network Open.*

Taunton2014

Bias	Authors' judgement	Support for judgement
Random sequence generation (selection bias)	Unclear risk	Not reported
Allocation concealment (selection bias)	Low risk	The patient was blinded with regard to the study group prior to the procedure, but it was not possible or planned for either the patient or the surgeon to be blinded after the procedure.
Blinding of participants and personnel (performance bias)	Unclear risk	Not reported
Blinding of outcome assessment (detection bias)	Unclear risk	Not reported
Incomplete outcome data (attrition bias)	Low risk	No patient was lost to follow-up.
Selective reporting (reporting bias)	Low risk	it was registered with clinical trials.gov (NCT01613508)
Other bias	Unclear risk	Not reported

© 2023 Yan L et al. *JAMA Network Open.*

Taunton2018

Bias	Authors' judgement	Support for judgement
Random sequence generation (selection bias)	Unclear risk	Not reported
Allocation concealment (selection bias)	Unclear risk	Not reported
Blinding of participants and personnel (performance bias)	Low risk	nonblinded
Blinding of outcome assessment (detection bias)	High risk	nonblinded
Incomplete outcome data (attrition bias)	Low risk	The flow chart shows the specific number and time of lost visits in each period.
Selective reporting (reporting bias)	Unclear risk	Not reported
Other bias	Low risk	All ICMJE Conflict of Interest Forms for authors and Clinical Orthopaedics and Related Research® editors and board members are on file with the publication and can be viewed on request.

© 2023 Yan L et al. *JAMA Network Open.*

Thaler 2018

Bias	Authors' judgement	Support for judgement
Random sequence generation (selection bias)	low risk	The type of surgical approach was selected according to a computerized randomization list.
Allocation concealment (selection bias)	Unclear risk	Not reported
Blinding of participants and personnel (performance bias)	Unclear risk	Not reported
Blinding of outcome assessment (detection bias)	Unclear risk	Not reported
Incomplete outcome data (attrition bias)	low risk	One patient in the DAA group was lost to follow-up after two years and one patient in the AL group died.
Selective reporting (reporting bias)	Unclear risk	Not reported
Other bias	low risk	There are no other conflicts of interest to declare.

© 2023 Yan L et al. *JAMA Network Open.*

Ulivi2021

Bias	Authors' judgement	Support for judgement
Random sequence generation (selection bias)	Low risk	The subjects were randomly allocated to one of the 2 treatment groups with a computer-generated 1:1 randomization list.
Allocation concealment (selection bias)	Unclear risk	Not reported
Blinding of participants and personnel (performance bias)	Unclear risk	Not reported
Blinding of outcome assessment (detection bias)	Unclear risk	Not reported
Incomplete outcome data (attrition bias)	Unclear risk	Not reported
Selective reporting (reporting bias)	Low risk	The study was registered at clinicaltrials.gov (NCT04358250).
Other bias	Low risk	The study was supported by the Italian Ministry of Health (Ricerca Corrente)

© 2023 Yan L et al. *JAMA Network Open.*

Varela 2013

Bias	Authors' judgement	Support for judgement
Random sequence generation (selection bias)	Low risk	Using a table of randomized numbers, the patients were divided into two groups of 25 members.
Allocation concealment (selection bias)	Unclear risk	Not reported
Blinding of participants and personnel (performance bias)	Unclear risk	Not reported
Blinding of outcome assessment (detection bias)	Unclear risk	Not reported
Incomplete outcome data (attrition bias)	low risk	No lost of follow-up.
Selective reporting (reporting bias)	Unclear risk	Not reported
Other bias	low risk	The authors have not received any Wnancial support and will not obtain any economical bonus from private or public institution.

© 2023 Yan L et al. *JAMA Network Open.*

Vasilakis 2012

Bias	Authors' judgement	Support for judgement
Random sequence generation (selection bias)	low risk	Hips were assigned to group A or B by a computer-generated randomization schedule.
Allocation concealment (selection bias)	low risk	Received a set of sealed, opaque envelopes containing the randomization assignment for each patient.
Blinding of participants and personnel (performance bias)	low risk	The treating surgeon and patient were blinded to group assignment until after surgical treatment.
Blinding of outcome assessment (detection bias)	low risk	Radiologists (I.V., E.S.) and orthopedic surgeons (V.V., P.K.) were blinded to the surgical approach used.
Incomplete outcome data (attrition bias)	low risk	There is 1/2 lost of follow up in MIS/conventional group.
Selective reporting (reporting bias)	Unclear risk	Not reported
Other bias	Unclear risk	Not reported

© 2023 Yan L et al. *JAMA Network Open.*

Wang2019

Bias	Authors' judgement	Support for judgement
Random sequence generation (selection bias)	Unclear risk	Not reported
Allocation concealment (selection bias)	Low risk	opaque envelope method.
Blinding of participants and personnel (performance bias)	Low risk	Surgical performers were blinded to the randomization of the participants.
Blinding of outcome assessment (detection bias)	Unclear risk	Not reported
Incomplete outcome data (attrition bias)	Low risk	The flow chart shows the specific number and time of lost visits in each period.
Selective reporting (reporting bias)	Low risk	Meanwhile, the study has been registered in Chinese Clinical Trial Registry (ChiCTR), the Clinical Trial Registry Number is ChiCTR-IOR17013007.
Other bias	Low risk	The authors declare no conflict of interest. No specific funding was received.

© 2023 Yan L et al. *JAMA Network Open.*

Wang2021

Bias	Authors' judgement	Support for judgement
Random sequence generation (selection bias)	Low risk	All patients were randomized into traditional longitudinan incision group (control group) or bikini incision group (bikini group) using a computer-generated list of numbers (Excel, Microsoft Corporation, Redmond, WA)
Allocation concealment (selection bias)	Low risk	The numbers were then sealed in opaque envelopes by an investigator (QW), who asked patients to select an envelope on the morning of their surgery.
Blinding of participants and personnel (performance bias)	Low risk	Investigators blinded to group assignment performed postoperative assessments of functional recovery (LC) and statistical analysis (ZY).
Blinding of outcome assessment (detection bias)	Low risk	Investigators blinded to group assignment performed postoperative assessments of functional recovery (LC) and statistical analysis (ZY).
Incomplete outcome data (attrition bias)	Low risk	The flow chart shows the specific number and time of lost visits in each period.
Selective reporting (reporting bias)	Low risk	The Clinical Trial Registration Number: ChiCTR1900022870.
Other bias	Low risk	Funding Sources: Funded by 1.3.5 project for disciplines of excellence, West China Hospital, Sichuan University, China

© 2023 Yan L et al. *JAMA Network Open.*

Witzleb2009

Bias	Authors' judgement	Support for judgement
Random sequence generation (selection bias)	Unclear risk	random numbers were generated by means of a block permutation algorithm
Allocation concealment (selection bias)	Unclear risk	Not reported
Blinding of participants and personnel (performance bias)	High risk	At the day before surgery, additional standard patient information about details of the surgery at hand was communicated to each individual patient by the surgeon.
Blinding of outcome assessment (detection bias)	Unclear risk	Not reported
Incomplete outcome data (attrition bias)	Unclear risk	Not reported
Selective reporting (reporting bias)	Unclear risk	Not reported
Other bias	Low risk	The authors have no commercial or political interests in the clinical products and findings presented in this manuscript. The investigation was not granted

© 2023 Yan L et al. *JAMA Network Open.*

Xie2017

Bias	Authors' judgement	Support for judgement
Random sequence generation (selection bias)	Unclear risk	Not reported
Allocation concealment (selection bias)	Unclear risk	Not reported
Blinding of participants and personnel (performance bias)	Unclear risk	Not reported
Blinding of outcome assessment (detection bias)	Unclear risk	Not reported
Incomplete outcome data (attrition bias)	Low risk	The flow chart shows the specific number and time of lost visits in each period.
Selective reporting (reporting bias)	Unclear risk	Not reported
Other bias	Low risk	This study was supported by the Health Science and Technology Special Projects Foundation of Zhenjiang, Jiangsu Province (SHW2016005).

© 2023 Yan L et al. *JAMA Network Open.*

Yang2019

Bias	Authors' judgement	Support for judgement
Random sequence generation (selection bias)	Low risk	By simple randomization method with table of random digit,
Allocation concealment (selection bias)	Low risk	On the evening before surgery, a sealed envelope containing a random group assignment was used to determine the patient group.
Blinding of participants and personnel (performance bias)	Low risk	their incisions would be obscured during their hospital stays
Blinding of outcome assessment (detection bias)	Low risk	the patients and all staff, except those directly attending to wound care, were blind to the technique used.
Incomplete outcome data (attrition bias)	Low risk	All patients were followed up at the third month and third year after operation.
Selective reporting (reporting bias)	Unclear risk	Not reported
Other bias	Unclear risk	Not reported

© 2023 Yan L et al. *JAMA Network Open.*

Zhao2017

**Bias**	**Authors' judgement**	**Support for judgement**
Random sequence generation (selection bias)	Low risk	OPENING NUMBERED AND SEALED ENVELOP
Allocation concealment (selection bias)	Low risk	They didn't know the operation until the night before the operation
Blinding of participants and personnel (performance bias)	Low risk	Following surgery, all patients had a standard-length wound dressing, ensuring that the patients and all staff, except those directly attending to wound care, were blind to the technique used.
Blinding of outcome assessment (detection bias)	Low risk	An independent investigator, also blind to the length of the incisions, analyzed the postoperative radiographs.
Incomplete outcome data (attrition bias)	Low risk	There are corresponding records and explanations for the lost people
Selective reporting (reporting bias)	Low risk	CTR-INR-16010136
Other bias	Low risk	This research did not receive financial support from funding agencies in the public, commercial, or not-for-profit sectors.

© 2023 Yan L et al. *JAMA Network Open.*

Zomar2018

Bias	Authors' judgement	Support for judgement
Random sequence generation (selection bias)	Unclear risk	Not reported
Allocation concealment (selection bias)	Unclear risk	Not reported
Blinding of participants and personnel (performance bias)	Unclear risk	Not reported
Blinding of outcome assessment (detection bias)	High risk	The assessor was not blinded to surgical approach.
Incomplete outcome data (attrition bias)	Low risk	The flow chart shows the specific number and time of lost visits in each period.
Selective reporting (reporting bias)	Unclear risk	Not reported
Other bias	Low risk	The author(s) declared no potential conflicts of interest with respect to the research, authorship, and/or publication of this article.

© 2023 Yan L et al. *JAMA Network Open.*

eTable 6. Certainty of Evidence for Direct, Indirect, and Network Estimates

Outcome : Hip score change

**Comparison groups**		**Direct evidence**								
Arm 1	Arm 2	MD (95%CrI)	RoB	Inconsistency	Indirectness	Publicaion bias	Direct rating without imprecision	Need to rate indiret?	Imprecision	Direct rating with imprecision
SuperPath	2-incision									
SuperPath	MIS-PA	10.20(1.46, 18.94)	no	no	no	no	high	no	no	high
SuperPath	PA	-1.48(-5.56, 2.60)	no	no	no	no	high	no	serious	mod
SuperPath	DAA									
SuperPath	MIS-DLA									
SuperPath	MIS-ALA									
SuperPath	DLA									
2-incision	MIS-PA	0.00(-6.79, 6.79)	no	no	no	no	high	no	serious	mod
2-incision	PA									
2-incision	DAA									

© 2023 Yan L et al. *JAMA Network Open.*

Comparison groups		Direct evidence								
Arm 1	Arm 2	MD (95%CrI)	RoB	Inconsistency	Indirectness	Publicaion bias	Direct rating without imprecision	Need to rate indiret?	Imprecision	Direct rating with imprecision
2-incision	MIS-DLA									
2-incision	MIS-ALA	5.00(-6.44, 16.44)	serious	no	no	no	mod	yes	serious	low
2-incision	DLA									
MIS-PA	PA	0.46(-2.20, 3.13)	no	no	no	no	high	no	serious	mod
MIS-PA	DAA	1.99(-1.52, 5.51)	serious	serious	no	no	low	yes	serious	V low
MIS-PA	MIS-DLA	-8.50(-17.03, 0.03)	serious	no	no	no	mod	yes	serious	low
MIS-PA	MIS-ALA	2.00(-7.05, 11.05)	no	no	no	no	high	no	serious	mod
MIS-PA	DLA	-1.30(-9.25, 6.65)	serious	no	no	no	mod	yes	serious	low
PA	DAA	-0.21(-3.09, 2.67)	no	serious	no	no	mod	yes	serious	low

© 2023 Yan L et al. *JAMA Network Open.*

Comparison groups		Direct evidence								
Arm 1	Arm 2	MD (95%CrI)	RoB	Inconsistency	Indirectness	Publicaion bias	Direct rating without imprecision	Need to rate indiret?	Imprecision	Direct rating with imprecision
PA	MIS-DLA	-1.33(-8.23, 5.57)	serious	no	no	no	mod	yes	serious	low
PA	MIS-ALA	-0.15(-4.13, 3.83)	no	no	no	no	high	no	serious	mod
PA	DLA	6.89(1.86, 11.93)	no	no	no	no	high	no	no	high
DAA	MIS-DLA	0.96(-2.65, 4.57)	serious	no	no	no	mod	yes	serious	low
DAA	MIS-ALA	1.51(-4.22, 7.24)	no	no	no	no	high	no	serious	mod
DAA	DLA	4.33(1.44, 7.22)	serious	no	no	no	mod	yes	no	mod
MIS-DLA	MIS-ALA	-0.72(-4.38, 2.93)	serious	no	no	no	mod	yes	serious	low
MIS-DLA	DLA	3.70(0.60, 6.80)	serious	no	no	no	mod	yes	no	mod
MIS-ALA	DLA	0.93(-3.53, 5.40)	serious	serious	no	no	low	yes	serious	V low

© 2023 Yan L et al. *JAMA Network Open.*

Comparison groups		Indirect evidence					Network evidence					
Arm 1	Arm 2	MD (95%CrI)	First order of the most contribution	Lowest of C1 and C2	Intransitivity	Indirect rating without imprecision	MD (95%CrI)	Incoherence, P-value	Higher rating of direct and indirect without imprecision	Incoherence	NMA imprecision	Network rating with imprecision
SuperPath	2-incision	-0.38(-7.46, 6.69)	MID-PA	high	no	high	-0.38(-7.46, 6.69)	NR	high	no	serious	mod
SuperPath	MIS-PA						0.54(-3.56, 4.65)	0.0141	high	serious	serious	low
SuperPath	PA						0.62(-3.09, 4.34)	0.0141	high	serious	serious	low
SuperPath	DAA	0.96(-3.21, 5.13)	PA	mod	no	mod	0.96(-3.21, 5.13)	NR	mod	no	serious	low
SuperPath	MIS-DLA	1.63(-2.89, 6.16)	PA	mod	no	mod	1.63(-2.89, 6.16)	NR	mod	no	serious	low
SuperPath	MIS-ALA	2.00(-2.49, 6.48)	PA	high	no	high	2.00(-2.49, 6.48)	NR	high	no	serious	mod

© 2023 Yan L et al. *JAMA Network Open.*

Comparison groups		Indirect evidence					Network evidence					
Arm 1	Arm 2	MD (95%CrI)	First order of the most contribution	Lowest of C1 and C2	Intransitivity	Indirect rating without imprecision	MD (95%CrI)	Incoherence, P-value	Higher rating of direct and indirect without imprecision	Incoherence	NMA imprecision	Network rating with imprecision
SuperPath	DLA	5.00(0.58, 9.42)	PA	high	no	high	5.00(0.58, 9.42)	NR	high	no	no	high
2-incision	MIS-PA						0.92(-4.97, 6.82)	0.5917	high	no	serious	mod
2-incision	PA	1.01(-5.13, 7.14)	MID-PA	high	no	high	1.01(-5.13, 7.14)	NR	high	no	serious	mod
2-incision	DAA	1.34(-4.84, 7.52)	MID-PA	low	no	low	1.34(-4.84, 7.52)	NR	low	no	serious	V low
2-incision	MIS-DLA	2.01(-4.37, 8.40)	MID-PA	mod	no	mod	2.01(-4.37, 8.40)	NR	mod	no	serious	low
2-incision	MIS-ALA	1.26(-6.19, 8.72)	MID-PA	high	no	high	2.38(-3.87, 8.62)	0.5917	high	no	serious	mod

© 2023 Yan L et al. *JAMA Network Open.*

Comparison groups		Indirect evidence					Network evidence					
Arm 1	Arm 2	MD (95%CrI)	First order of the most contribution	Lowest of C1 and C2	Intransitivity	Indirect rating without imprecision	MD (95%CrI)	Incoherence, P-value	Higher rating of direct and indirect without imprecision	Incoherence	NMA imprecision	Network rating with imprecision
2-incision	DLA	5.38(-0.96, 11.72)	MID-PA	mod	no	mod	5.38(-0.96, 11.72)	NR	mod	no	serious	low
MIS-PA	PA						0.08(-2.10, 2.26)	0.6267	high	no	serious	mod
MIS-PA	DAA	-0.9(-4.11, 2.31)	PA	mod	no	mod	0.42(-1.95, 2.79)	0.2336	mod	no	serious	low
MIS-PA	MIS-DLA	2.45(-0.76. 5.66)	DAA	low	no	low	1.09(-1.92, 4.09)	0.0186	mod	serious	serious	V low
MIS-PA	MIS-ALA						1.45(-1.54, 4.45)	0.9001	high	no	serious	mod
MIS-PA	DLA	5.31(2.25, 8.37)	DAA	low	no	low	4.46(1.60, 7.31)	0.1285	mod	no	no	mod

© 2023 Yan L et al. *JAMA Network Open.*

Comparison groups		Indirect evidence					Network evidence					
Arm 1	Arm 2	MD (95%CrI)	First order of the most contribution	Lowest of C1 and C2	Intransitivity	Indirect rating without imprecision	MD (95%CrI)	Incoherence, P-value	Higher rating of direct and indirect without imprecision	Incoherence	NMA imprecision	Network rating with imprecision
PA	DAA	0.89(-2.02, 3.79)	MID-PA	low	no	low	0.33(-1.71, 2.38)	0.5988	mod	no	serious	low
PA	MIS-DLA	1.42(-1.49, 4.33)	DAA	mod	no	mod	1.01(-1.67, 3.69)	0.4713	mod	no	serious	low
PA	MIS-ALA						1.37(-1.23, 3.98)	0.3225	high	no	serious	mod
PA	DLA						4.37(1.87, 6.88)	0.2587	high	no	no	high
DAA	MIS-DLA	0.46(-3.55, 2.63)	DLA	mod	no	mod	0.67(-1.68, 3.02)	0.836	mod	no	serious	low
DAA	MIS-ALA						1.04(-1.51, 3.58)	0.8575	high	no	serious	mod

© 2023 Yan L et al. *JAMA Network Open.*

Comparison groups		Indirect evidence					Network evidence					
Arm 1	Arm 2	MD (95%CrI)	First order of the most contribution	Lowest of C1 and C2	Intransitivity	Indirect rating without imprecision	MD (95%CrI)	Incoherence, P-value	Higher rating of direct and indirect without imprecision	Incoherence	NMA imprecision	Network rating with imprecision
DAA	DLA	3.7(0.56, 6.83)	MID-DLA	mod	no	mod	4.04(1.92, 6.16)	0.77	mod	no	no	mod
MIS-DLA	MIS-ALA	1.41(-2.18, 5)	DLA	low	no	low	0.36(-2.20, 2.93)	0.4137	mod	no	serious	low
MIS-DLA	DLA	2.95(-0.52, 6.42)	MIS-ALA	low	no	low	3.37(1.05, 5.68)	0.7518	mod	no	no	mod
MIS-ALA	DLA	4.05(0.88, 7.22)	MID-DLA	mod	no	mod	3.00(0.42, 5.59)	0.2648	mod	no	no	mod

© 2023 Yan L et al. *JAMA Network Open.*

Outcome : Pain score change

**Comparison groups**		**Direct evidence**								
Arm 1	Arm 2	MD (95%CrI)	RoB	Inconsistency	Indirectness	Publicaion bias	Direct rating without imprecision	Need to rate indiret?	Imprecision	Direct rating with imprecision
SuperPath	MIS-DLA									
SuperPath	PA	-0.35(-1.12, 0.42)	no	no	no	no	high	no	serious	mod
SuperPath	DAA									
SuperPath	MIS-PA	-1.32(-2.93, 0.29)	no	no	no	no	high	no	serious	mod
SuperPath	MIS-ALA									
SuperPath	DLA									
MIS-DLA	PA	0.31(-1.12, 1.74)	no	no	no	no	high	no	serious	mod

© 2023 Yan L et al. *JAMA Network Open.*

MIS-DLA	DAA	-0.55(-1.46, 0.36)	serious	no	no	no	mod	yes	serious	low
MIS-DLA	MIS-PA	-0.22(-1.68, 1.24)	no	no	no	no	high	no	serious	mod
MIS-DLA	MIS-ALA	-1.26(-2.77, 0.25)	serious	no	no	no	mod	yes	serious	low
MIS-DLA	DLA									
PA	DAA	0.03(-0.74, 0.80)	no	serious	no	no	mod	yes	serious	low
PA	MIS-PA	0.02(-0.84, 0.87)	no	no	no	no	high	no	serious	mod
PA	MIS-ALA									
PA	DLA	-1.00(-2.69, 0.69)	no	no	no	no	high	no	serious	mod
DAA	MIS-PA	-0.14(-0.95, 0.67)	serious	no	no	no	mod	yes	serious	low
DAA	MIS-ALA	-0.12(-1.54, 1.29)	no	no	no	no	high	no	serious	mod

© 2023 Yan L et al. *JAMA Network Open.*

DAA	DLA	-0.43(-1.23, 0.36)	serious	no	no	no	mod	yes	serious	low
MIS-PA	MIS-ALA									
MIS-PA	DLA									
MIS-ALA	DLA	-0.04(-0.93, 0.84)	no	no	no	no	high	no	serious	mod

© 2023 Yan L et al. *JAMA Network Open.*

Comparison groups		Indirect evidence					Network evidence					
Arm 1	Arm 2	MD (95%CrI)	First order of the most contribution	Lowest of C1 and C2	Intransitivity	Indirect rating without imprecision	MD (95% CrI)	Incoherence, P-value	Higher rating of direct and indirect without imprecision	Incoherence	NMA imprecision	Network rating with imprecision
SuperPath	MIS-DLA	-0.26(-1.26, 0.74)	PA	high	no	high	-0.26(-1.26, 0.74)	NR	high	no	serious	mod
SuperPath	PA						-0.49(-1.19, 0.21)	0.3701	high	no	serious	mod
SuperPath	DAA	-0.66(-1.52, 0.21)	PA	mod	no	mod	-0.66(-1.52, 0.21)	NR	mod	no	serious	low
SuperPath	MIS-PA						-0.69(-1.54, 0.15)	0.3701	high	no	serious	mod

© 2023 Yan L et al. *JAMA Network Open.*

SuperPath	MIS-ALA	-1.12(-2.25, 0.01)	PA, DAA	mod	no	mod	-1.12(-2.25, 0.01)	NR	mod	no	serious	low
SuperPath	DLA	-1.16(-2.20, -0.13)	PA	high	no	high	-1.16(-2.20, -0.13)	NR	high	no	no	high
MIS-DLA	PA						-0.23(-0.98, 0.52)	0.3844	high	no	serious	mod
MIS-DLA	DAA	-0.22(-1.19, 0.75)	PA	mod	no	mod	-0.39(-1.06, 0.27)	0.6224	mod	no	serious	low
MIS-DLA	MIS-PA						-0.43(-1.20, 0.34)	0.738	high	no	serious	mod
MIS-DLA	MIS-ALA	-0.64(-1.77, 0.49)	DAA	mod	no	mod	-0.86(-1.77, 0.04)	0.5188	mod	no	serious	low
MIS-DLA	DLA	-0.90(-1.76, -0.04)	MIS-ALA	mod	no	mod	-0.90(-1.76, -0.04)	NR	mod	no	no	mod

© 2023 Yan L et al. *JAMA Network Open.*

PA	DAA	-0.38(-0.44, 1.2)	MIS-PA	mod	no	mod	-0.16(-0.73, 0.40)	0.4782	mod	no	serious	low
PA	MIS-PA						-0.20(-0.80, 0.40)	0.4828	high	no	serious	mod
PA	MIS-ALA	-0.63(-1.54, 0.28)	DAA	mod	no	mod	-0.63(-1.54, 0.28)	NR	mod	no	serious	low
PA	DLA						-0.67(-1.46, 0.12)	0.6672	high	no	serious	mod
DAA	MIS-PA	0.09(-0.8, 0.97)	PA	mod	no	mod	-0.04(-0.63, 0.56)	0.7126	mod	no	serious	low
DAA	MIS-ALA						-0.47(-1.25, 0.32)	0.5685	high	no	serious	mod
DAA	DLA	-0.65(-1.76, 0.46)	MIS-ALA	high	no	high	-0.51(-1.15, 0.14)	0.7518	high	no	serious	mod

© 2023 Yan L et al. *JAMA Network Open.*

MIS-PA	MIS-ALA	-0.43(-1.38, 0.52)	MIS-DLA	mod	no	mod	-0.43(-1.38, 0.52)	NR	mod	no	serious	low
MIS-PA	DLA	-0.47(-1.31, 0.37)	PA	high	no	high	-0.47(-1.31, 0.37)	NR	high	no	serious	mod
MIS-ALA	DLA						-0.04(-0.77, 0.69)	0.9919	high	no	serious	mod

Outcome : Hospitalization time

**Comparison groups**		**Direct evidence**								
Arm 1	Arm 2	MD (95%CrI)	RoB	Inconsistency	Indirectness	Publicaion bias	Direct rating without imprecision	Need to rate indiret?	Imprecision	Direct rating with imprecision
SuperPath	DAA									
SuperPath	PA	-2.13 (-3.33, -0.94)	no	no	no	Undected	high	no	no	high
SuperPath	MIS-DLA									

© 2023 Yan L et al. *JAMA Network Open.*

Comparison groups		Direct evidence								
Arm 1	Arm 2	MD (95%CrI)	RoB	Inconsistency	Indirectness	Publicaion bias	Direct rating without imprecision	Need to rate indiret?	Imprecision	Direct rating with imprecision
SuperPath	MIS-ALA									
SuperPath	DLA									
SuperPath	2-incision									
SuperPath	MIS-PA	0.19 (-1.92, 2.30)	no	no	no	Undected	high	no	serious	mod
DAA	PA	-0.47 (-1.25, 0.31)	no	no	no	Undected	high	no	serious	mod
DAA	MIS-DLA	-0.82 (-2.14, 0.51)	serious	serious	no	Undected	low	yes	serious	V low
DAA	MIS-ALA	-1.00 (-3.93, 1.93)	no	no	no	Undected	high	no	serious	mod
DAA	DLA	-0.34 (-1.32, 0.64)	no	no	no	Undected	high	no	serious	mod
DAA	2-incision									
DAA	MIS-PA	0.07 (-1.08, 1.22)	serious	no	no	Undected	mod	yes	serious	low

© 2023 Yan L et al. *JAMA Network Open.*

Comparison groups		Direct evidence								
Arm 1	Arm 2	MD (95%CrI)	RoB	Inconsistency	Indirectness	Publicaion bias	Direct rating without imprecision	Need to rate indiret?	Imprecision	Direct rating with imprecision
PA	MIS-DLA									
PA	MIS-ALA									
PA	DLA									
PA	2-incision									
PA	MIS-PA	-2.25 (-3.17, -1.32)	no	serious	no	Undected	mod	yes	no	mod
MIS-DLA	MIS-ALA	0.03 (-1.85, 1.91)	no	no	no	Undected	high	no	serious	mod
MIS-DLA	DLA	-0.90 (-2.21, 0.40)	serious	no	no	Undected	mod	yes	serious	low
MIS-DLA	2-incision									
MIS-DLA	MIS-PA									
MIS-ALA	DLA	0.20 (-1.90, 2.30)	no	no	no	Undected	high	no	serious	mod
MIS-ALA	2-incision	-0.70 (-2.96, 1.56)	no	no	no	Undected	high	no	serious	mod

© 2023 Yan L et al. *JAMA Network Open.*

Comparison groups		Direct evidence								
Arm 1	Arm 2	MD (95%CrI)	RoB	Inconsistency	Indirectness	Publicaion bias	Direct rating without imprecision	Need to rate indiret?	Imprecision	Direct rating with imprecision
MIS-ALA	MIS-PA									
DLA	2-incision									
DLA	MIS-PA									
2-incision	MIS-PA	-0.09 (-1.45, 1.28)	no	no	no	Undected	high	no	serious	mod

© 2023 Yan L et al. *JAMA Network Open.*

Comparison groups		Indirect evidence					Network evidence					
Arm 1	Arm 2	MD (95%CrI)	First order of the most contribution	Lowest of C1 and C2	Intransitivity	Indirect rating without imprecision	MD (95%CrI)	Incoherence, P-value	Higher rating of direct and indirect without imprecision	Incoherence	NMA imprecision	Network rating with imprecision
Super Path	DAA	-1.33 (-2.54, -0.11)	PA	mod	no	mod	-1.33 (-2.54, -0.11)	NA	mod	no	no	mod
Super Path	PA						-1.31 (-2.36, -0.25)	0.0041	high	serious	no	mod
Super Path	MIS-DLA	-1.56 (-3.11, -0.01)	PA, DAA	low	no	low	-1.56 (-3.11, -0.01)	NA	low	no	no	low
Super Path	MIS-ALA	-1.88 (-3.62, -0.13)	PA, DAA	mod	no	mod	-1.88 (-3.62, -0.13)	NA	mod	no	no	mod
Super Path	DLA	-1.92 (-3.39, -0.45)	PA, DAA	mod	no	mod	-1.92 (-3.39, -0.45)	NA	mod	no	no	mod
Super Path	2-incision	-2.37 (-4.04, -0.70)	MIS-PA	high	no	high	-2.37 (-4.04, -0.70)	NA	high	no	no	high
Super Path	MIS-PA						-2.38 (-3.55, -1.20)	0.0041	high	serious	no	mod

© 2023 Yan L et al. *JAMA Network Open.*

Comparison groups		Indirect evidence					Network evidence					
Arm 1	Arm 2	MD (95%CrI)	First order of the most contribution	Lowest of C1 and C2	Intransitivity	Indirect rating without imprecision	MD (95%CrI)	Incoherence, P-value	Higher rating of direct and indirect without imprecision	Incoherence	NMA imprecision	Network rating with imprecision
DAA	PA						0.02 (-0.66, 0.70)	0.0125	high	serious	serious	low
DAA	MIS-DLA	0.49 (-0.99, 1.97)	DLA	mod	no	mod	-0.24 (-1.22, 0.75)	NA	mod	no	serious	low
DAA	MIS-ALA						-0.55 (-1.87, 0.76)	0.7387	high	no	serious	mod
DAA	DLA						-0.59 (-1.43, 0.24)	0.3311	high	no	serious	mod
DAA	2-incision	-1.04 (-2.41, 0.33)	MIS-PA	mod	no	mod	-1.04 (-2.41, 0.33)	NA	mod	no	serious	low
DAA	MIS-PA	-2.05 (-3.13, -0.96)	PA	mod	no	mod	-1.05 (-1.84, -0.26)	0.0087	mod	serious	no	low
PA	MIS-DLA	-0.26 (-1.44, 0.93)	DAA	low	no	low	-0.26 (-1.44, 0.93)	0.1968	low	no	serious	V low
PA	MIS-ALA	-0.57 (-2.01, 0.86)	DAA	high	no	high	-0.57 (-2.01, 0.86)	NA	high	no	serious	mod

© 2023 Yan L et al. *JAMA Network Open.*

Comparison groups		Indirect evidence					Network evidence					
Arm 1	Arm 2	MD (95%CrI)	First order of the most contribution	Lowest of C1 and C2	Intransitivity	Indirect rating without imprecision	MD (95%CrI)	Incoherence, P-value	Higher rating of direct and indirect without imprecision	Incoherence	NMA imprecision	Network rating with imprecision
PA	DLA	-0.61 (-1.68, 0.45)	DAA	mod	no	mod	-0.61 (-1.68, 0.45)	NA	mod	no	serious	low
PA	2-incision	-1.06 (-2.44, 0.32)	MIS-PA	mod	no	mod	-1.06 (-2.44, 0.32)	NA	mod	serious	serious	V low
PA	MIS-PA	0.81 (-0.36, 1.97)	DAA	mod	no	mod	-1.07 (-1.79, -0.35)	<0.0001	mod	no	no	mod
MIS-DLA	MIS-ALA						-0.32 (-1.65, 1.01)	0.6093	high	no	serious	mod
MIS-DLA	DLA	0.31 (-1.14, 1.76)	DAA	low	no	low	-0.36 (-1.33, 0.61)	0.2229	mod	no	serious	low
MIS-DLA	2-incision	-0.81 (-2.40, 0.79)	MIS-ALA	high	no	high	-0.81 (-2.40, 0.79)	NA	high	no	serious	mod
MIS-DLA	MIS-PA	-0.81 (-2.04, 0.42)	DAA	low	no	low	-0.81 (-2.04, 0.42)	NA	low	no	serious	V low

© 2023 Yan L et al. *JAMA Network Open.*

Comparison groups		Indirect evidence					Network evidence					
Arm 1	Arm 2	MD (95%CrI)	First order of the most contribution	Lowest of C1 and C2	Intransitivity	Indirect rating without imprecision	MD (95%CrI)	Incoherence, P-value	Higher rating of direct and indirect without imprecision	Incoherence	NMA imprecision	Network rating with imprecision
MIS-ALA	DLA						-0.04 (-1.35, 1.27)	0.7741	high	no	serious	mod
MIS-ALA	2-incision						-0.49 (-2.06, 1.08)	0.798	high	no	serious	mod
MIS-ALA	MIS-PA	-0.50 (-1.93, 0.94)	DAA	mod	no	mod	-0.50 (-1.93, 0.94)	NA	mod	no	serious	low
DLA	2-incision	-0.45 (-1.98, 1.08)	MIS-ALA	high	no	high	-0.45 (-1.98, 1.08)	NA	high	no	serious	mod
DLA	MIS-PA	-0.46 (-1.58, 0.67)	DAA	mod	no	mod	-0.46 (-1.58, 0.67)	NA	mod	no	serious	low
2-incision	MIS-PA						-0.01 (-1.24, 1.22)	0.798	high	no	serious	mod

© 2023 Yan L et al. *JAMA Network Open.*

Outcome : Operation time

Comparison groups		Direct evidence									
Arm 1	Arm 2	MD (95%CrI)	RoB	Inconsistency	Indirectness	Publicaion bias	Direct rating without imprecision	Need to rate indiret?	Imprecision	Direct rating with imprecision
PA	MIS-PA	0.98(-5.88, 7.84)	no	no	no	no	high	no	serious	mod
PA	MIS-DLA	-12.62(-24.76, -0.47)	serious	no	no	no	mod	yes	no	mod
PA	MIS-ALA	-1.20(-12.05, 9.66)	serious	no	no	no	mod	yes	serious	low
PA	DLA	-7.46(-17.43, 2.51)	no	serious	no	no	mod	yes	serious	low
PA	SuperPath	-17.09(-25.91, -8.26)	no	no	no	no	high	no	no	high
PA	DAA	-18.90(-25.36, -12.44)	no	no	no	no	high	no	no	high

© 2023 Yan L et al. *JAMA Network Open.*

Comparison groups		Direct evidence								
Arm 1	Arm 2	MD (95%CrI)	RoB	Inconsistency	Indirectness	Publicaion bias	Direct rating without imprecision	Need to rate indiret?	Imprecision	Direct rating with imprecision
PA	2-incision									
MIS-PA	MIS-DLA	-7.47(-19.52, 4.59)	no	no	no	no	high	no	serious	mod
MIS-PA	MIS-ALA	-7.30(-23.55, 8.95)	serious	no	no	no	mod	yes	serious	low
MIS-PA	DLA	-3.20(-24.52, 18.12)	serious	no	no	no	mod	yes	serious	low
MIS-PA	SuperPath	-1.50(-17.32, 14.32)	no	no	no	no	high	no	serious	mod
MIS-PA	DAA	-13.76(-24.74, -2.78)	serious	no	no	no	mod	yes	no	mod
MIS-PA	2-incision	-22.27(-34.64, -9.91)	no	no	no	no	high	no	no	high

© 2023 Yan L et al. *JAMA Network Open.*

**Comparison groups**		**Direct evidence**								
Arm 1	Arm 2	MD (95%CrI)	RoB	Inconsistency	Indirectness	Publicaion bias	Direct rating without imprecision	Need to rate indiret?	Imprecision	Direct rating with imprecision
MIS-DLA	MIS-ALA	-1.66(-11.00, 7.69)	serious	no	no	no	mod	yes	serious	low
MIS-DLA	DLA	-8.76(-16.54, -0.97)	serious	no	no	no	mod	yes	no	mod
MIS-DLA	SuperPath									
MIS-DLA	DAA	-8.80(-20.49, 2.88)	serious	no	no	no	mod	yes	serious	low
MIS-DLA	2-incision									
MIS-ALA	DLA	-0.27(-10.23, 9.69)	serious	no	no	no	mod	yes	serious	low
MIS-ALA	SuperPath									
MIS-ALA	DAA	-3.69(-15.48, 8.10)	no	serious	no	no	mod	yes	serious	low

© 2023 Yan L et al. *JAMA Network Open.*

Comparison groups		Direct evidence								
Arm 1	Arm 2	MD (95%CrI)	RoB	Inconsistency	Indirectness	Publicaion bias	Direct rating without imprecision	Need to rate indiret?	Imprecision	Direct rating with imprecision
MIS-ALA	2-incision	-13.00(-46.03, 20.03)	serious	no	no	no	mod	yes	serious	low
DLA	SuperPath									
DLA	DAA	0.20(-6.51, 6.92)	no	no	no	no	high	no	serious	mod
DLA	2-incision									
SuperPath	DAA									
SuperPath	2-incision									
DAA	2-incision									

© 2023 Yan L et al. *JAMA Network Open.*

Comparison groups		Indirect evidence					Network evidence					
Arm 1	Arm 2	MD (95%CrI)	First order of the most contribution	Lowest of C1 and C2	Intransitivity	Indirect rating without imprecision	MD (95% CrI)	Incoherence, P-value	Higher rating of direct and indirect without imprecision	Incoherence	NMA imprecision	Network rating with imprecision
PA	MIS-PA						-1.08(-6.29, 4.14)	<0.0001	high	serious	serious	low
PA	MIS-DLA	-3.72(-10.68, 3.23)	MIS-PA	mod	no	high	-5.92(-11.95, 0.11)	NR	high	no	serious	mod
PA	MIS-ALA	-11.4(-18.63, -4.17)	DAA	mod	no	mod	-8.26(-14.28, -2.24)	NR	mod	no	no	mod
PA	DLA	-15.3(-21.88, -8.71)	DAA	high	no	high	-12.92(-	NR	high	no	no	high

© 2023 Yan L et al. *JAMA Network Open.*

Comparison groups		Indirect evidence					Network evidence					
Arm 1	Arm 2	MD (95%CrI)	First order of the most contribution	Lowest of C1 and C2	Intransitivity	Indirect rating without imprecision	MD (95% CrI)	Incoherence, P-value	Higher rating of direct and indirect without imprecision	Incoherence	NMA imprecision	Network rating with imprecision
							18.41, -7.42)					
PA	SuperPath						-13.64(-21.45, -5.84)	0.0041	high	serious	no	mod
PA	DAA						-15.00(-19.76, -10.24)	0.0125	high	serious	no	mod
PA	2-incision	-23.10(-35.64, -10.55)	MIS-PA	high	no	high	-23.10(-35.64,	NR	high	no	no	high

© 2023 Yan L et al. *JAMA Network Open.*

Comparison groups		Indirect evidence					Network evidence					
Arm 1	Arm 2	MD (95%CrI)	First order of the most contribution	Lowest of C1 and C2	Intransitivity	Indirect rating without imprecision	MD (95% CrI)	Incoherence, P-value	Higher rating of direct and indirect without imprecision	Incoherence	NMA imprecision	Network rating with imprecision
							-10.55)					
MIS-PA	MIS-DLA						-4.84(-11.54, 1.86)	NR	high	no	serious	mod
MIS-PA	MIS-ALA	-7.16(-14.63, 0.32)	PA	mod	no	mod	-7.18(-13.98, -0.39)	NR	mod	no	no	mod
MIS-PA	DLA	-12.73(-19.59, -5.88)	DAA	mod	no	mod	-11.84(-18.36, -5.31)	NR	mod	no	no	mod
MIS-PA	SuperPath						-12.57(	0.0041	high	serious	no	mod

© 2023 Yan L et al. *JAMA Network Open.*

Comparison groups		Indirect evidence					Network evidence					
Arm 1	Arm 2	MD (95%CrI)	First order of the most contribution	Lowest of C1 and C2	Intransitivity	Indirect rating without imprecision	MD (95% CrI)	Incoherence, P-value	Higher rating of direct and indirect without imprecision	Incoherence	NMA imprecision	Network rating with imprecision
							-21.24, -3.89)					
MIS-PA	DAA	-13.99(-20.99, -6.98)	PA	high	no	high	-13.92(-19.83, -8.01)	0.0087	high	serious	no	mod
MIS-PA	2-incision						-22.02(-33.63, -10.41)	0.789	high	no	no	high
MIS-DLA	MIS-ALA	-2.89(-11.24, 5.46)	DLA	mod	no	mod	-2.34(-	0.6093	mod	no	serious	low

© 2023 Yan L et al. *JAMA Network Open.*

Comparison groups		Indirect evidence					Network evidence					
Arm 1	Arm 2	MD (95%CrI)	First order of the most contribution	Lowest of C1 and C2	Intransitivity	Indirect rating without imprecision	MD (95% CrI)	Incoherence, P-value	Higher rating of direct and indirect without imprecision	Incoherence	NMA imprecision	Network rating with imprecision
							8.57, 3.88)					
MIS-DLA	DLA	-5.06(-13.24, 3.12)	MIS-ALA	mod	no	mod	-7.00(-12.64, -1.36)	0.2229	mod	no	no	mod
MIS-DLA	SuperPath	-7.72(-17.36, 1.91)	MIS-PA	high	no	high	-7.72(-17.36, 1.91)	NR	high	no	serious	mod
MIS-DLA	DAA	-9.18(-16.10, -2.25)	DLA	mod	no	mod	-9.08(-15.04, -3.12)	0.1968	mod	no	no	mod
MIS-DLA	2-incision	-17.18(-30.34, -4.01)	MIS-PA	high	no	high	-17.18(-	NR	high	no	no	high

© 2023 Yan L et al. *JAMA Network Open.*

Comparison groups		Indirect evidence					Network evidence					
Arm 1	Arm 2	MD (95%CrI)	First order of the most contribution	Lowest of C1 and C2	Intransitivity	Indirect rating without imprecision	MD (95% CrI)	Incoherence, P-value	Higher rating of direct and indirect without imprecision	Incoherence	NMA imprecision	Network rating with imprecision
							30.34, -4.01)					
MIS-ALA	DLA	-7.14(-14.65, 0.36)	DAA	mod	no	mod	-4.66(-10.65, 1.34)	0.7741	mod	no	serious	low
MIS-ALA	SuperPath	-5.38(-15.03, 4.26)	MIS-PA	mod	no	mod	-5.38(-15.03, 4.26)	NR	mod	no	serious	low
MIS-ALA	DAA	-7.84(-14.93, -0.74)	PA	mod	no	mod	-6.74(-12.82, -0.66)	0.7378	mod	no	no	mod
MIS-ALA	2-incision	-15.17(-29.34, -1.00)	MIS-PA	mod	no	mod	-14.83(-	0.789	mod	no	no	mod

© 2023 Yan L et al. *JAMA Network Open.*

Comparison groups		Indirect evidence					Network evidence					
Arm 1	Arm 2	MD (95%CrI)	First order of the most contribution	Lowest of C1 and C2	Intransitivity	Indirect rating without imprecision	MD (95% CrI)	Incoherence, P-value	Higher rating of direct and indirect without imprecision	Incoherence	NMA imprecision	Network rating with imprecision
							27.85, -1.81)					
DLA	SuperPath	-0.73(-10.08, 8.63)	MIS-PA	mod	no	mod	-0.73(-10.08, 8.63)	NR	mod	no	serious	low
DLA	DAA						-2.08(-7.08, 2.92)	0.3311	high	no	serious	mod
DLA	2-incision	-10.18(-23.25, 2.89)	MIS-PA	mod	no	mod	-10.18(-23.25, 2.89)	NR	mod	no	serious	low
SuperPath	DAA	-1.36(-10.30, 7.59)	MIS-PA	mod	no	mod	-1.36(-	NR	mod	no	serious	low

© 2023 Yan L et al. *JAMA Network Open.*

Comparison groups		Indirect evidence					Network evidence					
Arm 1	Arm 2	MD (95%CrI)	First order of the most contribution	Lowest of C1 and C2	Intransitivity	Indirect rating without imprecision	MD (95% CrI)	Incoherence, P-value	Higher rating of direct and indirect without imprecision	Incoherence	NMA imprecision	Network rating with imprecision
							10.30, 7.59)					
SuperPath	2-incision	-9.45(-23.82, 4.92)	MIS-PA	high	no	high	-9.45(-23.82, 4.92)	NR	high	no	serious	mod
DAA	2-incision	-8.10(-20.91, 4.72)	MIS-PA	mod	no	mod	-8.10(-20.91, 4.72)	NR	mod	no	serious	low

© 2023 Yan L et al. *JAMA Network Open.*

Outcome : Blood loss

Comparison groups		Direct evidence								
Arm 1	Arm 2	MD (95%CrI)	RoB	Inconsistency	Indirectness	Publicaion bias	Direct rating without imprecision	Need to rate indiret ?	Imprecision	Direct rating with imprecision
MIS-ALA	MIS-DLA	30.82 (-109.68, 171.32)	serious	no	no	no	mod	yes	serious	low
MIS-ALA	MIS-PA	-79.00 (-282.78, 124.78)	no	no	no	no	high	no	serious	mod
MIS-ALA	SuperPath									
MIS-ALA	PA	-120.65 (-224.67, -16.64)	no	no	no	no	high	no	no	high
MIS-ALA	2-incision	-135.00 (-362.50, 92.50)	serious	no	no	no	mod	yes	serious	low
MIS-ALA	DAA	-15.16 (-232.09, 201.78)	no	no	no	no	high	no	serious	mod
MIS-ALA	DLA	-85.33 (-215.73, 45.07)	no	no	no	no	high	no	serious	mod
MIS-DLA	MIS-PA	8.10 (-108.54, 124.74)	no	no	no	no	high	no	serious	mod
MIS-DLA	SuperPath									

© 2023 Yan L et al. *JAMA Network Open.*

Comparison groups		Direct evidence								
Arm 1	Arm 2	MD (95%CrI)	RoB	Inconsistency	Indirectness	Publicaion bias	Direct rating without imprecision	Need to rate indiret?	Imprecision	Direct rating with imprecision
MIS-DLA	PA	1.00 (-131.90, 133.90)	no	no	no	no	high	no	serious	mod
MIS-DLA	2-incision									
MIS-DLA	DAA	-9.91 (-127.29, 107.47)	no	no	no	no	high	no	serious	mod
MIS-DLA	DLA	-236.72 (-335.28, -138.15)	serious	no	no	no	mod	yes	no	mod
MIS-PA	SuperPath	26.66 (-91.37, 144.69)	no	no	no	no	high	no	serious	mod
MIS-PA	PA	-58.67 (-111.48, -5.86)	no	no	no	no	high	no	no	high
MIS-PA	2-incision	-46.00 (-193.93, 101.93)	no	no	no	no	high	no	serious	mod
MIS-PA	DAA									
MIS-PA	DLA									
SuperPath	PA	-26.77 (-99.68, 46.14)	no	no	no	no	high	no	serious	mod
SuperPath	2-incision									

© 2023 Yan L et al. *JAMA Network Open.*

Comparison groups		Direct evidence								
Arm 1	Arm 2	MD (95%CrI)	RoB	Inconsistency	Indirectness	Publicaion bias	Direct rating without imprecision	Need to rate indiret?	Imprecision	Direct rating with imprecision
SuperPath	DAA									
SuperPath	DLA									
PA	2-incision									
PA	DAA	-45.53 (-117.30, 26.23)	no	no	no	no	high	no	serious	mod
PA	DLA	8.00 (-144.64, 160.64)	no	no	no	no	high	no	serious	mod
2-incision	DAA									
2-incision	DLA									
DAA	DLA	-67.83 (-130.03, -5.63)	no	no	no	no	high	no	no	high

© 2023 Yan L et al. *JAMA Network Open.*

Comparison groups		Indirect evidence					Network evidence					
Arm 1	Arm 2	MD (95%CrI)	First order of the most contribution	Lowest of C1 and C2	Intransitivity	Indirect rating without imprecision	MD (95%CrI)	Incoherence, P-value	Higher rating of direct and indirect without imprecision	Incoherence	NMA imprecision	Network rating with imprecision
MIS-ALA	MIS-DLA	-20.1(113.89,73.86)	DAA,DLA	mod	no	mod	-4.32 (-82.38, 73.73)	0.5554	mod	no	serious	low
MIS-ALA	MIS-PA						-16.17 (-95.09, 62.76)	0.5121	high	no	serious	mod
MIS-ALA	Super Path	-28.72 (-122.58, 65.14)	MIS-PA	high	no	high	-28.72 (-122.58, 65.14)	NR	high	no	serious	mod
MIS-ALA	PA						-70.45 (-141.61, 0.71)	0.1946	high	no	serious	mod
MIS-ALA	2-incision	-55.44(-224.81,113.94)	MIS-DLA,MIS-PA	high	no	high	-83.81 (-219.67, 52.05)	0.5824	high	no	serious	mod

© 2023 Yan L et al. *JAMA Network Open.*

Comparison groups		Indirect evidence					Network evidence					
Arm 1	Arm 2	MD (95%CrI)	First order of the most contribution	Lowest of C1 and C2	Intransitivity	Indirect rating without imprecision	MD (95%CrI)	Incoherence, P-value	Higher rating of direct and indirect without imprecision	Incoherence	NMA imprecision	Network rating with imprecision
MIS-ALA	DAA						-85.10 (-161.90, -8.31)	0.4992	high	no	no	high
MIS-ALA	DLA						-155.54 (-232.09, -78.99)	0.1923	high	no	no	high
MIS-DLA	MIS-PA						-11.84 (-81.29, 57.61)	0.6766	high	no	serious	mod
MIS-DLA	SuperPath	-24.40 (-112.05, 63.26)	MIS-PA	high	no	high	-24.40 (-112.05, 63.26)	NR	high	no	serious	mod
MIS-DLA	PA						-66.13 (-129.72, -2.54)	0.2596	high	no	no	high
MIS-DLA	2-incision	-79.49 (-218.34, 59.37)	MIS-PA	high	no	high	-79.49 (-218.34, 59.37)	NR	high	no	serious	mod

© 2023 Yan L et al. *JAMA Network Open.*

Comparison groups		Indirect evidence					Network evidence					
Arm 1	Arm 2	MD (95%CrI)	First order of the most contribution	Lowest of C1 and C2	Intransitivity	Indirect rating without imprecision	MD (95%CrI)	Incoherence, P-value	Higher rating of direct and indirect without imprecision	Incoherence	NMA imprecision	Network rating with imprecision
MIS-DLA	DAA						-80.78 (-145.46, -16.10)	0.1561	high	no	no	high
MIS-DLA	DLA	-85.33(-171.86,1.20)	DAA	high	no	high	-151.22 (-216.25, -86.19)	0.0237	high	serious	no	mod
MIS-PA	Super Path						-12.56 (-82.96, 57.85)	0.4171	high	no	serious	mod
MIS-PA	PA						-54.29 (-100.31, -8.26)	0.7403	high	no	no	high
MIS-PA	2-incision						-67.64 (-193.86, 58.57)	0.5824	high	no	serious	mod
MIS-PA	DAA	-68.94 (-137.51, -0.37)	PA	high	no	high	-68.94 (-137.51, -0.37)	NR	high	no	no	high

© 2023 Yan L et al. *JAMA Network Open.*

Comparison groups		Indirect evidence					Network evidence					
Arm 1	Arm 2	MD (95%CrI)	First order of the most contribution	Lowest of C1 and C2	Intransitivity	Indirect rating without imprecision	MD (95%CrI)	Incoherence, P-value	Higher rating of direct and indirect without imprecision	Incoherence	NMA imprecision	Network rating with imprecision
MIS-PA	DLA	-139.38 (-213.05, -65.70)	DAA,PA	high	no	high	-139.38 (-213.05, -65.70)	NR	high	no	no	high
Super Path	PA						-41.73 (-105.05, 21.59)	0.4171	high	no	serious	mod
Super Path	2-incision	-55.09 (-197.15, 86.97)	MIS-PA	high	no	high	-55.09 (-197.15, 86.97)	NR	high	no	serious	mod
Super Path	DAA	-56.38 (-140.02, 27.25)	PA,MIS-PA	high	no	high	-56.38 (-140.02, 27.25)	NR	high	no	serious	mod
Super Path	DLA	-126.82 (-215.40, -38.24)	PA,MIS-PA	high	no	high	-126.82 (-215.40, -38.24)	NR	high	no	no	high

© 2023 Yan L et al. *JAMA Network Open.*

Comparison groups		Indirect evidence					Network evidence					
Arm 1	Arm 2	MD (95%CrI)	First order of the most contribution	Lowest of C1 and C2	Intransitivity	Indirect rating without imprecision	MD (95%CrI)	Incoherence, P-value	Higher rating of direct and indirect without imprecision	Incoherence	NMA imprecision	Network rating with imprecision
PA	2-incision	-13.36 (-144.02, 117.31)	MIS-PA	high	no	high	-13.36 (-144.02, 117.31)	NR	high	no	serious	mod
PA	DAA						-14.65 (-70.68, 41.38)	0.177	high	no	serious	mod
PA	DLA						-85.09 (-148.64, -21.54)	0.1886	high	no	no	high
2-incision	DAA	-1.29 (-139.63, 137.04)	PA,MIS-PA	high	no	high	-1.29 (-139.63, 137.04)	NR	high	no	serious	mod
2-incision	DLA	-71.73 (-211.86, 68.39)	MIS-PA	high	no	high	-71.73 (-211.86, 68.39)	NR	high	no	serious	mod

© 2023 Yan L et al. *JAMA Network Open.*

Comparison groups		Indirect evidence					Network evidence					
Arm 1	Arm 2	MD (95%CrI)	First order of the most contribution	Lowest of C1 and C2	Intransitivity	Indirect rating without imprecision	MD (95%CrI)	Incoherence, P-value	Higher rating of direct and indirect without imprecision	Incoherence	NMA imprecision	Network rating with imprecision
DAA	DLA						-70.44 (-122.54, -18.34)	0.8802	high	no	no	high

© 2023 Yan L et al. *JAMA Network Open.*

Outcome : quality of life score change

Comparison groups		Direct evidence								
Arm 1	Arm 2	MD (95%CrI)	RoB	Inconsistency	Indirectness	Publicaion bias	Direct rating without imprecision	Need to rate indiret?	Imprecision	Direct rating with imprecision
MIS-DLA	SuperPath									
MIS-DLA	MIS-ALA									
MIS-DLA	PA	-0.17(-1.08,0.75)	no	no	no	no	high	no	serious	mod
MIS-DLA	MIS-PA									
MIS-DLA	DAA	1.07(0.42,1.72)	serious	no	no	no	mod	yes	no	mod
MIS-DLA	DLA									
MIS-DLA	2-incision									
SuperPath	MIS-ALA									
SuperPath	PA	-0.36(-1.24,0.53)	no	no	no	no	high	no	serious	mod

© 2023 Yan L et al. *JAMA Network Open.*

Comparison groups		Direct evidence								
Arm 1	Arm 2	MD (95%CrI)	RoB	Inconsistency	Indirectness	Publicaion bias	Direct rating without imprecision	Need to rate indiret?	Imprecision	Direct rating with imprecision
SuperPath	MIS-PA	1.04(0.04,2.05)	no	no	no	no	high	no	no	high
SuperPath	DAA									
SuperPath	DLA									
SuperPath	2-incision									
MIS-ALA	PA	0.27(-0.37,0.92)	no	no	no	no	high	no	serious	mod
MIS-ALA	MIS-PA	0.28(-0.67,1.24)	no	no	no	no	high	no	serious	mod
MIS-ALA	DAA									
MIS-ALA	DLA	0.19(-0.49,0.86)	serious	no	no	no	mod	yes	serious	low
MIS-ALA	2-incision									
PA	MIS-PA	-0.15(-0.67,0.37)	no	no	no	no	high	no	serious	mod

© 2023 Yan L et al. *JAMA Network Open.*

Comparison groups		Direct evidence								
Arm 1	Arm 2	MD (95%CrI)	RoB	Inconsistency	Indirectness	Publicaion bias	Direct rating without imprecision	Need to rate indiret?	Imprecision	Direct rating with imprecision
PA	DAA	0.38(-0.53,1.29)	no	no	no	no	high	no	serious	mod
PA	DLA	0.18(-0.76,1.11)	no	no	no	no	high	no	serious	mod
PA	2-incision									
MIS-PA	DAA	-0.18(-0.83,0.46)	serious	no	no	no	mod	yes	serious	low
MIS-PA	DLA									
MIS-PA	2-incision	0.21(-0.43,0.85)	no	no	no	no	high	no	serious	mod
DAA	DLA	0.09(-0.34,0.52)	no	no	no	no	high	no	serious	mod
DAA	2-incision									
DLA	2-incision									

© 2023 Yan L et al. *JAMA Network Open.*

Comparison groups		Indirect evidence					Network evidence					
Arm 1	Arm 2	MD (95%CrI)	First order of the most contribution	Lowest of C1 and C2	Intransitivity	Indirect rating without imprecision	MD (95%CrI)	Incoherence, P-value	Higher rating of direct and indirect without imprecision	Incoherence	NMA imprecision	Network rating with imprecision
MIS-DLA	SuperPath	0.33 (-0.56,1.23)	PA	high	no	high	0.33 (-0.56,1.23)	NR	high	no	serious	mod
MIS-DLA	MIS-ALA	0.42(-0.31,1.15)	PA	high	no	high	0.42(-0.31,1.15)	NR	high	no	serious	mod
MIS-DLA	PA						0.49(-0.11,1.10)	0.0596	high	no	serious	mod
MIS-DLA	MIS-PA	0.70(0.04, 1.37)	PA	high	no	high	0.70(0.04, 1.37)	NR	high	no	no	high
MIS-DLA	DAA	-1.01(-1.14,0.93)	PA	high	no	high	0.74(0.19, 1.29)	0.0596	high	no	no	high
MIS-DLA	DLA	0.75(0.11, 1.39)	PA	high	no	high	0.75(0.11, 1.39)	NR	high	no	no	high
MIS-DLA	2-incision	0.92(-0.01,1.84)	PA, MIS-PA	high	no	high	0.92(-0.01,1.84)	NR	high	no	serious	mod

© 2023 Yan L et al. *JAMA Network Open.*

Comparison groups		Indirect evidence					Network evidence					
Arm 1	Arm 2	MD (95%CrI)	First order of the most contribution	Lowest of C1 and C2	Intransitivity	Indirect rating without imprecision	MD (95%CrI)	Incoherence, P-value	Higher rating of direct and indirect without imprecision	Incoherence	NMA imprecision	Network rating with imprecision
Super Path	MIS-ALA	0.09(-0.74,0.92)	PA	high	no	high	0.09(-0.74,0.92)	NR	high	no	serious	mod
Super Path	PA						0.16(-0.53,0.85)	0.0674	high	no	serious	mod
Super Path	MIS-PA						0.37(-0.33,1.08)	0.0674	high	no	serious	mod
Super Path	DAA	0.41(-0.37,1.19)	PA	high	no	high	0.41(-0.37,1.19)	NR	high	no	serious	mod
Super Path	DLA	0.42(-0.39,1.23)	PA, DAA	high	no	high	0.42(-0.39,1.23)	NR	high	no	serious	mod
Super Path	2-incision	0.58(-0.37,1.54)	PA, MIS-PA	high	no	high	0.58(-0.37,1.54)	NR	high	no	serious	mod
MIS-ALA	PA						0.07(-0.43,0.58)	0.3209	high	no	serious	mod
MIS-ALA	MIS-PA						0.29(-0.29,0.86)	0.9957	high	no	serious	mod

© 2023 Yan L et al. *JAMA Network Open.*

Comparison groups		Indirect evidence					Network evidence					
Arm 1	Arm 2	MD (95%CrI)	First order of the most contribution	Lowest of C1 and C2	Intransitivity	Indirect rating without imprecision	MD (95%CrI)	Incoherence, P-value	Higher rating of direct and indirect without imprecision	Incoherence	NMA imprecision	Network rating with imprecision
MIS-ALA	DAA	0.32(-0.24,0.88)	DLA	mod	no	mod	0.32(-0.24,0.88)	NR	mod	no	serious	low
MIS-ALA	DLA	0.54(-0.26,1.34)	PA, DAA	high	no	high	0.33(-0.18,0.85)	0.5127	high	no	serious	mod
MIS-ALA	2-incision	0.50(-0.37,1.36)	PA, MIS-PA	high	no	high	0.50(-0.37,1.36)	NR	high	no	serious	mod
PA	MIS-PA						0.21(-0.20,0.63)	0.022	high	serious	serious	low
PA	DAA						0.25(-0.20,0.69)	0.7502	high	no	serious	mod
PA	DLA						0.26(-0.22,0.74)	0.8374	high	no	serious	mod
PA	2-incision	0.42(-0.34,1.19)	MIS-PA	high	no	high	0.42(-0.34,1.19)	NR	high	no	serious	mod

© 2023 Yan L et al. *JAMA Network Open.*

Comparison groups		Indirect evidence					Network evidence					
Arm 1	Arm 2	MD (95%CrI)	First order of the most contribution	Lowest of C1 and C2	Intransitivity	Indirect rating without imprecision	MD (95%CrI)	Incoherence, P-value	Higher rating of direct and indirect without imprecision	Incoherence	NMA imprecision	Network rating with imprecision
MIS-PA	DAA	0.28(-0.40,0.97)	PA	high	no	high	0.04(-0.43,0.51)	0.3309	high	no	serious	mod
MIS-PA	DLA	0.05(-0.48,0.58)	DAA	mod	no	mod	0.05(-0.48,0.58)	NR	mod	no	serious	low
MIS-PA	2-incision						0.21(-0.43,0.85)	NR	high	no	serious	mod
DAA	DLA						0.01(-0.37,0.39)	0.4494	high	no	serious	mod
DAA	2-incision	0.17(-0.62,0.97)	PA, MIS-PA	mod	no	mod	0.17(-0.62,0.97)	NR	mod	no	serious	low
DLA	2-incision	0.16(-0.67,1.00)	DAA, MIS-PA	mod	no	mod	0.16(-0.67,1.00)	NR	mod	no	serious	low

© 2023 Yan L et al. *JAMA Network Open.*

Outcome : Cup Abduction angle

Comparison groups		Direct evidence								
Arm 1	Arm 2	MD (95%CrI)	RoB	Inconsistency	Indirectness	Publicaion bias	Direct rating without imprecision	Need to rate indiret ?	Imprecision	Direct rating with imprecision
MIS-PA	DLA									
MIS-PA	PA	0.61 (-1.46, 2.67)	no	no	no	no	high	no	serious	mod
MIS-PA	MIS-ALA	-0.77 (-4.70, 3.16)	no	no	no	no	high	no	serious	mod
MIS-PA	2-incision									
MIS-PA	DAA	-1.01 (-3.25, 1.22)	no	no	no	no	high	no	serious	mod
MIS-PA	MIS-DLA									
MIS-PA	SuperPath	-1.00 (-5.63, 3.63)	no	no	no	no	high	no	serious	mod
DLA	PA	0.10 (-4.02, 4.22)	no	no	no	no	high	no	serious	mod
DLA	MIS-ALA	1.36 (-1.90, 4.63)	no	serious	no	no	mod	yes	serious	low

© 2023 Yan L et al. *JAMA Network Open.*

DLA	2-incision									
DLA	DAA	-1.00 (-3.46, 1.46)	serious	no	no	no	mod	yes	serious	low
DLA	MIS-DLA	-0.92 (-3.51, 1.66)	serious	no	no	no	mod	yes	serious	low
DLA	SuperPath									
PA	MIS-ALA	1.11 (-2.44, 4.67)	no	no	no	no	high	no	serious	mod
PA	2-incision									
PA	DAA	0.59 (-2.11, 3.28)	no	no	no	no	high	no	serious	mod
PA	MIS-DLA									
PA	SuperPath	-3.16 (-6.61, 0.28)	no	no	no	no	high	no	serious	mod
MIS-ALA	2-incision	-0.30 (-6.79, 6.19)	serious	no	no	no	mod	yes	serious	low
MIS-ALA	DAA	0.40 (-3.76, 4.56)	no	no	no	no	high	no	serious	mod
MIS-ALA	MIS-DLA	0.05 (-3.27, 3.37)	serious	no	no	no	mod	yes	serious	low
MIS-ALA	SuperPath									
2-incision	DAA									

© 2023 Yan L et al. *JAMA Network Open.*

2-incision	MIS-DLA									
2-incision	SuperPath									
DAA	MIS-DLA									
DAA	SuperPath									
MIS-DLA	SuperPath									

© 2023 Yan L et al. *JAMA Network Open.*

Comparison groups		Indirect evidence					Network evidence					
Arm 1	Arm 2	MD (95%CrI)	First order of the most contribution	Lowest of C1 and C2	Intransitivity	Indirect rating without imprecision	MD (95%CrI)	Incoherence, P-value	Higher rating of direct and indirect without imprecision	Incoherence	NMA imprecision	Network rating with imprecision
MIS-PA	DLA	-0.03 (-2.29, 2.23)	PA	high	no	high	-0.03 (-2.29, 2.23)	NR	high	no	serious	mod
MIS-PA	PA						-0.08 (-1.70, 1.54)	0.2962	high	no	serious	mod
MIS-PA	MIS-ALA						-0.07 (-2.30, 2.17)	0.6695	high	no	serious	mod
MIS-PA	2-incision	-0.37 (-7.23, 6.50)	MIS-ALA	mod	no	mod	-0.37 (-7.23, 6.50)	NR	mod	no	serious	low
MIS-PA	DAA						-0.51 (-2.21, 1.20)	0.4911	high	no	serious	mod
MIS-PA	MIS-DLA	-0.60 (-3.48, 2.27)	DAA,MIS-ALA	mod	no	mod	-0.60 (-3.48, 2.27)	NR	mod	no	serious	low

© 2023 Yan L et al. *JAMA Network Open.*

Comparison groups		Indirect evidence					Network evidence					
Arm 1	Arm 2	MD (95%CrI)	First order of the most contribution	Lowest of C1 and C2	Intransitivity	Indirect rating without imprecision	MD (95%CrI)	Incoherence, P-value	Higher rating of direct and indirect without imprecision	Incoherence	NMA imprecision	Network rating with imprecision
MIS-PA	Super Path						-2.44 (-5.40, 0.51)	0.4273	high	no	serious	mod
DLA	PA						-0.05 (-2.19, 2.10)	0.935	high	no	serious	mod
DLA	MIS-ALA	-0.90(-3.45,1.66)	MIS-DLA	mod	no	mod	-0.03 (-2.05, 1.98)	0.2853	mod	no	serious	low
DLA	2-incision	-0.33 (-7.13, 6.46)	MIS-ALA	mod	no	mod	-0.33 (-7.13, 6.46)	NR	mod	no	serious	low
DLA	DAA	0.31(-2.69,3.32)	MIS-ALA,MIS-DLA	mod	no	mod	-0.47 (-2.38, 1.43)	0.5073	mod	no	serious	low
DLA	MIS-DLA	0.29(-3.74,4.33)	MIS-DLA	mod	no	mod	-0.57 (-2.75, 1.61)	0.6185	mod	no	serious	low

© 2023 Yan L et al. *JAMA Network Open.*

Comparison groups		Indirect evidence					Network evidence					
Arm 1	Arm 2	MD (95%CrI)	First order of the most contribution	Lowest of C1 and C2	Intransitivity	Indirect rating without imprecision	MD (95%CrI)	Incoherence, P-value	Higher rating of direct and indirect without imprecision	Incoherence	NMA imprecision	Network rating with imprecision
DLA	Super Path	-2.41 (-5.85, 1.02)	DAA,MIS-PA	mod	no	mod	-2.41 (-5.85, 1.02)	NR	mod	no	serious	low
PA	MIS-ALA						0.01 (-2.15, 2.17)	0.4443	high	no	serious	mod
PA	2-incision	-0.29 (-7.13, 6.55)	MIS-ALA	mod	no	mod	-0.29 (-7.13, 6.55)	NR	mod	no	serious	low
PA	DAA						-0.43 (-2.17, 1.31)	0.3332	high	no	serious	mod
PA	MIS-DLA	-0.52 (-3.32, 2.27)	DLA	mod	no	mod	-0.52 (-3.32, 2.27)	NR	mod	no	serious	low
PA	Super Path						-2.37 (-5.19, 0.46)	0.4273	high	no	serious	mod

© 2023 Yan L et al. *JAMA Network Open.*

Comparison groups		Indirect evidence					Network evidence					
Arm 1	Arm 2	MD (95%CrI)	First order of the most contribution	Lowest of C1 and C2	Intransitivity	Indirect rating without imprecision	MD (95%CrI)	Incoherence, P-value	Higher rating of direct and indirect without imprecision	Incoherence	NMA imprecision	Network rating with imprecision
MIS-ALA	2-incision	-0.33 (-7.13, 6.46)	MIS-ALA	mod	no	mod	-0.33 (-7.13, 6.46)	NR	mod	no	serious	low
MIS-ALA	DAA						-0.44 (-2.53, 1.65)	0.6472	high	no	serious	mod
MIS-ALA	MIS-DLA	-1.17(-4.62,2.29)	DLA,PA	mod	no	mod	-0.53 (-2.93, 1.86)	0.6185	mod	no	serious	low
MIS-ALA	Super Path	-2.38 (-5.81, 1.06)	DAA,MIS-PA	high	no	high	-2.38 (-5.81, 1.06)	NR	high	no	serious	mod
2-incision	DAA	-0.14 (-6.96, 6.68)	MIS-ALA	mod	no	mod	-0.14 (-6.96, 6.68)	NR	mod	no	serious	low
2-incision	MIS-DLA	-0.23 (-7.16, 6.69)	MIS-ALA	mod	no	mod	-0.23 (-7.16, 6.69)	NR	mod	no	serious	low

© 2023 Yan L et al. *JAMA Network Open.*

Comparison groups		Indirect evidence					Network evidence					
Arm 1	Arm 2	MD (95%CrI)	First order of the most contribution	Lowest of C1 and C2	Intransitivity	Indirect rating without imprecision	MD (95%CrI)	Incoherence, P-value	Higher rating of direct and indirect without imprecision	Incoherence	NMA imprecision	Network rating with imprecision
2-incision	Super Path	-2.08 (-9.42, 5.27)	MIS-ALA,MIS-PA	mod	no	mod	-2.08 (-9.42, 5.27)	NR	mod	no	serious	low
DAA	MIS-DLA	-0.10 (-2.76, 2.57)	MIS-ALA	mod	no	mod	-0.10 (-2.76, 2.57)	NR	mod	no	serious	low
DAA	Super Path	-1.94 (-5.10, 1.23)	MIS-PA	high	no	high	-1.94 (-5.10, 1.23)	NR	high	no	serious	mod
MIS-DLA	Super Path	-1.84 (-5.72, 2.03)	DLA,PA	mod	no	mod	-1.84 (-5.72, 2.03)	NR	mod	no	serious	low

© 2023 Yan L et al. *JAMA Network Open.*

Outcome : Cup Anteversion angle

Comparison groups		Direct evidence								
Arm 1	Arm 2	MD (95%CrI)	RoB	Inconsistency	Indirectness	Publicaion bias	Direct rating without imprecision	Need to rate indiret ?	Imprecision	Direct rating with imprecision
2-incision	DAA									
2-incision	MIS-DLA									
2-incision	MIS-PA									
2-incision	PA									
2-incision	MIS-ALA	-1.90 (-10.55, 6.75)	serious	no	no	no	mod	yes	serious	low
2-incision	SuperPath									
2-incision	DLA									
DAA	MIS-DLA									
DAA	MIS-PA	-1.14 (-4.51, 2.22)	no	no	no	no	high	no	serious	mod

© 2023 Yan L et al. *JAMA Network Open.*

Comparison groups		Direct evidence								
Arm 1	Arm 2	MD (95%CrI)	RoB	Inconsistency	Indirectness	Publicaion bias	Direct rating without imprecision	Need to rate indiret?	Imprecision	Direct rating with imprecision
DAA	PA	-0.40 (-3.77, 2.96)	no	no	no	no	high	no	serious	mod
DAA	MIS-ALA	-0.10 (-5.30, 5.10)	no	no	no	no	high	no	serious	mod
DAA	SuperPath									
DAA	DLA									
MIS-DLA	MIS-PA									
MIS-DLA	PA									
MIS-DLA	MIS-ALA	-1.00 (-6.72, 4.72)	serious	no	no	no	mod	yes	serious	low
MIS-DLA	SuperPath									
MIS-DLA	DLA									
MIS-PA	PA	-0.34 (-3.48, 2.81)	no	no	no	no	high	no	serious	mod
MIS-PA	MIS-ALA									

© 2023 Yan L et al. *JAMA Network Open.*

Comparison groups		Direct evidence								
Arm 1	Arm 2	MD (95%CrI)	RoB	Inconsistency	Indirectness	Publicaion bias	Direct rating without imprecision	Need to rate indiret?	Imprecision	Direct rating with imprecision
MIS-PA	SuperPath	-1.00 (-6.17, 4.17)	no	no	no	no	high	no	serious	mod
MIS-PA	DLA									
PA	MIS-ALA	-0.30 (-5.39, 4.79)	no	no	no	no	high	no	serious	mod
PA	SuperPath	0.20 (-3.63, 4.02)	no	no	no	no	high	no	serious	mod
PA	DLA	-0.90 (-6.13, 4.33)	no	no	no	no	high	no	serious	mod
MIS-ALA	SuperPath									
MIS-ALA	DLA	0.52 (-5.29, 6.33)	no	no	no	no	high	no	serious	mod
SuperPath	DLA									

© 2023 Yan L et al. *JAMA Network Open.*

Comparison groups		Indirect evidence					Network evidence					
Arm 1	Arm 2	MD (95%CrI)	First order of the most contribution	Lowest of C1 and C2	Intransitivity	Indirect rating without imprecision	MD (95%CrI)	Incoherence, P-value	Higher rating of direct and indirect without imprecision	Incoherence	NMA imprecision	Network rating with imprecision
2-incision	DAA	-1.08 (-10.46, 8.30)	MIS-ALA	mod	no	mod	-1.08 (-10.46, 8.30)	NR	mod	no	serious	low
2-incision	MIS-DLA	-0.90 (-11.28, 9.48)	MIS-ALA	mod	no	mod	-0.90 (-11.28, 9.48)	NR	mod	no	serious	low
2-incision	MIS-PA	-1.65 (-11.16, 7.86)	MIS-ALA,PA	mod	no	mod	-1.65 (-11.16, 7.86)	NR	mod	no	serious	low
2-incision	PA	-1.75 (-11.06, 7.56)	MIS-ALA	mod	no	mod	-1.75 (-11.06, 7.56)	NR	mod	no	serious	low
2-incision	MIS-ALA						-1.90 (-10.55, 6.75)	NR	mod	no	serious	low

© 2023 Yan L et al. *JAMA Network Open.*

Comparison groups		Indirect evidence					Network evidence					
Arm 1	Arm 2	MD (95%CrI)	First order of the most contribution	Lowest of C1 and C2	Intransitivity	Indirect rating without imprecision	MD (95%CrI)	Incoherence, P-value	Higher rating of direct and indirect without imprecision	Incoherence	NMA imprecision	Network rating with imprecision
2-incision	Super Path	-1.94 (-11.75, 7.86)	MIS-ALA,PA	mod	no	mod	-1.94 (-11.75, 7.86)	NR	mod	no	serious	low
2-incision	DLA	-2.08 (-11.76, 7.59)	MIS-ALA	mod	no	mod	-2.08 (-11.76, 7.59)	NR	mod	no	serious	low
DAA	MIS-DLA	0.18 (-6.59, 6.95)	MIS-ALA	mod	no	mod	0.18 (-6.59, 6.95)	NR	mod	no	serious	low
DAA	MIS-PA						-0.57 (-3.18, 2.04)	0.5989	high	no	serious	mod
DAA	PA						-0.67 (-3.15, 1.81)	0.8162	high	no	serious	mod
DAA	MIS-ALA						-0.82 (-4.44, 2.80)	0.7057	high	no	serious	mod
DAA	Super Path	-0.86 (-4.67, 2.95)	MIS-PA	high	no	high	-0.86 (-4.67, 2.95)	NR	high	no	serious	mod

© 2023 Yan L et al. *JAMA Network Open.*

Comparison groups		Indirect evidence					Network evidence					
Arm 1	Arm 2	MD (95%CrI)	First order of the most contribution	Lowest of C1 and C2	Intransitivity	Indirect rating without imprecision	MD (95%CrI)	Incoherence, P-value	Higher rating of direct and indirect without imprecision	Incoherence	NMA imprecision	Network rating with imprecision
DAA	DLA	-1.00 (-5.63, 3.63)	PA	high	no	high	-1.00 (-5.63, 3.63)	NR	high	no	serious	mod
MIS-DLA	MIS-PA	-0.75 (-7.70, 6.20)	MIS-ALA,PA	mod	no	mod	-0.75 (-7.70, 6.20)	NR	mod	no	serious	low
MIS-DLA	PA	-0.85 (-7.53, 5.83)	MIS-ALA	mod	no	mod	-0.85 (-7.53, 5.83)	NR	mod	no	serious	low
MIS-DLA	MIS-ALA						-1.90 (-10.55, 6.75)	NR	mod	no	serious	low
MIS-DLA	SuperPath	-1.04 (-8.40, 6.31)	MIS-ALA,PA	mod	no	mod	-1.04 (-8.40, 6.31)	NR	mod	no	serious	low
MIS-DLA	DLA	-1.18 (-8.36, 5.99)	PA	high	no	high	-1.18 (-8.36, 5.99)	NR	high	no	serious	mod
MIS-PA	PA						-0.10 (-2.49, 2.29)	0.8203	high	no	serious	mod
MIS-PA	MIS-ALA	-0.25 (-4.20, 3.69)	PA,SuperPath	high	no	high	-0.25 (-4.20, 3.69)	NR	high	no	serious	mod

© 2023 Yan L et al. *JAMA Network Open.*

Comparison groups		Indirect evidence					Network evidence					
Arm 1	Arm 2	MD (95%CrI)	First order of the most contribution	Lowest of C1 and C2	Intransitivity	Indirect rating without imprecision	MD (95%CrI)	Incoherence, P-value	Higher rating of direct and indirect without imprecision	Incoherence	NMA imprecision	Network rating with imprecision
MIS-PA	SuperPath						-0.29 (-3.73, 3.15)	0.719	high	no	serious	mod
MIS-PA	DLA	-0.43 (-5.15, 4.29)	PA,SuperPath	high	no	high	-0.43 (-5.15, 4.29)	NR	high	no	serious	mod
PA	MIS-ALA						-0.15 (-3.60, 3.30)	0.9372	high	no	serious	mod
PA	SuperPath						-0.19 (-3.38, 3.00)	0.719	high	no	serious	mod
PA	DLA						-0.33 (-4.51, 3.85)	0.7226	high	no	serious	mod
MIS-ALA	SuperPath	-0.04 (-4.66, 4.57)	PA	high	no	high	-0.04 (-4.66, 4.57)	NR	high	no	serious	mod
MIS-ALA	DLA						-0.18 (-4.51, 4.15)	0.7226	high	no	serious	mod
SuperPath	DLA	-0.14 (-5.37, 5.09)	PA	high	no	high	-0.14 (-5.37, 5.09)	NR	high	no	serious	mod

© 2023 Yan L et al. *JAMA Network Open.*

eTable 7. League Table for Outcome Measures

League tables of other outcome analyses. The league tables show the relative effects of each approach (the approach on the column to the treatment of the row). Approaches are reported in order of P-score. The relative effects are measured as a stardandised mean difference for quality of life score change, a rate ratio for six complications (including dislocation, fracture, infection, nerve injury and thromboembolism), and mean difference for all the other outcomes, along with 95% CIs. Results of the network meta-analysis are presented in the left lower half and results from pairwise meta-analysis in the upper right half, if available. Cells in bold print indicate significant results. NR=not reported. DAA=direct anterior approach. DLA=Direct lateral approach. MIS-ALA=minimally invasive anterolateral approach. MIS-PA=minimally invasive posterior approach. PA=Posterior approach. PLA=posterolateral approach. SuperPath=supercapsular percutaneously assisted total hip arthroplasty.

© 2023 Yan L et al. *JAMA Network Open.*

Outcome : Short-term hip score

**SuperPath**	NR	NR	9.80 (-1.53,21.13)	NR	NR	NR	5.22 (-1.03,11.46)
1.91 (-4.80,8.63)	**DAA**	-0.70 (-10.59,9.19)	4.14 (-0.78,9.07)	NR	1.98 (-3.37,7.32)	3.61 (-2.05,9.27)	6.50 (-0.39,13.39)
3.20 (-4.86,11.25)	1.28 (-4.40,6.96)	**MIS-ALA**	-7.00 (-17.77,3.77)	1.00 (-9.17,11.17)	5.50 (-4.42,15.42)	6.85 (-4.50,18.20)	3.00 (-8.51,14.51)
4.85 (-1.52,11.23)	2.94 (-1.03,6.91)	1.65 (-4.27,7.58)	**MIS-PA**	2.00 (-8.48,12.48)	NR	NR	3.33 (-2.39,9.06)
5.48 (-4.39,15.36)	3.57 (-4.72,11.86)	2.29 (-5.55,10.13)	0.63 (-7.27,8.54)	**2-incision**	NR	NR	NR
5.29 (-2.49,13.07)	3.38 (-1.06,7.82)	2.09 (-4.25,8.43)	0.44 (-5.23,6.11)	-0.19 (-9.18,8.79)	**MIS-DLA**	-0.55 (-7.23,6.12)	NR
6.11 (-1.28,13.49)	**4.19 (0.04,8.35)**	2.91 (-3.18,9.00)	1.26 (-4.04,6.55)	0.62 (-8.16,9.41)	0.82 (-3.97,5.60)	**DLA**	-1.00 (-10.43,8.43)
**6.72 (1.16,12.28)**	**4.81 (0.42,9.19)**	3.53 (-2.73,9.78)	1.87 (-2.40,6.14)	1.24 (-7.33,9.81)	1.43 (-4.43,7.29)	0.62 (-4.66,5.89)	**PA**

© 2023 Yan L et al. *JAMA Network Open.*

Outcome : Long-term hip score

**DAA**	0.97 (-0.74,2.69)	NR	NR	0.42 (-2.12,2.95)	0.00 (-3.49,3.49)	2.19 (-0.16,4.55)	2.64 (-0.48,5.76)
0.36 (-1.08,1.81)	**MIS-PA**	1.03 (-1.24,3.29)	NR	1.51 (-1.28,4.30)	6.00 (1.22,10.78)	NR	NR
1.20 (-1.12,3.51)	0.83 (-1.17,2.84)	**2-incision**	NR	NR	1.00 (-2.78,4.78)	NR	NR
1.46 (-0.72,3.65)	1.10 (-1.23,3.42)	0.27 (-2.65,3.18)	**SuperPath**	0.02 (-1.48,1.52)	NR	NR	NR
1.48 (-0.11,3.07)	1.12 (-0.66,2.90)	0.29 (-2.21,2.78)	0.02 (-1.48,1.52)	**PA**	0.16 (-2.43,2.74)	-1.30 (-4.06,1.46)	6.00 (-8.09,20.09)
**1.65 (0.04,3.27)**	1.29 (-0.55,3.13)	0.46 (-1.91,2.83)	0.19 (-2.09,2.48)	0.17 (-1.56,1.90)	**MIS-ALA**	0.29 (-2.27,2.85)	1.09 (-0.98,3.15)
**1.78 (0.12,3.43)**	1.42 (-0.58,3.41)	0.58 (-2.03,3.20)	0.32 (-2.01,2.64)	0.29 (-1.48,2.07)	0.12 (-1.59,1.84)	**DLA**	-3.20 (-9.17,2.77)
**2.46 (0.49,4.44)**	2.10 (-0.15,4.35)	1.27 (-1.50,4.03)	1.00 (-1.66,3.66)	0.98 (-1.22,3.18)	0.81 (-0.92,2.54)	0.68 (-1.48,2.85)	**MIS-DLA**

© 2023 Yan L et al. *JAMA Network Open.*

Outcome : Dislocation

**SuperPath**	NR	NR	0.50 (0.05,5.32)	NR	NR	NR	NR
0.74 (0.03,19.68)	**MIS-DLA**	NR	0.32 (0.01,7.68)	NR	0.96 (0.06,14.96)	NR	NR
0.57 (0.04,8.02)	0.76 (0.06,9.86)	**MIS-PA**	0.94 (0.24,3.73)	NR	NR	0.65 (0.08,5.16)	0.53 (0.09,3.15)
0.50 (0.05,5.32)	0.67 (0.07,6.47)	0.88 (0.27,2.94)	**PA**	0.58 (0.07,5.04)	0.67 (0.12,3.74)	1.12 (0.26,4.75)	NR
0.48 (0.03,8.48)	0.65 (0.04,10.49)	0.85 (0.13,5.78)	0.96 (0.19,4.88)	**DLA**	NR	0.53 (0.06,4.49)	NR
0.41 (0.02,7.18)	0.56 (0.06,4.94)	0.73 (0.10,5.40)	0.83 (0.17,4.08)	0.86 (0.09,8.38)	**MIS-ALA**	NR	NR
0.42 (0.03,5.83)	0.56 (0.04,7.15)	0.74 (0.18,3.01)	0.84 (0.26,2.66)	0.87 (0.17,4.40)	1.01 (0.14,7.26)	**DAA**	NR
0.30 (0.01,7.35)	0.41 (0.02,9.17)	0.53 (0.09,3.15)	0.60 (0.07,5.15)	0.63 (0.05,8.55)	0.73 (0.05,10.55)	0.72 (0.08,6.91)	**2-incision**

© 2023 Yan L et al. *JAMA Network Open.*

Outcome : Fracture

**MIS-PA**	0.86 (0.21,3.58)	NR	1.02 (0.19,5.40)	NR	0.34 (0.07,1.63)	0.09 (0.01,0.68)
0.90 (0.30,2.64)	**PA**	5.14 (0.26,103.34)	0.69 (0.14,3.52)	0.42 (0.08,2.31)	NR	0.09 (0.01,1.63)
0.89 (0.20,3.98)	0.99 (0.23,4.28)	**MIS-DLA**	0.67 (0.11,4.03)	NR	0.38 (0.02,7.95)	0.99 (0.11,9.27)
0.57 (0.18,1.78)	0.64 (0.22,1.84)	0.65 (0.16,2.56)	**DAA**	1.25 (0.26,6.10)	NR	3.00 (0.13,69.42)
0.55 (0.13,2.31)	0.61 (0.18,2.09)	0.62 (0.12,3.33)	0.95 (0.29,3.19)	**DLA**	NR	0.29 (0.01,6.90)
0.34 (0.08,1.42)	0.38 (0.07,2.11)	0.38 (0.06,2.39)	0.59 (0.11,3.32)	0.62 (0.09,4.35)	**2-incision**	NR
**0.19 (0.05,0.78)**	**0.22 (0.05,0.90)**	0.22 (0.04,1.08)	0.34 (0.08,1.44)	0.36 (0.07,1.76)	0.57 (0.09,3.85)	**MIS-ALA**

© 2023 Yan L et al. *JAMA Network Open.*

Outcome : Infection

**PA**	0.20 (0.01,4.00)	0.93 (0.14,6.12)	NR	NR	0.58 (0.16,2.19)	NR
0.82 (0.15,4.57)	**DLA**	0.73 (0.12,4.27)	NR	0.68 (0.11,4.06)	NR	NR
0.81 (0.19,3.48)	0.98 (0.22,4.41)	**DAA**	NR	NR	0.33 (0.01,7.99)	NR
0.70 (0.07,6.74)	0.84 (0.07,10.48)	0.86 (0.07,10.18)	**2-incision**	NR	0.51 (0.05,5.42)	0.29 (0.01,6.19)
0.68 (0.08,5.55)	0.83 (0.16,4.19)	0.84 (0.11,6.49)	0.98 (0.08,12.75)	**MIS-ALA**	NR	0.14 (0.02,1.17)
0.48 (0.14,1.58)	0.58 (0.09,3.67)	0.59 (0.11,3.06)	0.68 (0.09,5.07)	0.70 (0.09,5.67)	**MIS-PA**	0.29 (0.01,6.19)
0.13 (0.02,1.06)	0.15 (0.02,1.25)	0.16 (0.02,1.45)	0.18 (0.02,1.71)	0.19 (0.03,1.16)	0.27 (0.04,1.92)	**MIS-DLA**

© 2023 Yan L et al. *JAMA Network Open.*

Outcome : Nerve injury

**PA**	NR	NR	NR	NR	**0.04 (0.01,0.33)**	NR
0.47 (0.03,7.67)	**MIS-ALA**	NR	0.38 (0.02,8.97)	0.20 (0.01,3.91)	0.07 (0.00,1.12)	NR
0.30 (0.01,6.83)	0.63 (0.04,11.40)	**MIS-PA**	NR	NR	**0.06 (0.00,0.99)**	0.09 (0.01,1.40)
0.25 (0.02,3.24)	0.54 (0.07,4.47)	0.85 (0.05,14.16)	**DLA**	NR	**0.16 (0.03,0.85)**	NR
**0.05 (0.00,0.87)**	**0.10 (0.01,0.94)**	0.16 (0.01,2.27)	0.19 (0.02,2.21)	**MIS-DLA**	5.00 (0.26,97.85)	0.10 (0.01,1.57)
**0.04 (0.01,0.33)**	**0.09 (0.01,0.64)**	0.15 (0.01,1.61)	**0.17 (0.04,0.82)**	0.92 (0.11,7.41)	**DAA**	NR
**0.01 (0.00,0.32)**	**0.02 (0.00,0.46)**	**0.04 (0.00,0.40)**	**0.05 (0.00,0.90)**	0.24 (0.02,2.50)	0.26 (0.02,3.70)	**2-incision**

© 2023 Yan L et al. *JAMA Network Open.*

Outcome : Reoperation

**2-incision**	NR	0.70 (0.23,2.14)	NR	NR	NR	NR
0.99 (0.09,11.11)	**MIS-DLA**	NR	0.49 (0.05,5.11)	NR	NR	0.33 (0.01,7.71)
0.70 (0.23,2.14)	0.71 (0.08,6.07)	**MIS-PA**	0.96 (0.30,3.13)	NR	NR	0.17 (0.03,1.01)
0.58 (0.12,2.73)	0.58 (0.08,4.00)	0.82 (0.28,2.43)	**PA**	0.98 (0.06,15.44)	0.76 (0.20,2.94)	0.49 (0.10,2.29)
0.41 (0.05,3.15)	0.41 (0.04,4.25)	0.58 (0.10,3.22)	0.71 (0.18,2.87)	**DLA**	0.96 (0.24,3.81)	1.00 (0.07,15.26)
0.42 (0.06,2.86)	0.42 (0.04,3.92)	0.59 (0.12,2.85)	0.72 (0.22,2.35)	1.02 (0.31,3.37)	**DAA**	NR
0.24 (0.04,1.31)	0.24 (0.03,1.82)	0.34 (0.09,1.23)	0.41 (0.13,1.30)	0.58 (0.12,2.93)	0.58 (0.12,2.69)	**MIS-ALA**

© 2023 Yan L et al. *JAMA Network Open.*

Outcome : Thromboembolism

**MIS-ALA**	0.69 (0.27,1.81)	0.33 (0.01,7.74)	NR	NR	NR	NR	NR
0.66 (0.26,1.68)	**MIS-DLA**	NR	NR	0.86 (0.06,13.22)	NR	NR	0.19 (0.01,3.90)
0.54 (0.07,4.21)	0.82 (0.11,6.05)	**DLA**	NR	0.57 (0.11,2.90)	NR	NR	0.20 (0.01,4.00)
0.50 (0.01,21.94)	0.75 (0.02,31.21)	0.92 (0.03,33.33)	**SuperPath**	NR	NR	NR	0.33 (0.01,7.97)
0.31 (0.04,2.20)	0.47 (0.07,2.99)	0.57 (0.14,2.30)	0.63 (0.02,19.84)	**DAA**	NR	0.97 (0.10,9.18)	0.66 (0.08,5.21)
0.26 (0.01,8.78)	0.39 (0.01,12.45)	0.47 (0.02,12.91)	0.51 (0.01,39.62)	0.82 (0.04,18.72)	**2-incision**	1.03 (0.07,15.81)	NR
0.26 (0.03,2.48)	0.40 (0.05,3.38)	0.48 (0.07,3.14)	0.53 (0.02,15.50)	0.85 (0.19,3.85)	1.03 (0.07,15.81)	**MIS-PA**	0.66 (0.19,2.33)
0.17 (0.02,1.30)	0.25 (0.04,1.76)	0.31 (0.06,1.65)	0.33 (0.01,7.97)	0.53 (0.14,2.10)	0.65 (0.03,12.57)	0.63 (0.20,1.99)	**PA**

© 2023 Yan L et al. *JAMA Network Open.*

Outcome : AMSs change

**PA**	**1.20 (0.91,1.49)**	NR	NR	NR
**1.20 (0.91,1.49)**	**MIS-ALA**	0.30 (-1.96,2.56)	**0.50 (0.01,0.99)**	0.90 (-0.94,2.74)
1.50 (-0.78,3.78)	0.30 (-1.96,2.56)	**DAA**	NR	NR
**1.70 (1.13,2.27)**	**0.50 (0.01,0.99)**	0.20 (-2.11,2.51)	**DLA**	NR
**2.10 (0.24,3.96)**	0.90 (-0.94,2.74)	0.60 (-2.32,3.52)	0.40 (-1.51,2.31)	**MIS-DLA**

© 2023 Yan L et al. *JAMA Network Open.*

Outcome : Analgesic consumption

**MIS-DLA**	2.77 (-111.37,116.91)	NR	**-1210.00 (-1646.20,-773.80)**	NR	NR	NR
-71.41 (-181.95,39.12)	**DAA**	NR	-18.00 (-98.71,62.72)	NR	-44.00 (-161.51,73.51)	-140.00 (-288.76,8.76)
-71.39 (-255.63,112.86)	0.03 (-150.10,150.15)	**MIS-ALA**	-55.12 (-182.49,72.25)	NR	NR	NR
-126.51 (-259.63,6.62)	-55.09 (-134.54,24.36)	-55.12 (-182.49,72.25)	**DLA**	NR	NR	NR
-148.79 (-334.57,36.99)	-77.38 (-226.70,71.94)	-77.40 (-289.14,134.34)	-22.28 (-191.42,146.86)	**2-incision**	0.00 (-112.71,112.71)	NR
**-148.79 (-296.47,-1.11)**	-77.38 (-175.31,20.56)	-77.40 (-256.65,101.84)	-22.28 (-148.40,103.83)	0.00 (-112.71,112.71)	**MIS-PA**	13.28 (-83.03,109.59)
**-157.93 (-311.28,-4.57)**	-86.51 (-192.81,19.78)	-86.54 (-270.48,97.41)	-31.42 (-164.13,101.29)	-9.14 (-150.83,132.56)	-9.14 (-95.00,76.73)	**PA**

© 2023 Yan L et al. *JAMA Network Open.*

Outcome : Cadence change

**DAA**	NR	0.20 (-2.84,3.24)	NR	NR	**9.00 (3.14,14.86)**
-0.50 (-9.45,8.45)	**MIS-PA**	0.70 (-7.72,9.12)	NR	NR	NR
0.20 (-2.84,3.24)	0.70 (-7.72,9.12)	**PA**	NR	NR	NR
5.80 (-6.86,18.46)	6.30 (-9.21,21.81)	5.60 (-7.42,18.62)	**MIS-DLA**	3.00 (-5.38,11.38)	NR
8.80 (-0.70,18.30)	9.30 (-3.75,22.35)	8.60 (-1.37,18.57)	3.00 (-5.38,11.38)	**MIS-ALA**	0.20 (-7.27,7.67)
**9.00 (3.14,14.86)**	9.50 (-1.20,20.20)	**8.80 (2.20,15.40)**	3.20 (-8.03,14.43)	0.20 (-7.27,7.67)	**DLA**

© 2023 Yan L et al. *JAMA Network Open.*

Outcome : CK change

**SuperPath**	NR	NR	NR	**-156.26 (-288.04,-24.49)**	NR	NR
-149.07 (-352.12,53.98)	**DAA**	NR	NR	-21.70 (-199.82,156.42)	-12.79 (-117.57,91.99)	NR
-149.58 (-371.78,72.63)	-0.51 (-154.48,153.46)	**MIS-ALA**	-26.77 (-162.81,109.28)	23.00 (-231.81,277.81)	1.71 (-176.77,180.19)	-39.00 (-277.83,199.83)
-156.83 (-395.48,81.81)	-7.76 (-175.08,159.56)	-7.25 (-126.87,112.36)	**MIS-DLA**	NR	-50.60 (-246.72,145.52)	NR
**-156.26 (-288.04,-24.49)**	-7.19 (-161.68,147.29)	-6.69 (-185.60,172.23)	0.57 (-198.40,199.53)	**PA**	NR	-62.00 (-295.57,171.57)
-166.88 (-380.96,47.19)	-17.81 (-118.00,82.38)	-17.31 (-148.35,113.74)	-10.05 (-152.61,132.51)	-10.62 (-179.33,158.10)	**DLA**	NR
-203.99 (-457.57,49.60)	-54.92 (-290.16,180.32)	-54.41 (-273.88,165.06)	-47.16 (-290.02,195.70)	-47.72 (-264.38,168.94)	-37.11 (-270.74,196.53)	**MIS-PA**

© 2023 Yan L et al. *JAMA Network Open.*

Outcome : CRP change

**SuperPath**	NR	-9.82 (-26.30,6.66)	NR	NR	NR	NR
-4.78 (-29.25,19.70)	**DAA**	-4.50 (-28.00,19.00)	-8.95 (-24.17,6.27)	NR	NR	NR
-9.82 (-26.30,6.66)	-5.04 (-23.13,13.05)	**PA**	-2.00 (-29.12,25.12)	-4.39 (-24.78,16.01)	NR	-5.00 (-31.44,21.44)
-13.50 (-38.05,11.04)	-8.72 (-22.58,5.13)	-3.68 (-21.87,14.51)	**DLA**	-1.00 (-29.20,27.20)	-1.13 (-20.56,18.29)	-3.00 (-30.59,24.59)
-14.39 (-40.09,11.31)	-9.61 (-33.30,14.07)	-4.57 (-24.30,15.15)	-0.89 (-23.23,21.45)	**MIS-PA**	NR	-2.00 (-29.55,25.55)
-15.01 (-42.99,12.98)	-10.23 (-31.36,10.90)	-5.19 (-27.81,17.43)	-1.51 (-18.40,15.39)	-0.62 (-26.20,24.96)	**MIS-ALA**	0.16 (-22.65,22.97)
-15.36 (-42.39,11.67)	-10.58 (-32.85,11.69)	-5.54 (-26.97,15.89)	-1.86 (-21.29,17.57)	-0.97 (-24.78,22.84)	-0.35 (-18.94,18.23)	**MIS-DLA**

© 2023 Yan L et al. *JAMA Network Open.*

Outcome : ESR change

**DAA**	**-6.50 (-11.66,-1.34)**	NR	**-26.80 (-33.08,-20.52)**
**-6.50 (-11.66,-1.34)**	**DLA**	NR	NR
**-26.65 (-34.53,-18.77)**	**-20.15 (-29.56,-10.73)**	**SuperPath**	-0.15 (-4.91,4.60)
**-26.80 (-33.08,-20.52)**	**-20.30 (-28.43,-12.17)**	**-0.15 (-4.91,4.60)**	**PA**

© 2023 Yan L et al. *JAMA Network Open.*

Outcome : Hb change

**2-incision**	NR	NR	**10.00 (3.36,16.64)**	NR	NR	NR	NR
**9.23 (0.80,17.66)**	**MIS-DLA**	NR	1.00 (-8.93,10.93)	-3.60 (-11.80,4.60)	2.52 (-3.61,8.65)	4.73 (-0.71,10.16)	NR
**9.92 (1.50,18.33)**	0.68 (-4.33,5.70)	**MIS-ALA**	0.00 (-7.63,7.63)	NR	3.22 (-4.78,11.22)	1.65 (-1.90,5.20)	NR
**10.00 (3.36,16.64)**	0.77 (-4.43,5.96)	0.08 (-5.09,5.26)	**MIS-PA**	NR	1.86 (-1.79,5.52)	-3.00 (-17.37,11.37)	6.85 (-3.65,17.35)
**11.37 (3.00,19.73)**	2.14 (-2.41,6.68)	1.45 (-3.25,6.16)	1.37 (-3.72,6.45)	**DAA**	-0.99 (-6.41,4.43)	-0.67 (-5.42,4.08)	NR
**11.49 (3.95,19.03)**	2.26 (-2.17,6.69)	1.58 (-3.12,6.27)	1.49 (-2.07,5.06)	0.12 (-4.00,4.25)	**PA**	-7.00 (-20.50,6.50)	NR
**11.76 (3.44,20.09)**	2.53 (-1.71,6.78)	1.85 (-1.43,5.13)	1.76 (-3.25,6.78)	0.40 (-3.44,4.23)	0.27 (-4.07,4.62)	**DLA**	NR
**16.85 (4.42,29.28)**	7.62 (-4.10,19.34)	6.93 (-4.77,18.64)	6.85 (-3.65,17.35)	5.48 (-6.19,17.15)	5.36 (-5.73,16.45)	5.09 (-6.56,16.73)	**SuperPath**

© 2023 Yan L et al. *JAMA Network Open.*

Outcome : HCT change

**SuperPath**	NR	NR	NR	-2.31 (-5.39,0.77)	NR	NR
-1.07 (-5.32,3.17)	**MIS-DLA**	NR	-1.15 (-3.31,1.01)	NR	NR	NR
-1.32 (-5.28,2.63)	-0.25 (-2.88,2.38)	**DLA**	-0.90 (-2.40,0.60)	NR	NR	-1.54 (-2.71,-0.36)
-2.22 (-5.88,1.44)	-1.15 (-3.31,1.01)	-0.90 (-2.40,0.60)	**DAA**	NR	-0.50 (-2.09,1.09)	NR
-2.31 (-5.39,0.77)	-1.24 (-4.16,1.69)	-0.99 (-3.47,1.49)	-0.09 (-2.06,1.88)	**MIS-PA**	-0.41 (-1.57,0.75)	NR
-2.72 (-6.02,0.57)	-1.65 (-4.33,1.03)	-1.40 (-3.59,0.79)	-0.50 (-2.09,1.09)	-0.41 (-1.57,0.75)	**PA**	NR
-2.86 (-6.99,1.26)	-1.79 (-4.67,1.09)	**-1.54 (-2.71,-0.36)**	-0.64 (-2.55,1.27)	-0.55 (-3.29,2.19)	-0.14 (-2.62,2.35)	**MIS-ALA**

© 2023 Yan L et al. *JAMA Network Open.*

Outcome : IL-6 change

**DAA**	NR	NR	-5.10 (-16.03,5.83)	-5.74 (-15.57,4.10)
-4.86 (-35.08,25.36)	**MIS-DLA**	-14.90 (-64.26,34.46)	2.40 (-30.71,35.51)	1.60 (-37.45,40.65)
-5.31 (-20.16,9.53)	-0.45 (-31.14,30.24)	**MIS-PA**	-0.12 (-10.80,10.55)	16.50 (-36.98,69.98)
-5.38 (-15.89,5.14)	-0.52 (-29.94,28.91)	-0.06 (-10.72,10.59)	**PA**	-0.80 (-39.78,38.18)
-5.52 (-15.05,4.02)	-0.65 (-31.30,29.99)	-0.20 (-17.35,16.94)	-0.14 (-13.82,13.55)	**DLA**

© 2023 Yan L et al. *JAMA Network Open.*

Outcome : Leg length discrepancy

**DAA**	0.00 (-1.80,1.80)	-2.00 (-4.36,0.36)	NR	NR
0.00 (-1.80,1.80)	**DLA**	NR	NR	NR
-2.00 (-4.36,0.36)	-2.00 (-4.97,0.97)	**PA**	-0.04 (-0.28,0.21)	-0.18 (-0.62,0.26)
-2.03 (-4.41,0.34)	-2.03 (-5.01,0.95)	-0.03 (-0.28,0.21)	**MIS-PA**	-0.01 (-0.46,0.45)
-2.13 (-4.53,0.27)	-2.13 (-5.13,0.87)	-0.13 (-0.55,0.29)	-0.09 (-0.52,0.33)	**MIS-ALA**

© 2023 Yan L et al. *JAMA Network Open.*

Outcome : Myoglobin change

**PA**	-22.00 (-77.22,33.22)	NR	-74.00 (-150.25,2.25)	NR	NR
-22.00 (-77.22,33.22)	**MIS-PA**	NR	-52.00 (-135.90,31.90)	NR	NR
-38.20 (-192.44,116.04)	-16.20 (-174.36,141.96)	**DAA**	NR	NR	**-92.00 (-126.28,-57.72)**
-74.00 (-150.25,2.25)	-52.00 (-135.90,31.90)	-35.80 (-169.88,98.28)	**MIS-ALA**	-36.00 (-144.62,72.62)	NR
-110.00 (-242.71,22.71)	-88.00 (-225.25,49.25)	-71.80 (-150.40,6.80)	-36.00 (-144.62,72.62)	**MIS-DLA**	-20.20 (-90.93,50.53)
-130.20 (-280.58,20.18)	-108.20 (-262.60,46.20)	**-92.00 (-126.28,-57.72)**	-56.20 (-185.82,73.42)	-20.20 (-90.93,50.53)	**DLA**

© 2023 Yan L et al. *JAMA Network Open.*

Outcome : Stem alignment

**MIS-ALA**	-0.00 (-1.22,1.22)	0.18 (-0.89,1.25)	NR	-1.00 (-2.46,0.46)	**-1.31 (-2.31,-0.30)**	-0.90 (-2.26,0.46)
-0.00 (-1.22,1.22)	**2-incision**	NR	NR	NR	NR	NR
-0.16 (-0.98,0.66)	-0.16 (-1.63,1.31)	**DLA**	0.03 (-0.79,0.85)	NR	NR	-1.10 (-2.49,0.29)
-0.39 (-1.34,0.55)	-0.39 (-1.94,1.15)	-0.23 (-0.97,0.51)	**DAA**	0.27 (-0.69,1.23)	NR	NR
-0.49 (-1.37,0.40)	-0.49 (-1.99,1.02)	-0.32 (-1.27,0.62)	-0.09 (-0.92,0.74)	**PA**	-0.33 (-1.06,0.39)	NR
**-0.89 (-1.76,-0.03)**	-0.89 (-2.39,0.60)	-0.73 (-1.75,0.29)	-0.50 (-1.48,0.49)	-0.41 (-1.09,0.28)	**MIS-PA**	NR
**-1.08 (-2.13,-0.03)**	-1.08 (-2.69,0.53)	-0.92 (-1.97,0.14)	-0.68 (-1.91,0.54)	-0.59 (-1.86,0.68)	-0.19 (-1.48,1.11)	**MIS-DLA**

© 2023 Yan L et al. *JAMA Network Open.*

Outcome : Step length change

**DAA**	NR	NR	NR	0.00 (-0.33,0.34)	NR	**2.54 (1.94,3.14)**
0.26 (-0.41,0.94)	**SuperPath**	0.00 (-0.46,0.46)	0.09 (-0.39,0.57)	NR	NR	NR
0.29 (-0.26,0.85)	0.03 (-0.35,0.41)	**PA**	0.01 (-0.45,0.47)	0.06 (-0.40,0.52)	NR	NR
0.33 (-0.35,1.00)	0.06 (-0.32,0.45)	0.03 (-0.35,0.42)	**MIS-PA**	NR	NR	NR
**0.35 (0.04,0.66)**	0.09 (-0.51,0.69)	0.06 (-0.40,0.52)	0.03 (-0.57,0.62)	**DLA**	0.01 (-0.31,0.34)	NR
**0.71 (0.31,1.10)**	0.44 (-0.23,1.11)	0.41 (-0.14,0.97)	0.38 (-0.29,1.05)	**0.35 (0.05,0.66)**	**MIS-ALA**	0.00 (-0.46,0.46)
**1.38 (0.94,1.82)**	**1.12 (0.38,1.86)**	**1.09 (0.45,1.72)**	**1.05 (0.31,1.79)**	**1.03 (0.59,1.47)**	**0.67 (0.28,1.07)**	**MIS-DLA**

© 2023 Yan L et al. *JAMA Network Open.*

Outcome : Time up and go test result change

**SuperPath**	NR	NR	NR	**-3.60 (-6.20,-1.00)**	NR
-2.50 (-5.46,0.46)	**DAA**	**-0.45 (-0.74,-0.16)**	**-0.70 (-0.97,-0.43)**	-1.10 (-2.51,0.31)	NR
-2.95 (-5.92,0.02)	-0.45 (-0.74,-0.16)	**DLA**	NR	NR	NR
**-3.20 (-6.17,-0.23)**	**-0.70 (-0.97,-0.43)**	-0.25 (-0.64,0.15)	**MIS-DLA**	NR	NR
**-3.60 (-6.20,-1.00)**	-1.10 (-2.51,0.31)	-0.65 (-2.09,0.79)	-0.40 (-1.83,1.03)	**PA**	-3.03 (-7.54,1.47)
**-6.63 (-11.83,-1.43)**	-4.13 (-8.85,0.59)	-3.68 (-8.41,1.05)	-3.43 (-8.16,1.29)	-3.03 (-7.54,1.47)	**MIS-PA**

© 2023 Yan L et al. *JAMA Network Open.*

Outcome : Volume of blood transfusion

**MIS-ALA**	**-219.36 (-310.72,-128.00)**	NR	NR	NR	NR
**-219.36 (-310.72,-128.00)**	**PA**	-2.06 (-46.66,42.53)	-70.00 (-193.31,53.31)	-42.05 (-101.90,17.80)	-15.37 (-104.84,74.10)
**-219.77 (-321.18,-118.35)**	-0.41 (-44.43,43.62)	**MIS-PA**	-58.00 (-187.82,71.82)	NR	-33.00 (-173.72,107.72)
**-236.39 (-347.72,-125.05)**	-17.03 (-80.65,46.59)	-16.62 (-89.35,56.11)	**MIS-DLA**	8.00 (-73.95,89.95)	**-60.17 (-113.29,-7.06)**
**-249.92 (-355.63,-144.22)**	-30.56 (-83.72,22.59)	-30.16 (-97.40,37.09)	-13.54 (-77.24,50.17)	**DAA**	NR
**-284.47 (-397.53,-171.41)**	-65.11 (-131.70,1.48)	-64.70 (-140.02,10.61)	-48.08 (-98.39,2.23)	-34.55 (-106.59,37.50)	**DLA**

© 2023 Yan L et al. *JAMA Network Open.*

Outcome : Walking speed change

**SuperPath**	NR	NR	**0.03 (0.01,0.05)**	NR	NR	NR	0.08 (-0.08,0.24)
-0.01 (-0.13,0.11)	**2-incision**	NR	NR	NR	0.00 (-0.16,0.16)	NR	**0.10 (0.00,0.20)**
0.02 (-0.03,0.07)	0.03 (-0.09,0.16)	**DAA**	0.00 (-0.05,0.05)	0.06 (-0.01,0.13)	**0.04 (0.03,0.05)**	NR	NR
**0.03 (0.01,0.05)**	0.04 (-0.08,0.16)	0.01 (-0.04,0.05)	**PA**	NR	NR	NR	-0.03 (-0.17,0.11)
0.06 (-0.02,0.14)	0.07 (-0.06,0.21)	0.04 (-0.02,0.10)	0.03 (-0.05,0.11)	**DLA**	NR	0.05 (-0.05,0.15)	NR
**0.06 (0.01,0.11)**	0.07 (-0.05,0.20)	**0.04 (0.03,0.05)**	0.03 (-0.01,0.08)	0.00 (-0.07,0.07)	**MIS-DLA**	-0.04 (-0.15,0.07)	0.10 (-0.06,0.26)
0.07 (-0.02,0.17)	0.08 (-0.06,0.23)	0.05 (-0.03,0.13)	0.04 (-0.05,0.13)	0.01 (-0.07,0.09)	0.01 (-0.07,0.09)	**MIS-ALA**	NR
0.07 (-0.02,0.16)	0.08 (-0.01,0.18)	0.05 (-0.04,0.14)	0.04 (-0.05,0.13)	0.01 (-0.10,0.12)	0.01 (-0.08,0.10)	0.00 (-0.12,0.13)	**MIS-PA**

© 2023 Yan L et al. *JAMA Network Open.*

eTable 8. Results of Regression Analysis

	Publication year	Incision length	Percentage male	Age	BMI	Follow-up time
Hip score change	-0.87 (-4.39,2.5)	0.87 (-3.23,5.01)	-4.29 (-9.25,0.63)	0.16 (-3.48,3.97)	1.48 (-1.56,4.65)	0.75 (-2.43,3.98)
Hospitalization time	**-17.34 (-33.78,-0.53)**	**-21.54 (-51.6,-0.1)**	9.49 (-38.47,24.25)	-1.24 (-5.44,3.03)	-1.25 (-14.42,15.43)	----
Operation time	**13.88 (5.51,22.29)**	-9.74 (-23.07,3.08)	3.79 (-4.85,12.27)	-0.43 (-10.94,10.3)	-7.19 (-16.71,1.82)	----
Blood loss	65.73 (-24.53,159.17)	-45.66 (-166.21,69.31)	-53.046 (-154.45,49.36)	-16.94 (-141.87,107.5)	59.78 (-43.11,167.29)	----
Pain score change	-0.24 (-0.62,1.07)	-0.42 (-1.14,0.29)	0.58 (-0.52,1.65)	-0.15 (-0.92,0.65)	-0.65 (-1.4,0.13)	-0.004 (-0.62, 0.61)
Quality of life score change	-1.54 (-5.99,11.21)	**-5.69 (-11.69,-1.29)**	14.36 (-28.19,108.28)	1.83 (-71.27,6.34)	3.12 (-1.12,8.4)	1.08 (-35.42, 6.3)
Cup abduction angle	0.15 (-2.71,2.93)	0.18 (-2.11,2.4)	-0.03 (-2.35,2.51)	1.34 (-1.62,4.11)	0.22 (-2.17,2.62)	----
Cup anteversion angle	-0.76 (-5.04,3.43)	-1.96 (-6.08,1.92)	1.16 (-4.74,6.89)	**-6.42 (-12.16,-0.71)**	-1.82 (-6.54，2.55）	----

© 2023 Yan L et al. *JAMA Network Open.*

eTable 9. Heterogeneity Assessments

Outcomes	Design-based Q statistic, comparisons, and overall statement	Q statistic	Degree of freedom	P value
Hip score change	Design-specific decomposition of within-designs Q statistic			
	DAA vs DLA	27.37	6	0.0001
	DAA vs MIS-DLA	13.01	1	0.0003
	DAA vs MIS-PA	3.28	3	0.3499
	DAA vs PA	9.7	5	0.0842
	DLA vs MIS-ALA	0.92	2	0.632
	DLA vs MIS-DLA	7.38	3	0.0607
	MIS-ALA vs MIS-DLA	7.37	3	0.061
	MIS-ALA vs PA	2.24	1	0.1342
	MIS-PA vs PA	6.56	5	0.2556
	PA vs SuperPath	1.4	2	0.4963
	Between-designs Q statistic after detaching of single designs			
	2-incision vs MIS-ALA	45.73	15	< 0.0001
	2-incision vs MIS-PA	45.73	15	< 0.0001
	DAA vs DLA	45.95	15	< 0.0001
	DAA vs MIS-ALA	44.13	15	0.0001
	DAA vs MIS-DLA	41.16	15	0.0003
	DAA vs MIS-PA	41.95	15	0.0002
	DAA vs PA	37.64	15	0.001

© 2023 Yan L et al. *JAMA Network Open.*

Outcomes	Design-based Q statistic, comparisons, and overall statement	Q statistic	Degree of freedom	P value
	DLA vs MIS-ALA	41.98	15	0.0002
	DLA vs MIS-DLA	46.4	15	< 0.0001
	DLA vs PA	39.33	15	0.0006
	MIS-ALA vs MIS-DLA	38.65	15	0.0007
	MIS-ALA vs PA	45.86	15	< 0.0001
	MIS-DLA vs PA	45.51	15	< 0.0001
	MIS-PA vs PA	46.52	15	< 0.0001
	MIS-PA vs SuperPath	35.16	15	0.0023
	PA vs SuperPath	35.16	15	0.0023
	DAA vs MIS-ALA vs MIS-DLA	44.04	14	< 0.0001
	DLA vs MIS-DLA vs MIS-PA vs PA	38.95	13	0.0002
	MIS-ALA vs MIS-PA vs PA	45.36	14	< 0.0001
	Q statistic to assess consistency under the assumption of a full design-by-treatment interaction random effects model vs between designs Q statistic, 18.49; degree of freedom, 16; p value, 0.2960; tau.within, 2.7353; tau^2.within, 7.4820.			
Hospitalization time	Design-specific decomposition of within-designs Q statistic			
	2-incision:MIS-PA	0.23	1	0.6281
	DAA:DLA	2.13	3	0.5455
	DAA:MIS-DLA	5.09	1	0.024
	DAA:MIS-PA	0.54	2	0.7641
	DAA:PA	33.55	5	< 0.0001
	DLA:MIS-DLA	16.06	2	0.0003

© 2023 Yan L et al. *JAMA Network Open.*

Outcomes	Design-based Q statistic, comparisons, and overall statement	Q statistic	Degree of freedom	P value
	MIS-PA:PA	407.24	5	< 0.0001
	PA:SuperPath	96.18	2	< 0.0001
	Between-designs Q statistic after detaching of single designs			
	2-incision:MIS-ALA	19.41	6	0.0035
	2-incision:MIS-PA	19.41	6	0.0035
	DAA:DLA	19.9	6	0.0029
	DAA:MIS-DLA	19.78	6	0.003
	DAA:MIS-PA	4.19	6	0.6509
	DAA:PA	5.87	6	0.4381
	DLA:MIS-ALA	20.15	6	0.0026
	DLA:MIS-DLA	19.28	6	0.0037
	MIS-ALA:MIS-DLA	20.12	6	0.0026
	MIS-PA:PA	5.72	6	0.4551
	MIS-PA:SuperPath	20.19	6	0.0026
	PA:SuperPath	20.19	6	0.0026
	DAA:MIS-ALA:MIS-DLA	18.11	5	0.0028
	Q statistic to assess consistency under the assumption of a full design-by-treatment interaction random effects model vs between designs Q statistic, 10.03; degree of freedom, 7; p value, 0.0081; tau.within, 1.0502; tau^2.within, 1.1029.			
Operation time	Design-specific decomposition of within-designs Q statistic			
	2-incision vs MIS-PA	0.13	1	0.7175
	DAA vs DLA	14.96	5	0.0105
	DAA vs MIS-PA	0.03	1	0.867

© 2023 Yan L et al. *JAMA Network Open.*

Outcomes	Design-based Q statistic, comparisons, and overall statement	Q statistic	Degree of freedom	P value
	DAA vs PA	44.25	4	< 0.0001
	DLA vs MIS-ALA	3.64	2	0.1623
	DLA vs MIS-DLA	8.04	3	0.0453
	DLA vs PA	46.08	1	< 0.0001
	MIS-ALA vs MIS-DLA	1.47	1	0.2259
	MIS-ALA vs PA	1.65	1	0.1986
	MIS-PA vs PA	27.21	4	< 0.0001
	PA vs SuperPath	70.1	2	< 0.0001
	Between-designs Q statistic after detaching of single designs			
	2-incision vs MIS-ALA	177.61	15	< 0.0001
	2-incision vs MIS-PA	177.61	15	< 0.0001
	DAA vs DLA	140.4	15	< 0.0001
	DAA vs MIS-ALA	166.23	15	< 0.0001
	DAA vs MIS-DLA	162.59	15	< 0.0001
	DAA vs MIS-PA	163.01	15	< 0.0001
	DAA vs PA	76.05	15	< 0.0001
	DLA vs MIS-ALA	167.68	15	< 0.0001
	DLA vs MIS-DLA	175.68	15	< 0.0001
	DLA vs PA	122	15	< 0.0001
	MIS-ALA vs MIS-DLA	175.25	15	< 0.0001
	MIS-ALA vs MIS-PA	176.46	15	< 0.0001

© 2023 Yan L et al. *JAMA Network Open.*

Outcomes	Design-based Q statistic, comparisons, and overall statement	Q statistic	Degree of freedom	P value
	MIS-ALA vs PA	160.07	15	< 0.0001
	MIS-DLA vs MIS-PA	170.55	15	< 0.0001
	MIS-DLA vs PA	177.63	15	< 0.0001
	MIS-PA vs PA	133.25	15	< 0.0001
	MIS-PA vs SuperPath	156.55	15	< 0.0001
	PA vs SuperPath	156.55	15	< 0.0001
	DAA vs MIS-ALA vs MIS-DLA	173.06	14	< 0.0001
	DLA vs MIS-DLA vs MIS-PA vs PA	167.16	13	< 0.0001
	Q statistic to assess consistency under the assumption of a full design-by-treatment interaction random effects model vs between designs Q statistic, 22.19; degree of freedom, 16; p value, 0.1373; tau.within, 6.2965; tau^2.within, 39.6456.			
Blood loss	Design-specific decomposition of within-designs Q statistic			
	DAA vs DLA	8.37	4	0.0788
	DAA vs PA	1.92	2	0.3827
	DLA vs MIS-DLA	9.36	2	0.0093
	MIS-PA vs PA	3	5	0.7007
	PA vs SuperPath	40.06	2	< 0.0001
	Between-designs Q statistic after detaching of single designs			
	2-incision vs MIS-ALA	55.56	12	< 0.0001
	2-incision vs MIS-PA	55.56	12	< 0.0001
	DAA vs DLA	56.09	12	< 0.0001
	DAA vs MIS-ALA	55.31	12	< 0.0001

© 2023 Yan L et al. *JAMA Network Open.*

Outcomes	Design-based Q statistic, comparisons, and overall statement	Q statistic	Degree of freedom	P value
	DAA vs MIS-DLA	44.46	12	< 0.0001
	DAA vs PA	47.87	12	< 0.0001
	DLA vs MIS-ALA	46.96	12	< 0.0001
	DLA vs MIS-DLA	45.57	12	< 0.0001
	DLA vs PA	51.71	12	< 0.0001
	MIS-ALA vs MIS-DLA	54.72	12	< 0.0001
	MIS-ALA vs PA	29.35	12	0.0035
	MIS-DLA vs MIS-PA	55.82	12	< 0.0001
	MIS-DLA vs PA	54.44	12	< 0.0001
	MIS-PA vs PA	54.97	12	< 0.0001
	MIS-PA vs SuperPath	56.04	12	< 0.0001
	PA vs SuperPath	56.04	12	< 0.0001
	DAA vs MIS-ALA vs MIS-DLA	55.96	11	< 0.0001
	MIS-ALA vs MIS-PA vs PA	47.53	11	< 0.0001
	Q statistic to assess consistency under the assumption of a full design-by-treatment interaction random effects model vs between designs Q statistic, 23.12; degree of freedom, 13; p value, 0.0402; tau.within, 51.0569; tau^2.within, 2606.8023.			
Pain score change	Design-specific decomposition of within-designs Q statistic			
	DAA:DLA	7.81	2	0.0202
	DAA:MIS-DLA	32.51	1	< 0.0001
	DAA:MIS-PA	22.45	2	< 0.0001

© 2023 Yan L et al. *JAMA Network Open.*

Outcomes	Design-based Q statistic, comparisons, and overall statement	Q statistic	Degree of freedom	P value
	DAA:PA	26.54	2	< 0.0001
	DLA:MIS-ALA	4.7	2	0.0954
	MIS-PA:PA	0.12	2	0.9404
	PA:SuperPath	2.1	2	0.3502
	Between-designs Q statistic after detaching of single designs			
	DAA:DLA	42.65	6	< 0.0001
	DAA:MIS-ALA	42.55	6	< 0.0001
	DAA:MIS-DLA	15.87	6	0.0145
	DAA:MIS-PA	37.4	6	< 0.0001
	DAA:PA	34.5	6	< 0.0001
	DLA:MIS-ALA	42.66	6	< 0.0001
	DLA:PA	40.01	6	< 0.0001
	MIS-ALA:MIS-DLA	42.92	6	< 0.0001
	MIS-DLA:MIS-PA	30.68	6	< 0.0001
	MIS-DLA:PA	21.02	6	0.0018
	MIS-PA:PA	41.47	6	< 0.0001
	MIS-PA:SuperPath	40.39	6	< 0.0001
	PA:SuperPath	40.39	6	< 0.0001
	Q statistic to assess consistency under the assumption of a full design-by-treatment interaction random effects model vs between designs Q statistic, 2.74; degree of freedom, 7; p value, 0.9077; tau.within, 0.6273; tau^2.within, 0.3935.			
	Design-specific decomposition of within-designs Q statistic			

© 2023 Yan L et al. *JAMA Network Open.*

Outcomes	Design-based Q statistic, comparisons, and overall statement	Q statistic	Degree of freedom	P value
QOL score change	2-incision:MIS-PA	1.58	1	0.2086
	DAA:DLA	1.76	3	0.6248
	DAA:MIS-DLA	31.35	1	< 0.0001
	DAA:MIS-PA	1.38	1	0.2394
	DLA:MIS-ALA	0.28	1	0.5951
	MIS-PA:PA	0.07	1	0.7931
	Between-designs Q statistic after detaching of single designs			
	DAA:DLA	26.69	6	0.0002
	DAA:MIS-DLA	18.86	6	0.0044
	DAA:MIS-PA	24.46	6	0.0004
	DAA:PA	27.18	6	0.0001
	DLA:MIS-ALA	26.73	6	0.0002
	DLA:PA	27.99	6	< 0.0001
	MIS-ALA:PA		6	< 0.0001
	MIS-DLA:PA	18.8 6	6	0.0044
	MIS-PA:PA	20.58	6	0.0022
	MIS-PA:SuperPath	16.05	6	0.0135
	PA:SuperPath	16.05	6	0.0135
	MIS-ALA:MIS-PA:PA	24.79	5	0.0002
	Q statistic to assess consistency under the assumption of a full design-by-treatment interaction random effects model vs between designs Q statistic, 9.03; degree of freedom, 7; p value, 0.2508; tau.within, 0.4005; tau^2.within, 0.1604.			
	Design-specific decomposition of within-designs Q statistic			

© 2023 Yan L et al. *JAMA Network Open.*

Outcomes	Design-based Q statistic, comparisons, and overall statement	Q statistic	Degree of freedom	P value
Cup Abduction angle	DAA vs DLA	12.98	2	0.0015
	DAA vs MIS-PA	8.12	3	0.0436
	DAA vs PA	12.35	2	0.0021
	DLA vs MIS-ALA	3.97	1	0.0462
	DLA vs MIS-DLA	0.03	2	0.9873
	MIS-ALA vs MIS-DLA	0.09	1	0.7646
	MIS-PA vs PA	25.05	3	< 0.0001
	PA vs SuperPath	5.06	1	0.0245
	Between-designs Q statistic after detaching of single designs			
	DAA vs DLA	59.9	8	< 0.0001
	DAA vs MIS-ALA	53.23	8	< 0.0001
	DAA vs MIS-PA	25.93	8	0.0011
	DAA vs PA	38.08	8	< 0.0001
	DLA vs MIS-ALA	54.6	8	< 0.0001
	DLA vs MIS-DLA	59.79	8	< 0.0001
	DLA vs PA	56.79	8	< 0.0001
	MIS-ALA vs MIS-DLA	59.79	8	< 0.0001
	MIS-ALA vs MIS-PA	49.52	8	< 0.0001
	MIS-ALA vs PA	55.1	8	< 0.0001
	MIS-PA vs PA	21.01	8	0.0071
	MIS-PA vs SuperPath	59.9	8	< 0.0001

© 2023 Yan L et al. *JAMA Network Open.*

Outcomes	Design-based Q statistic, comparisons, and overall statement	Q statistic	Degree of freedom	P value
	PA vs SuperPath	59.9	8	< 0.0001
	MIS-ALA vs MIS-PA vs PA	56.03	7	< 0.0001
	Q statistic to assess consistency under the assumption of a full design-by-treatment interaction random effects model vs between designs Q statistic, 8.39; degree of freedom, 9; p value, 0.4952; tau.within, 1.9773; tau².within, 3.9097.			
Cup Anteversion angle	Design-specific decomposition of within-designs Q statistic			
	DAA vs MIS-PA	7.49	2	0.0236
	DAA vs PA	22.7	2	< 0.0001
	MIS-PA vs PA	55.82	2	< 0.0001
	PA vs SuperPath	2.03	1	0.1541
	Between-designs Q statistic after detaching of single designs			
	DAA vs MIS-ALA	41.59	3	< 0.0001
	DAA vs MIS-PA	36.81	3	< 0.0001
	DAA vs PA	93.23	3	< 0.0001
	DLA vs MIS-ALA	97.67	3	< 0.0001
	DLA vs PA	97.67	3	< 0.0001
	MIS-ALA vs PA	55.16	3	< 0.0001
	MIS-PA vs PA	33.77	3	< 0.0001
	MIS-PA vs SuperPath	94.81	3	< 0.0001
	PA vs SuperPath	94.81	3	< 0.0001
	Q statistic to assess consistency under the assumption of a full design-by-treatment interaction random effects model vs between			

© 2023 Yan L et al. *JAMA Network Open.*

Outcomes	Design-based Q statistic, comparisons, and overall statement	Q statistic	Degree of freedom	P value
	designs Q statistic, 0.19; degree of freedom, 4; p value, 0.9959; tau.within, 3.5794; tau².within, 12.8124.			
Short-term hip score	Design-specific decomposition of within-designs Q statistic			
	DAA:DLA	7.06	2	0.0293
	DAA:MIS-DLA	24.34	1	< 0.0001
	DAA:MIS-PA	1.36	3	0.7152
	DAA:PA	2.56	1	0.1096
	DLA:MIS-DLA	20.25	1	< 0.0001
	MIS-PA:PA	0	1	0.9972
	PA:SuperPath	1.29	1	0.2557
	Between-designs Q statistic after detaching of single designs			
	2-incision:MIS-ALA	128.5	8	< 0.0001
	2-incision:MIS-PA	128.5	8	< 0.0001
	DAA:DLA	106.85	8	< 0.0001
	DAA:MIS-DLA	34.34	8	< 0.0001
	DAA:MIS-PA	129.49	8	< 0.0001
	DAA:PA	96.98	8	< 0.0001
	DLA:MIS-ALA	123.76	8	< 0.0001
	DLA:MIS-DLA	45.11	8	< 0.0001
	DLA:PA	91.16	8	< 0.0001
	MIS-PA:PA	124.24	8	< 0.0001

© 2023 Yan L et al. *JAMA Network Open.*

Outcomes	Design-based Q statistic, comparisons, and overall statement	Q statistic	Degree of freedom	P value
	MIS-PA:SuperPath	125.78	8	< 0.0001
	PA:SuperPath	125.78	8	< 0.0001
	DAA:MIS-ALA:MIS-DLA	122.18	7	< 0.0001
	MIS-ALA:MIS-PA:PA	114.46	7	< 0.0001
	Q statistic to assess consistency under the assumption of a full design-by-treatment interaction random effects model vs between designs Q statistic, 11.15; degree of freedom, 9; p value, 0.2658; tau.within, 3.1519; tau^2.within, 9.9342.			
Long-term hip score	Design-specific decomposition of within-designs Q statistic			
	2-incision:MIS-PA	1.38	2	0.5023
	DAA:DLA	1.43	3	0.6995
	DAA:MIS-PA	2.4	3	0.4931
	DAA:PA	0.15	1	0.6963
	DLA:MIS-ALA	0.45	2	0.797
	MIS-ALA:MIS-DLA	5.87	2	0.0531
	MIS-PA:PA	4.45	2	0.1082
	PA:SuperPath	2.75	2	0.2531
	Between-designs Q statistic after detaching of single designs			
	2-incision:MIS-ALA	24.02	9	0.0043
	2-incision:MIS-PA	24.02	9	0.0043
	DAA:DLA	23.56	9	0.0051
	DAA:MIS-ALA	22.84	9	0.0066
	DAA:MIS-DLA	24.23	9	0.0039
	DAA:MIS-PA	21.03	9	0.0125

© 2023 Yan L et al. *JAMA Network Open.*

Outcomes	Design-based Q statistic, comparisons, and overall statement	Q statistic	Degree of freedom	P value
	DAA:PA	22.26	9	0.0081
	DLA:MIS-ALA	24.11	9	0.0041
	DLA:MIS-DLA	21.42	9	0.0109
	DLA:PA	20.6	9	0.0146
	MIS-ALA:MIS-DLA	23.58	9	0.005
	MIS-ALA:PA	23.79	9	0.0046
	MIS-DLA:PA	23.82	9	0.0046
	MIS-PA:PA	20.44	9	0.0154
	MIS-ALA:MIS-PA:PA	9.8	8	0.279
	Q statistic to assess consistency under the assumption of a full design-by-treatment interaction random effects model vs between designs Q statistic, 22.47; degree of freedom, 10; p value, 0.0129; tau.within, 0.4679; tau^2.within, 0.2189.			
Dislocation	Design-specific decomposition of within-designs Q statistic			
	2-incision:MIS-PA	1.66	1	0.1977
	DAA:DLA	3.03	1	0.0816
	DAA:MIS-PA	0.29	1	0.5887
	DAA:PA	1.56	3	0.6695
	DLA:PA	1.93	1	0.1647
	MIS-PA:PA	1.88	3	0.5968
	Between-designs Q statistic after detaching of single designs			
	DAA:DLA	0.53	2	0.7683
	DAA:MIS-PA	0.98	2	0.6141
	DAA:PA	0.57	2	0.7506

© 2023 Yan L et al. *JAMA Network Open.*

Outcomes	Design-based Q statistic, comparisons, and overall statement	Q statistic	Degree of freedom	P value
	DLA:PA	0.53	2	0.7683
	MIS-ALA:MIS-DLA	0.59	2	0.7452
	MIS-ALA:PA	0.59	2	0.7452
	MIS-DLA:PA	0.59	2	0.7452
	MIS-PA:PA	0.98	2	0.6141
	Q statistic to assess consistency under the assumption of a full design-by-treatment interaction random effects model vs between designs Q statistic, 0.96; degree of freedom, 3; p value, 0.8102; tau.within, 0.2763; tau².within, 0.0763.			
Fracture	Design-specific decomposition of within-designs Q statistic			
	2-incision:MIS-PA	0	3	1
	DAA:DLA	1.12	2	0.5725
	DAA:MIS-PA	0.72	1	0.3948
	DAA:PA	1.13	2	0.5671
	DLA:PA	0.63	1	0.429
	MIS-ALA:MIS-PA	0	1	0.971
	MIS-PA:PA	2.35	3	0.5026
	Between-designs Q statistic after detaching of single designs			
	2-incision:MIS-DLA	5.85	8	0.6637
	2-incision:MIS-PA	5.85	8	0.6637
	DAA:DLA	5.59	8	0.6934
	DAA:MIS-DLA	5.54	8	0.6988
	DAA:MIS-PA	5	8	0.7579
	DAA:PA	5.84	8	0.6652

© 2023 Yan L et al. *JAMA Network Open.*

Outcomes	Design-based Q statistic, comparisons, and overall statement	Q statistic	Degree of freedom	P value
	DLA:MIS-ALA	5.83	8	0.6659
	DLA:PA	5.47	8	0.7059
	MIS-ALA:MIS-DLA	5.78	8	0.6717
	MIS-ALA:MIS-PA	4.79	8	0.7797
	MIS-ALA:PA	5.4	8	0.714
	MIS-DLA:PA	4.33	8	0.8264
	MIS-PA:PA	5.85	8	0.6645
	DAA:MIS-ALA:MIS-DLA	2.28	7	0.9429
	Q statistic to assess consistency under the assumption of a full design-by-treatment interaction random effects model vs between designs Q statistic, 5.85; degree of freedom, 9; p value, 0.7545; tau.within, 0; tau^2.within, 0.			
Infection	Design-specific decomposition of within-designs Q statistic			
	DAA:DLA	2.55	2	0.2794
	DAA:PA	0.79	1	0.373
	DLA:MIS-ALA	0.13	1	0.7165
	MIS-ALA:MIS-DLA	0.55	1	0.4598
	MIS-PA:PA	1.65	3	0.6477
	Between-designs Q statistic after detaching of single designs			
	2-incision:MIS-DLA	1.27	3	0.7368
	2-incision:MIS-PA	1.27	3	0.7368
	DAA:DLA	1.07	3	0.7836
	DAA:MIS-PA	1.3	3	0.7282
	DAA:PA	1.41	3	0.7021

© 2023 Yan L et al. *JAMA Network Open.*

Outcomes	Design-based Q statistic, comparisons, and overall statement	Q statistic	Degree of freedom	P value
	DLA:MIS-ALA	1.21	3	0.7506
	DLA:PA	0.19	3	0.9784
	MIS-ALA:MIS-DLA	1.21	3	0.7506
	MIS-DLA:MIS-PA	1.46	3	0.6906
	MIS-PA:PA	0.94	3	0.8167
	Q statistic to assess consistency under the assumption of a full design-by-treatment interaction random effects model vs between designs Q statistic, 1.47; degree of freedom, 4; p value, 0.8319; tau.within, 0; tau^2.within, 0.			
Nerve injury	Design-specific decomposition of within-designs Q statistic			
	DAA:DLA	0.18	2	0.9141
	DAA:PA	1.16	1	0.2821
	Between-designs Q statistic after detaching of single designs			
	2-incision:MIS-DLA	1.29	2	0.5246
	2-incision:MIS-PA	1.29	2	0.5246
	DAA:DLA	2.63	2	0.2681
	DAA:MIS-ALA	2.62	2	0.2703
	DAA:MIS-DLA	0.26	2	0.8784
	DAA:MIS-PA	1.29	2	0.5246
	DLA:MIS-ALA	2.63	2	0.2681
	MIS-ALA:MIS-DLA	2.26	2	0.3231
	Q statistic to assess consistency under the assumption of a full design-by-treatment interaction random effects model vs between designs Q statistic, 2.72; degree of freedom, 3; p value, 0.4373; tau.within, 0; tau^2.within, 0.			
Reoperation	Design-specific decomposition of within-designs Q statistic			

© 2023 Yan L et al. *JAMA Network Open.*

Outcomes	Design-based Q statistic, comparisons, and overall statement	Q statistic	Degree of freedom	P value
	2-incision:MIS-PA	0.06	2	0.9722
	DAA:DLA	4.87	4	0.3008
	DAA:PA	1.05	2	0.5907
	MIS-PA:PA	1.62	3	0.6547
	Between-designs Q statistic after detaching of single designs			
	DAA:DLA	2.98	5	0.7029
	DAA:PA	2.98	5	0.7029
	DLA:MIS-ALA	2.78	5	0.7341
	DLA:PA	2.94	5	0.7097
	MIS-ALA:MIS-DLA	2.94	5	0.7094
	MIS-ALA:MIS-PA	2.91	5	0.7133
	MIS-ALA:PA	1.28	5	0.9369
	MIS-DLA:PA	2.94	5	0.7094
	MIS-PA:PA	2	5	0.849
	MIS-ALA:MIS-PA:PA	1.61	4	0.8073
	Q statistic to assess consistency under the assumption of a full design-by-treatment interaction random effects model vs between designs Q statistic, 3.01; degree of freedom, 6; p value, 0.8078; tau.within, 0; tau^2.within, 0.			
Thromboembolism	Design-specific decomposition of within-designs Q statistic			
	DAA:DLA	1.35	2	0.5083
	DAA:MIS-PA	0.97	1	0.325
	DAA:PA	0.24	1	0.6259
	MIS-PA:PA	1.13	3	0.7708

© 2023 Yan L et al. *JAMA Network Open.*

Outcomes	Design-based Q statistic, comparisons, and overall statement	Q statistic	Degree of freedom	P value
	Between-designs Q statistic after detaching of single designs			
	DAA:DLA	0.46	3	0.9284
	DAA:MIS-DLA	0.12	3	0.9894
	DAA:MIS-PA	0.43	3	0.9338
	DAA:PA	0.39	3	0.9426
	DLA:MIS-ALA	0.28	3	0.9633
	DLA:PA	0.35	3	0.9513
	MIS-ALA:MIS-DLA	0.28	3	0.9633
	MIS-DLA:PA	0.41	3	0.9385
	MIS-PA:PA	0.43	3	0.9338
	Q statistic to assess consistency under the assumption of a full design-by-treatment interaction random effects model vs between designs Q statistic, 0.46; degree of freedom, 4; p value, 0.9775; tau.within, 0; tau^2.within, 0.			
Analgesic consumption	Design-specific decomposition of within-designs Q statistic			
	DAA vs DLA	5.24	1	0.0221
	MIS-PA vs PA	0.53	1	0.466
	Between-designs Q statistic after detaching of single designs			
	DAA vs DLA	3.12	1	0.0775
	DAA vs MIS-DLA	3.12	1	0.0775
	DAA vs MIS-PA	31.63	1	< 0.0001
	DAA vs PA	31.63	1	< 0.0001
	DLA vs MIS-DLA	3.12	1	0.0775
	MIS-PA vs PA	31.63	1	< 0.0001

© 2023 Yan L et al. *JAMA Network Open.*

Outcomes	Design-based Q statistic, comparisons, and overall statement	Q statistic	Degree of freedom	P value
	Q statistic to assess consistency under the assumption of a full design-by-treatment interaction random effects model vs between designs Q statistic, 32.75; degree of freedom, 2; p value, < 0.0001; tau.within, 19.3857; tau².within, 375.8071.			
Cadence change	Design-specific decomposition of within-designs Q statistic			
	DAA vs DLA	0	1	1
	Q statistic to assess consistency under the assumption of a full design-by-treatment interaction random effects model vs between designs Q statistic, 0; degree of freedom, 0; p value, --; tau.within, 0; tau².within, 0.			
CK change	Design-specific decomposition of within-designs Q statistic			
	DAA vs DLA	10.14	2	0.0063
	MIS-ALA vs MIS-DLA	1.17	1	0.2789
	PA vs SuperPath	71.25	1	< 0.0001
	Between-designs Q statistic after detaching of single designs			
	DAA vs DLA	1.36	1	0.2436
	DAA vs PA	1.36	1	0.2436
	DLA vs MIS-ALA	0.82	1	0.3651
	DLA vs MIS-DLA	0.19	1	0.664
	MIS-ALA vs MIS-DLA	0.19	1	0.664
	Q statistic to assess consistency under the assumption of a full design-by-treatment interaction random effects model vs between designs Q statistic, 0.32; degree of freedom, 2; p value, 0.8535; tau.within, 100.9128; tau².within, 10183.3979.			
CRP change	Design-specific decomposition of within-designs Q statistic			
	DAA vs DLA	8.5	2	0.0143
	DLA vs MIS-ALA	0.07	1	0.7897

© 2023 Yan L et al. *JAMA Network Open.*

Outcomes	Design-based Q statistic, comparisons, and overall statement	Q statistic	Degree of freedom	P value
	PA vs SuperPath	50.71	1	< 0.0001
	Between-designs Q statistic after detaching of single designs			
	DAA vs DLA	0.21	2	0.8986
	DAA vs PA	0.21	2	0.8986
	DLA vs MIS-ALA	0.84	2	0.656
	MIS-ALA vs MIS-DLA	0.84	2	0.656
	MIS-PA vs PA	1.76	2	0.4154
	DLA vs MIS-DLA vs MIS-PA vs PA	0	0	--
	Q statistic to assess consistency under the assumption of a full design-by-treatment interaction random effects model vs between designs Q statistic, 0.03; degree of freedom, 3; p value, 0.9985; tau.within, 14.5227; tau².within, 210.9080.			
ESR change	Design-specific decomposition of within-designs Q statistic			
	PA vs SuperPath	1.36	1	0.2429
	Q statistic to assess consistency under the assumption of a full design-by-treatment interaction random effects model vs between designs Q statistic, 0.00; degree of freedom, 0; p value, --; tau.within, 2.1255; tau².within, 4.5177.			
Hb change	Design-specific decomposition of within-designs Q statistic			
	DAA vs DLA	2.32	1	0.1279
	DAA vs PA	0.09	1	0.7677
	DLA vs MIS-ALA	1.35	1	0.2453
	DLA vs MIS-DLA	6.95	1	0.0084
	MIS-PA vs PA	0.2	2	0.9066
	Between-designs Q statistic after detaching of single designs			

© 2023 Yan L et al. *JAMA Network Open*.

Outcomes	Design-based Q statistic, comparisons, and overall statement	Q statistic	Degree of freedom	P value
	DAA vs DLA	6.49	6	0.3704
	DAA vs MIS-DLA	3.39	6	0.7592
	DAA vs PA	6.24	6	0.3963
	DLA vs MIS-ALA	6.57	6	0.3627
	DLA vs MIS-DLA	3.4	6	0.7572
	MIS-DLA vs PA	6.47	6	0.3731
	MIS-PA vs PA	6.83	6	0.337
	DLA vs MIS-DLA vs MIS-PA vs PA	5.4	4	0.2485
	MIS-ALA vs MIS-PA vs PA	6.3	5	0.2782
	Q statistic to assess consistency under the assumption of a full design-by-treatment interaction random effects model vs between designs Q statistic, 4.48; degree of freedom, 7; p value, 0.7230; tau.within, 2.7863; tau².within, 7.7636.			
HCT change	Design-specific decomposition of within-designs Q statistic			
	DLA vs MIS-ALA	1.22	2	0.5437
	MIS-PA vs PA	0.57	1	0.4514
	Q statistic to assess consistency under the assumption of a full design-by-treatment interaction random effects model vs between designs Q statistic, 0; degree of freedom, 0; p value, --; tau.within, 0; tau².within, 0.			
IL-6 change	Design-specific decomposition of within-designs Q statistic			
	DAA vs DLA	3.36	1	0.0667
	Between-designs Q statistic after detaching of single designs			
	DAA vs DLA	0.55	1	0.4571
	DAA vs PA	0.55	1	0.4571
	MIS-PA vs PA	0.03	1	0.8556

© 2023 Yan L et al. *JAMA Network Open.*

Outcomes	Design-based Q statistic, comparisons, and overall statement	Q statistic	Degree of freedom	P value
	Q statistic to assess consistency under the assumption of a full design-by-treatment interaction random effects model vs between designs Q statistic, 0.47; degree of freedom, 2; p value, 0.7917; tau.within, 11.1435; tau².within, 124.1765.			
LLD	Design-specific decomposition of within-designs Q statistic			
	MIS-PA vs PA	0.8	1	0.3705
	Between-designs Q statistic after detaching of single designs			
	MIS-ALA vs MIS-PA	1.74	1	0.1865
	MIS-PA vs PA	0.67	1	0.4145
	MIS-ALA vs MIS-PA vs PA	0	0	--
	Q statistic to assess consistency under the assumption of a full design-by-treatment interaction random effects model vs between designs Q statistic, 2.32; degree of freedom, 2; p value, 0.3127; tau.within, 0; tau².within, 0.			
Stem alignment	Design-specific decomposition of within-designs Q statistic			
	DAA vs DLA	0.48	1	0.4878
	DAA vs PA	4.87	1	0.0272
	MIS-PA vs PA	2.7	1	0.1002
	Between-designs Q statistic after detaching of single designs			
	DAA vs DLA	4.25	3	0.2355
	DAA vs PA	4.25	3	0.2355
	DLA vs MIS-ALA	6.86	3	0.0764
	DLA vs MIS-DLA	9.91	3	0.0194
	MIS-ALA vs MIS-DLA	9.91	3	0.0194
	MIS-ALA vs MIS-PA	9.99	3	0.0186
	MIS-PA vs PA	5.62	3	0.1314

© 2023 Yan L et al. *JAMA Network Open.*

Outcomes	Design-based Q statistic, comparisons, and overall statement	Q statistic	Degree of freedom	P value
	MIS-ALA vs MIS-PA vs PA	2.24	2	0.3255
	Q statistic to assess consistency under the assumption of a full design-by-treatment interaction random effects model vs between designs Q statistic, 4.58; degree of freedom, 4; p value, 0.3331; tau.within, 0.4711; tau^2.within, 0.2219.			
Step length change	Design-specific decomposition of within-designs Q statistic			
	DAA vs DLA	0.7	1	0.4014
	DLA vs MIS-ALA	0.05	1	0.8152
	Between-designs Q statistic after detaching of single designs			
	DAA vs DLA	0.93	1	0.3346
	DAA vs MIS-DLA	0.93	1	0.3346
	DLA vs MIS-ALA	0.93	1	0.3346
	MIS-ALA vs MIS-DLA	0.93	1	0.3346
	MIS-PA vs PA	159	1	< 0.0001
	MIS-PA vs SuperPath	159	1	< 0.0001
	PA vs SuperPath	159	1	< 0.0001
	Q statistic to assess consistency under the assumption of a full design-by-treatment interaction random effects model vs between designs Q statistic, 159.93; degree of freedom, 2; p value, < 0.0001; tau.within, 0; tau^2.within, 0.			
Time up and go test result change	Design-specific decomposition of within-designs Q statistic			
	DAA vs DLA	0.55	1	0.4573
	MIS-PA vs PA	1.08	1	0.2997
	Q statistic to assess consistency under the assumption of a full design-by-treatment interaction random effects model vs between designs Q statistic, 0; degree of freedom, 0; p value, --; tau.within, 0; tau^2.within, 0.			

© 2023 Yan L et al. *JAMA Network Open.*

Outcomes	Design-based Q statistic, comparisons, and overall statement	Q statistic	Degree of freedom	P value
Volume of blood transfusion	Design-specific decomposition of within-designs Q statistic			
	DLA vs MIS-DLA 1.61	1	0.2051	0.0788
	MIS-PA vs PA 4.52	2	0.1046	0.3827
	Between-designs Q statistic after detaching of single designs			
	DAA vs MIS-DLA	3.71	3	0.2949
	DAA vs PA	3.71	3	0.2949
	DLA vs MIS-DLA	0.48	3	0.9243
	DLA vs PA	1.66	3	0.6457
	MIS-PA vs PA	5.12	3	0.1629
	DLA vs MIS-DLA vs MIS-PA vs PA	3.63	1	0.0568
	Q statistic to assess consistency under the assumption of a full design-by-treatment interaction random effects model vs between designs Q statistic, 3.32; degree of freedom, 4; p value, 0.5060; tau.within, 35.6411; tau^2.within, 1270.2852.			
Walking speed change	Design-specific decomposition of within-designs Q statistic			
	DAA vs DLA	0.8	2	0.6716
	DAA vs PA	0	1	1
	DLA vs MIS-ALA	0.05	1	0.8221
	Between-designs Q statistic after detaching of single designs			
	DAA vs DLA	2.29	2	0.3185
	DAA vs MIS-DLA	4.16	2	0.1252
	DAA vs PA	2.46	2	0.293
	DLA vs MIS-ALA	2.29	2	0.3185
	MIS-ALA vs MIS-DLA	2.29	2	0.3185

© 2023 Yan L et al. *JAMA Network Open.*

Outcomes	Design-based Q statistic, comparisons, and overall statement	Q statistic	Degree of freedom	P value
	MIS-PA vs PA	2.49	2	0.2886
	MIS-PA vs SuperPath	4.2	2	0.1225
	PA vs SuperPath	4.2	2	0.1225
	Q statistic to assess consistency under the assumption of a full design-by-treatment interaction random effects model vs between designs Q statistic, 4.21; degree of freedom, 3; p value, 0.2394; tau.within, 0; tau^2.within, 0.			

© 2023 Yan L et al. *JAMA Network Open.*

eTable 10. Results of Sensitivity Analyses

eTable 10A. Exclusion of studies with high item in the ROB

Outcome : Hip score change

**SuperPath**	NR	NR	-1.43 (-5.85,2.99)	**10.20 (1.03,19.37)**	NR	NR	NR
-0.15 (-7.75,7.44)	**2-incision**	NR	NR	0.00 (-7.35,7.35)	NR	5.00 (-6.77,16.77)	NR
0.74 (-3.82,5.30)	0.89 (-5.76,7.55)	**DAA**	0.07 (-3.15,3.28)	-1.61 (-6.01,2.80)	1.10 (-2.93,5.13)	1.46 (-4.68,7.60)	**5.28 (1.88,8.68)**
0.75 (-3.26,4.75)	0.90 (-5.68,7.48)	0.01 (-2.33,2.34)	**PA**	-0.56 (-3.42,2.31)	-1.19 (-8.42,6.05)	-1.88 (-7.47,3.70)	**6.86 (1.45,12.28)**
0.81 (-3.62,5.25)	0.96 (-5.34,7.27)	0.07 (-2.67,2.81)	0.07 (-2.34,2.47)	**MIS-PA**	-8.50 (-17.48,0.48)	2.00 (-7.47,11.47)	-1.30 (-9.73,7.13)
1.54 (-3.48,6.57)	1.70 (-5.21,8.60)	0.80 (-1.94,3.54)	0.80 (-2.34,3.93)	0.73 (-2.74,4.20)	**MIS-DLA**	0.41 (-3.90,4.72)	3.28 (-0.84,7.40)
2.37 (-2.65,7.40)	2.52 (-4.19,9.24)	1.63 (-1.33,4.59)	1.63 (-1.51,4.76)	1.56 (-1.92,5.04)	0.83 (-2.19,3.85)	**MIS-ALA**	0.93 (-3.82,5.67)
**5.16 (0.28,10.04)**	5.31 (-1.51,12.14)	**4.42 (1.94,6.90)**	**4.41 (1.52,7.31)**	**4.35 (1.08,7.62)**	**3.62 (0.77,6.46)**	2.79 (-0.20,5.78)	**DLA**

© 2023 Yan L et al. *JAMA Network Open.*

Outcome : Pain score change

**SuperPath**	NR	-0.35 (-1.15,0.45)	NR	-1.32 (-2.98,0.34)	NR	NR
-0.27 (-1.31,0.76)	**MIS-DLA**	0.31 (-1.17,1.79)	-0.55 (-1.50,0.40)	-0.22 (-1.73,1.29)	-1.26 (-2.82,0.30)	NR
-0.50 (-1.23,0.23)	-0.22 (-1.00,0.56)	**PA**	0.02 (-0.78,0.82)	0.02 (-0.87,0.90)	NR	-1.00 (-2.73,0.73)
-0.66 (-1.56,0.24)	-0.39 (-1.08,0.30)	-0.17 (-0.75,0.42)	**DAA**	-0.13 (-0.97,0.71)	-0.12 (-1.59,1.34)	-0.46 (-1.51,0.59)
-0.69 (-1.57,0.18)	-0.42 (-1.22,0.38)	-0.20 (-0.82,0.42)	-0.03 (-0.65,0.59)	**MIS-PA**	NR	NR
-1.15 (-2.34,0.03)	-0.88 (-1.84,0.07)	-0.66 (-1.62,0.31)	-0.49 (-1.34,0.36)	-0.46 (-1.46,0.54)	**MIS-ALA**	-0.05 (-0.96,0.87)
-1.21 (-2.33,-0.09)	-0.94 (-1.89,0.02)	-0.71 (-1.60,0.17)	-0.55 (-1.32,0.23)	-0.52 (-1.46,0.43)	-0.06 (-0.83,0.71)	**DLA**

© 2023 Yan L et al. *JAMA Network Open.*

Outcome : Hospitalization time

**SuperPath**	**-2.15 (-3.40,-0.90)**	NR	NR	NR	NR	NR	0.19 (-2.01,2.39)
**-1.23 (-2.34,-0.12)**	**PA**	0.44 (-0.45,1.34)	NR	NR	NR	NR	**-2.92 (-4.01,-1.83)**
-1.31 (-2.63,0.01)	-0.08 (-0.87,0.71)	**DAA**	-0.83 (-2.21,0.55)	-1.00 (-4.00,2.00)	-0.34 (-1.37,0.69)	NR	0.09 (-1.40,1.57)
-1.39 (-3.07,0.30)	-0.16 (-1.48,1.16)	-0.08 (-1.16,1.00)	**MIS-DLA**	0.04 (-1.90,1.97)	**-1.93 (-3.66,-0.20)**	NR	NR
**-1.91 (-3.74,-0.07)**	-0.68 (-2.20,0.84)	-0.60 (-1.97,0.77)	-0.52 (-1.93,0.89)	**MIS-ALA**	0.20 (-1.99,2.39)	-0.70 (-3.05,1.65)	NR
**-2.03 (-3.61,-0.46)**	-0.80 (-1.98,0.37)	-0.72 (-1.62,0.17)	-0.65 (-1.79,0.49)	-0.13 (-1.51,1.26)	**DLA**	NR	NR
**-2.59 (-4.35,-0.83)**	-1.36 (-2.84,0.12)	-1.28 (-2.77,0.20)	-1.20 (-2.93,0.52)	-0.68 (-2.34,0.97)	-0.56 (-2.21,1.10)	**2-incision**	-0.09 (-1.52,1.35)
**-2.67 (-3.93,-1.41)**	**-1.44 (-2.29,-0.59)**	**-1.36 (-2.31,-0.41)**	-1.28 (-2.68,0.11)	-0.76 (-2.30,0.77)	-0.64 (-1.91,0.64)	-0.08 (-1.37,1.21)	**MIS-PA**

© 2023 Yan L et al. *JAMA Network Open.*

Outcome : Operation time

**PA**	0.96 (-6.01,7.93)	**-12.68 (-25.00,-0.35)**	-3.88 (-19.23,11.47)	-7.51 (-17.64,2.61)	**-17.10 (-26.09,-8.12)**	**-18.90 (-25.49,-12.30)**	NR
-1.53 (-7.07,4.01)	**MIS-PA**	-7.52 (-19.76,4.72)	-7.30 (-23.81,9.21)	-3.20 (-24.72,18.32)	-1.50 (-17.59,14.59)	-14.20 (-30.81,2.41)	**-22.28 (-34.81,-9.74)**
**-7.34 (-13.91,-0.78)**	-5.82 (-13.09,1.46)	**MIS-DLA**	-2.03 (-14.23,10.17)	-6.55 (-15.70,2.61)	NR	-8.85 (-20.72,3.03)	NR
**-10.27 (-17.12,-3.42)**	**-8.74 (-16.29,-1.19)**	-2.93 (-10.24,4.39)	**MIS-ALA**	-0.28 (-10.38,9.83)	NR	-3.72 (-15.69,8.26)	-13.00 (-46.16,20.16)
**-13.16 (-18.94,-7.38)**	**-11.63 (-18.64,-4.62)**	-5.81 (-12.16,0.54)	-2.89 (-9.46,3.68)	**DLA**	NR	0.17 (-6.66,7.01)	NR
**-13.76 (-21.71,-5.81)**	**-12.23 (-21.14,-3.32)**	-6.41 (-16.48,3.66)	-3.49 (-13.75,6.77)	-0.60 (-10.25,9.05)	**SuperPath**	NR	NR
**-15.49 (-20.54,-10.44)**	**-13.96 (-20.52,-7.40)**	**-8.15 (-14.65,-1.64)**	-5.22 (-11.94,1.49)	-2.33 (-7.52,2.85)	-1.73 (-10.99,7.52)	**DAA**	NR
**-23.74 (-36.54,-10.93)**	**-22.21 (-33.97,-10.45)**	**-16.39 (-29.97,-2.82)**	**-13.47 (-26.93,-0.01)**	-10.58 (-23.98,2.83)	-9.98 (-24.60,4.64)	-8.25 (-21.46,4.97)	**2-incision**

© 2023 Yan L et al. *JAMA Network Open.*

Outcome : Blood loss

**MIS-ALA**	30.83 (-109.82, 171.48)	-79.00 (-282.91, 124.91)	NR	**-120.59 (-224.73, -16.44)**	-135.00 (-362.61, 92.61)	-15.16 (-232.17, 201.84)	-85.33 (-215.92, 45.26)
-13.59 (-94.73, 67.55)	**MIS-DLA**	8.10 (-108.76, 124.96)	NR	1.00 (-132.10, 134.10)	NR	-9.93 (-127.50, 107.64)	**-318.03 (-487.34, -148.71)**
-18.01 (-97.15, 61.13)	-4.42 (-76.21, 67.38)	**MIS-PA**	26.66 (-91.58, 144.90)	**-58.50 (-111.45, -5.55)**	-46.00 (-194.11, 102.11)	NR	NR
-29.61 (-123.62, 64.41)	-16.01 (-106.03, 74.01)	-11.59 (-82.17, 58.98)	**SuperPath**	-26.69 (-99.72, 46.34)	NR	NR	NR
-70.89 (-142.15, 0.37)	-57.30 (-124.27, 9.68)	**-52.88 (-99.11, -6.65)**	-41.29 (-104.72, 22.15)	**PA**	NR	-45.55 (-117.45, 26.34)	8.00 (-144.81, 160.81)
-85.13 (-221.15, 50.89)	-71.53 (-211.82, 68.76)	-67.12 (-193.47, 59.24)	-55.52 (-197.75, 86.71)	-14.24 (-145.05, 116.58)	**2-incision**	NR	NR
**-82.51 (-159.64, -5.38)**	-68.91 (-139.44, 1.61)	-64.49 (-133.94, 4.95)	-52.90 (-137.06, 31.25)	-11.62 (-68.19, 44.96)	2.62 (-136.16, 141.40)	**DAA**	**-67.81 (-130.11, -5.52)**
**-147.20 (-226.30, -68.10)**	**-133.60 (-210.75, -56.46)**	**-129.18 (-206.68, -51.69)**	**-117.59 (-208.88, -26.30)**	**-76.30 (-143.22, -9.39)**	-62.07 (-204.13, 79.99)	**-64.69 (-118.59, -10.79)**	**DLA**

© 2023 Yan L et al. *JAMA Network Open.*

Outcome : Quality of life socre change

**MIS-DLA**	NR	-0.03 (-0.63,0.57)	NR	NR	**2.91 (2.06,3.76)**	NR	NR
-0.14 (-2.33,2.04)	**MIS-ALA**	1.07 (-1.52,3.65)	5.00 (-4.73,14.73)	NR	NR	1.24 (-2.55,5.03)	NR
**0.69 (0.17,1.21)**	0.83 (-1.30,2.96)	**PA**	-1.05 (-3.14,1.05)	2.87 (-0.45,6.19)	0.10 (-0.51,0.71)	3.00 (-5.66,11.66)	NR
1.19 (-0.55,2.92)	1.33 (-1.34,3.99)	0.50 (-1.17,2.17)	**MIS-PA**	**-20.25 (-31.46,-9.04)**	-0.92 (-3.78,1.93)	NR	1.87 (-1.33,5.06)
1.73 (-1.50,4.96)	1.87 (-1.96,5.71)	1.04 (-2.14,4.23)	0.55 (-2.99,4.08)	**SuperPath**	NR	NR	NR
**1.47 (0.87,2.07)**	1.61 (-0.55,3.77)	**0.78 (0.27,1.29)**	0.28 (-1.41,1.98)	-0.26 (-3.49,2.96)	**DAA**	0.01 (-0.34,0.35)	NR
**1.47 (0.78,2.16)**	1.62 (-0.55,3.78)	**0.79 (0.17,1.40)**	0.29 (-1.44,2.02)	-0.26 (-3.50,2.99)	0.01 (-0.34,0.35)	**DLA**	NR
3.05 (-0.59,6.69)	3.19 (-0.97,7.36)	2.36 (-1.24,5.97)	1.87 (-1.33,5.06)	1.32 (-3.45,6.09)	1.58 (-2.04,5.20)	1.58 (-2.06,5.21)	**2-incision**

© 2023 Yan L et al. *JAMA Network Open.*

Outcome : Cup abduction angle

**PA**	-0.59 (-2.74,1.55)	1.12 (-2.54,4.79)	-0.10 (-4.40,4.20)	0.62 (-2.17,3.41)	NR	NR	-3.17 (-6.72,0.38)
0.01 (-1.70,1.72)	**MIS-PA**	-0.73 (-4.75,3.30)	NR	-0.69 (-3.38,1.99)	NR	NR	-1.00 (-5.79,3.79)
0.00 (-2.25,2.25)	-0.01 (-2.38,2.37)	**MIS-ALA**	-1.37 (-4.74,2.01)	0.40 (-3.94,4.74)	-0.30 (-6.91,6.31)	0.05 (-3.38,3.49)	NR
-0.07 (-2.41,2.27)	-0.08 (-2.59,2.44)	-0.07 (-2.21,2.07)	**DLA**	-0.69 (-3.77,2.40)	NR	-0.92 (-3.65,1.81)	NR
-0.17 (-2.05,1.70)	-0.18 (-2.11,1.75)	-0.17 (-2.43,2.08)	-0.10 (-2.31,2.10)	**DAA**	NR	NR	NR
-0.30 (-7.28,6.68)	-0.31 (-7.33,6.72)	-0.30 (-6.91,6.31)	-0.23 (-7.17,6.72)	-0.13 (-7.11,6.86)	**2-incision**	NR	NR
-0.59 (-3.55,2.38)	-0.59 (-3.68,2.50)	-0.59 (-3.09,1.92)	-0.52 (-2.81,1.78)	-0.41 (-3.32,2.49)	-0.29 (-7.36,6.78)	**MIS-DLA**	NR
-2.40 (-5.32,0.52)	-2.41 (-5.47,0.65)	-2.40 (-5.97,1.17)	-2.33 (-5.97,1.31)	-2.23 (-5.55,1.10)	-2.10 (-9.61,5.41)	-1.81 (-5.88,2.25)	**SuperPath**

© 2023 Yan L et al. *JAMA Network Open.*

Outcome : Cup anteversion angle

**2-incision**	NR	NR	NR	NR	-1.90 (-10.55,6.75)	NR	NR
-1.08 (-10.46,8.30)	**DAA**	NR	-1.14 (-4.51,2.22)	-0.40 (-3.77,2.96)	-0.10 (-5.30,5.10)	NR	NR
-0.90 (-11.28,9.48)	0.18 (-6.59,6.95)	**MIS-DLA**	NR	NR	-1.00 (-6.72,4.72)	NR	NR
-1.65 (-11.16,7.86)	-0.57 (-3.18,2.04)	-0.75 (-7.70,6.20)	**MIS-PA**	-0.34 (-3.48,2.81)	NR	-1.00 (-6.17,4.17)	NR
-1.75 (-11.06,7.56)	-0.67 (-3.15,1.81)	-0.85 (-7.53,5.83)	-0.10 (-2.49,2.29)	**PA**	-0.30 (-5.39,4.79)	0.20 (-3.63,4.02)	-0.90 (-6.13,4.33)
-1.90 (-10.55,6.75)	-0.82 (-4.44,2.80)	-1.00 (-6.72,4.72)	-0.25 (-4.20,3.69)	-0.15 (-3.60,3.30)	**MIS-ALA**	NR	0.52 (-5.29,6.33)
-1.94 (-11.75,7.86)	-0.86 (-4.67,2.95)	-1.04 (-8.40,6.31)	-0.29 (-3.73,3.15)	-0.19 (-3.38,3.00)	-0.04 (-4.66,4.57)	**SuperPath**	NR
-2.08 (-11.76,7.59)	-1.00 (-5.63,3.63)	-1.18 (-8.36,5.99)	-0.43 (-5.15,4.29)	-0.33 (-4.51,3.85)	-0.18 (-4.51,4.15)	-0.14 (-5.37,5.09)	**DLA**

© 2023 Yan L et al. *JAMA Network Open.*

eTable 10B. Exclusion of studies with fewer than 50 participants

Outcome : Hip score change

**MIS-PA**	-0.00 (-6.67,6.67)	0.44 (-2.18,3.07)	2.00 (-1.44,5.45)	**-8.50 (-16.93,-0.07)**	2.00 (-6.95,10.95)	NR	-1.30 (-9.15,6.55)
-0.00 (-6.67,6.67)	**2-incision**	NR	NR	NR	NR	NR	NR
0.61 (-1.62,2.84)	0.61 (-6.42,7.64)	**PA**	-0.22 (-3.37,2.93)	-1.36 (-8.18,5.46)	-0.14 (-4.05,3.77)	1.30 (-3.19,5.79)	**6.90 (1.95,11.85)**
0.81 (-1.60,3.21)	0.81 (-6.28,7.89)	0.19 (-1.94,2.32)	**DAA**	0.93 (-2.58,4.43)	1.52 (-4.12,7.15)	NR	**4.27 (1.39,7.15)**
1.55 (-1.52,4.62)	1.55 (-5.78,8.89)	0.94 (-1.82,3.71)	0.75 (-1.62,3.12)	**MIS-DLA**	-2.27 (-6.95,2.41)	NR	**3.71 (0.67,6.74)**
1.58 (-1.54,4.70)	1.58 (-5.78,8.94)	0.97 (-1.72,3.66)	0.78 (-1.88,3.44)	0.03 (-2.81,2.86)	**MIS-ALA**	NR	0.94 (-3.46,5.33)
1.91 (-3.10,6.93)	1.91 (-6.43,10.25)	1.30 (-3.19,5.79)	1.11 (-3.86,6.07)	0.36 (-4.91,5.63)	0.33 (-4.90,5.56)	**SuperPath**	NR
**4.81 (1.94,7.69)**	4.81 (-2.45,12.07)	**4.20 (1.67,6.73)**	**4.01 (1.90,6.12)**	**3.26 (0.94,5.58)**	**3.23 (0.55,5.91)**	2.90 (-2.25,8.05)	**DLA**

© 2023 Yan L et al. *JAMA Network Open.*

Outcome : pain score change

**MIS-DLA**	NR	0.31 (-1.22,1.84)	-0.22 (-1.78,1.34)	-0.55 (-1.53,0.44)	-1.26 (-2.87,0.35)	NR
0.11 (-1.20,1.42)	**SuperPath**	-0.38 (-1.41,0.64)	NR	NR	NR	NR
-0.28 (-1.09,0.54)	-0.38 (-1.41,0.64)	**PA**	0.02 (-0.89,0.93)	0.02 (-0.81,0.85)	NR	-1.00 (-2.77,0.77)
-0.36 (-1.20,0.48)	-0.47 (-1.70,0.76)	-0.08 (-0.76,0.60)	**MIS-PA**	0.12 (-0.75,0.99)	NR	NR
-0.38 (-1.10,0.34)	-0.49 (-1.68,0.71)	-0.10 (-0.72,0.51)	-0.02 (-0.67,0.64)	**DAA**	-0.12 (-1.64,1.39)	-0.76 (-1.80,0.29)
-0.91 (-1.92,0.09)	-1.02 (-2.48,0.43)	-0.64 (-1.67,0.39)	-0.56 (-1.63,0.52)	-0.54 (-1.45,0.37)	**MIS-ALA**	-0.16 (-1.29,0.98)
**-1.13 (-2.13,-0.14)**	-1.24 (-2.62,0.13)	-0.86 (-1.78,0.06)	-0.77 (-1.76,0.21)	-0.76 (-1.56,0.04)	-0.22 (-1.11,0.68)	**DLA**

© 2023 Yan L et al. *JAMA Network Open.*

Outcome : Hospitalization time

**SuperPath**	**-3.76 (-5.26,-2.26)**	NR	NR	NR	NR	NR	NR
**-3.76 (-5.26,-2.26)**	**PA**	0.56 (-0.26,1.37)	NR	NR	NR	NR	**-2.86 (-3.87,-1.85)**
**-4.00 (-5.66,-2.33)**	-0.24 (-0.95,0.48)	**DAA**	-0.80 (-2.08,0.48)	-1.00 (-3.89,1.89)	-0.34 (-1.28,0.61)	NR	0.07 (-1.04,1.18)
**-4.27 (-6.21,-2.33)**	-0.51 (-1.73,0.72)	-0.27 (-1.27,0.72)	**MIS-DLA**	1.00 (-2.13,4.13)	-0.89 (-2.16,0.38)	NR	NR
**-4.59 (-6.97,-2.21)**	-0.83 (-2.67,1.01)	-0.59 (-2.29,1.11)	-0.32 (-2.08,1.44)	**MIS-ALA**	0.20 (-1.83,2.23)	NR	NR
**-4.60 (-6.45,-2.74)**	-0.84 (-1.93,0.25)	-0.60 (-1.42,0.22)	-0.33 (-1.31,0.65)	-0.01 (-1.62,1.61)	**DLA**	NR	NR
**-5.31 (-7.47,-3.15)**	-1.55 (-3.10,0.00)	-1.31 (-2.87,0.25)	-1.04 (-2.89,0.81)	-0.72 (-3.03,1.59)	-0.71 (-2.48,1.05)	**2-incision**	-0.09 (-1.40,1.23)
**-5.39 (-7.11,-3.68)**	**-1.64 (-2.45,-0.82)**	**-1.40 (-2.24,-0.55)**	-1.13 (-2.43,0.18)	-0.81 (-2.70,1.09)	-0.80 (-1.98,0.38)	-0.09 (-1.40,1.23)	**MIS-PA**

© 2023 Yan L et al. *JAMA Network Open.*

Outcome : Operation time

**MIS-PA**	-3.59 (-10.84,3.66)	-7.37 (-19.09,4.36)	-7.30 (-23.09,8.49)	NR	-3.20 (-24.17,17.77)	**-13.76 (-24.40,-3.12)**	**-22.27 (-34.33,-10.21)**
-1.44 (-7.07,4.20)	**PA**	-12.50 (-24.32,-0.69)	-1.20 (-11.71,9.31)	-8.87 (-19.05,1.31)	-7.36 (-17.06,2.35)	**-21.27 (-28.21,-14.34)**	NR
-6.48 (-13.35,0.39)	-5.04 (-11.19,1.10)	**MIS-DLA**	-2.19 (-13.87,9.49)	NR	**-8.87 (-16.46,-1.28)**	-8.72 (-20.08,2.64)	NR
**-9.25 (-16.34,-2.16)**	**-7.81 (-13.97,-1.66)**	-2.77 (-9.47,3.93)	**MIS-ALA**	NR	-0.26 (-9.98,9.45)	-3.65 (-15.12,7.82)	NR
-10.31 (-21.94,1.33)	-8.87 (-19.05,1.31)	-3.82 (-15.71,8.07)	-1.06 (-12.95,10.84)	**SuperPath**	NR	NR	NR
**-13.82 (-20.53,-7.10)**	**-12.38 (-17.96,-6.81)**	**-7.34 (-13.02,-1.66)**	-4.57 (-10.69,1.55)	-3.51 (-15.12,8.09)	**DLA**	0.32 (-6.99,7.63)	NR
**-16.41 (-22.54,-10.29)**	**-14.98 (-19.95,-10.01)**	**-9.93 (-16.01,-3.86)**	**-7.16 (-13.41,-0.92)**	-6.11 (-17.44,5.22)	-2.60 (-7.80,2.61)	**DAA**	NR
**-22.27 (-34.33,-10.21)**	**-20.83 (-34.15,-7.52)**	**-15.79 (-29.67,-1.91)**	-13.02 (-27.01,0.97)	-11.96 (-28.72,4.79)	-8.45 (-22.26,5.35)	-5.86 (-19.38,7.67)	**2-incision**

© 2023 Yan L et al. *JAMA Network Open.*

Outcome : Blood loss

**SuperPath**	NR	NR	NR	NR	**-83.84 (-163.24, -4.44)**	NR	NR
-11.44 (-112.58, 89.70)	**MIS-DLA**	-30.69 (-167.53, 106.15)	8.10 (-103.26, 119.46)	NR	1.00 (-127.30, 129.30)	-9.37 (-122.09, 103.35)	**-234.62 (-330.04, -139.19)**
-11.05 (-118.11, 96.00)	0.39 (-76.70, 77.47)	**MIS-ALA**	-79.00 (-279.81, 121.81)	NR	**-122.35 (-223.28, -21.42)**	-15.00 (-230.14, 200.14)	-85.33 (-211.03, 40.37)
-21.74 (-116.66, 73.18)	-10.30 (-80.59, 59.99)	-10.69 (-93.85, 72.47)	**MIS-PA**	-46.00 (-189.81, 97.81)	-54.82 (-111.35, 1.71)	NR	NR
-67.74 (-240.05, 104.57)	-56.30 (-216.37, 103.77)	-56.69 (-222.81, 109.43)	-46.00 (-189.81, 97.81)	**2-incision**	NR	NR	NR
**-83.84 (-163.24, -4.44)**	**-72.40 (-135.05, -9.76)**	**-72.79 (-144.59, -0.99)**	**-62.10 (-114.11, -10.09)**	-16.10 (-169.02, 136.82)	**PA**	-33.68 (-109.75, 42.38)	8.00 (-140.64, 156.64)
-93.27 (-191.65, 5.10)	**-81.83 (-146.28, -17.39)**	**-82.22 (-159.99, -4.46)**	-71.53 (-144.78, 1.72)	-25.53 (-186.92, 135.85)	-9.43 (-67.51, 48.65)	**DAA**	-45.66 (-115.52, 24.20)
**-153.62 (-256.25, -50.98)**	**-142.18 (-206.41, -77.95)**	**-142.56 (-218.90, -66.23)**	**-131.87 (-209.40, -54.35)**	-85.87 (-249.24, 77.49)	**-69.77 (-134.80, -4.74)**	**-60.34 (-116.46, -4.22)**	**DLA**

© 2023 Yan L et al. *JAMA Network Open.*

Outcome : Quality of life socre change

**MIS-DLA**	NR	-0.03 (-0.59,0.53)	NR	**2.91 (2.08,3.73)**	NR	NR	NR
-0.07 (-2.27,2.12)	**MIS-ALA**	1.06 (-1.52,3.64)	5.00 (-4.73,14.73)	NR	0.83 (-3.09,4.75)	NR	NR
**0.66 (0.18,1.15)**	0.74 (-1.41,2.89)	**PA**	-1.04 (-3.13,1.04)	0.10 (-0.46,0.66)	3.00 (-5.66,11.66)	NR	2.87 (-0.44,6.18)
0.68 (-1.05,2.42)	0.75 (-1.93,3.44)	0.02 (-1.66,1.70)	**MIS-PA**	-0.92 (-3.77,1.93)	NR	1.86 (-1.33,5.06)	NR
**1.40 (0.84,1.97)**	1.48 (-0.69,3.65)	**0.74 (0.26,1.22)**	0.72 (-0.98,2.42)	**DAA**	0.03 (-0.29,0.35)	NR	NR
**1.43 (0.78,2.08)**	1.50 (-0.68,3.68)	**0.77 (0.19,1.34)**	0.75 (-0.98,2.48)	0.03 (-0.29,0.35)	**DLA**	NR	NR
2.54 (-1.09,6.18)	2.62 (-1.55,6.79)	1.88 (-1.73,5.49)	1.86 (-1.33,5.06)	1.14 (-2.48,4.76)	1.12 (-2.52,4.75)	**2-incision**	NR
**3.53 (0.18,6.88)**	3.61 (-0.34,7.56)	2.87 (-0.44,6.18)	2.85 (-0.86,6.57)	2.13 (-1.22,5.48)	2.10 (-1.26,5.47)	0.99 (-3.91,5.89)	**SuperPath**

© 2023 Yan L et al. *JAMA Network Open.*

Outcome : Cup abduction angle

**PA**	-0.63 (-2.59,1.33)	1.10 (-2.30,4.50)	-0.10 (-3.97,3.77)	-0.90 (-5.42,3.62)	NR	-1.05 (-4.03,1.93)
-0.07 (-1.70,1.55)	**MIS-PA**	-0.83 (-4.63,2.96)	NR	NR	NR	-1.05 (-3.16,1.07)
-0.25 (-2.36,1.85)	-0.18 (-2.34,1.98)	**MIS-ALA**	-1.36 (-4.47,1.75)	NR	-0.20 (-4.31,3.91)	0.40 (-3.52,4.32)
-0.41 (-2.50,1.68)	-0.33 (-2.51,1.84)	-0.15 (-2.14,1.83)	**DLA**	NR	-0.92 (-3.43,1.59)	-1.01 (-3.34,1.31)
-0.90 (-5.42,3.62)	-0.83 (-5.62,3.97)	-0.65 (-5.63,4.34)	-0.49 (-5.47,4.48)	**SuperPath**	NR	NR
-1.09 (-3.95,1.77)	-1.02 (-3.93,1.90)	-0.84 (-3.42,1.75)	-0.68 (-2.89,1.53)	-0.19 (-5.54,5.16)	**MIS-DLA**	NR
-1.06 (-2.83,0.71)	-0.99 (-2.65,0.68)	-0.81 (-2.84,1.22)	-0.65 (-2.48,1.17)	-0.16 (-5.01,4.69)	0.03 (-2.68,2.74)	**DAA**

© 2023 Yan L et al. *JAMA Network Open.*

Outcome : Cup anteversion angle

**DAA**	NR	NR	-1.00 (-4.72,2.73)	-0.87 (-5.37,3.64)	-0.10 (-5.94,5.74)	NR
0.32 (-6.21,6.86)	**SuperPath**	NR	NR	-1.10 (-6.86,4.66)	NR	NR
0.11 (-7.44,7.66)	-0.21 (-9.61,9.19)	**MIS-DLA**	NR	NR	-1.00 (-7.31,5.31)	NR
-0.74 (-3.77,2.29)	-1.06 (-7.53,5.40)	-0.85 (-8.61,6.90)	**MIS-PA**	-0.26 (-3.76,3.24)	NR	NR
-0.78 (-3.86,2.31)	-1.10 (-6.86,4.66)	-0.89 (-8.31,6.54)	-0.04 (-2.97,2.90)	**PA**	-0.30 (-6.04,5.44)	-0.90 (-6.76,4.96)
-0.89 (-5.03,3.25)	-1.21 (-8.18,5.75)	-1.00 (-7.31,5.31)	-0.15 (-4.65,4.35)	-0.11 (-4.02,3.79)	**MIS-ALA**	0.52 (-5.87,6.91)
-1.08 (-6.36,4.20)	-1.40 (-8.82,6.02)	-1.19 (-9.13,6.75)	-0.34 (-5.71,5.03)	-0.30 (-4.98,4.37)	-0.19 (-5.00,4.62)	**DLA**

© 2023 Yan L et al. *JAMA Network Open.*

eTable 10C. Exclusion of studies with follow-up time <1year

Outcome : Hip score change

**MIS-PA**	-0.00 (-6.58,6.58)	1.92 (-1.29,5.13)	1.68 (-2.20,5.56)	NR	2.00 (-6.89,10.89)	NR	NR
-0.40 (-6.15,5.36)	**2-incision**	NR	NR	NR	5.00 (-6.31,16.31)	NR	NR
1.66 (-1.04,4.36)	2.06 (-4.12,8.23)	**PA**	2.19 (-1.75,6.12)	1.50 (-2.44,5.44)	-1.88 (-6.93,3.17)	6.00 (-7.43,19.43)	NR
1.88 (-0.99,4.75)	2.28 (-3.91,8.46)	0.22 (-2.53,2.97)	**DAA**	NR	2.10 (-4.28,8.48)	2.88 (-2.53,8.29)	**4.71 (1.61,7.81)**
3.16 (-1.62,7.94)	3.56 (-3.77,10.88)	1.50 (-2.44,5.44)	1.28 (-3.53,6.09)	**SuperPath**	NR	NR	NR
3.43 (-0.12,6.97)	3.82 (-2.45,10.10)	1.77 (-1.52,5.06)	1.55 (-1.36,4.45)	0.27 (-4.87,5.40)	**MIS-ALA**	0.81 (-3.09,4.70)	0.94 (-3.41,5.29)
**5.03 (0.91,9.15)**	5.43 (-1.28,12.14)	3.37 (-0.54,7.29)	3.15 (-0.24,6.55)	1.87 (-3.68,7.43)	1.61 (-1.51,4.73)	**MIS-DLA**	-2.80 (-11.14,5.54)
**5.53 (1.83,9.24)**	5.93 (-0.59,12.46)	**3.87 (0.32,7.43)**	**3.66 (1.01,6.30)**	2.37 (-2.93,7.68)	2.11 (-0.95,5.17)	0.50 (-3.16,4.17)	**DLA**

© 2023 Yan L et al. *JAMA Network Open.*

Outcome : Pain score change

**SuperPath**	-0.34 (-0.93,0.25)	NR	NR	NR	NR	NR
-0.34 (-0.93,0.25)	**PA**	-0.31 (-1.46,0.84)	-0.45 (-1.62,0.72)	0.02 (-0.68,0.72)	NR	NR
-0.38 (-1.32,0.55)	-0.04 (-0.77,0.68)	**MIS-DLA**	0.13 (-0.88,1.14)	-0.22 (-1.41,0.97)	**-1.26 (-2.51,-0.01)**	NR
-0.46 (-1.34,0.42)	-0.12 (-0.77,0.53)	-0.07 (-0.72,0.57)	**DAA**	-0.24 (-0.87,0.40)	-0.12 (-1.25,1.00)	-0.71 (-1.46,0.04)
-0.54 (-1.36,0.28)	-0.20 (-0.77,0.38)	-0.15 (-0.83,0.52)	-0.08 (-0.61,0.45)	**MIS-PA**	NR	NR
**-1.09 (-2.16,-0.02)**	-0.75 (-1.64,0.15)	-0.70 (-1.51,0.10)	-0.63 (-1.31,0.05)	-0.55 (-1.37,0.27)	**MIS-ALA**	-0.04 (-0.77,0.70)
**-1.15 (-2.21,-0.09)**	-0.81 (-1.69,0.08)	-0.76 (-1.59,0.07)	**-0.69 (-1.32,-0.06)**	-0.61 (-1.41,0.19)	-0.06 (-0.68,0.56)	**DLA**

© 2023 Yan L et al. *JAMA Network Open.*

Outcome : Hospitalization time

**SuperPath**	**-2.28 (-4.04,-0.52)**	NR	NR	NR	NR	NR	NR
**-2.28 (-4.04,-0.52)**	**PA**	NR	0.40 (-1.30,2.09)	NR	NR	NR	**-4.06 (-5.77,-2.34)**
-2.50 (-5.51,0.52)	-0.22 (-2.67,2.23)	**MIS-DLA**	-0.06 (-3.00,2.88)	-0.50 (-3.73,2.73)	-3.00 (-6.32,0.32)	NR	NR
**-3.18 (-5.44,-0.93)**	-0.91 (-2.33,0.51)	-0.69 (-2.76,1.38)	**DAA**	NR	-0.41 (-2.13,1.31)	NR	0.10 (-2.02,2.22)
**-3.74 (-6.82,-0.66)**	-1.46 (-3.99,1.06)	-1.24 (-3.55,1.07)	-0.56 (-2.82,1.71)	**MIS-ALA**	0.20 (-2.86,3.26)	**-0.70 (-3.87,2.47)**	NR
**-3.91 (-6.60,-1.22)**	-1.63 (-3.67,0.40)	-1.41 (-3.50,0.67)	-0.72 (-2.25,0.80)	-0.17 (-2.34,2.00)	**DLA**	NR	NR
**-4.76 (-7.63,-1.89)**	**-2.49 (-4.76,-0.22)**	-2.27 (-5.02,0.48)	-1.58 (-3.80,0.64)	-1.02 (-3.45,1.40)	-0.86 (-3.34,1.62)	**2-incision**	-0.09 (-2.17,2.00)
**-4.99 (-7.25,-2.72)**	**-2.71 (-4.14,-1.28)**	**-2.49 (-4.93,-0.05)**	**-1.80 (-3.31,-0.29)**	-1.25 (-3.67,1.17)	-1.08 (-3.13,0.97)	-0.22 (-2.11,1.66)	**MIS-PA**

© 2023 Yan L et al. *JAMA Network Open.*

Outcome : operation time

**MIS-PA**	-5.85 (-14.45,2.74)	-1.37 (-17.25,14.51)	NR	NR	-14.20 (-31.12,2.72)	NR	**-22.28 (-35.01,-9.54)**
-2.01 (-9.18,5.15)	**PA**	-6.00 (-21.92,9.92)	-3.88 (-19.55,11.79)	**-27.00 (-44.31,-9.69)**	**-18.46 (-27.18,-9.75)**	**-17.12 (-26.29,-7.95)**	NR
**-11.31 (-20.55,-2.06)**	**-9.29 (-17.54,-1.05)**	**MIS-DLA**	2.33 (-9.97,14.63)	1.44 (-17.64,20.52)	-1.54 (-17.43,14.35)	NR	NR
**-12.22 (-21.98,-2.46)**	**-10.20 (-18.45,-1.96)**	-0.91 (-9.31,7.49)	**MIS-ALA**	-0.28 (-10.55,9.99)	2.10 (-14.27,18.47)	NR	-13.00 (-46.31,20.31)
**-14.95 (-24.95,-4.96)**	**-12.94 (-21.32,-4.56)**	-3.65 (-12.69,5.40)	-2.73 (-10.51,5.04)	**DLA**	-3.38 (-14.49,7.72)	NR	NR
**-17.14 (-25.62,-8.67)**	**-15.13 (-21.76,-8.51)**	-5.84 (-14.21,2.53)	-4.93 (-13.07,3.22)	-2.19 (-9.99,5.61)	**DAA**	NR	NR
**-19.14 (-30.77,-7.50)**	**-17.12 (-26.29,-7.95)**	-7.83 (-20.16,4.50)	-6.92 (-19.25,5.42)	-4.18 (-16.61,8.24)	-1.99 (-13.31,9.32)	**SuperPath**	NR
**-22.65 (-34.61,-10.69)**	**-20.64 (-34.22,-7.06)**	-11.35 (-25.99,3.30)	-10.43 (-25.06,4.19)	-7.70 (-22.73,7.33)	-5.51 (-19.72,8.71)	-3.52 (-19.90,12.87)	**2-incision**

© 2023 Yan L et al. *JAMA Network Open.*

Outcome : Blood loss

**MIS-ALA**	-79.00 (-305.77, 147.77)	28.00 (-149.61, 205.61)	NR	-0.00 (-266.96, 266.96)	-111.48 (-237.70, 14.74)	-135.00 (-383.30, 113.30)	-85.33 (-249.34, 78.68)
-28.96 (-131.38, 73.46)	**MIS-PA**	-8.10 (-161.40, 145.20)	NR	NR	-55.04 (-134.77, 24.69)	-46.00 (-224.27, 132.27)	NR
-46.51 (-151.82, 58.79)	-17.55 (-116.29, 81.19)	**MIS-DLA**	NR	-2.50 (-159.22, 154.22)	1.00 (-165.01, 167.01)	NR	NR
-64.43 (-193.40, 64.54)	-35.48 (-153.40, 82.45)	-17.92 (-148.92, 113.08)	**SuperPath**	NR	-17.47 (-110.87, 75.93)	NR	NR
-84.47 (-189.12, 20.19)	-55.51 (-161.35, 50.33)	-37.96 (-140.66, 64.75)	-20.03 (-147.56, 107.50)	**DAA**	51.95 (-66.64, 170.55)	NR	-24.54 (-157.80, 108.72)
-81.91 (-170.85, 7.03)	-52.95 (-124.95, 19.05)	-35.39 (-127.25, 56.46)	-17.47 (-110.87, 75.93)	2.56 (-84.28, 89.40)	**PA**	NR	8.00 (-174.19, 190.19)
-95.38 (-255.19, 64.42)	-66.42 (-215.36, 82.52)	-48.87 (-218.64, 120.90)	-30.95 (-213.84, 151.95)	-10.91 (-183.35, 161.52)	-13.48 (-170.72, 143.77)	**2-incision**	NR
-93.33 (-203.29, 16.62)	-64.37 (-185.59, 56.84)	-46.82 (-170.79, 77.15)	-28.90 (-169.29, 111.49)	-8.87 (-110.37, 92.64)	-11.43 (-116.24, 93.39)	2.05 (-178.02, 182.11)	**DLA**

© 2023 Yan L et al. *JAMA Network Open.*

Outcome : Quality of life socre change

**MIS-ALA**	1.04 (-1.49,3.57)	NR	5.00 (-4.71,14.71)	NR	1.23 (-2.52,4.98)	NR	NR
1.03 (-1.05,3.12)	**PA**	0.03 (-0.09,0.15)	-2.38 (-7.04,2.27)	0.10 (-0.05,0.25)	NR	NR	2.87 (-0.40,6.14)
1.06 (-1.03,3.15)	0.03 (-0.09,0.15)	**MIS-DLA**	NR	3.26 (-3.58,10.10)	NR	NR	NR
1.35 (-1.76,4.46)	0.32 (-2.06,2.70)	0.29 (-2.09,2.68)	**MIS-PA**	-0.90 (-3.71,1.91)	NR	1.86 (-1.31,5.03)	NR
1.13 (-0.96,3.22)	0.10 (-0.05,0.25)	0.07 (-0.12,0.26)	-0.22 (-2.60,2.16)	**DAA**	0.01 (-0.08,0.10)	NR	NR
1.14 (-0.95,3.23)	0.11 (-0.06,0.28)	0.08 (-0.13,0.29)	-0.21 (-2.59,2.17)	0.01 (-0.08,0.10)	**DLA**	NR	NR
3.21 (-1.23,7.65)	2.18 (-1.79,6.14)	2.15 (-1.82,6.12)	1.86 (-1.31,5.03)	2.08 (-1.88,6.04)	2.07 (-1.90,6.03)	**2-incision**	NR
**3.90 (0.02,7.78)**	2.87 (-0.40,6.14)	2.84 (-0.43,6.11)	2.55 (-1.49,6.59)	2.77 (-0.50,6.04)	2.76 (-0.51,6.03)	0.69 (-4.45,5.83)	**SuperPath**

© 2023 Yan L et al. *JAMA Network Open.*

Outcome : Cup abduction angle

**MIS-ALA**	0.06 (-3.73,3.85)	-1.38 (-5.11,2.36)	-0.30 (-7.28,6.68)	0.40 (-4.49,5.29)	-1.15 (-5.17,2.87)	-5.00 (-11.73,1.73)	NR
-0.46 (-3.71,2.79)	**MIS-DLA**	1.00 (-4.58,6.58)	NR	NR	NR	NR	NR
-0.60 (-3.27,2.07)	-0.14 (-3.77,3.50)	**DLA**	NR	-1.68 (-6.80,3.44)	0.10 (-4.76,4.96)	NR	NR
-0.30 (-7.28,6.68)	0.16 (-7.54,7.87)	0.30 (-7.18,7.78)	**2-incision**	NR	NR	NR	NR
-0.90 (-3.79,2.00)	-0.43 (-4.55,3.69)	-0.30 (-3.36,2.76)	-0.60 (-8.16,6.96)	**DAA**	-2.29 (-6.34,1.76)	0.61 (-2.39,3.60)	NR
-1.29 (-4.06,1.49)	-0.82 (-4.87,3.22)	-0.69 (-3.66,2.29)	-0.99 (-8.50,6.53)	-0.39 (-2.80,2.02)	**PA**	-0.56 (-2.94,1.82)	-3.20 (-7.09,0.70)
-1.34 (-4.40,1.73)	-0.87 (-5.13,3.38)	-0.74 (-4.02,2.54)	-1.04 (-8.66,6.59)	-0.44 (-2.79,1.91)	-0.05 (-2.13,2.03)	**MIS-PA**	NR
-4.48 (-9.27,0.30)	-4.02 (-9.64,1.60)	-3.89 (-8.79,1.02)	-4.18 (-12.65,4.28)	-3.59 (-8.17,0.99)	-3.20 (-7.09,0.70)	-3.15 (-7.56,1.27)	**SuperPath**

© 2023 Yan L et al. *JAMA Network Open.*

Outcome : Cup anteversion angle

**2-incision**	NR	NR	NR	NR	NR	-1.90 (-11.80,8.00)	NR
-1.12 (-12.10,9.86)	**PA**	0.14 (-4.98,5.27)	NR	-0.90 (-8.00,6.20)	0.17 (-4.03,4.37)	-0.30 (-7.30,6.70)	-2.54 (-8.15,3.08)
-0.98 (-13.09,11.14)	0.14 (-4.98,5.27)	**SuperPath**	NR	NR	NR	NR	NR
-0.90 (-13.31,11.51)	0.22 (-8.63,9.07)	0.08 (-10.15,10.31)	**MIS-DLA**	NR	NR	-1.00 (-8.48,6.48)	NR
-1.72 (-13.17,9.73)	-0.60 (-6.23,5.03)	-0.74 (-8.36,6.87)	-0.82 (-10.25,8.61)	**DLA**	NR	-0.52 (-8.07,7.03)	NR
-1.81 (-13.10,9.48)	-0.69 (-4.22,2.84)	-0.83 (-7.06,5.39)	-0.91 (-10.15,8.33)	-0.09 (-6.54,6.36)	**MIS-PA**	NR	0.78 (-3.65,5.20)
-1.90 (-11.80,8.00)	-0.78 (-5.52,3.96)	-0.92 (-7.90,6.06)	-1.00 (-8.48,6.48)	-0.18 (-5.93,5.57)	-0.09 (-5.52,5.34)	**MIS-ALA**	0.10 (-6.99,7.19)
-1.99 (-13.09,9.11)	-0.87 (-4.64,2.89)	-1.02 (-7.38,5.35)	-1.09 (-10.10,7.92)	-0.27 (-6.64,6.09)	-0.18 (-3.82,3.45)	-0.09 (-5.12,4.93)	**DAA**

© 2023 Yan L et al. *JAMA Network Open.*

eTable 10D. League table with fixed-effect model

Outcome : Hip score change

**2-incision**	NR	5.00 (-5.22,15.22)	0.00 (-4.46,4.46)	NR	NR	NR	NR
0.72 (-3.55,5.00)	**MIS-DLA**	-1.23 (-2.62,0.15)	**8.50 (1.68,15.32)**	1.97 (-3.56,7.50)	NR	**1.38 (0.75,2.00)**	**4.37 (3.56,5.18)**
0.80 (-3.53,5.13)	0.07 (-1.05,1.20)	**MIS-ALA**	-2.00 (-9.45,5.45)	-0.25 (-2.72,2.22)	NR	-1.76 (-5.63,2.10)	1.00 (-2.31,4.31)
0.80 (-3.30,4.89)	0.08 (-1.38,1.53)	0.00 (-1.71,1.71)	**MIS-PA**	-0.10 (-1.87,1.66)	-10.20 (-17.27,-3.13)	**2.49 (0.58,4.40)**	-1.30 (-7.38,4.78)
1.14 (-3.12,5.41)	0.42 (-0.73,1.58)	0.35 (-1.06,1.76)	0.35 (-1.01,1.71)	**PA**	2.00 (-0.25,4.25)	-0.66 (-2.06,0.74)	**7.15 (3.67,10.63)**
1.99 (-2.75,6.74)	1.27 (-1.15,3.69)	1.20 (-1.36,3.75)	1.20 (-1.28,3.67)	0.85 (-1.30,3.00)	**SuperPath**	NR	NR
1.85 (-2.41,6.10)	**1.12 (0.54,1.71)**	1.05 (-0.14,2.24)	1.05 (-0.33,2.42)	0.70 (-0.37,1.77)	-0.15 (-2.53,2.23)	**DAA**	**3.97 (1.98,5.96)**
**5.12 (0.81,9.44)**	**4.40 (3.67,5.13)**	**4.33 (3.05,5.60)**	**4.32 (2.75,5.90)**	**3.98 (2.69,5.27)**	**3.13 (0.64,5.61)**	**3.28 (2.41,4.14)**	**DLA**

© 2023 Yan L et al. *JAMA Network Open.*

Outcome : Pain score change

**MIS-DLA**	**-1.04 (-1.17,-0.90)**	NR	**-1.26 (-2.12,-0.40)**	0.31 (-0.39,1.01)	NR	-0.22 (-0.98,0.54)
**-0.94 (-1.07,-0.81)**	**DAA**	NR	-0.12 (-0.79,0.54)	**-0.45 (-0.62,-0.29)**	**-0.37 (-0.60,-0.14)**	**-0.72 (-0.95,-0.48)**
**-0.97 (-1.27,-0.68)**	-0.04 (-0.31,0.23)	**SuperPath**	NR	**-0.27 (-0.51,-0.04)**	NR	**-1.32 (-2.34,-0.30)**
**-1.23 (-1.62,-0.83)**	-0.29 (-0.67,0.09)	-0.25 (-0.72,0.21)	**MIS-ALA**	NR	-0.01 (-0.52,0.50)	NR
**-1.29 (-1.48,-1.10)**	**-0.35 (-0.51,-0.20)**	**-0.32 (-0.54,-0.09)**	-0.06 (-0.47,0.35)	**PA**	-1.00 (-2.14,0.14)	0.02 (-0.43,0.48)
**-1.33 (-1.58,-1.08)**	**-0.39 (-0.61,-0.17)**	**-0.35 (-0.70,-0.01)**	-0.10 (-0.48,0.28)	-0.04 (-0.30,0.22)	**DLA**	NR
**-1.51 (-1.74,-1.27)**	**-0.57 (-0.77,-0.37)**	**-0.53 (-0.85,-0.22)**	-0.28 (-0.71,0.15)	-0.22 (-0.45,0.01)	-0.18 (-0.48,0.12)	**MIS-PA**

© 2023 Yan L et al. *JAMA Network Open*.

Outcome : Hospitalization time

**DAA**	-0.20 (-0.62,0.23)	-0.19 (-0.37,0.01)	-1.00 (-3.26,1.26)	NR	-0.04 (-0.29,0.21)	NR	**-0.59 (-0.67,-0.51)**
-0.06 (-0.41,0.30)	**MIS-DLA**	-0.42 (-1.10,0.25)	-0.15 (-1.38,1.08)	NR	NR	NR	NR
**-0.20 (-0.38,-0.02)**	-0.14 (-0.51,0.23)	**DLA**	-0.20 (-1.15,0.75)	NR	NR	NR	NR
-0.25 (-0.90,0.41)	-0.19 (-0.88,0.51)	-0.05 (-0.70,0.61)	**MIS-ALA**	-0.70 (-1.98,0.58)	NR	NR	NR
-0.41 (-0.76,-0.06)	-0.35 (-0.84,0.14)	-0.21 (-0.60,0.18)	-0.16 (-0.86,0.53)	**2-incision**	-0.10 (-0.44,0.24)	NR	NR
**-0.48 (-0.60,-0.35)**	**-0.42 (-0.79,-0.04)**	**-0.27 (-0.49,-0.06)**	-0.23 (-0.89,0.43)	-0.06 (-0.39,0.26)	**MIS-PA**	-0.19 (-1.17,0.79)	0.03 (-0.09,0.15)
**-0.47 (-0.72,-0.23)**	-0.41 (-0.84,0.02)	-0.27 (-0.57,0.03)	-0.23 (-0.92,0.47)	-0.06 (-0.47,0.35)	0.00 (-0.25,0.26)	**SuperPath**	-0.09 (-0.33,0.15)
**-0.55 (-0.63,-0.47)**	**-0.49 (-0.85,-0.13)**	**-0.35 (-0.54,-0.15)**	-0.30 (-0.96,0.35)	-0.14 (-0.48,0.21)	-0.07 (-0.18,0.04)	-0.08 (-0.31,0.15)	**PA**

© 2023 Yan L et al. *JAMA Network Open.*

Outcome : Operation time

**MIS-PA**	**-3.54 (-4.53,-2.55)**	-3.87 (-9.21,1.48)	-7.30 (-15.32,0.72)	-1.50 (-8.61,5.61)	-3.20 (-19.16,12.76)	**-13.58 (-17.51,-9.65)**	**-22.13 (-29.35,-14.92)**
**-2.53 (-3.46,-1.59)**	**PA**	**-8.68 (-14.12,-3.23)**	-1.78 (-5.90,2.34)	**-16.67 (-19.39,-13.95)**	-0.97 (-5.46,3.52)	**-20.58 (-21.67,-19.49)**	NR
**-8.58 (-10.94,-6.22)**	**-6.05 (-8.32,-3.79)**	**MIS-DLA**	-3.24 (-6.66,0.17)	NR	**-12.21 (-15.93,-8.49)**	**-5.21 (-10.41,-0.01)**	NR
**-11.51 (-13.99,-9.03)**	**-8.98 (-11.36,-6.61)**	**-2.93 (-5.45,-0.41)**	**MIS-ALA**	NR	0.06 (-5.64,5.75)	-1.85 (-7.73,4.03)	-13.00 (-42.85,16.85)
**-16.94 (-19.60,-14.27)**	**-14.41 (-16.95,-11.87)**	**-8.36 (-11.75,-4.96)**	**-5.43 (-8.90,-1.96)**	**SuperPath**	NR	NR	NR
**-19.71 (-21.36,-18.07)**	**-17.19 (-18.61,-15.77)**	**-11.13 (-13.42,-8.85)**	**-8.20 (-10.68,-5.73)**	-2.78 (-5.69,0.13)	**DLA**	0.28 (-0.92,1.49)	NR
**-20.81 (-22.12,-19.50)**	**-18.28 (-19.28,-17.28)**	**-12.23 (-14.47,-9.98)**	**-9.30 (-11.69,-6.91)**	**-3.87 (-6.60,-1.14)**	-1.09 (-2.22,0.03)	**DAA**	NR
**-22.26 (-29.28,-15.25)**	**-19.74 (-26.81,-12.66)**	**-13.68 (-21.06,-6.30)**	**-10.75 (-18.15,-3.36)**	-5.33 (-12.83,2.17)	-2.55 (-9.74,4.65)	-1.45 (-8.58,5.67)	**2-incision**

© 2023 Yan L et al. *JAMA Network Open.*

Outcome : Blood loss

**SuperPath**	NR	-26.66 (-67.77, 14.45)	NR	**-89.88 (-114.83, -64.94)**	NR	NR	NR
-21.37 (-68.39, 25.65)	**MIS-ALA**	-79.00 (-250.14, 92.14)	29.36 (-65.55, 124.28)	**-155.97 (-219.73, -92.20)**	-135.00 (-333.79, 63.79)	-13.38 (-210.91, 184.15)	**-85.33 (-154.35, -16.31)**
**-29.99 (-56.41, -3.57)**	-8.62 (-53.95, 36.72)	**MIS-PA**	-8.10 (-45.05, 28.85)	**-61.29 (-88.59, -33.98)**	-46.00 (-144.21, 52.21)	NR	NR
**-44.64 (-77.39, -11.90)**	-23.27 (-68.36, 21.82)	-14.65 (-41.90, 12.59)	**MIS-DLA**	1.00 (-72.65, 74.65)	NR	-3.86 (-52.83, 45.11)	**-205.89 (-252.95, -158.84)**
**-88.66 (-110.74, -66.57)**	**-67.29 (-109.34, -25.23)**	**-58.67 (-80.01, -37.32)**	**-44.01 (-70.36, -17.66)**	**PA**	NR	**-37.85 (-52.77, -22.92)**	8.00 (-97.16, 113.16)
-91.76 (-183.54, 0.02)	-70.39 (-165.68, 24.90)	-61.77 (-150.26, 26.73)	-47.12 (-138.90, 44.67)	-3.10 (-93.33, 87.13)	**2-incision**	NR	NR
**-119.46 (-145.37, -93.55)**	**-98.09 (-140.56, -55.61)**	**-89.47 (-113.89, -65.05)**	**-74.81 (-101.22, -48.41)**	**-30.80 (-44.93, -16.68)**	-27.70 (-118.59, 63.19)	**DAA**	**-70.15 (-95.76, -44.55)**
**-189.73 (-222.38, -157.07)**	**-168.36 (-211.51, -125.20)**	**-159.74 (-190.31, -129.16)**	**-145.08 (-174.46, -115.71)**	**-101.07 (-125.87, -76.27)**	**-97.97 (-190.41, -5.53)**	**-70.27 (-92.48, -48.06)**	**DLA**

© 2023 Yan L et al. *JAMA Network Open*.

Outcome : Quality of life socre change

**MIS-ALA**	NR	1.04 (-1.49,3.57)	5.00 (-4.71,14.71)	NR	1.23 (-2.52,4.98)	NR	NR
0.98 (-1.10,3.07)	**MIS-DLA**	-0.03 (-0.11,0.05)	NR	**2.90 (2.29,3.52)**	NR	NR	NR
1.00 (-1.08,3.09)	0.02 (-0.06,0.11)	**PA**	-1.03 (-3.07,1.01)	0.10 (-0.02,0.22)	3.00 (-5.64,11.64)	2.87 (-0.40,6.14)	NR
1.27 (-1.34,3.88)	0.29 (-1.34,1.92)	0.27 (-1.36,1.89)	**MIS-PA**	-0.90 (-3.71,1.91)	NR	**-20.25 (-31.44,-9.06)**	1.86 (-1.31,5.03)
1.21 (-0.88,3.29)	**0.23 (0.08,0.37)**	**0.20 (0.08,0.32)**	-0.06 (-1.69,1.56)	**DAA**	0.01 (-0.05,0.06)	NR	NR
1.21 (-0.87,3.30)	**0.23 (0.08,0.38)**	**0.21 (0.08,0.34)**	-0.06 (-1.68,1.57)	0.01 (-0.05,0.06)	**DLA**	NR	NR
2.08 (-1.69,5.84)	1.10 (-2.04,4.24)	1.08 (-2.06,4.21)	0.81 (-2.67,4.28)	0.87 (-2.27,4.01)	0.87 (-2.28,4.01)	**SuperPath**	NR
3.13 (-0.98,7.23)	2.15 (-1.41,5.71)	2.13 (-1.43,5.69)	1.86 (-1.31,5.03)	1.92 (-1.64,5.48)	1.92 (-1.65,5.48)	1.05 (-3.65,5.75)	**2-incision**

© 2023 Yan L et al. *JAMA Network Open.*

Outcome : Cup abduction angle

**PA**	-0.10 (-1.52,1.32)	0.89 (-1.11,2.88)	NR	NR	**-2.64 (-3.03,-2.26)**	**-2.92 (-4.99,-0.85)**	-0.83 (-1.84,0.17)
**-1.16 (-1.95,-0.36)**	**DLA**	1.26 (-0.52,3.03)	NR	-0.88 (-2.15,0.39)	NR	NR	**-1.65 (-2.55,-0.75)**
**-1.65 (-2.63,-0.67)**	-0.49 (-1.46,0.47)	**MIS-ALA**	-0.30 (-5.52,4.92)	-0.05 (-1.75,1.65)	1.61 (-1.10,4.33)	NR	0.40 (-1.13,1.93)
-1.95 (-7.26,3.36)	-0.79 (-6.10,4.51)	-0.30 (-5.52,4.92)	**2-incision**	NR	NR	NR	NR
**-1.92 (-3.17,-0.66)**	-0.76 (-1.84,0.32)	-0.27 (-1.46,0.92)	0.03 (-5.32,5.38)	**MIS-DLA**	NR	NR	NR
**-2.19 (-2.54,-1.83)**	**-1.03 (-1.83,-0.22)**	-0.54 (-1.52,0.45)	-0.24 (-5.54,5.07)	-0.27 (-1.53,0.99)	**MIS-PA**	-1.00 (-3.54,1.54)	**-1.82 (-2.47,-1.16)**
**-3.03 (-4.64,-1.41)**	**-1.87 (-3.65,-0.08)**	-1.37 (-3.25,0.50)	-1.07 (-6.62,4.47)	-1.11 (-3.14,0.92)	-0.84 (-2.46,0.78)	**SuperPath**	NR
**-2.84 (-3.40,-2.29)**	**-1.69 (-2.40,-0.97)**	**-1.19 (-2.13,-0.25)**	-0.89 (-6.19,4.41)	-0.92 (-2.14,0.29)	**-0.66 (-1.18,-0.13)**	0.18 (-1.50,1.87)	**DAA**

© 2023 Yan L et al. *JAMA Network Open.*

Outcome : Cup anteversion angle

**DAA**	**-2.81 (-3.45,-2.17)**	NR	NR	NR	-0.10 (-1.29,1.09)	**-3.63 (-4.40,-2.86)**	NR
**-1.18 (-1.68,-0.68)**	**MIS-PA**	NR	-1.00 (-2.01,0.01)	NR	NR	**-4.15 (-4.61,-3.70)**	NR
-2.12 (-9.16,4.93)	-0.94 (-7.97,6.10)	**2-incision**	NR	NR	-1.90 (-8.91,5.11)	NR	NR
**-3.00 (-3.73,-2.27)**	**-1.82 (-2.44,-1.20)**	-0.88 (-7.94,6.17)	**SuperPath**	NR	NR	**-0.98 (-1.65,-0.30)**	NR
**-3.02 (-5.75,-0.28)**	-1.84 (-4.56,0.89)	-0.90 (-8.40,6.60)	-0.02 (-2.78,2.75)	**MIS-DLA**	-1.00 (-3.67,1.67)	NR	NR
**-4.02 (-4.64,-3.39)**	**-2.84 (-3.41,-2.26)**	-1.90 (-8.91,5.11)	**-1.02 (-1.74,-0.29)**	-1.00 (-3.67,1.67)	**MIS-ALA**	0.30 (-0.19,0.79)	0.52 (-2.33,3.37)
**-4.34 (-4.84,-3.84)**	**-3.16 (-3.55,-2.77)**	-2.23 (-9.25,4.80)	**-1.34 (-1.91,-0.77)**	-1.33 (-4.03,1.38)	-0.33 (-0.78,0.13)	**PA**	-0.90 (-2.18,0.38)
**-4.95 (-6.22,-3.68)**	**-3.77 (-5.00,-2.54)**	-2.83 (-9.95,4.29)	**-1.95 (-3.25,-0.65)**	-1.93 (-4.87,1.00)	-0.93 (-2.16,0.29)	-0.61 (-1.78,0.56)	**DLA**

© 2023 Yan L et al. *JAMA Network Open.*

eTable 10E. Included studies where all surgeries were carried out by a single surgeon

Outcome1: Hip score change

**SuperPath**	NR	NR	-1.37 (-6.53,3.79)	NR	**10.20 (0.01,20.40)**	NR	NR
0.35 (-5.43,6.14)	**DAA**	NR	1.16 (-4.46,6.78)	1.36 (-5.67,8.39)	-3.32 (-9.16,2.52)	3.74 (-2.79,10.28)	**6.31 (1.26,11.36)**
-0.13 (-9.00,8.73)	-0.49 (-8.35,7.38)	**2-incision**	NR	5.00 (-7.59,17.59)	0.00 (-8.59,8.59)	NR	NR
0.84 (-3.82,5.50)	0.49 (-3.26,4.24)	0.98 (-6.83,8.78)	**PA**	-1.81 (-10.23,6.61)	1.25 (-3.17,5.67)	-3.90 (-13.49,5.69)	3.30 (-5.78,12.38)
1.21 (-5.53,7.95)	0.85 (-3.90,5.61)	1.34 (-6.65,9.33)	0.37 (-4.69,5.42)	**MIS-ALA**	NR	6.20 (-6.08,18.48)	2.10 (-7.49,11.69)
1.57 (-3.85,6.99)	1.22 (-2.72,5.15)	1.70 (-5.60,9.00)	0.73 (-2.85,4.31)	0.36 (-5.02,5.75)	**MIS-PA**	-8.50 (-18.52,1.52)	-1.30 (-10.84,8.24)
2.78 (-3.99,9.55)	2.43 (-2.00,6.86)	2.91 (-5.67,11.50)	1.94 (-3.19,7.07)	1.57 (-4.19,7.34)	1.21 (-4.08,6.51)	**MIS-DLA**	1.88 (-3.56,7.32)
4.75 (-1.67,11.16)	**4.39 (0.61,8.18)**	4.88 (-3.41,13.17)	3.90 (-0.75,8.56)	3.54 (-1.69,8.76)	3.18 (-1.66,8.01)	1.97 (-2.46,6.39)	**DLA**

© 2023 Yan L et al. *JAMA Network Open.*

Outcome : Pain score change

**MIS-ALA**	0.12 (-0.63,0.88)	-0.35 (-1.07,0.36)	NR	NR	NR	NR
-0.12 (-0.72,0.48)	**DAA**	0.20 (-0.49,0.89)	NR	-0.13 (-0.68,0.42)	**-0.69 (-1.09,-0.29)**	**-1.00 (-1.45,-0.55)**
-0.13 (-0.71,0.46)	-0.01 (-0.58,0.57)	**DLA**	NR	NR	NR	NR
-0.40 (-1.15,0.36)	-0.28 (-0.74,0.18)	-0.27 (-1.01,0.46)	**SuperPath**	NR	-0.30 (-0.62,0.02)	**-1.32 (-2.40,-0.24)**
-0.43 (-1.20,0.33)	-0.31 (-0.79,0.16)	-0.31 (-1.05,0.44)	-0.03 (-0.68,0.61)	**MIS-DLA**	NR	-0.22 (-1.06,0.62)
**-0.76 (-1.46,-0.06)**	**-0.64 (-1.00,-0.27)**	-0.63 (-1.31,0.05)	**-0.36 (-0.67,-0.05)**	-0.33 (-0.91,0.26)	**PA**	-0.10 (-1.03,0.83)
**-1.07 (-1.77,-0.36)**	**-0.95 (-1.31,-0.58)**	**-0.94 (-1.62,-0.26)**	**-0.67 (-1.18,-0.15)**	**-0.63 (-1.16,-0.11)**	-0.31 (-0.76,0.14)	**MIS-PA**

© 2023 Yan L et al. *JAMA Network Open.*

Outcome : Hospitalization time

**SuperPath**	NR	NR	NR	0.19 (-1.34,1.72)	NR	NR	**-1.83 (-2.66,-1.00)**
-0.27 (-1.57,1.03)	**MIS-DLA**	0.28 (-0.87,1.43)	1.00 (-1.82,3.82)	NR	NR	**-1.88 (-3.19,-0.56)**	NR
-0.76 (-1.71,0.18)	-0.49 (-1.40,0.42)	**DAA**	-1.00 (-3.55,1.55)	0.04 (-1.28,1.36)	NR	-0.17 (-0.88,0.54)	-0.70 (-1.40,0.01)
-0.86 (-2.27,0.55)	-0.59 (-1.93,0.75)	-0.10 (-1.24,1.04)	**MIS-ALA**	NR	-0.70 (-2.44,1.04)	0.20 (-1.31,1.71)	NR
**-0.97 (-1.92,-0.01)**	-0.69 (-1.90,0.51)	-0.20 (-1.04,0.63)	-0.10 (-1.35,1.14)	**MIS-PA**	0.09 (-0.81,0.99)	NR	0.12 (-1.07,1.30)
-1.02 (-2.25,0.21)	-0.75 (-2.13,0.63)	-0.26 (-1.36,0.84)	-0.16 (-1.43,1.11)	-0.06 (-0.90,0.78)	**2-incision**	NR	NR
**-1.13 (-2.25,-0.01)**	-0.86 (-1.78,0.07)	-0.36 (-1.00,0.27)	-0.26 (-1.39,0.86)	-0.16 (-1.17,0.85)	-0.10 (-1.31,1.10)	**DLA**	NR
**-1.49 (-2.24,-0.73)**	**-1.22 (-2.31,-0.12)**	**-0.72 (-1.36,-0.09)**	-0.62 (-1.87,0.63)	-0.52 (-1.31,0.27)	-0.46 (-1.56,0.63)	-0.36 (-1.24,0.53)	**PA**

© 2023 Yan L et al. *JAMA Network Open.*

Outcome : Operation time

**PA**	-9.63 (-21.24,1.97)	**-23.80 (-42.95,-4.65)**	**-17.01 (-25.24,-8.78)**	-3.88 (-17.91,10.15)	**-19.43 (-28.90,-9.97)**	**-20.61 (-33.24,-7.99)**	NR
**-9.46 (-16.68,-2.25)**	**MIS-PA**	-7.26 (-18.62,4.11)	-1.50 (-16.34,13.34)	-7.30 (-22.60,8.00)	-13.40 (-27.17,0.37)	-3.20 (-23.81,17.41)	**-22.27 (-34.00,-10.53)**
**-13.21 (-21.27,-5.14)**	-3.74 (-11.61,4.12)	**MIS-DLA**	NR	-8.00 (-22.10,6.10)	-8.63 (-19.64,2.38)	-4.35 (-14.24,5.55)	NR
**-15.59 (-22.99,-8.20)**	-6.13 (-15.20,2.94)	-2.38 (-12.71,7.95)	**SuperPath**	NR	NR	NR	NR
**-16.10 (-23.91,-8.28)**	-6.64 (-14.69,1.42)	-2.89 (-11.10,5.32)	-0.51 (-10.73,9.71)	**MIS-ALA**	-3.60 (-14.73,7.52)	4.20 (-11.94,20.34)	-13.00 (-45.57,19.57)
**-18.88 (-25.35,-12.40)**	**-9.41 (-16.63,-2.19)**	-5.67 (-12.62,1.28)	-3.29 (-12.60,6.03)	-2.78 (-10.00,4.44)	**DAA**	-2.78 (-9.43,3.87)	NR
**-20.39 (-27.57,-13.20)**	**-10.92 (-18.78,-3.07)**	**-7.18 (-14.24,-0.12)**	-4.80 (-14.62,5.03)	-4.29 (-12.02,3.45)	-1.51 (-6.94,3.92)	**DLA**	NR
**-31.43 (-44.40,-18.45)**	**-21.96 (-33.05,-10.88)**	**-18.22 (-31.55,-4.88)**	**-15.84 (-29.98,-1.69)**	**-15.33 (-28.47,-2.18)**	-12.55 (-25.49,0.39)	-11.04 (-24.34,2.26)	**2-incision**

© 2023 Yan L et al. *JAMA Network Open.*

Outcome : Blood loss

**MIS-ALA**	60.00 (-412.53, 532.53)	NR	NR	-135.00 (-361.35, 91.35)	-15.09 (-231.25, 201.07)	**-228.82 (-360.11, -97.53)**	NR
-90.34 (-220.76, 40.08)	**MIS-DLA**	8.10 (-106.28, 122.48)	NR	NR	-9.67 (-125.06, 105.72)	NR	**-600.00 (-880.51, -319.49)**
-98.57 (-212.62, 15.48)	-8.23 (-96.49, 80.04)	**MIS-PA**	26.66 (-89.14, 142.46)	-46.00 (-192.16, 100.16)	NR	**-80.18 (-153.02, -7.34)**	NR
**-120.61 (-241.04, -0.18)**	-30.27 (-135.45, 74.91)	-22.04 (-96.40, 52.31)	**SuperPath**	NR	NR	-27.54 (-99.18, 44.11)	NR
-141.75 (-288.57, 5.06)	-51.41 (-202.67, 99.85)	-43.19 (-170.47, 84.10)	-21.14 (-164.48, 122.19)	**2-incision**	NR	NR	NR
**-163.15 (-276.79, -49.51)**	-72.81 (-159.48, 13.86)	-64.58 (-144.19, 15.03)	-42.54 (-130.14, 45.06)	-21.40 (-165.09, 122.30)	**DAA**	33.68 (-44.60, 111.95)	**-76.61 (-140.61, -12.62)**
**-166.79 (-271.22, -62.37)**	-76.45 (-166.69, 13.79)	**-68.23 (-127.17, -9.28)**	-46.18 (-109.25, 16.89)	-25.04 (-159.34, 109.26)	-3.64 (-67.00, 59.71)	**PA**	8.00 (-142.92, 158.92)
**-246.98 (-370.81, -123.15)**	**-156.64 (-255.77, -57.50)**	**-148.41 (-240.73, -56.10)**	**-126.37 (-225.46, -27.28)**	-105.23 (-256.56, 46.11)	**-83.83 (-142.52, -25.14)**	**-80.19 (-158.57, -1.81)**	**DLA**

© 2023 Yan L et al. *JAMA Network Open.*

Outcome : Quality of life socre change

**SuperPath**	NR	NR	NR	-2.87 (-12.18,6.44)	NR	**20.25 (6.06,34.44)**	NR
-0.85 (-17.17,15.46)	**MIS-DLA**	NR	3.26 (-7.82,14.34)	NR	NR	NR	NR
1.79 (-9.33,12.92)	2.65 (-11.96,17.26)	**MIS-ALA**	NR	0.54 (-8.56,9.64)	2.28 (-6.04,10.61)	NR	NR
2.41 (-9.57,14.38)	3.26 (-7.82,14.34)	0.61 (-8.92,10.14)	**DAA**	NR	-0.00 (-8.72,8.72)	4.92 (-6.00,15.84)	NR
3.29 (-4.70,11.28)	4.14 (-10.57,18.85)	1.49 (-6.58,9.56)	0.88 (-8.80,10.56)	**PA**	NR	-1.14 (-7.93,5.65)	NR
3.28 (-8.84,15.39)	4.13 (-9.43,17.69)	1.48 (-6.06,9.03)	0.87 (-6.94,8.69)	-0.01 (-9.62,9.60)	**DLA**	NR	NR
5.95 (-2.88,14.79)	6.81 (-7.52,21.13)	4.16 (-4.99,13.31)	3.55 (-5.53,12.63)	2.67 (-3.30,8.64)	2.68 (-7.16,12.51)	**MIS-PA**	2.17 (-4.78,9.12)
8.12 (-3.12,19.36)	8.97 (-6.95,24.90)	6.33 (-5.16,17.82)	5.71 (-5.72,17.15)	4.83 (-4.33,14.00)	4.84 (-7.20,16.89)	2.17 (-4.78,9.12)	**2-incision**

© 2023 Yan L et al. *JAMA Network Open.*

Outcome : Cup abduction angle

**MIS-PA**	-0.10 (-3.22,3.02)	NR	NR	NR	**-4.60 (-8.64,-0.56)**	-1.95 (-3.93,0.03)	-1.00 (-4.48,2.48)
-0.58 (-2.36,1.20)	**PA**	-0.10 (-2.87,2.67)	NR	NR	0.60 (-2.66,3.86)	-1.50 (-4.12,1.12)	**-3.07 (-5.75,-0.39)**
-0.99 (-3.06,1.08)	-0.41 (-2.24,1.42)	**DLA**	NR	-0.88 (-3.16,1.40)	-0.40 (-3.78,2.98)	-0.89 (-2.90,1.12)	NR
-2.16 (-8.26,3.93)	-1.58 (-7.64,4.47)	-1.18 (-7.25,4.90)	**2-incision**	NR	0.30 (-5.43,6.03)	NR	NR
-1.87 (-4.94,1.21)	-1.28 (-4.21,1.64)	-0.88 (-3.16,1.40)	0.30 (-6.19,6.79)	**MIS-DLA**	NR	NR	NR
-1.86 (-3.94,0.22)	-1.28 (-3.25,0.68)	-0.88 (-2.89,1.14)	0.30 (-5.43,6.03)	0.00 (-3.04,3.05)	**MIS-ALA**	0.40 (-2.42,3.22)	NR
**-1.87 (-3.47,-0.27)**	-1.29 (-2.91,0.33)	-0.88 (-2.49,0.73)	0.29 (-5.72,6.31)	-0.01 (-2.80,2.78)	-0.01 (-1.83,1.82)	**DAA**	NR
**-2.67 (-5.06,-0.27)**	-2.09 (-4.31,0.14)	-1.68 (-4.41,1.05)	-0.50 (-6.88,5.87)	-0.80 (-4.36,2.76)	-0.80 (-3.59,1.99)	-0.79 (-3.31,1.73)	**SuperPath**

© 2023 Yan L et al. *JAMA Network Open.*

Outcome : Cup anteversion angle

**DAA**	NR	-0.10 (-3.82,3.62)	-3.00 (-6.59,0.59)	NR	**-4.20 (-7.82,-0.58)**	NR
0.10 (-8.28,8.47)	**2-incision**	-1.90 (-9.75,5.95)	NR	NR	NR	NR
-1.80 (-4.72,1.12)	-1.90 (-9.75,5.95)	**MIS-ALA**	NR	NR	0.30 (-3.26,3.86)	NR
-2.54 (-5.54,0.47)	-2.63 (-11.38,6.11)	-0.73 (-4.58,3.12)	**MIS-PA**	-1.00 (-4.67,2.67)	NR	NR
-3.05 (-6.24,0.14)	-3.15 (-11.79,5.50)	-1.25 (-4.87,2.37)	-0.52 (-3.56,2.53)	**SuperPath**	-0.29 (-3.09,2.51)	NR
**-3.06 (-5.70,-0.42)**	-3.16 (-11.52,5.20)	-1.26 (-4.13,1.61)	-0.53 (-3.80,2.75)	-0.01 (-2.54,2.52)	**PA**	-0.90 (-4.65,2.85)
-3.96 (-8.55,0.63)	-4.06 (-13.22,5.10)	-2.16 (-6.88,2.56)	-1.43 (-6.41,3.56)	-0.91 (-5.44,3.62)	-0.90 (-4.65,2.85)	**DLA**

© 2023 Yan L et al. *JAMA Network Open.*

eTable 10F. Included studies where osteoarthritis serves as the only reason for THA

Outcome : Hip score change

**PA**	0.36 (-6.21,6.93)	NR	NR	1.84 (-4.20,7.87)	NR	6.00 (-8.23,20.23)	**10.00 (1.85,18.15)**
0.36 (-6.21,6.93)	**SuperPath**	NR	NR	NR	NR	NR	NR
1.36 (-5.17,7.88)	0.99 (-8.26,10.25)	**MIS-PA**	-0.00 (-8.08,8.08)	1.95 (-2.56,6.45)	NR	NR	NR
1.36 (-9.03,11.74)	0.99 (-11.29,13.28)	0.00 (-8.08,8.08)	**2-incision**	NR	NR	NR	NR
3.30 (-1.41,8.02)	2.94 (-5.14,11.03)	1.95 (-2.56,6.45)	1.95 (-7.30,11.20)	**DAA**	1.40 (-5.27,8.06)	1.25 (-3.29,5.80)	**4.39 (0.95,7.82)**
5.19 (-0.90,11.28)	4.83 (-4.13,13.79)	3.84 (-2.39,10.06)	3.84 (-6.36,14.04)	1.89 (-2.41,6.19)	**MIS-ALA**	2.96 (-3.91,9.84)	0.37 (-5.83,6.57)
5.38 (-0.45,11.20)	5.02 (-3.76,13.80)	4.02 (-1.98,10.03)	4.02 (-6.04,14.09)	2.07 (-1.90,6.05)	0.19 (-4.72,5.09)	**MIS-DLA**	NR
**7.53 (2.49,12.56)**	7.16 (-1.11,15.44)	**6.17 (0.75,11.60)**	6.17 (-3.56,15.90)	**4.22 (1.20,7.25)**	2.33 (-2.09,6.75)	2.15 (-2.58,6.87)	**DLA**

© 2023 Yan L et al. *JAMA Network Open.*

Outcome : Pain score change

**SuperPath**	-0.46 (-1.44,0.51)	NR	NR	NR	NR	NR
-0.46 (-1.44,0.51)	**PA**	-0.31 (-1.77,1.15)	-0.45 (-1.93,1.03)	NR	-1.00 (-2.71,0.71)	NR
-0.67 (-2.09,0.74)	-0.21 (-1.23,0.81)	**MIS-DLA**	-0.55 (-1.48,0.38)	-0.22 (-1.71,1.27)	NR	NR
-0.99 (-2.35,0.38)	-0.52 (-1.48,0.44)	-0.31 (-1.07,0.44)	**DAA**	-0.62 (-1.57,0.33)	-0.75 (-1.75,0.24)	-0.12 (-1.57,1.32)
-1.40 (-2.96,0.16)	-0.94 (-2.15,0.28)	-0.73 (-1.69,0.24)	-0.41 (-1.25,0.42)	**MIS-PA**	NR	NR
**-1.50 (-2.96,-0.04)**	-1.04 (-2.13,0.05)	-0.83 (-1.89,0.23)	-0.52 (-1.32,0.28)	-0.10 (-1.25,1.04)	**DLA**	-0.34 (-1.44,0.76)
-1.57 (-3.20,0.06)	-1.11 (-2.42,0.20)	-0.90 (-2.14,0.34)	-0.59 (-1.60,0.42)	-0.17 (-1.48,1.13)	-0.07 (-0.99,0.85)	**MIS-ALA**

© 2023 Yan L et al. *JAMA Network Open.*

Outcome : Hospitalization time

**SuperPath**	NR	NR	NR	-0.69 (-1.53,0.16)	NR	NR	NR
-0.54 (-2.11,1.04)	**2-incision**	NR	-0.09 (-0.86,0.68)	NR	NR	NR	NR
-0.60 (-1.74,0.55)	-0.06 (-1.14,1.02)	**DAA**	-0.03 (-0.78,0.73)	-0.09 (-0.86,0.68)	-1.00 (-3.46,1.46)	-0.29 (-0.84,0.27)	-0.68 (-1.46,0.10)
-0.62 (-2.00,0.75)	-0.09 (-0.86,0.68)	-0.03 (-0.78,0.73)	**MIS-PA**	NR	NR	NR	NR
-0.69 (-1.53,0.16)	-0.15 (-1.48,1.17)	-0.09 (-0.86,0.68)	-0.06 (-1.15,1.02)	**PA**	NR	NR	NR
-1.06 (-3.56,1.43)	-0.53 (-2.99,1.94)	-0.46 (-2.68,1.75)	-0.44 (-2.78,1.90)	-0.37 (-2.72,1.97)	**MIS-ALA**	NR	-1.00 (-3.74,1.74)
-0.88 (-2.16,0.39)	-0.35 (-1.56,0.87)	-0.29 (-0.84,0.27)	-0.26 (-1.20,0.68)	-0.20 (-1.15,0.76)	0.18 (-2.10,2.46)	**DLA**	NR
-1.28 (-2.67,0.11)	-0.74 (-2.07,0.59)	-0.68 (-1.46,0.10)	-0.65 (-1.74,0.43)	-0.59 (-1.69,0.51)	-0.22 (-2.46,2.02)	-0.40 (-1.35,0.56)	**MIS-DLA**

© 2023 Yan L et al. *JAMA Network Open.*

Outcome : Operation time

**PA**	NR	-6.00 (-28.26,16.26)	NR	9.50 (-12.64,31.64)	**-17.07 (-32.44,-1.71)**	**-16.28 (-32.22,-0.35)**	NR
0.68 (-18.68,20.05)	**MIS-PA**	-1.37 (-23.60,20.86)	NR	NR	-13.40 (-35.32,8.52)	NR	**-22.31 (-39.15,-5.47)**
-2.92 (-16.82,10.97)	-3.61 (-20.25,13.03)	**MIS-DLA**	-4.47 (-20.06,11.11)	NR	-9.51 (-25.91,6.89)	NR	NR
-6.32 (-21.20,8.55)	-7.01 (-25.78,11.76)	-3.40 (-15.83,9.03)	**MIS-ALA**	-2.67 (-19.44,14.10)	-4.06 (-20.50,12.38)	NR	NR
-7.11 (-20.06,5.84)	-7.80 (-26.19,10.60)	-4.19 (-17.41,9.04)	-0.79 (-12.62,11.05)	**DLA**	-0.38 (-10.97,10.20)	NR	NR
-10.54 (-22.17,1.09)	-11.22 (-27.81,5.36)	-7.61 (-19.00,3.77)	-4.21 (-15.52,7.10)	-3.43 (-12.41,5.55)	**DAA**	NR	NR
**-16.28 (-32.22,-0.35)**	-16.97 (-42.05,8.11)	-13.36 (-34.50,7.79)	-9.96 (-31.76,11.84)	-9.17 (-29.71,11.36)	-5.74 (-25.47,13.98)	**SuperPath**	NR
-21.62 (-47.29,4.04)	**-22.31 (-39.15,-5.47)**	-18.70 (-42.37,4.98)	-15.30 (-40.51,9.91)	-14.51 (-39.45,10.42)	-11.09 (-34.72,12.55)	-5.34 (-35.55,24.87)	**2-incision**

© 2023 Yan L et al. *JAMA Network Open.*

Outcome : Blood loss

**PA**	NR	NR	-1.00 (-105.21, 103.21)	-37.03 (-109.42, 35.35)	-94.90 (-235.53, 45.73)	NR	NR
-17.60 (-138.31, 103.11)	**MIS-PA**	NR	-8.10 (-90.57, 74.37)	NR	NR	-46.00 (-168.80, 76.80)	NR
-19.82 (-158.66, 119.02)	-2.22 (-152.09, 147.66)	**MIS-ALA**	60.00 (-405.83, 525.83)	NR	-14.21 (-220.70, 192.28)	NR	-85.33 (-186.33, 15.67)
-25.70 (-113.85, 62.44)	-8.10 (-90.57, 74.37)	-5.88 (-131.03, 119.26)	**MIS-DLA**	NR	-6.71 (-93.84, 80.42)	NR	NR
-37.03 (-109.42, 35.35)	-19.43 (-160.18, 121.32)	-17.21 (-173.79, 139.37)	-11.33 (-125.38, 102.73)	**SuperPath**	NR	NR	NR
-49.92 (-147.52, 47.69)	-32.31 (-145.62, 81.00)	-30.10 (-130.02, 69.83)	-24.21 (-101.92, 53.49)	-12.88 (-134.40, 108.63)	**DAA**	NR	-46.97 (-102.59, 8.64)
-63.60 (-235.80, 108.59)	-46.00 (-168.80, 76.80)	-43.78 (-237.54, 149.98)	-37.90 (-185.83, 110.03)	-26.57 (-213.36, 160.22)	-13.69 (-180.78, 153.41)	**2-incision**	NR
-98.81 (-210.11, 12.48)	-81.21 (-206.39, 43.97)	-78.99 (-169.83, 11.85)	-73.11 (-167.28, 21.06)	-61.78 (-194.54, 70.98)	-48.90 (-102.88, 5.08)	-35.21 (-210.57, 140.15)	**DLA**

© 2023 Yan L et al. *JAMA Network Open.*

Outcome : Quality of life socre change

**MIS-DLA**	NR	-0.03 (-0.59,0.53)	**2.91 (2.08,3.73)**	NR	NR	NR	NR
-5.42 (-20.07,9.23)	**MIS-ALA**	NR	NR	6.90 (-7.73,21.53)	NR	NR	NR
**0.65 (0.16,1.14)**	6.07 (-8.58,20.71)	**PA**	0.10 (-0.47,0.67)	3.00 (-5.66,11.66)	NR	2.87 (-0.44,6.18)	NR
**1.45 (0.88,2.02)**	6.87 (-7.77,21.51)	**0.80 (0.31,1.30)**	**DAA**	0.03 (-0.30,0.35)	0.92 (-1.93,3.77)	NR	NR
**1.48 (0.82,2.14)**	6.90 (-7.73,21.53)	**0.83 (0.24,1.42)**	0.03 (-0.29,0.35)	**DLA**	NR	NR	NR
2.37 (-0.54,5.28)	7.79 (-7.12,22.70)	1.72 (-1.17,4.61)	0.92 (-1.93,3.77)	0.89 (-1.98,3.76)	**MIS-PA**	NR	1.86 (-1.33,5.06)
**3.52 (0.17,6.87)**	8.94 (-6.08,23.95)	2.87 (-0.44,6.18)	2.07 (-1.28,5.42)	2.04 (-1.33,5.40)	1.15 (-3.25,5.55)	**SuperPath**	NR
4.23 (-0.09,8.55)	9.65 (-5.60,24.90)	3.59 (-0.72,7.89)	2.78 (-1.50,7.06)	2.75 (-1.54,7.05)	1.86 (-1.33,5.06)	0.72 (-4.72,6.15)	**2-incision**

© 2023 Yan L et al. *JAMA Network Open.*

Outcome : Cup abduction angle

**MIS-PA**	NR	NR	-1.96 (-4.38,0.47)	NR	NR
-0.59 (-4.63,3.44)	**MIS-ALA**	-3.22 (-7.84,1.40)	0.40 (-3.70,4.50)	NR	NR
-1.57 (-4.89,1.74)	-0.98 (-4.30,2.34)	**DLA**	-1.00 (-3.43,1.43)	NR	NR
-1.96 (-4.38,0.47)	-1.36 (-4.59,1.86)	-0.39 (-2.65,1.88)	**DAA**	-2.27 (-5.86,1.32)	NR
-4.23 (-8.56,0.10)	-3.64 (-8.47,1.19)	-2.66 (-6.90,1.58)	-2.27 (-5.86,1.32)	**PA**	-3.16 (-6.57,0.25)
**-7.39 (-12.91,-1.88)**	**-6.80 (-12.71,-0.89)**	**-5.82 (-11.26,-0.38)**	**-5.44 (-10.39,-0.49)**	-3.16 (-6.57,0.25)	**SuperPath**

© 2023 Yan L et al. *JAMA Network Open.*

Outcome : Cup anteversion angle

**SuperPath**	-0.70 (-2.04,0.63)	NR	NR	NR	NR
-0.70 (-2.04,0.63)	**PA**	NR	-2.20 (-4.95,0.55)	NR	NR
-2.49 (-7.21,2.24)	-1.78 (-6.32,2.75)	**DLA**	NR	-0.52 (-3.66,2.62)	NR
-2.91 (-5.96,0.15)	-2.20 (-4.95,0.55)	-0.42 (-4.03,3.19)	**DAA**	-0.10 (-1.87,1.67)	**-2.72 (-3.98,-1.47)**
-3.01 (-6.54,0.53)	-2.30 (-5.58,0.97)	-0.52 (-3.66,2.62)	-0.10 (-1.87,1.67)	**MIS-ALA**	NR
**-5.63 (-8.94,-2.32)**	**-4.93 (-7.95,-1.90)**	-3.14 (-6.96,0.68)	**-2.72 (-3.98,-1.47)**	**-2.62 (-4.80,-0.45)**	**MIS-PA**

© 2023 Yan L et al. *JAMA Network Open.*

eTable 10G. Included studies where both the femoral stem prosthesis and the acetabular prosthesis were non-cemented fixations

Outcome : Hip score change

**DAA**	-0.73 (-5.52,4.07)	NR	NR	1.48 (-4.45,7.41)	3.55 (-1.73,8.83)	1.00 (-5.96,7.96)	**7.85 (3.68,12.03)**
0.19 (-2.88,3.26)	**PA**	1.46 (-2.79,5.70)	NR	0.36 (-4.35,5.06)	-1.26 (-8.32,5.80)	1.81 (-3.40,7.03)	**6.88 (1.66,12.10)**
0.13 (-4.70,4.95)	-0.06 (-3.96,3.84)	**SuperPath**	NR	NR	NR	**10.20 (1.25,19.15)**	NR
1.84 (-5.05,8.73)	1.65 (-5.12,8.43)	1.71 (-5.84,9.26)	**2-incision**	5.00 (-6.60,16.60)	NR	0.00 (-7.06,7.06)	NR
2.11 (-1.15,5.37)	1.92 (-1.28,5.13)	1.98 (-2.96,6.92)	0.27 (-6.55,7.09)	**MIS-ALA**	0.71 (-3.10,4.51)	NR	2.00 (-5.49,9.49)
2.69 (-0.46,5.84)	2.50 (-0.88,5.88)	2.56 (-2.48,7.60)	0.85 (-6.16,7.85)	0.58 (-2.39,3.55)	**MIS-DLA**	8.50 (-0.25,17.25)	3.27 (-0.72,7.26)
3.59 (-0.46,7.65)	3.41 (-0.40,7.21)	3.47 (-1.47,8.40)	1.75 (-4.39,7.90)	1.48 (-2.89,5.85)	0.90 (-3.49,5.30)	**MIS-PA**	-1.30 (-9.48,6.88)
**6.43 (3.47,9.40)**	**6.25 (2.97,9.53)**	**6.31 (1.33,11.28)**	4.59 (-2.42,11.61)	**4.33 (0.98,7.67)**	**3.75 (0.71,6.78)**	2.84 (-1.48,7.16)	**DLA**

© 2023 Yan L et al. *JAMA Network Open.*

Outcome : Pain score change

**SuperPath**	-0.32 (-0.70,0.06)	NR	NR	**-1.32 (-2.45,-0.19)**	NR	NR
-0.34 (-0.71,0.02)	**PA**	-0.31 (-1.17,0.55)	-0.35 (-0.86,0.16)	NR	-1.00 (-2.24,0.24)	NR
-0.65 (-1.30,0.00)	-0.31 (-0.86,0.25)	**MIS-DLA**	0.13 (-0.52,0.78)	NR	NR	**-1.26 (-2.25,-0.27)**
**-0.78 (-1.31,-0.25)**	**-0.43 (-0.85,-0.02)**	-0.12 (-0.62,0.37)	**DAA**	-0.20 (-0.91,0.51)	-0.35 (-0.95,0.25)	-0.12 (-0.95,0.70)
**-1.07 (-1.79,-0.36)**	**-0.73 (-1.41,-0.05)**	-0.42 (-1.20,0.35)	-0.30 (-0.92,0.32)	**MIS-PA**	NR	NR
**-1.16 (-1.85,-0.47)**	**-0.82 (-1.42,-0.21)**	-0.51 (-1.18,0.16)	-0.38 (-0.89,0.12)	-0.09 (-0.88,0.70)	**DLA**	-0.20 (-1.30,0.90)
**-1.33 (-2.09,-0.57)**	**-0.98 (-1.67,-0.30)**	**-0.68 (-1.33,-0.02)**	-0.55 (-1.14,0.04)	-0.25 (-1.10,0.59)	-0.17 (-0.84,0.51)	**MIS-ALA**

© 2023 Yan L et al. *JAMA Network Open.*

Outcome : Hospitalization time

**SuperPath**	0.19 (-2.38,2.76)	NR	NR	NR	NR	**-2.22 (-3.69,-0.75)**	NR
-0.80 (-2.76,1.17)	**MIS-PA**	NR	NR	0.17 (-2.24,2.58)	0.08 (-2.31,2.47)	NR	NR
-0.84 (-3.18,1.49)	-0.05 (-2.24,2.15)	**MIS-DLA**	0.08 (-2.09,2.24)	NR	0.60 (-1.39,2.59)	NR	-1.94 (-3.91,0.02)
-0.93 (-3.52,1.65)	-0.14 (-2.45,2.18)	-0.09 (-1.98,1.81)	**MIS-ALA**	-0.70 (-3.40,2.00)	1.00 (-2.28,4.28)	NR	NR
-1.07 (-3.72,1.58)	-0.28 (-2.35,1.80)	-0.23 (-2.72,2.26)	-0.14 (-2.35,2.07)	**2-incision**	NR	NR	NR
-1.13 (-2.99,0.73)	-0.34 (-2.12,1.44)	-0.29 (-1.80,1.22)	-0.20 (-2.21,1.81)	-0.06 (-2.39,2.28)	**DAA**	-0.34 (-2.04,1.36)	-0.53 (-2.30,1.24)
**-1.90 (-3.27,-0.53)**	-1.10 (-3.09,0.89)	-1.06 (-3.18,1.06)	-0.97 (-3.41,1.47)	-0.83 (-3.42,1.77)	-0.77 (-2.31,0.77)	**PA**	NR
-2.17 (-4.51,0.18)	-1.37 (-3.62,0.88)	-1.32 (-2.88,0.24)	-1.23 (-3.47,1.00)	-1.09 (-3.73,1.54)	-1.03 (-2.51,0.44)	-0.27 (-2.38,1.85)	**DLA**

© 2023 Yan L et al. *JAMA Network Open.*

Outcome : Operation time

**PA**	2.22 (-11.84,16.28)	-13.34 (-28.33,1.64)	-1.16 (-15.00,12.69)	-8.05 (-20.35,4.24)	**-17.29 (-28.57,-6.01)**	**-23.09 (-36.36,-9.81)**	NR
-1.10 (-11.18,8.98)	**MIS-PA**	-17.50 (-40.56,5.56)	-7.30 (-27.59,12.99)	-3.20 (-27.74,21.34)	-1.50 (-21.45,18.45)	NR	**-21.00 (-41.90,-0.10)**
-7.36 (-16.03,1.30)	-6.26 (-17.96,5.44)	**MIS-DLA**	-1.26 (-13.02,10.51)	-5.85 (-16.86,5.15)	NR	-9.31 (-23.86,5.24)	NR
-8.12 (-16.59,0.35)	-7.02 (-18.09,4.05)	-0.76 (-9.29,7.77)	**MIS-ALA**	3.00 (-17.98,23.98)	NR	-3.95 (-18.56,10.66)	-13.00 (-48.19,22.19)
**-11.44 (-19.68,-3.19)**	-10.34 (-22.02,1.35)	-4.07 (-12.22,4.07)	-3.32 (-12.36,5.73)	**DLA**	NR	-3.70 (-14.31,6.90)	NR
**-13.73 (-23.85,-3.61)**	**-12.63 (-25.07,-0.19)**	-6.37 (-19.32,6.59)	-5.61 (-18.33,7.11)	-2.29 (-15.03,10.45)	**SuperPath**	NR	NR
**-16.29 (-24.75,-7.83)**	**-15.19 (-27.20,-3.18)**	**-8.93 (-17.70,-0.15)**	-8.17 (-17.24,0.90)	-4.85 (-12.72,3.02)	-2.56 (-15.48,10.36)	**DAA**	NR
**-21.84 (-41.67,-2.02)**	**-20.75 (-38.95,-2.54)**	-14.48 (-34.96,6.00)	-13.72 (-33.47,6.02)	-10.41 (-30.94,10.12)	-8.11 (-29.47,13.24)	-5.55 (-26.23,15.12)	**2-incision**

© 2023 Yan L et al. *JAMA Network Open.*

Outcome : Blood loss

**MIS-ALA**	32.36 (-148.57, 213.30)	-17.06 (-256.33, 222.20)	NR	NR	-135.00 (-395.79, 125.79)	**-228.82 (-413.25, -44.39)**	NR
-15.46 (-144.43, 113.51)	**MIS-DLA**	-16.27 (-181.94, 149.39)	NR	NR	NR	1.00 (-183.17, 185.17)	**-330.67 (-523.71, -137.64)**
-87.69 (-231.19, 55.82)	-72.23 (-187.61, 43.15)	**DAA**	NR	NR	NR	NR	-61.80 (-155.93, 32.34)
-92.74 (-265.57, 80.09)	-77.28 (-256.70, 102.14)	-5.05 (-195.57, 185.46)	**MIS-PA**	26.66 (-147.08, 200.40)	-46.00 (-241.30, 149.30)	-121.70 (-348.34, 104.94)	NR
-110.47 (-265.98, 45.05)	-95.01 (-250.71, 60.69)	-22.78 (-191.22, 145.66)	-17.73 (-153.46, 118.01)	**SuperPath**	NR	-14.26 (-118.57, 90.06)	NR
-137.40 (-328.97, 54.17)	-121.94 (-332.08, 88.21)	-49.71 (-269.29, 169.87)	-44.66 (-212.86, 123.55)	-26.93 (-222.00, 168.15)	**2-incision**	NR	NR
**-140.72 (-268.66, -12.79)**	**-125.26 (-250.20, -0.32)**	-53.04 (-193.55, 87.48)	-47.98 (-186.89, 90.93)	-30.26 (-126.97, 66.46)	-3.33 (-192.55, 185.89)	**PA**	8.00 (-190.89, 206.89)
**-178.91 (-325.27, -32.54)**	**-163.45 (-280.15, -46.74)**	**-91.22 (-176.73, -5.71)**	-86.17 (-273.63, 101.30)	-68.44 (-232.47, 95.59)	-41.51 (-260.08, 177.06)	-38.18 (-172.97, 96.60)	**DLA**

© 2023 Yan L et al. *JAMA Network Open.*

Outcome : Quality of life socre change

**MIS-ALA**	0.54 (-2.09,3.17)	NR	NR	6.90 (-7.73,21.53)	NR	NR	NR
0.73 (-1.85,3.32)	**PA**	0.03 (-0.31,0.37)	0.10 (-0.25,0.45)	3.00 (-5.65,11.65)	2.87 (-0.41,6.15)	NR	NR
0.76 (-1.85,3.36)	0.02 (-0.32,0.36)	**MIS-DLA**	3.26 (-3.58,10.10)	NR	NR	NR	NR
0.87 (-1.74,3.48)	0.14 (-0.21,0.48)	0.11 (-0.37,0.60)	**DAA**	0.01 (-0.23,0.25)	NR	NR	0.00 (-3.13,3.13)
0.88 (-1.73,3.50)	0.15 (-0.28,0.57)	0.13 (-0.42,0.67)	0.01 (-0.23,0.26)	**DLA**	NR	NR	NR
1.91 (-2.18,5.99)	1.17 (-1.99,4.33)	1.15 (-2.03,4.33)	1.04 (-2.14,4.21)	1.02 (-2.16,4.21)	**SuperPath**	NR	**20.25 (9.05,31.45)**
2.41 (-3.35,8.17)	1.68 (-3.47,6.82)	1.65 (-3.50,6.81)	1.54 (-3.59,6.67)	1.53 (-3.61,6.67)	0.50 (-5.41,6.42)	**2-incision**	0.00 (-4.15,4.15)
2.41 (-1.58,6.40)	1.68 (-1.36,4.72)	1.65 (-1.40,4.71)	1.54 (-1.48,4.56)	1.53 (-1.50,4.56)	0.50 (-3.71,4.72)	-0.00 (-4.15,4.15)	**MIS-PA**

© 2023 Yan L et al. *JAMA Network Open.*

Outcome : Cup abduction angle

**MIS-PA**	NR	-2.40 (-6.02,1.22)	NR	NR	**-4.60 (-8.44,-0.76)**	-2.00 (-4.95,0.95)	-1.00 (-4.24,2.24)
-0.72 (-3.02,1.58)	**DLA**	0.10 (-2.36,2.56)	-0.91 (-2.69,0.88)	NR	NR	**-2.66 (-4.97,-0.35)**	NR
-1.30 (-3.32,0.72)	-0.58 (-2.39,1.24)	**PA**	NR	NR	0.60 (-2.41,3.61)	-0.30 (-4.12,3.52)	**-3.04 (-5.56,-0.52)**
-1.86 (-4.36,0.63)	-1.14 (-2.72,0.44)	-0.56 (-2.70,1.57)	**MIS-DLA**	NR	-0.00 (-2.29,2.29)	NR	NR
-2.55 (-8.54,3.45)	-1.83 (-7.72,4.07)	-1.25 (-7.15,4.65)	-0.69 (-6.57,5.20)	**2-incision**	0.30 (-5.29,5.89)	NR	NR
**-2.25 (-4.41,-0.09)**	-1.53 (-3.40,0.34)	-0.95 (-2.83,0.93)	-0.39 (-2.21,1.44)	0.30 (-5.29,5.89)	**MIS-ALA**	0.40 (-2.13,2.93)	NR
**-2.39 (-4.45,-0.34)**	-1.67 (-3.41,0.07)	-1.10 (-2.97,0.78)	-0.53 (-2.59,1.53)	0.16 (-5.71,6.02)	-0.14 (-1.91,1.63)	**DAA**	NR
**-3.08 (-5.43,-0.73)**	-2.36 (-5.02,0.30)	-1.78 (-3.91,0.35)	-1.22 (-4.08,1.64)	-0.53 (-6.71,5.65)	-0.83 (-3.47,1.81)	-0.69 (-3.29,1.92)	**SuperPath**

© 2023 Yan L et al. *JAMA Network Open.*

Outcome : Cup anteversion angle

**PA**	NR	NR	-0.90 (-5.41,3.61)	-0.30 (-4.65,4.05)	0.23 (-3.09,3.56)	-4.30 (-10.44,1.84)	-4.20 (-9.30,0.90)
1.02 (-7.96,9.99)	**2-incision**	NR	NR	-1.90 (-10.14,6.34)	NR	NR	NR
0.12 (-6.09,6.32)	-0.90 (-10.58,8.78)	**MIS-DLA**	NR	-1.00 (-6.08,4.08)	NR	NR	NR
-0.90 (-5.41,3.61)	-1.92 (-11.96,8.13)	-1.02 (-8.68,6.65)	**DLA**	NR	NR	NR	NR
-0.88 (-4.45,2.68)	-1.90 (-10.14,6.34)	-1.00 (-6.08,4.08)	0.02 (-5.73,5.76)	**MIS-ALA**	NR	0.10 (-4.38,4.58)	NR
-1.06 (-3.96,1.85)	-2.08 (-11.42,7.27)	-1.18 (-7.90,5.55)	-0.16 (-5.52,5.20)	-0.18 (-4.59,4.24)	**SuperPath**	NR	1.00 (-3.44,5.44)
-1.40 (-4.95,2.15)	-2.42 (-11.42,6.58)	-1.52 (-7.75,4.71)	-0.50 (-6.24,5.23)	-0.52 (-4.14,3.10)	-0.34 (-4.54,3.85)	**DAA**	-2.00 (-6.76,2.76)
-2.36 (-5.61,0.89)	-3.38 (-12.67,5.92)	-2.48 (-9.14,4.18)	-1.46 (-7.01,4.09)	-1.48 (-5.78,2.83)	-1.30 (-4.68,2.08)	-0.96 (-4.63,2.71)	**MIS-PA**

© 2023 Yan L et al. *JAMA Network Open.*

eTable 10H. Inclusion of studies that state all procedures were unilateral

Outcome:Hip score change

**PA**	1.54 (-2.18,5.26)	-1.88 (-6.69,2.94)	NR	-1.48 (-8.04,5.08)	0.85 (-2.06,3.76)	1.57 (-3.17,6.31)	**6.93 (2.27,11.60)**
-0.18 (-3.59,3.24)	**SuperPath**	NR	NR	NR	**10.20 (1.89,18.51)**	NR	NR
1.02 (-2.13,4.18)	1.20 (-3.38,5.78)	**MIS-ALA**	NR	0.83 (-2.80,4.47)	-2.00 (-10.64,6.64)	NR	0.28 (-5.05,5.61)
1.44 (-5.24,8.12)	1.62 (-5.75,8.99)	0.42 (-6.69,7.53)	**2-incision**	NR	0.00 (-6.23,6.23)	NR	NR
1.44 (-1.50,4.38)	1.62 (-2.79,6.03)	0.42 (-2.39,3.23)	0.00 (-6.95,6.95)	**MIS-DLA**	**8.50 (0.41,16.59)**	-0.30 (-3.60,3.00)	**4.73 (0.70,8.75)**
1.44 (-0.94,3.82)	1.62 (-2.31,5.55)	0.42 (-3.00,3.83)	-0.00 (-6.23,6.23)	-0.00 (-3.08,3.07)	**MIS-PA**	2.04 (-1.16,5.23)	-1.30 (-8.78,6.18)
2.20 (-0.34,4.74)	2.38 (-1.76,6.51)	1.18 (-1.95,4.30)	0.76 (-5.94,7.46)	0.76 (-1.70,3.22)	0.76 (-1.70,3.22)	**DAA**	2.61 (-0.57,5.79)
**4.80 (1.98,7.63)**	**4.98 (0.64,9.32)**	**3.78 (0.69,6.87)**	3.36 (-3.55,10.28)	**3.36 (0.71,6.01)**	**3.36 (0.37,6.35)**	**2.60 (0.22,4.98)**	**DLA**

© 2023 Yan L et al. *JAMA Network Open.*

Outcome : Pain score change

**SuperPath**	NR	-0.35 (-1.12,0.42)	NR	-1.32 (-2.94,0.30)	NR	NR
-0.48 (-1.53,0.58)	**MIS-DLA**	0.31 (-1.13,1.75)	-0.55 (-1.47,0.37)	-0.22 (-1.69,1.25)	-1.26 (-2.78,0.26)	NR
-0.44 (-1.15,0.27)	0.04 (-0.83,0.90)	**PA**	-0.45 (-1.91,1.01)	0.10 (-1.35,1.55)	NR	-1.00 (-2.70,0.70)
-0.93 (-1.91,0.06)	-0.45 (-1.14,0.24)	-0.48 (-1.27,0.30)	**DAA**	-0.14 (-0.95,0.68)	NR	-0.43 (-1.23,0.37)
-0.92 (-1.87,0.04)	-0.44 (-1.25,0.37)	-0.48 (-1.28,0.33)	0.01 (-0.66,0.68)	**MIS-PA**	NR	NR
**-1.48 (-2.76,-0.19)**	-1.00 (-2.01,0.01)	-1.04 (-2.16,0.09)	-0.55 (-1.51,0.41)	-0.56 (-1.67,0.55)	**MIS-ALA**	-0.04 (-0.93,0.84)
**-1.43 (-2.55,-0.32)**	**-0.96 (-1.83,-0.08)**	**-0.99 (-1.92,-0.07)**	-0.51 (-1.20,0.19)	-0.52 (-1.42,0.39)	0.04 (-0.76,0.84)	**DLA**

© 2023 Yan L et al. *JAMA Network Open.*

Outcome : Hospitalization time

**SuperPath**	-2.34 (-4.98,0.30)	NR	NR	NR	NR	NR	0.19 (-4.39,4.77)
-1.18 (-3.52,1.17)	**PA**	0.09 (-3.10,3.27)	NR	NR	NR	NR	**-4.32 (-7.00,-1.64)**
-2.47 (-5.61,0.66)	-1.30 (-3.66,1.07)	**DAA**	-0.65 (-3.87,2.56)	NR	NR	-0.59 (-3.81,2.63)	0.08 (-2.53,2.70)
-2.88 (-7.02,1.26)	-1.71 (-5.30,1.89)	-0.41 (-3.11,2.29)	**MIS-DLA**	NR	-0.50 (-5.19,4.19)	-1.00 (-5.66,3.66)	NR
-3.15 (-8.43,2.13)	-1.97 (-6.92,2.97)	-0.68 (-5.68,4.32)	-0.27 (-5.95,5.41)	**2-incision**	NR	NR	-0.17 (-4.67,4.33)
-3.44 (-8.52,1.63)	-2.27 (-6.91,2.37)	-0.97 (-4.96,3.01)	-0.56 (-4.16,3.04)	-0.30 (-6.69,6.10)	**MIS-ALA**	0.20 (-4.37,4.77)	NR
-3.31 (-7.45,0.84)	-2.13 (-5.73,1.46)	-0.84 (-3.54,1.87)	-0.43 (-3.34,2.49)	-0.16 (-5.84,5.53)	0.14 (-3.43,3.71)	**DLA**	NR
**-3.32 (-6.08,-0.56)**	**-2.14 (-4.21,-0.08)**	-0.85 (-3.04,1.34)	-0.44 (-3.91,3.04)	-0.17 (-4.67,4.33)	0.13 (-4.42,4.67)	-0.01 (-3.49,3.46)	**MIS-PA**

© 2023 Yan L et al. *JAMA Network Open.*

Outcome : Operation time

**PA**	0.86 (-15.55,17.27)	-3.88 (-23.59,15.83)	-13.39 (-28.61,1.84)	-8.09 (-20.58,4.41)	**-17.30 (-28.79,-5.81)**	**-17.09 (-30.76,-3.43)**	NR
-2.34 (-11.69,7.01)	**MIS-PA**	-7.30 (-27.93,13.33)	-8.14 (-23.28,7.00)	-3.20 (-28.02,21.62)	-1.50 (-21.80,18.80)	-13.78 (-27.99,0.43)	-21.00 (-42.24,0.24)
-7.22 (-17.59,3.14)	-4.88 (-15.89,6.13)	**MIS-ALA**	-2.69 (-17.72,12.35)	-2.03 (-17.23,13.17)	NR	NR	NR
-7.58 (-16.84,1.68)	-5.24 (-15.12,4.65)	-0.36 (-10.34,9.62)	**MIS-DLA**	-8.26 (-21.20,4.67)	NR	-1.54 (-21.43,18.35)	NR
**-12.30 (-20.72,-3.88)**	**-9.96 (-19.83,-0.09)**	-5.08 (-14.95,4.79)	-4.72 (-13.52,4.07)	**DLA**	NR	-1.77 (-11.99,8.46)	NR
**-14.04 (-24.29,-3.78)**	-11.70 (-23.95,0.56)	-6.81 (-20.77,7.14)	-6.46 (-19.59,6.68)	-1.73 (-14.43,10.96)	**SuperPath**	NR	NR
**-14.69 (-23.31,-6.06)**	**-12.35 (-21.76,-2.93)**	-7.46 (-18.44,3.51)	-7.11 (-16.61,2.39)	-2.38 (-10.28,5.51)	-0.65 (-13.37,12.07)	**DAA**	NR
**-23.34 (-46.54,-0.14)**	-21.00 (-42.24,0.24)	-16.12 (-40.04,7.80)	-15.76 (-39.19,7.66)	-11.04 (-34.46,12.38)	-9.30 (-33.82,15.21)	-8.65 (-31.89,14.58)	**2-incision**

© 2023 Yan L et al. *JAMA Network Open.*

Outcome : Blood loss

**MIS-ALA**	28.00 (-151.43, 207.43)	-79.00 (-307.19, 149.19)	NR	NR	NR	-111.07 (-238.60, 16.46)	-85.33 (-251.31, 80.65)
-7.72 (-113.90, 98.45)	**MIS-DLA**	8.10 (-147.30, 163.50)	NR	NR	-2.50 (-161.28, 156.28)	1.00 (-166.95, 168.95)	**-326.86 (-511.95, -141.77)**
-31.04 (-142.63, 80.55)	-23.32 (-123.10, 76.46)	**MIS-PA**	26.66 (-129.78, 183.10)	-46.00 (-226.08, 134.08)	NR	-39.93 (-127.61, 47.74)	NR
-42.83 (-168.22, 82.55)	-35.11 (-154.63, 84.40)	-11.79 (-108.73, 85.15)	**SuperPath**	NR	NR	-17.08 (-111.65, 77.49)	NR
-77.04 (-288.89, 134.81)	-69.32 (-275.20, 136.55)	-46.00 (-226.08, 134.08)	-34.21 (-238.73, 170.31)	**2-incision**	NR	NR	NR
-79.40 (-198.07, 39.27)	-71.68 (-173.49, 30.13)	-48.36 (-167.15, 70.44)	-36.57 (-168.01, 94.88)	-2.36 (-218.09, 213.38)	**DAA**	94.90 (-97.78, 287.58)	-70.25 (-160.09, 19.60)
-73.96 (-170.88, 22.95)	-66.24 (-157.58, 25.09)	-42.92 (-115.79, 29.94)	-31.13 (-114.38, 52.12)	3.08 (-191.19, 197.34)	5.44 (-99.02, 109.90)	**PA**	8.00 (-175.97, 191.97)
**-152.35 (-260.80, -43.90)**	**-144.63 (-244.03, -45.22)**	**-121.31 (-235.83, -6.78)**	-109.51 (-236.97, 17.94)	-75.31 (-288.72, 138.11)	-72.95 (-151.76, 5.86)	-78.38 (-177.71, 20.94)	**DLA**

© 2023 Yan L et al. *JAMA Network Open.*

Outcome : Quality of life socre change

**MIS-DLA**	NR	-0.03 (-0.63,0.57)	NR	NR	NR	**2.91 (2.05,3.76)**	NR
-0.12 (-2.31,2.06)	**MIS-ALA**	1.07 (-1.52,3.66)	NR	5.00 (-4.73,14.73)	NR	NR	1.24 (-2.55,5.03)
**0.69 (0.17,1.21)**	0.81 (-1.32,2.95)	**PA**	NR	-0.94 (-3.20,1.32)	2.87 (-0.45,6.19)	0.10 (-0.51,0.71)	3.00 (-5.66,11.66)
1.40 (-3.15,5.96)	1.53 (-3.45,6.51)	0.72 (-3.82,5.25)	**2-incision**	0.00 (-4.18,4.18)	NR	NR	NR
1.40 (-0.41,3.21)	1.53 (-1.19,4.24)	0.72 (-1.04,2.47)	0.00 (-4.18,4.18)	**MIS-PA**	**-20.25 (-31.46,-9.04)**	-0.92 (-3.78,1.93)	NR
1.75 (-1.48,4.98)	1.87 (-1.96,5.71)	1.06 (-2.13,4.25)	0.35 (-5.15,5.84)	0.35 (-3.22,3.91)	**SuperPath**	NR	NR
**1.48 (0.87,2.08)**	1.60 (-0.56,3.76)	**0.79 (0.27,1.31)**	0.07 (-4.47,4.61)	0.07 (-1.70,1.84)	-0.27 (-3.50,2.95)	**DAA**	0.03 (-0.32,0.38)
**1.51 (0.81,2.20)**	1.63 (-0.53,3.80)	**0.82 (0.20,1.44)**	0.10 (-4.45,4.66)	0.10 (-1.70,1.91)	-0.24 (-3.49,3.00)	0.03 (-0.32,0.38)	**DLA**

© 2023 Yan L et al. *JAMA Network Open.*

Outcome : Cup abduction angle

**MIS-PA**	-0.92 (-4.55,2.70)	NR	0.35 (-2.18,2.87)	NR	-1.10 (-3.06,0.86)	-1.00 (-5.12,3.12)
-0.13 (-2.37,2.11)	**MIS-ALA**	-1.35 (-4.26,1.55)	-1.08 (-4.28,2.12)	0.04 (-2.92,2.99)	NR	NR
-0.25 (-2.33,1.83)	-0.12 (-2.05,1.81)	**DLA**	0.10 (-3.44,3.64)	-0.89 (-3.64,1.87)	-1.03 (-3.17,1.11)	NR
-0.63 (-2.40,1.15)	-0.50 (-2.65,1.65)	-0.38 (-2.38,1.63)	**PA**	NR	2.26 (-1.04,5.56)	**-3.13 (-6.24,-0.03)**
-0.65 (-3.44,2.14)	-0.52 (-2.78,1.74)	-0.40 (-2.61,1.80)	-0.02 (-2.75,2.70)	**MIS-DLA**	NR	NR
-0.73 (-2.37,0.90)	-0.60 (-2.83,1.63)	-0.48 (-2.25,1.29)	-0.11 (-1.98,1.77)	-0.08 (-2.75,2.59)	**DAA**	NR
**-2.76 (-5.49,-0.03)**	-2.63 (-5.82,0.56)	-2.51 (-5.60,0.58)	-2.13 (-4.70,0.43)	-2.11 (-5.71,1.49)	-2.03 (-4.97,0.91)	**SuperPath**

© 2023 Yan L et al. *JAMA Network Open.*

Outcome : Cup anteversion angle

**PA**	NR	-2.40 (-6.18,1.38)	-0.90 (-4.93,3.13)	0.27 (-2.73,3.26)	-0.30 (-4.15,3.55)	-0.50 (-4.97,3.97)
0.39 (-5.31,6.09)	**MIS-DLA**	NR	NR	NR	-1.00 (-5.66,3.66)	NR
-0.56 (-3.39,2.26)	-0.96 (-7.32,5.41)	**DAA**	NR	NR	NR	-1.48 (-4.15,1.19)
-0.56 (-3.93,2.81)	-0.95 (-6.86,4.95)	0.00 (-4.40,4.40)	**DLA**	NR	-0.52 (-5.29,4.25)	NR
-0.60 (-3.19,1.98)	-1.00 (-7.25,5.26)	-0.04 (-3.38,3.30)	-0.04 (-4.29,4.20)	**SuperPath**	NR	1.00 (-2.95,4.95)
-0.61 (-3.89,2.67)	-1.00 (-5.66,3.66)	-0.04 (-4.37,4.29)	-0.05 (-3.67,3.58)	-0.00 (-4.18,4.17)	**MIS-ALA**	NR
-1.13 (-3.82,1.57)	-1.52 (-7.82,4.79)	-0.56 (-2.92,1.80)	-0.56 (-4.88,3.75)	-0.52 (-3.46,2.42)	-0.52 (-4.76,3.73)	**MIS-PA**

© 2023 Yan L et al. *JAMA Network Open.*

eTable 10I. Exclusion of studies without standard deviation

Outcome : Hip score change

**2-incision**	NR	0.00 (-6.78,6.78)	NR	NR	5.00 (-6.43,16.43)	NR	NR
0.44 (-6.63,7.51)	**SuperPath**	**10.20 (1.47,18.93)**	-1.48 (-5.55,2.59)	NR	NR	NR	NR
0.99 (-4.90,6.87)	0.55 (-3.55,4.65)	**MIS-PA**	0.46 (-2.20,3.13)	1.99 (-1.52,5.51)	2.00 (-7.04,11.04)	-8.50 (-17.02,0.02)	-1.30 (-9.25,6.65)
1.06 (-5.07,7.19)	0.62 (-3.09,4.33)	0.07 (-2.11,2.24)	**PA**	-0.21 (-3.09,2.67)	-0.15 (-4.13,3.83)	-1.33 (-8.22,5.56)	**6.89 (1.86,11.93)**
1.44 (-4.74,7.62)	1.01 (-3.16,5.17)	0.46 (-1.91,2.83)	0.39 (-1.66,2.43)	**DAA**	1.51 (-4.22,7.23)	0.96 (-2.64,4.56)	**4.33 (1.45,7.21)**
2.20 (-4.05,8.44)	1.76 (-2.74,6.26)	1.21 (-1.81,4.23)	1.14 (-1.49,3.77)	0.75 (-1.83,3.34)	**MIS-ALA**	2.12 (-1.96,6.20)	0.94 (-3.52,5.39)
2.32 (-4.08,8.72)	1.89 (-2.65,6.43)	1.33 (-1.70,4.37)	1.27 (-1.45,3.98)	0.88 (-1.49,3.25)	0.12 (-2.57,2.82)	**MIS-DLA**	**3.70 (0.61,6.80)**
5.50 (-0.84,11.83)	**5.06 (0.64,9.48)**	**4.51 (1.65,7.36)**	**4.44 (1.93,6.94)**	**4.05 (1.93,6.17)**	**3.30 (0.67,5.93)**	**3.17 (0.84,5.51)**	**DLA**

© 2023 Yan L et al. *JAMA Network Open.*

Outcome : Pain score change

**SuperPath**	NR	-0.35 (-1.10,0.40)	NR	-1.32 (-2.91,0.27)	NR	NR
-0.19 (-1.23,0.84)	**MIS-DLA**	0.31 (-1.09,1.71)	-0.55 (-1.44,0.33)	NR	-1.26 (-2.75,0.23)	NR
-0.48 (-1.17,0.20)	-0.29 (-1.09,0.51)	**PA**	0.03 (-0.72,0.78)	0.02 (-0.82,0.85)	NR	-1.00 (-2.66,0.66)
-0.64 (-1.49,0.21)	-0.44 (-1.14,0.26)	-0.15 (-0.70,0.40)	**DAA**	-0.15 (-0.94,0.64)	-0.12 (-1.51,1.26)	-0.43 (-1.21,0.34)
-0.71 (-1.55,0.13)	-0.52 (-1.41,0.37)	-0.23 (-0.84,0.38)	-0.08 (-0.70,0.54)	**MIS-PA**	NR	NR
-1.09 (-2.20,0.02)	-0.90 (-1.81,0.01)	-0.61 (-1.51,0.30)	-0.45 (-1.23,0.32)	-0.38 (-1.34,0.59)	**MIS-ALA**	-0.04 (-0.91,0.83)
-1.14 (-2.15,-0.12)	**-0.95 (-1.82,-0.07)**	-0.66 (-1.43,0.12)	-0.50 (-1.14,0.13)	-0.43 (-1.28,0.43)	-0.05 (-0.77,0.67)	**DLA**

© 2023 Yan L et al. *JAMA Network Open.*

Outcome : Hospitalization time

**PA**	NR	0.47 (-0.32,1.26)	NR	NR	NR	**-2.90 (-3.95,-1.84)**
-0.04 (-1.34,1.25)	**MIS-DLA**	0.50 (-1.17,2.16)	0.03 (-1.86,1.93)	-0.91 (-2.23,0.41)	NR	NR
-0.09 (-0.78,0.61)	-0.04 (-1.16,1.07)	**DAA**	-1.00 (-3.95,1.95)	-0.34 (-1.34,0.66)	NR	0.07 (-1.09,1.24)
-0.53 (-2.01,0.95)	-0.49 (-1.85,0.88)	-0.44 (-1.80,0.91)	**MIS-ALA**	0.20 (-1.92,2.32)	-0.70 (-2.99,1.59)	NR
-0.58 (-1.68,0.52)	-0.54 (-1.57,0.49)	-0.49 (-1.36,0.37)	-0.05 (-1.38,1.27)	**DLA**	NR	NR
-1.17 (-2.59,0.25)	-1.13 (-2.80,0.54)	-1.09 (-2.49,0.31)	-0.64 (-2.24,0.96)	-0.59 (-2.15,0.97)	**2-incision**	-0.09 (-1.47,1.30)
**-1.24 (-2.03,-0.45)**	-1.19 (-2.54,0.15)	**-1.15 (-1.97,-0.33)**	-0.71 (-2.18,0.76)	-0.66 (-1.82,0.51)	-0.07 (-1.31,1.18)	**MIS-PA**

© 2023 Yan L et al. *JAMA Network Open.*

Outcome : Operation time

**PA**	-2.25 (-14.15,9.65)	0.25 (-6.85,7.36)	-1.19 (-12.23,9.85)	-3.14 (-13.15,6.87)	**-17.10 (-26.08,-8.12)**	**-18.90 (-25.48,-12.31)**	NR
-2.22 (-8.37,3.93)	**MIS-DLA**	-6.04 (-18.16,6.08)	-1.62 (-11.09,7.85)	**-11.23 (-19.78,-2.68)**	NR	-8.74 (-20.52,3.04)	NR
-2.10 (-7.47,3.27)	0.12 (-6.73,6.96)	**MIS-PA**	-7.30 (-23.80,9.20)	**23.80 (2.39,45.21)**	-1.50 (-17.58,14.58)	**-13.76 (-24.93,-2.59)**	**-22.28 (-34.81,-9.75)**
**-6.71 (-12.81,-0.62)**	-4.50 (-10.85,1.86)	-4.61 (-11.52,2.30)	**MIS-ALA**	-0.46 (-10.41,9.49)	NR	-3.71 (-15.68,8.25)	-13.00 (-46.15,20.15)
**-10.91 (-16.51,-5.31)**	**-8.69 (-14.63,-2.76)**	**-8.81 (-15.51,-2.11)**	-4.20 (-10.29,1.90)	**DLA**	NR	0.03 (-6.77,6.83)	NR
**-13.89 (-21.83,-5.95)**	**-11.68 (-21.48,-1.87)**	**-11.79 (-20.64,-2.95)**	-7.18 (-16.97,2.61)	-2.98 (-12.51,6.55)	**SuperPath**	NR	NR
**-14.01 (-18.85,-9.16)**	**-11.79 (-17.90,-5.68)**	**-11.91 (-17.95,-5.87)**	**-7.29 (-13.45,-1.14)**	-3.10 (-8.19,1.99)	-0.11 (-9.21,8.98)	**DAA**	NR
**-23.79 (-36.52,-11.07)**	**-21.57 (-34.92,-8.23)**	**-21.69 (-33.45,-9.94)**	**-17.08 (-30.27,-3.89)**	-12.88 (-26.15,0.39)	-9.90 (-24.48,4.68)	-9.79 (-22.78,3.21)	**2-incision**

© 2023 Yan L et al. *JAMA Network Open*.

Out come 5: Blood loss

**MIS-ALA**	30.84 (-110.27 ,171.95)	-79.00 (-283.29 ,125.29)	NR	-120.38 (-224.91 ,-15.85)	-135.00 (-362.95 ,92.95)	-65.00 (-514.46 ,384.46)	-85.33 (-216.51 ,45.85)
-10.58 (-90.79 ,69.63)	**MIS-DLA**	8.10 (-109.42 ,125.62)	NR	1.00 (-132.68 ,134.68)	NR	-10.00 (-128.15 ,108.15)	**-237.06 (-336.14 ,-137.98)**
-25.89 (-107.37 ,55.60)	-15.31 (-85.76 ,55.14)	**MIS-PA**	26.66 (-92.23 ,145.55)	-52.05 (-107.85 ,3.76)	-46.00 (-194.63 ,102.63)	NR	NR
-35.11 (-130.94 ,60.72)	-24.53 (-112.76 ,63.70)	-9.22 (-80.72 ,62.28)	**SuperPath**	-26.47 (-99.87 ,46.93)	NR	NR	NR
**-75.26 (-148.76 ,-1.76)**	**-64.68 (-128.79 ,-0.57)**	**-49.37 (-97.43 ,-1.31)**	-40.15 (-104.00 ,23.70)	**PA**	NR	-45.61 (-117.89 ,26.67)	8.00 (-145.31 ,161.31)
-90.71 (-227.72 ,46.29)	-80.14 (-219.82 ,59.55)	-64.83 (-191.68 ,62.02)	-55.61 (-198.56 ,87.35)	-15.45 (-147.15 ,116.24)	**2-incision**	NR	NR
**-93.67 (-174.89 ,-12.44)**	**-83.09 (-148.78 ,-17.40)**	-67.78 (-138.31 ,2.75)	-58.56 (-143.23 ,26.12)	-18.41 (-75.47 ,38.66)	-2.95 (-142.84 ,136.94)	**DAA**	-67.76 (-130.34 ,-5.18)

© 2023 Yan L et al. *JAMA Network Open.*

**-162.20 (-241.54 ,-82.86)**	**-151.62 (-217.10 ,-86.14)**	**-136.31 (-211.27 ,-61.36)**	**-127.09 (-216.30 ,-37.89)**	**-86.94 (-150.96 ,-22.92)**	-71.49 (-212.66 ,69.69)	**-68.53 (-121.28 ,-15.78)**	**DLA**

Outcome : Cup abduction angle

**MIS-PA**	-0.75 (-4.72,3.21)	NR	0.60 (-1.50,2.70)	NR	NR	-1.00 (-3.27,1.27)	-1.00 (-5.69,3.69)
-0.04 (-2.31,2.23)	**MIS-ALA**	-1.37 (-4.67,1.94)	-1.12 (-4.71,2.48)	-0.30 (-6.84,6.24)	0.05 (-3.31,3.41)	0.40 (-3.83,4.63)	NR
-0.05 (-2.35,2.25)	-0.01 (-2.09,2.07)	**DLA**	0.10 (-4.09,4.29)	NR	-0.89 (-4.07,2.29)	-1.00 (-3.50,1.50)	NR
-0.08 (-1.72,1.56)	-0.04 (-2.24,2.15)	-0.03 (-2.22,2.15)	**PA**	NR	NR	0.60 (-2.13,3.33)	-3.17 (-6.65,0.32)
-0.34 (-7.26,6.58)	-0.30 (-6.84,6.24)	-0.29 (-7.15,6.57)	-0.26 (-7.15,6.64)	**2-incision**	NR	NR	NR
-0.49 (-3.57,2.59)	-0.45 (-3.01,2.11)	-0.44 (-2.95,2.07)	-0.41 (-3.42,2.60)	-0.15 (-7.17,6.87)	**MIS-DLA**	NR	NR
-0.50 (-2.23,1.23)	-0.46 (-2.59,1.67)	-0.45 (-2.39,1.49)	-0.42 (-2.18,1.35)	-0.16 (-7.04,6.72)	-0.01 (-2.91,2.89)	**DAA**	NR
-2.45 (-5.44,0.54)	-2.41 (-5.89,1.08)	-2.40 (-5.89,1.09)	-2.37 (-5.22,0.49)	-2.11 (-9.52,5.30)	-1.96 (-6.01,2.09)	-1.95 (-5.15,1.26)	**SuperPath**

© 2023 Yan L et al. *JAMA Network Open.*

eTable 10J. Included studies where all surgerns were experienced.

(Performed over forty surgeries)

Outcome: Hip score change

**DAA**	NR	2.10 (-3.12,7.32)	1.32 (-1.43,4.08)	NR	0.40 (-4.34,5.13)	NR	**3.92 (0.00,7.84)**
0.26 (-3.48,4.00)	**MIS-DLA**	-1.96 (-9.11,5.20)	3.90 (-3.02,10.82)	NR	**8.50 (0.99,16.01)**	NR	3.17 (-0.07,6.40)
0.33 (-3.19,3.86)	0.07 (-4.27,4.41)	**MIS-ALA**	1.81 (-3.37,6.99)	NR	NR	NR	NR
1.47 (-0.74,3.68)	1.21 (-2.59,5.01)	1.14 (-2.40,4.67)	**PA**	NR	1.18 (-1.57,3.92)	2.22 (-5.69,10.13)	3.30 (-2.90,9.50)
2.24 (-3.90,8.39)	1.98 (-4.87,8.84)	1.91 (-4.90,8.72)	0.77 (-5.20,6.75)	**2-incision**	0.00 (-5.46,5.46)	NR	NR
2.24 (-0.57,5.06)	1.98 (-2.16,6.13)	1.91 (-2.17,5.99)	0.77 (-1.67,3.21)	0.00 (-5.46,5.46)	**MIS-PA**	NR	-1.30 (-8.15,5.55)
3.69 (-4.52,11.90)	3.43 (-5.34,12.20)	3.36 (-5.30,12.02)	2.22 (-5.69,10.13)	1.45 (-8.47,11.36)	1.45 (-6.83,9.72)	**SuperPath**	NR
**3.35 (0.28,6.42)**	**3.09 (0.06,6.12)**	3.02 (-1.17,7.20)	1.88 (-1.42,5.18)	1.11 (-5.48,7.69)	1.11 (-2.58,4.79)	-0.34 (-8.91,8.23)	**DLA**

© 2023 Yan L et al. *JAMA Network Open.*

Outcome: Pain score change

**MIS-PA**	NR	-0.10 (-1.57,1.37)	-0.44 (-1.51,0.63)	NR	NR
-0.01 (-1.67,1.65)	**SuperPath**	-0.29 (-1.63,1.05)	NR	NR	NR
-0.30 (-1.29,0.68)	-0.29 (-1.63,1.05)	**PA**	0.02 (-0.77,0.81)	NR	NR
-0.34 (-1.24,0.56)	-0.32 (-1.85,1.20)	-0.03 (-0.76,0.69)	**DAA**	-0.12 (-1.57,1.32)	-1.23 (-2.76,0.30)
-0.85 (-2.36,0.66)	-0.84 (-2.78,1.11)	-0.55 (-1.96,0.87)	-0.51 (-1.72,0.70)	**MIS-ALA**	0.20 (-1.42,1.82)
-1.13 (-2.67,0.41)	-1.12 (-3.09,0.85)	-0.83 (-2.28,0.61)	-0.80 (-2.05,0.45)	-0.28 (-1.57,1.00)	**DLA**

© 2023 Yan L et al. *JAMA Network Open.*

Outcome: Hospitalization time

**PA**	NR	-0.28 (-3.32,2.76)	0.44 (-1.08,1.97)	NR	NR	**-6.23 (-8.52,-3.94)**
0.14 (-4.09,4.37)	**MIS-DLA**	NR	NR	-1.96 (-4.34,0.41)	NR	NR
-0.28 (-3.32,2.76)	-0.42 (-5.63,4.79)	**SuperPath**	NR	NR	NR	NR
-0.82 (-2.20,0.55)	-0.96 (-4.97,3.04)	-0.54 (-3.87,2.79)	**DAA**	-1.00 (-4.23,2.23)	NR	0.10 (-2.11,2.31)
-1.82 (-5.33,1.68)	-1.96 (-4.34,0.41)	-1.54 (-6.18,3.10)	-1.00 (-4.23,2.23)	**DLA**	NR	NR
**-3.29 (-6.07,-0.51)**	-3.43 (-8.29,1.44)	-3.01 (-7.12,1.11)	-2.46 (-5.23,0.30)	-1.46 (-5.71,2.79)	**2-incision**	-0.09 (-2.25,2.08)
**-3.37 (-5.11,-1.63)**	-3.51 (-7.87,0.85)	-3.09 (-6.59,0.41)	**-2.55 (-4.27,-0.83)**	-1.55 (-5.21,2.11)	-0.09 (-2.25,2.08)	**MIS-PA**

© 2023 Yan L et al. *JAMA Network Open.*

Outcome: Operation time

**MIS-PA**	-4.55 (-13.07,3.96)	-17.50 (-35.32,0.32)	NR	-3.20 (-22.90,16.50)	**-22.25 (-33.18,-11.32)**	**-14.20 (-28.37,-0.03)**	NR
-2.76 (-10.13,4.60)	**PA**	**-23.80 (-41.97,-5.63)**	-3.88 (-16.54,8.78)	-9.50 (-29.51,10.51)	NR	**-21.38 (-28.24,-14.52)**	**-36.50 (-50.44,-22.56)**
-9.48 (-20.37,1.41)	-6.72 (-16.59,3.15)	**MIS-DLA**	-1.00 (-13.44,11.44)	-6.82 (-14.60,0.95)	NR	NR	NR
**-13.14 (-23.62,-2.67)**	**-10.38 (-18.96,-1.80)**	-3.66 (-13.30,5.97)	**MIS-ALA**	NR	NR	2.10 (-11.41,15.61)	NR
**-14.47 (-25.78,-3.15)**	**-11.70 (-22.10,-1.31)**	-4.99 (-12.40,2.42)	-1.33 (-12.19,9.54)	**DLA**	NR	**-21.00 (-39.59,-2.41)**	NR
**-22.25 (-33.18,-11.32)**	**-19.49 (-32.67,-6.31)**	-12.77 (-28.20,2.66)	-9.11 (-24.25,6.03)	-7.78 (-23.51,7.95)	**2-incision**	NR	NR
**-21.59 (-29.78,-13.39)**	**-18.82 (-24.68,-12.97)**	**-12.11 (-22.21,-2.01)**	-8.44 (-17.20,0.31)	-7.12 (-17.57,3.33)	0.67 (-12.99,14.33)	**DAA**	NR
**-39.26 (-55.03,-23.50)**	**-36.50 (-50.44,-22.56)**	**-29.78 (-46.86,-12.71)**	**-26.12 (-42.49,-9.75)**	**-24.80 (-42.18,-7.41)**	-17.01 (-36.19,2.17)	**-17.68 (-32.79,-2.56)**	**SuperPath**

© 2023 Yan L et al. *JAMA Network Open.*

Outcome: Blood loss

**MIS-DLA**	NR	NR	**-327.44 (-513.69, -141.19)**	NR	NR	NR	NR
-195.81 (-544.33, 152.71)	**MIS-ALA**	NR	NR	NR	-0.00 (-269.69, 269.69)	**-228.82 (-399.47, -58.17)**	NR
-290.82 (-637.86, 56.21)	-95.01 (-275.70, 85.67)	**MIS-PA**	NR	-46.00 (-228.34, 136.34)	NR	-71.09 (-172.43, 30.24)	NR
**-327.44 (-513.69, -141.19)**	-131.63 (-426.21, 162.95)	-36.62 (-329.44, 256.20)	**DLA**	NR	-25.00 (-261.86, 211.86)	NR	NR
-336.82 (-728.85, 55.20)	-141.01 (-397.71, 115.69)	-46.00 (-228.34, 136.34)	-9.38 (-354.33, 335.57)	**2-incision**	NR	NR	NR
**-352.44 (-653.76, -51.13)**	-156.63 (-331.78, 18.51)	-61.62 (-233.79, 110.56)	-25.00 (-261.86, 211.86)	-15.62 (-266.40, 235.16)	**DAA**	42.05 (-112.63, 196.73)	NR
**-361.92 (-693.83, -30.01)**	**-166.11 (-315.70, -16.51)**	-71.09 (-172.43, 30.24)	-34.48 (-309.21, 240.26)	-25.09 (-233.70, 183.51)	-9.48 (-148.67, 129.72)	**PA**	-162.83 (-348.36, 22.70)
**-524.75 (-905.00, -144.50)**	**-328.94 (-567.27, -90.61)**	**-233.92 (-445.33, -22.52)**	-197.31 (-528.81, 134.20)	-187.92 (-467.10, 91.25)	-172.31 (-404.25, 59.64)	-162.83 (-348.36, 22.70)	**SuperPath**

© 2023 Yan L et al. *JAMA Network Open.*

Outcome: Quality of life socre change

**MIS-ALA**	NR	0.54 (-2.07,3.15)	NR	6.90 (-7.72,21.52)	NR
0.30 (-2.85,3.45)	**MIS-PA**	0.71 (-1.55,2.97)	-0.00 (-3.11,3.11)	NR	1.86 (-1.31,5.03)
0.73 (-1.83,3.30)	0.43 (-1.40,2.26)	**PA**	0.10 (-0.02,0.22)	NR	NR
0.83 (-1.74,3.40)	0.53 (-1.30,2.36)	0.10 (-0.02,0.22)	**DAA**	0.01 (-0.06,0.08)	NR
0.84 (-1.73,3.41)	0.54 (-1.29,2.37)	0.11 (-0.03,0.25)	0.01 (-0.06,0.08)	**DLA**	NR
2.16 (-2.31,6.63)	1.86 (-1.31,5.03)	1.43 (-2.23,5.09)	1.33 (-2.33,4.99)	1.32 (-2.34,4.98)	**2-incision**

© 2023 Yan L et al. *JAMA Network Open.*

Outcome: Cup abduction angle

**MIS-PA**	-1.64 (-4.47, 1.19)	.	-0.01 (-2.77, 2.74)	.
-0.72 (-2.92, 1.47)	**PA**	0.60 (-2.90, 4.10)	-1.11 (-3.51, 1.28)	**-5.72 (-9.90, -1.54)**
-0.77 (-3.82, 2.27)	-0.05 (-2.59, 2.50)	**MIS-ALA**	0.40 (-2.69, 3.49)	.
-0.88 (-3.06, 1.30)	-0.16 (-2.03, 1.72)	-0.11 (-2.56, 2.35)	**DAA**	.
**-6.44 (-11.17, -1.72)**	**-5.72 (-9.90, -1.54)**	**-5.67 (-10.57, -0.78)**	**-5.56 (-10.15, -0.98)**	**SuperPath**

© 2023 Yan L et al. *JAMA Network Open.*

Outcome: Cup anteversion angle

**PA**	0.97 (-3.28,5.22)	-0.30 (-5.66,5.06)	-0.95 (-6.95,5.05)	-2.30 (-6.47,1.87)
-0.18 (-3.38,3.01)	**DAA**	-0.10 (-5.57,5.37)	NR	0.35 (-4.29,5.00)
-0.29 (-4.43,3.84)	-0.11 (-4.27,4.05)	**MIS-ALA**	NR	NR
-0.95 (-6.95,5.05)	-0.77 (-7.56,6.03)	-0.66 (-7.94,6.63)	**SuperPath**	NR
-1.20 (-4.61,2.22)	-1.01 (-4.59,2.56)	-0.91 (-5.83,4.02)	-0.25 (-7.15,6.65)	**MIS-PA**

© 2023 Yan L et al. *JAMA Network Open.*

eTable 10K. Inclusion of studies in which all patients underwent spinal anesthesia

Outcome: Hip score change

**DAA**	1.40 (-5.29,8.08)	3.69 (-2.46,9.84)	NR	NR	1.00 (-7.01,9.01)	NR	**6.30 (1.30,11.29)**
1.02 (-4.45,6.49)	**MIS-ALA**	6.20 (-5.74,18.14)	1.81 (-6.11,9.73)	NR	NR	NR	NR
2.62 (-1.92,7.17)	1.60 (-4.70,7.91)	**MIS-DLA**	3.90 (-5.26,13.06)	NR	8.50 (-1.11,18.11)	NR	3.78 (-2.36,9.92)
3.48 (-1.85,8.81)	2.46 (-3.39,8.32)	0.86 (-4.95,6.67)	**PA**	NR	1.19 (-2.93,5.31)	2.64 (-4.61,9.89)	3.30 (-5.32,11.92)
4.27 (-5.35,13.89)	3.25 (-7.01,13.50)	1.64 (-8.33,11.61)	0.78 (-8.22,9.79)	**2-incision**	0.00 (-8.10,8.10)	NR	NR
4.27 (-0.91,9.45)	3.25 (-3.04,9.53)	1.64 (-4.16,7.45)	0.78 (-3.13,4.70)	-0.00 (-8.10,8.10)	**MIS-PA**	NR	-1.30 (-10.40,7.80)
6.12 (-2.88,15.13)	5.10 (-4.22,14.42)	3.50 (-5.80,12.80)	2.64 (-4.61,9.89)	1.86 (-9.71,13.42)	1.86 (-6.39,10.10)	**SuperPath**	NR
**5.83 (1.76,9.91)**	4.81 (-1.49,11.12)	3.21 (-1.61,8.03)	2.35 (-3.30,8.00)	1.57 (-8.28,11.42)	1.57 (-4.03,7.16)	-0.29 (-9.48,8.90)	**DLA**

© 2023 Yan L et al. *JAMA Network Open.*

Outcome: Pain score change

**DAA**	NR	-0.12 (-0.79,0.54)	-0.13 (-0.55,0.29)	-0.20 (-0.71,0.31)	NR
-0.13 (-0.95,0.69)	**SuperPath**	NR	NR	NR	-0.14 (-0.48,0.20)
-0.13 (-0.79,0.54)	0.01 (-1.04,1.06)	**MIS-ALA**	NR	NR	NR
-0.13 (-0.55,0.29)	0.00 (-0.92,0.92)	-0.00 (-0.79,0.78)	**MIS-DLA**	NR	NR
-0.20 (-0.71,0.31)	-0.07 (-0.70,0.57)	-0.08 (-0.91,0.76)	-0.07 (-0.74,0.60)	**MIS-PA**	-0.07 (-0.61,0.46)
-0.27 (-1.01,0.47)	-0.14 (-0.48,0.20)	-0.15 (-1.14,0.85)	-0.14 (-1.00,0.71)	-0.07 (-0.61,0.46)	**PA**

© 2023 Yan L et al. *JAMA Network Open.*

Outcome: Hospitalization time

**SuperPath**	**-4.35 (-8.46,-0.24)**	NR	NR	NR	NR	NR	NR
**-4.35 (-8.46,-0.24)**	**PA**	NR	NR	**-4.04 (-6.40,-1.67)**	NR	NR	NR
**-8.31 (-14.51,-2.10)**	-3.96 (-8.59,0.68)	**DAA**	NR	-0.08 (-4.07,3.91)	-1.00 (-5.58,3.58)	-0.80 (-3.86,2.27)	-0.17 (-2.48,2.14)
**-8.30 (-13.83,-2.77)**	**-3.95 (-7.64,-0.26)**	0.01 (-4.89,4.90)	**2-incision**	-0.09 (-2.92,2.75)	NR	NR	NR
**-8.39 (-13.13,-3.64)**	**-4.04 (-6.40,-1.67)**	-0.08 (-4.07,3.91)	-0.09 (-2.92,2.75)	**MIS-PA**	NR	NR	NR
**-8.52 (-16.00,-1.04)**	-4.17 (-10.42,2.08)	-0.22 (-4.40,3.97)	-0.22 (-6.66,6.22)	-0.14 (-5.92,5.65)	**MIS-ALA**	-1.00 (-5.73,3.73)	NR
**-8.63 (-15.35,-1.91)**	-4.28 (-9.59,1.03)	-0.32 (-2.91,2.26)	-0.33 (-5.87,5.21)	-0.24 (-5.00,4.51)	-0.11 (-4.34,4.12)	**MIS-DLA**	-1.00 (-5.19,3.19)
**-8.74 (-15.29,-2.19)**	-4.39 (-9.49,0.70)	-0.44 (-2.55,1.67)	-0.44 (-5.78,4.89)	-0.36 (-4.88,4.16)	-0.22 (-4.75,4.30)	-0.11 (-2.95,2.72)	**DLA**

© 2023 Yan L et al. *JAMA Network Open.*

Outcome: Operation time

**MIS-PA**	-4.15 (-9.93,1.62)	**-17.50 (-33.98,-1.02)**	NR	NR	-3.20 (-21.69,15.29)	**-22.23 (-32.04,-12.42)**	NR
-4.69 (-10.38,1.01)	**PA**	**-23.80 (-40.65,-6.95)**	-3.88 (-14.56,6.80)	NR	-9.50 (-28.32,9.32)	NR	**-19.39 (-29.29,-9.49)**
-7.91 (-18.26,2.45)	-3.22 (-12.88,6.45)	**MIS-DLA**	-8.00 (-18.78,2.78)	-7.82 (-16.63,0.98)	-9.27 (-19.16,0.62)	NR	NR
**-12.00 (-21.83,-2.18)**	-7.31 (-16.03,1.40)	-4.09 (-11.84,3.65)	**MIS-ALA**	-3.19 (-12.21,5.83)	NR	NR	NR
**-13.99 (-24.62,-3.37)**	-9.30 (-19.19,0.58)	-6.09 (-12.71,0.53)	-1.99 (-9.49,5.50)	**DAA**	-3.41 (-9.25,2.42)	NR	NR
**-17.67 (-28.45,-6.88)**	**-12.98 (-23.11,-2.85)**	**-9.76 (-16.80,-2.72)**	-5.66 (-14.05,2.72)	-3.67 (-8.93,1.59)	**DLA**	NR	NR
**-22.23 (-32.04,-12.42)**	**-17.54 (-28.88,-6.20)**	**-14.32 (-28.58,-0.06)**	-10.23 (-24.11,3.65)	-8.24 (-22.70,6.22)	-4.56 (-19.14,10.02)	**2-incision**	NR
**-24.08 (-35.50,-12.66)**	**-19.39 (-29.29,-9.49)**	**-16.17 (-30.01,-2.34)**	-12.08 (-25.27,1.12)	-10.09 (-24.08,3.91)	-6.41 (-20.58,7.76)	-1.85 (-16.90,13.21)	**SuperPath**

© 2023 Yan L et al. *JAMA Network Open.*

Outcome: Blood loss

**MIS-DLA**	-60.00 (-533.46, 413.46)	-10.07 (-128.82, 108.68)	NR	**-600.00 (-882.09, -317.91)**	NR	NR	NR
-71.27 (-302.12, 159.58)	**MIS-ALA**	-15.20 (-232.68, 202.27)	NR	NR	NR	NR	**-228.82 (-363.44, -94.20)**
-84.54 (-193.96, 24.87)	-13.27 (-223.97, 197.43)	**DAA**	NR	-54.82 (-132.72, 23.08)	NR	NR	NR
-161.47 (-452.93, 129.99)	-90.20 (-268.12, 87.72)	-76.93 (-352.70, 198.84)	**SuperPath**	NR	NR	NR	**-138.62 (-254.94, -22.30)**
**-172.01 (-298.39, -45.62)**	-100.74 (-324.07, 122.59)	**-87.46 (-162.95, -11.97)**	-10.54 (-296.07, 275.00)	**DLA**	NR	NR	NR
-232.43 (-511.64, 46.78)	**-161.16 (-318.21, -4.11)**	-147.89 (-410.68, 114.91)	-70.96 (-212.64, 70.72)	-60.42 (-333.45, 212.60)	**MIS-PA**	-46.00 (-195.16, 103.16)	-67.66 (-148.55, 13.23)
-278.43 (-594.99, 38.13)	-207.16 (-423.76, 9.44)	-193.89 (-496.06, 108.29)	-116.96 (-322.69, 88.77)	-106.42 (-417.53, 204.69)	-46.00 (-195.16, 103.16)	**2-incision**	NR
**-300.09 (-567.33, -32.85)**	**-228.82 (-363.44, -94.20)**	-215.55 (-465.58, 34.49)	**-138.62 (-254.94, -22.30)**	-128.08 (-388.85, 132.68)	-67.66 (-148.55, 13.23)	-21.66 (-191.34, 148.02)	**PA**

© 2023 Yan L et al. *JAMA Network Open.*

Outcome: Quality of life socre change

**MIS-DLA**	NR	3.26 (-4.80,11.32)	NR	NR	NR	NR
3.26 (-6.37,12.89)	**MIS-PA**	-0.00 (-5.28,5.28)	NR	NR	0.71 (-4.12,5.54)	2.04 (-2.35,6.43)
3.26 (-4.80,11.32)	-0.00 (-5.28,5.28)	**DAA**	-0.00 (-4.27,4.26)	NR	NR	NR
3.26 (-5.86,12.37)	-0.00 (-6.79,6.78)	-0.00 (-4.27,4.26)	**DLA**	NR	NR	NR
3.43 (-8.45,15.31)	0.17 (-6.78,7.12)	0.17 (-8.56,8.90)	0.17 (-9.54,9.88)	**MIS-ALA**	0.54 (-4.46,5.54)	NR
3.97 (-6.80,14.74)	0.71 (-4.12,5.54)	0.71 (-6.44,7.86)	0.71 (-7.62,9.04)	0.54 (-4.46,5.54)	**PA**	NR
5.30 (-5.28,15.89)	2.04 (-2.35,6.43)	2.04 (-4.82,8.91)	2.04 (-6.04,10.13)	1.87 (-6.35,10.09)	1.33 (-5.19,7.86)	**2-incision**

© 2023 Yan L et al. *JAMA Network Open.*

Outcome: Cup abduction angle

**MIS-PA**	0.11 (-3.45,3.68)	NR	NR	NR	-2.00 (-8.11,4.11)
-0.21 (-3.58,3.17)	**PA**	NR	0.60 (-5.54,6.74)	NR	NR
-0.44 (-7.04,6.17)	-0.23 (-7.10,6.64)	**DLA**	NR	-0.80 (-6.75,5.15)	-0.61 (-4.81,3.59)
-0.56 (-6.01,4.88)	-0.36 (-5.46,4.75)	-0.13 (-6.66,6.40)	**MIS-ALA**	NR	0.40 (-5.51,6.31)
-1.24 (-10.13,7.65)	-1.03 (-10.12,8.06)	-0.80 (-6.75,5.15)	-0.67 (-9.51,8.16)	**MIS-DLA**	NR
-1.05 (-6.15,4.04)	-0.84 (-6.29,4.60)	-0.61 (-4.81,3.59)	-0.49 (-5.49,4.51)	0.19 (-7.10,7.47)	**DAA**

© 2023 Yan L et al. *JAMA Network Open.*

Outcome: Cup anteversion angle

**DAA**	-0.10 (-8.07,7.87)	NR	-2.00 (-10.13,6.13)
-0.79 (-7.47,5.89)	**MIS-ALA**	0.30 (-7.59,8.19)	NR
-1.17 (-8.37,6.03)	-0.38 (-7.02,6.27)	**PA**	0.13 (-4.57,4.83)
-1.28 (-8.04,5.48)	-0.49 (-7.64,6.66)	-0.11 (-4.56,4.34)	**MIS-PA**

© 2023 Yan L et al. *JAMA Network Open.*

eFigure 1. Schematic Showing the Entrance Location of the 8 Surgical Approaches for THA

The line segments in the figure indicate the location and length of the incisions for each approach. DAA=direct anterior approach. DLA=direct lateral approach. MIS-DLA=minimally invasive direct lateral approach. MIS-ALA=minimally invasive anterolateral approach. PA=posterior approach. MIS-PA=minimally invasive posterior approach. SuperPath=supercapsular percutaneously assisted total hip arthroplasty.

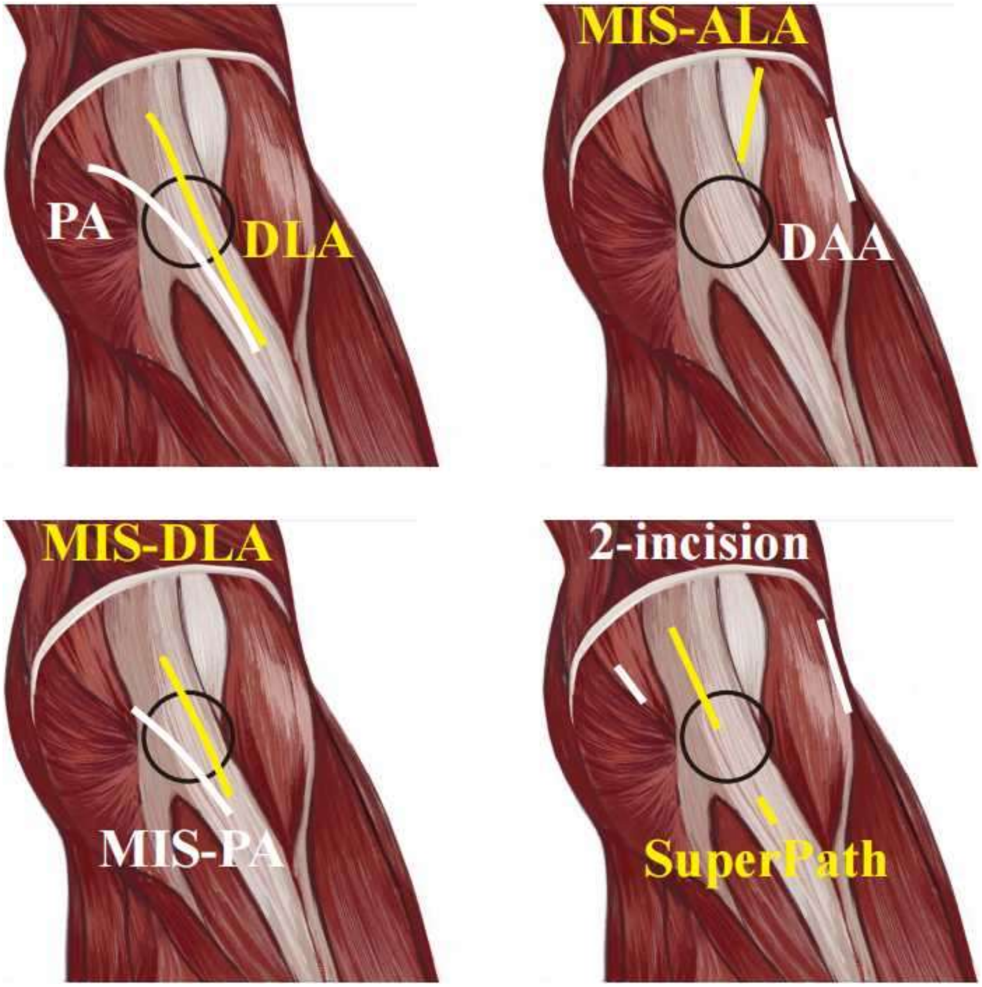

© 2023 Yan L et al. *JAMA Network Open.*

eFigure 2. Network Plots for Other Outcome Measures

Outcome : Short-term hip score

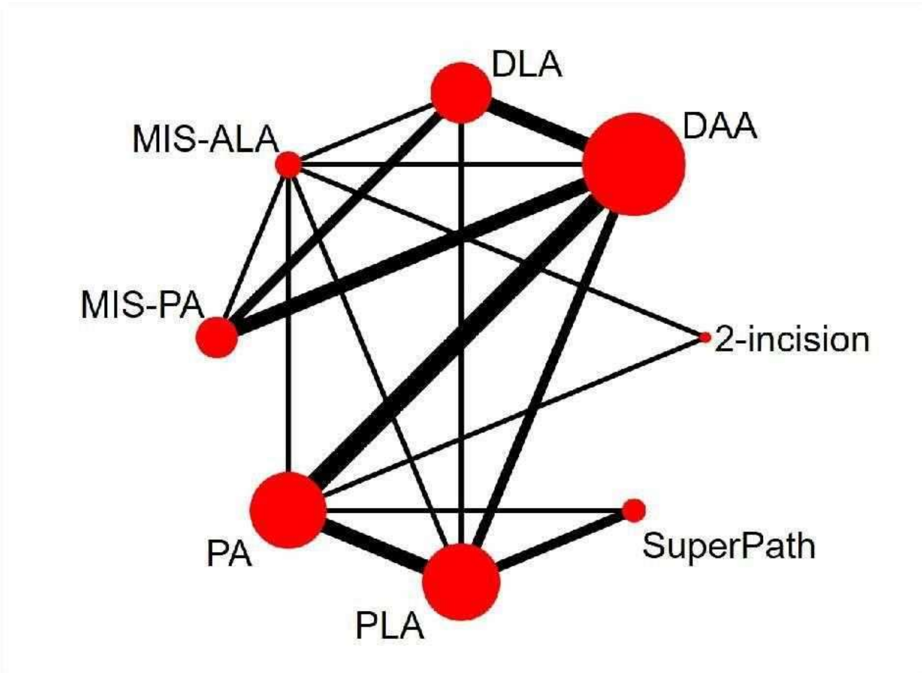

Outcome : Long-term hip score

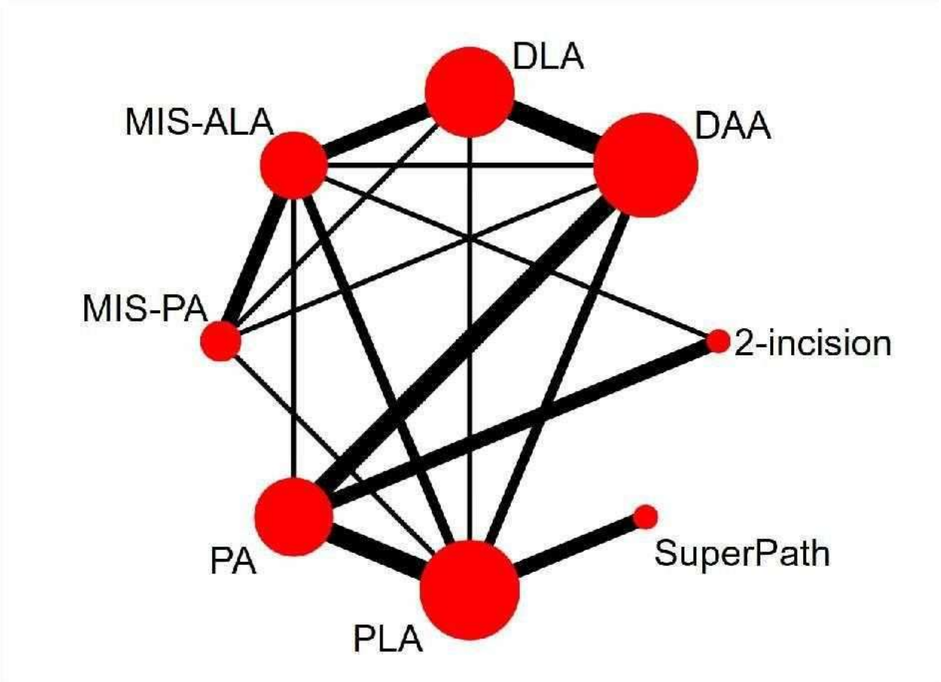

Outcome : Dislocation

© 2023 Yan L et al. *JAMA Network Open.*

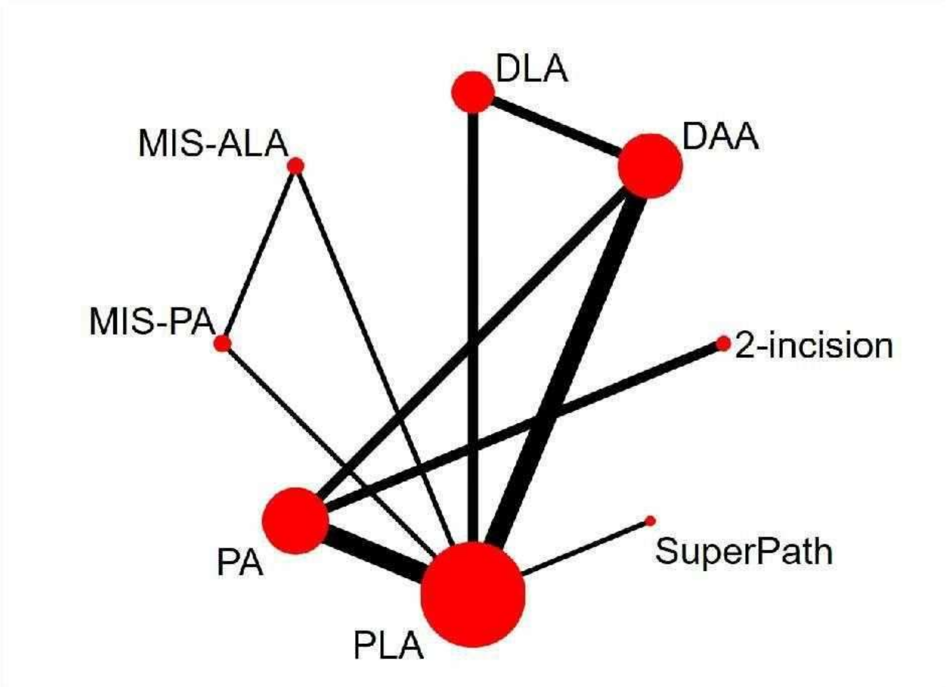

Outcome : Fracture

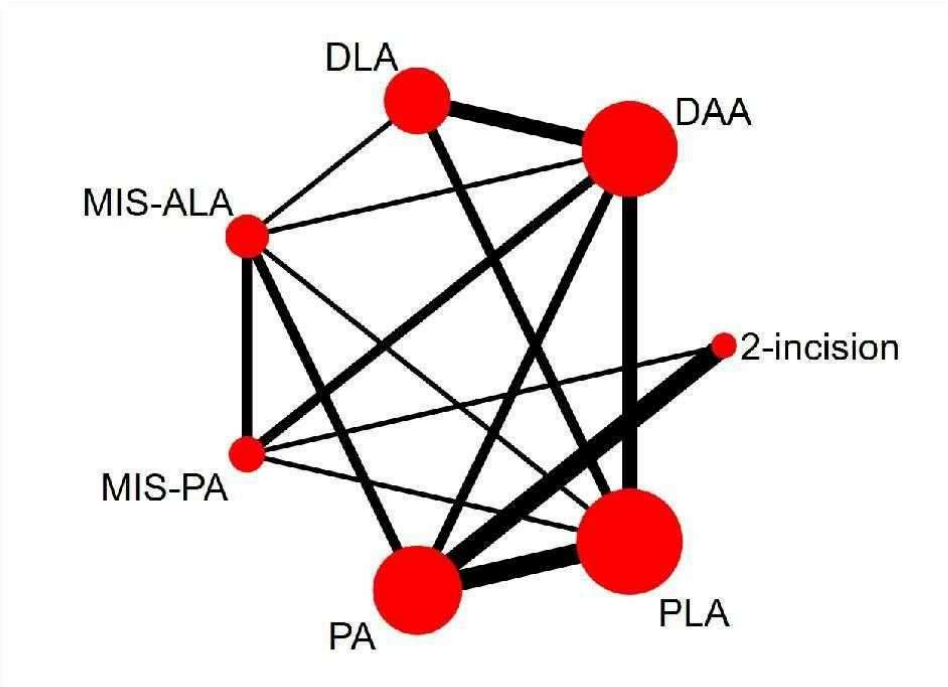

© 2023 Yan L et al. *JAMA Network Open.*

Outcome : Infection

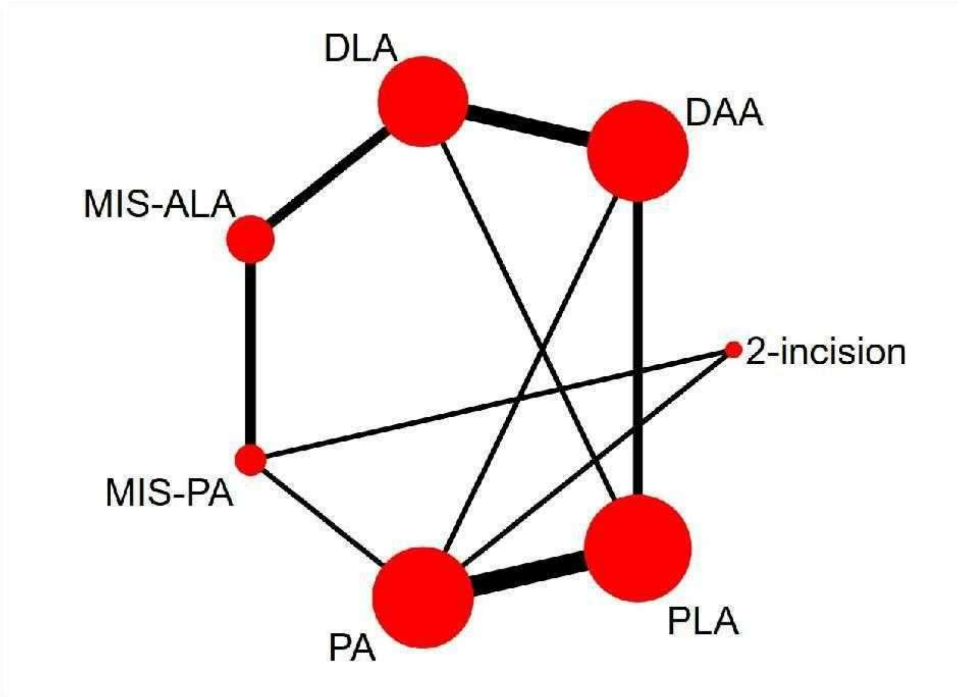

Outcome : Nerve injury

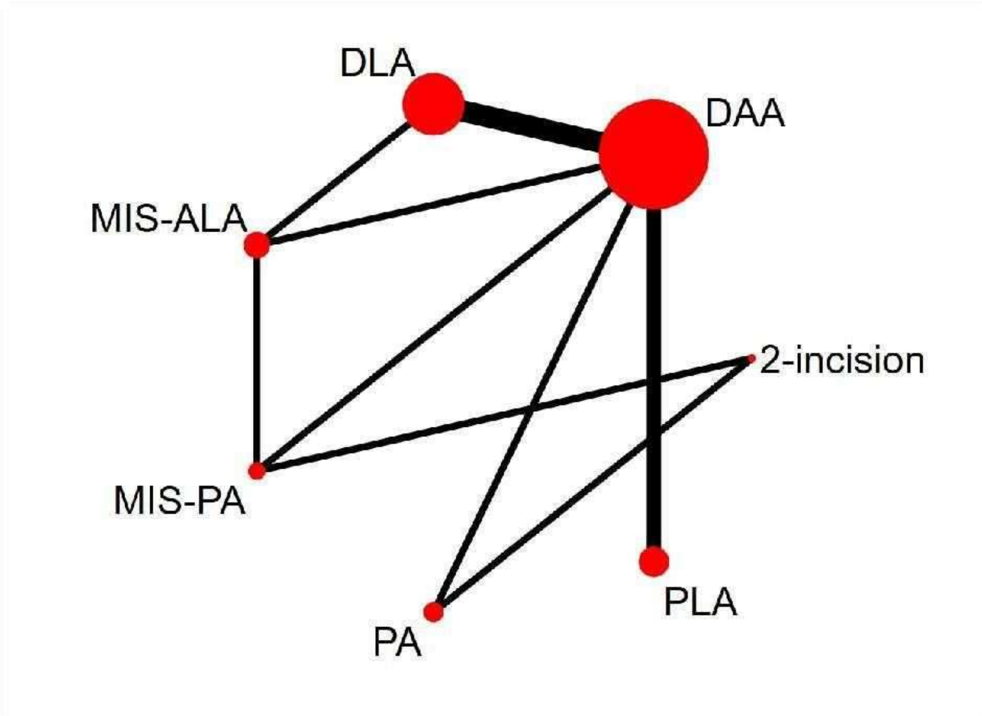

© 2023 Yan L et al. *JAMA Network Open.*

Outcome : Reoperation

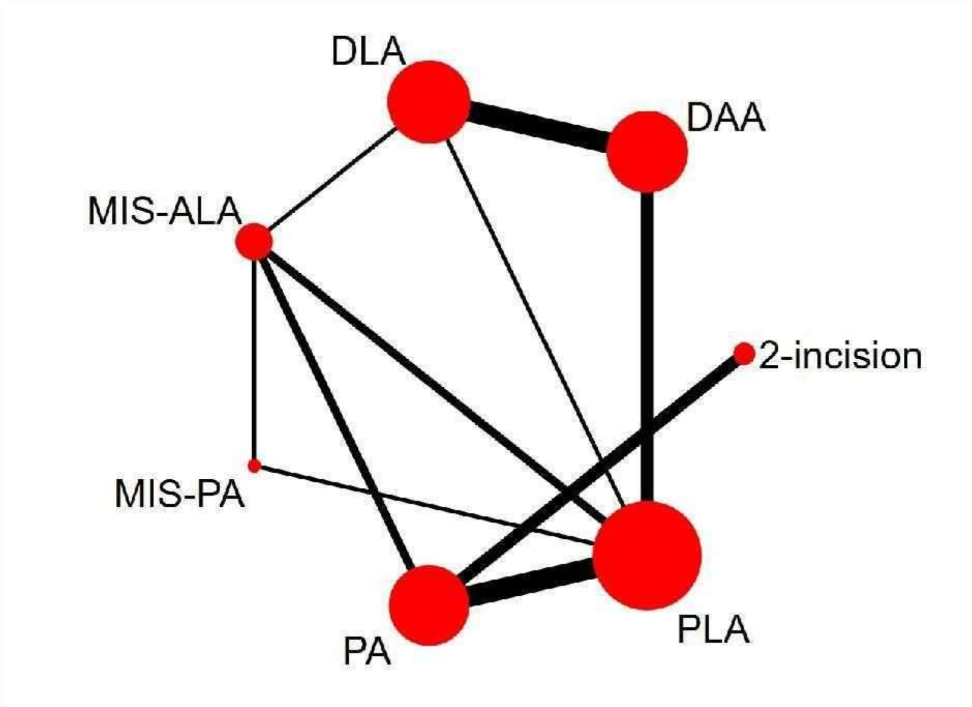

Outcome : Thromboembolism

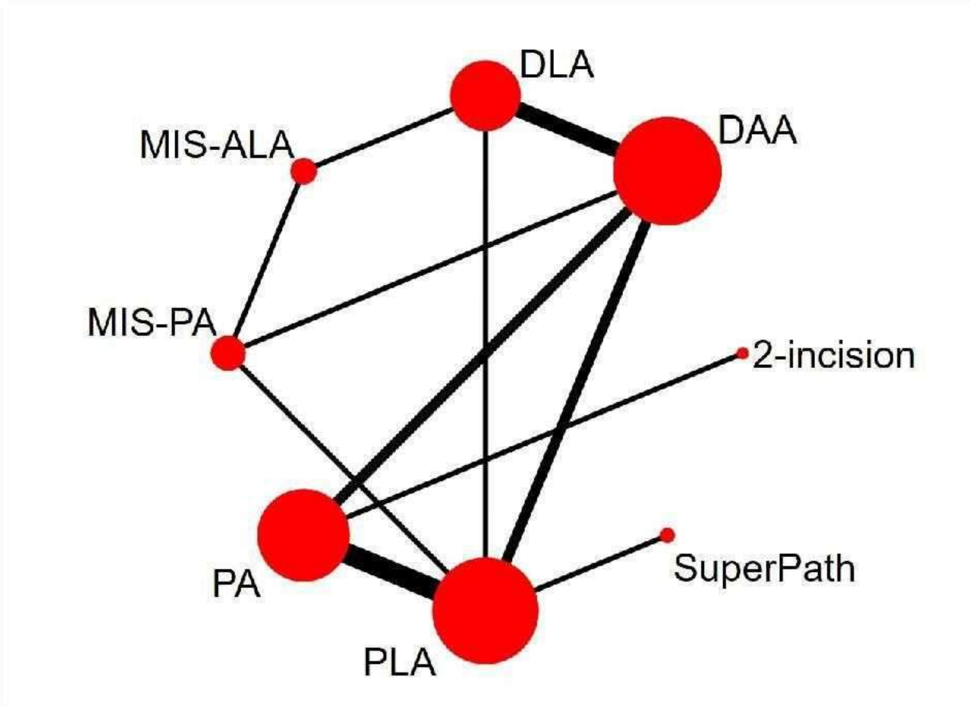

© 2023 Yan L et al. *JAMA Network Open.*

Outcome : Abductor muscle strengths change

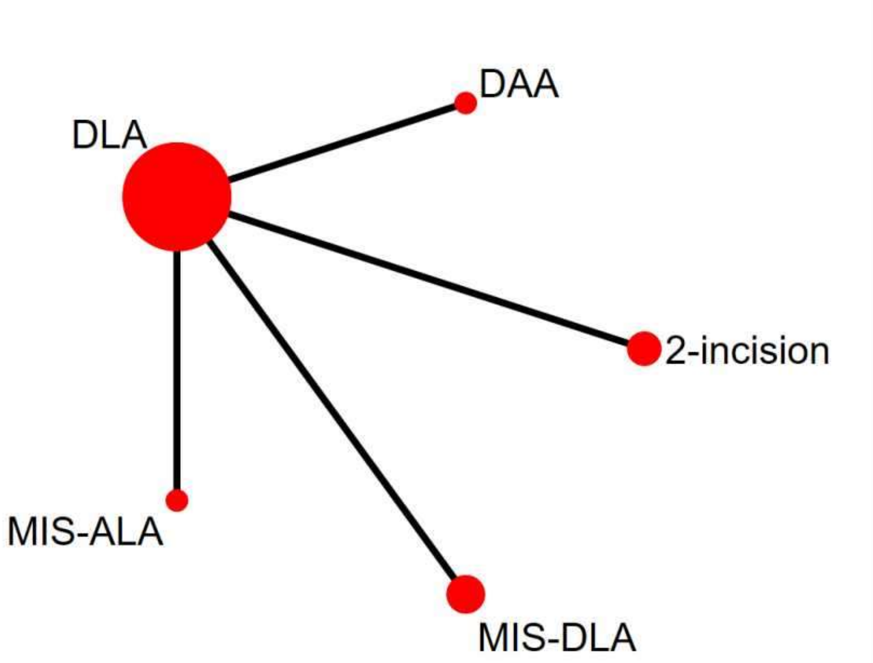

Outcome : Analgesic consumption

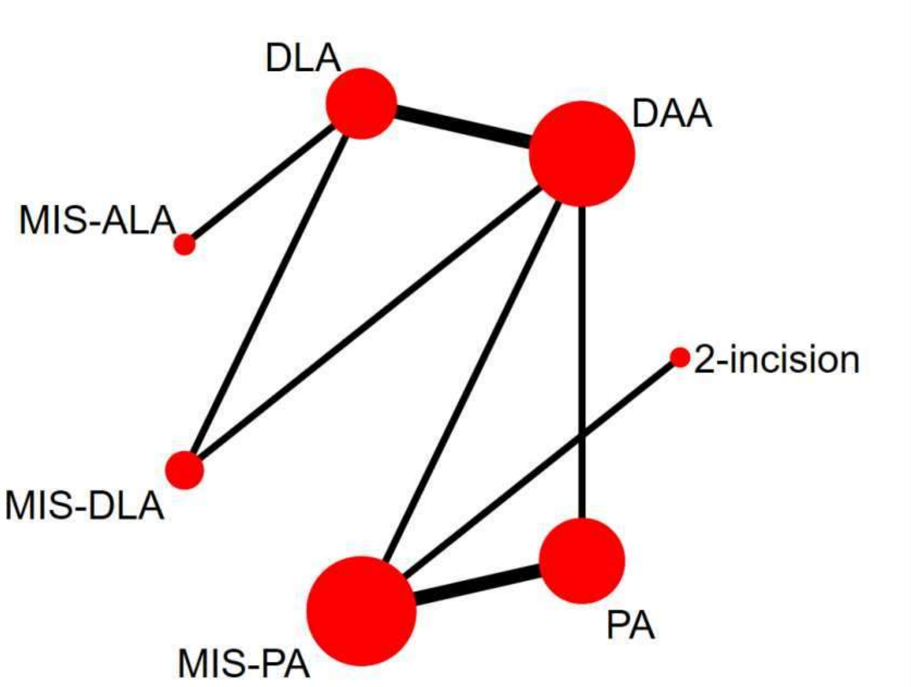

© 2023 Yan L et al. *JAMA Network Open.*

Outcome : Cadence change

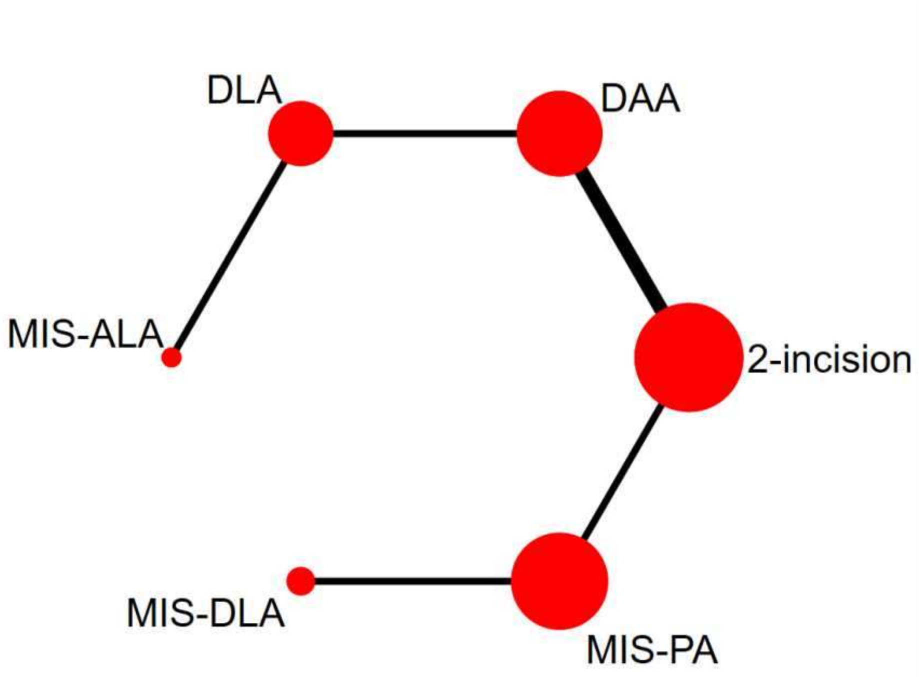

Outcome : Creatine kinase change

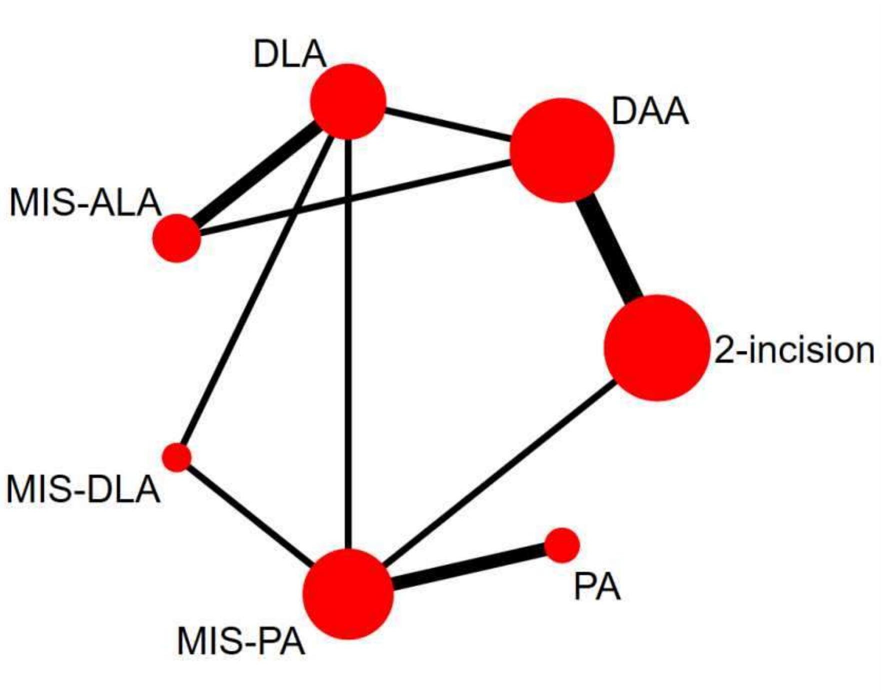

© 2023 Yan L et al. *JAMA Network Open.*

Outcome : C-reactive protein change

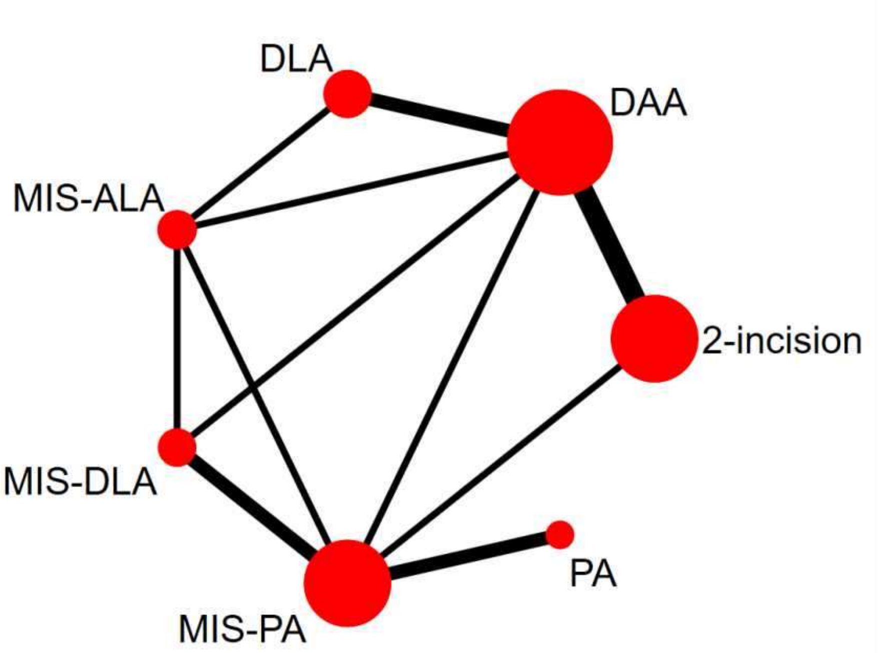

Outcome : Erythrocyte sedimentation rate change

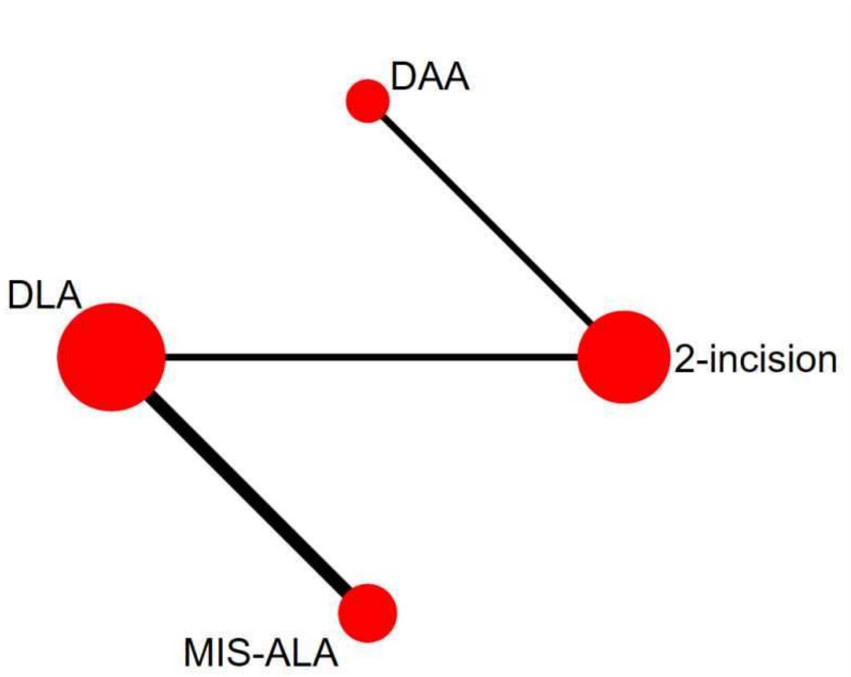

© 2023 Yan L et al. *JAMA Network Open.*

Outcome : Hemoglobin change

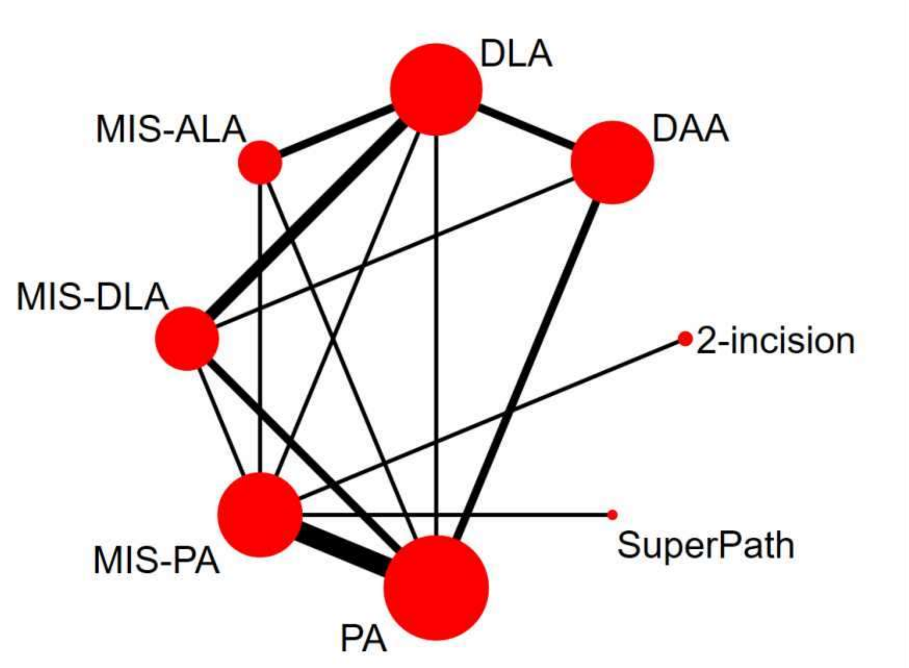

Outcome : Hematocrit change

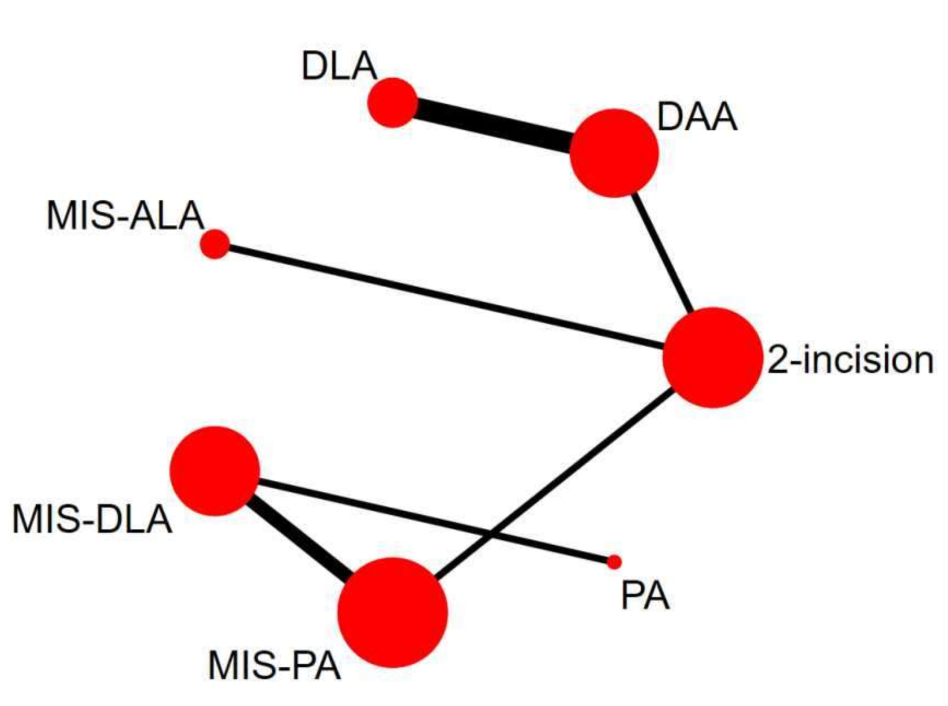

© 2023 Yan L et al. *JAMA Network Open.*

Outcome : Interleukin-6 change

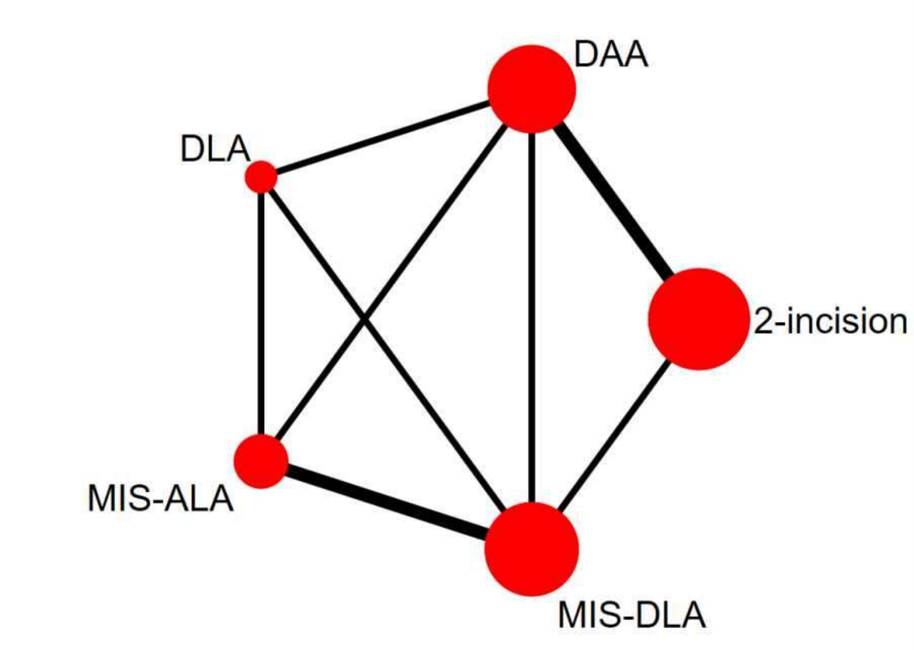

Outcome : Leg length discrepancy

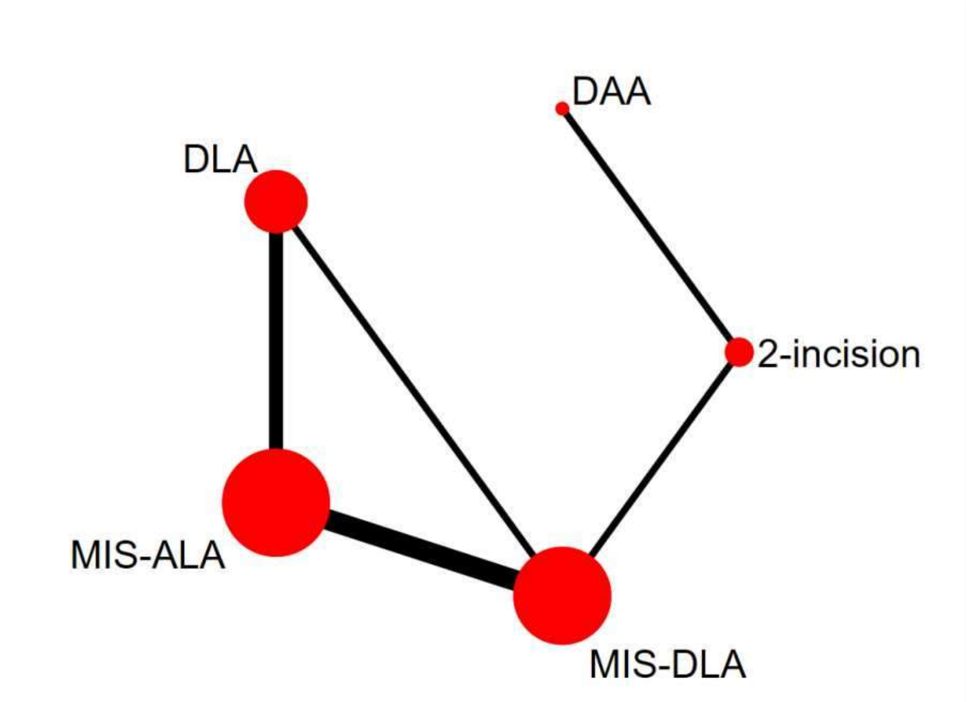

© 2023 Yan L et al. *JAMA Network Open.*

Outcome : Myoglobin change

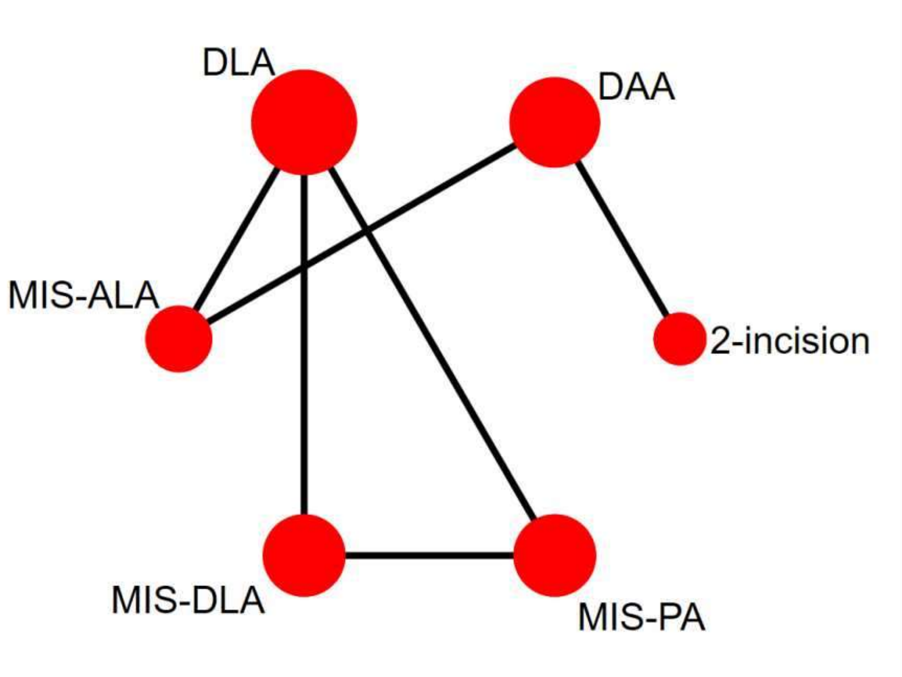

Outcome : Stem alignment

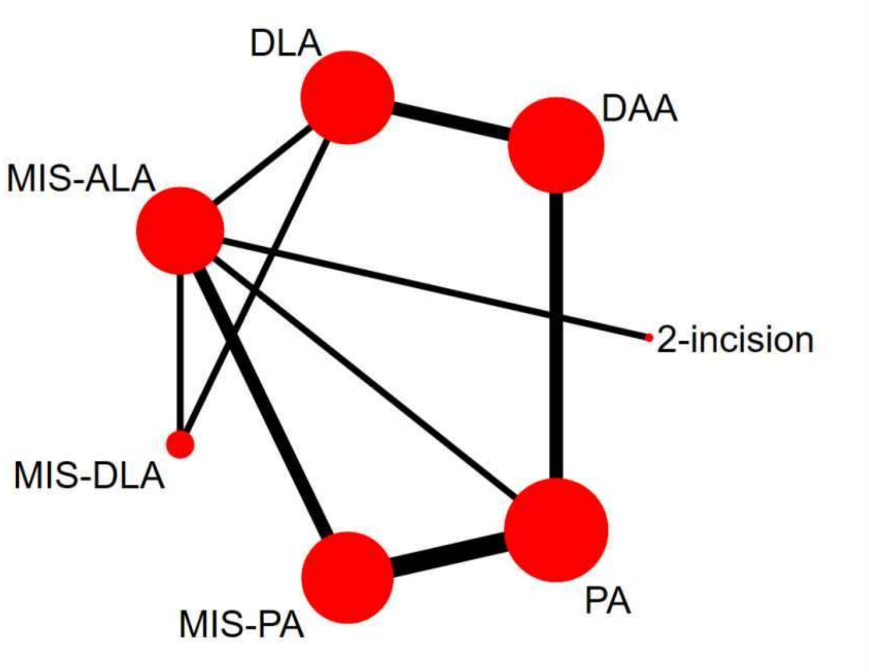

© 2023 Yan L et al. *JAMA Network Open.*

Outcome : Step length change

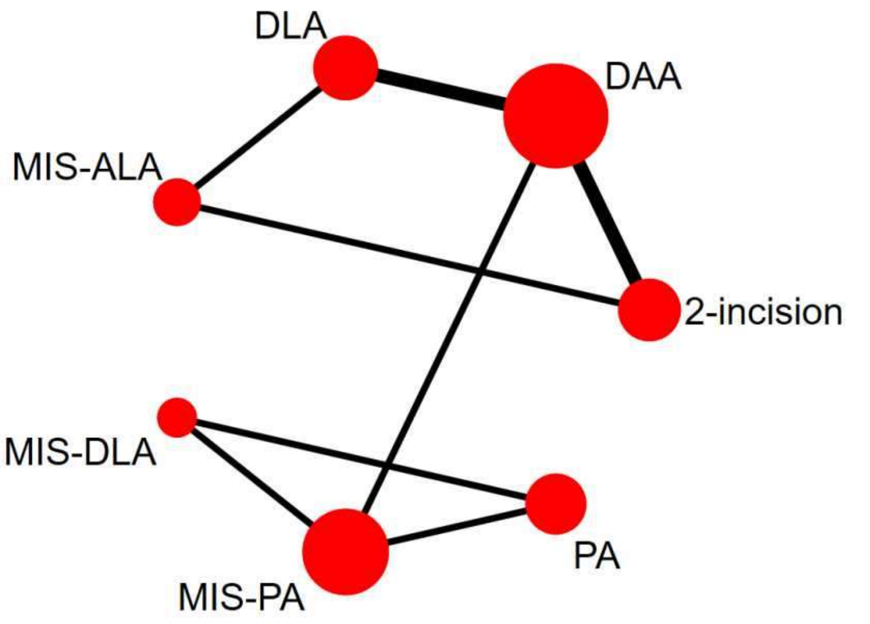

Outcome : Timed Up and Go Test change

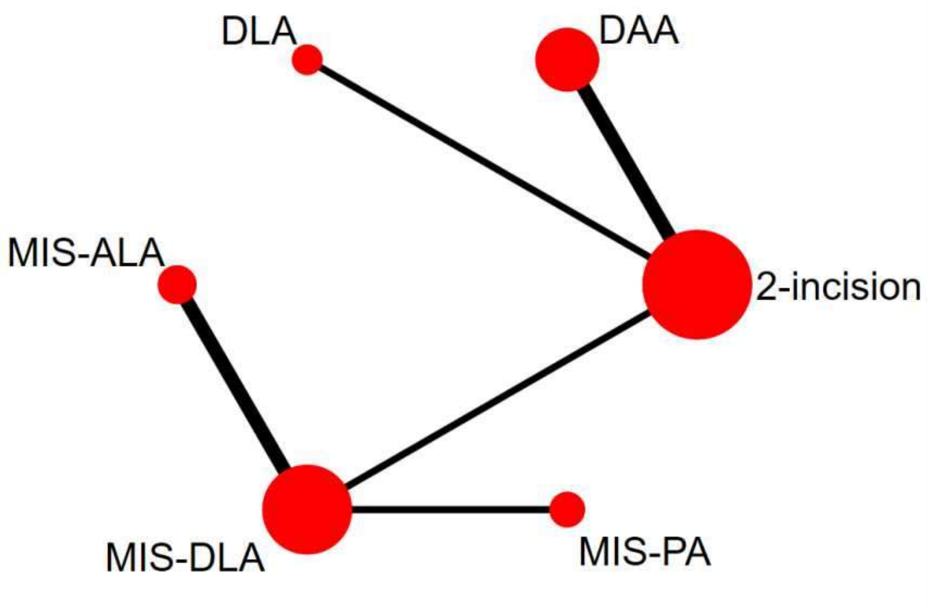

© 2023 Yan L et al. *JAMA Network Open.*

Outcome : Volume of blood transfusion change

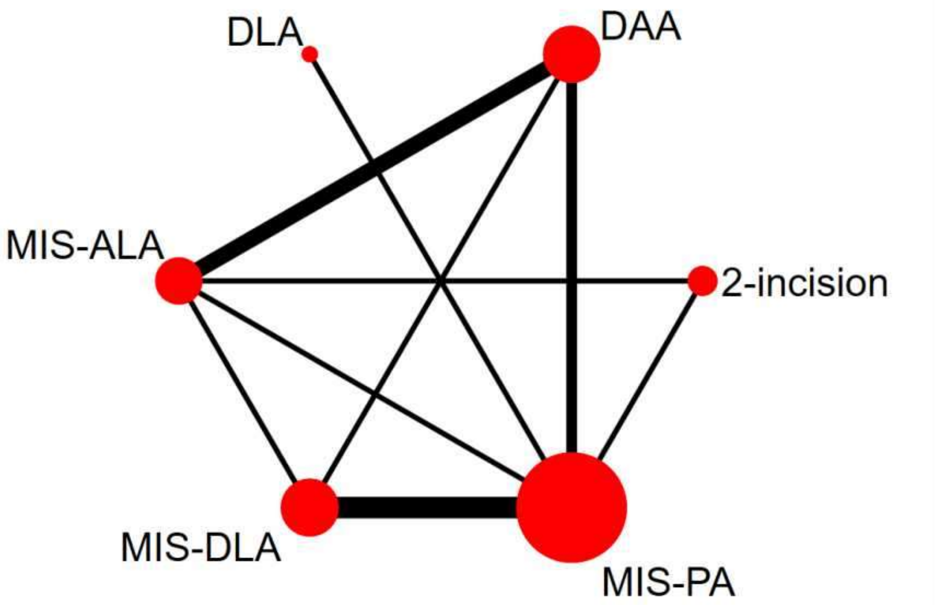

Outcome : Walking speed change

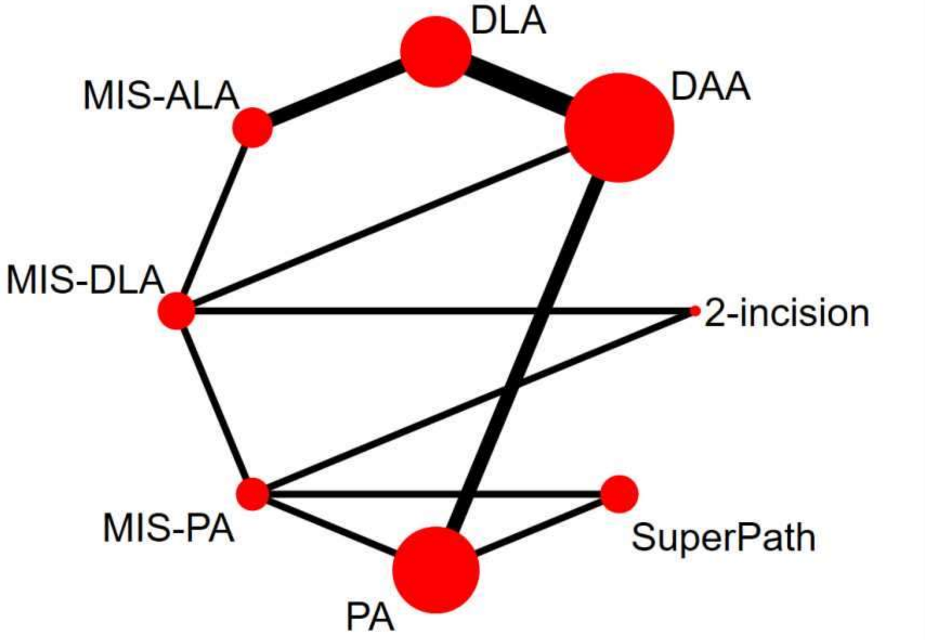

© 2023 Yan L et al. *JAMA Network Open.*

eFigure 3. Risk of Bias Assessments

eFigure 3A. Risk of bias assessment for the individual domains

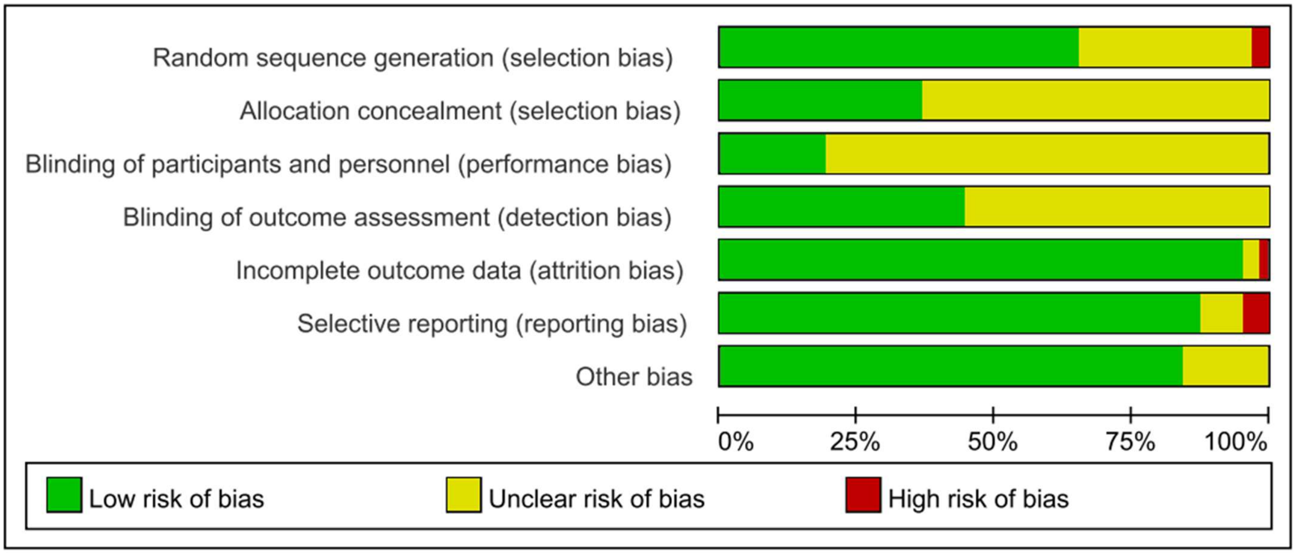

© 2023 Yan L et al. *JAMA Network Open.*

eFigure 3B. Risk of bias results for each article

	Random sequence generation (selection bias)	Allocation concealment (selection bias)	Blinding of participants and personnel (performance bias)	Blinding of outcome assessment (detection bias)	Incomplete outcome data (attrition bias)	Selective reporting (reporting bias)	Other bias
Abdel2017	?	?	?	?	+	+	+
Barrett 2019	+	?	?	?	+	+	+
Bon2019	+	?	?	?	+	−	+
Brismar2018	+	+	?	?	+	+	?
Cao2020	+	+	?	+	+	+	+
Catma 2017	−	?	?	?	+	+	+
Cheng2017	+	+	?	?	+	+	+
Chimento2005	?	?	?	?	+	+	+
Christensen 2015	?	?	?	?	+	−	?
D'Arrigo2009	?	?	?	?	?	+	+
De Anta-Diza2016	+	?	?	+	+	+	+
Della2010	+	+	?	+	+	+	?
Dienstknecht2014	?	?	?	?	+	+	+
Dorr2007	?	?	+	+	+	+	?
Dutka2007	−	?	?	+	+	+	+
Gossen2011	+	+	+	+	+	+	+
Hu 2012	?	?	?	?	+	+	+
Inaba2011	?	?	?	+	?	+	+
Ji2012	+	?	?	?	+	+	+
Khan 2012	+	+	+	+	+	+	+
Kim2006	+	?	?	?	+	+	+
Korytkin2021	+	+	?	+	+	+	?
Laffosse2008	?	?	?	?	+	+	+
Landgraeber2013	+	?	?	+	+	+	+

eFigure 4. Contribution Matrices

Outcome : Hip score change

Direct comparisons in the network

Network meta-analysis estimates	AvsD	AvsF	BvsC	BvsD	BvsE	BvsF	BvsG	CvsD	CvsE	CvsF	CvsG	DvsE	DvsF	DvsG	EvsF	EvsG	FvsG	FvsH	GvsH
Mixed estimates																			
AvsD	7.0	29.4	1.9	6.8	2.0	10.5	0.9	5.6	0.5	2.4	0.8	3.7	2.7	10.6	1.9	0.4	9.8	1.0	1.0
AvsF	10.5	63.5	0.8	2.6	0.8	4.6	0.4	2.2	0.2	1.0	0.3	1.4	1.1	4.1	0.7	0.1	3.9	0.4	0.4
BvsC	0.5	0.5	12.2	7.4	8.8	10.0	9.5	12.5	16.9	5.4	4.4	2.3	1.1	5.8	2.2	1.1	1.6	0.2	0.2
BvsD	1.4	1.4	5.8	14.9	6.1	12.0	12.7	9.6	1.2	1.7	0.9	6.5	3.1	16.1	1.2	0.4	4.3	0.4	0.4
BvsE	0.4	0.5	7.4	6.9	14.8	10.5	8.7	4.3	16.8	2.8	2.2	9.4	1.0	5.4	4.8	2.5	1.3	0.1	0.1
BvsF	0.9	0.9	3.3	4.7	3.6	41.0	14.6	0.2	0.5	3.1	0.5	0.3	2.0	2.2	2.5	0.3	16.0	1.7	1.7
BvsG	0.1	0.1	3.6	6.2	3.8	16.5	29.2	1.7	0.8	0.2	2.5	1.4	0.2	9.6	0.3	1.3	16.9	1.8	1.8
CvsD	0.9	0.9	7.9	7.7	3.1	0.4	2.8	25.0	14.8	4.4	4.1	9.7	2.0	10.8	1.2	0.9	2.7	0.3	0.3
CvsE	0.1	0.1	7.0	0.7	9.5	0.7	1.0	10.9	44.6	3.7	3.1	10.4	0.1	0.5	3.8	2.1	0.5	0.1	0.1
CvsF	1.0	1.0	8.6	3.5	5.1	16.3	0.9	10.1	10.9	6.7	4.2	2.2	2.3	6.5	3.5	1.1	10.6	1.2	1.2
CvsG	0.4	0.4	8.5	2.2	4.8	3.3	12.3	12.5	12.0	5.0	5.7	3.0	0.9	12.1	2.2	1.9	10.6	1.1	1.1
DvsE	0.9	0.9	2.1	7.6	9.8	0.9	3.4	14.2	19.1	1.3	1.5	16.5	2.0	10.3	3.9	2.3	2.9	0.3	0.3
DvsF	1.9	1.9	2.9	10.2	3.0	17.5	1.3	8.5	0.7	3.7	1.2	5.6	4.1	16.0	2.8	0.5	14.9	1.6	1.6
DvsG	1.4	1.4	2.9	10.3	3.1	3.7	12.6	8.7	0.5	2.0	3.2	5.6	3.1	25.7	1.5	1.6	10.6	1.1	1.1
EvsF	0.9	1.0	4.3	3.0	10.5	16.3	1.5	3.8	14.7	4.3	2.2	8.2	2.1	6.0	5.6	2.3	10.9	1.1	1.1
EvsG	0.3	0.3	3.9	1.7	10.3	3.6	12.4	5.0	15.1	2.6	3.6	9.1	0.7	10.3	4.3	3.1	10.5	1.1	1.1
FvsG	0.9	0.9	0.6	2.0	0.5	19.3	16.2	1.6	0.4	2.6	2.0	1.1	2.1	7.7	2.0	1.1	32.1	3.4	3.4
FvsH	0.6	0.6	0.4	1.2	0.3	10.9	10.0	1.0	0.2	1.6	1.2	0.7	1.3	4.8	1.3	0.7	19.8	6.0	36.5
GvsH	0.1	0.1	0.1	0.3	0.1	2.5	2.1	0.2	·	0.3	0.3	0.2	0.3	1.0	0.3	0.1	4.2	7.6	80.3
Indirect estimates																			
AvsB	7.0	34.8	2.5	4.4	2.7	23.0	9.8	1.3	0.4	1.4	0.1	1.0	0.7	0.9	1.2	0.1	7.8	0.8	0.8
AvsC	5.7	26.7	6.3	1.6	3.5	10.5	0.9	9.4	9.0	4.7	3.1	2.3	1.3	3.2	2.4	0.8	7.2	0.7	0.8
AvsE	5.6	26.1	3.0	1.2	7.7	10.6	1.3	3.9	10.3	2.9	1.6	6.9	1.2	2.9	4.0	1.7	6.8	0.7	0.7
AvsG	7.1	34.9	·	0.1	0.1	10.4	10.7	0.2	0.1	1.2	1.5	·	0.8	7.5	1.0	0.8	19.4	2.0	2.1
AvsH	5.3	26.3	·	0.2	0.1	7.0	7.8	0.2	0.1	0.8	1.1	0.1	0.5	5.3	0.6	0.5	10.1	4.3	27.3
BvsH	·	·	2.3	3.9	2.4	10.2	18.0	1.0	0.5	0.3	1.5	0.8	·	5.8	0.3	0.8	9.1	4.6	35.3
CvsH	0.3	0.3	6.1	1.7	3.5	3.2	8.0	8.9	8.6	3.7	4.0	2.1	0.7	8.8	1.7	1.3	6.1	3.5	27.8
DvsH	1.0	1.0	2.0	7.0	2.1	3.5	7.5	5.8	0.4	1.5	2.0	3.8	2.1	16.7	1.1	1.0	5.3	3.9	32.5
EvsH	0.3	0.3	2.9	1.3	7.5	3.4	8.8	3.5	10.0	2.0	2.5	6.6	0.6	7.8	3.2	2.2	6.2	3.4	27.0
**Entire network**	2.4	9.7	3.9	4.1	4.6	10.1	7.7	6.1	7.8	2.7	2.3	4.3	1.4	8.1	2.3	1.2	9.4	2.0	10.6
**Included studies**	1	1	7	2	3	4	6	3	5	1	2	5	1	3	1	2	8	1	3

© 2023 Yan L et al. *JAMA Network Open.*

Outcome : Pain score change

Direct comparisons in the network

Network meta-analysis estimates	AvsB	AvsC	AvsD	AvsE	AvsF	BvsC	BvsF	CvsD	DvsE	DvsF	EvsF	EvsG	FvsG
Mixed estimates													
AvsB	42.4	10.5	2.4	3.3	6.1	16.2	9.2	2.7	0.6	0.3	2.3	0.4	0.4
AvsC	10.5	34.6	3.8	3.6	5.9	16.6	1.2	10.2	3.8	4.6	0.2	·	·
AvsD	7.6	10.2	9.5	8.2	12.8	3.3	4.4	10.4	10.2	10.2	2.4	0.5	0.5
AvsE	6.5	6.7	5.2	12.8	14.8	0.8	5.7	7.5	9.7	3.0	19.8	3.7	3.7
AvsF	8.5	7.7	5.7	10.5	20.9	0.2	8.8	8.0	3.9	9.7	12.1	2.3	2.3
BvsC	22.7	20.5	1.5	0.6	0.3	28.3	6.7	9.0	3.1	4.5	2.1	0.4	0.4
BvsF	20.8	2.4	3.1	6.4	10.2	10.7	12.7	8.8	3.6	7.8	8.4	1.6	1.6
CvsD	3.8	16.1	6.5	5.4	8.1	9.1	5.3	24.0	8.1	10.6	2.3	0.4	0.4
DvsE	0.7	4.3	4.3	5.9	3.4	2.6	1.9	6.9	23.8	18.8	20.8	3.8	3.8
DvsF	0.3	4.7	4.9	1.6	7.5	3.5	3.8	8.1	17.0	26.8	15.7	3.0	3.0
EvsF	1.3	0.1	0.5	5.9	5.1	0.9	2.2	1.0	9.8	8.4	47.1	8.9	8.9
EvsG	0.8	0.1	0.3	3.7	3.2	0.6	1.4	0.6	6.2	5.3	29.8	8.2	39.8
FvsG	0.1	·	·	0.4	0.4	0.1	0.2	0.1	0.7	0.6	3.3	4.4	89.8
Indirect estimates													
AvsG	5.8	5.2	3.9	7.8	10.1	0.2	5.6	5.4	2.9	6.4	7.0	3.2	33.0
BvsD	19.4	1.1	6.5	4.9	6.9	12.6	9.2	13.7	9.0	12.1	3.4	0.6	0.6
BvsE	17.8	2.0	2.8	8.5	8.5	9.5	9.6	7.5	8.1	2.2	17.1	3.2	3.2
BvsG	10.6	1.7	2.2	4.8	9.4	7.7	9.1	6.0	2.8	5.4	5.0	2.6	28.8
CvsE	3.3	14.9	1.9	8.0	8.8	8.6	5.2	13.1	9.9	5.1	15.7	3.0	3.0
CvsF	2.9	16.8	1.9	5.7	12.2	10.1	7.2	14.2	5.5	10.5	9.4	1.8	1.8
CvsG	2.2	12.4	1.4	4.3	8.9	7.4	5.2	10.4	4.3	7.5	5.9	2.7	27.5
DvsG	0.2	3.1	3.3	1.3	5.0	2.3	2.5	5.4	10.7	17.7	9.2	3.8	34.4
**Entire network**	9.1	8.8	3.4	5.6	8.8	7.1	5.9	8.9	7.8	8.7	10.9	2.7	10.4
**Included studies**	3	1	2	3	3	3	1	1	1	1	3	1	3

© 2023 Yan L et al. *JAMA Network Open.*

Outcome : Hospitalization time

Direct comparisons in the network (columns); **Network meta-analysis estimates** (rows)

	AvsD	AvsF	BvsC	BvsD	BvsE	BvsF	BvsG	CvsD	CvsE	DvsE	FvsG	FvsH	GvsH
**Mixed estimates**													
AvsD	10.9	22.5	16.1	2.7	3.7	22.2	0.3	14.8	1.3	5.0	0.2	0.1	0.1
AvsF	4.1	83.7	2.9	0.5	0.7	4.1	0.1	2.7	0.2	0.9	·	·	·
BvsC	1.0	1.0	93.6	0.3	0.8	1.0	·	1.6	0.4	0.3	·	·	·
BvsD	10.5	10.5	24.1	4.1	5.5	10.4	0.1	22.1	2.0	7.4	0.1	0.1	0.1
BvsE	5.7	5.7	19.6	2.0	23.0	5.7	0.1	10.7	8.9	18.4	·	·	·
BvsF	2.3	2.3	1.6	0.3	0.4	88.3	1.1	1.5	0.1	0.5	0.7	0.4	0.4
BvsG	·	·	·	·	·	1.5	96.2	·	·	·	0.9	0.6	0.6
CvsD	10.7	10.7	21.2	4.1	5.4	10.5	0.1	24.1	2.3	7.7	0.1	0.1	0.1
CvsE	4.8	4.8	27.2	1.7	20.7	4.7	0.1	10.6	8.4	17.0	·	·	·
DvsE	7.5	7.5	7.6	2.6	10.7	7.4	0.1	10.6	7.0	28.0	0.1	·	·
FvsG	1.2	1.2	0.9	0.1	0.2	46.1	47.3	0.8	0.1	0.3	0.8	0.5	0.5
FvsH	0.2	0.2	0.1	·	·	6.5	6.7	0.1	·	·	0.1	79.2	6.8
GvsH	0.8	0.8	0.6	0.1	0.1	30.4	31.2	0.5	·	0.2	0.5	31.7	3.1
**Indirect estimates**													
AvsB	3.4	44.6	2.5	0.4	0.6	44.0	0.6	2.3	0.2	0.8	0.3	0.2	0.2
AvsC	2.8	31.1	31.4	0.2	0.1	30.7	0.4	2.2	0.3	0.4	0.2	0.2	0.2
AvsE	5.5	24.2	10.0	0.9	13.2	23.9	0.3	4.9	5.1	10.4	0.2	0.1	0.1
AvsG	2.3	30.4	1.7	0.3	0.4	29.5	31.9	1.5	0.1	0.5	0.5	0.4	0.4
AvsH	2.2	43.2	1.6	0.3	0.4	1.3	3.6	1.5	0.1	0.5	·	41.8	3.6
BvsH	1.1	1.1	0.8	0.1	0.2	42.8	4.3	0.7	0.1	0.2	0.3	44.2	4.1
CvsF	1.7	1.7	46.7	·	0.2	46.3	0.6	1.6	0.3	0.1	0.4	0.2	0.2
CvsG	0.5	0.5	47.8	0.2	0.4	0.3	48.1	0.8	0.2	0.2	0.5	0.3	0.3
CvsH	1.1	1.1	31.6	·	0.1	28.8	3.0	1.0	0.2	0.1	0.2	30.1	2.8
DvsF	10.4	10.4	19.2	3.2	4.4	26.4	0.3	17.6	1.6	5.9	0.2	0.1	0.1
DvsG	8.0	8.0	16.7	2.8	3.8	7.4	30.7	16.3	1.4	5.2	0.4	0.2	0.2
DvsH	7.6	7.6	14.0	2.4	3.2	17.2	2.4	12.9	1.1	4.3	0.1	24.9	2.2
EvsF	5.0	5.0	13.5	1.3	16.4	30.8	0.4	7.1	6.4	13.5	0.2	0.2	0.2
EvsG	3.8	3.8	13.1	1.3	15.4	3.3	33.1	7.1	6.0	12.3	0.4	0.2	0.2
EvsH	3.6	3.6	9.9	1.0	12.1	20.7	2.4	5.3	4.7	9.9	0.1	24.4	2.2
**Entire network**	4.7	12.8	16.2	1.3	5.8	21.4	10.2	7.2	2.3	5.8	0.3	9.9	1.0
**Included studies**	1	2	4	1	3	3	6	1	3	2	6	1	3

© 2023 Yan L et al. *JAMA Network Open.*

Outcome : Operation time

Direct comparisons in the network

Network meta-analysis estimates	AvsD	AvsF	BvsC	BvsD	BvsE	BvsF	BvsG	CvsD	CvsE	CvsF	CvsG	DvsE	DvsF	DvsG	EvsF	EvsG	FvsG	FvsH	GvsH
**Mixed estimates**																			
AvsD	2.5	31.5	5.3	2.4	0.6	10.4	3.1	5.0	1.4	1.0	0.1	4.1	8.7	10.6	1.5	0.5	8.8	0.6	0.6
AvsF	4.8	84.4	0.9	0.4	0.1	2.0	0.5	0.7	0.2	0.2	·	0.6	1.4	1.8	0.3	0.1	1.4	0.2	0.1
BvsC	0.2	0.2	48.5	1.8	1.4	7.4	7.9	8.8	6.0	2.8	1.0	2.3	2.9	6.2	1.2	1.0	0.3	·	·
BvsD	0.6	0.6	10.8	4.8	1.5	10.8	16.4	9.4	3.4	0.1	0.1	6.6	9.0	17.9	1.0	0.7	2.0	0.1	0.1
BvsE	0.3	0.3	16.1	2.5	4.3	10.0	10.2	2.3	10.4	0.5	0.1	17.1	4.0	8.1	4.5	3.6	0.7	0.1	·
BvsF	0.4	0.4	6.7	1.7	0.8	41.4	16.2	2.7	1.7	2.1	0.2	0.8	5.7	0.9	1.7	0.1	14.6	1.0	1.0
BvsG	0.1	0.1	6.9	2.0	0.9	15.5	39.1	3.7	2.0	0.5	0.7	1.7	1.5	9.0	·	1.2	15.4	0.9	0.9
CvsD	0.5	0.4	21.7	3.3	0.5	7.8	10.5	15.4	7.5	1.9	0.8	8.1	6.7	16.2	0.1	·	1.8	0.1	0.1
CvsE	0.2	0.2	17.7	1.4	3.8	5.7	6.7	9.0	20.6	1.5	0.6	17.5	2.3	4.6	4.2	3.3	0.6	0.1	·
CvsF	0.4	0.4	27.9	0.1	0.4	22.9	5.6	7.7	5.2	3.2	0.8	2.1	5.7	3.5	1.9	0.7	10.0	0.7	0.7
CvsG	0.1	0.1	27.6	0.2	0.4	5.8	22.0	8.4	5.4	2.2	1.2	2.7	0.9	10.4	0.8	1.5	9.2	0.6	0.6
DvsE	0.4	0.3	5.7	2.4	3.8	2.2	4.9	8.2	14.7	0.5	0.3	30.1	5.5	10.7	4.6	3.9	1.5	0.1	0.1
DvsF	0.9	0.9	7.9	3.5	1.0	17.0	4.6	7.4	2.1	1.5	0.1	6.0	15.0	17.1	2.2	0.7	12.4	0.9	0.8
DvsG	0.6	0.6	8.9	3.7	1.0	1.4	15.0	7.7	2.2	0.5	0.6	6.2	9.1	28.7	1.1	1.8	9.4	0.6	0.6
EvsF	0.5	0.6	10.0	1.3	3.6	16.4	0.5	0.5	12.6	1.7	0.3	16.7	7.8	7.1	5.3	3.5	9.7	0.7	0.6
EvsG	0.2	0.2	10.2	1.1	3.7	0.6	16.3	0.2	12.9	0.8	0.6	18.0	2.9	14.1	4.4	4.4	8.4	0.5	0.6
FvsG	0.4	0.5	0.4	0.4	0.1	23.2	22.4	1.0	0.3	1.4	0.5	0.9	6.6	9.4	1.6	1.1	26.2	1.8	1.8
FvsH	0.1	0.2	0.2	0.1	·	4.2	3.8	0.2	0.1	0.3	0.1	0.1	1.2	1.6	0.3	0.2	4.6	72.8	10.2
GvsH	0.3	0.3	0.2	0.2	·	10.9	10.5	0.6	0.2	0.9	0.3	0.5	4.0	5.7	0.9	0.6	15.8	35.7	6.4
**Indirect estimates**																			
AvsB	2.4	37.8	4.5	1.2	0.5	24.2	10.0	2.0	1.2	1.2	0.1	0.7	2.8	1.3	0.9	·	8.2	0.5	0.6
AvsC	2.0	29.7	19.6	0.1	0.3	15.6	4.2	5.7	3.8	2.2	0.6	1.7	3.6	1.9	1.3	0.5	6.6	0.4	0.4
AvsE	2.0	28.5	7.8	0.8	2.6	10.4	0.2	0.1	9.2	1.2	0.2	12.5	4.9	4.6	3.8	2.5	6.7	0.5	0.4
AvsG	2.4	36.7	0.2	0.1	·	14.1	14.2	0.3	0.1	0.8	0.3	0.3	3.6	6.8	0.9	0.7	16.2	1.1	1.1
AvsH	2.5	43.3	0.4	0.1	·	1.3	1.9	0.3	0.1	0.1	0.1	0.2	0.1	1.8	·	0.1	1.8	40.2	5.7
BvsH	0.2	0.2	4.3	1.1	0.5	23.9	12.1	1.8	1.1	1.2	0.2	0.6	3.0	0.2	0.9	0.1	6.9	36.0	5.8
CvsH	0.2	0.2	19.8	·	0.3	14.7	5.4	5.5	3.7	2.2	0.6	1.5	3.6	3.1	1.3	0.6	5.3	27.4	4.4
DvsH	0.6	0.6	5.6	2.5	0.7	10.5	1.8	5.3	1.5	1.0	0.1	4.3	8.9	18.1	1.5	0.6	7.1	29.6	4.8
EvsH	0.3	0.3	8.0	0.9	2.6	10.5	1.0	0.3	9.2	1.2	0.2	12.3	4.9	5.8	3.8	2.6	5.5	26.2	4.2
**Entire network**	0.9	10.7	10.0	1.4	1.3	12.6	8.8	4.2	5.3	1.3	0.4	6.5	4.9	7.7	2.0	1.3	7.6	10.0	1.9
**Included studies**	1	2	6	2	2	2	5	3	5	1	3	3	1	2	2	2	6	1	3

© 2023 Yan L et al. *JAMA Network Open.*

Outcome : Blood loss

Direct comparisons in the network

Network meta-analysis estimates	AvsD	AvsF	BvsC	BvsD	BvsE	BvsG	CvsD	CvsE	CvsG	DvsE	DvsF	DvsG	EvsF	EvsG	FvsG	FvsH	GvsH
Mixed estimates																	
AvsD	7.6	18.5	9.8	2.9	0.1	7.8	19.2	0.5	1.9	10.3	4.1	1.5	7.8	0.3	5.8	0.2	0.4
AvsF	6.4	30.3	5.0	0.4	2.6	8.9	1.2	0.4	1.1	2.5	1.4	0.2	16.3	0.8	16.0	9.1	0.4
BvsC	0.7	1.0	43.2	1.2	4.6	16.2	8.4	1.9	7.5	3.9	1.2	0.6	·	1.4	5.6	1.8	0.6
BvsD	2.4	1.0	16.4	3.1	7.5	16.4	19.0	0.3	2.5	10.5	3.6	1.6	1.9	2.6	8.4	1.9	0.8
BvsE	·	0.5	4.4	0.5	20.6	24.8	2.6	1.0	0.3	3.1	0.2	0.2	10.4	8.1	16.3	1.4	1.0
BvsG	0.1	0.3	2.4	0.2	3.9	82.0	0.5	0.1	1.2	0.2	0.2	0.1	2.5	1.6	3.9	0.4	0.3
CvsD	2.6	0.5	16.8	3.0	5.6	7.4	31.4	1.2	3.2	10.4	3.6	1.6	2.4	2.2	6.0	0.8	0.5
CvsE	0.4	1.0	23.8	0.3	16.8	9.6	7.4	2.1	4.5	5.0	0.9	0.2	16.3	5.7	8.2	2.3	0.4
CvsG	0.5	0.9	31.3	0.8	1.4	33.9	6.6	1.5	6.3	3.1	1.0	0.5	1.4	2.0	6.4	1.6	0.6
DvsE	2.5	1.5	12.1	2.8	10.4	4.4	17.5	1.3	2.3	16.7	3.9	1.5	16.5	4.4	4.0	3.2	-
DvsF	2.5	2.4	10.8	2.5	1.5	10.0	16.2	0.6	2.2	10.3	4.1	1.4	16.5	0.2	16.9	5.3	0.6
DvsG	2.3	1.0	16.0	2.8	5.1	21.8	17.2	0.4	2.8	10.6	3.4	1.5	2.8	3.2	9.4	1.9	0.9
EvsF	0.3	1.7	·	0.1	12.5	10.2	0.8	0.8	0.2	2.9	1.0	0.1	40.8	6.0	16.5	4.0	0.9
EvsG	·	0.3	3.0	0.4	16.4	23.2	2.3	1.0	1.0	3.0	·	0.3	16.9	9.1	16.6	1.2	1.2
FvsG	0.2	2.0	3.6	0.4	9.8	16.5	1.9	0.4	1.0	0.8	1.0	0.3	16.6	5.0	34.4	5.0	2.2
FvsH	·	1.9	2.0	0.2	1.6	2.9	0.5	0.2	0.4	1.1	0.6	0.1	6.5	0.6	8.6	67.2	5.6
GvsH	0.2	0.5	3.8	0.4	6.6	10.2	1.7	0.2	1.0	·	0.4	0.3	8.5	3.5	21.7	32.3	4.7
Indirect estimates																	
AvsB	4.8	19.7	7.9	0.7	8.6	26.4	2.8	0.1	0.9	0.9	·	0.3	5.1	2.7	16.5	2.0	1.4
AvsC	4.9	17.8	23.5	0.2	4.7	16.1	8.6	1.5	4.5	1.9	0.8	0.1	4.8	1.5	10.5	0.5	0.9
AvsE	5.9	24.7	4.4	0.3	10.3	3.0	0.3	1.1	0.7	4.9	0.2	0.1	27.3	6.4	1.8	4.1	0.5
AvsG	5.1	21.8	7.1	0.7	6.9	21.8	2.6	0.1	1.7	1.1	0.2	0.4	4.0	3.9	19.1	2.3	1.7
AvsH	5.3	23.9	2.8	0.2	1.1	5.3	0.7	0.2	0.6	1.3	0.7	0.1	6.6	0.2	7.4	39.3	4.2
BvsF	0.2	1.7	4.2	0.4	10.0	31.0	1.8	0.4	0.1	0.8	0.9	0.1	16.9	3.1	25.5	4.3	1.6
BvsH	0.2	0.5	4.4	0.4	7.5	24.3	1.7	0.2	0.3	0.1	0.4	0.2	8.5	2.3	17.1	27.9	3.9
CvsF	0.6	2.0	22.4	0.4	5.4	16.9	6.5	1.5	4.4	3.0	1.4	0.2	10.4	1.7	17.7	4.6	0.9
CvsH	0.5	1.0	19.1	0.3	4.1	12.9	6.0	1.2	3.7	2.2	1.0	0.2	7.5	1.3	12.2	23.7	3.2
DvsH	2.2	1.3	8.9	2.2	0.6	8.6	16.3	0.5	1.8	9.7	3.4	1.2	9.4	0.1	8.8	24.1	2.9
EvsH	0.2	0.2	1.1	·	10.2	6.6	0.3	0.7	0.1	2.8	0.4	·	26.5	4.8	7.8	34.9	3.8
**Entire network**	2.0	5.8	10.5	1.1	7.1	16.4	7.7	0.8	2.2	4.8	1.5	0.6	10.7	3.0	12.4	10.8	1.7
**Included studies**	1	1	5	2	2	3	1	3	1	2	1	2	1	1	7	1	3

© 2023 Yan L et al. *JAMA Network Open.*

Outcome : quality of life score change

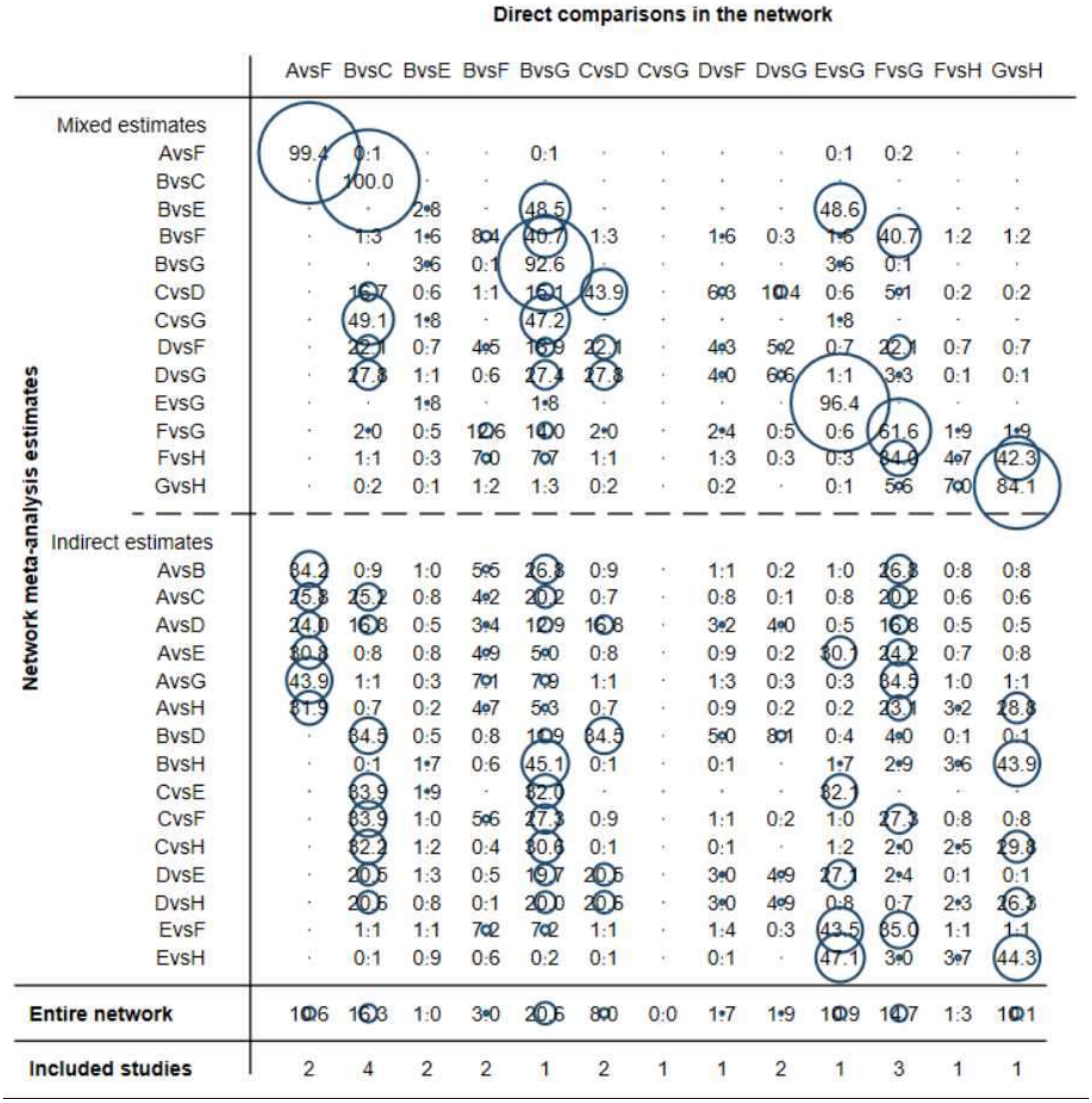

© 2023 Yan L et al. *JAMA Network Open.*

Outcome : Cup Abduction angle

Network meta-analysis estimates	AvsD	BvsC	BvsD	BvsF	BvsG	CvsD	CvsE	CvsG	DvsE	DvsF	DvsG	FvsG	FvsH	GvsH
	**Direct comparisons in the network**													
**Mixed estimates**														
AvsD	99.3	·	0.2	0.1	·	·	·	·	0.1	·	0.1	·	·	·
BvsC	·	10.8	18.4	8.2	4.3	3.8	10.0	16.2	10.0	0.2	3.9	6.7	1.3	1.3
BvsD	·	8.9	39.9	8.1	4.0	2.7	7.9	1.8	7.9	0.8	9.5	6.1	1.2	1.2
BvsF	·	4.6	9.4	45.6	3.3	0.7	1.9	7.1	1.9	1.1	5.7	10.5	2.6	2.6
BvsG	·	9.8	18.3	12.8	6.8	1.5	4.4	16.3	4.4	0.1	12.5	10.6	2.0	2.0
CvsD	·	8.1	12.0	2.5	1.5	6.5	18.6	16.5	18.5	0.4	12.1	2.4	0.5	0.5
CvsE	·	3.9	5.8	1.2	0.7	3.1	55.0	7.9	14.8	0.2	5.8	1.2	0.2	0.2
CvsG	·	6.9	1.6	5.5	3.0	3.3	9.5	42.9	9.5	0.2	10.1	4.7	0.9	0.9
DvsE	·	5.8	8.7	1.8	1.1	4.7	22.5	10.9	31.9	0.3	8.8	1.8	0.3	0.3
DvsF	·	3.0	20.9	24.5	0.6	2.3	6.6	5.9	6.6	1.3	10.2	10.0	2.5	2.5
DvsG	·	2.6	10.3	6.8	3.8	3.8	10.0	17.5	10.0	0.6	20.9	6.2	1.2	1.2
FvsG	·	6.1	10.5	22.0	4.4	1.0	3.0	10.1	3.0	1.0	8.4	21.8	4.1	4.1
FvsH	·	2.0	3.8	7.2	1.4	0.3	1.0	3.3	1.0	0.3	2.7	6.9	55.7	10.4
GvsH	·	3.9	7.5	14.3	2.9	0.7	2.0	6.6	2.0	0.6	5.5	13.8	28.7	10.7
**Indirect estimates**														
AvsB	37.8	5.5	24.8	5.1	2.5	1.7	4.9	1.1	4.9	0.5	5.9	3.8	0.8	0.7
AvsC	33.1	5.4	8.0	1.7	1.0	4.3	12.4	10.1	12.4	0.3	8.1	1.6	0.3	0.3
AvsE	35.2	3.8	5.6	1.2	0.7	3.0	14.6	7.8	20.7	0.2	5.7	1.2	0.2	0.2
AvsF	29.2	2.1	14.8	17.4	0.4	1.6	4.7	4.2	4.7	0.9	7.2	9.2	1.8	1.8
AvsG	33.1	1.8	8.9	4.6	2.6	2.6	7.4	10.7	7.4	0.4	14.0	4.2	0.8	0.8
AvsH	24.2	1.1	10.0	10.9	0.2	1.5	4.2	4.6	4.2	0.6	6.9	5.3	17.8	6.5
BvsE	·	10.2	19.9	6.7	3.4	1.7	21.8	10.0	18.4	0.3	0.2	5.3	1.0	1.0
BvsH	·	4.0	8.0	24.7	2.9	0.6	1.8	6.4	1.8	0.5	5.1	4.6	29.8	9.8
CvsF	·	9.1	10.1	20.9	1.7	2.9	8.3	18.3	8.3	0.9	0.1	14.0	2.7	2.7
CvsH	·	6.5	6.5	16.7	0.7	2.2	6.3	16.3	6.3	0.6	1.3	8.3	22.6	8.6
DvsH	·	1.4	14.5	15.7	0.2	1.9	5.6	6.1	5.6	0.8	9.2	6.9	23.5	8.5
EvsF	·	5.8	10.1	18.0	1.1	1.0	18.4	10.7	10.8	0.8	2.8	10.2	2.2	2.2
EvsG	·	2.4	4.7	4.5	2.5	0.4	27.6	24.9	15.9	0.3	10.3	4.0	0.8	0.8
EvsH	·	4.1	7.8	12.2	0.4	0.7	15.7	10.9	10.0	0.6	3.3	6.6	19.4	7.3
**Entire network**	10.3	4.9	10.8	10.7	1.9	2.1	10.6	10.7	9.2	0.6	7.0	7.1	8.6	3.6
**Included studies**	1	3	1	4	3	2	3	1	2	2	2	5	1	2

© 2023 Yan L et al. *JAMA Network Open.*

Outcome : Short-term hip score

Direct comparisons in the network

Network meta-analysis estimates

	AvsD	AvsF	BvsC	BvsD	BvsE	BvsF	BvsG	CvsD	CvsE	CvsG	DvsE	DvsF	DvsG	FvsG	FvsH	GvsH
Mixed estimates																
AvsD	83.1	21.2	1.8	8.1	2.8	10.9	1.1	2.6	0.4	1.1	3.2	4.8	2.6	1.8	0.8	0.8
AvsF	23.6	25.4	2.1	9.0	3.2	16.5	1.2	2.8	0.4	1.2	3.6	5.3	2.9	2.0	0.9	0.9
BvsC	1.4	1.5	19.4	4.6	2.6	16.0	7.2	6.8	2.3	20.2	0.2	2.2	1.7	7.9	3.4	3.4
BvsD	6.2	6.2	4.5	18.0	6.3	19.9	3.1	5.5	0.8	1.8	7.2	9.5	5.5	2.9	1.3	1.3
BvsE	3.4	3.4	4.0	9.9	20.8	10.8	2.2	2.3	4.2	2.6	23.7	5.2	2.8	2.2	1.0	0.9
BvsF	0.7	0.7	1.0	1.4	0.5	88.2	1.4	0.1	·	0.9	0.5	1.1	0.1	1.7	0.7	0.7
BvsG	1.0	1.0	8.4	3.7	1.7	23.0	12.8	2.2	0.9	10.5	0.8	1.5	4.8	10.3	6.2	6.2
CvsD	4.0	3.9	10.7	10.1	3.0	3.3	3.8	10.8	2.8	16.7	5.9	6.1	6.2	4.6	2.0	2.0
CvsE	1.6	1.6	12.2	4.3	14.2	2.4	3.9	7.3	5.2	10.9	19.1	2.4	3.6	4.5	1.9	2.0
CvsG	0.6	0.6	10.9	1.2	1.1	9.5	6.8	5.8	1.8	39.2	0.7	0.9	3.8	7.6	3.3	3.3
DvsE	3.0	3.0	0.3	8.8	18.5	8.6	0.8	3.5	4.4	1.2	38.8	4.6	3.0	0.7	0.3	0.3
DvsF	6.0	6.0	3.6	16.6	5.4	26.7	2.1	4.9	0.8	2.1	6.2	9.2	5.0	3.5	1.5	1.5
DvsG	4.4	4.3	3.6	12.0	3.9	3.5	8.7	6.6	1.5	10.7	5.3	6.7	8.9	10.1	4.4	4.4
FvsG	1.4	1.4	7.7	2.9	1.4	24.0	10.9	2.3	0.8	10.9	0.5	2.1	4.7	16.0	6.5	6.5
FvsH	1.0	1.0	5.6	2.1	1.0	17.5	8.7	1.7	0.6	7.9	0.4	1.5	3.4	10.9	5.7	30.9
GvsH	0.1	0.1	0.4	0.1	0.1	1.2	0.6	0.1	·	0.6	·	0.1	0.2	0.8	2.2	93.4
Indirect estimates																
AvsB	20.8	21.6	2.3	8.4	3.0	27.1	1.7	2.5	0.4	0.6	3.3	4.1	2.5	1.0	0.4	0.4
AvsC	17.1	16.8	10.4	3.4	0.6	10.9	3.5	6.6	1.9	16.2	2.4	1.6	3.1	4.6	2.0	2.0
AvsE	22.0	17.3	1.2	0.3	13.8	16.5	0.3	0.4	3.1	1.5	22.4	0.5	·	0.9	0.4	0.4
AvsG	18.1	17.4	4.1	4.4	1.3	6.2	7.8	3.7	0.9	8.7	2.2	2.3	5.5	9.4	4.1	4.1
AvsH	14.3	13.8	3.1	3.5	1.0	4.6	6.0	2.9	0.7	6.8	1.8	1.8	4.3	7.2	3.9	24.3
BvsH	0.7	0.7	6.0	2.7	1.2	17.3	9.2	1.6	0.6	8.2	0.6	1.1	3.4	10.2	5.4	31.0
CvsF	1.6	1.6	16.1	3.3	2.0	26.8	5.5	5.9	2.0	17.6	·	2.4	1.4	7.5	3.2	3.2
CvsH	0.4	0.4	9.2	0.9	0.8	6.6	4.1	3.8	1.2	25.2	0.4	0.6	2.3	4.6	3.0	36.3
DvsH	3.3	3.2	2.5	8.9	2.9	3.0	6.3	4.9	1.1	8.5	4.0	5.0	6.5	7.3	4.0	28.6
EvsF	3.1	3.1	2.8	7.4	16.2	27.6	1.2	1.8	3.4	2.5	19.3	4.7	2.3	2.5	1.1	1.1
EvsG	1.8	1.8	3.1	4.7	13.8	7.8	7.5	3.3	3.7	10.2	18.0	2.7	5.5	8.6	3.7	3.8
EvsH	1.4	1.4	2.3	3.7	10.8	6.5	5.7	2.6	2.9	7.8	14.1	2.2	4.2	6.5	3.5	24.3
**Entire network**	7.2	6.7	6.0	5.8	5.6	14.1	5.1	3.9	1.8	9.8	7.4	3.2	3.8	6.1	2.9	10.2
**Included studies**	1	1	3	1	3	4	2	1	2	1	1	1	1	3	1	2

© 2023 Yan L et al. *JAMA Network Open.*

Outcome : Long-term hip score

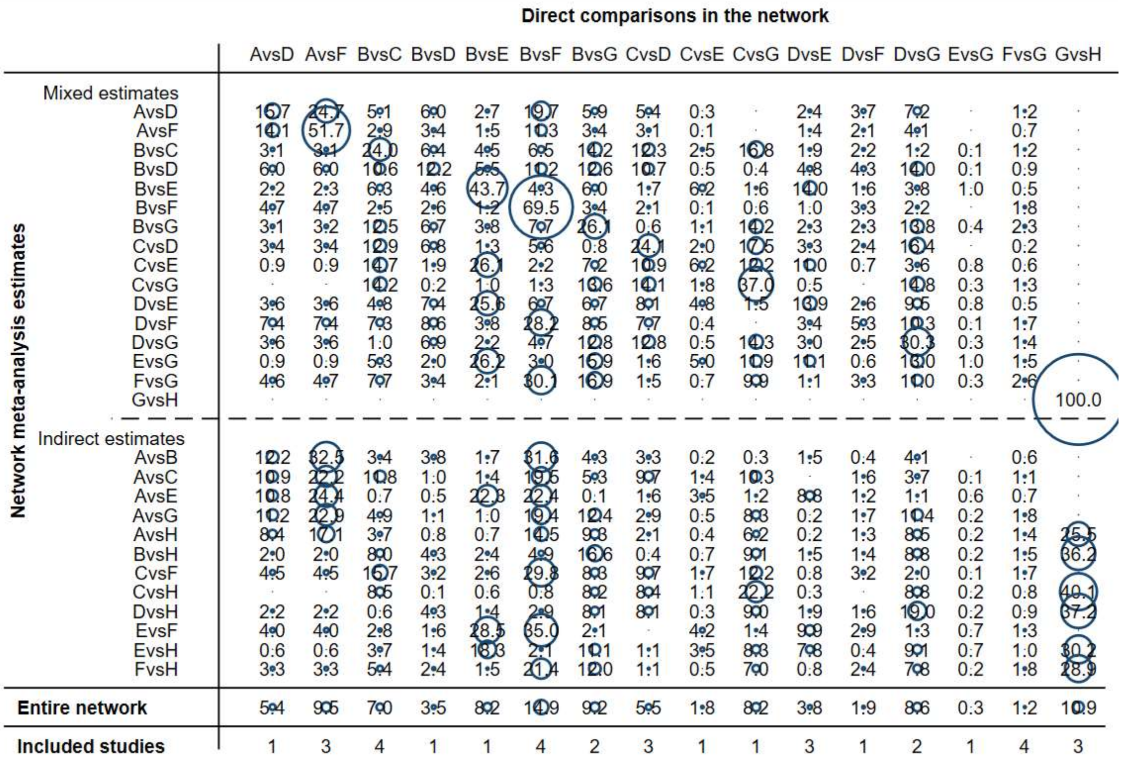

© 2023 Yan L et al. *JAMA Network Open.*

Outcome : Dislocation

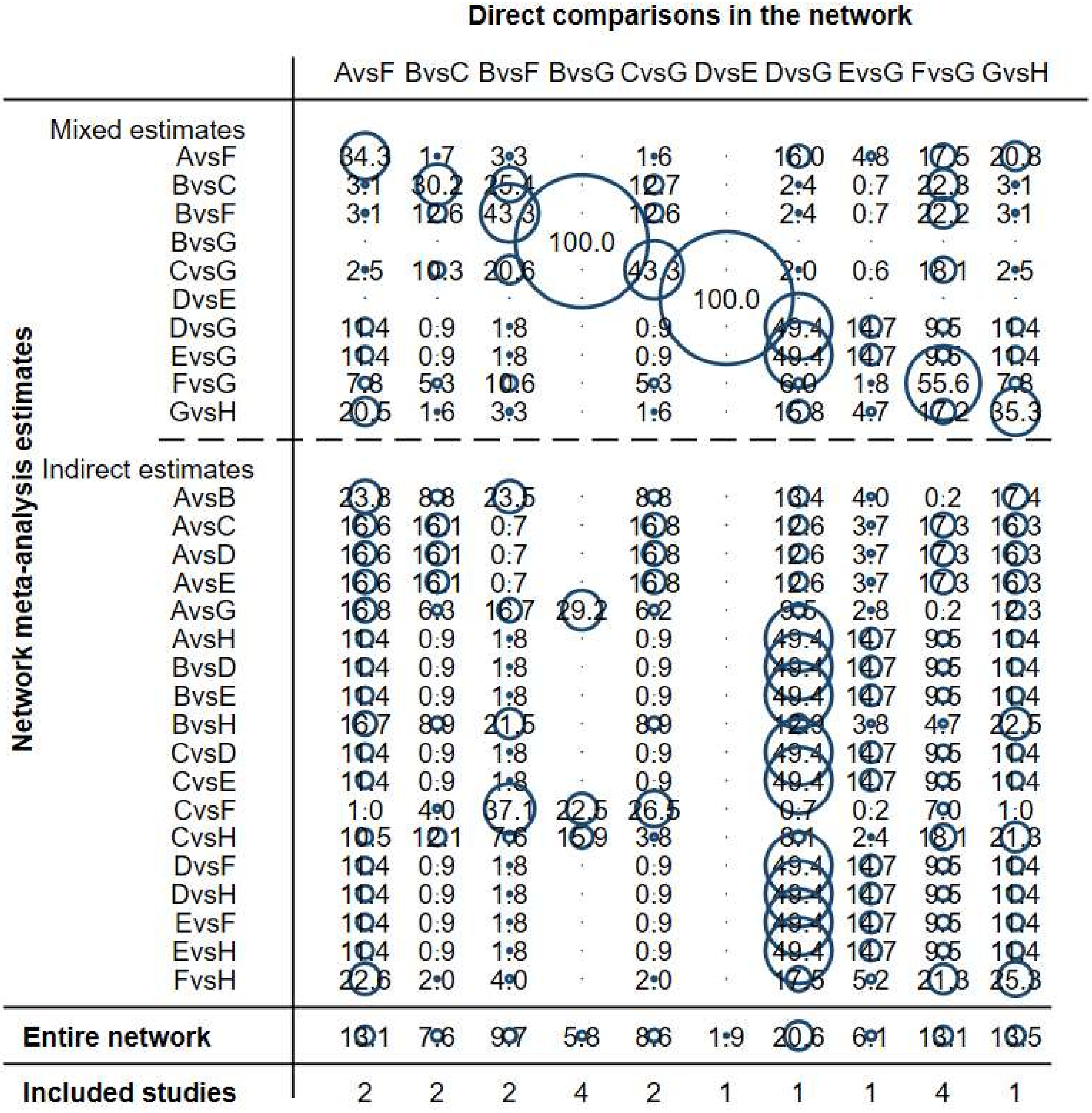

© 2023 Yan L et al. *JAMA Network Open.*

Outcome : Fracture

Direct comparisons in the network

Network meta-analysis estimates	AvsE	AvsF	BvsC	BvsD	BvsE	BvsF	BvsG	CvsD	CvsG	DvsE	DvsF	DvsG	EvsG	FvsG
Mixed estimates														
AvsE	10.4	26.5	1.0	0.1	13.0	9.7	2.4	0.4	1.4	8.2	6.8	0.9	5.4	10.0
AvsF	12.4	60.0	0.5	0.1	6.1	4.5	1.1	0.2	0.6	3.8	3.2	0.4	2.5	4.7
BvsC	0.4	0.4	35.1	2.7	4.6	7.0	10.7	6.2	19.8	2.4	1.7	0.6	1.8	5.7
BvsD	0.2	0.2	8.9	9.6	10.9	12.1	10.3	7.3	1.7	12.0	14.9	8.2	0.9	3.0
BvsE	6.2	6.1	5.4	3.9	29.9	8.6	8.3	2.5	2.9	10.7	3.4	2.0	8.3	1.0
BvsF	3.9	3.9	7.0	3.6	7.3	25.0	12.3	1.8	5.2	2.4	8.2	0.4	1.0	18.1
BvsG	1.0	1.0	12.1	3.2	7.3	12.8	24.6	0.1	12.0	1.6	0.9	4.1	4.6	14.6
CvsD	0.4	0.4	18.0	6.3	6.1	5.3	0.3	10.8	16.0	8.6	10.6	7.5	2.1	6.7
CvsG	0.6	0.6	21.9	0.6	2.7	5.7	12.8	6.0	30.8	0.7	2.5	3.4	2.8	8.9
DvsE	5.5	5.5	4.0	6.0	16.7	4.1	2.7	5.0	1.0	22.7	10.6	6.4	6.8	2.1
DvsF	3.9	3.9	2.4	6.4	4.2	10.8	1.2	5.7	3.3	9.9	23.1	8.0	1.9	14.4
DvsG	1.0	1.0	1.5	6.8	4.6	1.0	10.8	7.1	8.6	10.4	15.4	10.6	4.8	15.4
EvsG	5.8	5.8	4.8	0.7	18.7	2.7	10.9	1.9	6.7	10.7	3.5	4.7	10.2	10.9
FvsG	3.3	3.3	4.7	0.7	0.7	14.8	10.6	1.9	6.5	1.0	8.2	4.6	3.6	35.2
Indirect estimates														
AvsB	9.6	30.0	4.8	2.6	8.5	15.5	8.2	1.4	3.4	0.4	4.1	0.5	0.7	10.4
AvsC	7.7	25.0	14.9	0.7	4.6	9.0	0.5	4.4	13.4	1.6	4.3	0.7	1.5	10.7
AvsD	9.9	30.4	2.1	4.8	0.2	6.4	0.3	4.2	2.1	9.7	15.7	5.9	·	8.3
AvsG	8.9	30.7	3.2	0.5	3.7	7.1	8.1	1.3	4.6	1.4	3.5	3.2	3.7	20.1
CvsE	4.8	4.8	19.8	1.0	19.0	1.6	1.8	6.0	15.6	10.3	1.4	1.8	7.4	4.6
CvsF	2.9	2.9	20.3	1.0	2.6	15.4	1.4	6.1	19.0	0.2	8.0	0.7	0.5	19.1
EvsF	8.2	8.2	1.4	0.2	18.3	13.7	3.4	0.5	1.9	10.5	9.5	1.3	7.6	14.2
**Entire network**	5.6	12.6	9.1	2.8	9.0	9.1	6.4	3.9	8.3	6.7	7.7	3.6	3.7	10.4
**Included studies**	1	4	3	1	2	2	3	1	2	2	2	1	1	4

© 2023 Yan L et al. *JAMA Network Open.*

Outcome : Infection

Direct comparisons in the network

Network meta-analysis estimates	AvsE	AvsF	BvsC	BvsF	BvsG	CvsD	CvsG	DvsE	EvsF	FvsG
Mixed estimates										
AvsE	25.4	21.8	5.5	2.3	3.2	9.3	3.7	9.3	12.5	6.9
AvsF	16.5	43.6	4.2	1.8	2.4	7.0	2.8	7.0	9.4	5.3
BvsC	3.0	3.0	39.6	5.4	14.3	7.8	12.0	7.7	4.8	2.3
BvsF	2.5	2.5	10.7	12.8	23.0	6.4	4.3	6.4	4.0	27.3
BvsG	1.6	1.6	13.2	10.7	38.0	4.1	9.1	4.1	2.5	14.9
CvsD	4.1	4.1	6.3	2.6	3.7	50.1	4.2	10.5	6.5	7.9
CvsG	3.4	3.5	20.6	3.7	16.9	9.0	15.8	9.0	5.5	12.7
DvsE	4.9	4.9	7.6	3.2	4.4	12.8	5.1	39.6	7.8	9.5
EvsF	10.4	10.4	8.1	3.4	4.7	13.5	5.5	13.6	18.2	10.2
FvsG	2.0	2.0	1.3	7.5	8.7	5.3	4.0	5.3	3.2	60.7
Indirect estimates										
AvsB	10.5	21.5	9.6	7.9	14.5	8.2	1.4	8.2	2.3	16.0
AvsC	10.7	19.3	10.4	4.8	6.6	12.0	7.7	12.0	0.3	14.3
AvsD	14.1	17.6	8.4	3.5	4.8	14.0	5.6	17.8	3.7	10.5
AvsG	10.4	26.4	3.3	2.7	6.0	7.1	3.8	7.1	4.3	27.9
BvsD	4.3	4.3	20.7	5.0	10.1	25.5	4.9	10.2	6.9	6.2
BvsE	6.9	6.9	13.7	6.4	12.7	14.8	1.1	14.8	10.0	10.5
CvsE	5.9	5.9	9.2	3.8	5.3	21.4	6.2	21.4	9.4	10.5
CvsF	3.9	4.0	16.6	7.0	9.6	10.2	10.2	10.2	6.3	20.8
DvsF	5.8	5.8	10.4	4.8	6.6	19.1	7.7	15.2	9.8	14.3
DvsG	4.9	4.9	12.1	1.3	10.7	21.7	9.6	12.8	7.9	14.1
EvsG	8.3	8.3	7.2	0.8	8.0	13.5	6.3	13.5	13.3	20.8
**Entire network**	8.0	10.9	10.4	4.7	9.9	14.1	6.2	12.6	7.1	15.0
**Included studies**	1	1	3	1	2	2	1	2	1	4

© 2023 Yan L et al. *JAMA Network Open.*

Outcome : Nerve injury

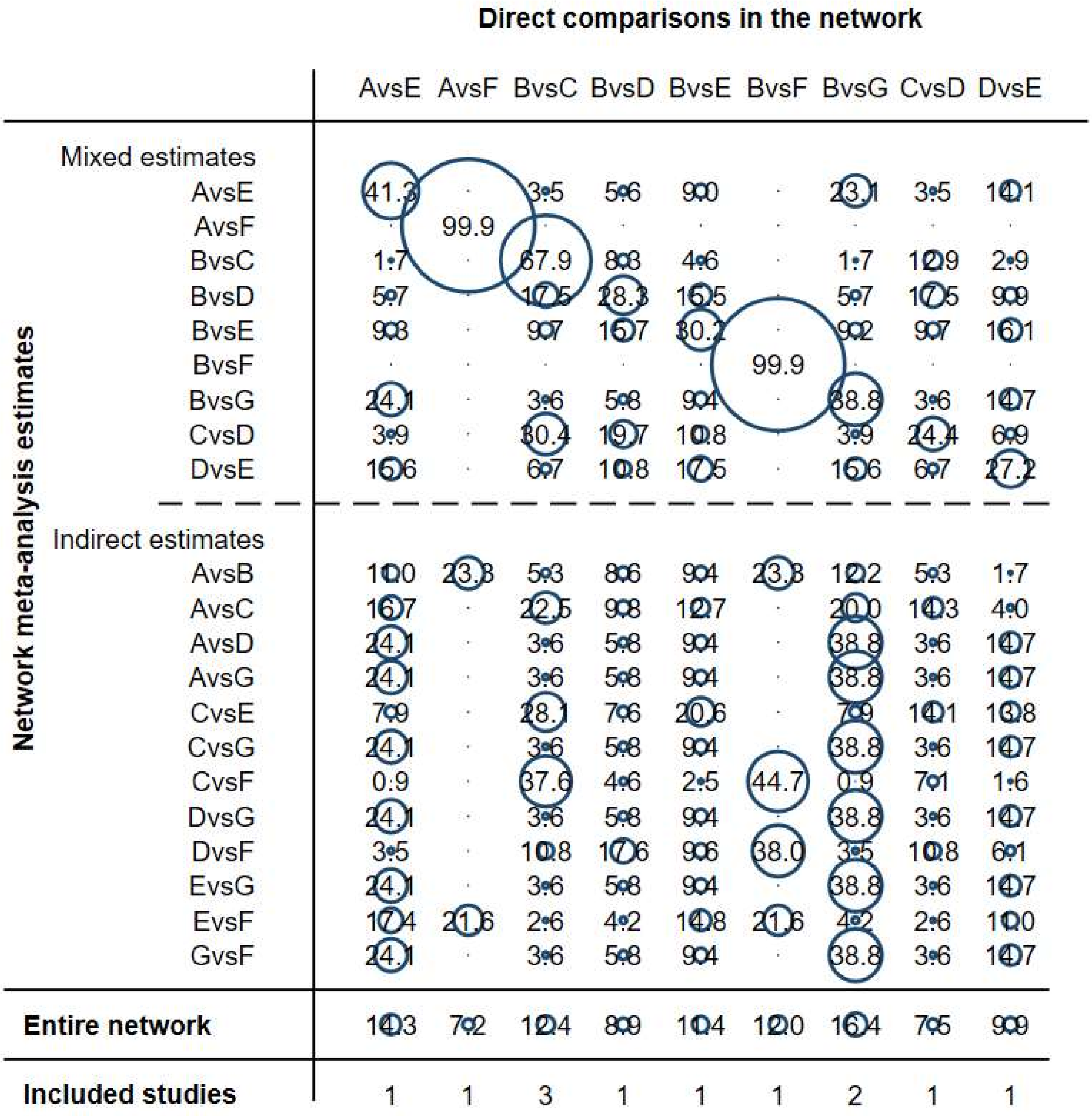

© 2023 Yan L et al. *JAMA Network Open.*

Outcome : Reoperation

Direct comparisons in the network

Network meta-analysis estimates	AvsF	BvsC	BvsG	CvsD	CvsG	DvsE	DvsF	DvsG	EvsG	FvsG
Mixed estimates										
AvsF	100.0	·	·	·	·	·	·	·	·	·
BvsC	·	53.6	1.4	6.4	9.6	3.6	13.2	2.7	1.9	7.7
BvsG	·	1.3	79.9	4.6	0.5	3.0	2.5	7.6	0.1	0.4
CvsD	·	16.6	12.3	20.3	5.9	10.1	4.2	24.7	1.2	4.7
CvsG	·	20.5	1.2	4.9	18.0	7.2	19.4	2.3	5.3	21.3
DvsE	·	10.1	8.6	10.8	9.3	16.3	18.6	17.1	1.9	7.4
DvsF	·	16.0	3.1	2.0	10.9	8.1	42.8	6.2	2.2	8.7
DvsG	·	5.5	16.7	19.0	2.2	12.4	10.2	33.0	0.4	1.7
EvsG	·	5.7	0.3	1.3	7.4	2.0	5.4	0.6	16.5	61.8
FvsG	·	5.7	0.3	1.3	7.4	2.0	5.4	0.6	16.5	61.8
Indirect estimates										
AvsB	100.0	·	·	·	·	·	·	·	·	·
AvsC	100.0	·	·	·	·	·	·	·	·	·
AvsD	100.0	·	·	·	·	·	·	·	·	·
AvsE	100.0	·	·	·	·	·	·	·	·	·
AvsG	100.0	·	·	·	·	·	·	·	·	·
BvsD	·	2.6	29.0	14.4	3.9	10.9	5.3	15.8	3.4	13.7
BvsE	·	16.0	3.1	2.0	10.9	8.1	42.8	6.2	2.2	8.7
BvsF	·	16.0	3.1	2.0	10.9	8.1	42.8	6.2	2.2	8.7
CvsE	·	25.6	1.4	6.1	15.4	9.0	24.2	2.9	3.1	12.3
CvsF	·	16.0	3.1	2.0	10.9	8.1	42.8	6.2	2.2	8.7
EvsF	·	16.0	3.1	2.0	10.9	8.1	42.8	6.2	2.2	8.7
**Entire network**	19.1	12.4	8.4	5.8	7.5	6.9	17.6	7.9	2.9	10.5
**Included studies**	3	5	3	1	1	1	2	2	1	5

© 2023 Yan L et al. *JAMA Network Open.*

Outcome : Thromboembolism

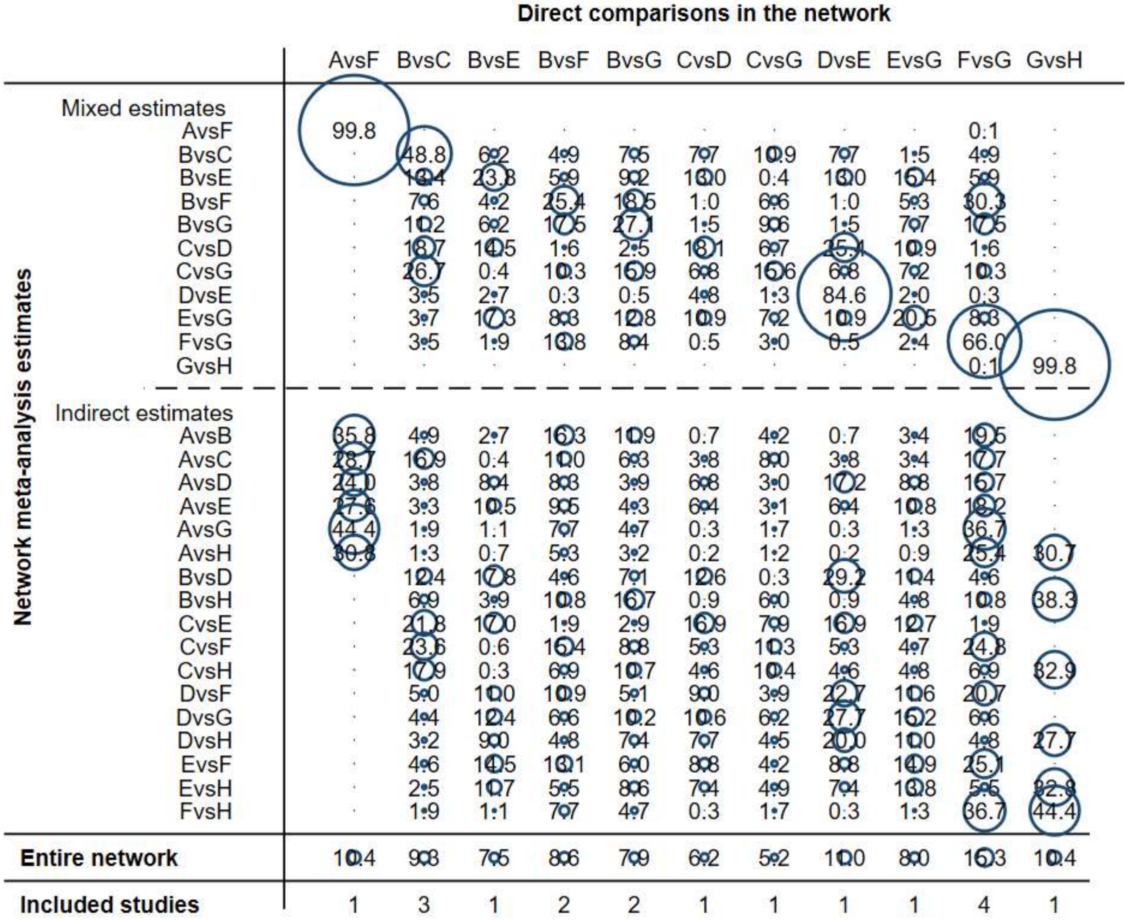

Outcome : Cup Anteversion angle

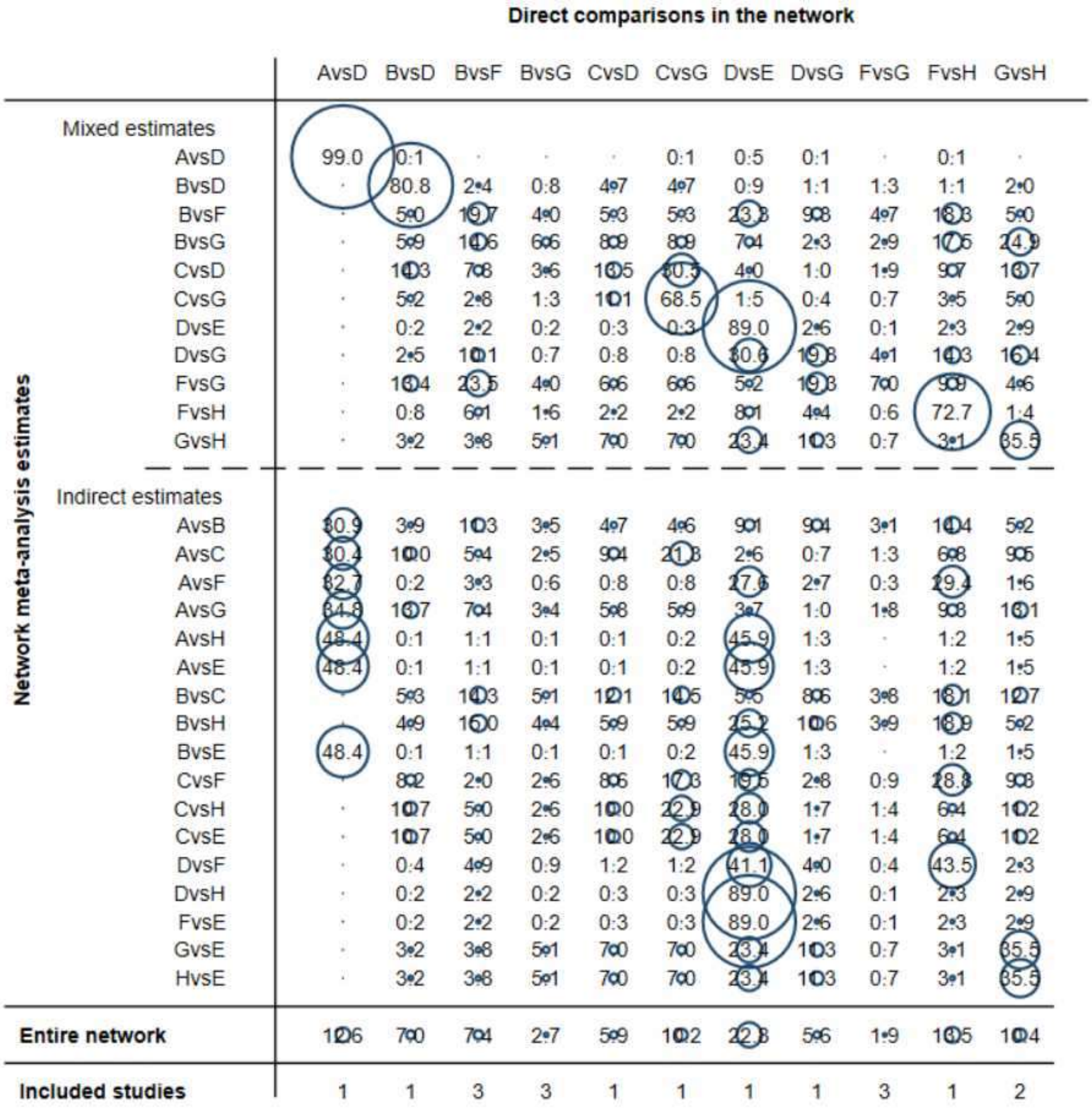

© 2023 Yan L et al. *JAMA Network Open.*

Outcome : Abductor muscle strengths change

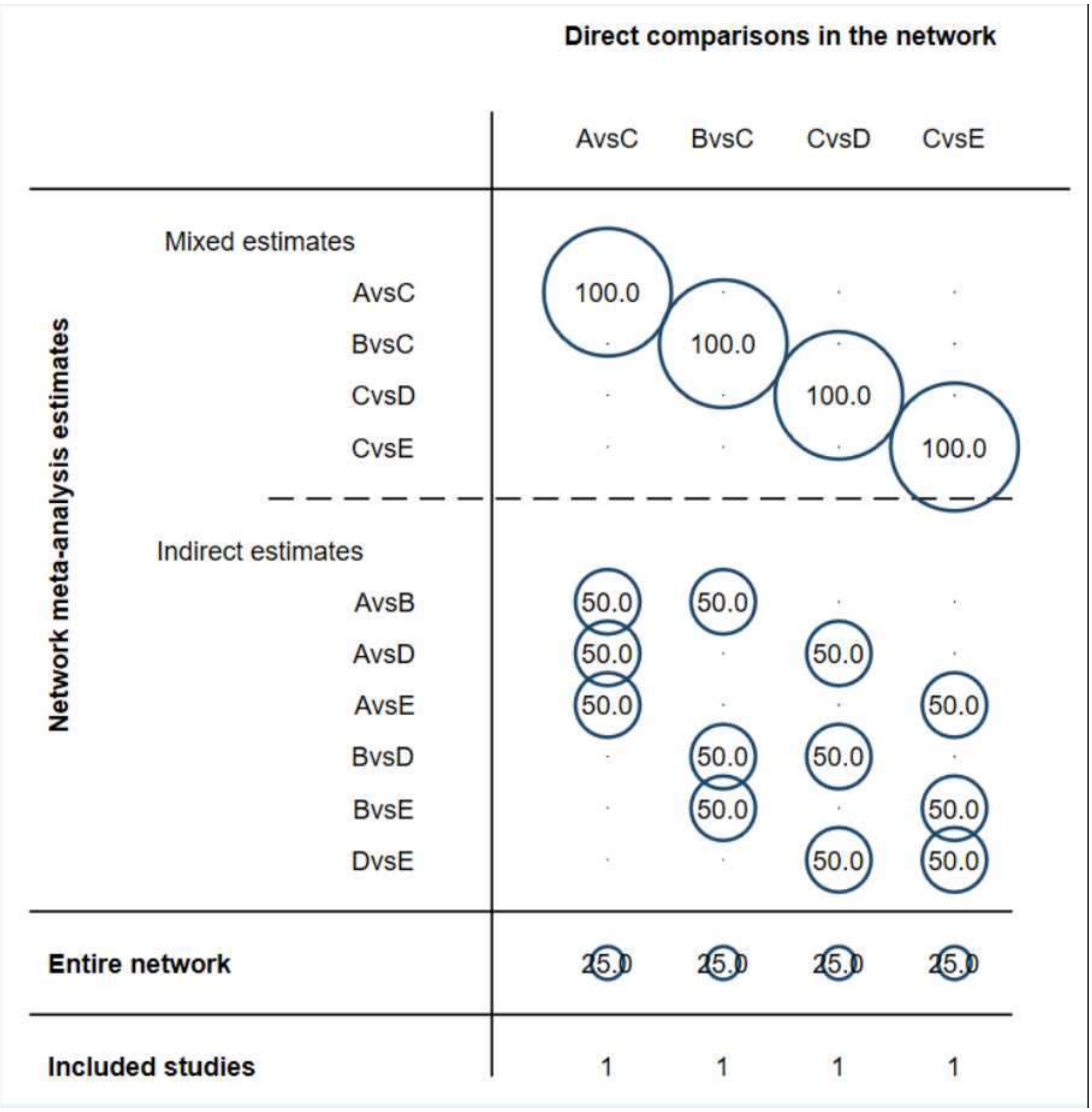

© 2023 Yan L et al. *JAMA Network Open.*

Outcome : Analgesic consumption

Direct comparisons in the network

Network meta-analysis estimates	AvsF	BvsC	BvsE	BvsF	BvsG	CvsD	CvsE	FvsG
Mixed estimates								
AvsF	98.4	0.1	0.1	0.4	·	·	·	0.9
BvsC	2.1	92.1	0.2	3.6	0.5	·	1.1	0.5
BvsE	0.8	0.1	96.8	1.4	0.2	·	0.4	0.2
BvsF	7.0	3.8	3.9	67.3	7.6	·	·	10.3
BvsG	0.8	2.8	2.9	41.8	8.1	0.2	·	43.5
CvsD	48.6	0.1	0.1	3.2	2.6	0.2	·	45.2
CvsE	0.1	4.1	4.2	0.1	·	·	91.5	·
FvsG	8.7	0.3	0.3	6.0	4.6	0.3	·	79.8
Indirect estimates								
AvsB	46.6	2.3	2.3	38.8	4.4	·	·	5.5
AvsC	31.9	33.2	1.6	25.9	2.9	·	0.4	4.0
AvsE	31.9	1.6	33.3	26.3	3.0	·	0.2	3.9
AvsG	48.6	0.1	0.1	3.2	2.6	0.2	·	45.2
AvsD	48.6	0.1	0.1	3.2	2.6	0.2	·	45.2
BvsD	48.6	0.1	0.1	3.2	2.6	0.2	·	45.2
CvsF	4.3	1.2	22.0	38.7	4.4	·	23.4	6.0
CvsG	0.3	1.0	17.2	28.0	5.4	0.1	18.5	29.4
EvsF	4.6	21.7	1.2	38.5	4.3	·	23.5	6.1
EvsG	0.1	17.1	1.1	27.9	5.4	0.1	18.6	29.7
EvsD	48.6	0.1	0.1	3.2	2.6	0.2	·	45.2
FvsD	48.6	0.1	0.1	3.2	2.6	0.2	·	45.2
GvsD	48.6	0.1	0.1	3.2	2.6	0.2	·	45.2
**Entire network**	27.9	8.1	8.3	19.0	3.5	0.1	7.4	25.7
**Included studies**	1	2	1	1	1	1	1	2

© 2023 Yan L et al. *JAMA Network Open.*

Outcome : Cadence change

Direct comparisons in the network

Network meta-analysis estimates	AvsB	AvsF	BvsC	CvsD	EvsF
**Mixed estimates**					
AvsB	98.4	0.5	0.5	0.3	0.3
AvsF	0.1	99.6	0.1	·	0.1
BvsC	0.8	0.6	97.8	0.6	0.3
CvsD	0.5	0.4	0.7	98.2	0.2
EvsF	0.5	0.7	0.4	0.2	98.2
**Indirect estimates**					
AvsC	49.4	0.5	49.4	0.4	0.3
AvsE	0.3	49.6	0.2	0.1	49.7
AvsD	33.0	0.5	33.0	33.2	0.2
BvsE	33.1	33.1	0.3	0.2	33.2
BvsF	49.6	49.7	0.3	0.2	0.2
BvsD	0.7	0.5	49.2	49.4	0.2
CvsE	24.8	24.9	24.9	0.3	25.1
CvsF	33.1	33.3	33.1	0.3	0.2
EvsD	19.9	20.0	19.9	20.1	20.1
FvsD	24.9	25.0	24.8	25.0	0.2
**Entire network**	25.6	22.9	22.7	14.4	14.4
**Included studies**	2	1	1	1	1

© 2023 Yan L et al. *JAMA Network Open.*

Outcome : Creatine kinase change

Direct comparisons in the network

Network meta-analysis estimates	AvsB	AvsF	BvsC	BvsD	CvsD	CvsE	CvsF	EvsF	FvsG
**Mixed estimates**									
AvsB	56.9	12.6	14.6	2.1	5.4	1.8	3.3	2.8	0.6
AvsF	3.1	91.7	1.7	0.2	0.6	0.3	1.0	1.1	0.3
BvsC	4.3	2.0	78.6	5.1	7.3	0.9	1.0	0.5	0.1
BvsD	4.9	2.2	40.6	10.1	41.2	·	0.4	0.5	0.1
CvsD	3.2	1.4	14.5	10.3	69.0	0.9	0.5	0.3	0.1
CvsE	16.9	10.0	29.2	0.1	13.5	14.6	1.7	13.7	0.3
CvsF	26.1	31.8	27.8	1.2	6.4	1.5	2.6	2.1	0.5
EvsF	20.4	34.2	10.8	1.5	3.3	10.9	2.0	15.0	0.9
FvsG	24.8	42.9	13.0	1.9	5.2	1.2	2.4	4.8	4.0
**Indirect estimates**									
AvsC	35.9	9.7	37.3	1.7	8.1	1.8	2.9	2.2	0.5
AvsD	27.7	8.6	23.0	6.6	28.6	1.0	2.1	1.9	0.4
AvsE	25.6	21.8	14.8	1.9	4.2	12.8	1.6	16.5	0.8
AvsG	37.0	17.9	19.4	2.8	7.7	1.8	2.5	5.7	5.3
BvsE	20.2	10.6	26.4	3.6	8.5	14.2	1.0	14.2	0.3
BvsF	35.6	41.5	10.5	1.5	3.9	1.3	2.7	2.4	0.5
BvsG	23.2	5.6	37.8	5.5	14.5	4.1	0.9	3.1	5.2
CvsG	18.9	3.6	40.6	0.4	21.8	5.0	2.0	2.7	5.1
DvsE	12.6	7.7	16.0	6.6	31.9	12.1	1.2	10.6	0.2
DvsF	21.8	27.0	18.1	5.3	23.0	0.9	2.1	2.0	0.4
DvsG	13.4	1.8	21.9	8.9	42.8	3.5	1.3	2.1	4.3
EvsG	8.9	18.5	1.4	0.9	4.6	27.5	0.9	29.2	8.2
**Entire network**	22.1	19.7	23.6	3.8	16.9	4.9	1.8	5.8	1.4
**Included studies**	3	1	1	1	2	1	1	1	2

© 2023 Yan L et al. *JAMA Network Open.*

Outcome : C-reactive protein change

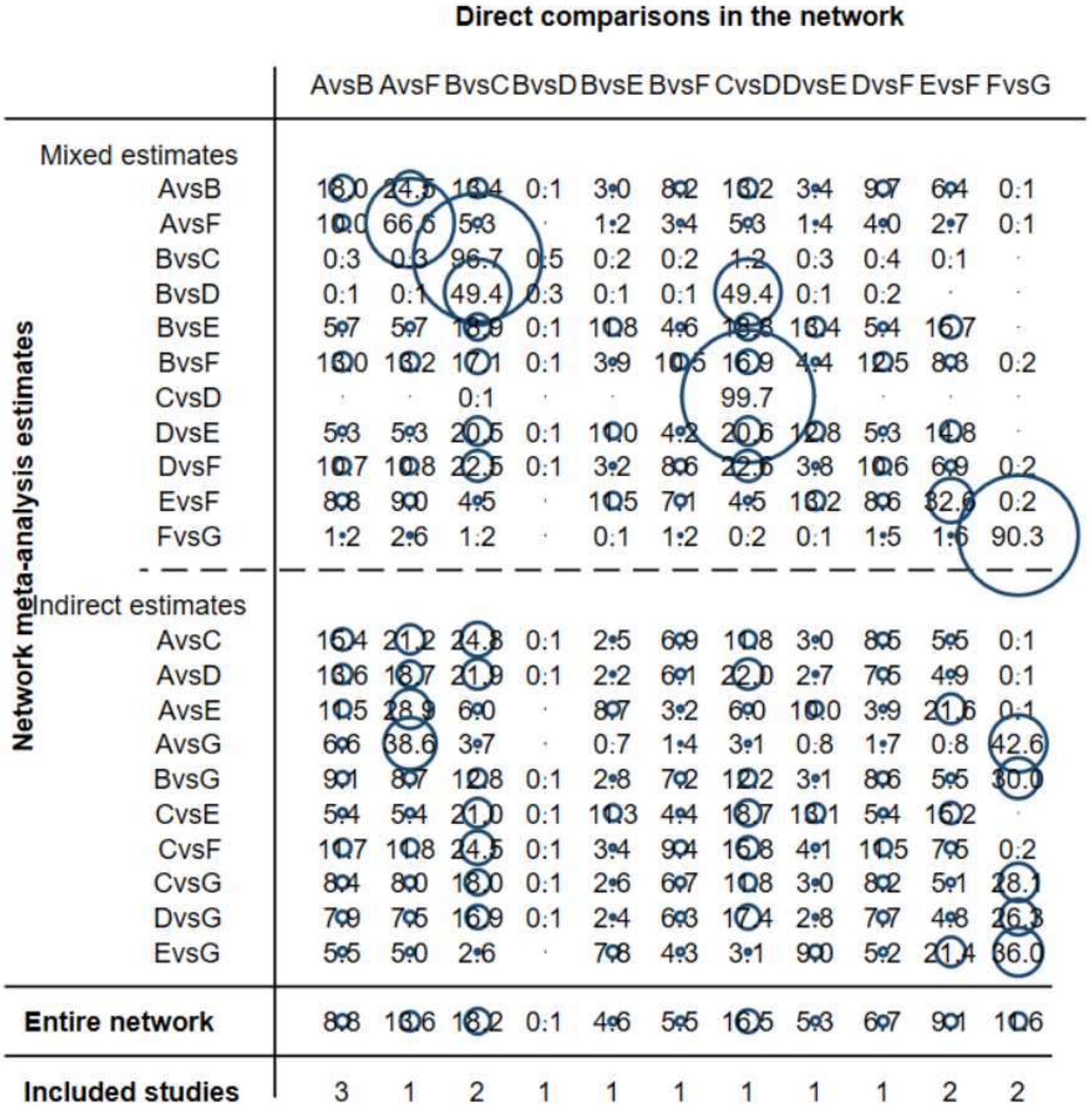

© 2023 Yan L et al. *JAMA Network Open.*

Outcome : Erythrocyte sedimentation rate change

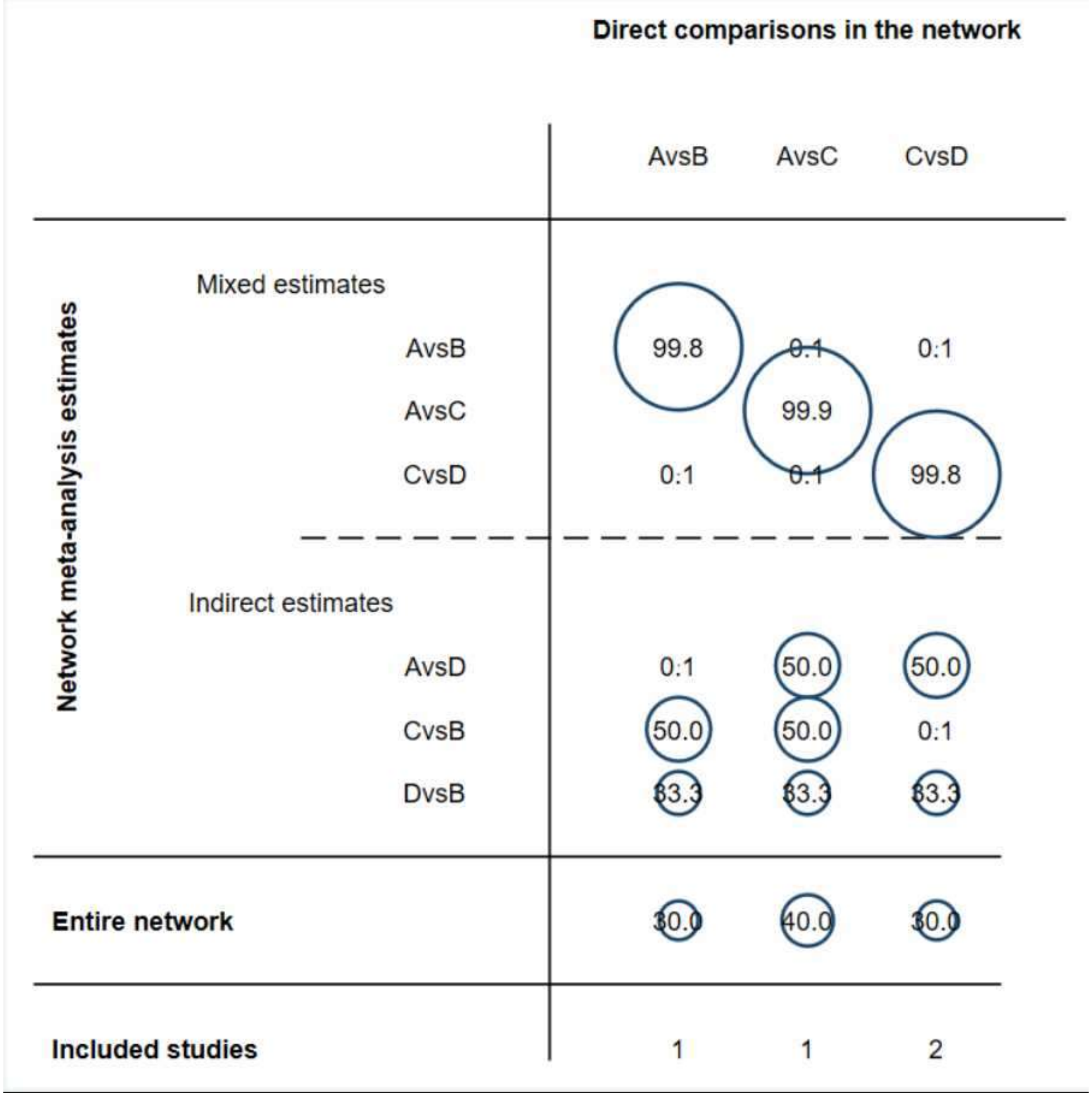

© 2023 Yan L et al. *JAMA Network Open.*

Outcome : Hemoglobin change

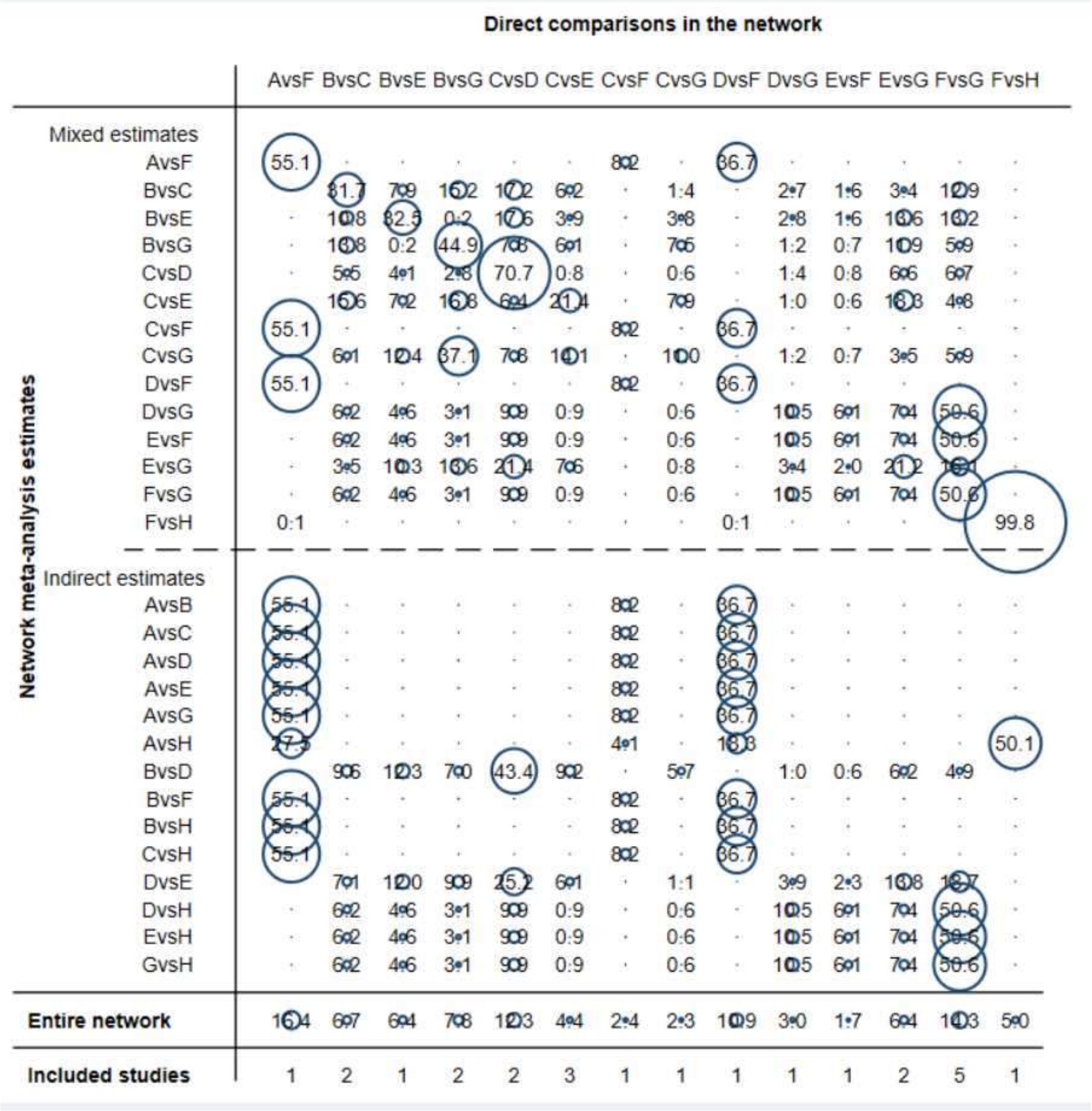

© 2023 Yan L et al. *JAMA Network Open.*

Outcome : Hematocrit change

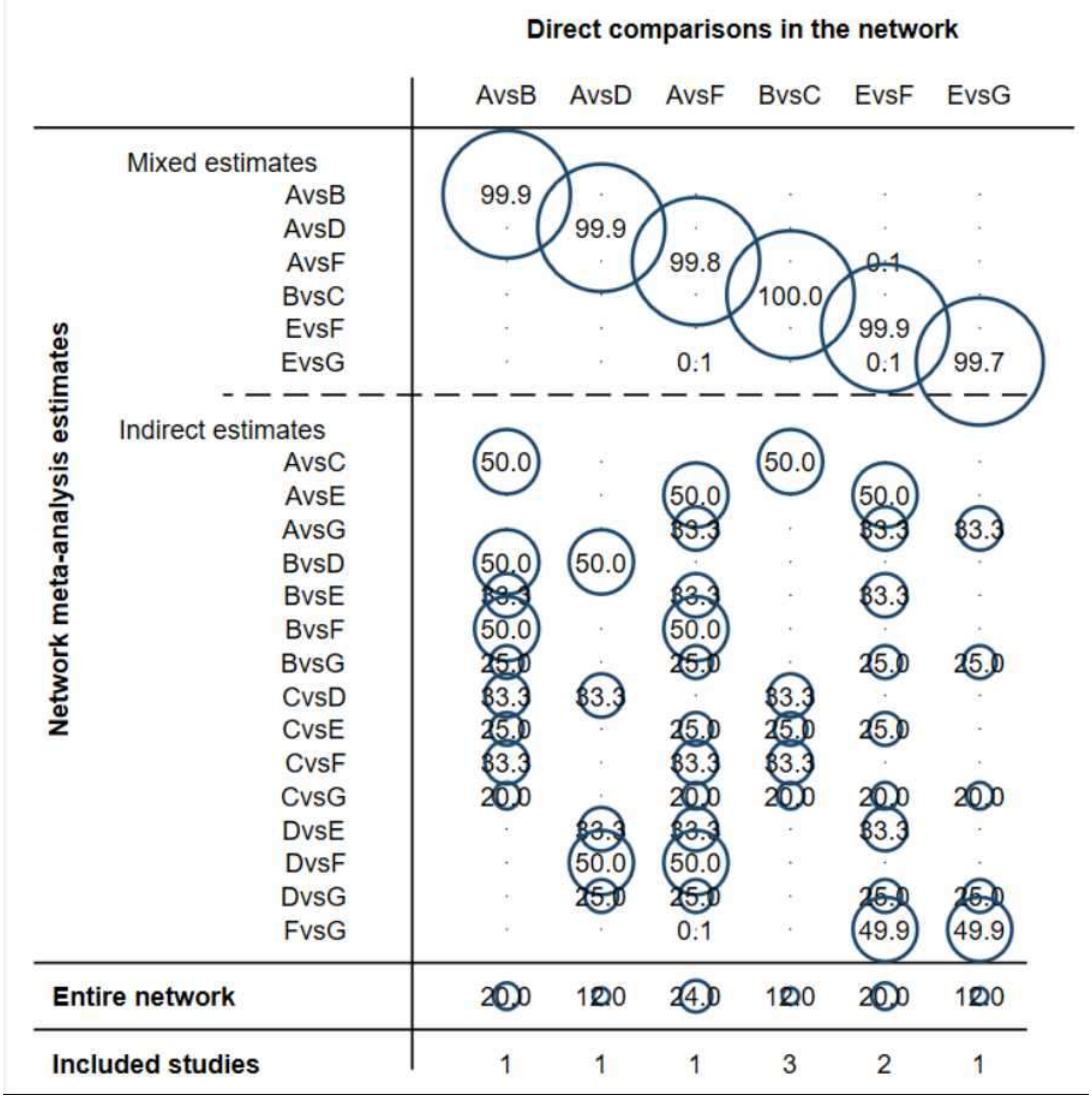

© 2023 Yan L et al. *JAMA Network Open.*

Outcome : Interleukin-6 change

Direct comparisons in the network

Network meta-analysis estimates	AvsB	AvsE	BvsC	BvsD	BvsE	CvsD	CvsE	DvsE
Mixed estimates								
AvsB	44.3	21.1	6.5	5.0	9.7	1.9	4.5	6.9
AvsE	0.5	98.7	0.1	0.1	0.2	·	0.1	0.2
BvsC	20.9	20.4	18.2	2.4	4.7	8.2	19.4	5.8
BvsD	25.2	25.2	3.8	3.0	5.6	1.2	2.6	33.5
BvsE	35.7	35.8	5.4	4.1	8.0	1.6	3.7	5.7
CvsD	9.5	10.1	12.8	1.2	2.1	9.1	21.5	33.7
CvsE	12.5	13.2	16.8	1.4	2.8	10.9	28.1	13.3
DvsE	0.2	0.2	·	0.2	·	0.2	0.1	99.1
Indirect estimates								
AvsC	9.8	33.6	12.9	1.0	2.1	9.1	21.5	10.1
AvsD	0.3	49.6	0.1	0.1	0.1	0.1	·	49.7
**Entire network**	16.9	28.7	8.5	2.0	3.8	4.8	10.3	24.0
**Included studies**	2	1	1	1	1	1	1	2

© 2023 Yan L et al. *JAMA Network Open.*

Outcome : Leg length discrepancy

Direct comparisons in the network

Network meta-analysis estimates	AvsB	AvsE	CvsD	CvsE	DvsE
Mixed estimates					
AvsB	99.9	·	·	·	·
AvsE	·	99.9	·	·	·
CvsD	·	·	40.6	29.7	29.7
CvsE	·	·	28.2	43.7	28.2
DvsE	·	·	16.6	16.6	68.8
Indirect estimates					
AvsC	·	41.8	16.4	25.4	16.4
AvsD	·	45.8	8.5	8.5	37.3
CvsB	29.5	29.5	10.6	17.9	10.6
DvsB	31.4	31.4	5.8	5.8	25.6
EvsB	50.0	50.0	·	·	·
**Entire network**	20.9	31.3	12.1	14.6	21.1
**Included studies**	1	1	2	1	3

© 2023 Yan L et al. *JAMA Network Open.*

Outcome : Myoglobin change

Direct comparisons in the network

Network meta-analysis estimates	AvsB	BvsD	CvsD	CvsE	CvsF	EvsF
**Mixed estimates**						
AvsB	93.5	4.6	1.4	0.3	0.3	·
BvsD	17.9	63.5	13.1	2.4	2.8	0.2
CvsD	10.4	25.3	32.5	14.2	16.6	1.1
CvsE	1.1	2.6	8.1	33.7	25.6	29.0
CvsF	1.1	2.7	8.2	22.0	40.2	25.9
EvsF	·	0.1	0.3	15.7	16.3	67.5
**Indirect estimates**						
AvsC	36.1	18.4	16.5	12.9	16.2	1.0
AvsD	46.2	40.1	9.7	1.8	2.1	0.1
AvsE	33.4	16.4	9.3	14.2	5.7	21.9
AvsF	33.7	16.6	9.5	4.4	16.1	20.7
BvsC	22.8	24.8	19.8	14.7	17.2	1.1
BvsE	21.9	20.0	10.2	16.0	6.6	24.4
BvsF	22.1	20.3	10.5	5.0	18.1	23.0
DvsE	9.8	22.6	19.4	17.0	8.2	23.6
DvsF	9.4	22.9	19.9	6.5	19.4	22.1
**Entire network**	23.5	20.0	13.1	12.1	14.1	17.3
**Included studies**	1	1	1	1	1	1

© 2023 Yan L et al. *JAMA Network Open.*

Outcome : Stem alignment

Direct comparisons in the network

Network meta-analysis estimates	AvsD	BvsC	BvsG	CvsD	CvsE	DvsE	DvsF	DvsG	FvsG
**Mixed estimates**									
AvsD	100.0	·	·	·	·	·	·	·	·
BvsC	·	84.3	4.6	4.2	0.4	0.4	1.6	3.0	1.6
BvsG	·	25.3	12.8	23.0	2.3	2.3	8.9	16.4	8.9
CvsD	·	4.3	4.3	71.3	7.2	7.2	1.5	2.8	1.5
CvsE	·	1.8	1.8	29.1	34.1	30.9	0.6	1.1	0.6
DvsE	·	1.7	1.7	27.6	29.3	37.5	0.6	1.1	0.6
DvsF	·	9.2	9.2	8.4	0.8	0.8	23.0	19.6	28.8
DvsG	·	10.3	10.3	12.1	1.2	1.2	16.3	28.3	16.3
FvsG	·	4.3	4.3	3.9	0.4	0.4	10.6	9.2	63.9
**Indirect estimates**									
AvsB	32.1	28.8	3.3	26.1	2.6	2.6	1.2	2.2	1.2
AvsC	45.3	2.4	2.4	39.0	3.9	3.9	0.8	1.5	0.8
AvsE	40.0	1.0	1.0	16.6	17.6	22.5	0.4	0.6	0.4
AvsF	34.2	6.1	6.1	5.5	0.6	0.6	16.2	12.9	19.0
AvsG	36.3	8.5	8.5	7.7	0.8	0.8	9.8	18.1	9.8
BvsD	·	42.3	4.9	38.5	3.9	3.9	1.7	3.2	1.7
BvsE	·	36.8	3.1	16.0	20.7	19.1	1.1	2.0	1.1
BvsF	·	23.2	9.1	21.1	2.1	2.1	13.2	10.1	19.1
CvsF	·	8.7	8.7	26.5	2.7	2.7	16.1	13.0	21.7
CvsG	·	12.3	12.3	28.0	2.8	2.8	10.8	20.0	10.8
EvsF	·	6.7	6.7	8.4	15.1	18.1	14.4	12.0	18.7
EvsG	·	9.5	9.5	7.4	16.9	19.9	9.6	17.7	9.6
**Entire network**	13.1	15.4	6.5	19.5	7.7	8.4	8.3	10.0	10.2
**Included studies**	1	2	2	1	1	1	2	1	3

© 2023 Yan L et al. *JAMA Network Open.*

Outcome : Step length change

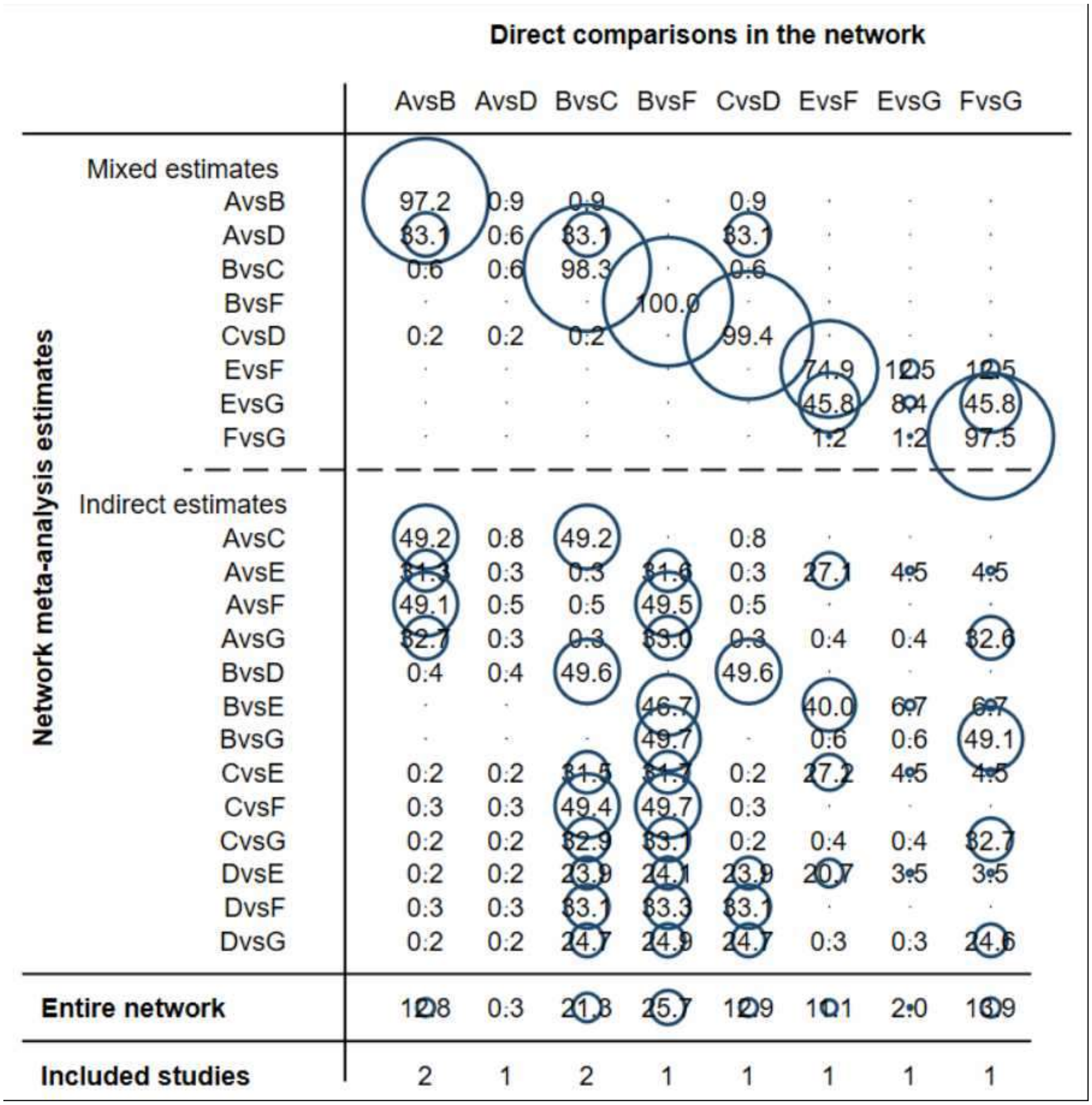

© 2023 Yan L et al. *JAMA Network Open.*

Outcome : Timed Up and Go Test result change

Direct comparisons in the network

Network meta-analysis estimates	AvsB	AvsC	AvsE	DvsE	EvsF
Mixed estimates					
AvsB	100.0	·	·	·	·
AvsC	·	100.0	·	·	·
AvsE	·	·	99.9	·	·
DvsE	0.1	0.1	0.2	99.5	0.1
EvsF	·	·	0.1	·	99.9
Indirect estimates					
AvsD	·	·	49.9	49.9	·
AvsF	·	·	50.0	·	50.0
BvsC	50.0	50.0	·	·	·
BvsD	33.4	·	33.3	33.3	·
BvsE	50.0	·	50.0	·	·
BvsF	33.3	·	33.3	·	33.3
CvsD	·	33.4	33.3	33.3	·
CvsE	·	50.0	50.0	·	·
CvsF	·	33.3	33.3	·	33.3
DvsF	·	·	0.1	49.9	50.0
**Entire network**	17.3	17.3	31.0	17.2	17.2
**Included studies**	2	1	1	2	1

© 2023 Yan L et al. *JAMA Network Open.*

Outcome : Volume of blood transfusion

Direct comparisons in the network

Network meta-analysis estimates	AvsD	AvsF	BvsD	BvsE	BvsF	CvsF	DvsE	DvsF	EvsF
Mixed estimates									
AvsD	29.8	25.3	10.6	2.0	7.3	2.5	6.0	7.9	7.5
AvsF	3.8	87.9	1.4	0.4	1.5	1.0	0.9	1.3	1.9
BvsD	15.9	12.8	38.2	5.9	15.8	0.7	4.5	4.9	1.3
BvsE	8.1	9.4	17.3	9.8	16.2	1.5	6.0	2.6	29.7
BvsF	13.1	17.2	20.8	7.3	21.6	3.6	2.5	4.3	9.6
CvsF	5.8	14.7	1.1	0.9	4.7	61.1	0.7	2.1	9.0
DvsE	18.6	17.7	10.2	4.6	4.4	0.9	8.8	5.9	28.9
DvsF	25.8	27.1	10.8	2.1	7.7	2.9	6.2	8.2	8.2
EvsF	5.3	8.9	0.7	5.2	3.8	2.7	6.6	1.8	65.1
Indirect estimates									
AvsB	13.1	30.4	18.4	6.0	17.7	2.6	1.7	3.0	7.2
AvsC	6.1	45.9	1.6	0.3	2.1	38.7	0.1	0.5	4.6
AvsE	5.4	42.7	1.2	3.5	1.5	1.2	4.6	0.4	39.6
BvsC	9.0	7.6	18.9	6.4	17.6	31.9	2.0	2.8	3.8
CvsD	20.6	17.0	10.5	1.5	4.6	30.6	5.3	6.4	2.6
CvsE		3.4	0.3	4.7	0.3	40.9	5.6	0.1	44.7
**Entire network**	12.8	23.4	10.7	4.2	9.0	13.6	4.3	3.7	17.3
**Included studies**	1	1	3	1	2	1	1	1	4

© 2023 Yan L et al. *JAMA Network Open.*

Outcome : Walking speed change

Direct comparisons in the network

Network meta-analysis estimates	AvsE	AvsF	BvsC	BvsE	BvsG	CvsD	DvsE	EvsF	FvsG	FvsH	GvsH
Mixed estimates											
AvsE	16.1	23.2	·	16.3	16.4	·	·	7.8	8.7	6.7	6.7
AvsF	13.6	50.7	·	9.0	9.0	·	·	4.6	5.1	3.9	3.9
BvsC	·	·	57.8	14.0	·	14.0	14.0	·	·	·	·
BvsE	0.1	0.1	0.3	98.1	0.3	0.3	0.3	0.2	0.2	0.1	0.1
BvsG	2.7	2.7	·	6.4	75.1	·	·	3.7	3.7	2.8	2.8
CvsD	·	·	21.5	21.4	0.1	35.4	21.5	·	·	·	·
DvsE	·	·	22.9	22.8	0.1	22.9	31.1	·	·	·	·
EvsF	7.9	7.9	0.1	21.8	21.8	0.1	0.1	10.8	12.1	9.2	9.2
FvsG	7.4	7.4	0.1	17.5	17.5	0.1	0.1	10.1	16.8	12.0	12.0
FvsH	6.1	6.1	·	14.3	14.4	·	·	8.8	13.0	10.4	27.4
GvsH	0.2	0.2	·	0.5	0.5	·	·	0.3	0.4	0.9	97.0
Indirect estimates											
AvsB	14.8	21.4	0.1	21.8	14.3	0.1	0.1	7.1	8.1	6.2	6.2
AvsC	10.9	15.7	21.4	10.9	10.5	5.2	5.2	5.2	5.9	4.5	4.5
AvsD	10.3	16.4	10.8	0.9	10.9	10.8	15.9	5.5	6.2	4.7	4.7
AvsG	13.0	21.6	0.1	18.2	18.2	0.1	0.1	5.2	9.8	7.1	7.1
AvsH	10.8	18.1	·	15.0	15.1	·	·	4.3	7.6	6.2	22.7
BvsD	·	·	21.8	28.5	0.1	21.8	28.6	·	·	·	·
BvsF	8.1	8.1	0.1	19.1	22.0	0.1	0.1	10.0	12.5	9.5	9.5
BvsH	1.6	1.6	·	3.9	42.4	·	·	2.2	1.9	2.0	44.3
CvsE	·	·	40.0	39.9	0.1	9.9	9.9	0.1	0.1	·	·
CvsF	5.8	5.8	23.4	7.8	16.6	5.7	5.7	7.8	8.9	6.7	6.7
CvsG	1.4	1.4	32.7	4.7	37.4	7.9	7.9	1.9	1.8	1.4	1.4
CvsH	1.1	1.1	23.8	3.3	27.1	5.8	5.8	1.4	1.2	1.3	28.3
DvsF	5.7	5.7	12.2	3.1	16.3	12.2	16.4	7.7	8.7	6.6	6.6
DvsG	1.1	1.1	13.9	16.1	30.0	13.9	18.7	1.5	1.5	1.1	1.1
DvsH	0.9	0.9	10.7	12.2	22.9	10.7	14.4	1.2	1.0	1.1	23.9
EvsG	1.7	1.7	0.1	44.1	44.2	0.1	0.1	2.3	2.2	1.7	1.7
EvsH	1.2	1.2	0.1	30.4	30.5	0.1	0.1	1.7	1.4	1.5	31.9
**Entire network**	5.7	8.8	10.2	16.2	18.7	6.3	7.2	4.4	5.4	4.2	12.3
**Included studies**	1	1	3	1	2	2	1	1	1	1	1

© 2023 Yan L et al. *JAMA Network Open.*

eFigure 5. Intransitivity Assessments

The transitivity is a key assumption for robustness of indirect comparisons that consist of direct comparisons. These direct comparisons require the similar characteristics of trials including similar distributions of either baseline characteristics or the effect of control group. Thus, we compared the distributions of the characteristics across arms grouped by the corresponding interventions. The following figures are violin plots and half box plots that aims to display the distribution of the effect of different arms in the same group (i.e., same approach). The overlaps in the y-axis dimension represent similar distribution of these effects or characteristics. The meaning of symbols in the figure is shown in the figure below.

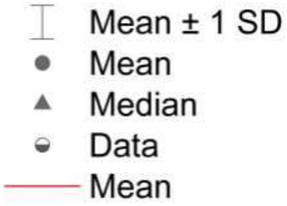

Percentage male.

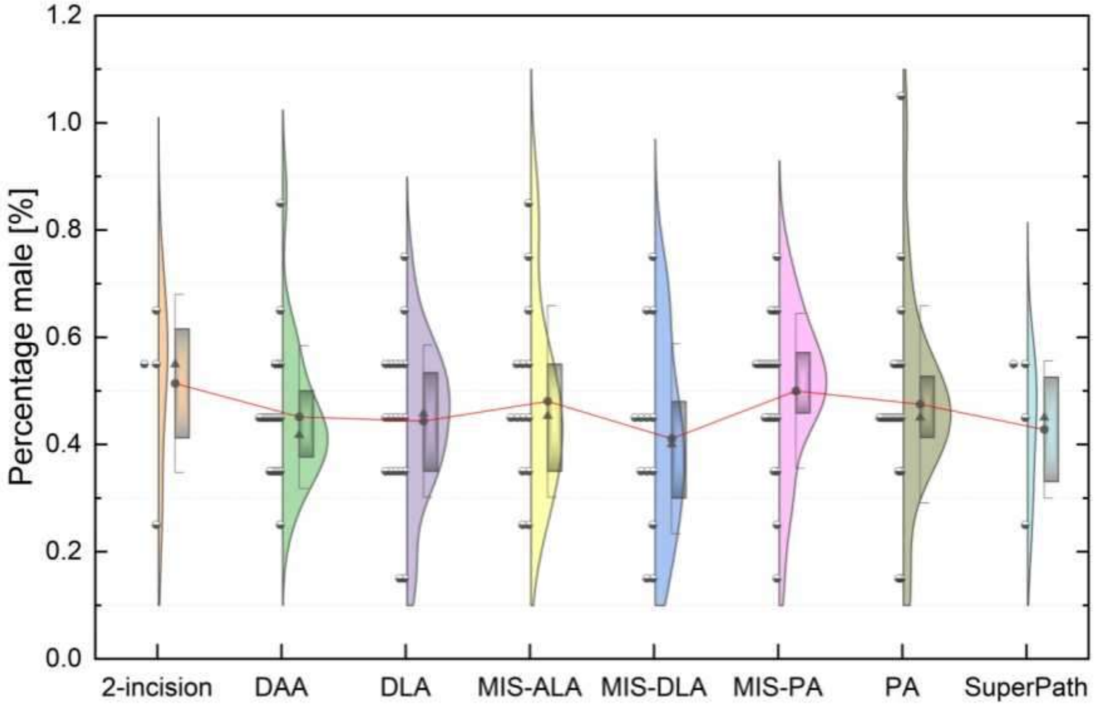

© 2023 Yan L et al. *JAMA Network Open.*

Mean BMI at baseline.

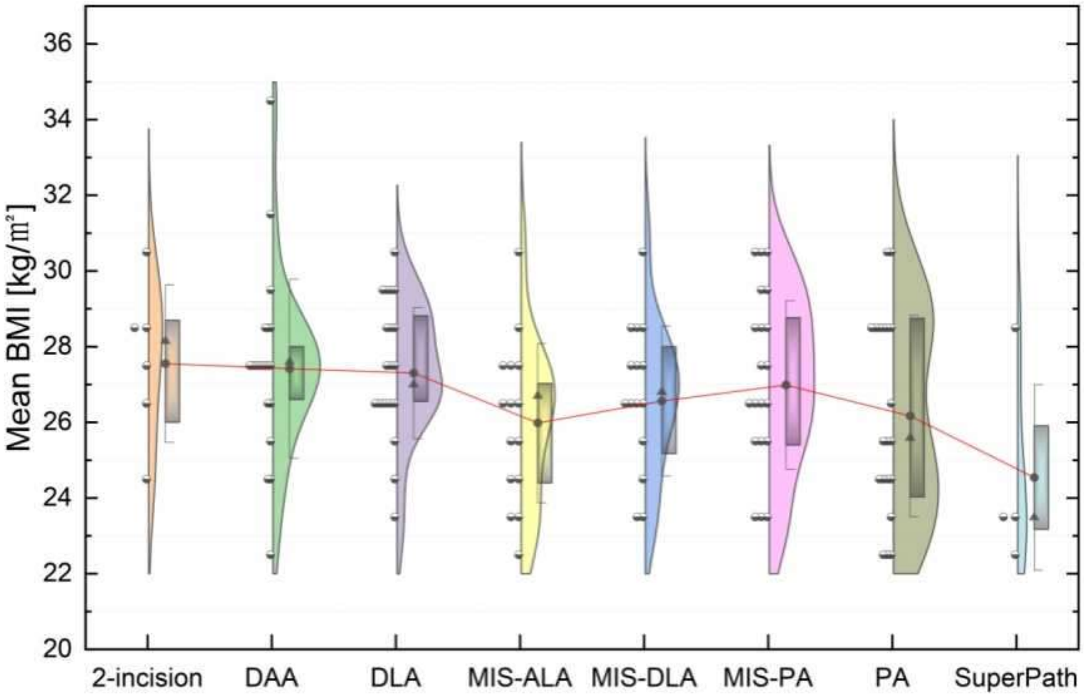

Publication year.

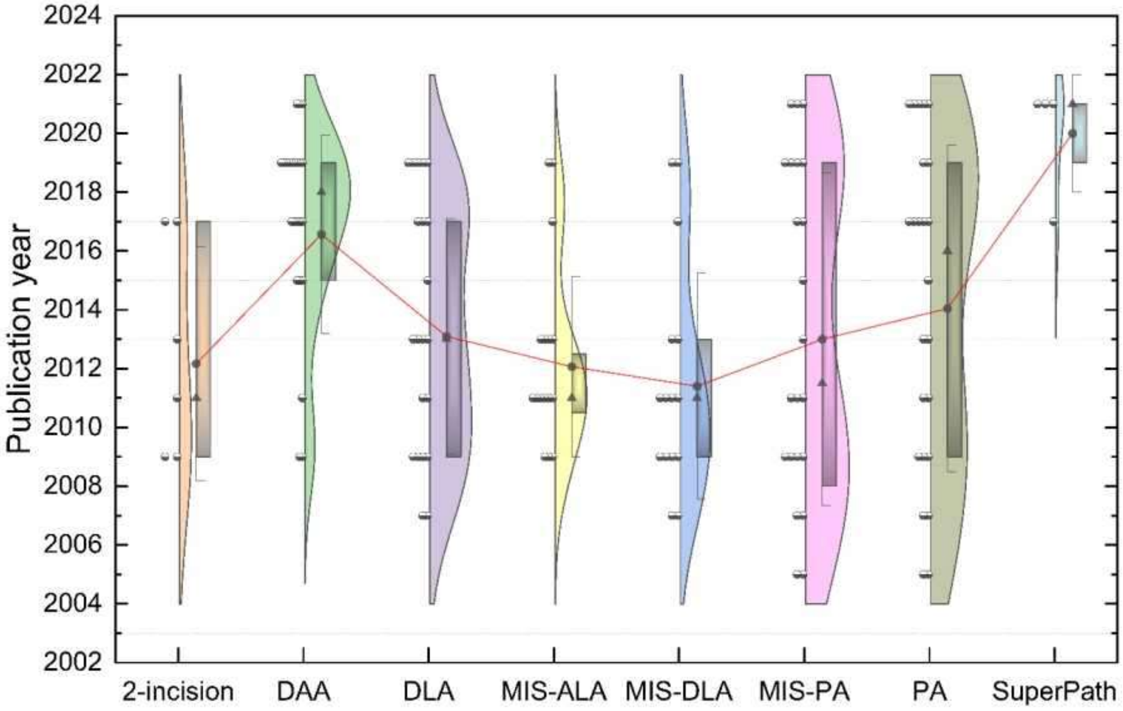

© 2023 Yan L et al. *JAMA Network Open.*

Mean age at baseline.

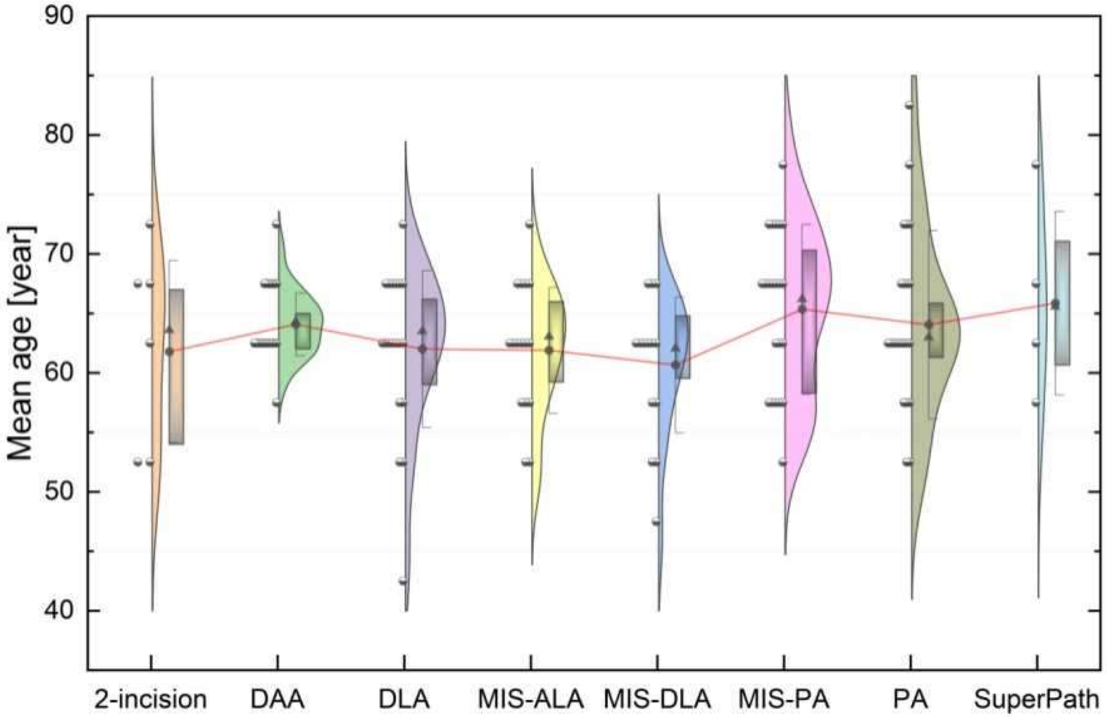

Incision length.

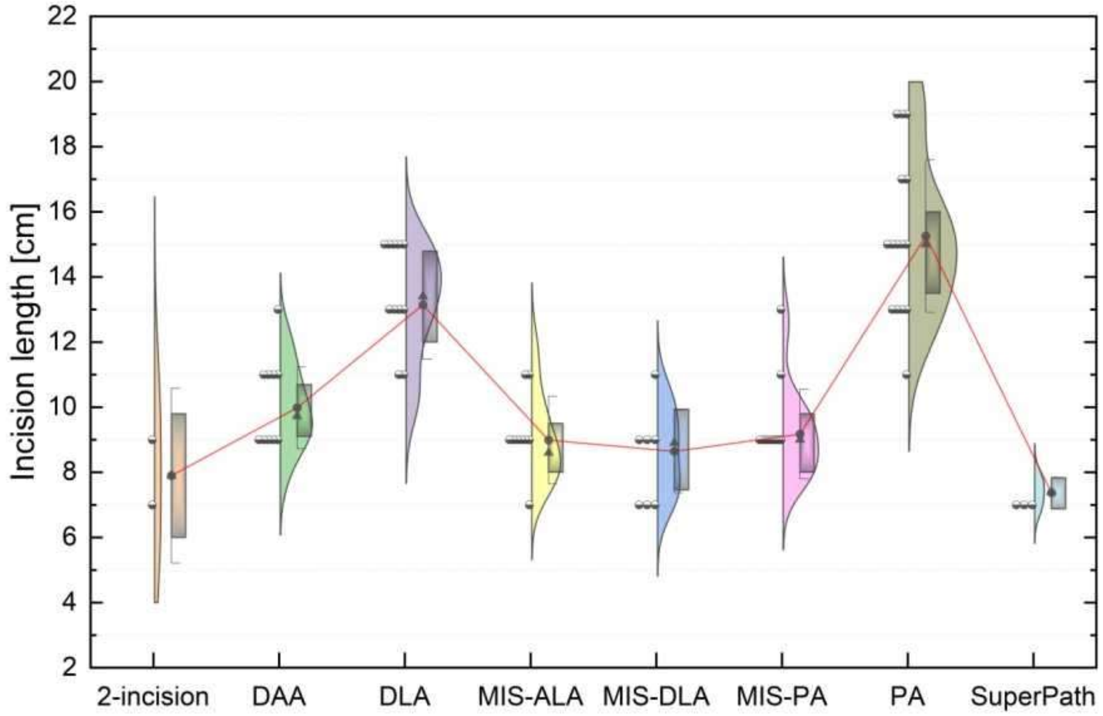

© 2023 Yan L et al. *JAMA Network Open.*

Sample size.

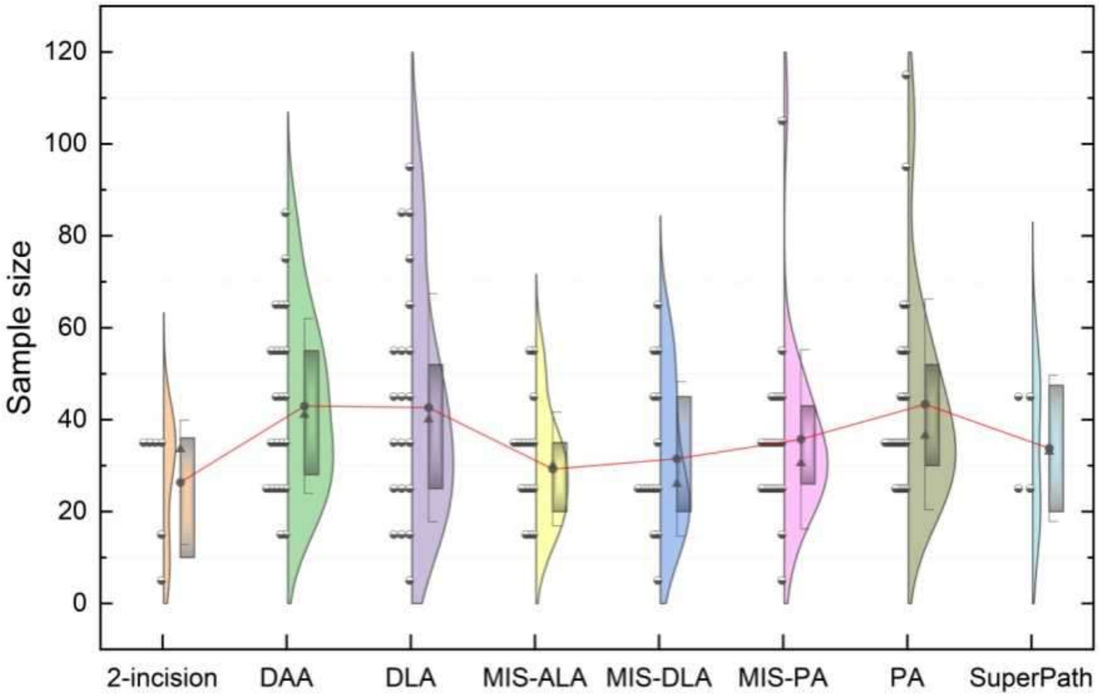

Length of follow-up in studies.

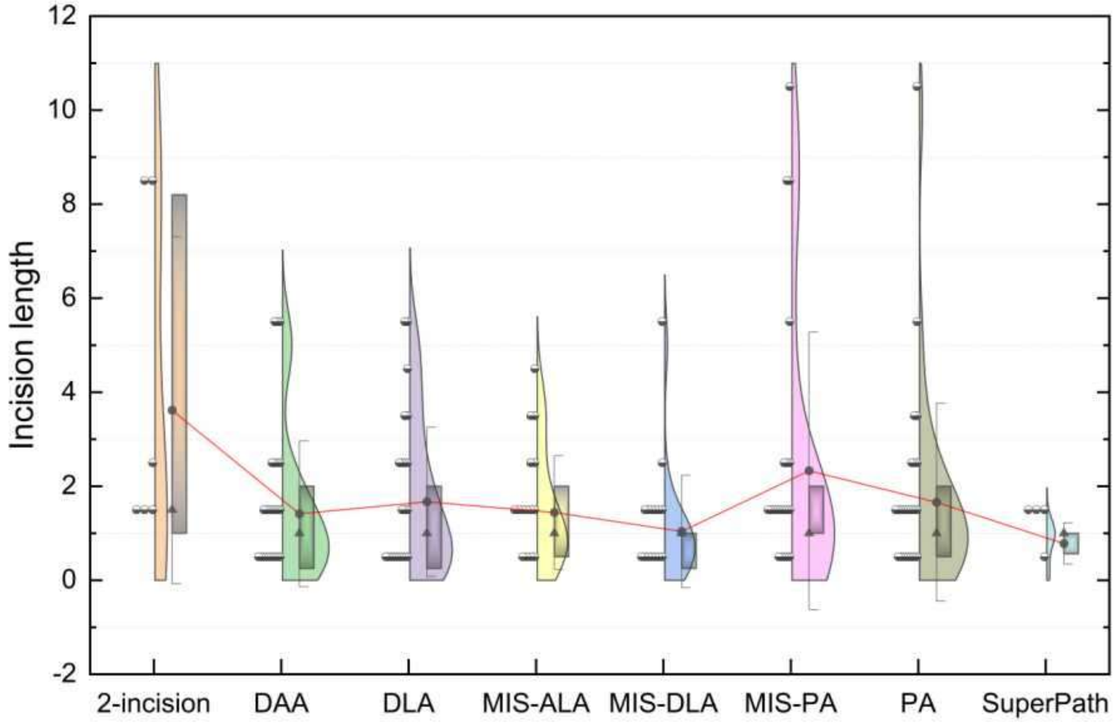

© 2023 Yan L et al. *JAMA Network Open.*

eFigure 6. Inconsistency Assessments

Outcome : Short-term hip score

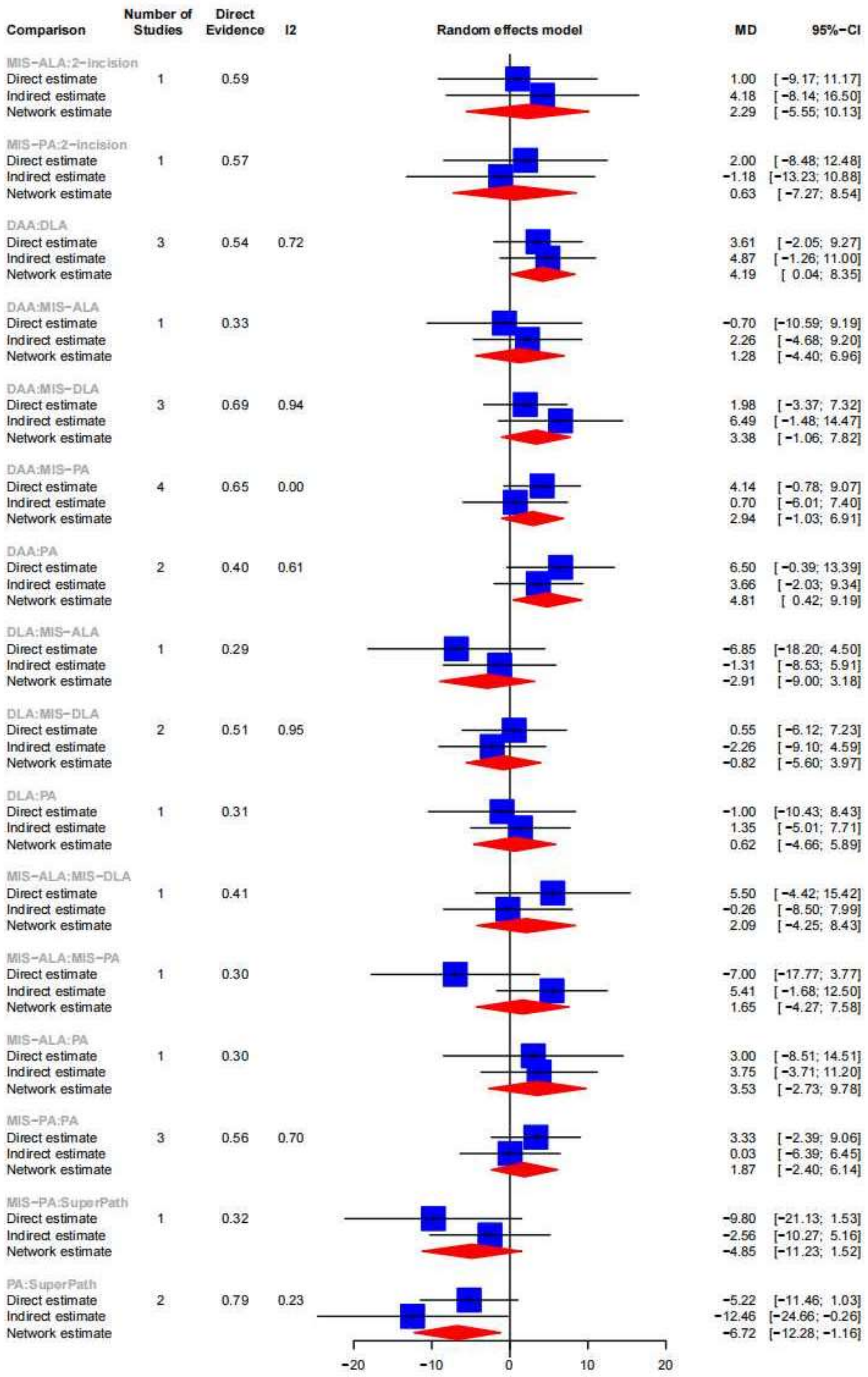

© 2023 Yan L et al. *JAMA Network Open.*

Outcome : Long-term hip score

Comparison	Number of Studies	Direct Evidence	I2	MD	95%-CI
MIS-ALA:2-incision					
Direct estimate	1	0.39		-1.00	[-4.78; 2.78]
Indirect estimate				-0.11	[-3.15; 2.93]
Network estimate				-0.46	[-2.83; 1.91]
MIS-PA:2-incision					
Direct estimate	3	0.78	0.00	1.03	[-1.24; 3.29]
Indirect estimate				0.14	[-4.15; 4.43]
Network estimate				0.83	[-1.17; 2.84]
DAA:DLA					
Direct estimate	4	0.49	0.00	2.19	[-0.16; 4.55]
Indirect estimate				1.37	[-0.96; 3.70]
Network estimate				1.78	[0.12; 3.43]
DAA:MIS-ALA					
Direct estimate	1	0.21		0.00	[-3.49; 3.49]
Indirect estimate				2.11	[0.28; 3.93]
Network estimate				1.65	[0.04; 3.27]
DAA:MIS-DLA					
Direct estimate	1	0.40		2.64	[-0.48; 5.76]
Indirect estimate				2.34	[-0.21; 4.89]
Network estimate				2.46	[0.49; 4.44]
DAA:MIS-PA					
Direct estimate	4	0.71	0.00	0.97	[-0.74; 2.69]
Indirect estimate				-1.14	[-3.83; 1.54]
Network estimate				0.36	[-1.08; 1.81]
DAA:PA					
Direct estimate	2	0.39	0.00	0.42	[-2.12; 2.95]
Indirect estimate				2.17	[0.13; 4.21]
Network estimate				1.48	[-0.11; 3.07]
DLA:MIS-ALA					
Direct estimate	3	0.45	0.00	-0.29	[-2.85; 2.27]
Indirect estimate				0.01	[-2.31; 2.33]
Network estimate				-0.12	[-1.84; 1.59]
DLA:MIS-DLA					
Direct estimate	1	0.13		-3.20	[-9.17; 2.77]
Indirect estimate				1.27	[-1.05; 3.59]
Network estimate				0.68	[-1.48; 2.85]
DLA:PA					
Direct estimate	1	0.41		1.30	[-1.46; 4.06]
Indirect estimate				-1.42	[-3.74; 0.90]
Network estimate				-0.29	[-2.07; 1.48]
MIS-ALA:MIS-DLA					
Direct estimate	3	0.70	0.66	1.09	[-0.98; 3.15]
Indirect estimate				0.15	[-3.01; 3.32]
Network estimate				0.81	[-0.92; 2.54]
MIS-ALA:MIS-PA					
Direct estimate	1	0.15		-6.00	[-10.78; -1.22]
Indirect estimate				-0.47	[-2.47; 1.53]
Network estimate				-1.29	[-3.13; 0.55]
MIS-ALA:PA					
Direct estimate	2	0.45	0.00	-0.16	[-2.74; 2.43]
Indirect estimate				-0.18	[-2.50; 2.14]
Network estimate				-0.17	[-1.90; 1.56]
MIS-DLA:PA					
Direct estimate	1	0.02		-6.00	[-20.09; 8.09]
Indirect estimate				-0.85	[-3.08; 1.37]
Network estimate				-0.98	[-3.18; 1.22]
MIS-PA:PA					
Direct estimate	4	0.41	0.81	1.51	[-1.28; 4.30]
Indirect estimate				0.86	[-1.45; 3.17]
Network estimate				1.12	[-0.66; 2.90]

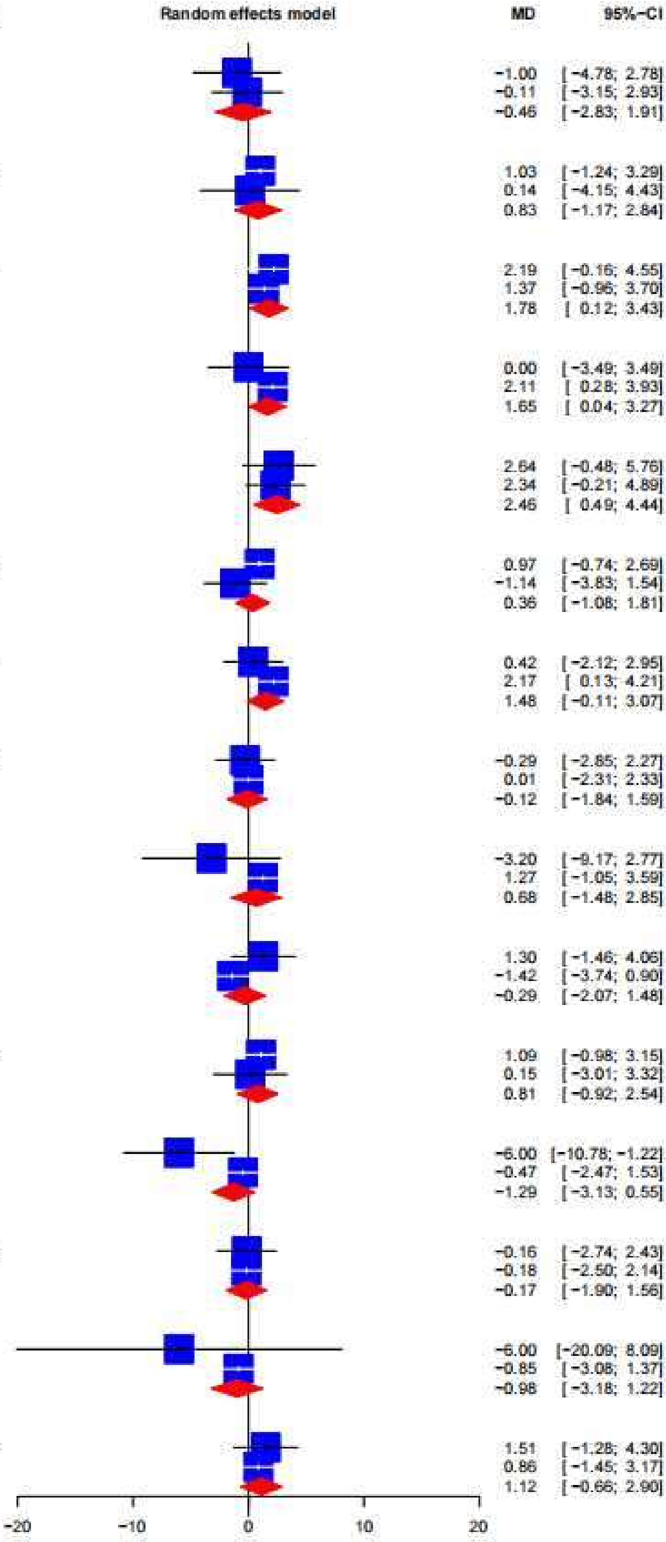

© 2023 Yan L et al. *JAMA Network Open.*

Outcome : Dislocation

Comparison	Number of Studies	Direct Evidence	I2	RR	95%-CI
DAA:DLA					
Direct estimate	2	0.58	0.67	1.87	[0.22; 15.71]
Indirect estimate				0.59	[0.05; 7.11]
Network estimate				1.15	[0.23; 5.79]
DAA:MIS−PA					
Direct estimate	2	0.46	0.00	1.55	[0.19; 12.33]
Indirect estimate				1.20	[0.18; 8.03]
Network estimate				1.35	[0.33; 5.47]
DAA:PA					
Direct estimate	4	0.64	0.00	0.89	[0.21; 3.78]
Indirect estimate				2.00	[0.29; 13.71]
Network estimate				1.19	[0.38; 3.79]
DLA:PA					
Direct estimate	2	0.57	0.48	1.72	[0.20; 14.82]
Indirect estimate				0.54	[0.05; 6.37]
Network estimate				1.04	[0.20; 5.27]
MIS−ALA:MIS−DLA					
Direct estimate	1	0.63		1.04	[0.07; 16.18]
Indirect estimate				4.63	[0.13; 170.40]
Network estimate				1.80	[0.20; 15.97]
MIS−ALA:PA					
Direct estimate	1	0.86		1.50	[0.27; 8.42]
Indirect estimate				0.34	[0.01; 22.24]
Network estimate				1.21	[0.25; 5.95]
MIS−DLA:PA					
Direct estimate	1	0.51		0.32	[0.01; 7.68]
Indirect estimate				1.44	[0.06; 36.88]
Network estimate				0.67	[0.07; 6.47]
MIS−PA:PA					
Direct estimate	4	0.76	0.00	0.94	[0.24; 3.73]
Indirect estimate				0.73	[0.06; 8.50]
Network estimate				0.88	[0.27; 2.94]

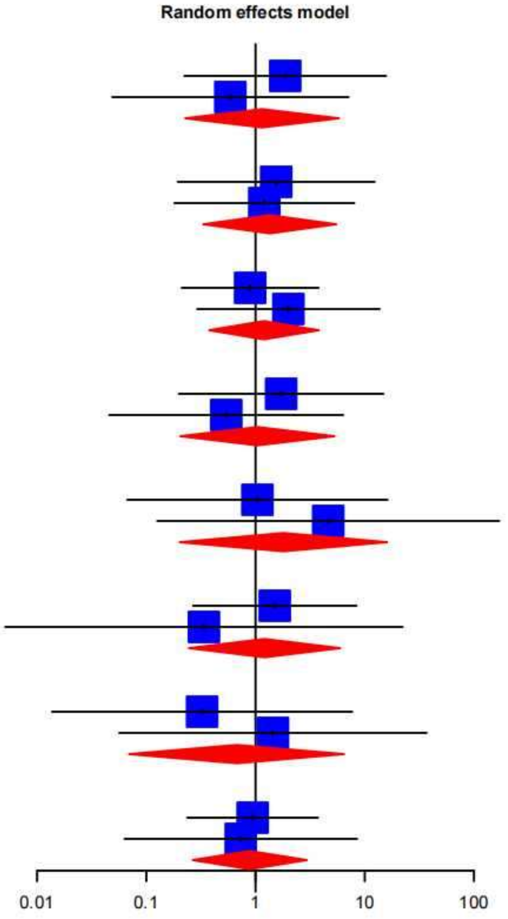

© 2023 Yan L et al. *JAMA Network Open.*

Outcome : Fracture

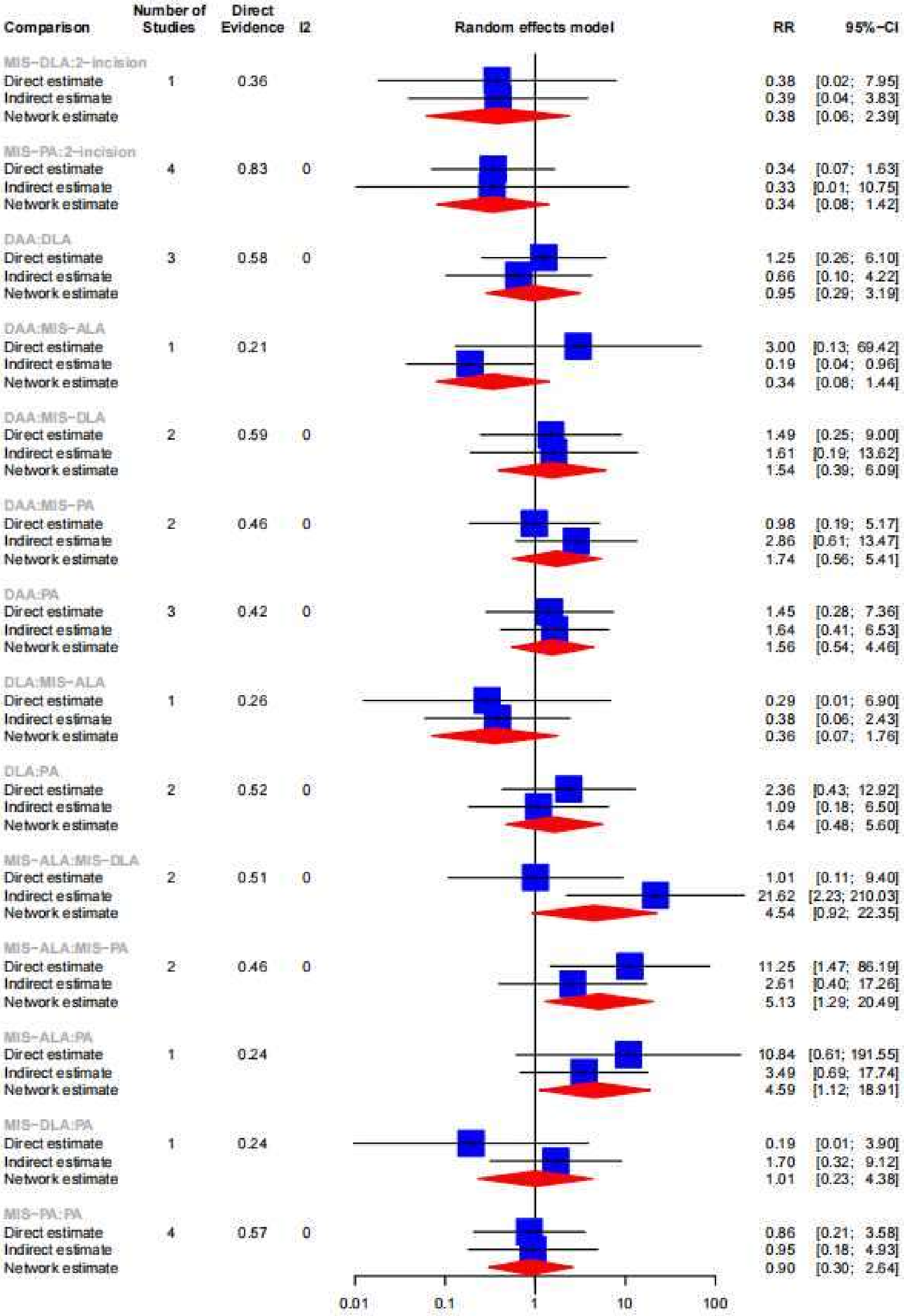

© 2023 Yan L et al. *JAMA Network Open.*

Outcome : Infection

Comparison	Number of Studies	Direct Evidence	I2	RR	95%-CI
**MIS-DLA:2-incision**					
Direct estimate	1	0.54		3.40	[0.16; 71.52]
Indirect estimate				9.53	[0.35; 255.61]
Network estimate				5.47	[0.59; 51.14]
**MIS-PA:2-incision**					
Direct estimate	1	0.72		1.94	[0.18; 20.49]
Indirect estimate				0.69	[0.02; 31.50]
Network estimate				1.46	[0.20; 10.86]
**DAA:DLA**					
Direct estimate	3	0.72	0.22	1.37	[0.23; 8.06]
Indirect estimate				0.47	[0.03; 7.96]
Network estimate				1.02	[0.23; 4.56]
**DAA:MIS-PA**					
Direct estimate	1	0.27		0.33	[0.01; 7.99]
Indirect estimate				0.72	[0.10; 4.99]
Network estimate				0.59	[0.11; 3.06]
**DAA:PA**					
Direct estimate	2	0.60	0.00	1.07	[0.16; 7.01]
Indirect estimate				1.53	[0.15; 15.39]
Network estimate				1.23	[0.29; 5.30]
**DLA:MIS-ALA**					
Direct estimate	2	0.82	0.00	0.68	[0.11; 4.06]
Indirect estimate				2.06	[0.04; 95.80]
Network estimate				0.83	[0.16; 4.19]
**DLA:PA**					
Direct estimate	1	0.33		5.00	[0.25; 99.89]
Indirect estimate				0.61	[0.08; 4.93]
Network estimate				1.21	[0.22; 6.74]
**MIS-ALA:MIS-DLA**					
Direct estimate	2	0.75	0.00	0.14	[0.02; 1.17]
Indirect estimate				0.43	[0.01; 16.77]
Network estimate				0.19	[0.03; 1.16]
**MIS-DLA:MIS-PA**					
Direct estimate	1	0.42		3.40	[0.16; 71.52]
Indirect estimate				4.00	[0.30; 53.29]
Network estimate				3.74	[0.52; 26.88]
**MIS-PA:PA**					
Direct estimate	4	0.83	0.00	1.71	[0.46; 6.43]
Indirect estimate				5.59	[0.31; 100.27]
Network estimate				2.10	[0.63; 7.00]

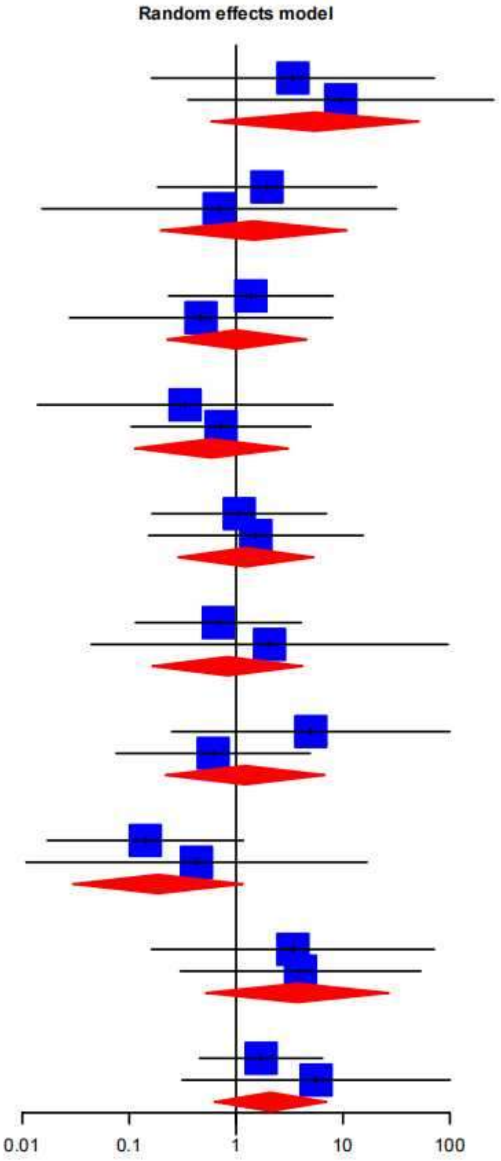

© 2023 Yan L et al. *JAMA Network Open.*

Outcome : Nerve injury

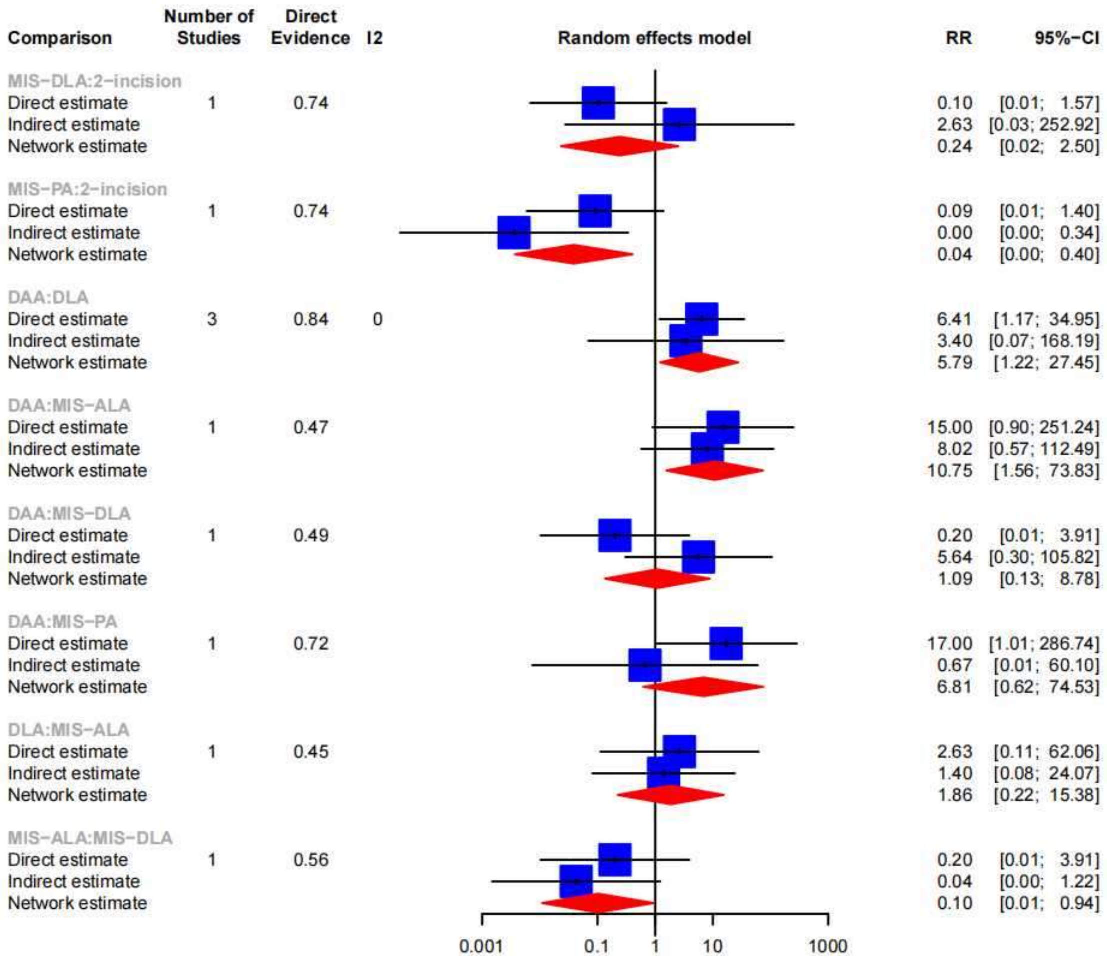

© 2023 Yan L et al. *JAMA Network Open.*

Outcome : Reoperation

Comparison	Number of Studies	Direct Evidence	I2	RR	95%-CI
**DAA:DLA**					
Direct estimate	5	0.76	0.18	1.04	[0.26; 4.14]
Indirect estimate				0.83	[0.07; 9.43]
Network estimate				0.99	[0.30; 3.27]
**DAA:PA**					
Direct estimate	3	0.77	0.00	1.32	[0.34; 5.09]
Indirect estimate				1.66	[0.14; 19.27]
Network estimate				1.39	[0.43; 4.54]
**DLA:MIS−ALA**					
Direct estimate	1	0.35		1.00	[0.07; 15.26]
Indirect estimate				0.44	[0.06; 3.23]
Network estimate				0.58	[0.12; 2.93]
**DLA:PA**					
Direct estimate	1	0.26		1.02	[0.06; 16.09]
Indirect estimate				1.58	[0.31; 7.98]
Network estimate				1.41	[0.35; 5.71]
**MIS−ALA:MIS−DLA**					
Direct estimate	1	0.41		3.00	[0.13; 69.42]
Indirect estimate				5.21	[0.37; 72.87]
Network estimate				4.15	[0.55; 31.27]
**MIS−ALA:MIS−PA**					
Direct estimate	2	0.54	0.00	5.72	[0.99; 33.09]
Indirect estimate				1.35	[0.20; 9.05]
Network estimate				2.94	[0.81; 10.69]
**MIS−ALA:PA**					
Direct estimate	2	0.55	0.40	2.05	[0.44; 9.60]
Indirect estimate				2.94	[0.54; 15.98]
Network estimate				2.42	[0.77; 7.56]
**MIS−DLA:PA**					
Direct estimate	1	0.67		0.49	[0.05; 5.11]
Indirect estimate				0.84	[0.03; 24.28]
Network estimate				0.58	[0.08; 4.00]
**MIS−PA:PA**					
Direct estimate	5	0.85	0.00	0.96	[0.30; 3.13]
Indirect estimate				0.32	[0.02; 5.47]
Network estimate				0.82	[0.28; 2.43]

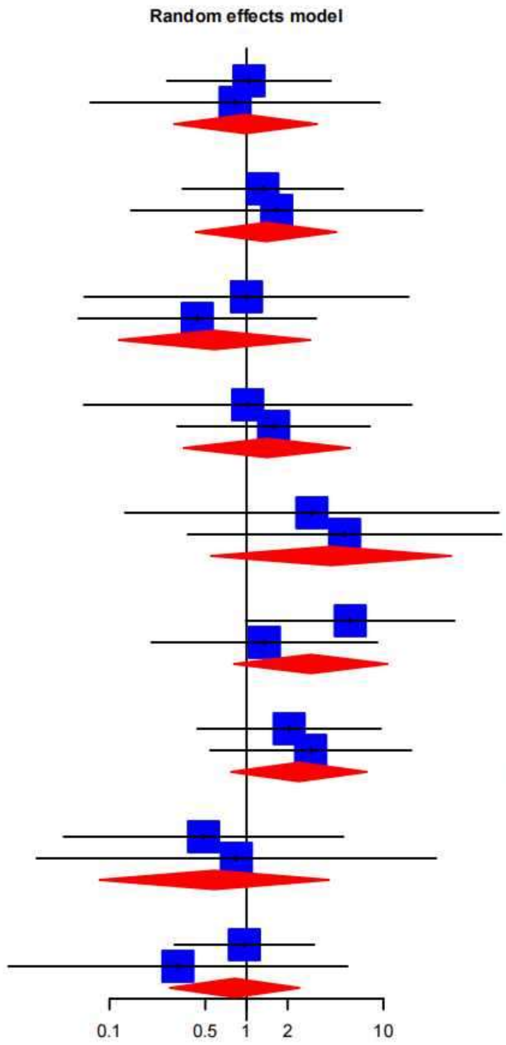

© 2023 Yan L et al. *JAMA Network Open.*

Outcome : Thromboembolism

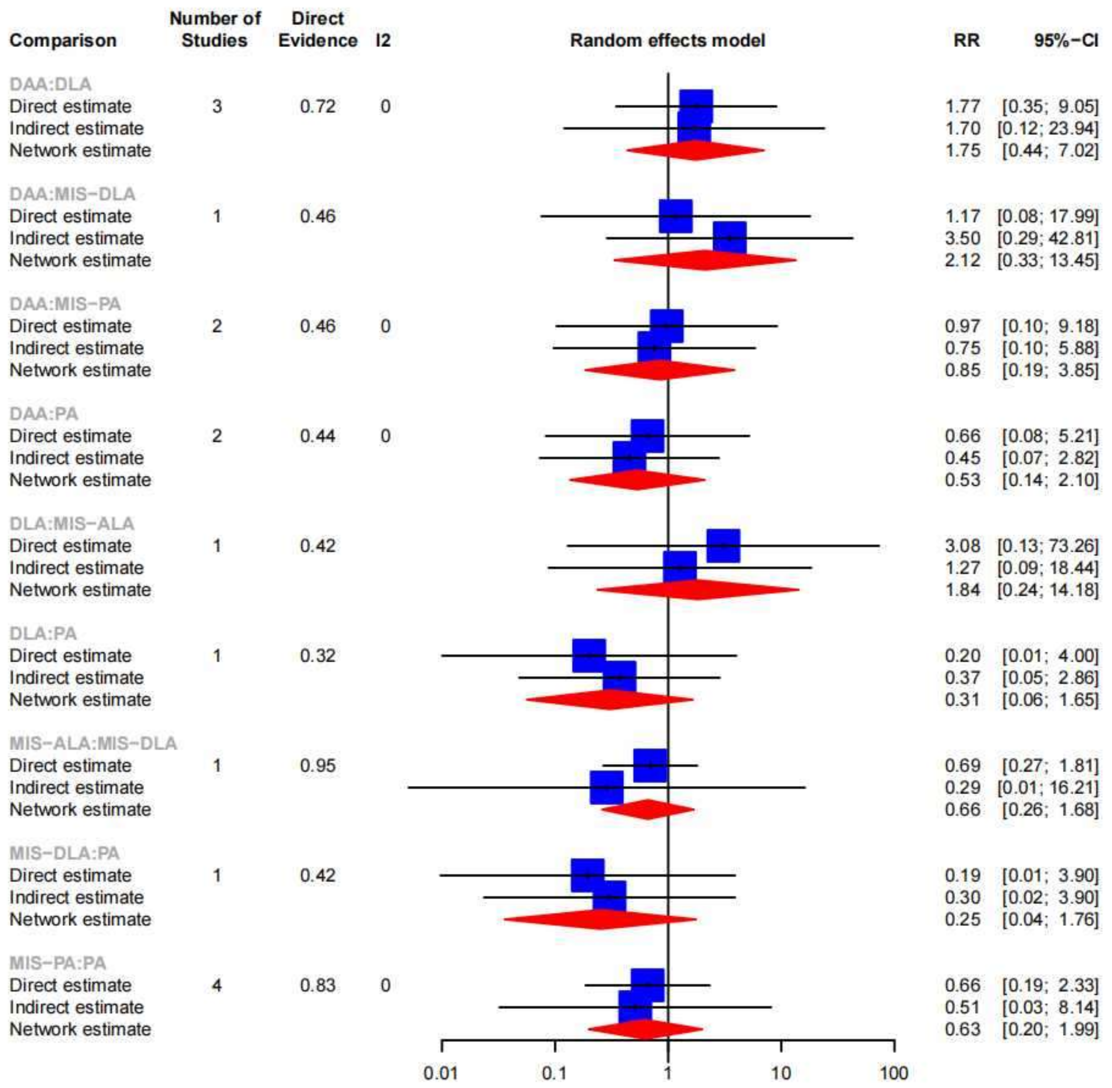

© 2023 Yan L et al. *JAMA Network Open.*

Outcome : Analgesic consumption

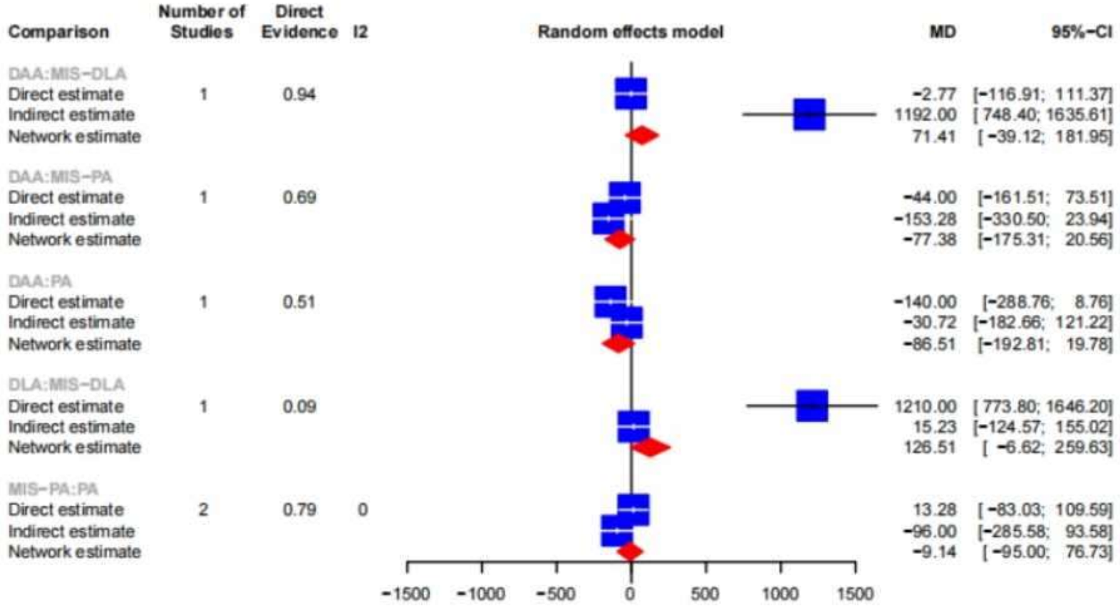

© 2023 Yan L et al. *JAMA Network Open.*

Outcome : Creatine kinase change

Comparison	Number of Studies	Direct Evidence	I2	MD	95%-CI
DLA:DAA					
Direct estimate	3	0.91	0.80	12.79	[−91.99; 117.57]
Indirect estimate				71.35	[−270.83; 413.53]
Network estimate				17.81	[−82.38; 118.00]
PA:DAA					
Direct estimate	1	0.75		21.70	[−156.42; 199.82]
Indirect estimate				−36.86	[−347.25; 273.53]
Network estimate				7.19	[−147.29; 161.68]
DLA:MIS−ALA					
Direct estimate	1	0.54		−1.71	[−180.19; 176.77]
Indirect estimate				39.55	[−153.47; 232.56]
Network estimate				17.31	[−113.74; 148.35]
DLA:MIS−DLA					
Direct estimate	1	0.53		50.60	[−145.52; 246.72]
Indirect estimate				−35.37	[−242.95; 172.21]
Network estimate				10.05	[−132.51; 152.61]
MIS−ALA:MIS−DLA					
Direct estimate	2	0.77	0.15	−26.77	[−162.81; 109.28]
Indirect estimate				59.21	[−191.88; 310.29]
Network estimate				−7.25	[−126.87; 112.36]
MIS−ALA:MIS−PA					
Direct estimate	1	0.84		−39.00	[−277.83; 199.83]
Indirect estimate				−138.09	[−694.57; 418.39]
Network estimate				−54.41	[−273.88; 165.06]
MIS−ALA:PA					
Direct estimate	1	0.49		23.00	[−231.81; 277.81]
Indirect estimate				−35.56	[−286.83; 215.71]
Network estimate				−6.69	[−185.60; 172.23]
MIS−PA:PA					
Direct estimate	1	0.86		62.00	[−171.57; 295.57]
Indirect estimate				−40.31	[−620.34; 539.71]
Network estimate				47.72	[−168.94; 264.38]

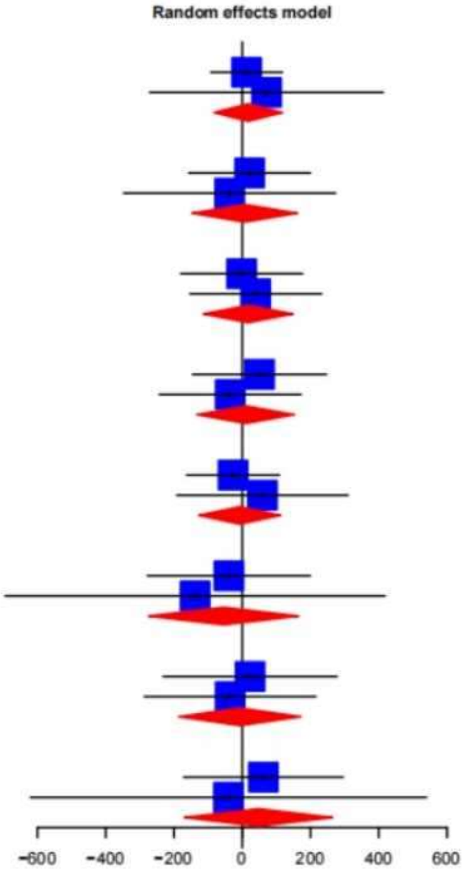

© 2023 Yan L et al. *JAMA Network Open.*

Outcome : C-reactive protein change

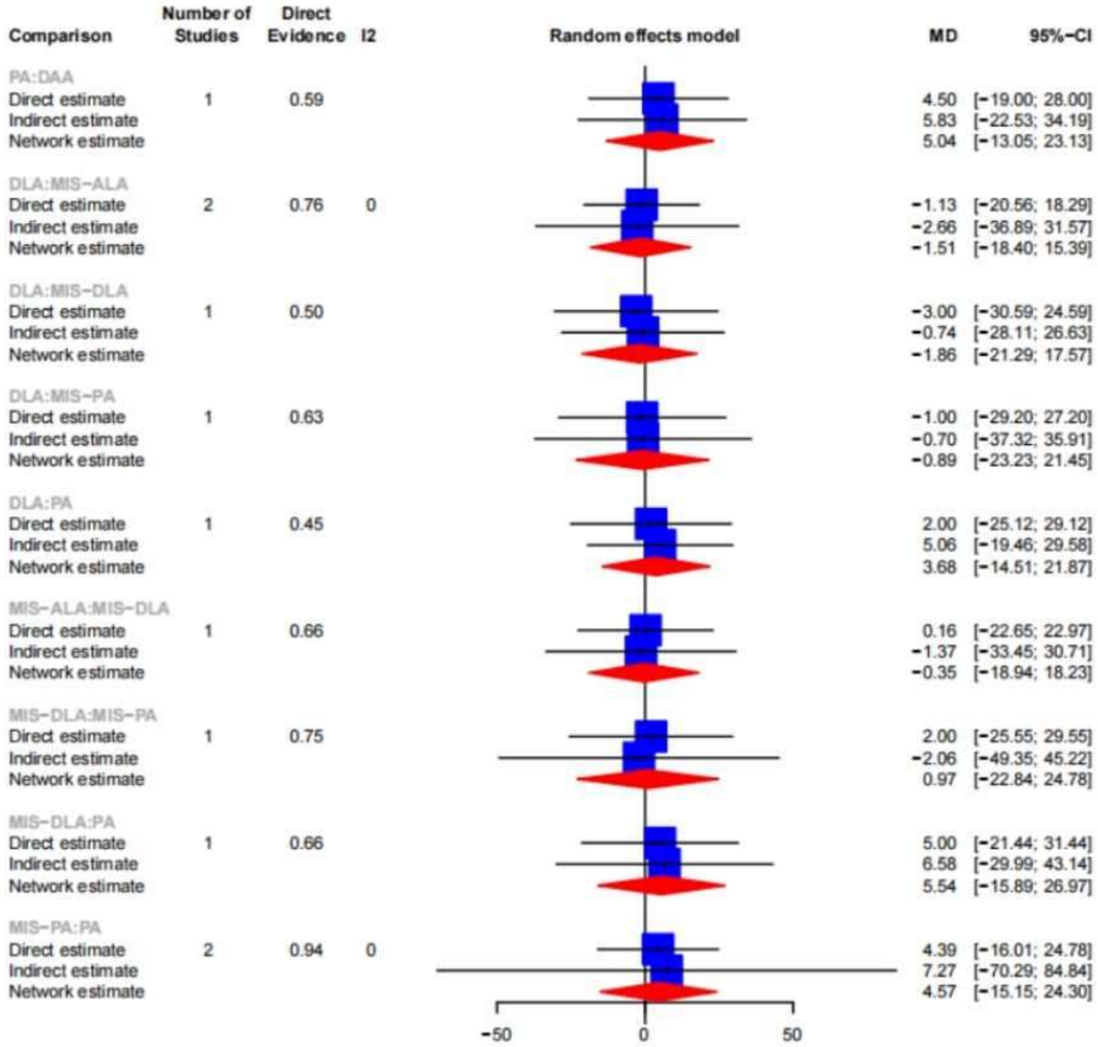

© 2023 Yan L et al. *JAMA Network Open.*

Outcome : Hemoglobin change

Comparison	Number of Studies	Direct Evidence	I2	MD	95%-CI
DAA:DLA					
Direct estimate	2	0.65	0.57	-0.67	[-5.42; 4.08]
Indirect estimate				2.40	[-4.10; 8.90]
Network estimate				0.40	[-3.44; 4.23]
DAA:MIS-DLA					
Direct estimate	1	0.31		3.60	[-4.60; 11.80]
Indirect estimate				-4.69	[-10.15; 0.78]
Network estimate				-2.14	[-6.68; 2.41]
DAA:PA					
Direct estimate	2	0.58	0.00	-0.99	[-6.41; 4.43]
Indirect estimate				1.66	[-4.71; 8.03]
Network estimate				0.12	[-4.00; 4.25]
DLA:MIS-ALA					
Direct estimate	2	0.85	0.26	-1.65	[-5.20; 1.90]
Indirect estimate				-3.01	[-11.57; 5.56]
Network estimate				-1.85	[-5.13; 1.43]
DLA:MIS-DLA					
Direct estimate	3	0.61	0.76	-4.73	[-10.16; 0.71]
Indirect estimate				0.90	[-5.90; 7.70]
Network estimate				-2.53	[-6.78; 1.71]
DLA:MIS-PA					
Direct estimate	1	0.12		3.00	[-11.37; 17.37]
Indirect estimate				-2.43	[-7.78; 2.93]
Network estimate				-1.76	[-6.78; 3.25]
DLA:PA					
Direct estimate	1	0.10		7.00	[-6.50; 20.50]
Indirect estimate				-1.11	[-5.70; 3.47]
Network estimate				-0.27	[-4.62; 4.07]
MIS-ALA:MIS-PA					
Direct estimate	1	0.46		0.00	[-7.63; 7.63]
Indirect estimate				0.16	[-6.88; 7.19]
Network estimate				0.08	[-5.09; 5.26]
MIS-ALA:PA					
Direct estimate	1	0.34		3.22	[-4.78; 11.22]
Indirect estimate				0.71	[-5.09; 6.51]
Network estimate				1.58	[-3.12; 6.27]
MIS-DLA:MIS-PA					
Direct estimate	1	0.27		1.00	[-8.93; 10.93]
Indirect estimate				0.68	[-5.41; 6.78]
Network estimate				0.77	[-4.43; 5.96]
MIS-DLA:PA					
Direct estimate	2	0.52	0.00	2.52	[-3.61; 8.65]
Indirect estimate				1.97	[-4.45; 8.40]
Network estimate				2.26	[-2.17; 6.69]
MIS-PA:PA					
Direct estimate	5	0.95	0.00	1.86	[-1.79; 5.52]
Indirect estimate				-5.74	[-21.82; 10.34]
Network estimate				1.49	[-2.07; 5.06]

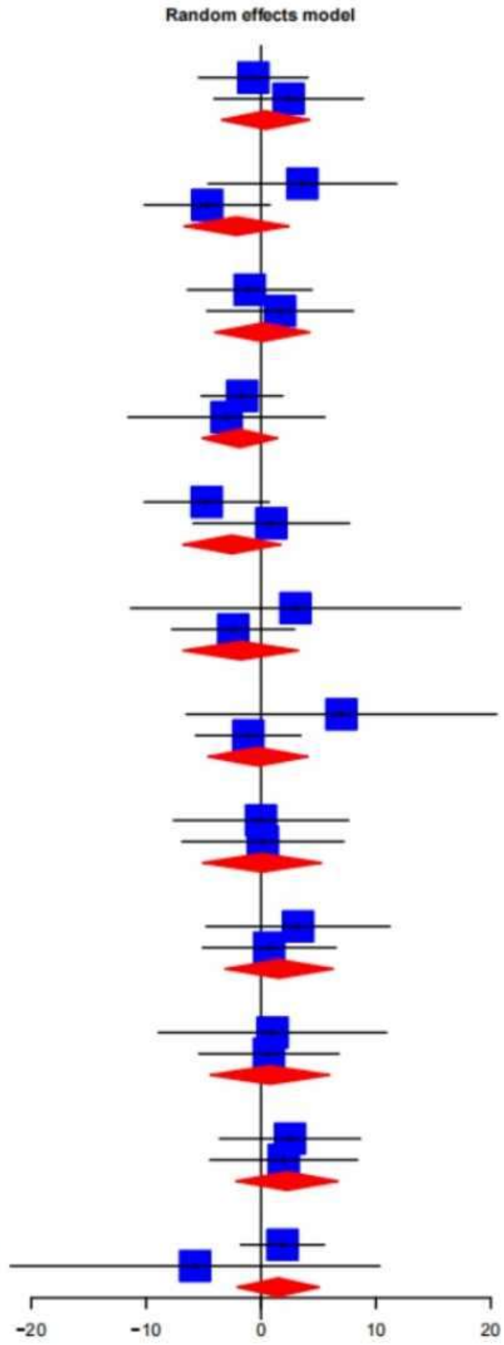

© 2023 Yan L et al. *JAMA Network Open.*

Outcome : Interleukin-6 change

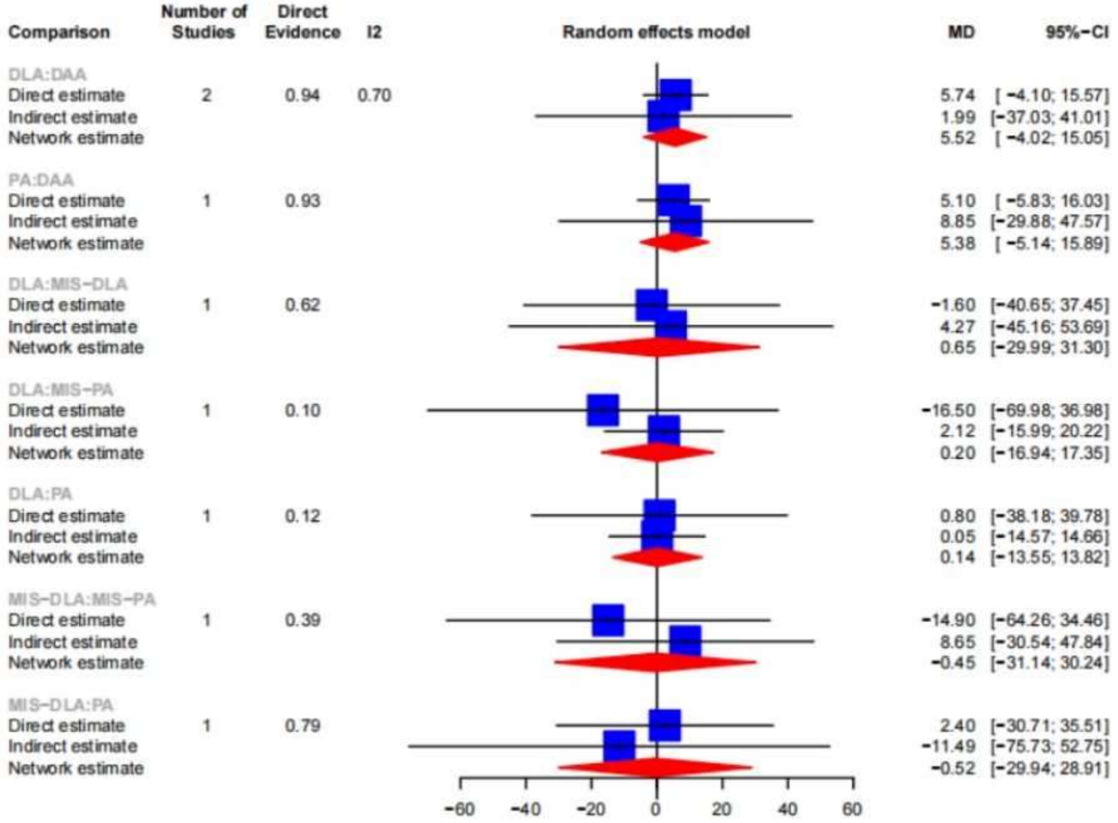

© 2023 Yan L et al. *JAMA Network Open.*

Outcome : Leg length discrepancy

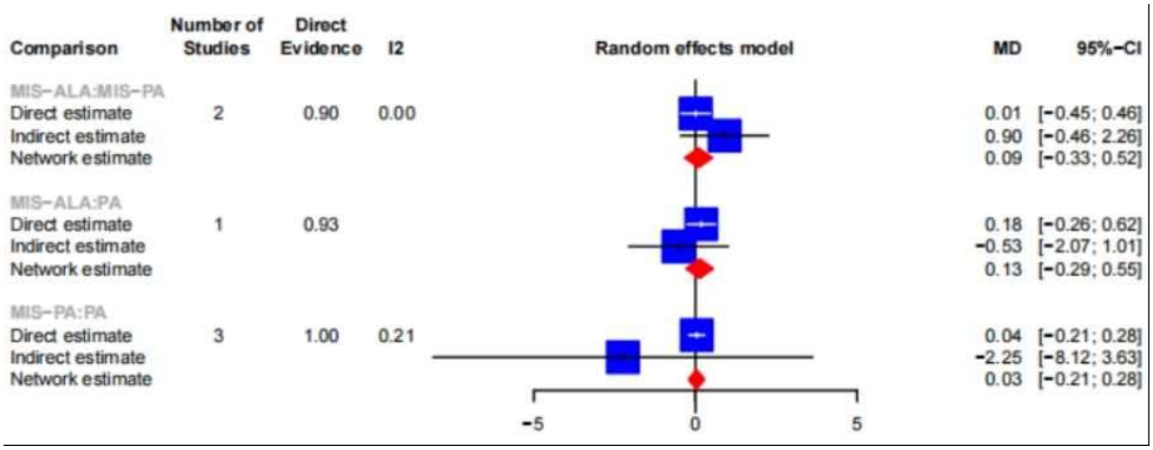

© 2023 Yan L et al. *JAMA Network Open.*

Outcome : Stem alignment

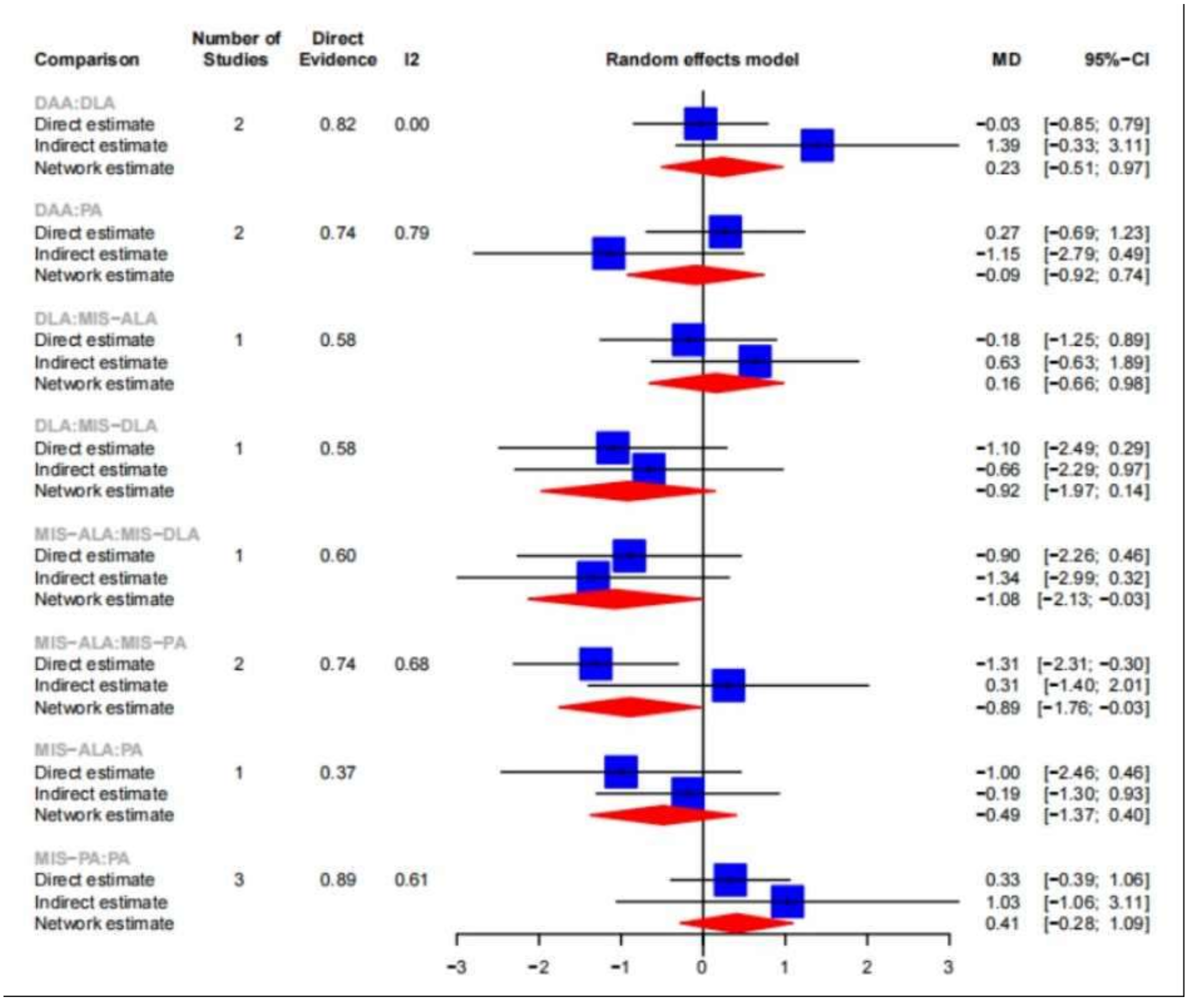

Outcome : Step length change

Comparison	Number of Studies	Direct Evidence	I2	MD	95%-CI
DLA:DAA					
Direct estimate	2	0.86	0	−0.00	[−0.34; 0.33]
Indirect estimate				−2.53	[−3.35; −1.70]
Network estimate				−0.35	[−0.66; −0.04]
MIS−DLA:DAA					
Direct estimate	1	0.54		−2.54	[−3.14; −1.94]
Indirect estimate				−0.02	[−0.67; 0.64]
Network estimate				−1.38	[−1.82; −0.94]
DLA:MIS−ALA					
Direct estimate	2	0.87	0	0.01	[−0.31; 0.34]
Indirect estimate				2.54	[1.71; 3.36]
Network estimate				0.35	[0.05; 0.66]
MIS−ALA:MIS−DLA					
Direct estimate	1	0.73		0.00	[−0.46; 0.46]
Indirect estimate				2.52	[1.76; 3.28]
Network estimate				0.67	[0.28; 1.07]
MIS−PA:PA					
Direct estimate	1	0.67		−0.01	[−0.47; 0.45]
Indirect estimate				−0.09	[−0.75; 0.58]
Network estimate				−0.03	[−0.42; 0.35]
MIS−PA:SuperPath					
Direct estimate	1	0.65		−0.09	[−0.57; 0.39]
Indirect estimate				−0.01	[−0.67; 0.64]
Network estimate				−0.06	[−0.45; 0.32]
PA:SuperPath					
Direct estimate	1	0.68		−0.00	[−0.46; 0.46]
Indirect estimate				−0.08	[−0.75; 0.59]
Network estimate				−0.03	[−0.41; 0.35]

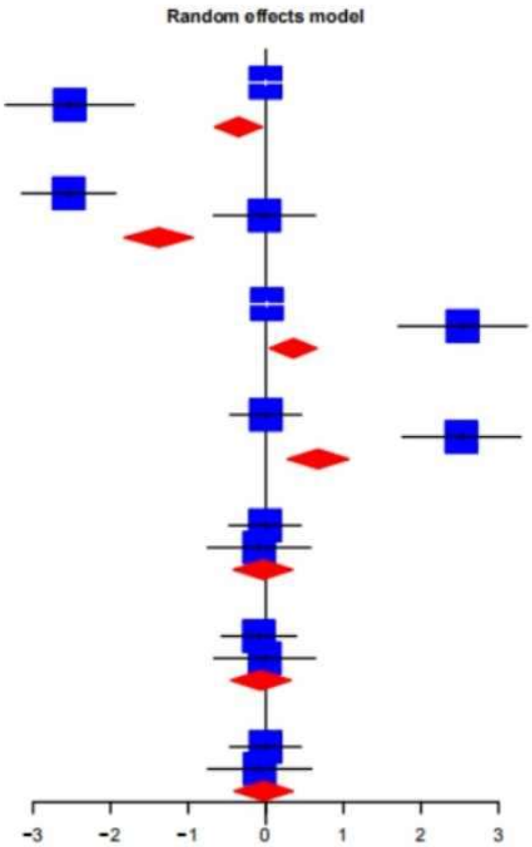

© 2023 Yan L et al. *JAMA Network Open.*

Outcome : Volume of blood transfusion change

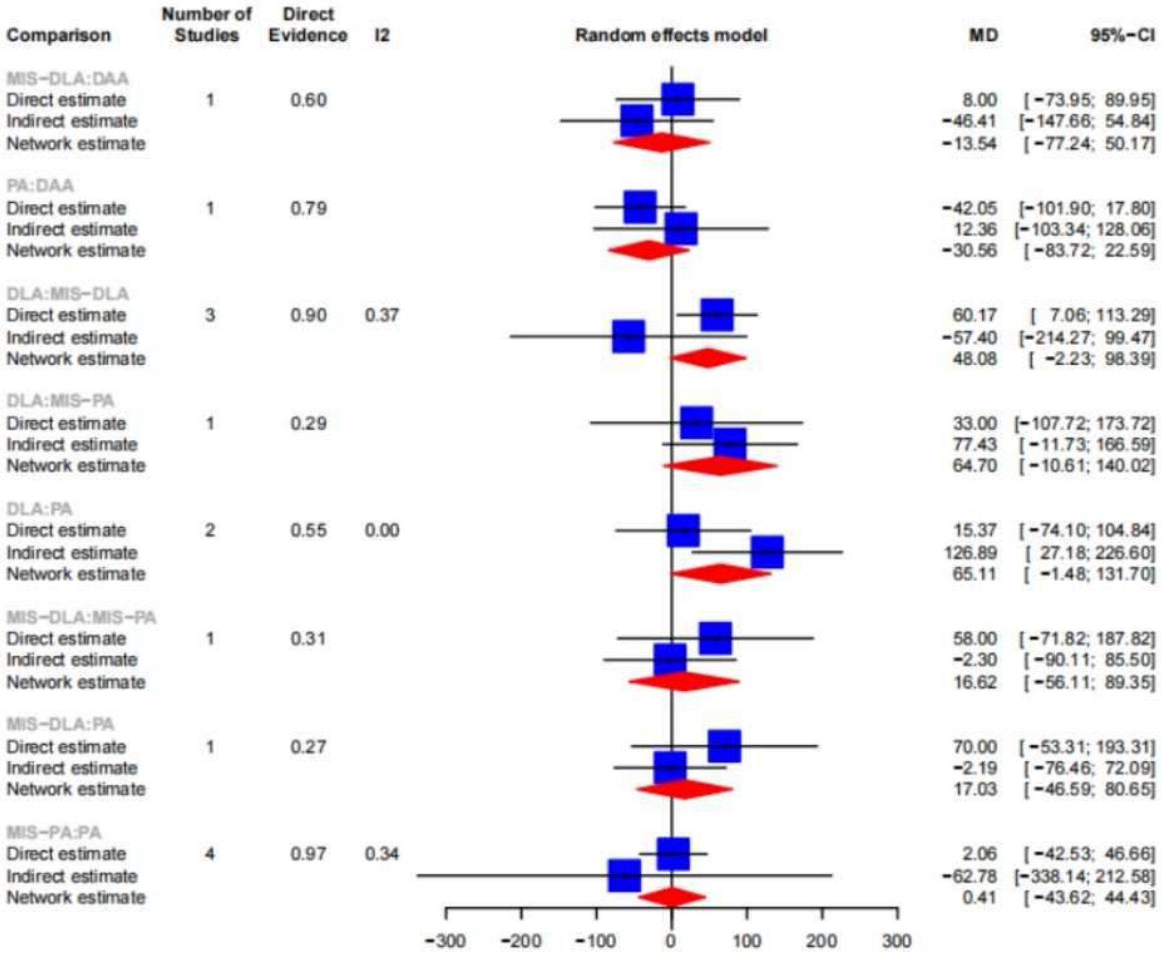

© 2023 Yan L et al. *JAMA Network Open.*

Outcome : Walking speed change

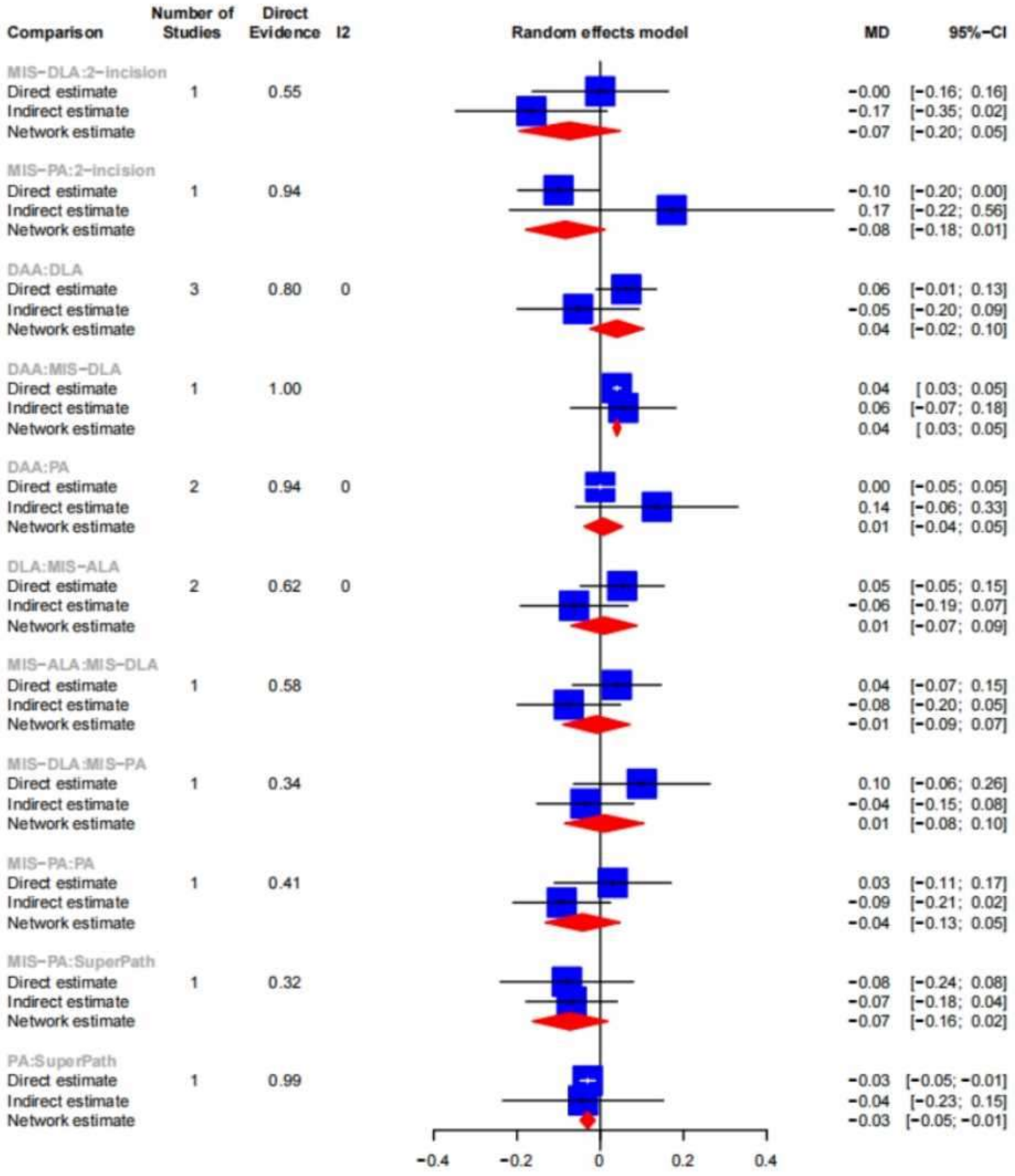

© 2023 Yan L et al. *JAMA Network Open.*

eFigure 7. Publication Bias: Funnel Plot

Outcome : Hip score change

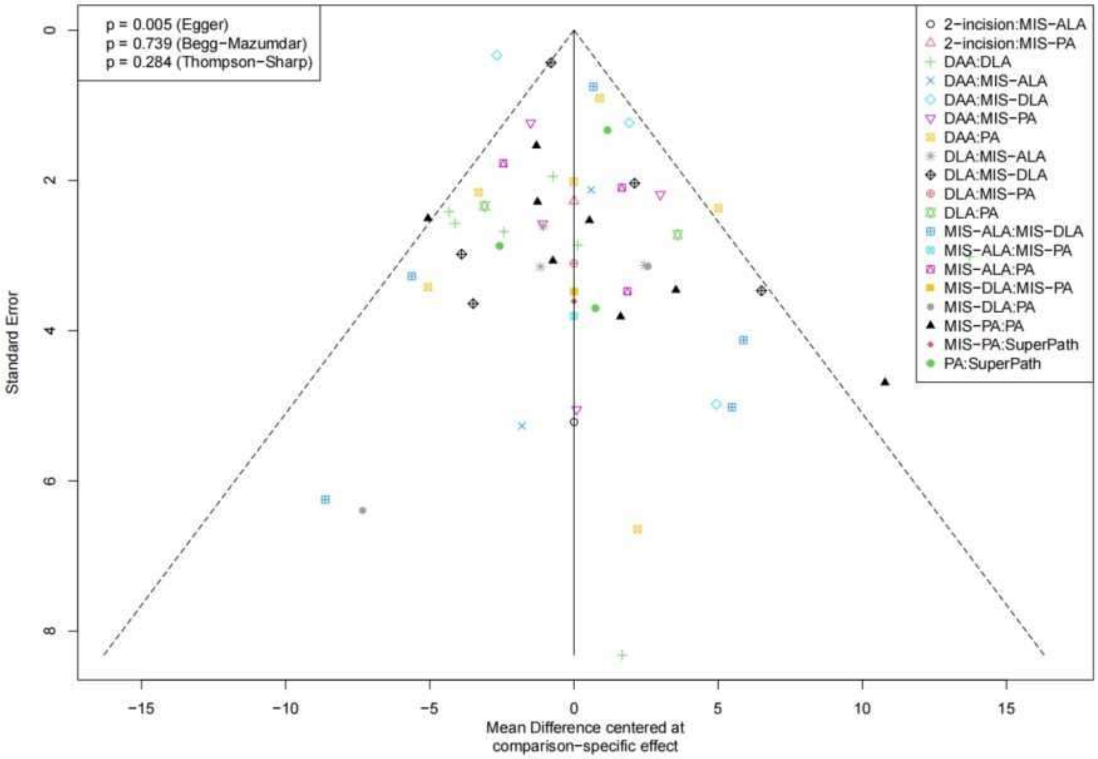

© 2023 Yan L et al. *JAMA Network Open.*

Outcome : Pain score change

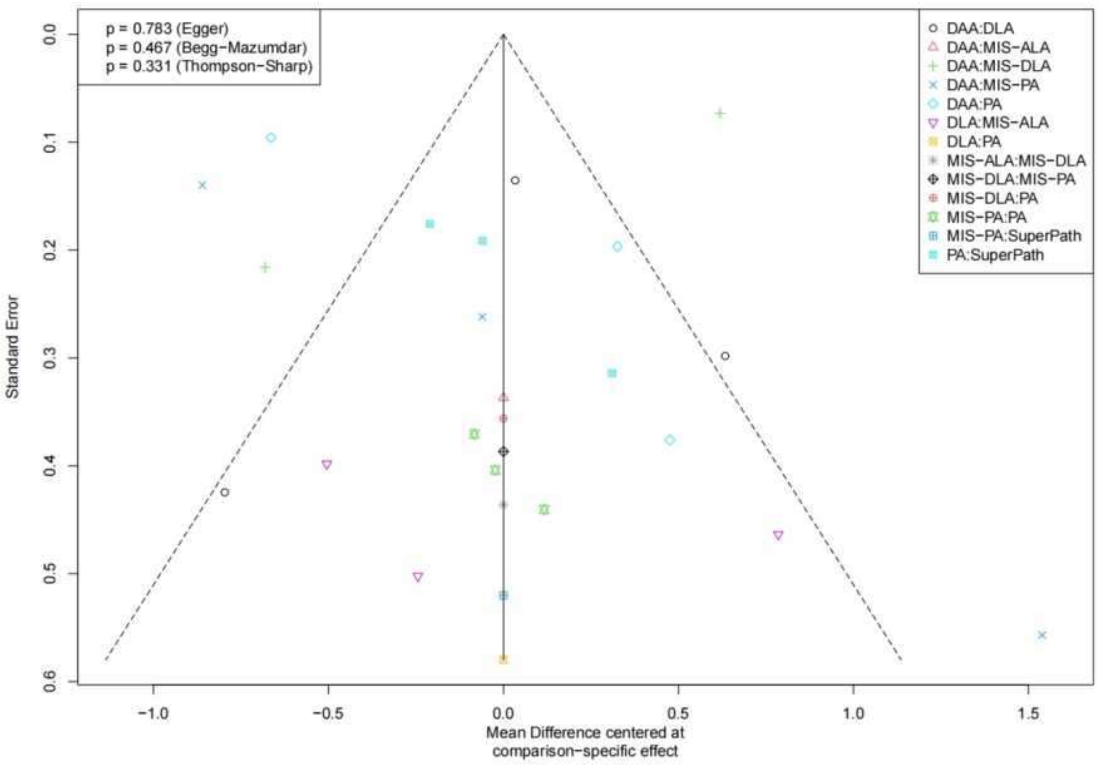

© 2023 Yan L et al. *JAMA Network Open.*

Outcome : Hospitalization time

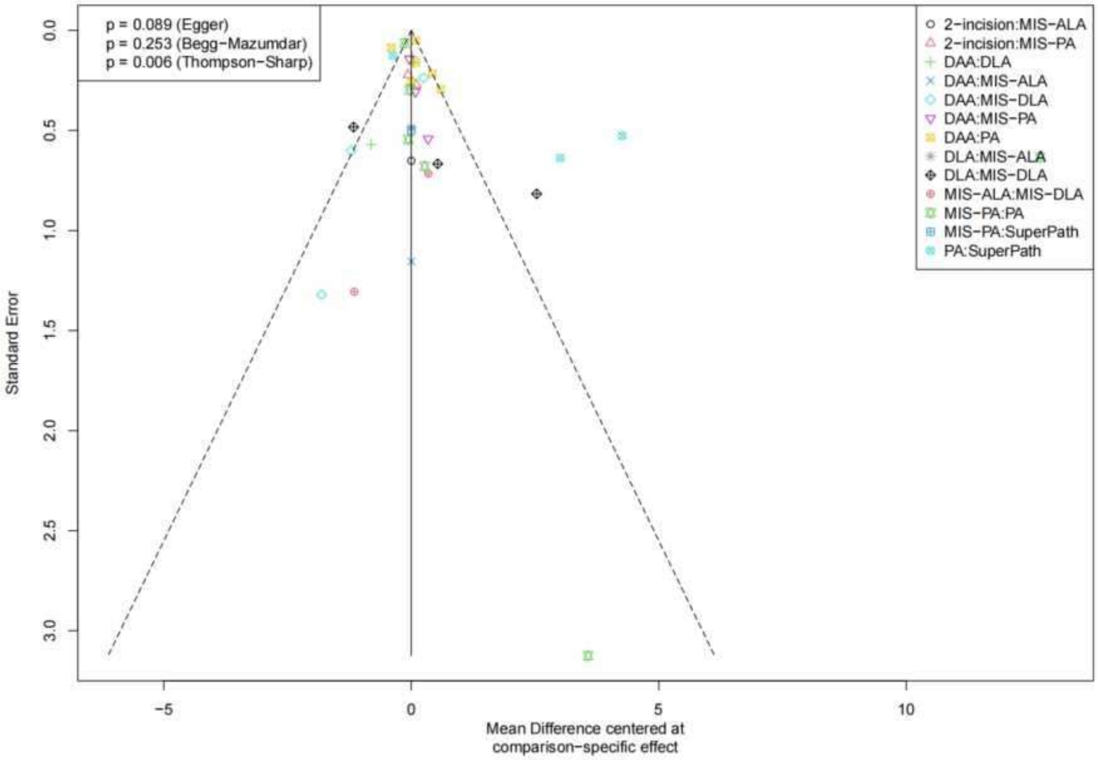

© 2023 Yan L et al. *JAMA Network Open.*

Outcome : Operation time

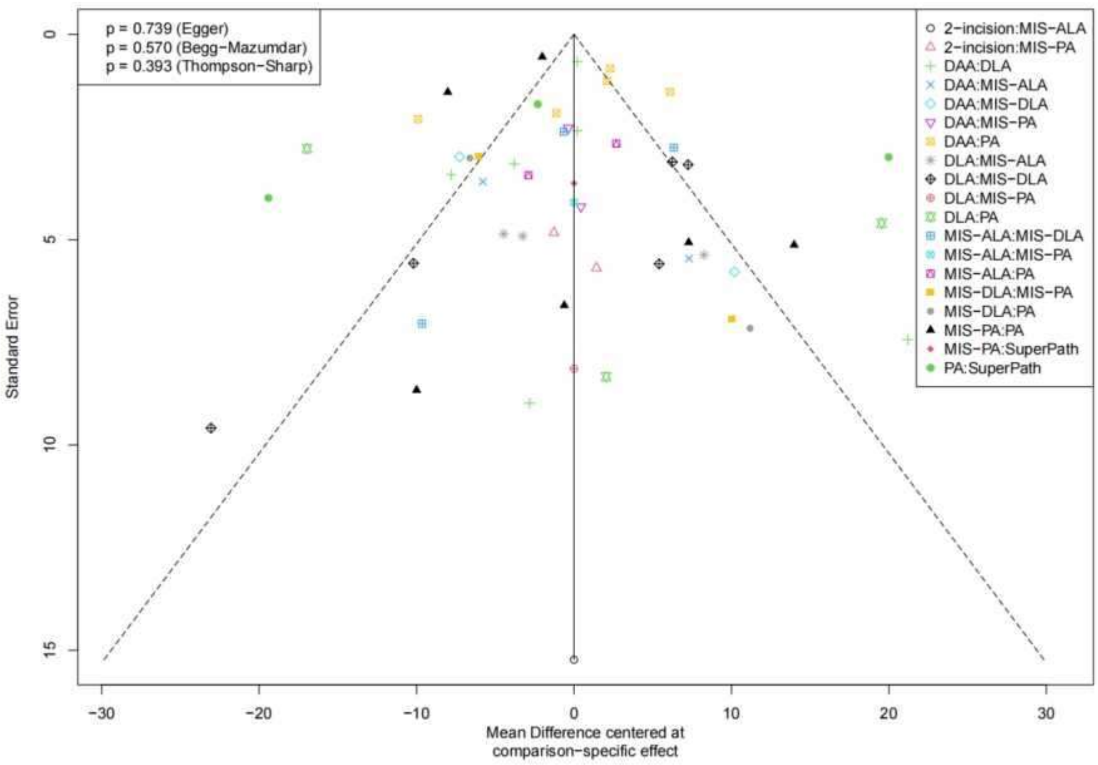

© 2023 Yan L et al. *JAMA Network Open.*

Outcome : Blood loss

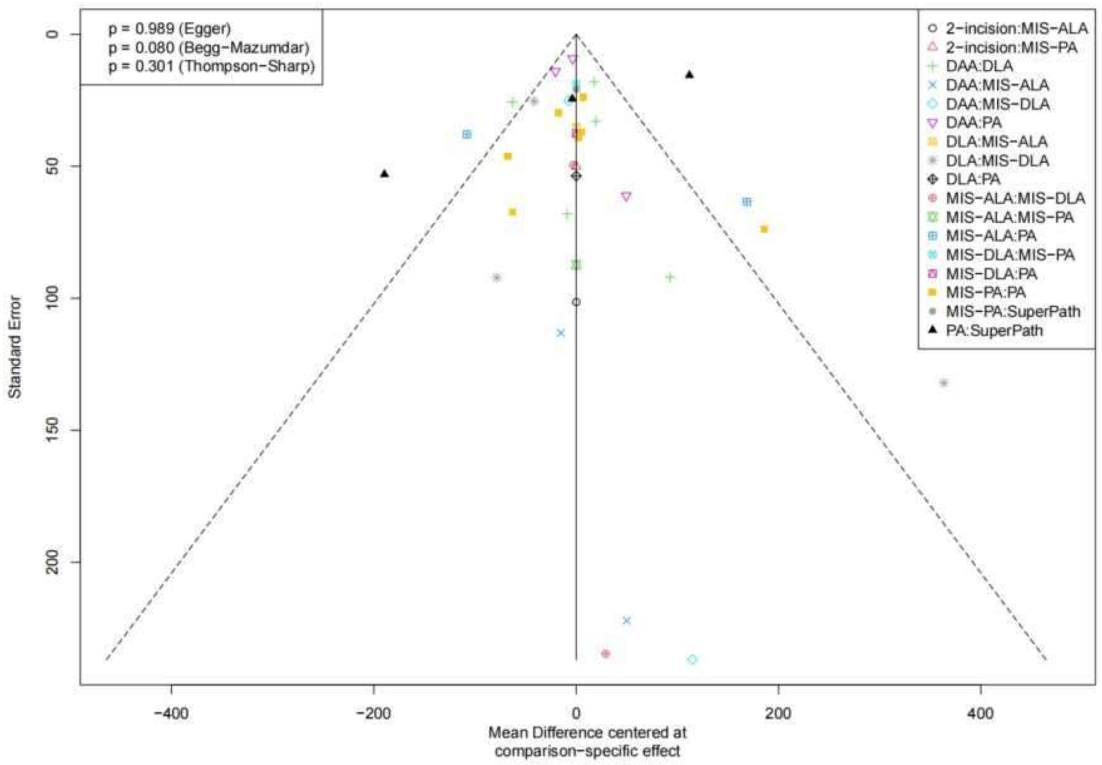

© 2023 Yan L et al. *JAMA Network Open.*

Outcome : Quqlity of life score change

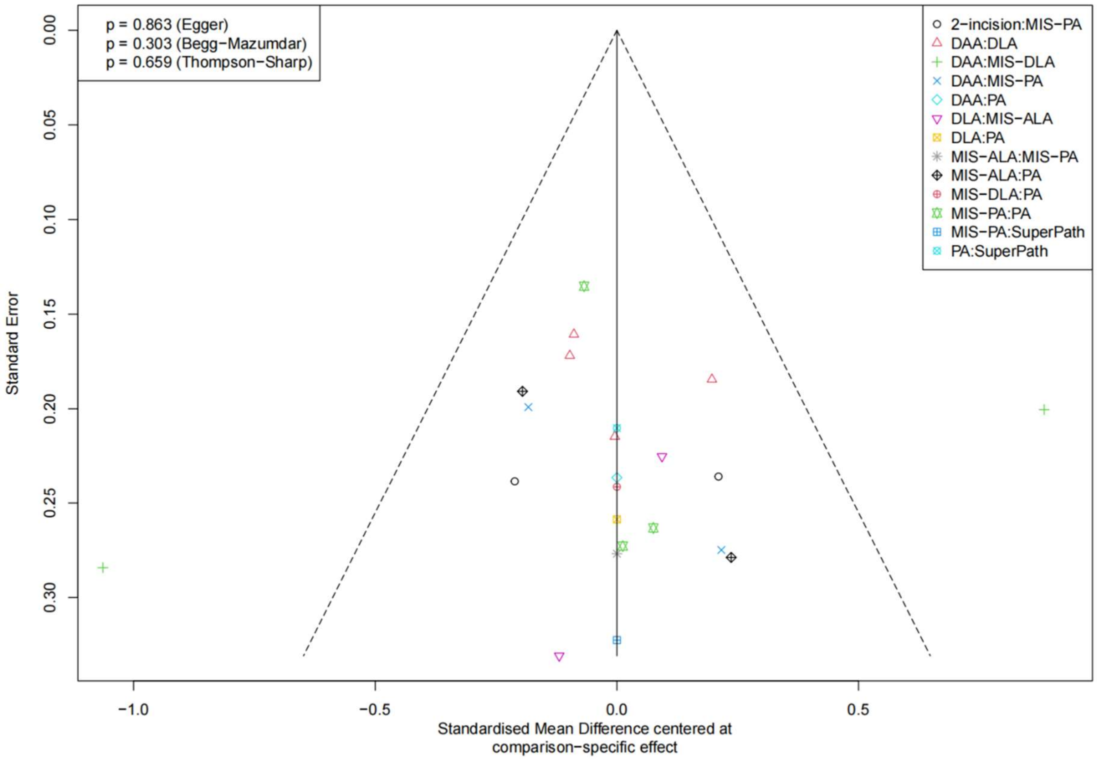

© 2023 Yan L et al. *JAMA Network Open.*

Outcome : Cup Abduction angle

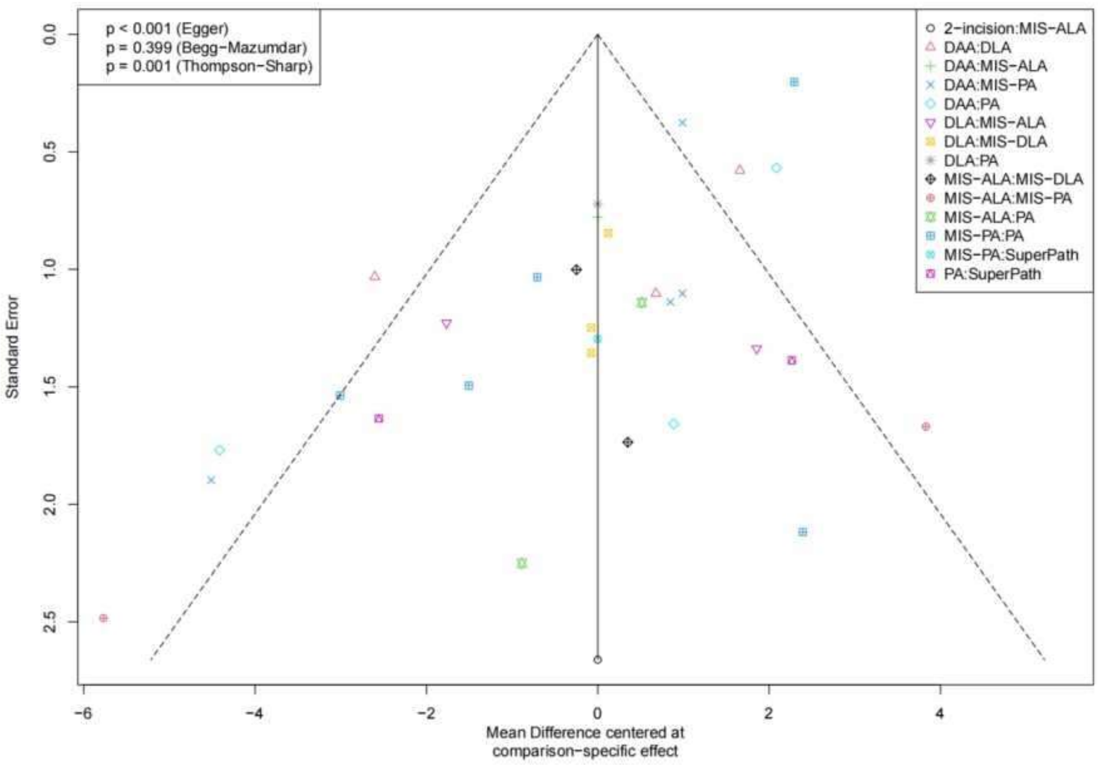

© 2023 Yan L et al. *JAMA Network Open.*

Outcome : Cup Anteversion angle

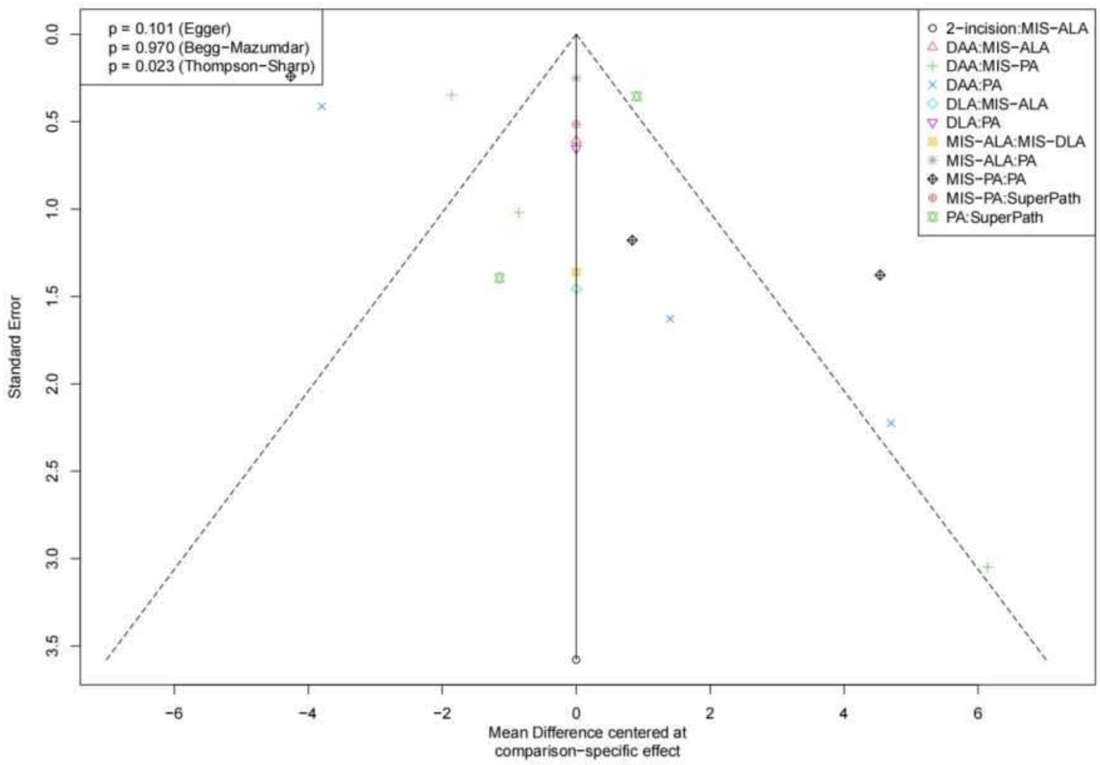

© 2023 Yan L et al. *JAMA Network Open.*

Outcome : Short-term hip score

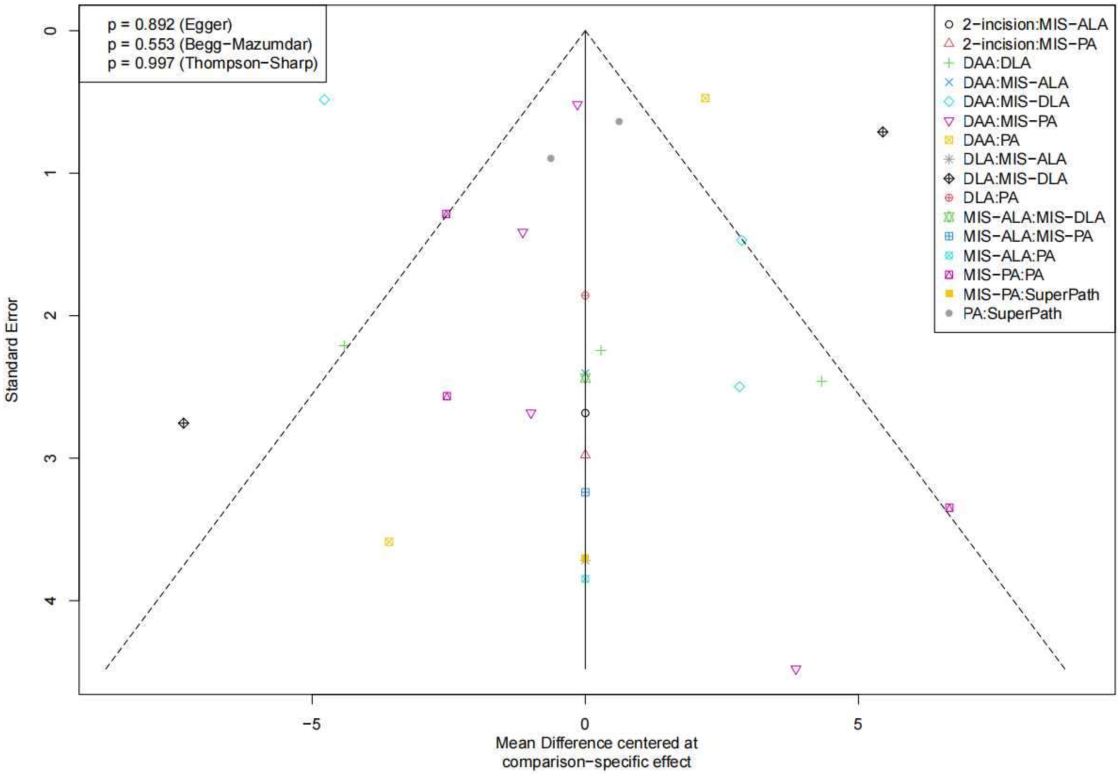

© 2023 Yan L et al. *JAMA Network Open.*

Outcome : Long-term hip score

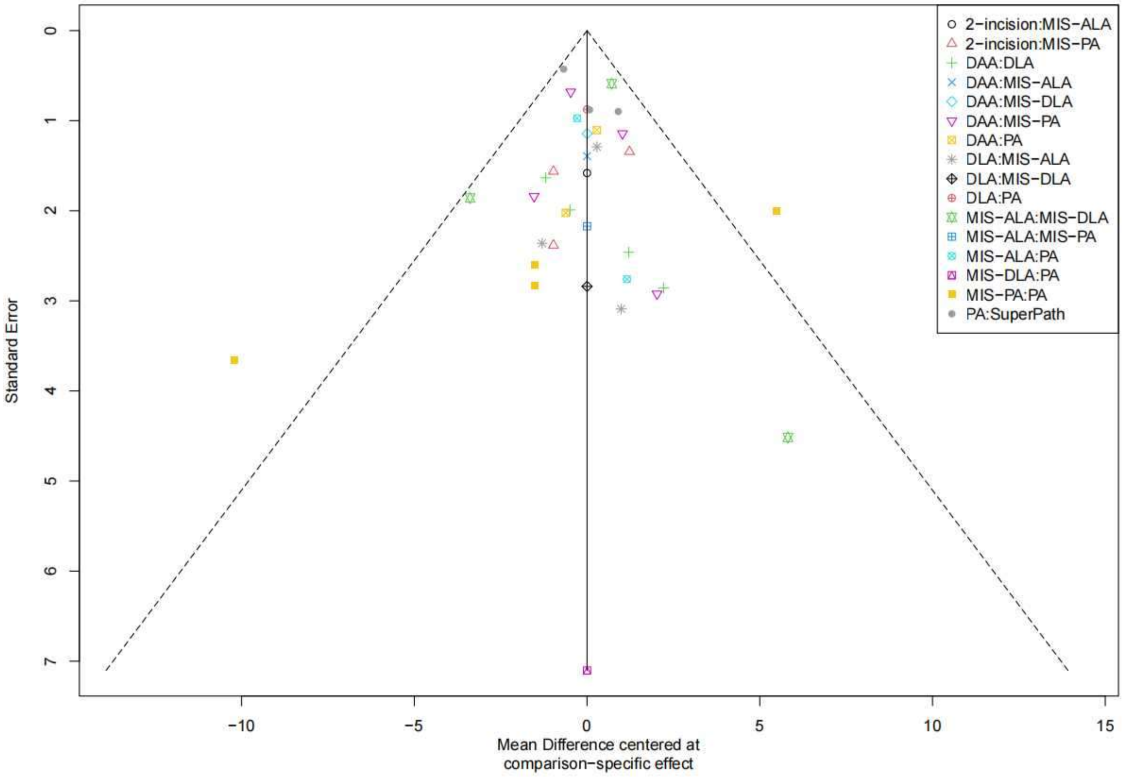

© 2023 Yan L et al. *JAMA Network Open.*

Outcome : Dislocation

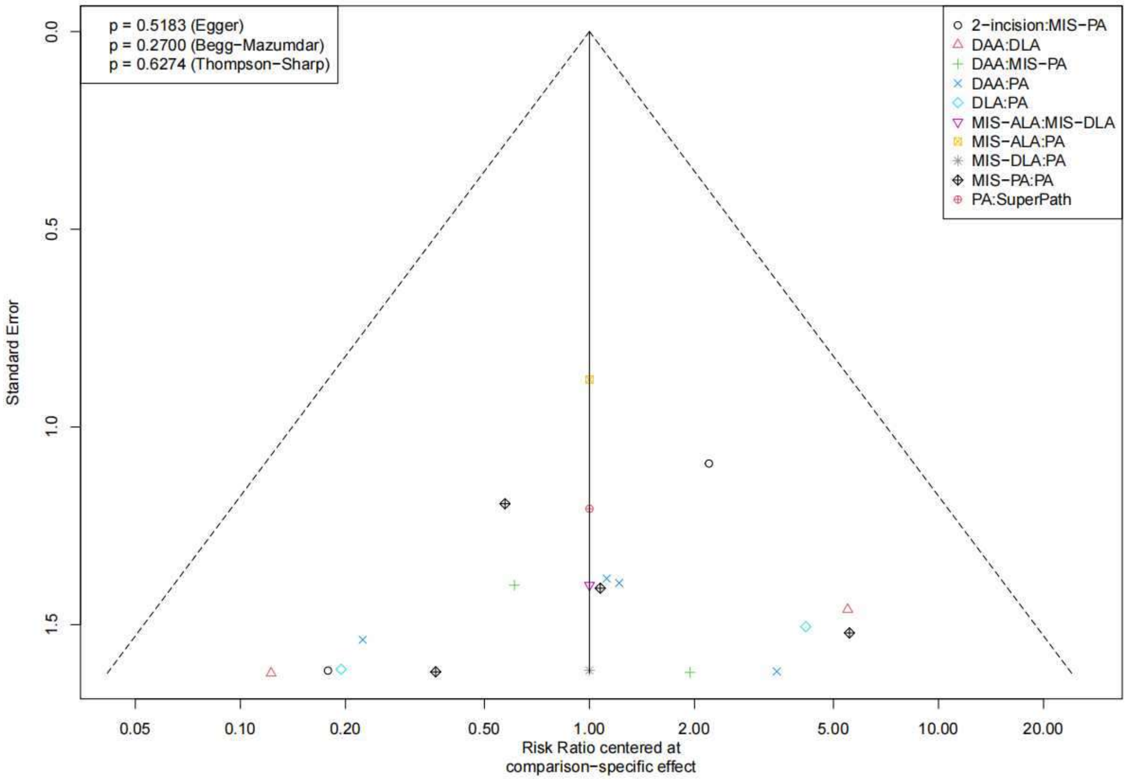

© 2023 Yan L et al. *JAMA Network Open.*

Outcome : Fracture

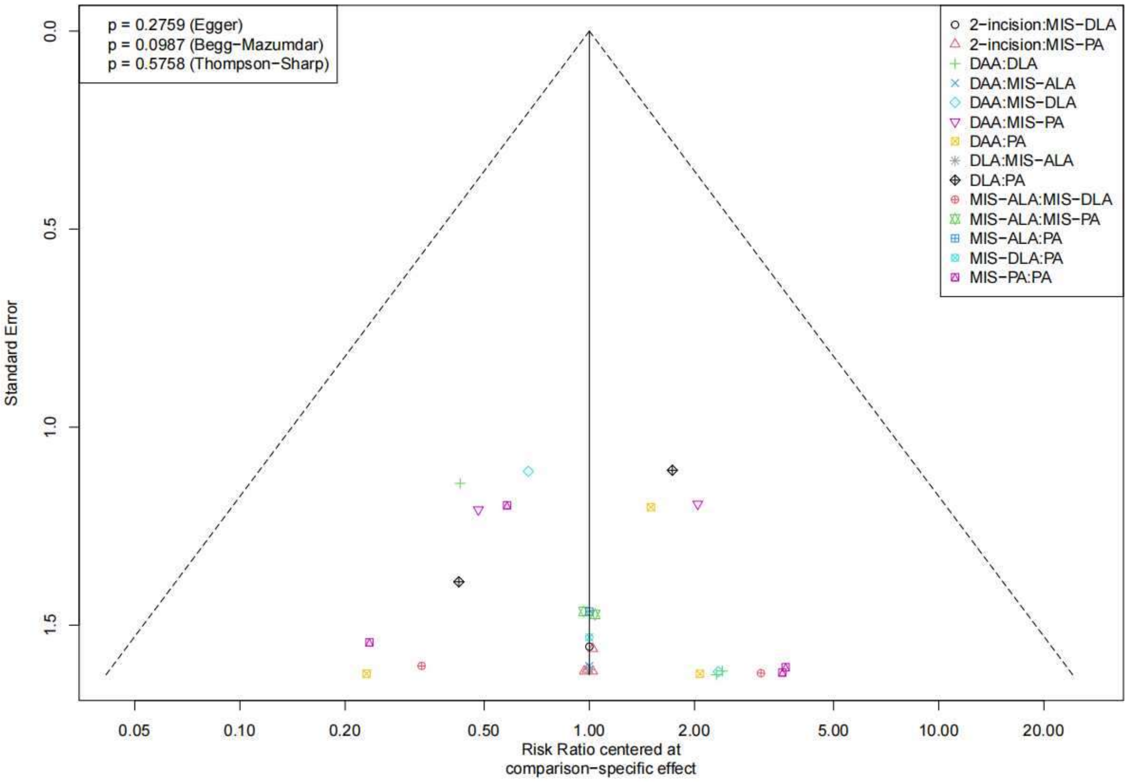

© 2023 Yan L et al. *JAMA Network Open.*

Outcome : Infection

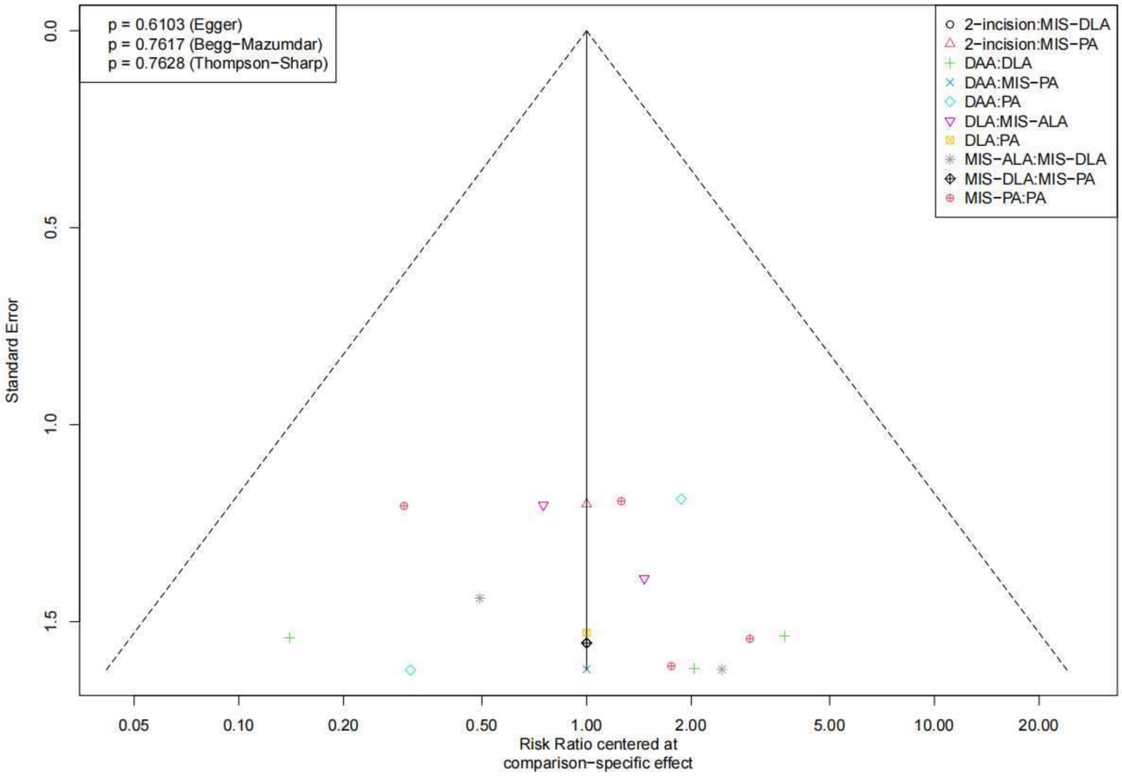

© 2023 Yan L et al. *JAMA Network Open.*

Outcome : Nerve injury

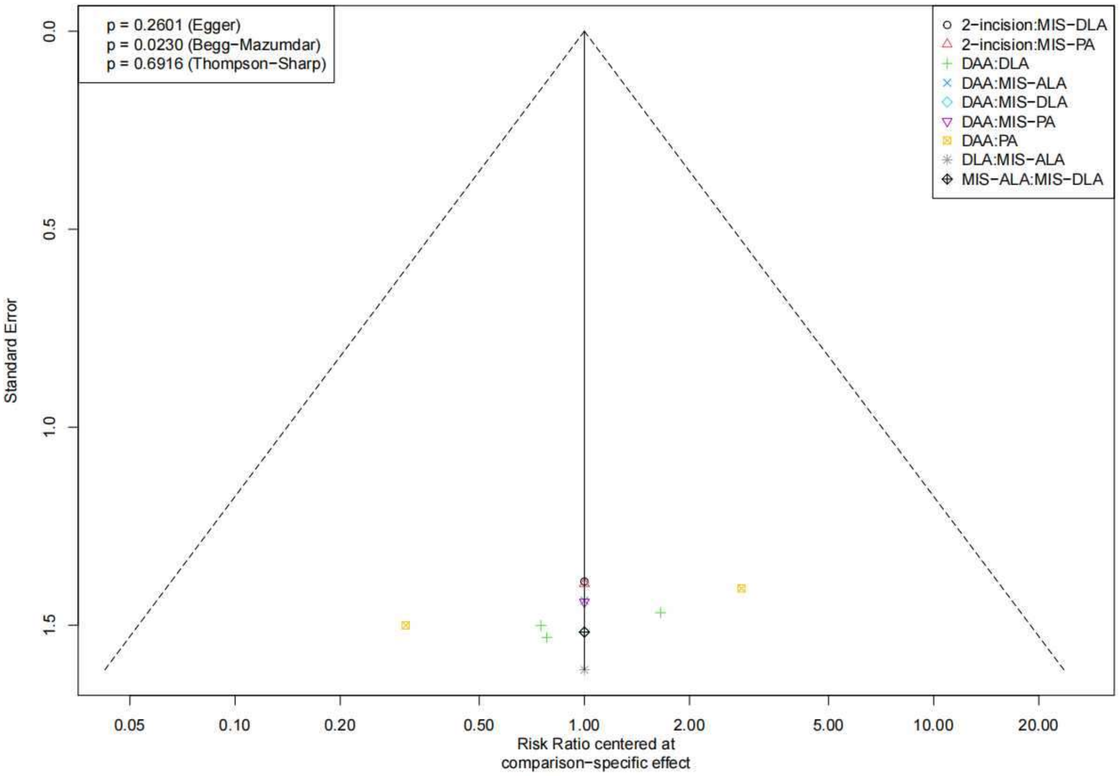

© 2023 Yan L et al. *JAMA Network Open.*

Outcome : Reoperation

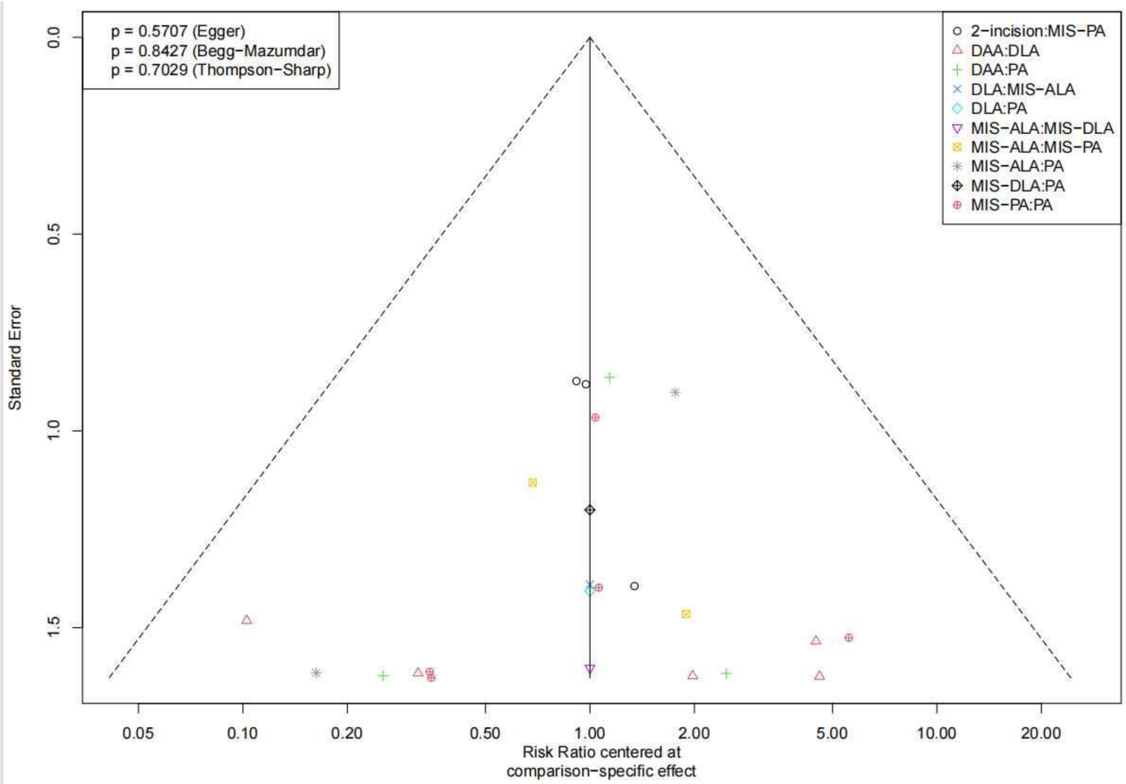

© 2023 Yan L et al. *JAMA Network Open.*

Outcome : Thromboembolism

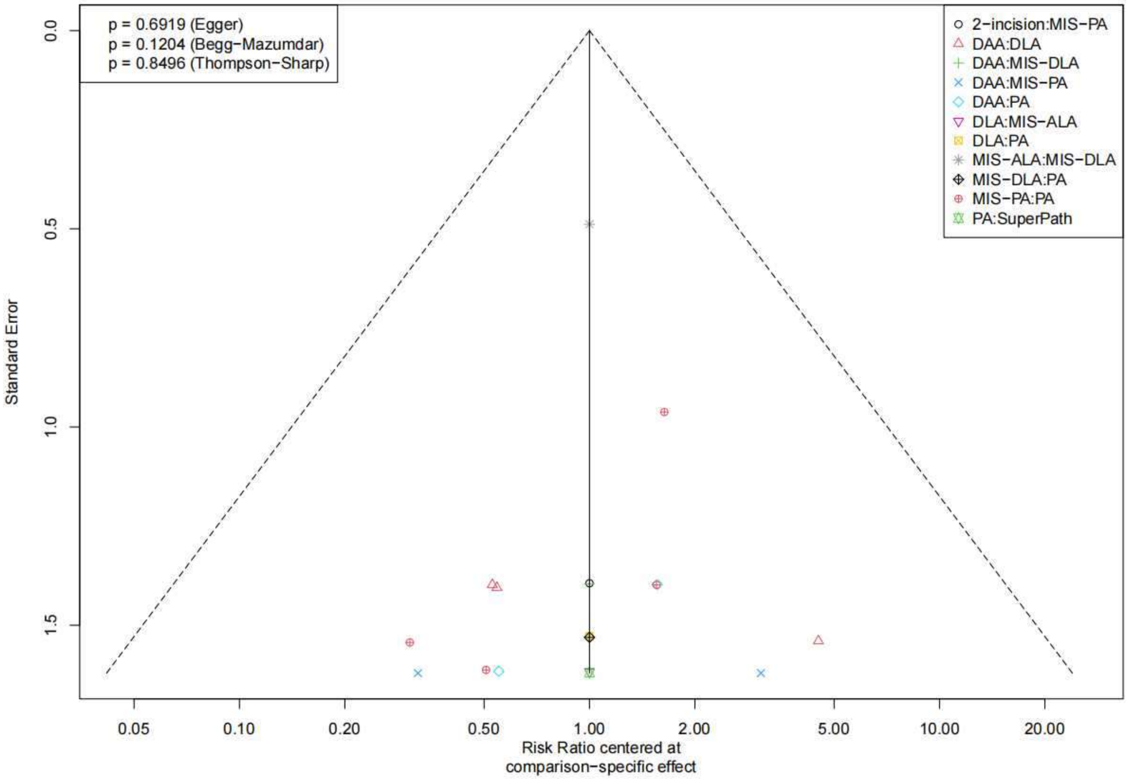

© 2023 Yan L et al. *JAMA Network Open.*

Outcome : Analgesic consumption

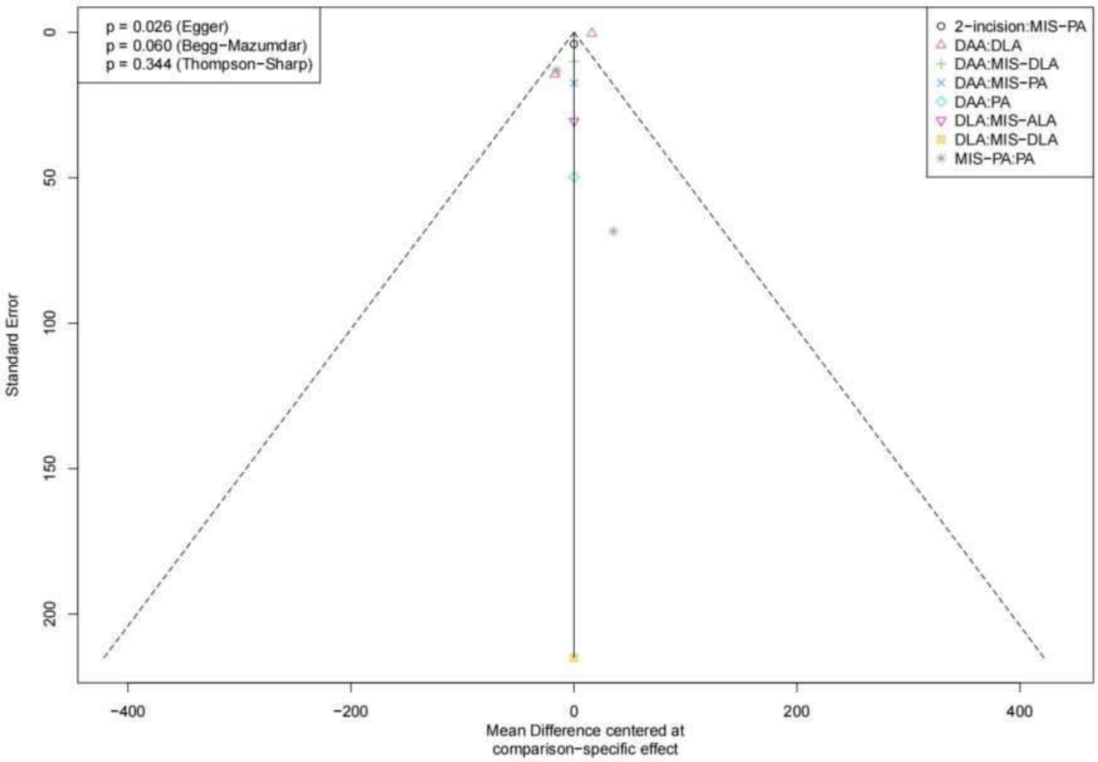

© 2023 Yan L et al. *JAMA Network Open.*

Outcome : Creatine kinase change

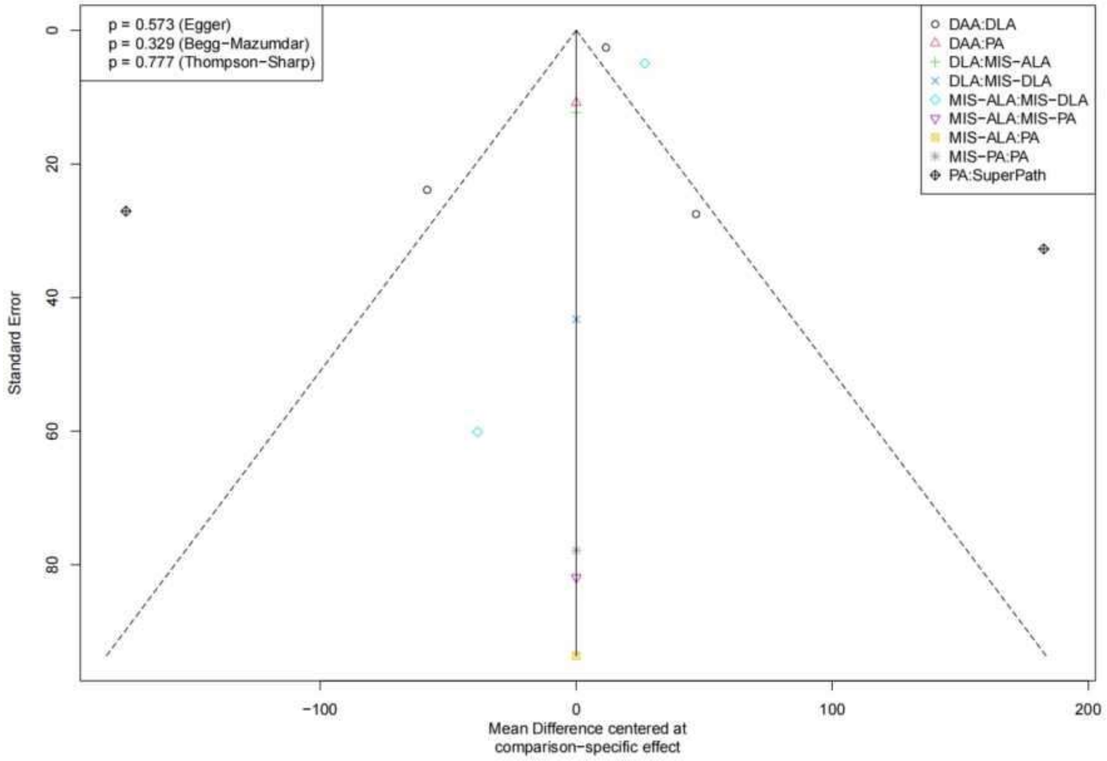

© 2023 Yan L et al. *JAMA Network Open.*

Outcome : C-reactive protein change

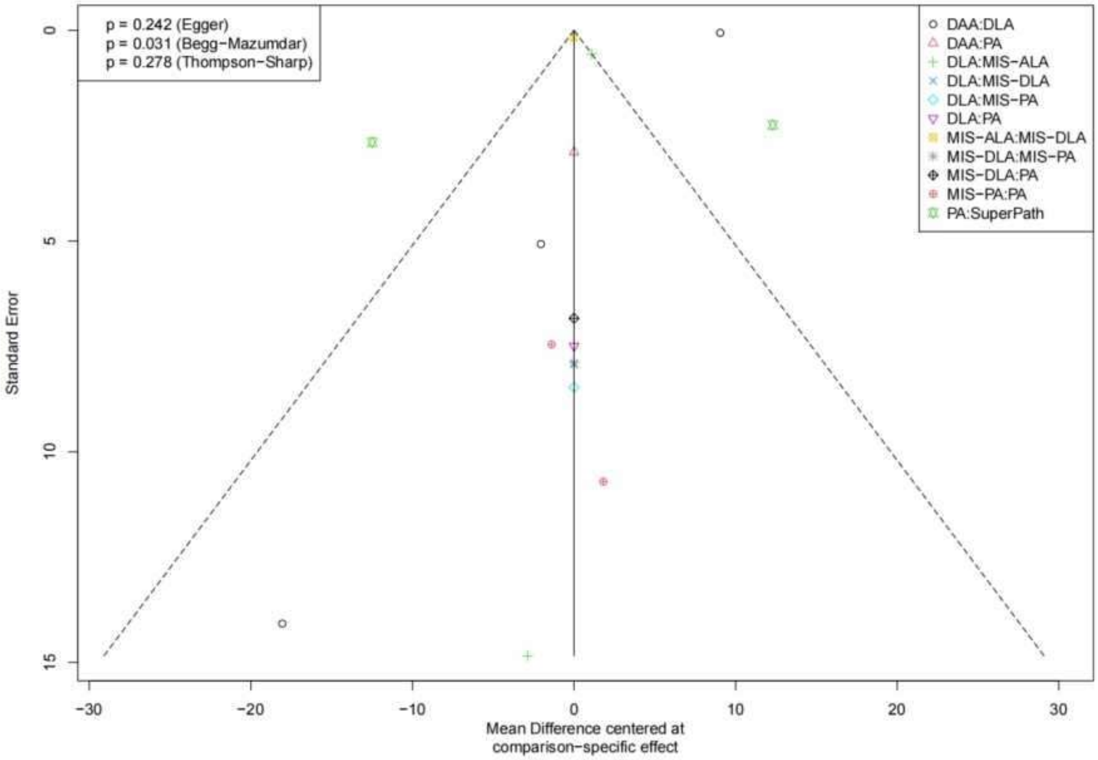

© 2023 Yan L et al. *JAMA Network Open.*

Outcome : Hemoglobin change

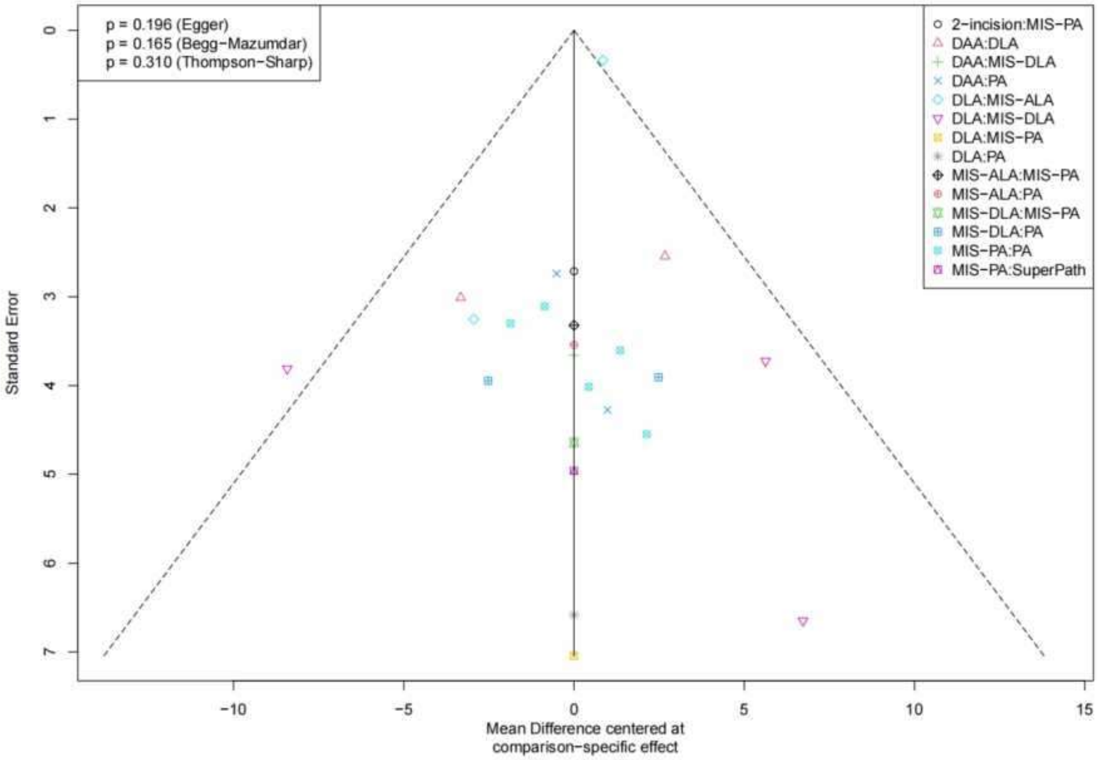

© 2023 Yan L et al. *JAMA Network Open.*

Outcome : Interleukin-6 change

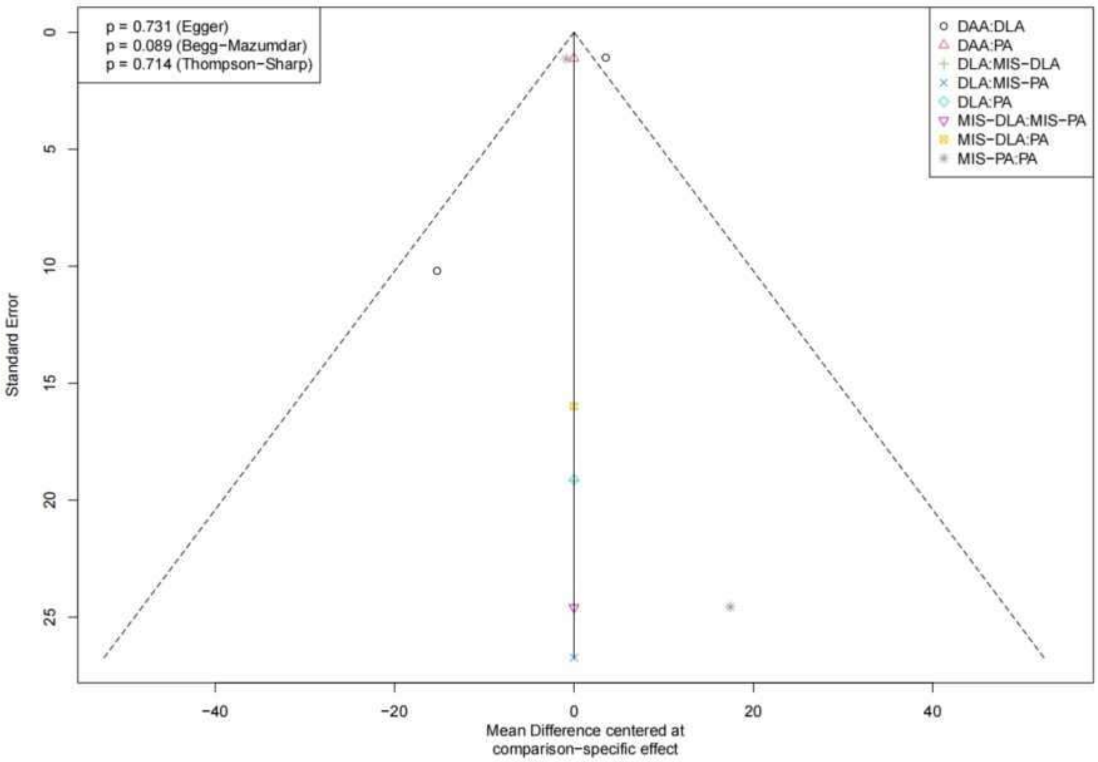

© 2023 Yan L et al. *JAMA Network Open.*

Outcome : Stem alignment

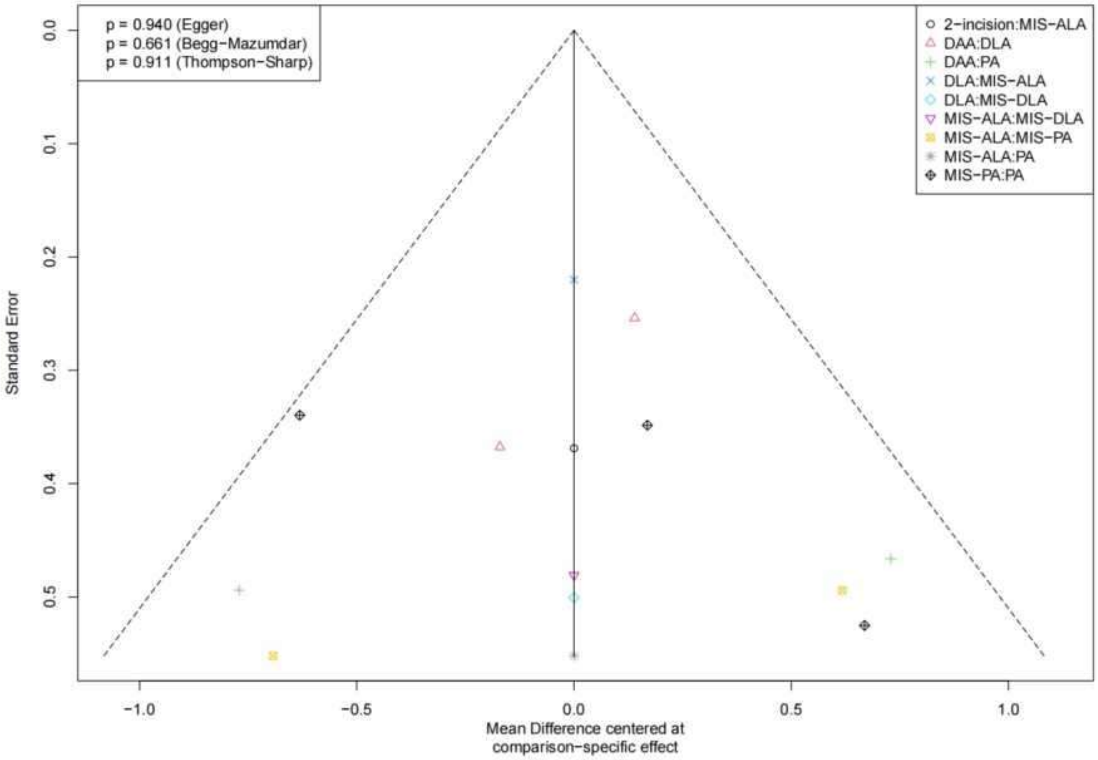

© 2023 Yan L et al. *JAMA Network Open.*

Outcome : Step length change

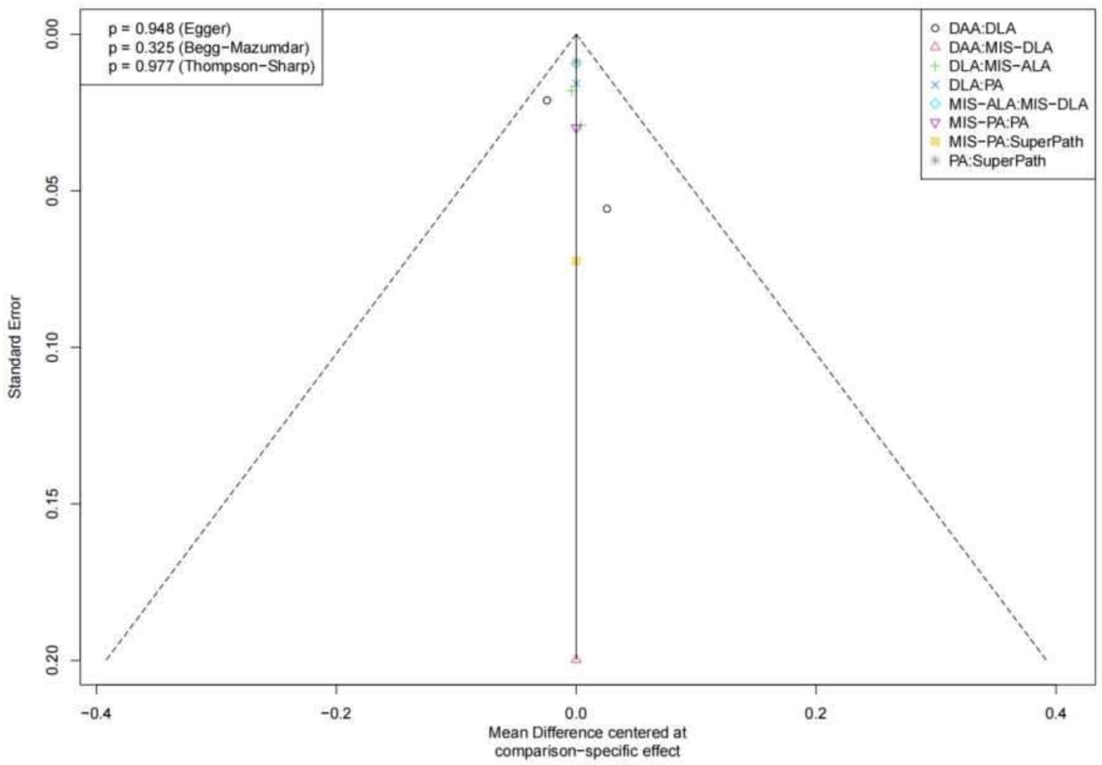

Outcome : Volume of blood transfusion change

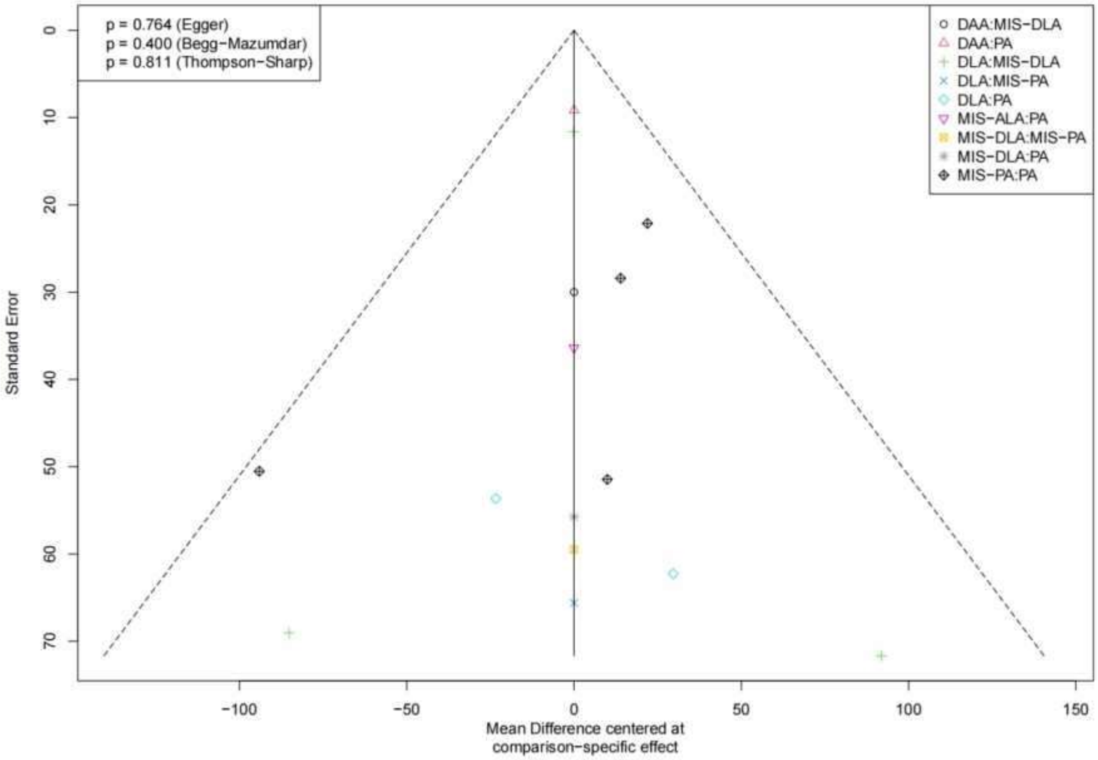

© 2023 Yan L et al. *JAMA Network Open.*

Outcome : Walking speed change

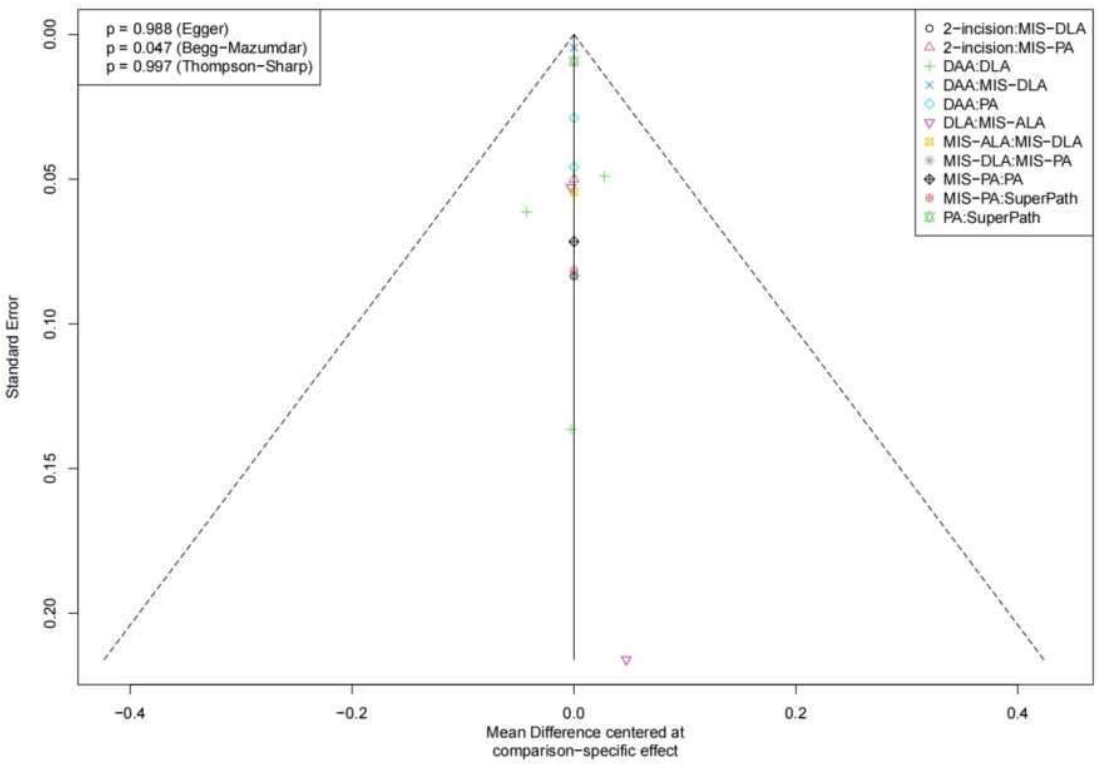

© 2023 Yan L et al. *JAMA Network Open.*

eFigure 8. Incidence Rate (Sample Size) of 6 Complication Types

DAA=direct anterior approach. DLA=direct lateral approach. MIS-DLA=minimally invasive direct lateral approach. MIS-ALA=minimally invasive anterolateral approach. PA=posterior approach. MIS-PA=minimally invasive posterior approach. SuperPath=supercapsular percutaneously assisted total hip arthroplasty

	Dislocation	Fracture	Infection	Nerve injury	Reoperation	Thromboembolism
2-incision	5.63(71)	3.64(110)	2.33(43)	62.50(8)	4.95(101)	2.86(35)
DAA	2.65(377)	2.41(457)	1.64(304)	15.00(380)	1.99(403)	1.53(393)
DLA	1.83(219)	2.23(358)	2.25(267)	0.52(193)	1.67(418)	6.18(275)
MIS-ALA	4.76(84)	6.45(155)	2.13(141)	0.00(78)	6.38(141)	3.33(90)
MIS-DLA	1.15(87)	0.64(156)	9.33(75)	7.41(27)	1.82(55)	1.55(129)
MIS-PA	1.88(372)	1.30(460)	2.97(303)	0.00(58)	3.42(380)	3.78(344)
PA	2.30(610)	1.80(556)	1.54(324)	0.00(96)	2.27(528)	0.25(403)
SuperPath	2.17(46)	------	------	------	------	0.00(46)

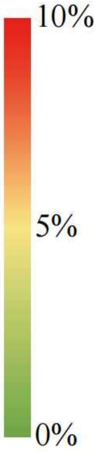

© 2023 Yan L et al. *JAMA Network Open.*

eAppendix 1. Search Strategy

Pubmed
#1 randomized controlled trial [pt]
#2 controlled clinical trial [pt]
#3 randomized [tiab]
#4 placebo [tiab]
#5 drug therapy [sh]
#6 randomly [tiab]
#7 trial [tiab]
#8 groups [tiab]
#9 #1 OR #2 OR #3 OR #4 OR #5 OR #6 OR #7 OR #8
#10 animals [mh] NOT humans [mh]
#11 #9 NOT #10
#12 Arthroplasty, Replacement, Hip [mh]
#13 Arthroplasties, Replacement, Hip [tiab]
#14 Arthroplasty, Hip Replacement [tiab]
#15 Hip Prosthesis Implantation [tiab]
#16 Hip Prosthesis Implantations [tiab]
#17 Implantation, Hip Prosthesis [tiab]
#18 Implantations, Hip Prosthesis [tiab]
#19 Prosthesis Implantation, Hip [tiab]
#20 Prosthesis Implantations, Hip [tiab]
#21 Hip Replacement Arthroplasty [tiab]
#22 Replacement Arthroplasties, Hip [tiab]
#23 Replacement Arthroplasty, Hip [tiab]
#24 Arthroplasties, Hip Replacement [tiab]
#25 Hip Replacement Arthroplasties [tiab]
#26 Hip Replacement, Total [tiab]
#27 Replacement, Total Hip [tiab]
#28 Hip Replacements, Total [tiab]
#29 Replacements, Total Hip [tiab]
#30 Total Hip Replacements [tiab]
#31 Total Hip Replacement [tiab]
#32 #12 OR #13 OR #14 OR #15 OR #16 OR #17 OR #18 OR #19 OR #20 OR #21 OR #22 OR #23 OR #24 OR #25 OR #26 OR #27 OR #28 OR #29 OR #30 OR #31
#33 #11 AND #32
#34 approach [tiab]
#35 approaches [tiab]
#36 #34 OR #35
#37 #33 AND #36

© 2023 Yan L et al. *JAMA Network Open.*

EMBASE

#1 'clinical trial'/de OR 'randomized controlled trial'/de OR 'randomization'/de OR 'single blind procedure'/de OR 'double blind procedure'/de OR 'crossover procedure'/de OR 'placebo'/de OR 'prospective study'/de OR 'randomi?ed controlled' NEXT/1 trial* OR rct OR 'randomly allocated' OR 'allocated randomly' OR 'random allocation' OR allocated NEAR/2 random OR single NEXT/1 blind* OR double NEXT/1 blind* OR (treble OR triple) NEAR/1 blind* OR placebo*

#2 'Arthroplasties, Replacement, Hip':ab,ti

#3 'Arthroplasty, Hip Replacement':ab,ti

#4 'Hip Prosthesis Implantation':ab,ti

#5 'Hip Prosthesis Implantations':ab,ti

#6 'Implantation, Hip Prosthesis':ab,ti

#7 'Implantations, Hip Prosthesis':ab,ti

#8 'Prosthesis Implantation, Hip':ab,ti

#9 'Prosthesis Implantations, Hip':ab,ti

#10 'Hip Replacement Arthroplasty':ab,ti

#11 'Replacement Arthroplasties, Hip':ab,ti

#12 'Replacement Arthroplasty, Hip':ab,ti

#13 'Arthroplasties, Hip Replacement':ab,ti

#14 'Hip Replacement Arthroplasties':ab,ti

#15 'Hip Replacement, Total':ab,ti

#16 'Replacement, Total Hip':ab,ti

#17 'Hip Replacements, Total':ab,ti

#18 'Replacements, Total Hip':ab,ti

#19 'Total Hip Replacements':ab,ti

#20 'Total Hip Replacement':ab,ti

#21 'Arthroplasty, Replacement, Hip':ab,ti

#22 #2 OR #3 OR #4 OR #5 OR #6 OR #7 OR #8 OR #9 OR #10 OR #11 OR #12 OR #13 OR #14 OR #15 OR #16 OR #17 OR #18 OR #19 OR #20 OR #21

#23 #1 AND #22

#24 approach:ab,ti

#25 approaches:ab,ti

#26 #24 OR #25

#27 #23 AND #26

© 2023 Yan L et al. *JAMA Network Open.*

Cochrane
#1 (Arthroplasty, Replacement, Hip):ti,ab,kw
#2 (Arthroplasties, Replacement, Hip):ti,ab,kw
#3 (Arthroplasty, Hip Replacement):ti,ab,kw
#4 (Hip Prosthesis Implantation):ti,ab,kw
#5 (Hip Prosthesis Implantations):ti,ab,kw
#6 (Implantation, Hip Prosthesis):ti,ab,kw
#7 (Implantations, Hip Prosthesis):ti,ab,kw
#8 (Prosthesis Implantation, Hip):ti,ab,kw
#9 (Prosthesis Implantations, Hip):ti,ab,kw
#10 (Hip Replacement Arthroplasty):ti,ab,kw
#11 (Replacement Arthroplasties, Hip):ti,ab,kw
#12 (Replacement Arthroplasty, Hip):ti,ab,kw
#13 (Arthroplasties, Hip Replacement):ti,ab,kw
#14 (Hip Replacement Arthroplasties):ti,ab,kw
#15 (Hip Replacement, Total):ti,ab,kw
#16 (Replacement, Total Hip):ti,ab,kw
#17 (Hip Replacements, Total):ti,ab,kw
#18 (Replacements, Total Hip):ti,ab,kw
#19 (Total Hip Replacements):ti,ab,kw
#20 (Total Hip Replacement):ti,ab,kw
#21 #1 OR #2 OR #3 OR #4 OR #5 OR #6 OR #7 OR #8 OR #9 OR #10 OR #11 OR #12 OR #13 OR #14 OR #15 OR #16 OR #17 OR #18 OR #19 OR #20
#22 approach:ti,ab,kw
#23 approaches:ti,ab,kw
#24 #22 OR #23
#25 #21 AND #24

© 2023 Yan L et al. *JAMA Network Open.*

ClinicalTrials.gov

approach OR approaches | "Arthroplasty, Replacement, Hip" OR "Arthroplasties, Replacement, Hip" OR "THA" OR "Arthroplasty, Hip Replacement" OR "Hip Prosthesis Implantation" OR "Hip Prosthesis Implantations" OR "Implantation, Hip Prosthesis" OR "Implantations, Hip Prosthesis" OR "Prosthesis Implantation, Hip" OR "Prosthesis Implantations, Hip" OR "Hip Replacement Arthroplasty" OR "Replacement Arthroplasties, Hip" OR "Replacement Arthroplasty, Hip" OR "Arthroplasties, Hip Replacement" OR "Hip Replacement Arthroplasties" OR "Hip Replacement, Total" OR "Replacement, Total Hip" OR "Hip Replacements, Total" OR "Replacements, Total Hip" OR "" OR "" OR "" OR "" OR "" OR "" OR "" OR "" OR "" OR "Total Hip Replacements" OR "Total Hip Replacement"

© 2023 Yan L et al. *JAMA Network Open.*

eAppendix 2. Supplementary Methods

eAppendix 2A. Methods for imputation of missing standard deviation

We used published standard deviations (SDs), where available. When standard errors instead of SDs were presented, the former was converted to SDs.1 If both were missing, we estimated SDs from P values or confidence interval (CIs) according to the recommendations of the Cochrane Handbook for Systematic Reviews.1 We also estimated SDs from graphs when they were missing in tables or in text. If studies reported medians and interquartile ranges (IQRs), we used median to impute the missing mean and calculated SDs by dividing IQRs by 1.35.1 We also estimated SDs from the formula range/4.2 If none of these options are viable, we imputed the missing SDs using pooled SDs from other studies included in our NMA following the formula below:3

$$SD_{pooled} = \sqrt{\frac{\sum(n_i - 1)SD_i^2}{\sum(n_i - 1)}}$$

Higgins JPT, Deeks JJ (editors). Chapter 7: Selecting studies and collecting data. In: Higgins JPT, Green S (editors), Cochrane Handbook for Systematic Reviews of Interventions. Version 5.1.0 [updated March 2011]. The Cochrane Collaboration, 2011. Available from www.cochranehandbook.org.

Hozo, Stela Pudar et al. "Estimating the mean and variance from the median, range, and the size of a sample." BMC medical research methodology vol. 5 13. 20 Apr. 2005, doi:10.1186/1471-2288-5-13

Furukawa T A, Barbui C, Cipriani A, et al. Imputing missing standard deviations in metaanalyses can provide accurate results. Journal of clinical epidemiology, 2006, 59(1): 7-10.

© 2023 Yan L et al. *JAMA Network Open.*

eAppendix 2B. Details of the sensitivity analyses

Multiple sensitivity analyses were carried out to assess the robustness of the final results, including:

1. Exclusion of studies with high item in the ROB
2. Exclusion of studies with fewer than 50 participants
3. Exclusion of studies with follow-up time <1year
4. League table with fixed-effect model
5. Included studies where all surgeries were carried out by a single surgeon
6. Included studies where osteoarthritis serves as the only reason for THA
7. Included studies where both the femoral stem prosthesis and the acetabular prosthesis were non-cemented fixations
8. Inclusion of studies that state all procedures were unilateral
9. Exclusion of studies without standard deviation
10. Included studies where all surgerns were experienced.
11. Inclusion of studies in which all patients underwent spinal anesthesia

eAppendix 2C. Details of the publication bias assessments

Comparison-adjusted funnel plots were used to explore publication bias for all direct comparisons with treatments ordered by P-scores. Egger's regression test, Begg's rank test and Thompson−Sharpl' test were used to assess asymmetry.

eAppendix 2D. Change from protocol

We initially planned a Bayesian analysis but during conduct of the study identified innovative methods for rating evidence quality that require a frequentist analysis; we therefore changed our analytic approach. Besides, due to our increasing understanding of the re-themes at a later stage, we added meta-regression analysis, sensitivity analysis and GRADE ratings.

© 2023 Yan L et al. *JAMA Network Open.*

eAppendix 2E. Instructions for GRADE assessment

Our certainty of assessments addressed the following categories: risk of bias, imprecision, inconsistency, and indirectness. Due to the limited number of direct comparison (less than 10), we did not assess the publication bias. For both direct and indirect comparisons, the starting point for certainty in estimates was 'high'. The certainty in indirect estimates was inferred from examination of the dominant lowest order loop. We identified the dominant lowest order loop by per comparison contribution matrix which could show the contribution percentage of each direct comparison to each indirect comparison. The certainty rating chosen was the lowest of the direct estimates contributing to the indirect comparison. For instance, consider a comparison of A versus B that is informed by A versus C and B versus C. If A versus C was rated as high certainty and B versus C as moderate certainty, the overall indirect certainty rating was moderate (moderate from the B versus C comparison). We considered further rating down each indirect comparison for intransitivity if the interventions or populations were dissimilar between the direct comparisons informing the loop that contributed most to the indirect estimate.

The certainty rating of network estimate was the highest of the direct estimates and indirect estimates contributing to the network estimates. We considered rating down the certainty in the network estimate if there was incoherence between the indirect and direct estimates or if there was imprecision (credible interval pass through the invalid line) around the treatment effect.

Instruction for each domain:

Risk of Bias: We classified an overall risk of bias for every study based on the individual risk of bias items. A study is at high risk of bias if one item of ROB is high or if there are more than 2 items that are unclear. For each direct estimate, we rated down for risk of bias if studies with high risk of bias or had contributed more to the overall effect estimate. If half the studies were at high risk and half at low risk, we assigned risk of bias based on the total number of patients randomized within each risk of bias category. For example, if 2 studies are high risk with a total of 400 patients, while 2 studies are at low risk with a total of 500 patients, the risk of bias was low.

Inconsistency/heterogeneity: We assessed the inconsistency for each direct estimate by visually inspecting the distribution of point estimates and corresponding 95% credible intervals. We looked to see if the point estimates were in the same direction, similar in magnitude and we assessed if the estimates all fell within the widest 95% credible intervals.

Publication bias: not detectable.

Indirectness: We detected the inconsistency between studies by half-violin plot, and no significant indirectness was found.

© 2023 Yan L et al. *JAMA Network Open.*

Imprecision: Among all outcomes, we rated down for imprecision among direct estimates if the credible interval pass through the invalid line.

GRADE certainty in estimates
High certainty: Further research is very unlikely to change our certainty in the estimate of effect;
Moderate certainty: Further research is likely to have an important impact on our certainty in the estimate of effect and may change the estimate;
Low certainty: Further research is very likely to have an important impact on our certainty in the estimate of effect and is likely to change the estimate;
Very low certainty: Any estimate of effect is very uncertain.

References

(1) Guyatt GH, Oxman AD, Vist GE, et al. GRADE: an emerging consensus on rating quality of evidence and strength of recommendations. BMJ (Clinical research ed). 2008;336(7650):924-926.
(2) Puhan MA, Schunemann HJ, Murad MH, et al. A GRADE Working Group approach for rating the quality of treatment effect estimates from network meta-analysis. BMJ (Clinical research ed). 2014;349:g5630.
(3) Brignardello-Petersen R, Bonner A, Alexander PE, et al. Advances in the GRADE approach to rate the certainty in estimates from a network meta-analysis. J Clin Epidemiol. 2018;93:36-44.

© 2023 Yan L et al. *JAMA Network Open.*

eAppendix 3. Reference List of Eligible Studies

1. Meng W, Gao L, Huang Z, et al. Supercapsular percutaneously-assisted total hip (SuperPath) versus mini-incision posterolateral total hip arthroplasty for hip osteoarthritis: a prospective randomized controlled trial. *Annals of translational medicine.* 2021;9(5).
2. Li X, Ma L, Wang Q, Rong K. Comparison of total hip arthroplasty with minimally invasive SuperPath approach vs. conventional posterolateral approach in elderly patients: A one-year follow-up randomized controlled research. *Asian journal of surgery.* 2021;44(3):531-536.
3. Xie J, Zhang H, Wang L, Yao X, Pan Z, Jiang Q. Comparison of supercapsular percutaneously assisted approach total hip versus conventional posterior approach for total hip arthroplasty: a prospective, randomized controlled trial. *Journal of orthopaedic surgery and research.* 2017;12(1):138.
4. Khan RJK, Maor D, Hofmann M, Haebich S. A comparison of a less invasive piriformissparing approach versus the standard posterior approach to the hip: A randomised controlled trial. *Journal of Bone and Joint Surgery - Series B.* 2012;94 B(1):43-50.
5. Roy L, Laflamme GY, Carrier M, Kim PR, Leduc S. A randomised clinical trial comparing minimally invasive surgery to conventional approach for endoprosthesis in elderly patients with hip fractures. *Injury-International Journal of the Care of the Injured.* 2010;41(4):365-369.
6. Ulivi M, Orlandini L, Vitale JA, et al. Direct superior approach versus posterolateral approach in total hip arthroplasty: a randomized controlled trial on early outcomes on gait, risk of fall, clinical and self-reported measurements. *Acta Orthop.* 2021;92(3):274-279.
7. Ogonda L, Wilson R, Archbold P, et al. A minimal-incision technique in total hip arthroplasty does not improve early postoperative outcomes. A prospective, randomized, controlled trial. *The Journal of bone and joint surgery American volume.* 2005;87(4):701-710.
8. Kim Y-H. Comparison of primary total hip arthroplasties performed with a minimally invasive technique or a standard technique - A prospective and randomized study. *Journal of Arthroplasty.* 2006;21(8):1092-1098.
9. Dorr LD, Maheshwari AV, Long WT, Wan Z, Sirianni LE. Early pain relief and function after posterior minimally invasive and conventional total hip arthroplasty - A prospective, randomized, blinded study. *Journal of Bone and Joint Surgery-American Volume.* 2007;89A(6):1153-1160.
10. Chimento GF, Pavone V, Sharrock N, Kahn B, Cahill J, Sculco TP. Minimally invasive

total hip arthroplasty - A prospective randomized study. *Journal of Arthroplasty.* 2005;20(2):139-144.

11. Catma FM, Ozturk A, Unlu S, Ersan O, Altay M. Posterior hip approach yields better functional results vis-a-vis anterolateral approach in total hip arthroplasty for patients with severe hip dysplasia: a prospective randomized controlled clinical study. *Journal of orthopaedic surgery (Hong Kong).* 2017;25(2):2309499017717179.
12. Ji HM, Kim KC, Lee YK, Ha YC, Koo KH. Dislocation after total hip arthroplasty: a randomized clinical trial of a posterior approach and a modified lateral approach. *J Arthroplasty.* 2012;27(3):378-385.
13. Witzleb WC, Stephan L, Krummenauer F, Neuke A, Gunther KP. Short-term outcome after posterior versus lateral surgical approach for total hip arthroplasty - A randomized clinical trial. *European journal of medical research.* 2009;14(6):256-263.
14. Tan BKL, Khan RJK, Haebich SJ, Maor D, Blake EL, Breidahl WH. Piriformis-Sparing Minimally Invasive Versus the Standard Posterior Approach for Total Hip Arthroplasty: A 10-Year Follow-Up of a Randomized Control Trial. *J Arthroplasty.* 2019;34(2):319-326.
15. Korytkin AA, El Moudni YM, Novikova YS, Kovaldov KA, Morozova EA. A prospective randomised comparison of earlier function after total hip arthroplasty with a mini posterior approach or supercapsular percutaneously-assisted total hip approach: a gait analysis study. *Hip international.* 2021.
16. Wang T, Shao L, Xu W, Chen H, Huang W. Comparison of morphological changes of gluteus medius and abductor strength for total hip arthroplasty via posterior and modified direct lateral approaches. *International orthopaedics.* 2019.
17. Pagnano MW, Trousdale RT, Meneghini RM, Hanssen AD. Slower Recovery After Two-Incision Than Mini-Posterior-Incision Total Hip Arthroplasty. *Journal of Bone and Joint Surgery-American Volume.* 2009;91A:50-73.
18. Rosenlund S, Broeng L, Holsgaard-Larsen A, Jensen C, Overgaard S. Patient-reported outcome after total hip arthroplasty: comparison between lateral and posterior approach. *Acta Orthop.* 2017;88(3):239-247.
19. Yang C, Zhu Q, Han Y, et al. Minimally-invasive total hip arthroplasty will improve early postoperative outcomes: a prospective, randomized, controlled trial. *Irish journal of medical science.* 2009;179(2):285-290.
20. Laffosse JM, Accadbled F, Molinier F, Chiron P, Hocine B, Puget J. Anterolateral mini-invasive versus posterior mini-invasive approach for primary total hip replacement. Comparison of exposure and implant positioning. *Archives of Orthopaedic and Trauma Surgery.* 2008;128(4):363-369.
21. Müller M, Schwachmeyer V, Tohtz S, et al. The direct lateral approach: impact on gait patterns, foot progression angle and pain in comparison with a minimally invasive anterolateral approach. *Archives of orthopaedic and trauma surgery.* 2012;132(5):725-731.
22. Muller M, Tohtz S, Springer I, Dewey M, Perka C. Randomized controlled trial of abductor muscle damage in relation to the surgical approach for primary total hip

© 2023 Yan L et al. *JAMA Network Open.*

replacement: minimally invasive anterolateral versus modified direct lateral approach. *Arch Orthop Trauma Surg.* 2011;131(2):179-189.

23. Matziolis D, Wassilew G, Strube P, Matziolis G, Perka C. Differences in muscle trauma quantifiable in the laboratory between the minimally invasive anterolateral and transgluteal approach. *Archives of orthopaedic and trauma surgery.* 2011;131(5):651-655.
24. Inaba Y, Kobayashi N, Yukizawa Y, Ishida T, Iwamoto N, Saito T. Little clinical advantage of modified Watson-Jones approach over modified mini-incision direct lateral approach in primary total hip arthroplasty. *J Arthroplasty.* 2011;26(7):1117-1122.
25. Schwarze M, Budde S, von Lewinski G, et al. No effect of conventional vs. minimally invasive surgical approach on clinical outcome and migration of a short stem total hip prosthesis at 2-year follow-up: A randomized controlled study. *Clinical biomechanics (Bristol, Avon).* 2018;51:105-112.
26. Shitama T, Kiyama T, Naito M, Shiramizu K, Huang G. Which is more invasive-mini versus standard incisions in total hip arthroplasty? *International orthopaedics.* 2009;33(6):1543-1547.
27. Varela-Egocheaga JR, Suarez-Suarez MA, Fernandez-Villan M, Gonzalez-Sastre V, Varela-Gomez JR, Murcia-Mazon A. Minimally invasive hip surgery: the approach did not make the difference. *European journal of orthopaedic surgery & traumatology : orthopedie traumatologie.* 2013;23(1):47-52.
28. Speranza A, Iorio R, Ferretti M, D'Arrigo C, Ferretti A. A lateral minimal-incision technique in total hip replacement: a prospective, randomizes, controlled trial. *Hip international : the journal of clinical and experimental research on hip pathology and therapy.* 2007;17(1):4-8.
29. Mazoochian F, Weber P, Schramm S, Utzschneider S, Fottner A, Jansson V. Minimally invasive total hip arthroplasty: a randomized controlled prospective trial. *Archives of Orthopaedic and Trauma Surgery.* 2009;129(12):1633-1639.
30. Dutka J, Sosin P, Libura M, Skowronek P. Total hip arthroplasty through a minimally invasive lateral approach--our experience and early results. *Ortopedia, traumatologia, rehabilitacja.* 2007;9(1):39-45.
31. Vasilakis I, Solomou E, Vitsas V, Fennema P, Korovessis P, Siamblis DK. Correlative analysis of MRI-evident abductor hip muscle degeneration and power after minimally invasive versus conventional unilateral cementless THA. *Orthopedics.* 2012;35(12):e1684-1691.
32. Pospischill M, Kranzl A, Attwenger B, Knahr K. Minimally invasive compared with traditional transgluteal approach for total hip arthroplasty: a comparative gait analysis. *The Journal of bone and joint surgery American volume.* 2010;92(2):328-337.
33. Martin R, Clayson PE, Troussel S, Fraser BP, Docquier P-L. Anterolateral Minimally Invasive Total Hip Arthroplasty A Prospective Randomized Controlled Study With a Follow-Up of 1 Year. *Journal of Arthroplasty.* 2011;26(8):1362-1372.
34. Landgraeber S, Quitmann H, Guth S, et al. A prospective randomized peri- and post-operative comparison of the minimally invasive anterolateral approach versus the

© 2023 Yan L et al. *JAMA Network Open.*

lateral approach. *Orthopedic reviews.* 2013;5(3):e19.
35. Zhao HY, Kang PD, Xia YY, Shi XJ, Nie Y, Pei FX. Comparison of Early Functional Recovery After Total Hip Arthroplasty Using a Direct Anterior or Posterolateral Approach: A Randomized Controlled Trial. *J Arthroplasty.* 2017;32(11):3421-3428.
36. Rykov K, Meys T, Knobben BAS, Sietsma MS, Reininga IHF, ten Have B. MRI Assessment of Muscle Damage After the Posterolateral Versus Direct Anterior Approach for THA (Polada Trial). A Randomized Controlled Trial. *Journal of arthroplasty.* 2021.
37. Christensen CP, Jacobs CA. Comparison of Patient Function during the First Six Weeks after Direct Anterior or Posterior Total Hip Arthroplasty (THA): a Randomized Study. *Journal of arthroplasty.* 2015;30(9 Suppl):94-97.
38. Cheng TE, Wallis JA, Taylor NF, et al. A Prospective Randomized Clinical Trial in Total Hip Arthroplasty-Comparing Early Results Between the Direct Anterior Approach and the Posterior Approach. *Journal of Arthroplasty.* 2017;32(3):883-890.
39. Barrett WP, Turner SE, Murphy JA, Flener JL, Alton TB. Prospective, Randomized Study of Direct Anterior Approach vs Posterolateral Approach Total Hip Arthroplasty: a Concise 5-Year Follow-Up Evaluation. *Journal of arthroplasty.* 2019.
40. Taunton MJ, Mason JB, Odum SM, Springer BD. Direct anterior total hip arthroplasty yields more rapid voluntary cessation of all walking aids: a prospective, randomized clinical trial. *J Arthroplasty.* 2014;29(9 Suppl):169-172.
41. Moerenhout K, Derome P, Laflamme GY, Leduc S, Gaspard HS, Benoit B. Direct anterior versus posterior approach for total hip arthroplasty: a multicentre, prospective, randomized clinical trial. *Canadian journal of surgery Journal canadien de chirurgie.* 2020;63(5):E412-e417.
42. Taunton MJ, Trousdale RT, Sierra RJ, Kaufman K, Pagnano MW. John Charnley Award: Randomized Clinical Trial of Direct Anterior and Miniposterior Approach THA: Which Provides Better Functional Recovery? *Clin Orthop Relat Res.* 2018;476(2):216-229.
43. Bon G, Kacem EB, Lepretre PM, et al. Does the direct anterior approach allow earlier recovery of walking following total hip arthroplasty? A randomized prospective trial using accelerometry. *Orthopaedics & traumatology, surgery & research : OTSR.* 2019;105(3):445-452.
44. D'Arrigo C, Speranza A, Monaco E, Carcangiu A, Ferretti A. Learning curve in tissue sparing total hip replacement: comparison between different approaches. *Journal of orthopaedics and traumatology : official journal of the Italian Society of Orthopaedics and Traumatology.* 2009;10(1):47-54.
45. Zomar BO, Bryant D, Hunter S, Howard JL, Vasarhelyi EM, Lanting BA. A randomised trial comparing spatio-temporal gait parameters after total hip arthroplasty between the direct anterior and direct lateral surgical approaches. *Hip international : the journal of clinical and experimental research on hip pathology and therapy.* 2018;28(5):478-484.
46. Restrepo C, Parvizi J, Pour AE, Hozack WJ. Prospective randomized study of two surgical approaches for total hip arthroplasty. *J Arthroplasty.* 2010;25(5):671-679.e671.
47. Takada R, Jinno T, Miyatake K, et al. Direct anterior versus anterolateral approach in

one-stage supine total hip arthroplasty. Focused on nerve injury: a prospective, randomized, controlled trial. *Journal of orthopaedic science.* 2018;23(5):783-787.
48. Thaler M, Lechner R, Putzer D, et al. Two-year gait analysis controls of the minimally invasive total hip arthroplasty by the direct anterior approach. *Clinical biomechanics (Bristol, Avon).* 2018;58:34-38.
49. Reichert JC, von Rottkay E, Roth F, et al. A prospective randomized comparison of the minimally invasive direct anterior and the transgluteal approach for primary total hip arthroplasty. *BMC musculoskeletal disorders.* 2018;19(1):241.
50. Nistor DV, Caterev S, Bolboacă SD, Cosma D, Lucaciu DOG, Todor A. Transitioning to the direct anterior approach in total hip arthroplasty. Is it a true muscle sparing approach when performed by a low volume hip replacement surgeon? *International orthopaedics.* 2017;41(11):2245-2252.
51. Mjaaland KE, Kivle K, Svenningsen S, Nordsletten L. Do Postoperative Results Differ in a Randomized Trial Between a Direct Anterior and a Direct Lateral Approach in THA? *Clinical orthopaedics and related research.* 2019;477(1):145-155.
52. Mayr E, Nogler M, Benedetti MG, et al. A prospective randomized assessment of earlier functional recovery in THA patients treated by minimally invasive direct anterior approach: a gait analysis study. *Clinical biomechanics (Bristol, Avon).* 2009;24(10):812-818.
53. Dienstknecht T, Luring C, Tingart M, Grifka J, Sendtner E. Total hip arthroplasty through the mini-incision (Micro-hip) approach versus the standard transgluteal (Bauer) approach: a prospective, randomised study. *Journal of orthopaedic surgery (Hong Kong).* 2014;22(2):168-172.
54. De Anta-Diaz B, Serralta-Gomis J, Lizaur-Utrilla A, Benavidez E, Lopez-Prats FA. No differences between direct anterior and lateral approach for primary total hip arthroplasty related to muscle damage or functional outcome. *International orthopaedics.* 2016;40(10):2025-2030.
55. Brismar BH, Hallert O, Tedhamre A, Lindgren JU. Early gain in pain reduction and hip function, but more complications following the direct anterior minimally invasive approach for total hip arthroplasty: a randomized trial of 100 patients with 5 years of follow up. *Acta orthopaedica.* 2018;89(5):484-489.
56. Parvizi J, Restrepo C, Maltenfort MG. Total Hip Arthroplasty Performed Through Direct Anterior Approach Provides Superior Early Outcome: Results of a Randomized, Prospective Study. *The Orthopedic clinics of North America.* 2016;47(3):497-504.
57. Cao J, Zhou Y, Xin W, et al. Natural outcome of hemoglobin and functional recovery after the direct anterior versus the posterolateral approach for total hip arthroplasty: a randomized study. *Journal of orthopaedic surgery and research.* 2020;15(1):200.
58. Goosen JH, Kollen BJ, Castelein RM, Kuipers BM, Verheyen CC. Minimally invasive versus classic procedures in total hip arthroplasty: a double-blind randomized controlled trial. *Clin Orthop Relat Res.* 2011;469(1):200-208.
59. Abdel MP, Chalmers BP, Trousdale RT, Hanssen AD, Pagnano MW. Randomized Clinical Trial of 2-Incision vs Mini-Posterior Total Hip Arthroplasty: Differences

© 2023 Yan L et al. *JAMA Network Open.*

Persist at 10 Years. *J Arthroplasty.* 2017;32(9):2744-2747.
60. Meneghini RM, Smits SA, Swinford RR, Bahamonde RE. A randomized, prospective study of 3 minimally invasive surgical approaches in total hip arthroplasty: comprehensive gait analysis. *J Arthroplasty.* 2008;23(6 Suppl 1):68-73.
61. Sershon RA, Tetreault MW, Della Valle CJ. A Prospective Randomized Trial of Mini-Incision Posterior and 2-Incision Total Hip Arthroplasty: Minimum 5-Year Follow-Up. *J Arthroplasty.* 2017;32(8):2462-2465.
62. Della Valle CJ, Dittle E, Moric M, Sporer SM, Buvanendran A. A prospective randomized trial of mini-incision posterior and two-incision total hip arthroplasty. *Clin Orthop Relat Res.* 2010;468(12):3348-3354.
63. Hu C-C, Chern J-S, Hsieh P-H, Shih C-H, Ueng SWN, Lee MS. Two-incision versus modified Watson-Jones total hip arthroplasty in the same patients-- a prospective study of clinical outcomes and patient preferences. *Chang Gung medical journal.* 2012;35(1):54-61.

© 2023 Yan L et al. *JAMA Network Open.*

eAppendix 4. Categories and Description of 8 Surgical Approaches in THA

Surgical approaches	Abbreviation	Alternative name
2-incision approach	2-incision	
Direct anterior approach	DAA	1. Modified Smith-Peterson approach[1]<br>2. Hueter's anterior approach[2]
Direct lateral approach	DLA	1. Hardinge approach[3]<br>2. Transgluteal approach[4]<br>3. Bauer approach[4]
Minimally invasive direct lateral approach	MIS-DLA	1. Modified Hardinge approach[5]
Minimally invasive anterolateral approach	MIS-ALA	1. Modified Watson–Jones approach[6]<br>2. OCM approach[6]<br>3. Orthopa¨dische Chirurgie Mu¨nchen approach[6]
Posterior approach	PA	1. Modified Gibson-Moore approach[7]<br>2. K-L approach[8]<br>3. Kocher-Langenbeck approach[8]<br>4. Moore approach[9]
Minimally invasive posterior approach	MIS-PA	
Supercapsular percutaneously assisted total hip arthroplasty	SuperPath	

2-incision[10]

An incision is made directly over the femoral neck from the base of the femoral head distally 1.5 inches to expose the fascia.[11] Another incision is made from the edge of the acetabulum distally to the intertrochanteric line.[11]

For the two-incision technique, the surgical approach involved a 6-cm anterior incision and dissection through the SmithPetersen interval to expose the hip, to cut the femoral neck, and to prepare the socket. A second incision of 3.8 to 5 cm was then made in the buttock, and the abductors and external rotators were identified and were protected with use of a cannula, through which the reamers were placed. The femur was then reamed, and the femoral component was placed through that posterior incision. Intraoperative fluoroscopy was used routinely at key intervals throughout the procedure to verify acetabular reaming depth, acetabular component positioning, femoral alignment for reaming, femoral sizing, and

© 2023 Yan L et al. *JAMA Network Open.*

femoral component positioning.[12]

DAA

For the DAA, the patient is placed in a supine decubitus position. Made over and in the direction of the lateral part of the femoral head and neck and The skin incision was at a point 2 fingerbreadths lateral to the anterior sciatic spine and extended 8–10 cm distally. [13]After division of skin and subcutis, the interval between the tensor fasciae latae muscle and the sartorius muscle is identified and the overlying fascia is opened. In this part of the operation, care was taken to avoid damaging the lateral femoral cutaneous nerve. The intermuscular plane between the tensor fasciae latae and sartorius muscles is developed further down to the hip capsule. Subsequently, the hip capsule is opened, allowing access to the hip joint. Next, osteotomy of the femoral neck, removal of the femoral head, and reaming of the acetabulum is performed. Subsequently, bone cementis pressurized into the acetabular cavity, followed by insertion of the acetabular cup. After reaming of the femur, the femoral component is placed without bone cement, followed by placement of a head on the femoral component, repositioning of the joint and closure in layers.[14]

DLA

The direct lateral approach was performed with the patient in a lateral decubitus position. A straight skin incision, measuring approximately 14 cm, centered over the greater trochanter was used. The subcutaneous tissue and the fascia lata were divided in line with the skin incision. Part of the gluteus medius along with the gluteus minimus was released from the greater trochanter followed by exposure and removal of the anterior part of the joint capsule. The hip was dislocated, and an osteotomy was performed after releasing the capsule down to the lesser trochanter to decide the level of the osteotomy compared with the preoperative template. The head was removed before traditional preparation of the acetabulum using a straight reamer and cementation of the cup. The leg was then placed in external rotation and adduction before opening of the femoral canal, standard preparation of the femoral canal by a straight reamer, and stem implantation.[15]

MIS-DLA

As a minimally invasive approach, if an approach makes any of the following improvements based on DLA, it will be defined as MIS-DLA:

a. The gluteus medius was blunt separated.
b. The incision is approximately (or less than) 10cm.

MIS-ALA (OCM)

In this group, the patient was positioned on the operating table in the supine position and both lower limbs were draped in a sterile fashion. An oblique skin incision measuring 8 to 10 cm was performed, extending distally from the anterior superior iliac spine and ending at the flare of the greater trochanter. After division of the subcutaneous tissue and fascia, the interval between the tensor fasciae latae and the gluteus medius was opened bluntly with the

© 2023 Yan L et al. *JAMA Network Open.*

insertion of a finger. No muscle was split or detached with use of this technique. The acetabulum was prepared in a traditional fashion with use of standard reamers. For preparation of the femur, the distal half of the operating table was lowered approximately 30° and the involved lower limb was placed in external rotation under the contralateral lower limb. In this position, an elevating retractor was placed posterior to the greater trochanter to lever the femur out of the wound. Again, no muscle was detached.[16]

PA

The curvilinear incision 10-15 cm long centres over the posterior third of the greater trochanter. Dissection through the fascia in line with the fibres of gluteus maximus was conducted to reach the short external rotators. With the piriformis muscle identified, the short external rotators (including piriformis, internal obturator muscle, superior gemellus, and inferior gemellus) and hip capsule were tagged and reflected. Subsequent hip joint dislocation was followed by a femoral neck osteotomy at the templated level.[17]

MIS-PA

As a minimally invasive approach, if an approach makes any of the following improvements based on PA, it will be defined as MIS-PA:

c. The quadratus femoris was preserved.
d. The piriformis muscle was preserved.
e. The incision is less than 10cm.

SuperPath

Lateral decubitus position, skin incision of 6–10cm from the tip of the greater trochanter in line with the femoral axis, incision of the fascia of the gluteus maximus muscle, blunt dissection of the fibers, incision of the bursa at the posterior boarder of the gluteus maximus muscle, using the space between the piriformis posterior and the gluteus minimus and medius muscle anterior, incision of the capsule, opening of the femoral canal with a starter reamer, creating a channel in the corticalis of the lateral neck up to the lateral part of the head with a round calcar punch, sequentially broaching of the femur, osteotomy the femoral neck at the tip of the femoral broach left in situ, removal of the femoral head, preparation of the acetabulum, use of a cannula posterior of the femur to pass the reamer drive shaft, connecting the acetabular basket reamer through the main incision, cup impaction and implantation of the inlay, trial modular neck and head, reposition, intra-operative radiograph, test of leg length, impingement and stability, implantation of the definitive components, closure of the capsule, standard wound closure.[18]

Note: One article[19] mentioned anterolateral approach(ALA), but because it did not specifically describe the surgical method, we did not include this approach.

Reference:

1. Michel MC, Witschger P. MicroHip: A minimally invasive procedure for total hip

© 2023 Yan L et al. *JAMA Network Open.*

replacement surgery - A modified Smith-Petersen approach. *Hip International* 2006;16:S40-S47. doi: 10.5301/hip.2009.4852
2. Bon G, Kacem EB, Lepretre PM, et al. Does the direct anterior approach allow earlier recovery of walking following total hip arthroplasty? A randomized prospective trial using accelerometry. *Orthopaedics & traumatology, surgery & research : OTSR* 2019;105(3):445-52. doi: 10.1016/j.otsr.2019.02.008 [published Online First: 2019/03/12]
3. Hardinge K. THE DIRECT LATERAL APPROACH TO THE HIP. *Journal of Bone and Joint Surgery-British Volume* 1982;64(1):17-19.
4. Bauer R, Kerschbaumer F, Poisel S, et al. TRANSGLUTEAL APPROACH TO THE HIP-JOINT. *Archives of Orthopaedic and Trauma Surgery* 1979;95(1-2):47-49. doi: 10.1007/bf00379169
5. D'Arrigo C, Speranza A, Monaco E, et al. Learning curve in tissue sparing total hip replacement: comparison between different approaches. *Journal of orthopaedics and traumatology : official journal of the Italian Society of Orthopaedics and Traumatology* 2009;10(1):47-54. doi: 10.1007/s10195-008-0043-1 [published Online First: 2009/04/23]
6. Bertin KC, Röttinger H. Anterolateral Mini-incision Hip Replacement Surgery: A Modified Watson-Jones Approach. *Clin Orthop Relat Res* 2004;429
7. Zhao HY, Kang PD, Xia YY, et al. Comparison of Early Functional Recovery After Total Hip Arthroplasty Using a Direct Anterior or Posterolateral Approach: A Randomized Controlled Trial. *J Arthroplasty* 2017;32(11):3421-28. doi: 10.1016/j.arth.2017.05.056 [published Online First: 2017/07/01]
8. Tosounidis TH, Giannoudis VP, Kanakaris NK, et al. The Kocher-Langenbeck Approach: State of the Art. *JBJS essential surgical techniques* 2018;8(2):e18. doi: 10.2106/jbjs.St.16.00102 [published Online First: 2018/09/21]
9. Moore AT. The self-locking metal hip prosthesis. *The Journal of bone and joint surgery American volume* 1957;39-a(4):811-27. [published Online First: 1957/07/01]
10. Berger RA. Total hip arthroplasty using the minimally invasive two-incision approach. *Clin Orthop Relat Res* 2003(417):232-41. doi: 10.1097/01.blo.0000096828.67494.95 [published Online First: 2003/12/04]
11. Berger RA. Total Hip Arthroplasty Using the Minimally Invasive Two-Incision Approach. *Clinical Orthopaedics & Related Research* 2003;417(417):232-41.
12. Pagnano MW, Trousdale RT, Meneghini RM, et al. Slower Recovery After Two-Incision Than Mini-Posterior-Incision Total Hip Arthroplasty. *Journal of Bone and Joint Surgery-American Volume* 2009;91A:50-73. doi: 10.2106/jbjs.h.01531
13. Brismar BH, Hallert O, Tedhamre A, et al. Early gain in pain reduction and hip function, but more complications following the direct anterior minimally invasive approach for total hip arthroplasty: a randomized trial of 100 patients with 5 years of follow up. *Acta orthopaedica* 2018;89(5):484-89. doi: 10.1080/17453674.2018.1504505
14. Rykov K, Meys T, Knobben BAS, et al. MRI Assessment of Muscle Damage After the Posterolateral Versus Direct Anterior Approach for THA (Polada Trial). A Randomized

© 2023 Yan L et al. *JAMA Network Open.*

Controlled Trial. *Journal of arthroplasty* 2021 doi: 10.1016/j.arth.2021.05.009

15. Mjaaland KE, Kivle K, Svenningsen S, et al. Do Postoperative Results Differ in a Randomized Trial Between a Direct Anterior and a Direct Lateral Approach in THA? *Clinical orthopaedics and related research* 2019;477(1):145-55. doi: 10.1097/CORR.0000000000000439
16. Pospischill M, Kranzl A, Attwenger B, et al. Minimally invasive compared with traditional transgluteal approach for total hip arthroplasty: a comparative gait analysis. *The Journal of bone and joint surgery American volume* 2010;92(2):328-37. doi: 10.2106/jbjs.h.01086 [published Online First: 2010/02/04]
17. Cheng TE, Wallis JA, Taylor NF, et al. A Prospective Randomized Clinical Trial in Total Hip Arthroplasty-Comparing Early Results Between the Direct Anterior Approach and the Posterior Approach. *Journal of Arthroplasty* 2017;32(3):883-90. doi: 10.1016/j.arth.2016.08.027
18. Quitmann H. Supercapsular percutaneously assisted (SuperPath) approach in total hip arthroplasty : Surgical technique and preliminary results. *Operative Orthopadie und Traumatologie* 2019;31(6):536-46. doi: 10.1007/s00064-019-0597-5 [published Online First: 2019/04/17]
19. Goosen JH, Kollen BJ, Castelein RM, et al. Minimally invasive versus classic procedures in total hip arthroplasty: a double-blind randomized controlled trial. *Clin Orthop Relat Res* 2011;469(1):200-8. doi: 10.1007/s11999-010-1331-7 [published Online First: 2010/03/31]

© 2023 Yan L et al. *JAMA Network Open.*
