# Supplementary material for: Evaluation of Comparative Efficacy and Safety of Surgical Approaches for Total Hip Arthroplasty: A Systematic Review and Network Meta-analysis
Source: JAMA Netw Open. 2023 Jan 31;6(1):e2253942. doi: 10.1001/jamanetworkopen.2022.53942 (PMC9890287; doi:10.1001/jamanetworkopen.2022.53942)
Supplement: Supplement 2. — Data Sharing Statement [file jamanetwopen-e2253942-s002.pdf]

Data Sharing Statement

Yan. Evaluation of Comparative Efficacy and Safety of Surgical Approaches for Total Hip Arthroplasty. *JAMA Netw Open*. Published January 31, 2023.
doi:10.1001/jamanetworkopen.2022.53942

Data
Data available: Yes
Data types: Data (not involving human participants)
How to access data: wangbin_pku@zju.edu.cn
When available: With publication

Supporting Documents
Document types: Statistical/analytic code, Informed consent form
How to access documents: wangbin_pku@zju.edu.cn
When available: With publication

Additional Information
Who can access the data: anyone requesting the data
Types of analyses: for a specified purpose
Mechanisms of data availability: with a signed data access agreement
